# Global pentapeptide statistics are far away from expected distributions

**DOI:** 10.1038/s41598-018-33433-8

**Published:** 2018-10-11

**Authors:** Jarosław Poznański, Jan Topiński, Anna Muszewska, Konrad J. Dębski, Marta Hoffman-Sommer, Krzysztof Pawłowski, Marcin Grynberg

**Affiliations:** 1 Institute of Biochemistry and Biophysics PAS, Warsaw, Poland; 2 in4mates sp. z o.o. sp.k, Warsaw, Poland; 3 Warsaw University of Life Sciences – SGGW, Warsaw, Poland; 4 University of Warsaw, Warsaw, Poland; 5 Fork Systems Konrad Dębski, Duchnice, Poland; 6 Dept. of Translational Medicine, Lund University, Malmö, Sweden

## Abstract

The relationships between polypeptide composition, sequence, structure and function have been puzzling biologists ever since first protein sequences were determined. Here, we study the statistics of occurrence of all possible pentapeptide sequences in known proteins. To compensate for the non-uniform distribution of individual amino acid residues in protein sequences, we investigate separately all possible permutations of every given amino acid composition. For the majority of permutation groups we find that pentapeptide occurrences deviate strongly from the expected binomial distributions, and that the observed distributions are also characterized by high numbers of outlier sequences. An analysis of identified outliers shows they often contain known motifs and rare amino acids, suggesting that they represent important functional elements. We further compare the pentapeptide composition of regions known to correspond to protein domains with that of non-domain regions. We find that a substantial number of pentapeptides is clearly strongly favored in protein domains. Finally, we show that over-represented pentapeptides are significantly related to known functional motifs and to predicted ancient structural peptides.

## Introduction

The oligopeptide composition of known protein sequences is believed to reflect important physico-chemical and biological factors of evolution and has been studied for a long time. From the beginning it was expected that the observed and hypothetical oligopeptide spaces would differ. An early study from 1995, limited by the modest content of sequence databases available at the time and conducted with data from only three organisms, concluded that only 18% of all possible pentapeptides are found in protein sequences^1^.

With the appearance of full proteomes more accurate studies became possible. In 2004 an analysis encompassing 72 full proteomes belonging to three Superkingdoms (Eukaryota, Archaea and Eubacteria) showed differences in the average amino acid and short peptide composition of proteins^2^. Statistically significant preferences were observed even towards particular di- and tripeptides. The relative abundance of specific peptides varied substantially across the superkingdoms, and a detailed analysis showed that even for single organisms the observed occurrences of many di- and tripeptides deviated from the expected values, which were estimated based on the probabilities of organism-specific amino acid usage in the total proteomes.

This result was independently supported by analyses performed for all protein sequences from the non-redundant database^3,4^, where substantial deviations from random distributions were found for tri-, tetra- and pentapeptide sequences. Also the segregation of species by amino acid usage has been confirmed for larger databases: for a collection of 208 proteomes^5^ or 386 proteomes^6^, and for the whole Uniprot database^7^.

In the first study mentioned above^1^, the vast majority of all possible pentapeptides was absent from the databases. This has of course changed with the growing body of sequence data. Only approximately 0.4% possible pentapeptides were absent from the protein sequences of all 72 proteomes studied by Pe’er^2^. The same missing pentapeptides were also identified in another study, encompassing 386 proteomes^8^. Six of these zero-abundance peptides were then synthesized using a Fmoc approach and expressed in an *E. coli* system as parts of soluble proteins, demonstrating that neither steric clashes nor toxicity precluded them from natural proteins^2^. It was observed that some non-existent pentapeptides displayed anticancer activity^9^, and that rare pentapeptides induced a stronger immune response than highly represented pentapeptides^10^, and that in general the information content of an amino acid motif correlates with the motif rarity^11^. On the other hand, a later study, using four different models for the calculation of expected random peptide abundances, showed that for most oligopeptides their frequencies are random and argued that the frequencies of tetra- and pentapeptides are subject to few constraints^12^.

Here, as in many of the studies cited above, we aim to establish if there are any general rules governing the usage of particular pentapeptides in known proteins. In particular, we were interested to see if domain and non-domain protein regions are somehow different in their pentapeptide composition. To investigate this we use pentapeptide permutation groups, i.e. we compare the abundances of individual peptides with the abundances of other peptides that have identical amino acid composition. This allows us to leave out effects connected with different amino acid usage in different organisms. Using this methodology we show that certain pentapeptides are actively favored in the process of evolution, and in particular, a significant group of pentapeptides are favored in regions corresponding to structural protein domains, whereas a separate small group of pentapeptides is favored in non-domain regions.

The reasons why some pentapeptides are more common than others and why there are differences in the pentapeptide composition between domain and non-domain regions, are yet unknown, but they touch the most basic questions in biophysics, with implications for biochemistry and systems biology. In the postgenomic era, with increasing sequencing coverage of the Tree of Life, it is possible to gain a systematic overview of common features of protein sequences found in living organisms, and to formulate universal rules determining protein structures.

## Results

### Determining the abundances of all possible pentapeptides in known protein sequences

We were interested to see if the peptide composition of naturally occurring proteins is random or if it displays any regularities. To test this, we created a dataset of protein sequences by taking the non-redundant NCBI protein database and clearing it of redundancy caused by duplicated entries from different strains of well-studied organisms (throughout this paper, we refer to this dataset as the SQ dataset; see Methods). Then, we counted the number of occurrences N_obs_ of all possible peptides of length 5 that can be built of the 20 standard naturally occurring amino acids (for aggregated results see Table 1). It is important to note that with the current content of sequence databases all possible pentapeptides are represented - none are forbidden.

**Table 1:** Characteristic features of the analyzed pentapeptide categories.

Pentapeptide category	abcde	a_2_bcd	a_2_b_2_c	a_3_bc	a_3_b_2_	a_4_b	a_5_	Total
# different residues	5	4	3	3	2	2	1	
# permutation classes, g	15504	19380	3420	3420	380	380	20	42504
# sequences in each class, m	120	60	30	20	10	5	1	
# sequences in category, g*m	1860480	1162800	102600	68400	3800	1900	20	3200000
SQ								
# peptides	11826966639	10678009933	1474713851	1250343041	137441351	118960963	16794874	25145695663
**DM**								
# peptides	8403194988	7331367618	946224643	761468629	66880015	48819824	2475299	17560431016
% peptides	71.1	68.7	64.2	60.9	48.7	41.0	14.7	69.8
avr. count of peptides per sequence	4517	6305	9222	11133	17600	25695	123765	5488
α = 0.05								
# outlier peptides	493138899	424035844	53363368	42424412	4159480	7698699		1024820702
% outlier peptides	5.9	5.8	5.6	5.6	6.2	15.8		5.8
# sequences with high-abundance outliers	53101	39865	4056	2897	203	248		100370
# sequences with low-abundance outliers	0	1	4	12	8	114		139
# sequences with outliers	53101	39866	4060	2909	211	362		100509
% sequences with outliers	2.9	3.4	4.0	4.3	5.6	19.1		3.1
# classes with no outliers	1268	4079	1298	1622	229	204	20	8720
% classes with no outliers	8.2	21.0	38.0	47.4	60.3	53.7		20.5
α = 0.001								
# outlier peptides	280213162	220140554	26341865	15042352	1528111	5171988		548438032
# sequences with outliers	23395	15522	1445	814	60	159		41395
# classes with no outliers	4442	9749	2314	2714	322	276	20	19837
**ND**								
# peptides	1945203546	1941405338	318589904	302524159	46571312	47671781	9690386	4611656426
% peptides	16.4	18.2	21.6	24.2	33.9	40.1	57.7	18.1
avr. count of peptides per sequence	1046	1670	3105	4423	12256	25090	484519	1441
α = 0.05								
# outlier peptides	65287845	60332804	11852693	10445205	7080780	8322425		163321752
% outlier peptides	3.4	3.1	3.7	3.5	15.2	17.5		3.5
# sequences with high-abundance outliers	45401	31854	3205	2331	255	257		83303
# sequences with low-abundance outliers	0	6	9	19	20	84		138
# sequences with outliers	45401	31860	3214	2350	275	341		83441
% sequences with outliers	2.4	2.7	3.1	3.4	7.2	17.9		2.6
# classes with no outliers	2400	6238	1673	1956	183	215	20	12685
% classes with no outliers	15.5	32.2	48.9	57.2	48.2	56.6		29.8
α = 0.001								
# outlier peptides	29230408	23141426	3649728	2180996	4410942	4902831		67516331
# sequences with outliers	17112	10753	884	535	109	164		29557
# classes with no outliers	6940	12430	2713	2951	276	273	20	25603
**NN**								
# peptides	1309190957	1254681724	190474037	170300897	22748621	21599223	4612762	2973608221
% peptides	11.1	11.8	12.9	13.6	16.6	18.2	27.5	11.7
avr. count of peptides per sequence	704	1079	1856	2490	5986	11368	230638	929
α = 0.05								
# outlier peptides	27845910	27168059	5035864	4734407	3100618	4456107		72340965
% outlier peptides	2.1	2.2	2.6	2.8	13.6	20.6		2.4
# sequences with high-abundance outliers	28667	21968	2487	1822	304	284		55532
# sequences with low-abundance outliers	1	10	10	18	12	93		144
# sequences with outliers	28668	21978	2497	1840	316	377		55676
% sequences with outliers	1.5	1.9	2.4	2.7	8.3	19.8		1.7
# classes with no outliers	4067	8309	1902	2153	146	202	20	16799
% classes with no outliers	26.2	42.9	55.6	63.0	38.4	53.2		39.5
α = 0.001								
# outlier peptides	10275506	8847995	1465607	683925	2104751	2921002		26298786
# sequences with outliers	10017	6975	665	355	156	192		18360
# classes with no outliers	9279	14491	2856	3105	229	259	20	30239

### Testing occurrences of individual pentapeptides using permutation groups and z-scores

The representation of individual amino acids in the sequence database varies substantially. This means that differences in oligopeptide occurrences in the database result in part directly from the different availability of specific amino acids. But in this work, we were interested to analyze differences resulting from other factors. For this purpose, our statistical analysis that compares observed occurrences of oligopeptides should be corrected for their amino acid composition. The correction allowed us to separate the two phenomena: differences in peptide occurrences resulting from variation in amino acid abundance and differences resulting from other factors.

To achieve this kind of correction, we used an approach based on the analysis of the distribution of occurrences of all pentapeptides that share the same amino acid composition. Instead of analyzing individually each peptide from the space of all possible pentapeptides, we clustered them into permutation groups (see Methods). Each group contained all possible permutations of a given amino acid composition. In our analysis, we assume that the expected pentapeptide occurrences should be uniform within each permutation group. This is because in the absence of any evolutionary pressure, all possible permutations should be equally represented in every group. This means that for a pentapeptide *abcde* the number of its occurrences in the database is expected to be close to the number of occurrences of the peptides *abced*, *abdce*, *abdec*, etc. We neglected here the effects of close neighbors, which may cause the permutations to be nonequivalent.

For each pentapeptide in each permutation group, we converted the observed number of occurrences N_obs_ into a z-score (see Methods). The z-score gives us a formal statistical measure of how strongly the N_obs_ of a particular peptide differs from its expected number of occurrences (assuming a binomial distribution of occurrences that implies a common probability for each peptide of the same composition; see Methods). Pentapeptides that differed significantly from their expected numbers of occurrences are further referred to as “overrepresented” or “underrepresented”, accordingly.

### Domain and non-domain regions compared

We then asked how does the pentapeptide distribution differ between defined protein structural domains and other sequences. To investigate this, we constructed three subsets of the SQ dataset (see Methods). One, termed DM, contains all sequences corresponding to known protein domains (included in the CDD database). The second, termed ND, is also derived from proteins that contain CDD domains, but it includes all the sequence regions outside of these domains (these may be interdomain or transmembrane regions, but they may also represent unknown domains). The third set, termed NN, contains all peptides from proteins that are devoid of identified domains. This set served as an internal control that allowed us to ascertain that non-domain proteins do not behave in our analysis differently from non-domain regions. This was necessary because it might be suspected that domain-less proteins include a substantial fraction of artifacts, i.e. protein sequences resulting from wrongly predicted open reading frames. We then determined the number of occurrences of each possible pentapeptide in these sub-datasets (Table 1).

**Figure 1:**
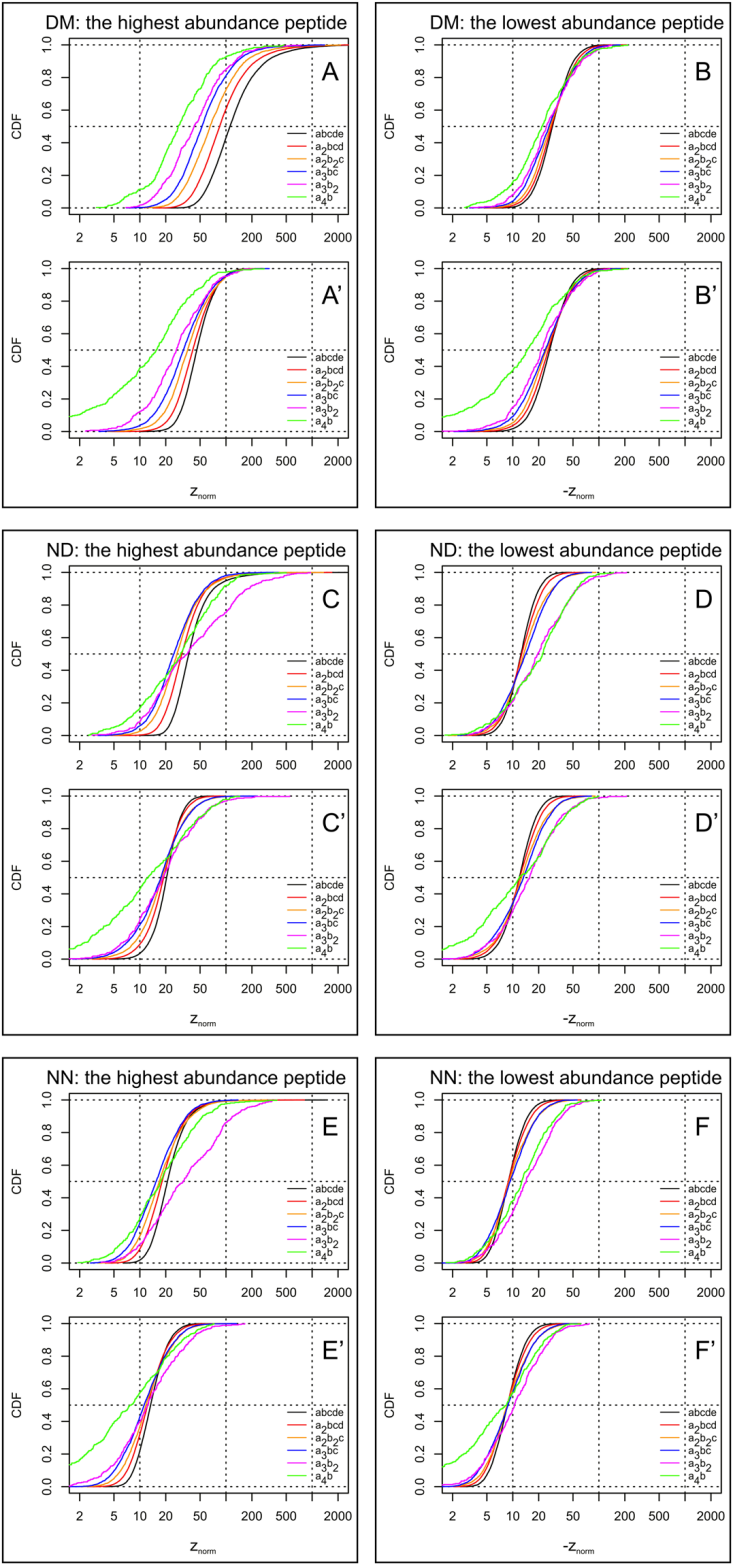
Cumulative distribution functions (CDFs) of the z-score for the most overrepresented (**A,C,E**) and most underrepresented (**B,D,F**) pentapeptides from each permutation class. **A’–F’** represent the CDFs of z_max and z_min for each pentapeptide category - in a manner similar as in **A–F** - but using data where all outlier peptides have been removed from each category. The z-scores are calculated as described in the Methods (“*Analysis of pentapeptide abundances using permutation groups and z-scores*” section). The z_norm_ notation points out that normal distribution is assumed. The distributions are plotted for all permutation classes of the indicated compositions identified in domain regions (**A,B**), non-domain regions (**C,D**), or in proteins with no domains identified (**E,F**). ND – non-domain, DM – domain, NN – peptides from non-domain proteins.

To assess the statistical abundance of a particular peptide in the DM, ND and NN datasets, we calculated the z-scores for all individual pentapeptides. We identified the highest and lowest abundance pentapeptides in each permutation group, and we estimated the associated values of z-scores and termed these z_max and z_min. In Fig. 1, we present cumulative distribution functions (CDFs) of the parameters z_max (Fig. 1A,C,E) and z_min (Fig. 1B,D,F) for each pentapeptide category analyzed in domain (DM) and non-domain regions (ND) and in domain-less proteins (NN), separately. The graphs show that for the majority of peptide classes the observed distributions of their extreme pentapeptides disagree substantially with the expected binomial distributions. For domain regions (DM) the medians of the plotted z-scores for z_max range from 28 for the class a_4_b to over 100 for the class abcde (Fig. 1A), and for z_min values they vary in a range from 22 to 29 (Fig. 1B). For non-domain regions (ND) the distances from the theoretical distributions are smaller, but still substantial: the z-score medians range from above 25 to 40 for z_max values (Fig. 1C), and for z_min from 14 to over 24 (Fig. 1D). Importantly, pentapeptides in proteins with no assigned domains display distributions close to those observed for non-domain regions (Fig. 1E,F vs. Fig. 1C,D), clearly confirming the applicability of the method used for domain identification and suggesting that, generally, domain-less proteins are not qualitatively different from non-domain regions in those with domains.

We analyze here 42,484 different pentapeptide permutation classes extracted from 3 different contexts, (i.e. from domains, DM, from regions outside of domains, ND, and from proteins with no identified domains, NN). Each permutation class consists of 5 (a_4_b) to 120 (abcde) pentapeptide sequences (the 20 trivial classes a_5_ were excluded from the analysis; see Table 1 for details). When applied to this analysis, the Bonferroni correction for multiple comparisons^13^ implies that 1% confidence intervals (CI) for z-scores equal (−5.72; 5.72), (−5.64; 5.64), (−5.20; 5.20), (−5.13; 5.13), (−4.55; 4.55) and (−4.41; 4.41) for the abcde, a_2_bcd, a_2_b_2_c, a_3_bc, a_3_b_2_ and a_4_b permutation classes, respectively. However, the majority of the highest/lowest abundance peptides are outside of the associated CIs, demonstrating that the abundances of pentapeptides within each permutation class disagree with the binomial distribution.

Interestingly, the distributions shown in Fig. 1 also differ qualitatively. For domain regions, we observe that among the most overrepresented peptides the lower the complexity of the peptide composition, the less significant the deviation from the binomial distribution (Fig. 1A; note the a_4_b and a_3_b_2_ groups at the left of the graph). The median of the curve at the far right deviates from the expected distribution by a z-score of >100, indicating that in domain regions there is an extremely strong pressure favoring certain complex peptides. At the same time, among the most underrepresented peptides no substantial effect of peptide complexity is observed (Fig. 1B). This might reflect the fact that the existence of a limited number of strongly overrepresented peptides implies an apparent underrepresentation of some other peptides. For non-domain regions (ND and NN) the situation is different: among overrepresented peptides there is less divergence (however, the observed differences are still statistically significant) and - surprisingly - the low-complexity permutation groups show highest deviation from the expected distribution (Fig. 1C,E), and among the most underrepresented peptides again the low-complexity categories deviate stronger from the binomial distribution than other categories (Fig. 1D,F). This could indicate a possible role of low-complexity sequences within non-domain regions.

In the Supplementary Material (Suppl. Fig. S1A and S1B), we also present individual instances of the data aggregated in Fig. 1.

**Figure 2:**
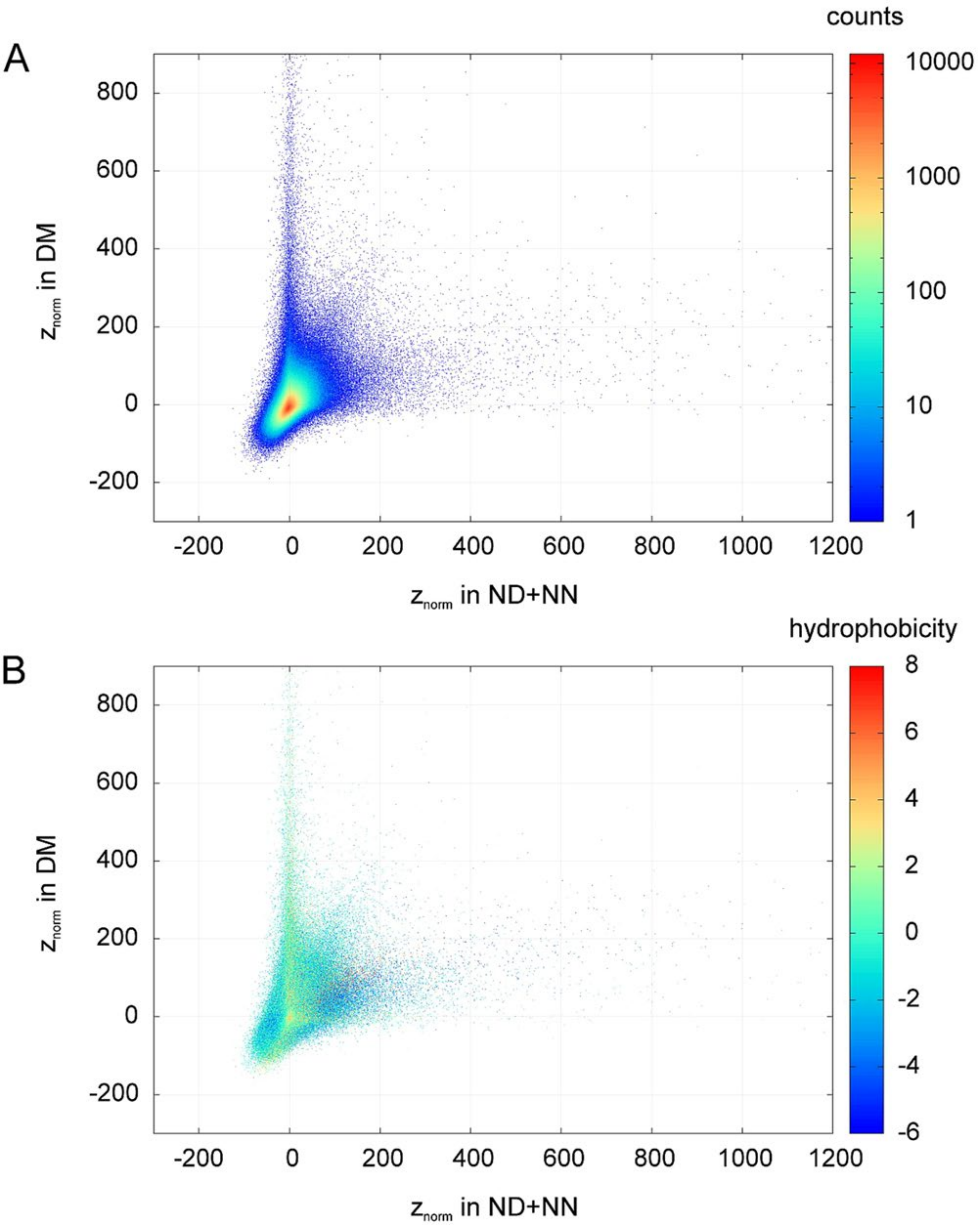
Comparison of z-scores expressing the observed numbers of occurrences of pentapeptides in relation to the expected numbers of occurrences (see Methods, “*Analysis of pentapeptide abundances using permutation groups and z-scores*” section) for domain (DM) and non-domain (ND + NN) regions. The z_norm_ notation points out that normal distribution is assumed. Every point represents a pentapeptide. Colouring by abundance (**A**) or by hydrophobicity scale (**B**)^33^. Arithmetic average is taken when two points overlap. In Fig. 2A, red means many overlapping pentapeptides, blue - single pentapeptides.

Next we attempted to compare the pentapeptide distributions between domain and non-domain regions. Since no important differences were found between the ND and NN datasets in the distributions presented in Fig. 1, the combined dataset ND + NN was used for some of the further analyses, considering it representative for non-domain regions. For every possible pentapeptide, we plotted the z-scores calculated from the DM and ND + NN datasets (Fig. 2). Almost all permutation groups are large enough to justify the use of the normal approximation of the binomial distribution (see Methods). We analyzed 3,200,000 different pentapeptide sequences; assuming a statistical pentapeptide distribution and applying the Bonferroni correction for multiple comparisons, in this analysis only several peptides are expected in this analysis to display z-scores outside of the (−5; 5) range (z = 5 corresponds to a p-value of 2.9·10^−7^). The plots in Fig. 2 show that this is not the case: the vast majority of all pentapeptide sequences are non-statistically distributed (i.e. they lie far away from the (0,0) point). However, the majority of pentapeptides are neither strongly overrepresented nor strongly underrepresented in any dataset - they are relatively close to the (0,0) point. This is clearly visible in panel A (the coloring indicates the number of peptides represented in each dot).

The most interesting feature of the graph in Fig. 2A is the fact that a substantial number of pentapeptides is clearly strongly favored solely in one of the datasets (either DM or ND + NN), while no peptides favored in both datasets were identified. The underrepresented pentapeptides do not display this feature: here the graph shows no tendency towards either the DM or ND + NN axis.

During random evolution, the number of peptide occurrences within a permutation group (N_obs_) is attracted to the expected uniform probability, i.e. to the (0, 0) point in the plots. Any step away from this attractor has to be functionally or thermodynamically justified - indicating the existence of strong evolutionary pressure in domain regions.

We also investigated the relation between peptide abundance and hydrophobicity (Fig. 2B). We noticed that strongly hydrophobic peptides appear to be favored among those overrepresented in ND + NN regions, as well as among those underrepresented in both DM and ND + NN regions. This asymmetric hydrophobicity pattern supports the notion that different functional constraints and pressures function in the evolution of domain and non-domain sequences – balance of subtle short-range interactions between hydrophobic side-chains together with solvation effect may be one of the factors influencing the non-statistical distribution of pentapeptides.

Next we looked whether the distribution of pentapeptides in the human proteome differs from the distribution in the whole dataset (Fig. S2). We note that for human proteins, relatively fewer underrepresented peptides are observed than in the total protein universe (compare Fig. S2 and Fig. 2B), but otherwise the picture is similar to that of all proteomes: we see a subset of pentapeptides strongly favored in DM regions.

### Outliers: far away from other sequences

On one hand, the permutation group approach allowed us to overcome the problem of estimating the significance level of the observed differences. On the other hand, it enabled us to screen every permutation group separately for the existence of outliers, i.e. pentapeptides whose abundance is very different from the overall distribution of their permutation group. To identify outliers in our data, we applied the Grubb’s test, which is widely used in outlier identification (see Methods).

**Figure 3:**
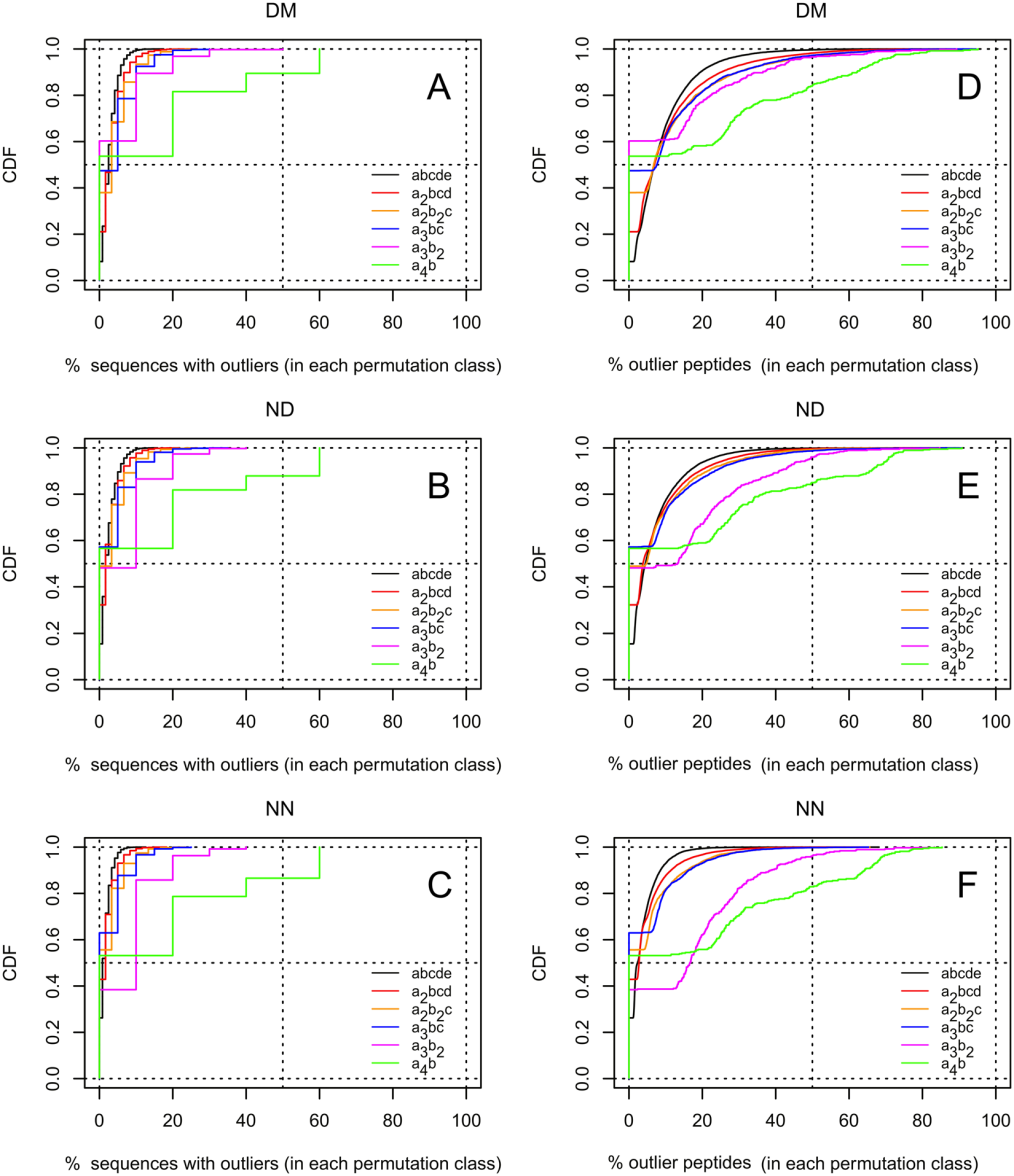
Cumulative distribution functions (CDFs) of the relative contribution of sequences with overrepresented outlier peptides (**A–C**) or underrepresented outlier peptides (**D–F**). The distributions were calculated for each pentapeptide category separately for domain (**A**,**D**) and non-domain (**B**,**E**) regions, and for non-domain proteins (**C**,**F**).

A confidence level of α = 0.05 was used to ensure that the majority of potential outliers were identified in the analyzed distributions. We extracted 100,370 high-abundance outlier sequences from the DM dataset, 83,303 from the ND dataset and 55,532 from NN. This implies that as much as 3.1%, 2.6% and 1.7% of all possible sequences in the DM, ND and NN datasets, respectively, might represent outliers. The outliers are distributed in such a way that the majority of permutation groups contain at least one outlier peptide (see Fig. 3 and Table 1). However, there is a difference in the presence of outliers between domain and non-domain sequences. For the NN dataset, 60.5% of the permutation groups contain outliers, for the ND dataset it is 70.2%, while for the DM dataset, as many as 79.5%. This difference probably reflects the fact that more functional (structure-related) constraints are imposed by evolution on short peptide motifs (only 5 aa in length) when they are part of structural/functional domains. This difference may also be related to the fact that the NN set contains fewer peptides than the other sets (ND and DM), hence fewer outlier pentapeptides can reach significance.

We also searched for low-abundance outliers. At the confidence level of 0.05 we found only 139 low-abundance outliers from 109 permutation groups in the DM dataset, 138 such outliers in the ND dataset, and 144 in the NN dataset. Examples of low-abundance outlier peptides (see also Table 3) include: DVVDD (found 15,871 times in the DM dataset, with 21,716 occurrences expected), CTCTT (found 402 times in domain regions, with 849 occurrences expected), GPGPP (NN dataset, 2337 occurrences; 10,080 expected), GPGPP (ND dataset, 10,793 occurrences; 58,698 expected) and RSSRR (ND dataset, 24,251 occurrences; 51,045 expected). In the Supplementary Figures S3 and S4, we exemplify the CDFs for the permutation classes containing outliers with the highest scores. Figure S3 shows results for the DM dataset and S4 for the ND + NN datasets.

The number of outlier sequences found in a particular permutation class does not significantly depend on the peptide category. Generally, up to 20% of sequences may be regarded as outliers, except for the a_4_b category, where in 10% of the permutation classes the outlier proportion reaches 75% (see Fig. 3A–C). A similar relation is observed for the contribution of outlier peptides, however the simpler the class composition, the higher the number of outlier peptides observed (see Fig. 3D–F). Interestingly, we observed statistically significant differences between the ND and NN versus DM datasets. For the categories abcde, a_2_bcd, a_2_b_2_c and a_3_bc, the relative contribution of outlier peptides is significantly higher in the DM than in the ND and NN datasets. In the a_4_b category the distribution of outliers is almost the same for both datasets, while for the a_3_b_2_ category the contribution of outlier peptides is substantially higher in the ND or NN than the DM dataset. Although we should bear in mind that due to smaller representation the determination of outlier peptides is less sensitive in the ND and NN datasets, the observed differences still indicate that for the two categories with simplest sequences (a_4_b and a_3_b_2_) the ND and NN datasets have relatively more highly overrepresented outlier peptides than the DM dataset.

**Figure 4:**
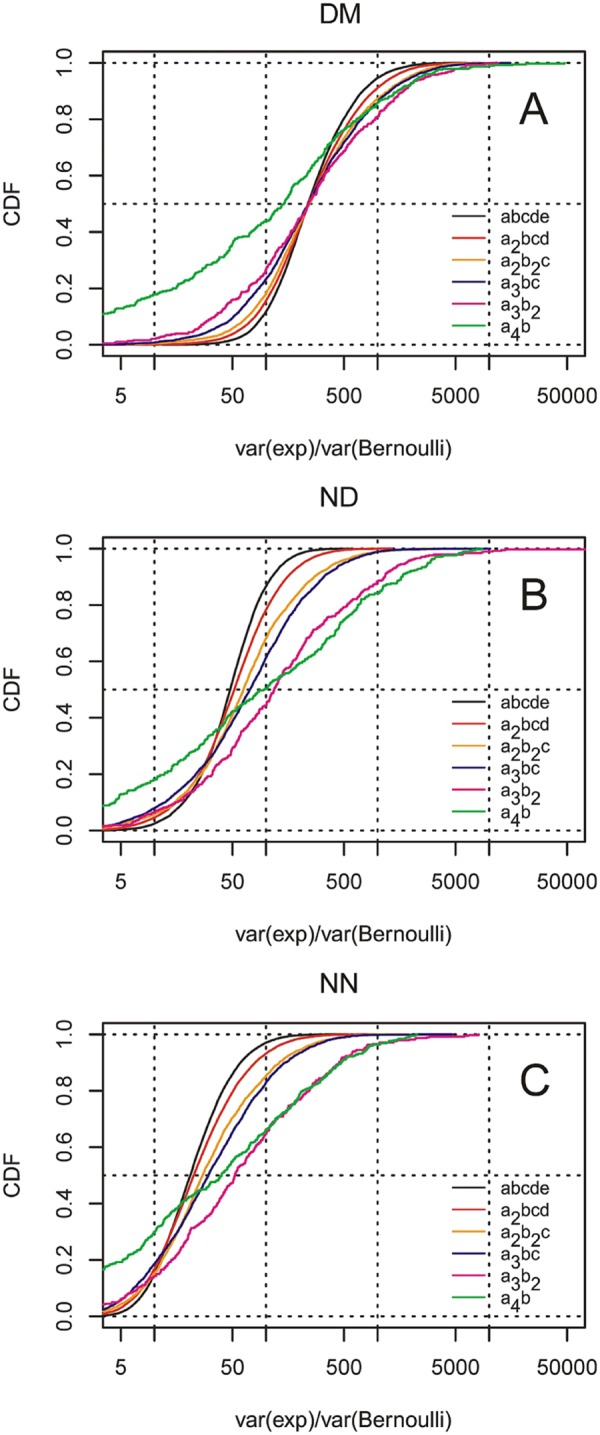
Cumulative distribution functions (CDFs) of the ratio of the variance estimated experimentally within each permutation class (var(exp)) and the value expected for the same data (var(Bernoulli)). The distributions are plotted for all permutation classes of the indicated composition identified in either domain (**A**) or non-domain (**B**) regions, or in regions from non-domain proteins (**C**). The square root of the abscissa value could be roughly interpreted as the number of folds that the observed distribution is wider than the expected distribution.

We next plotted the CDFs of z_max and z_min for each pentapeptide category - in a manner similar as in Fig. 1A–F - but using data where all putative outlier peptides determined at α = 0.05 have been removed from each category. The results are presented in panels A’-F’ of Fig. 1. They show that even after the removal of outliers the pentapeptide distributions are much wider than those expected for binomial distributions. This is true for the whole distributions of peptide occurrences in most groups (also after the correction for multiple comparisons). Consequently, the ratio of observed to expected variance substantially exceeds 1 for most permutation classes (Fig. 4). In the DM dataset, this effect can be observed uniformly in all peptide classes, while in the ND and NN datasets, the simpler the compositions, the higher the observed divergence. This again shows that in non-domain regions there are strong pressures in low-complexity regions.

### Gibbs clustering of the identified outliers

For further analysis, to ensure that all analyzed pentapeptide sequences are true outliers, they were identified at a much more restrictive significance level of 0.001. In this case the Bonferroni correction for multiple comparisons implies that approximately 50 false positives are expected among the 41,395 and 20,796 outlier peptides identified in DM and ND + NN, respectively. The high-abundance outliers identified at the 0.001 level were then grouped according to sequence similarity, using Gibbs sampling clustering (see Methods). The most striking feature in both the DM and ND + NN high-abundance outlier datasets is the large number of peptides containing CxxC motifs, known from oxidoreductases and zinc fingers (Fig. S5).

Other pentapeptides overrepresented both in domain and non-domain regions include further motifs of functional importance (see Table 2). For example, we found several conserved ATP-binding motifs (the P-loop-like G[ST]GK), as well as zinc-dependent metalloprotease active site motifs (HExxH) and the classical kinase active site motif ([HY]RD). The relatively large number of outliers containing motifs corresponding to enzyme active sites in the ND and also in the NN regions suggests that novel, undescribed enzyme families (maybe kinases or metalloproteases) may still lurk in these regions in substantial numbers.

**Table 2:** Examples of known, biologically relevant sequence motifs found in overrepresented outlier pentapeptides.

Sequence motif	Motif function	Number of high-abundance outliers in DM	Number of high-abundance outliers in ND	Number of high-abundance outliers in NN
[HY]RD	protein kinase active site^34^	56	23	14
HExxH	zinc metallo-protease active site^19,35^	41	8	37
G[ST]GK	ATP-binding motif, P-loop-like^34^	18	2	8
CxxC	Zn-binding motif in zinc fingers or oxido-reductase active site^36,37^	1587	680	1085

Many pentapeptides may be abundant for a biological reason. Here, we provide several examples, and further down a systematic analysis of outlier pentapeptides of the type a2bcd is presented, arguing for their functional relevance. Among the most abundant outliers from the ND and NN datasets, GGRGG is a well-known generic arginine methylation motif^14^. This finding may suggest that some of the pentapeptides frequent in ND and NN regions actually represent yet-undescribed functional motifs in domains or regulatory motifs outside of those. Quite unexpectedly, another of the most abundant outliers from the ND dataset (and also significant in the NN dataset), QITLW is found at the N-terminus of the human immunodeficiency virus protease (PR) and is recognized by an antibody blocking this enzyme^15^. The use of such an abundant peptide in a viral protein may have reason in an evolutionary arms race whereby use of a common peptide may be an attempt at mimicry.

Interestingly, among the very few identified low-abundance outliers in the domain regions, AAEAP occurred^16^. This motif is a building block of an outer membrane lipoprotein, designated Lip, present in all tested strains of pathogenic *Neisseria* species. The use of this rare motif by a pathogen may also reflect the evolutionary arms race whereby the pathogen attempts to evade detection, this time by using a very rarely seen epitope.

A summary of the top outlier pentapeptides (ten most underrepresented and ten most overrepresented peptides in DM, in ND and in NN, respectively) is presented as Table 3. Strikingly, the most significant outliers contribute more than 70% of occurrences of their permutation class (as much as 86% for the HPDKW peptide in the ND set).

**Table 3:** Summary of the top outlier pentapeptides.

Protein region	Permutation class	Number of sequences in the class	Total occurrences of the class	Average occurrences of the class	Variance	Sequence	Occurrences of the sequence	%Total	Z score
**Under-represented**									
DM	LLLLS	5	1103203	220641	176512	SLLLL	129826	11.8	−216
DM	AAELR	60	7061680	117695	115733	LEARA	46773	0.7	−208
DM	AELLR	60	5987177	99786	98123	LELRA	35795	0.6	−204
DM	AAAAL	5	2256485	451297	361038	LAAAA	335710	14.9	−192
DM	GGGGI	5	442963	88593	70874	GGGGI	41341	9.3	−177
DM	AAAGL	20	4815784	240789	228750	LGAAA	156132	3.2	−177
DM	AEKLL	60	4064306	67738	66609	LKLEA	24337	0.6	−168
DM	ELLRR	30	2152724	71757	69366	RLELR	29344	1.4	−161
DM	AAALR	20	4377052	218853	207910	LRAAA	145448	3.3	−161
DM	EEKLL	30	1999573	66652	64431	ELKLE	26021	1.3	−160
**Over-represented**									
DM	EGHKT	120	827985	6900	6842	HTGEK	413267	49.9	4913
DM	FMNSW	120	268723	2239	2221	NMSFW	198813	74.0	4171
DM	PTVWY	120	351052	2925	2901	WTVYP	209428	59.7	3834
DM	GKLST	120	3009236	25077	24868	GKSTL	595718	19.8	3619
DM	EGKPY	120	911766	7598	7535	GEKPY	318604	34.9	3583
DM	EGKPT	120	1461844	12182	12081	TGEKP	389313	26.6	3431
DM	GTVWY	120	445075	3709	3678	GWTVY	210756	47.4	3414
DM	EGMWY	120	217167	1810	1795	WMGYE	145689	67.1	3396
DM	FMNPR	120	339635	2830	2807	FPRMN	177146	52.2	3290
DM	FLMSW	120	362542	3021	2996	MSFWL	182120	50.2	3272
**Under-represented**									
ND	GGPPP	10	586979	58698	52828	GPGPP	10793	1.8	−208
ND	AAAPP	10	1239944	123994	111595	APPAA	71014	5.7	−159
ND	AGGGG	5	839291	167858	134287	GAGGG	114103	13.6	−147
ND	DDSSS	10	606514	60651	54586	SDDSS	26740	4.4	−145
ND	GPPPP	5	388310	77662	62130	PGPPP	42721	11.0	−140
ND	DDDSS	10	487092	48709	43838	DDDSS	19800	4.1	−138
ND	GGGRR	10	580934	58093	52284	GRRGG	26697	4.6	−137
ND	AAGGG	10	948949	94895	85405	AGGGA	58015	6.1	−126
ND	RRRSS	10	510453	51045	45941	RSSRR	24251	4.8	−125
ND	RRSSS	10	563675	56368	50731	SRSSR	29134	5.2	−121
**Over-represented**									
ND	DHKPW	120	141538	1179	1170	HPDKW	122449	86.5	3546
ND	DKPTW	120	168175	1401	1390	PDKWT	121326	72.1	3217
ND	KQTVW	120	156893	1307	1297	KWTVQ	116218	74.1	3191
ND	ILQTW	120	171694	1431	1419	QITLW	121321	70.7	3183
ND	DGKMP	120	207222	1727	1712	KPGMD	129015	62.3	3076
ND	DKTVW	120	175850	1465	1453	DKWTV	117182	66.6	3036
ND	GKLMP	120	249781	2082	2064	LKPGM	128663	51.5	2786
ND	ILPQT	120	431365	3595	3565	PQITL	122621	28.4	1994
ND	FIPPS	60	241704	4028	3961	FPISP	110649	45.8	1694
ND	EIPST	120	536102	4468	4430	SPIET	106093	19.8	1527
**Under-represented**									
NN	AGGGG	5	395836	79167	63334	AGGGG	52481	13.3	−106
NN	GGGGN	5	157711	31542	25234	NGGGG	17608	11.2	−88
NN	AAAPP	10	469392	46939	42245	APPAA	29107	6.2	−87
NN	GGPPP	10	100799	10080	9072	GPGPP	2337	2.3	−81
NN	AAGGG	10	484217	48422	43580	AGGGA	32303	6.7	−77
NN	GGGNN	10	126506	12651	11386	GGGNN	4638	3.7	−75
NN	GGGGT	5	151280	30256	24205	GTGGG	18821	12.4	−74
NN	LLQQQ	10	160339	16034	14431	QQLQL	8129	5.1	−66
NN	DDSSS	10	179338	17934	16140	SDDSS	9821	5.5	−64
NN	DDDSS	10	144807	14481	13033	DDSSD	7326	5.1	−63
**Over-represented**									
NN	CEFHK	120	41470	346	343	KHCFE	26831	64.7	1431
NN	CEFHV	120	44399	370	367	HCFEV	27459	61.8	1414
NN	CFHKS	120	45270	377	374	SKHCF	26211	57.9	1336
NN	DESTV	120	348198	2902	2877	TDEVS	48183	13.8	844
NN	CEFVV	60	44179	736	724	CFEVV	22635	51.2	814
NN	HKSSV	60	89154	1486	1461	VSSKH	31133	34.9	776
NN	DEFVV	60	148928	2482	2441	FEVVD	37821	25.4	715
NN	CHKSS	60	34640	577	568	SSKHC	16273	47.0	659
NN	DDERT	60	122092	2035	2001	DRTDE	30087	24.6	627
NN	DERTV	120	255182	2127	2109	RTDEV	30351	11.9	615

Ten most underrepresented and ten most overrepresented peptides in DM, in ND and in NN protein regions, respectively.

It has been well-known for several decades now that functional properties of protein sequences are only interpretable in the context of three-dimensional structures^17,18^. In this study, we have conscientiously decided to focus on sequence properties, to ignore the temptation of mapping the analyses onto structural resources and to keep this work focused as a computational study. Although the structural databases still represent only a fraction of the sequence world and there is a severe bias towards proteins of particular scientific interest and a bias resulting from technical issues (e.g. crystallizability), we decided to perform a limited in-depth analysis of a group of outlier pentapeptides relating to structural and functional properties. For simplicity, we selected pentapeptides of the type a_2_bcd, and focused on the Cys-, His-, Ile- and Leu-containing ones (see Table 4).

**Table 4:** In-depth analysis of the outlier pentapeptides of the type a2bcd.

Focus residue	Protein region	% “aa”	%“axa”	%“axxa”	%“axxxa”	Total counts
A	DM	23	26	22	27	443
A	ND	20	22	18	37	30
A	NN	100	0	0	0	6
C	DM	2	5	82	9	530
C	ND	3	17	44	34	60
C	NN	2	0	73	24	22
D	DM	20	36	22	20	366
D	ND	38	20	20	20	27
D	NN	0	33	66	0	7
E	DM	37	15	31	15	456
E	ND	45	0	42	12	23
E	NN	85	0	14	0	13
F	DM	27	25	20	26	340
F	ND	30	27	23	18	29
F	NN	100	0	0	0	2
G	DM	21	28	28	21	673
G	ND	22	18	50	8	63
G	NN	33	66	0	0	5
H	DM	18	20	15	46	361
H	ND	10	17	10	62	17
H	NN	0	0	0	100	3
I	DM	23	20	28	27	395
I	ND	21	8	27	42	34
I	NN	0	0	0	100	1
K	DM	31	20	25	22	330
K	ND	41	21	20	16	35
K	NN	56	24	0	18	17
L	DM	18	12	30	37	511
L	ND	36	9	41	11	46
L	NN	50	0	16	33	8
M	DM	18	17	30	33	231
M	ND	7	13	39	39	24
M	NN	0	0	33	66	2
N	DM	24	24	21	29	296
N	ND	20	53	26	0	11
N	NN	0	100	0	0	3
P	DM	15	21	31	32	464
P	ND	15	20	38	25	44
P	NN	20	0	0	80	2
Q	DM	34	28	19	18	227
Q	ND	10	28	17	42	29
Q	NN					0
R	DM	30	29	28	11	383
R	ND	31	47	7	14	21
R	NN	0	33	33	33	6
S	DM	30	21	27	20	278
S	ND	29	12	38	19	13
S	NN	63	0	36	0	9
T	DM	21	29	26	23	376
T	ND	11	49	15	23	32
T	NN	0	57	0	42	5
V	DM	31	30	18	19	375
V	ND	7	28	21	42	19
V	NN	57	21	0	21	15
W	DM	23	38	19	18	305
W	ND	28	28	14	28	27
W	NN					0
Y	DM	28	20	25	25	282
Y	ND	42	22	0	34	8
Y	NN	0	0	0	100	2

For every “focus residue” a, the highest abundant outliers (z > 100) were considered that contained exactly two occurrences of the focus residue. Then, occurrences were considered where the focus residue was separated by 0, 1, 2 or 3 residues.

For example, among permutation classes of the type L_2_bcd consisting of exactly two leucine residues, the majority of DM region outliers were of the type LxxxL (37%) and LxxL (30%) while LxL (12%) and LL (18%) were far less common. This is very different from preferences for Ile-containing outliers and clearly indicates that in DM regions, LxL or LL organization is much less favoured than LxxxL and LxxL. This likely reflects preference for peptides building leucine zippers (leucine residues located in inter-helical hydrophobic interface, e.g. in coiled-coil structures) and proves that chemically very similar residues (Leu and Ile) are subject to different evolutionary pressures, most likely related to their structural properties. Another interesting example are His-containing peptides. Here, the HxxxH species is most common, and this requirement is most pronounced in the ND and NN sequence sets. The HxxxH motif corresponds to metal ion binding sites, such as in metalloprotease active sites, and its abundance in non-domain regions may indicate presence of yet-unidentified metalloproteases or metallo-motifs^19^. Then, Cys-containing peptides show a striking preference for CxxC motifs, more pronounced for the DM set (82%) but also preferred in ND and NN (44% and 73%, respectively). This motif is characteristic of oxidoreductase active sites and zinc fingers. This again points at possible numerous functional motifs in the non-domain regions that are typically not functionally characterized yet.

### Mapping the overrepresented outlier pentapeptides onto biologically relevant motifs

We analyzed the relationship between the overrepresented pentapeptides elucidated in this study and three special datasets: 1) the Prosite database of protein functional motifs^20^, 2) the ELM database of short linear motifs involved in protein-protein interactions^21^ and 3) the set of ancient structural peptides described in a recent paper by Andrei Lupas and co-workers^22^.

**Figure 5:**
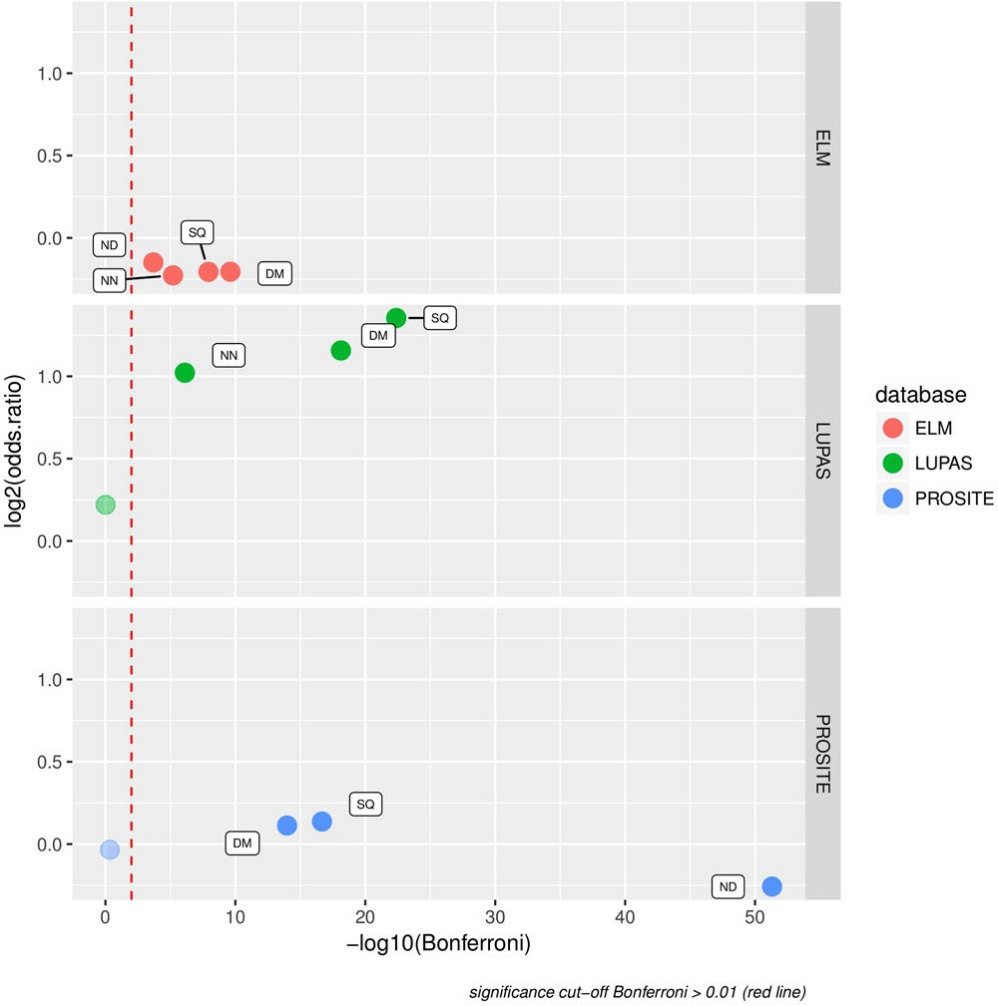
Overrepresented outlier pentapeptides relate to functional motifs. Relationships between overrepresented outliers and Prosite functional motifs (blue), ELM protein-protein interaction motifs (red) and predicted ancient structural peptides from Lupas *et al*.^22^ (green). Fisher’s exact test was applied. P-value with Bonferroni correction is shown. Non-significant datapoints are dimmed. The Y axis shows log2 of odds ratio, hence value of 1 corresponds to 2-fold odds ratio. The dashed red line indicates the significance threshold (P-value = 0.01).

The Fisher’s exact test allowed us to assess whether, for example, outlier pentapeptides overrepresented in protein domains (DM) were significantly more likely than other peptides to match the Lupas ancient peptides. This was indeed the case (green symbols in Fig. 5, two-fold overrepresentation) which strongly supports the idea that pentapeptides overrepresented in protein domains correspond to ancient structural motifs. Interestingly, also pentapeptides overrepresented in domain-less proteins (NN) are significantly likely to match the ancient peptides. This may indicate that a substantial fraction of the “domain-less” proteins may contain yet-undescribed protein domains.

For the ELM database, the relationship to overrepresented outlier peptides is different. The pentapeptides overrepresented in protein domains (DM), in non-domain regions (ND) and in domain-less proteins (NN) are very significantly unlikely to correspond to ELM motifs (red symbols in Fig. 5). These observations may be suggestive that the ELM protein-protein interaction motifs are specific and do not utilize very common pentapeptides^21^. Thus, although structural motifs are known to be important for predicting protein-protein interactions^23,24^ the need for specificity within the interaction interfaces could possibly require such motifs to be built of uncommon sequences.

Lastly, the outlier pentapeptides overrepresented in domain regions (DM) and those overrepresented in the whole sequence set (SQ) are very significantly likely to match Prosite functional motifs (blue symbols in Fig. 5) albeit the overrepresentation is not high in relative numbers. This plays well with the notion that frequent pentapeptide motifs will often play functional roles. It has to be borne in mind that Prosite motifs are by large based on experimental functional (e.g. biochemical) characterization of proteins and often correspond to active sites while the Lupas ancient peptides are derived from structure analyses. Thus, for outlier ND peptides (from non-domain parts of domain-containing proteins) compared versus Prosite database underrepresentation is not unexpected – Prosite motifs are typically identified in well-studied and described domains. The fact that the outlier pentapeptides overrepresented in non-domain regions (ND) are actually *unlikely* to match Prosite motifs could also reflect the fact that the Prosite database is focused on functional motifs discovered previously within known protein domains. The explanation of this effect is not obvious, however, one might speculate that functional motifs in ND regions may differ from those in known domains. Since Prosite motifs can be expected to occur within known protein domains, one might also presume that if recognizable Prosite motifs were present in ND regions, then the presence of those motifs would have allowed the surrounding sequence regions to be assigned to known domains, hence the motif would have ended up in a DM region.

### Most common amino acids in the high-abundance outlier pentapeptides

The most common amino-acids in the high-abundance outliers are Cysteine (C), Tryptophan (W), Methionine (M) and Histidine (H). This is interesting when we take into account the fact that these amino acids are the *least* frequent in the overall space of sequences in the Uniprot database (as summarized by the “Amino acid scale: Amino acid composition (%)” in the UniProtKB/Swiss-Prot data bank). All these amino acids are also biologically unusual: i) Tryptophan is a scarce resource, hard to synthesize, ii) Cysteine is used for disulfide bonds and ion coordination, iii) Methionine is the universal starting amino-acid and iv) Histidine often contributes to enzymatic active sites and to ion coordination. This non-random amino acid composition of highly overrepresented peptides may indicate that there is non-random placement of certain important components in a well-defined order and structural context (i.e. fixed in the term of a protein sequence) for full functionality.

It has to be stressed that the issue of enrichment of outliers in unexpected features (amino acids) has been resolved by our *permutation-based approach* which corrects for bias linked to different occurrence frequencies of amino acids in nature. For example, the most abundant outlier from the DM set, the HTGEK pentapeptide (see Table 3), provides 49.9% of all occurrences of the pentapeptides of the same amino acid composition. Hence, it is not only the use of relatively rare amino acids that distinguishes the outlier pentapeptides, it is also the specific sequence order and neighbourhood of those.

**Figure 6:**
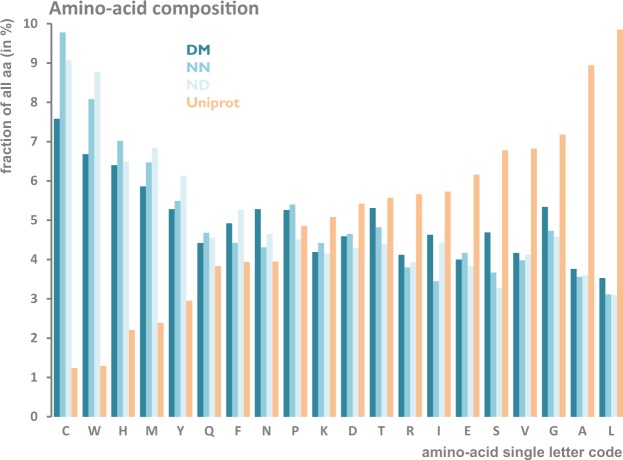
Frequency of each of the 20 canonical amino-acids in all UNIPROT proteins compared to frequency among high-abundance outlier pentapeptides identified in this work.

The three datasets of high-abundance outliers identified at the 0.001 significance level - in domain (DM),non-domain (ND) regions as well as in domain-less protein (NN) - differ substantially in terms of amino acid frequencies. The highest differences were noted for Cysteine (9.78% in NN, 9.07% in ND and 7.58% in DM), Tryptophan (8.08% in NN, 8.77% in ND and 6.68% in DM) and Serine (4.69% in DM, 3.67% in NN and 3.28% in ND). Still, despite these differences, Cysteine and Tryptophan are among the most abundant amino acids in both groups. And the differences between the two datasets of outliers are smaller than the difference between the outlier groups and the Uniprot data, as shown in Fig. 6. This pattern can be explained by the extreme nature of outliers regardless of their position in the protein sequence.

### Phylogenetic spread of high-abundance outlier pentapeptides

The dataset studied (SQ) was mapped to unique NCBI Taxonomy identifiers at the species level (taxids): Eukaryota, 362596 species (84.4%), Bacteria, 48015 species (11.2%), Viruses 13455 species (3.1%) and Archaea, 2805 species (0.65%).

**Figure 7:**
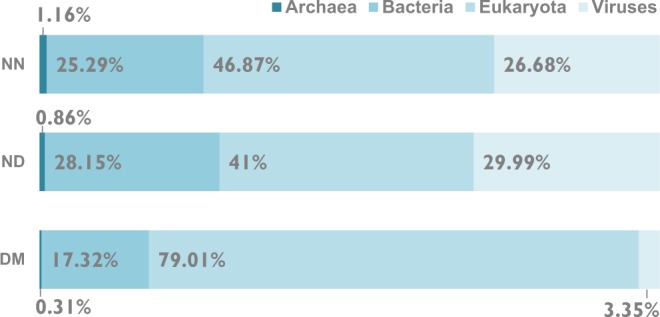
Taxonomic composition of all high-abundance outlier pentapeptides split into domain regions (DM), regions outside of domains (ND) and domain-less proteins (NN).

In the DM dataset, we identified 41,395 high-abundance outlier peptides in proteins from 424,454 different species. In the NN dataset, we found 18,343 high-abundance outliers in proteins from 372,361 different species. In the ND dataset, we found 29,544 high-abundance outliers in proteins from 378,124 different species. The overall taxonomic composition of the outlier datasets is very similar due to the prevalence of shared taxa, only 0.2% of taxa are specific to ND outliers, 0.1% are specific to NN outliers and 4.99% to DM outliers. In both datasets Eukaryotic sequences dominated: 85.2% of the DM outliers and 85.7% of ND and 85.6% of NN outliers represented eukaryotic sequences. There is however a difference in the taxonomic composition of the outlier groups that are specific for DM, ND or NN regions (Fig. 7). The group of outliers specific for DM regions has a similar composition to the overall outlier taxonomic composition, with the dominance of Eukaryota (79.01%) followed by Bacteria (17.32%). The ND and NN specific outliers are also dominated by Eukaryota (46.87% in NN, 41% in ND), but Bacteria (25.29% in NN, 28.15% in ND) and Viruses (26.68% in NN, 29.99% in ND) comprise together more than half of the dataset.

**Figure 8:**
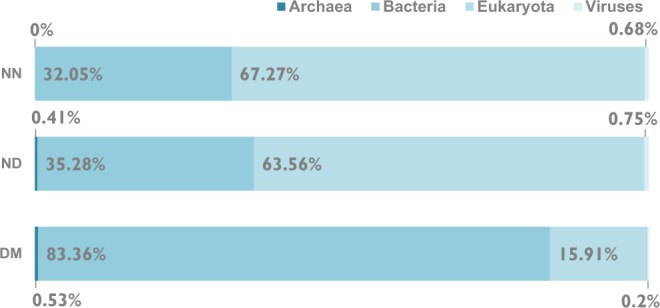
Taxonomic distribution of taxa with more than 20,000 copies of high-abundance outlier pentapeptides. datasets labeled, ND (regions outside of domains), NN (domain-less proteins) and DM (domains) show that the difference in not in the taxa with the highest number of overrepresented pentapeptides.

When only organisms with more than 20,000 high-abundance outliers were considered, a clear difference was observed in the taxon distribution between the DM, ND and NN datasets (Fig. 8). For the DM dataset, there were 6,437 organisms with more than 20,000 overrepresented peptides, and of these 5,367 were bacteria and 1,024 were eukaryotes.

For the ND dataset, there were only 1,457 organisms with more than 20,000 overrepresented peptides, and of these the majority were eukaryotes (926 taxa) and only 514 were bacteria. There were 1,320 organisms with more than 20,000 NN overrepresented pentapeptides of which 888 belonged to Eukaryota and 423 to Bacteria. All taxa with more than 20,000 high-abundance ND and NN outliers had also more than 20,000 DM outliers (Fig. 8). This abundance of outlier sequences in Eukaryota might be related to a difference in codon usage between the domains of life, and to the fact that protein composition in Eukaryota is more strongly influenced by protein-protein interactions. In principle, the whole SQ datasets could also be built separately for different taxa, e.g. for the four kingdoms: Archaea, Bacteria, Eukaryota, and viruses. Such an analysis, although potentially very interesting, would have to be carefully performed in order to avoid the bias present in the quantitative representation of different species and higher taxonomic units in the sequence databases.

## Discussion

In this paper we are exploring the pentapeptide composition of known proteins. Previous studies of this kind have found that many pentapeptides were completely absent from known proteomes^1,2,8^. Now we show that – due to the increased number of sequences available for analysis – no “forbidden pentapeptides” exist anymore. The sequencing coverage of living organisms has reached a point where all possible pentapeptides can be found in known protein sequences.

However, we argue here that more important than *absolute* frequencies of particular peptides are their *normalized* frequencies, i.e. frequencies corrected for amino acid usage. To investigate these normalized frequencies, we use pentapeptide permutation groups, and using this methodology we show that it is not a small subset of peptides that are more or less abundant than statistically expected, but instead the whole observed distributions differ strongly from the expected distributions.

We also identified numerous outlier sequences, i.e. sequences that are very far away from the overall distributions of their permutation groups. The outliers are important because they represent cases where strong evolutionary pressure could be present. And, accordingly, they contain many rare amino acids and they often correspond to known protein sequence motifs, as shown by the comparison of high-abundance outliers from the analyzed datasets to the functional protein motifs from the Prosite database. Our results show that the outlier sequences are useful, very likely all of them perform some specific functions.

It is important to note that even after leaving out all outliers, the overall observed distributions of pentapeptides still differ strongly from the theoretical expected distributions. This means that these distributions are highly “non-statistical”: rare permutations are much more rare than expected, while frequent ones are more frequent than expected. This could be partly related to the fact that we ignored close-neighbor effects, which may cause the permutations to be nonequivalent. Still, this observation warrants a functional explanation.

The most interesting results appear when comparing known protein domains with non-domain regions. Domains contain many times more outliers than non-domain regions, and there are also qualitative differences between both types of regions: in the domain dataset, the strongest pressures are visible for the most complex pentapeptides (composed of three or more different amino acids), while in all non-domain regions the least complex pentapeptides (composed of only two different amino acids) are subject to strongest pressures. Further, the high-abundance outliers derived from known protein domains correspond very often to the sequences of predicted ancient structural peptides^22^, while neither domain nor non-domain high-abundance outliers match protein-protein interaction motifs from the ELM database. These results show that although all types of outliers are subject to strong pressure, the characteristics of overrepresented peptides from domain and non-domain regions will be different. Future work should cast light on their detailed structural, kinetic and thermodynamic properties.

Additionally, our results indicate that the probability for a peptide to be favored in domain regions is much higher than in non-domain regions, which is consistent with the notion that domain and non-domain regions of protein sequences are subject to different functional constraints and pressures. In particular, the most extremely overrepresented peptides (with z-scores above 200) are overrepresented either within domains or – less often – outside of them, and only very rarely in both types of regions simultaneously. Thus, overabundance seems to appear in evolution in response to some kind of pressure: structural, dynamical, or – most likely – functional. Although ultimate confirmation of functional importance of the outlier pentapeptides will require experimental studies, the sequence signals observed by us are statistically extremely significant, and thus are unlikely to be caused by bias of any sort but possibly reflect evolutionary pressure.

## Materials and Methods

### Sequence database preparation

The non-redundant (NR) database of protein sequences was obtained from NCBI in September 2016. Each sequence was mapped on the NCBI taxonomy and assigned a taxonomy identifier and a species level identifier, if these were not available, it was labeled as unknown. First, the NR database was clustered – for each species separately – using CD-HIT^25^ with sequence identity set to 100% and length similarity set to 90% of the shorter sequence. This was done in order to remove duplicates, i.e. identical sequences from different strains of well-studied taxa (e.g. model organisms, such as *Escherichia coli*). Next, known protein domains from the CDD database version 3.15 (June 2016)^26^ were mapped onto the unified database using the RPS-Blast + tool^27^, with an e-value threshold of 0.001.

### Identification of all short peptides (up to 5 residues) in the database

The database described above was used to determine the abundance of all possible short peptides in all known protein sequences. In particular, we analyzed the differences in peptide composition between known protein domains and interdomain regions.

Using an in-house script, we scanned all sequences in the database in order to determine the abundance of all possible peptides of length ranging from 1 to 5. We first scanned the entire database, and in this way we obtained a list showing the number of occurrences in the database of each possible peptide (we termed this list SQ). Then we constructed three subsets of the database: one that includes only sequences corresponding to known domain regions, a second one consisting of all regions found outside of any known domains (these sequences represent either interdomain regions or unknown domains), and a third one grouping proteins without any domains; we scanned these subsets in the same manner as the entire database. In this way we obtained three more lists, showing the number of occurrences of each peptide in known domain regions (this list we termed DM), in non-domain regions (ND) and in non-domain proteins (NN). In our analysis, a peptide occurrence is only then counted if the peptide fits entirely in the analyzed region – thus, for a given peptide, the count in the SQ list is usually larger than the sum of counts in the DM, ND and NN lists. Transmembrane regions were not treated separately. They were either included in the domain regions (if they belonged to known domains) or they were included in the non-domain regions. For some analyses, the combined dataset ND + NN was used, to represent all non-domain regions.

### Analysis of pentapeptide abundances using permutation groups and z-scores

In order to separate peptide abundances from the abundances of the individual amino acids that constitute them, we decided to analyze abundances in permutation groups. For this purpose, the set of all possible pentapeptides (20^5^ = 3,200,000 pentapeptides) was clustered into 42,504 permutation groups, so that each group consisted of all pentapeptides that share the same amino acid composition – i.e. those that are permutations of a single sequence.

Since the same amino acid residue may come up more than once in a given peptide, we have seven different categories of pentapeptides: a_1_b_1_c_1_d_1_e_1_, a_1_b_1_c_1_d_2_, a_1_b_2_c_2_, a_1_b_1_c_3_, a_2_b_3_, a_1_b_4_ and a_5_ (where a-e stand for any, but different from each other, amino acid residue). Each category includes many different groups, e.g. the category a_1_b_4_ includes the permutation groups A_1_W_4_, W_1_A_4_, A_1_T_4_, etc. Each group includes all individual peptides that are permutations of the given composition, e.g. A_1_W_4_ has five permutations: AWWWW, WAWWW, WWAWW, WWWAW, WWWWA. Table 1 shows the number of groups in each category, the number of permutations in each group, and the total number of different pentapeptides in each category.

The number of occurrences of an individual peptide (N_obs_) in a group can be viewed as the result of a classical Bernoulli experiment, B[n,p], with probability p = 1/m (where m is the number of permutations in the group, i.e. the number of possible sequences corresponding to the given amino acid composition) and number of trials n (which stands for the total number of occurrences of all pentapeptides of a given composition identified in the analyzed subset of the database). In terms of the probability theory, the random trial in this experiment corresponds to the procedure of pentapeptide sequence shuffling, while the sample space is the set of all possible pentapeptide sequences of a given composition. (The sequence shuffling here is orthogonal to what is really happening in the course of evolution, i.e. to residue replacement). Further, since the samples in this experiment are large, the normal approximation with μ = n∙p and σ^2^ = n∙p∙(1-p) could be applied^28^. Hence, for each peptide, the number of its occurrences N_obs_ could be scored statistically using the expected value μ, and each N_obs_ could also be examined by its (dis)agreement with the value μ. For example, N_obs_ may be the number of occurrences of the peptide AWWWW, while n would be the number of occurrences of all peptides of the A_1_W_4_ group, and m = 5 would be the number of possible permutations in this example.

The null hypothesis stating that *the observed distribution of pentapeptides sharing the same amino acid composition is random* can be thus tested against the expected binomial distribution. In order to test the hypothesis, we estimated the probabilities that the number of occurrences of the most over- and underrepresented pentapeptides in each group could be regarded as following the binomial distribution B[n,p], in which n is the total number of peptide occurrences in the tested permutation group, and p =1/m, where m is the number of different pentapeptides in the group. We screened 42,484 permutation groups (starting from all possible 42,504 groups but excluding the 20 trivial a_5_ groups). Almost all of these groups are large enough to justify the use of the normal approximation (N) of the theoretical binomial distribution (B): B[n,p]~N[n∙p, n∙p∙(1-p)]^28^. Hence, the observed number of occurrences N_obs_ of any peptide can be converted to the corresponding z-score according to the following equation:

$${\rm{z}}=({{\rm{N}}}_{{\rm{obs}}}-{\rm{\mu }})/\sigma =({{\rm{N}}}_{{\rm{obs}}}-{\rm{n}}\cdot {\rm{p}})/\mathrm{sqrt}({\rm{n}}\cdot {\rm{p}}\cdot (1-{\rm{p}}))$$

We calculated the z-scores for all observed pentapeptides in the datasets. The extreme values, corresponding to the most overrepresented and the most underrepresented pentapeptide in each permutation group, we denoted by z_max and z_min. It should be noted that even for groups consisting of rare amino acids, the value of n∙p∙(1-p) always exceeded 10, which confirms the applicability of the normal approximation.

### Identification and analysis of outlier pentapeptides

For each permutation group, possible outliers were identified iteratively, according to the Grubb’s test with the significance level α = 0.05 for filtering, and 0.001 for outlier identification^29^.

The rigorous Bonferroni correction for multiple comparisons was used to adjust significance levels or to define appropriate confidence intervals^13^.

For sequence-based clustering of the thousands of identified outlier pentapeptides, the GibbsCluster Server^30^ was used. The number of clusters allowed was set to the maximal value (10). An extra “trash” bin was used for pentapeptides that didn’t match any of the clusters.

In order to identify well-annotated proteins that harbour certain selected outlier pentapeptides, we used the ScanProsite tool^31^ on the SwissProt database.

### Relating overrepresented pentapeptides to functional and structural motifs

Overrepresented outlier pentapeptides from the DM, NN and ND sets (identified at significance level of 0.001) were analyzed in order to test whether they are over- or underrepresented in: (1) the set of ancient structural peptides defined by Lupas and co-workers^22^, (2) the ELM database of functional motifs^21^ and (3) the Prosite database of functional motifs^20^.

Significance was estimated with the two-sided Fisher’s exact test. Contingency tables were constructed according to the scheme where pentapeptides in the tested group (e.g. overrepresented DM outliers) were analyzed vs all other pentapeptides within and outside of the tested dataset (e.g. ELM). P-values were adjusted separately in all three groups with the Bonferroni correction; adjusted p-values of less than 0.01 were considered significant. Motifs from the ELM and Prosite databases were converted into 5-character long regular expressions. The obtained unique regular expressions were considered only if they met the following criteria: 1) no more than two positions with undefined amino acid residues (e.g. AAxxA or AAxAA but not AxxxA) and 2) no more than one additional position in which one of no more than five amino acids can fit (e.g. [ACDEF] but not [ACDEFG]). This means that the regular expressions considered could match at most 2000 pentapeptides. Thus, the most generic motifs were discarded from the analysis. The regular expressions used for the analysis cover 90.76% of motifs from the Prosite database (1188/1309) and 60% of motifs from the ELM database (159/265). They were used to scan all pentapeptides. The Lupas set of ancient structural peptides was prepared using the supplementary data for the paper by Lupas and co-workers^22^. The 305 peptide sequences representing fourty “primordial fragments” and five “B-set fragments” described by Lupas *et al*. were scanned for identical substring matches against all pentapeptides.

All analyses were performed in R version 3.3.2^32^ with homemade scripts. Plots were generated with the ggplot2 (2.2.1) and ggrepel (0.7.0) packages.

## Electronic supplementary material

Dataset 1
