# Supplementary material for: Global pentapeptide statistics are far away from expected distributions
Source: Sci Rep. 2018 Oct 11;8:15178. doi: 10.1038/s41598-018-33433-8 (PMC6181984; doi:10.1038/s41598-018-33433-8)

# Global pentapeptide statistics are far away from expected distributions

---

Jarosław Poznański*, Jan Topiński*, Anna Muszewska, Konrad J. Dębski, Marta Hoffman-Sommer, Krzysztof Pawłowski**, Marcin Grynberg**

**Supplementary Figure S1.** Individual instances of the data aggregated in Fig. 1

**Supplementary Figure S2.** N observed vs N expected ratios (see Methods), for S2: domain and non-domain regions. Colouring by hydrophobocity scale (Guy, 1985) arithmetic average is taken when two points overlap. As in Fig. 2B., but human proteins only

**Supplementary Figure S3.** Cumulative distribution functions (CDFs) of pentapeptide occurrences for selected permutation groups for domain regions (DM). For each group, all permutants are shown.

**Supplementary Figure 4.** Cumulative distribution functions (CDFs) of pentapeptide occurrences for selected permutation groups for non-domain regions (ND+NN). For each group, all permutants are shown

**Supplementary Figure 5.** A. Gibbs clustering, DM outliers. B. Gibbs clustering, ND+NN outliers.

Suppl. Fig. S1A
legend on last page

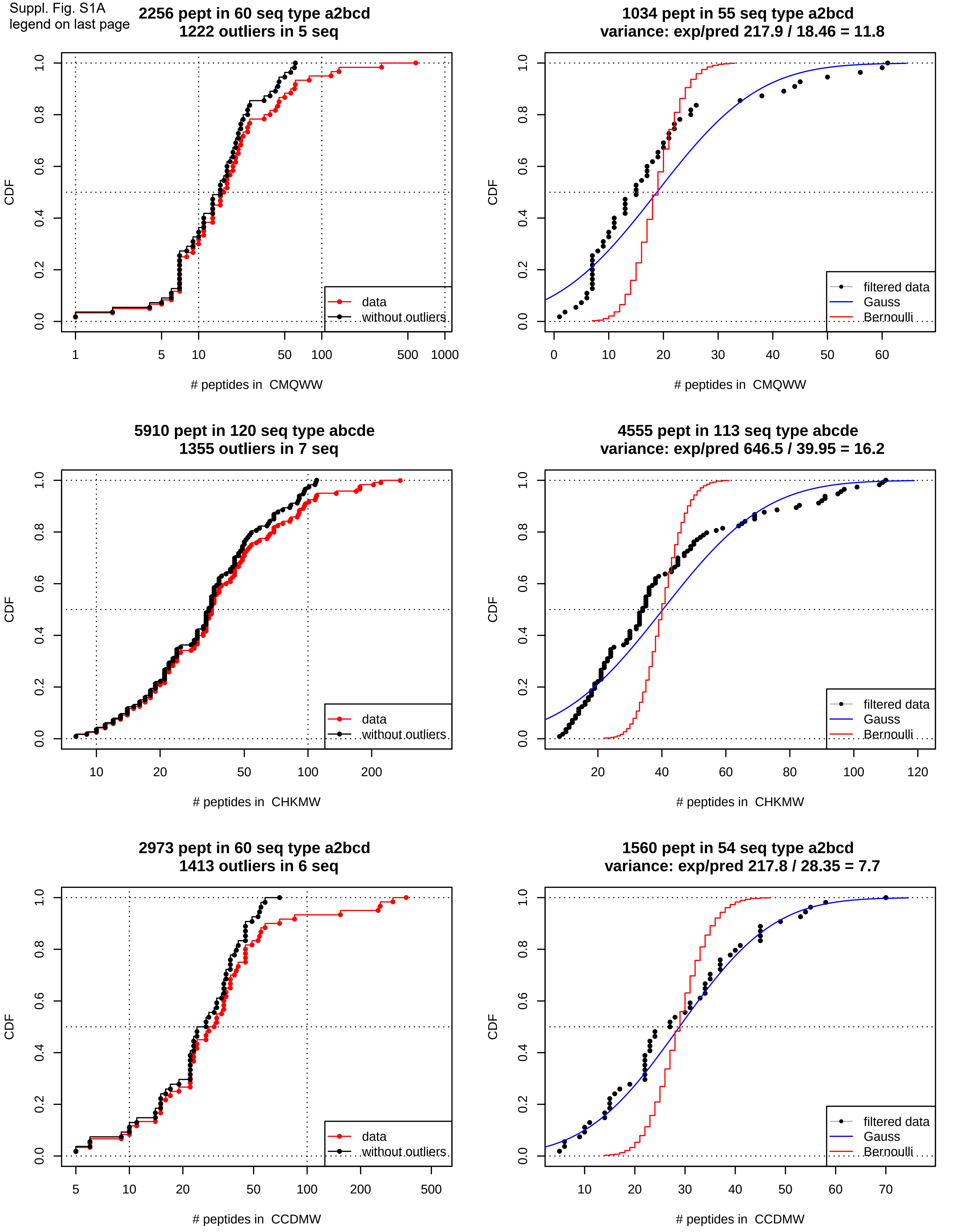

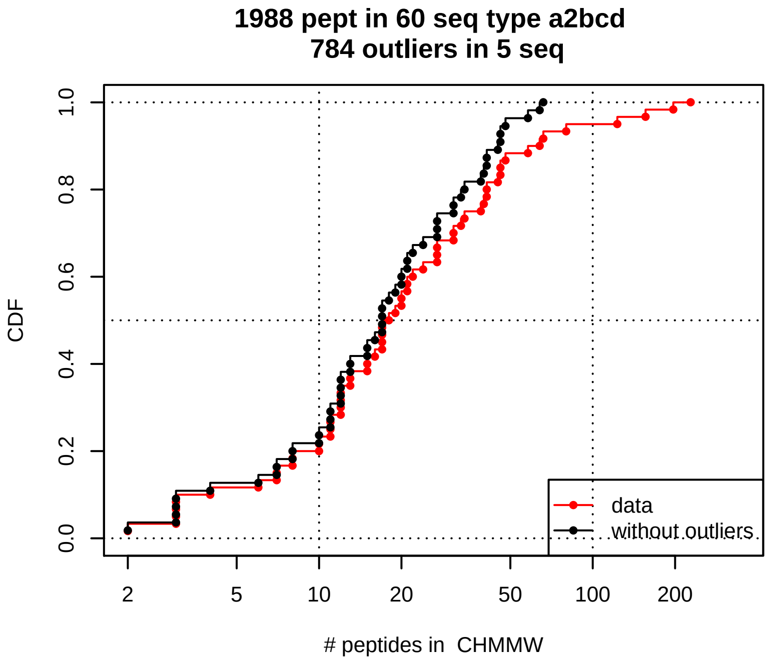
1988 pept in 60 seq type a2bcd
784 outliers in 5 seq
CDF
# peptides in CHMMW
data
without outliers
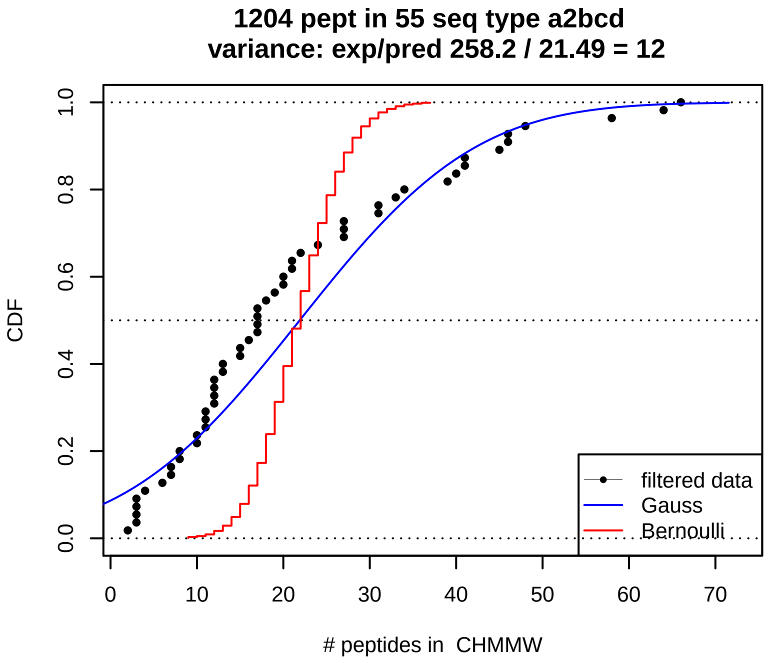
1204 pept in 55 seq type a2bcd
variance: exp/pred 258.2 / 21.49 = 12
CDF
# peptides in CHMMW
filtered data
Gauss
Bernoulli
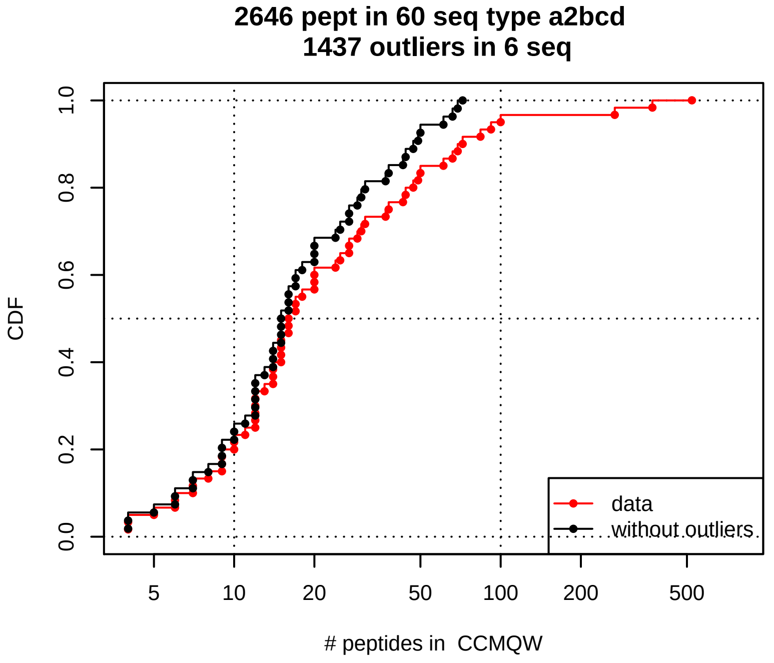
2646 pept in 60 seq type a2bcd
1437 outliers in 6 seq
CDF
# peptides in CCMQW
data
without outliers
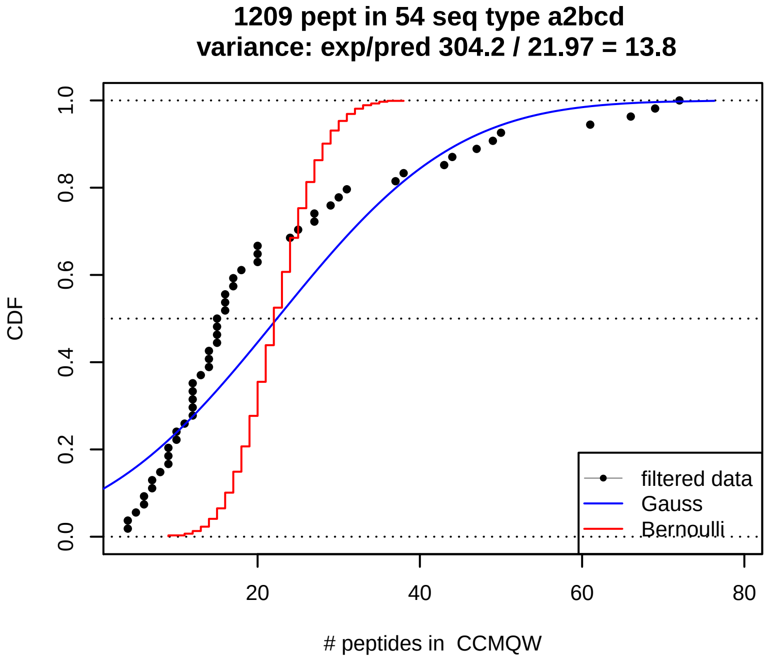
1209 pept in 54 seq type a2bcd
variance: exp/pred 304.2 / 21.97 = 13.8
CDF
# peptides in CCMQW
filtered data
Gauss
Bernoulli
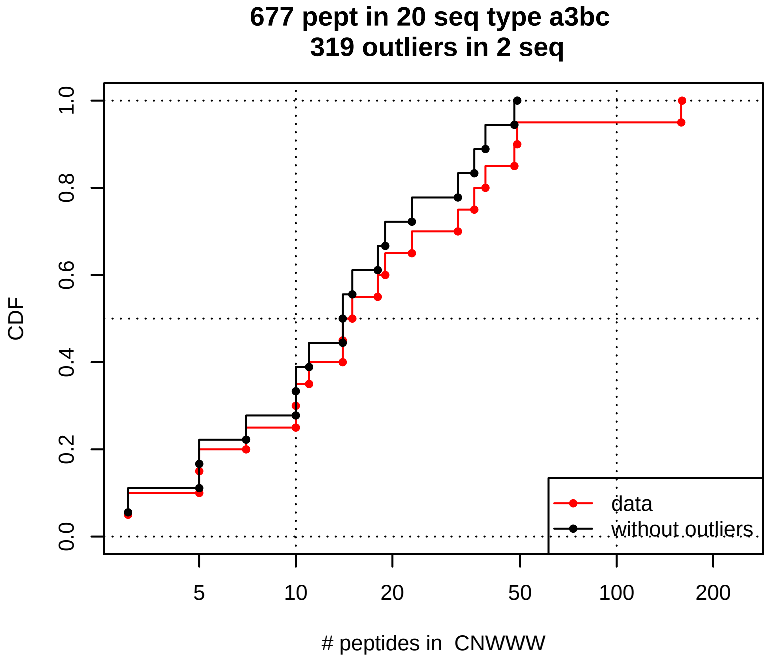
677 pept in 20 seq type a3bc
319 outliers in 2 seq
CDF
# peptides in CNWWW
data
without outliers
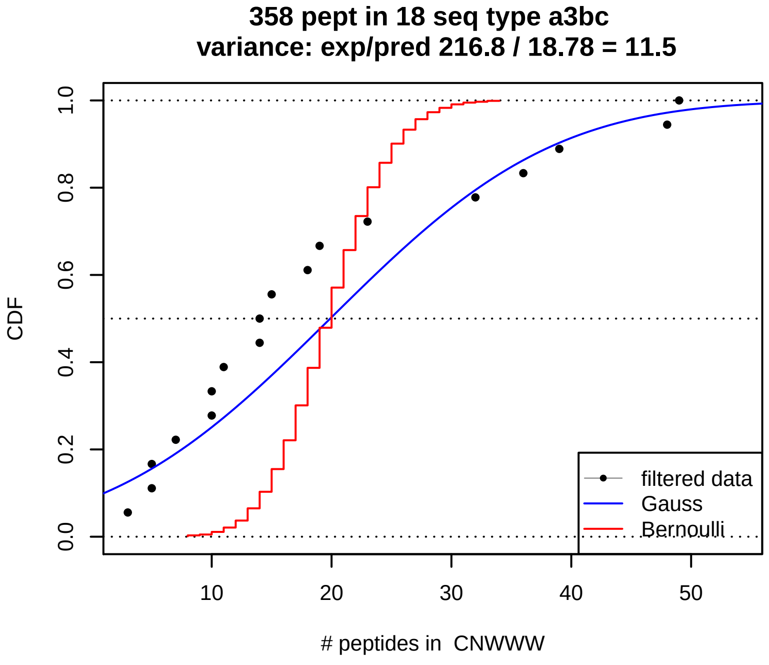
358 pept in 18 seq type a3bc
variance: exp/pred 216.8 / 18.78 = 11.5
CDF
# peptides in CNWWW
filtered data
Gauss
Bernoulli

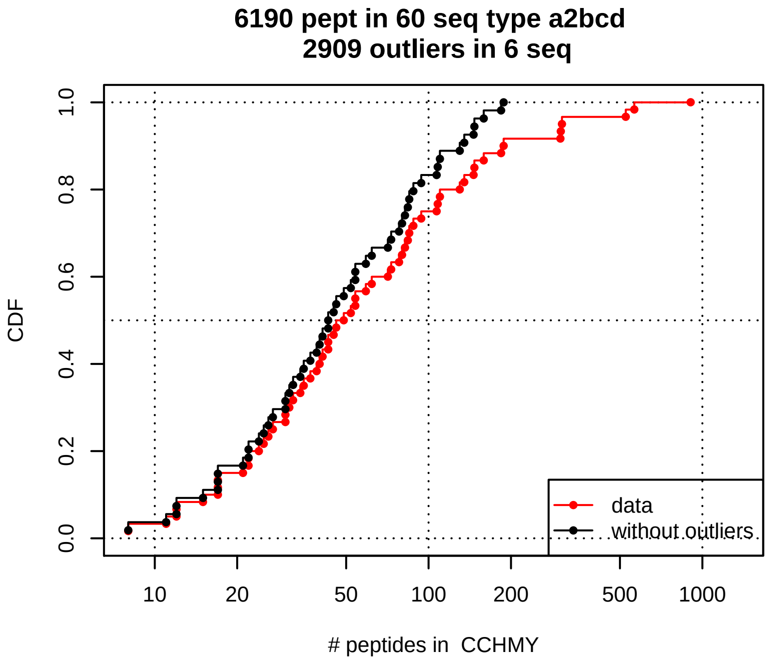
6190 pept in 60 seq type a2bcd
2909 outliers in 6 seq
CDF
# peptides in CCHMY
data
without outliers

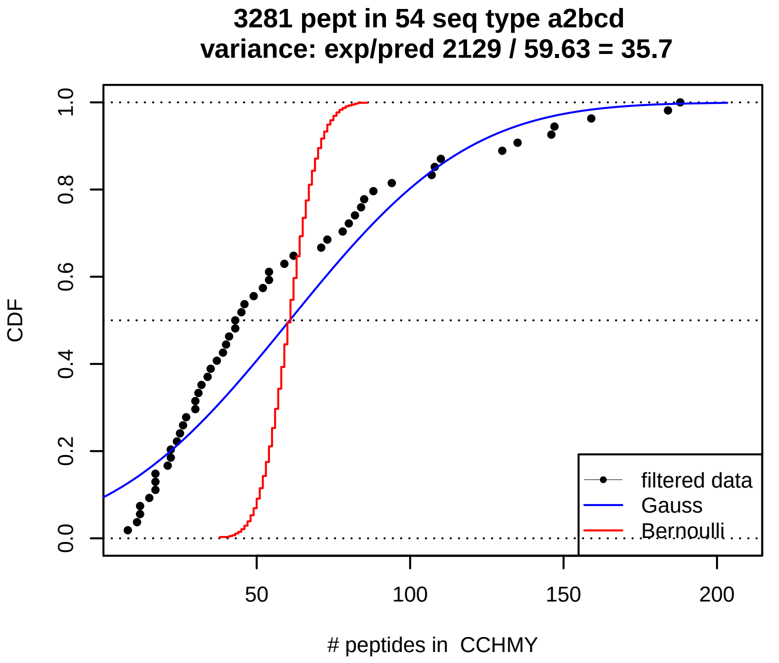
3281 pept in 54 seq type a2bcd
variance: exp/pred 2129 / 59.63 = 35.7
CDF
# peptides in CCHMY
filtered data
Gauss
Bernoulli

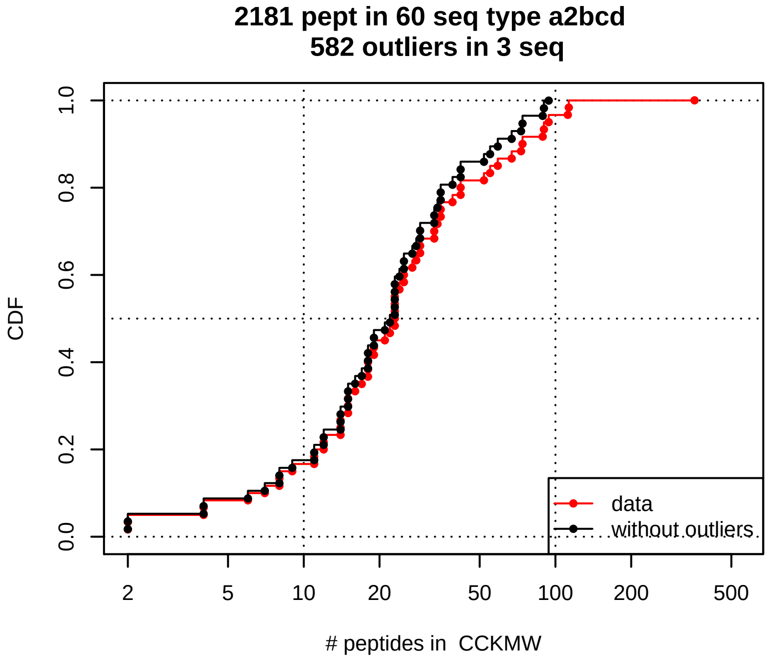
2181 pept in 60 seq type a2bcd
582 outliers in 3 seq
CDF
# peptides in CCKMW
data
without outliers

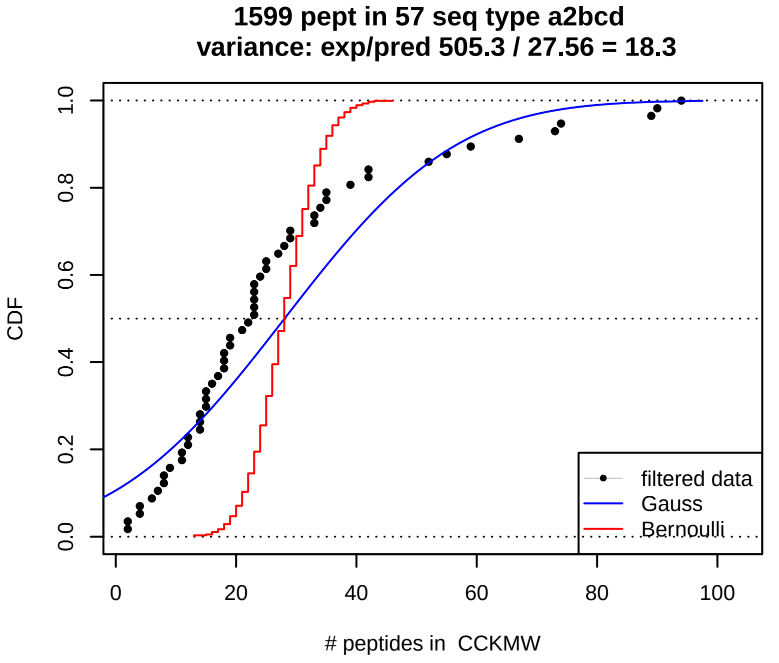
1599 pept in 57 seq type a2bcd
variance: exp/pred 505.3 / 27.56 = 18.3
CDF
# peptides in CCKMW
filtered data
Gauss
Bernoulli

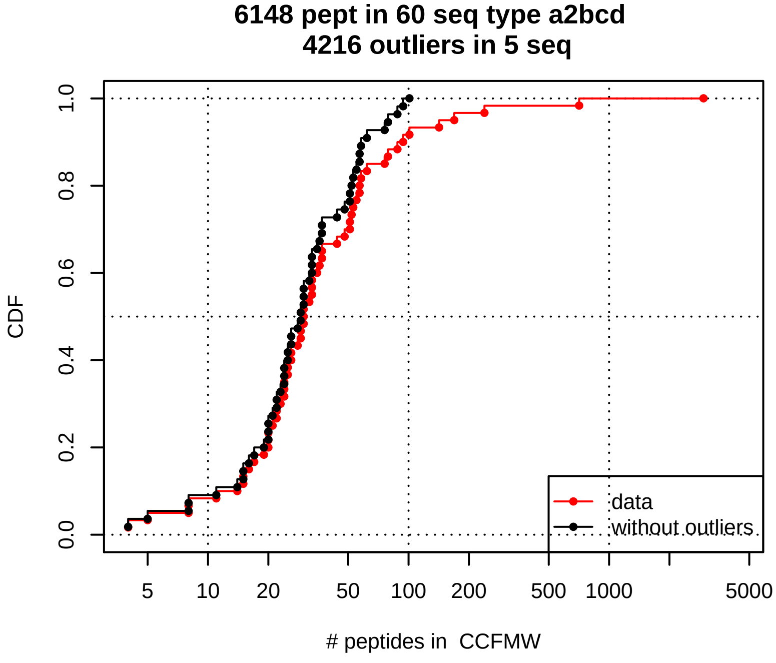
6148 pept in 60 seq type a2bcd
4216 outliers in 5 seq
CDF
# peptides in CCFMW
data
without outliers

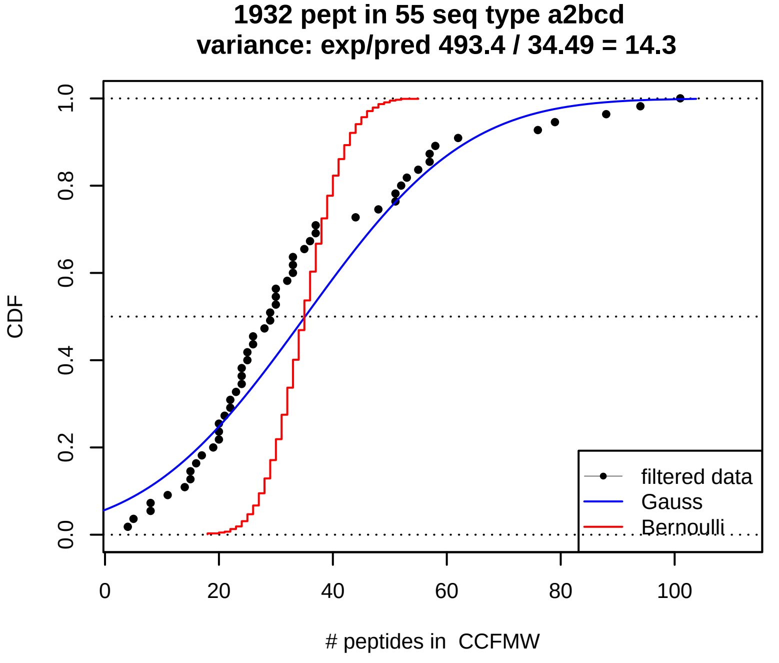
1932 pept in 55 seq type a2bcd
variance: exp/pred 493.4 / 34.49 = 14.3
CDF
# peptides in CCFMW
filtered data
Gauss
Bernoulli

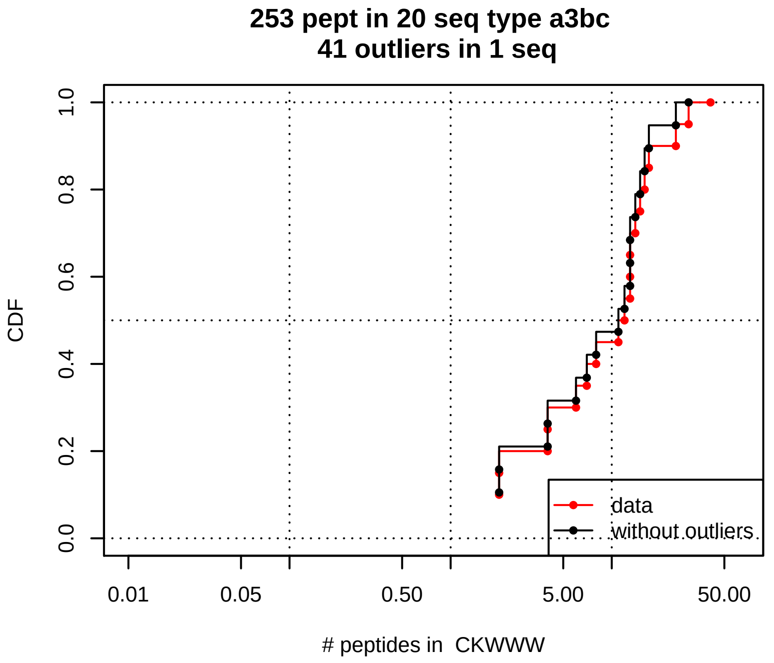
253 pept in 20 seq type a3bc
41 outliers in 1 seq
CDF
0.0
0.2
0.4
0.6
0.8
1.0
0.01
0.05
0.50
5.00
50.00
# peptides in CKWWW
data
without outliers

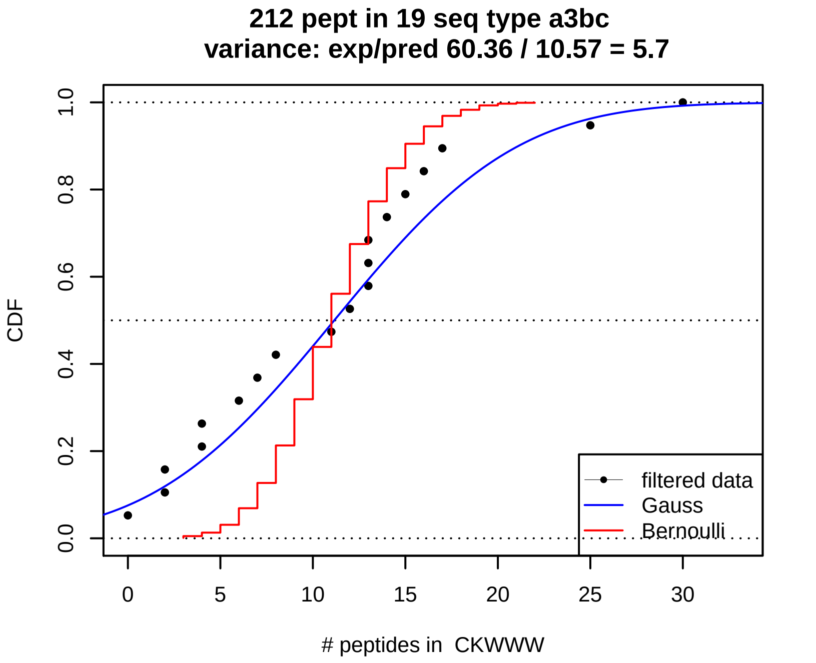
212 pept in 19 seq type a3bc
variance: exp/pred 60.36 / 10.57 = 5.7
CDF
0.0
0.2
0.4
0.6
0.8
1.0
0
5
10
15
20
25
30
# peptides in CKWWW
filtered data
Gauss
Bernoulli

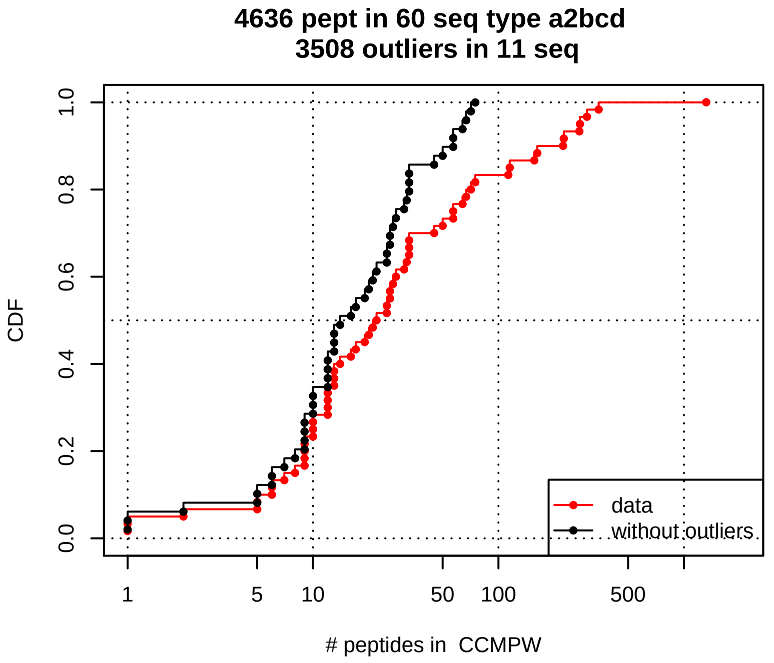
4636 pept in 60 seq type a2bcd
3508 outliers in 11 seq
CDF
0.0
0.2
0.4
0.6
0.8
1.0
1
5
10
50
100
500
# peptides in CCMPW
data
without outliers

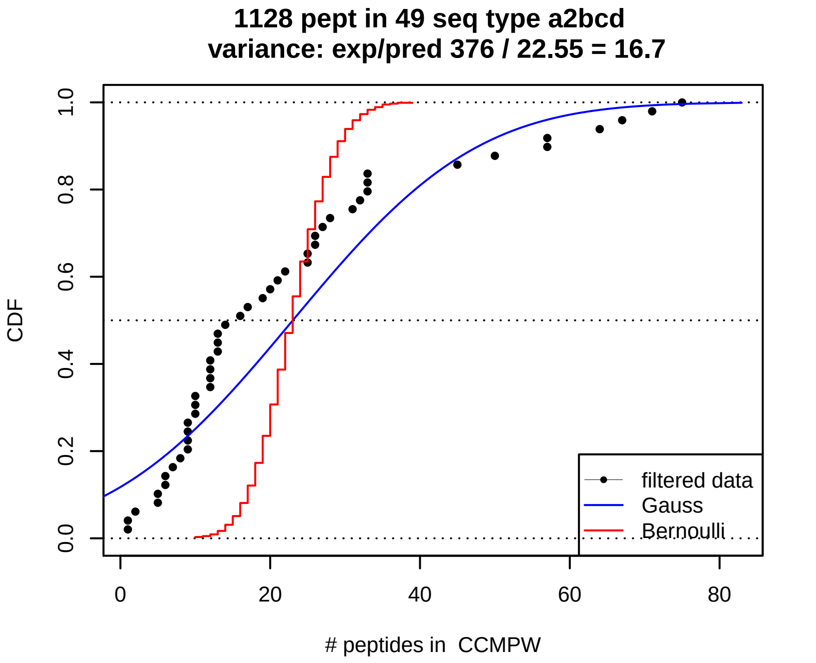
1128 pept in 49 seq type a2bcd
variance: exp/pred 376 / 22.55 = 16.7
CDF
0.0
0.2
0.4
0.6
0.8
1.0
0
20
40
60
80
# peptides in CCMPW
filtered data
Gauss
Bernoulli

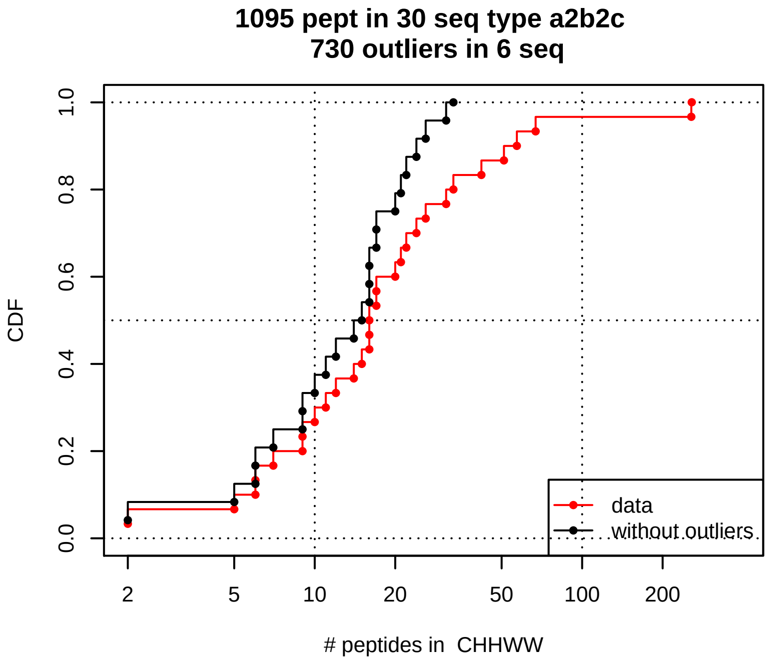
1095 pept in 30 seq type a2b2c
730 outliers in 6 seq
CDF
0.0
0.2
0.4
0.6
0.8
1.0
2
5
10
20
50
100
200
# peptides in CHHWW
data
without outliers

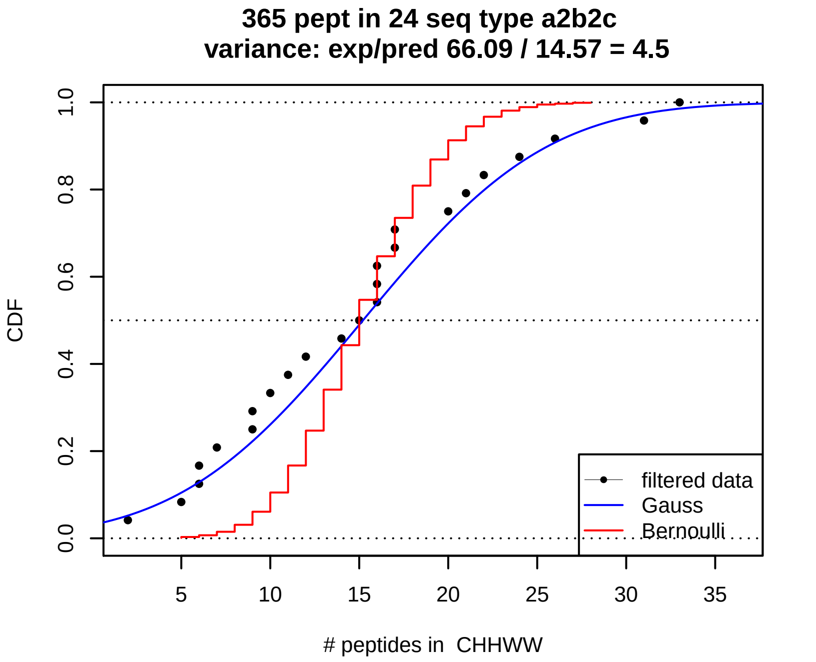
365 pept in 24 seq type a2b2c
variance: exp/pred 66.09 / 14.57 = 4.5
CDF
0.0
0.2
0.4
0.6
0.8
1.0
5
10
15
20
25
30
35
# peptides in CHHWW
filtered data
Gauss
Bernoulli

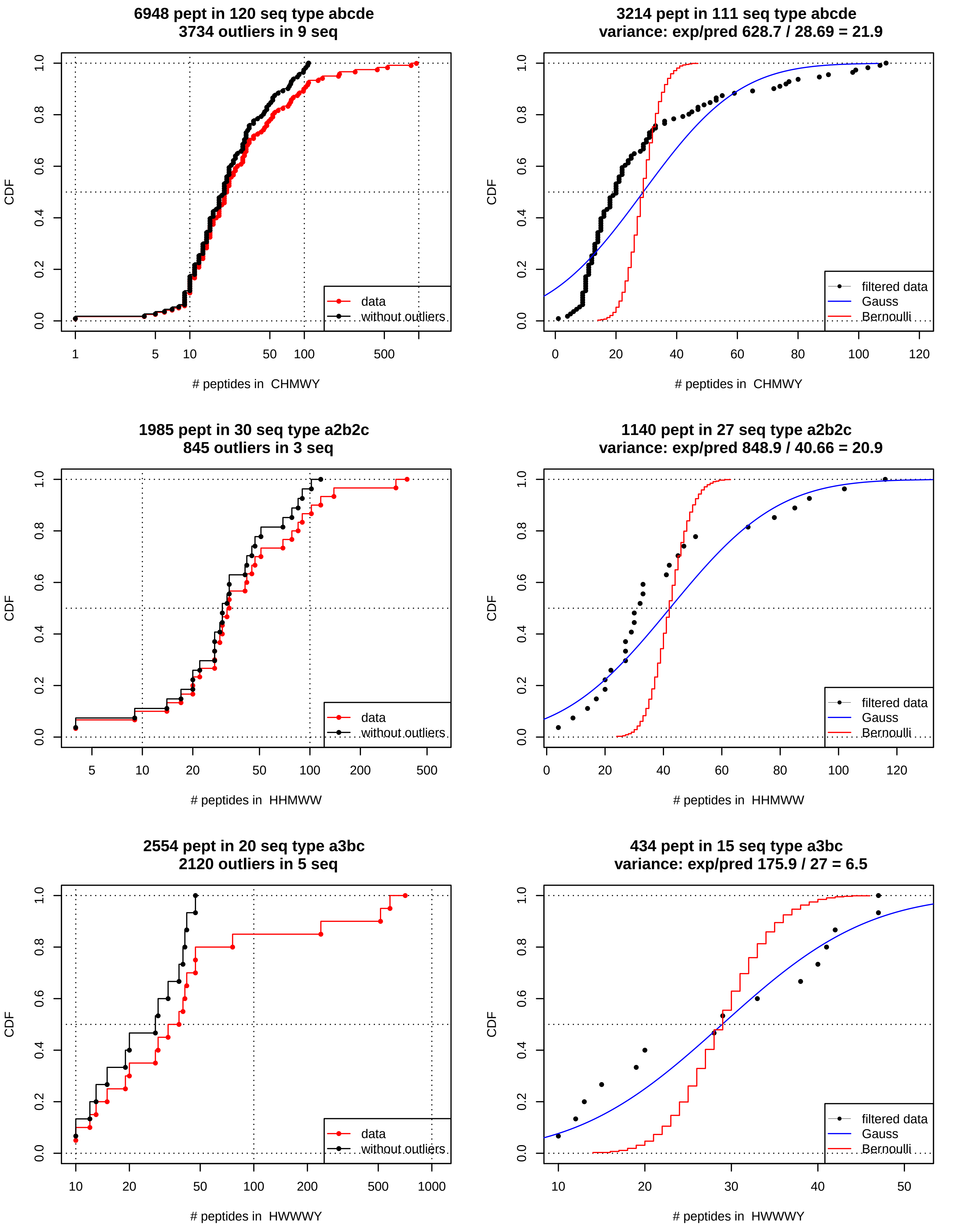
6948 pept in 120 seq type abcde
3734 outliers in 9 seq
CDF
# peptides in CHMWY
data
without outliers
3214 pept in 111 seq type abcde
variance: exp/pred 628.7 / 28.69 = 21.9
CDF
# peptides in CHMWY
filtered data
Gauss
Bernoulli
1985 pept in 30 seq type a2b2c
845 outliers in 3 seq
CDF
# peptides in HHMWW
data
without outliers
1140 pept in 27 seq type a2b2c
variance: exp/pred 848.9 / 40.66 = 20.9
CDF
# peptides in HHMWW
filtered data
Gauss
Bernoulli
2554 pept in 20 seq type a3bc
2120 outliers in 5 seq
CDF
# peptides in HWWWY
data
without outliers
434 pept in 15 seq type a3bc
variance: exp/pred 175.9 / 27 = 6.5
CDF
# peptides in HWWWY
filtered data
Gauss
Bernoulli

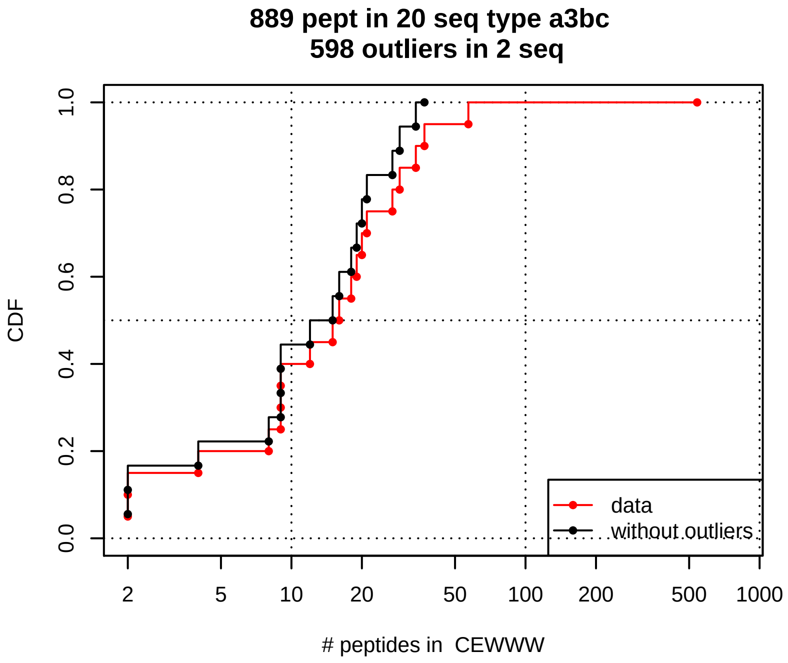
889 pept in 20 seq type a3bc
598 outliers in 2 seq
CDF
# peptides in CEWWW
data
without outliers

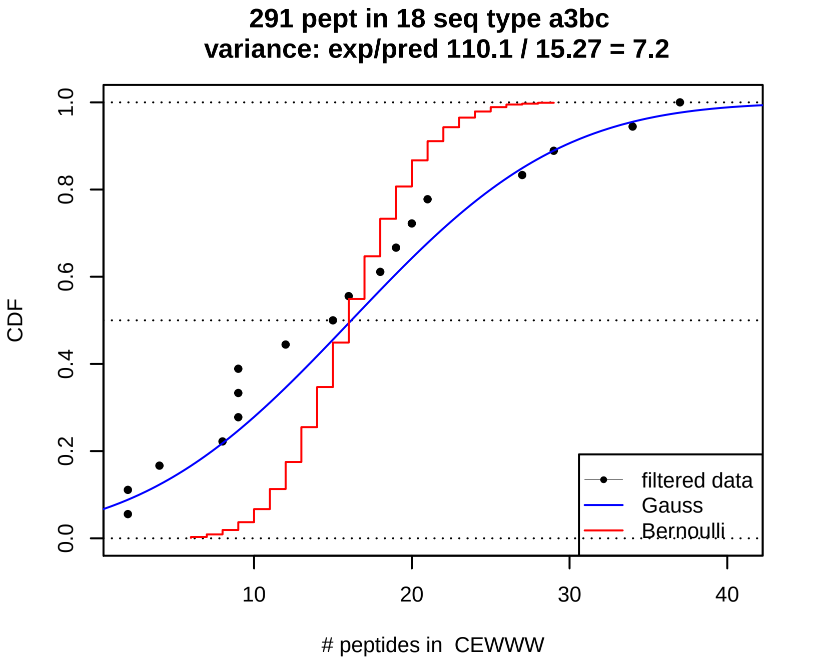
291 pept in 18 seq type a3bc
variance: exp/pred 110.1 / 15.27 = 7.2
CDF
# peptides in CEWWW
filtered data
Gauss
Bernoulli

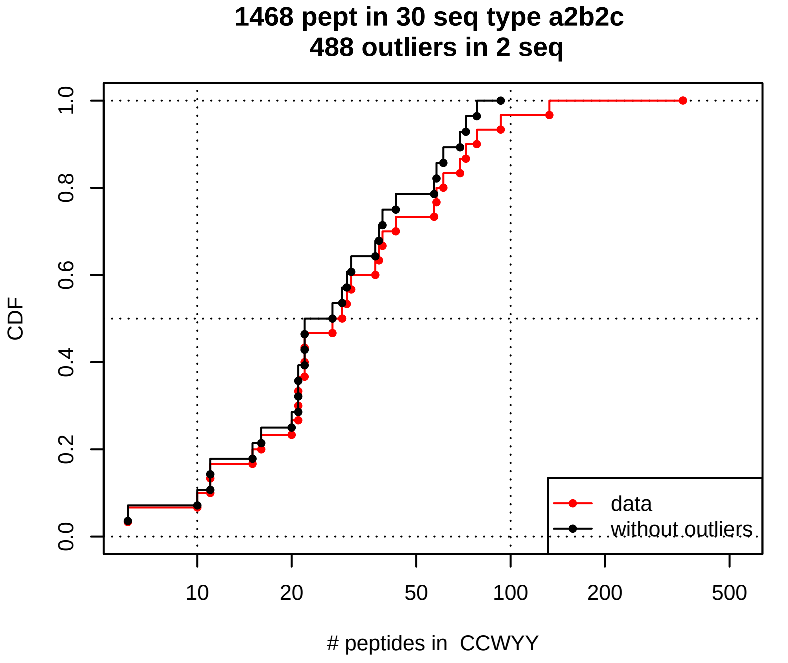
1468 pept in 30 seq type a2b2c
488 outliers in 2 seq
CDF
# peptides in CCWYY
data
without outliers

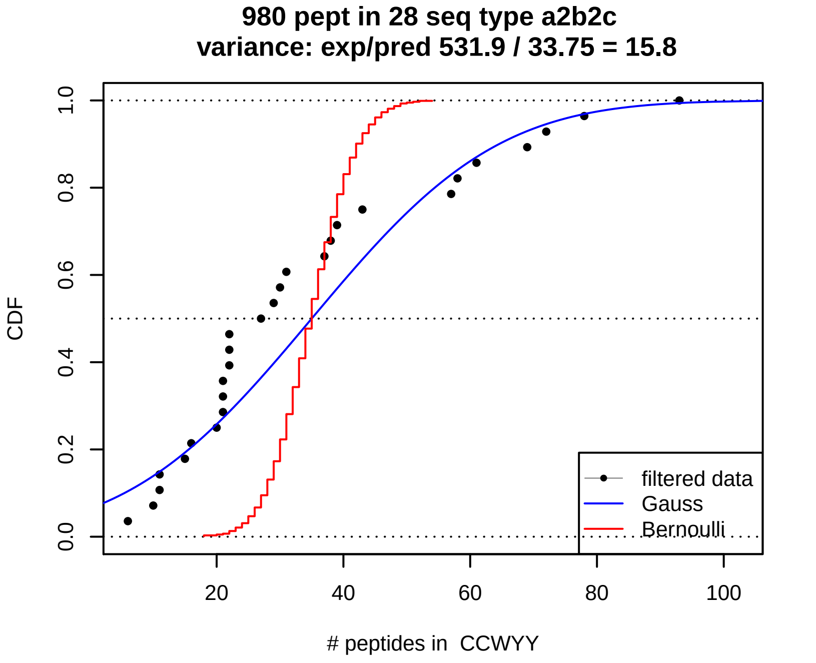
980 pept in 28 seq type a2b2c
variance: exp/pred 531.9 / 33.75 = 15.8
CDF
# peptides in CCWYY
filtered data
Gauss
Bernoulli

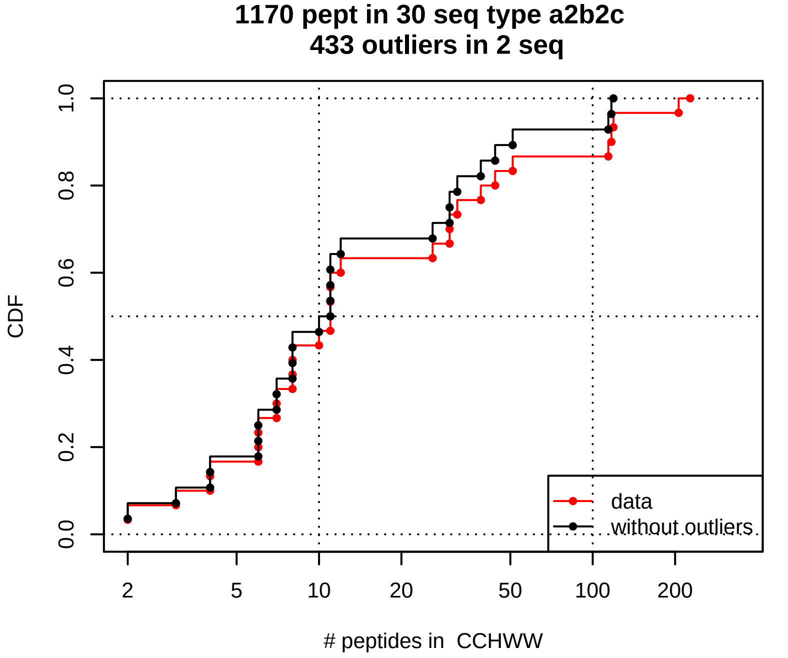
1170 pept in 30 seq type a2b2c
433 outliers in 2 seq
CDF
# peptides in CCHWW
data
without outliers

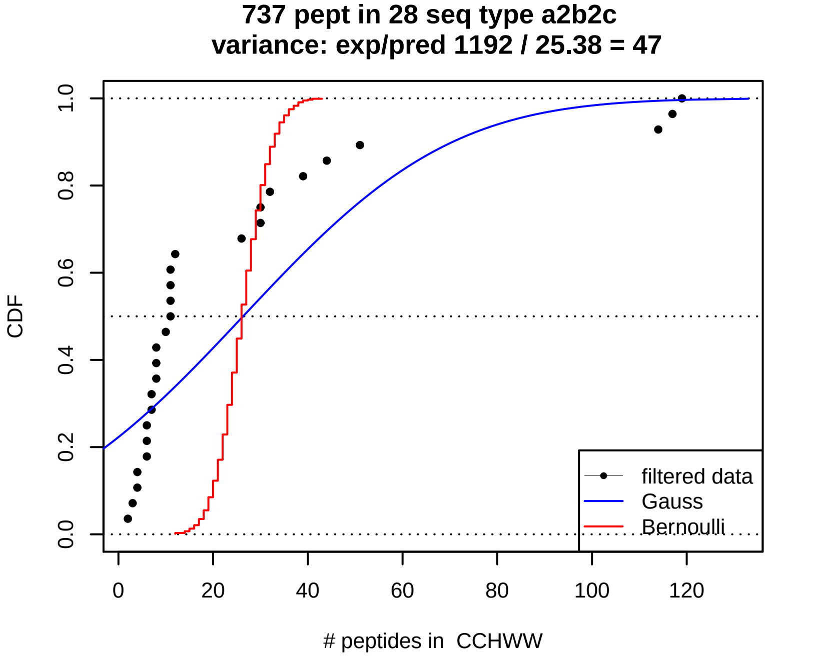
737 pept in 28 seq type a2b2c
variance: exp/pred 1192 / 25.38 = 47
CDF
# peptides in CCHWW
filtered data
Gauss
Bernoulli

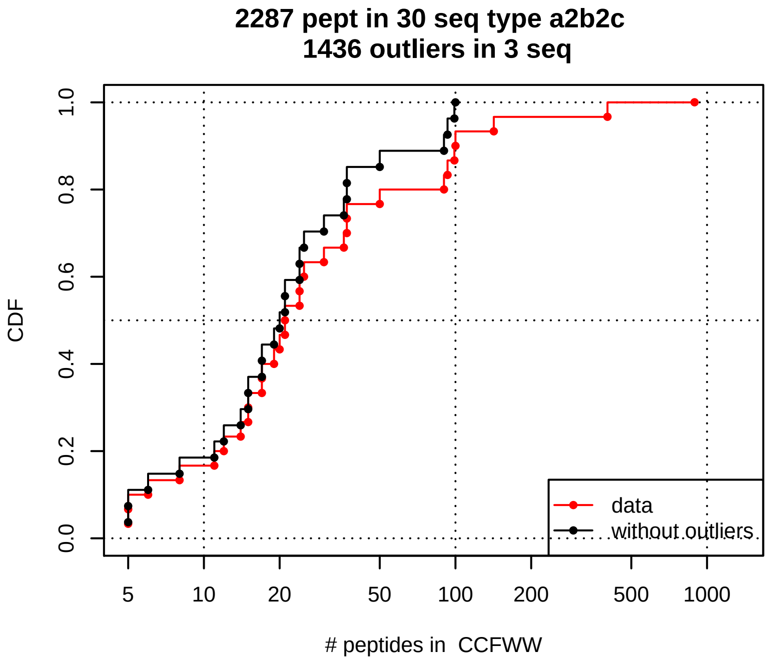
2287 pept in 30 seq type a2b2c
1436 outliers in 3 seq
CDF
# peptides in CCFWW
data
without outliers

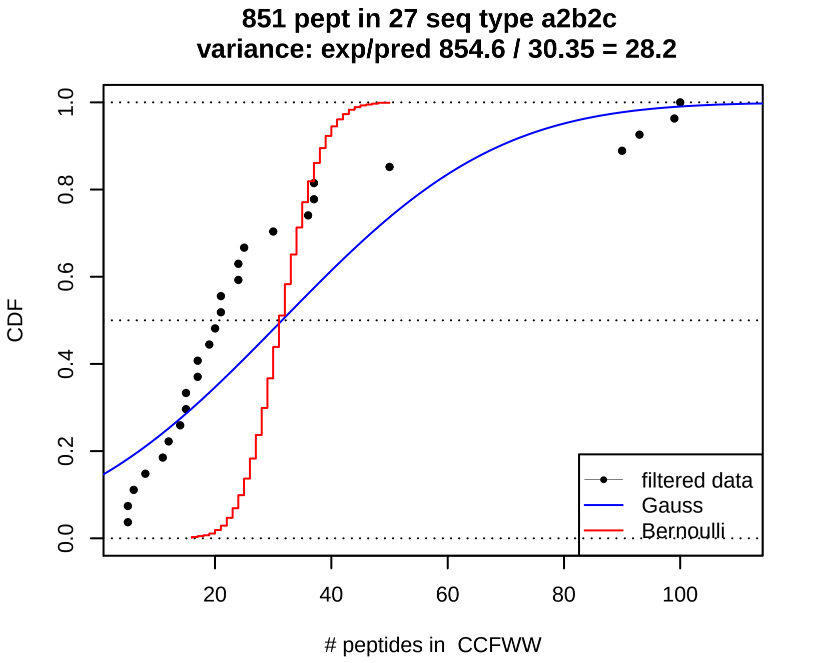
851 pept in 27 seq type a2b2c
variance: exp/pred 854.6 / 30.35 = 28.2
CDF
# peptides in CCFWW
filtered data
Gauss
Bernoulli

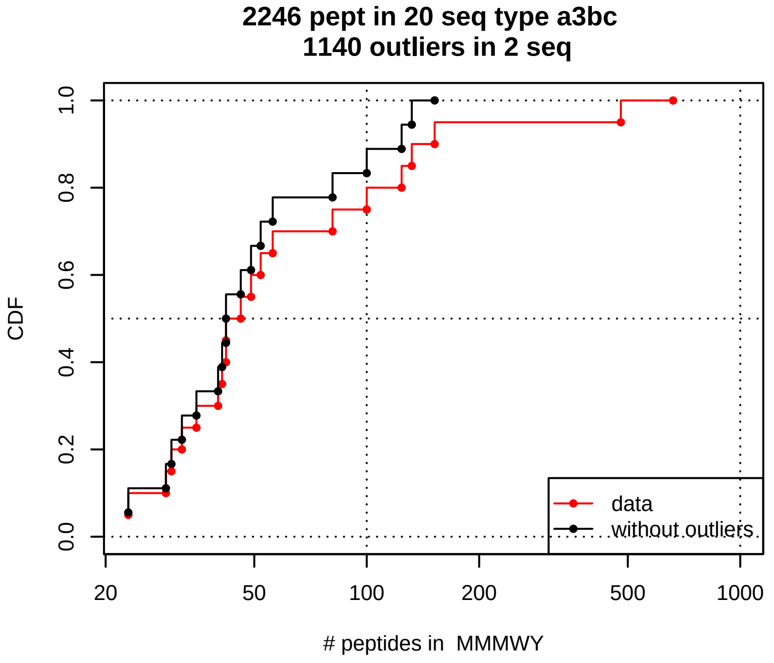
2246 pept in 20 seq type a3bc
1140 outliers in 2 seq
CDF
# peptides in MMMWY
data
without outliers

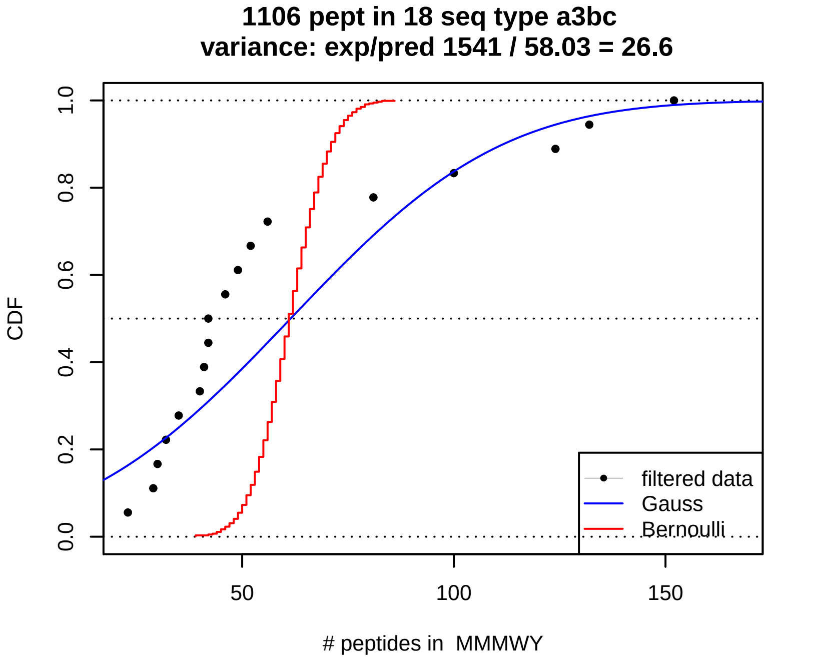
1106 pept in 18 seq type a3bc
variance: exp/pred 1541 / 58.03 = 26.6
CDF
# peptides in MMMWY
filtered data
Gauss
Bernoulli

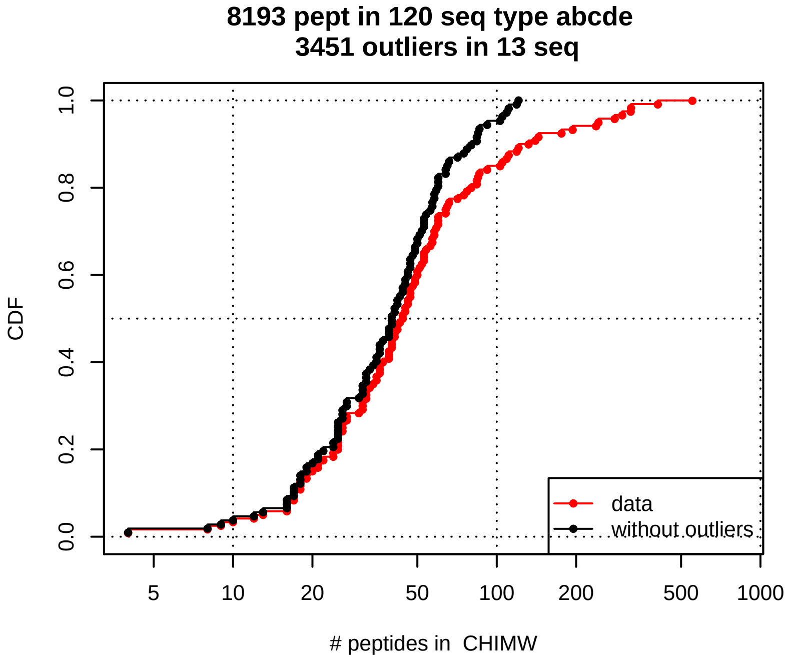
8193 pept in 120 seq type abcde
3451 outliers in 13 seq
CDF
# peptides in CHIMW
data
without outliers

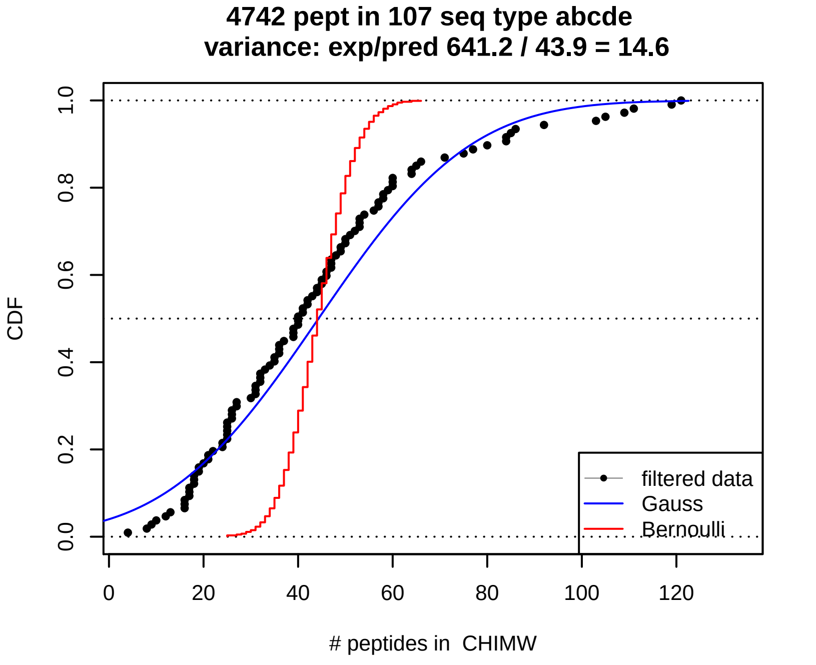
4742 pept in 107 seq type abcde
variance: exp/pred 641.2 / 43.9 = 14.6
CDF
# peptides in CHIMW
filtered data
Gauss
Bernoulli

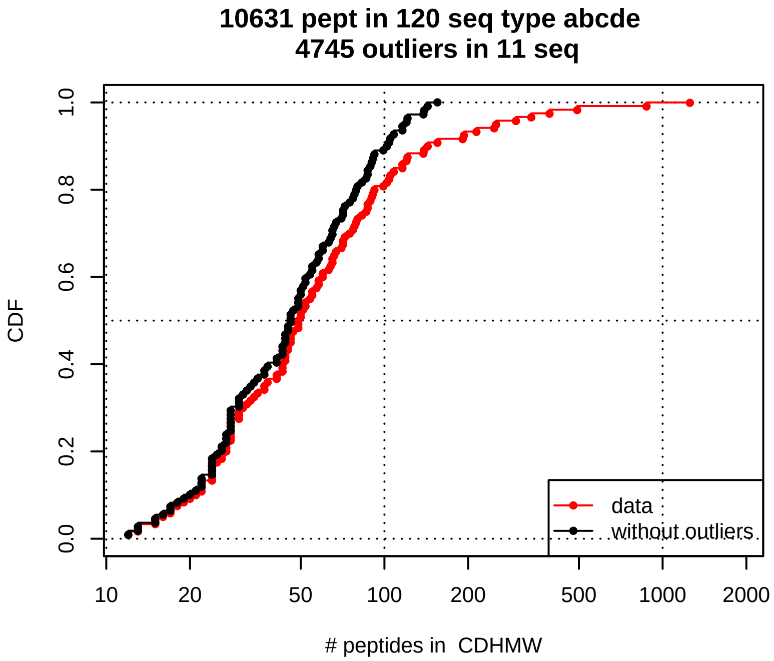
10631 pept in 120 seq type abcde
4745 outliers in 11 seq
CDF
0.0
0.2
0.4
0.6
0.8
1.0
10
20
50
100
200
500
1000
2000
# peptides in CDHMW
data
without outliers

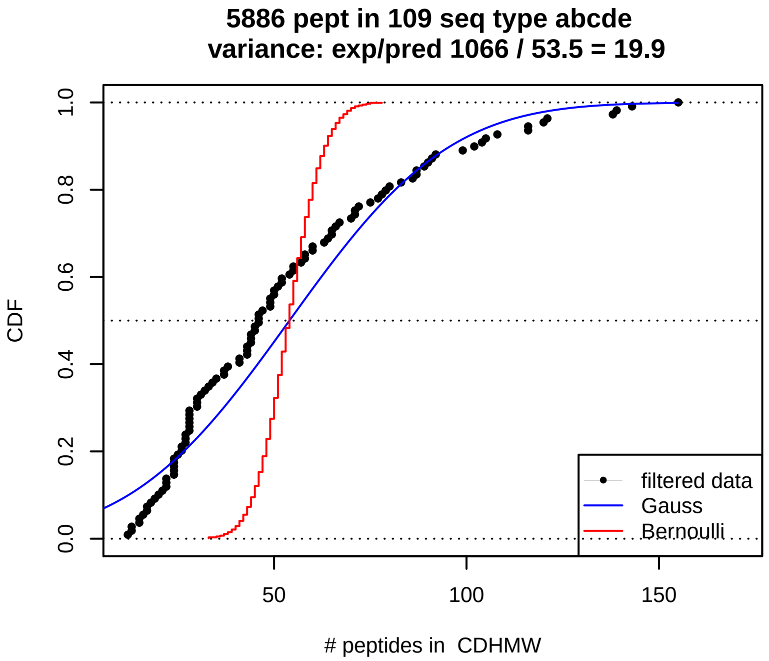
5886 pept in 109 seq type abcde
variance: exp/pred 1066 / 53.5 = 19.9
CDF
0.0
0.2
0.4
0.6
0.8
1.0
50
100
150
# peptides in CDHMW
filtered data
Gauss
Bernoulli

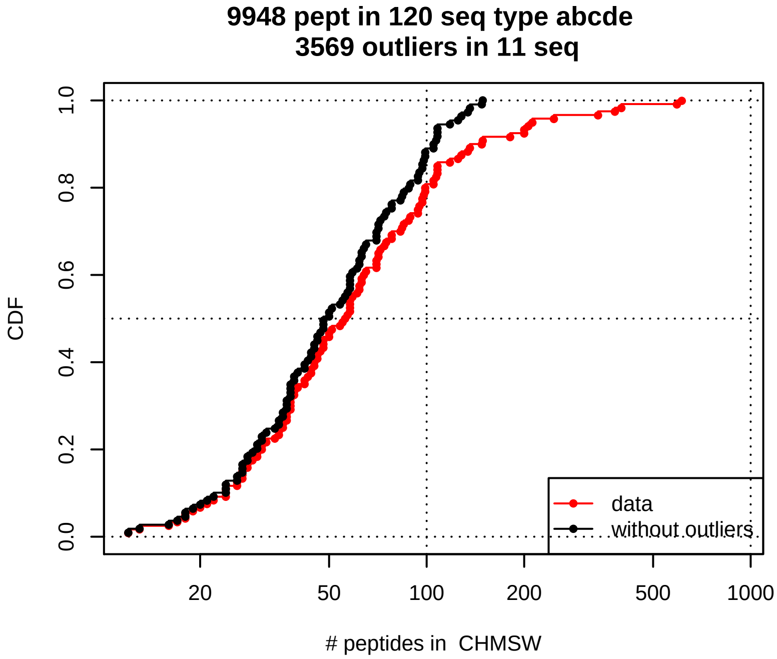
9948 pept in 120 seq type abcde
3569 outliers in 11 seq
CDF
0.0
0.2
0.4
0.6
0.8
1.0
20
50
100
200
500
1000
# peptides in CHMSW
data
without outliers

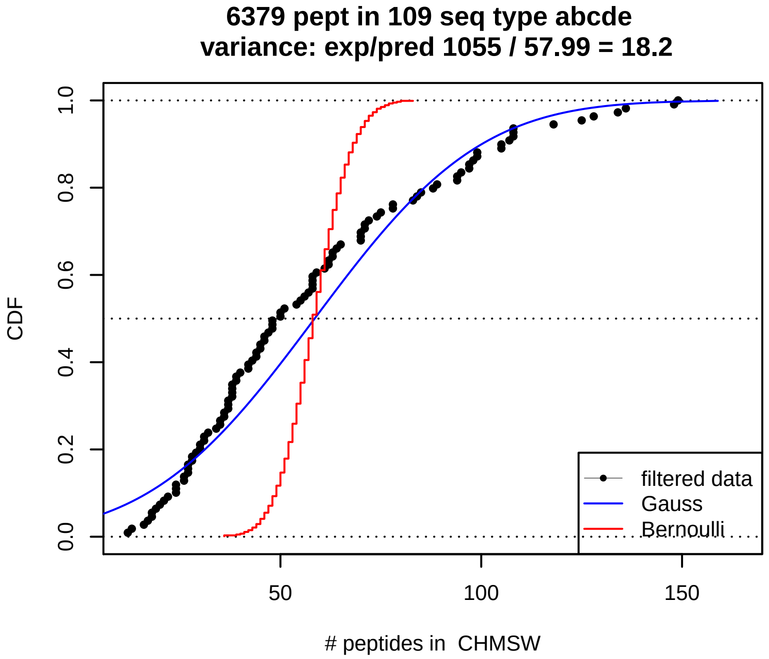
6379 pept in 109 seq type abcde
variance: exp/pred 1055 / 57.99 = 18.2
CDF
0.0
0.2
0.4
0.6
0.8
1.0
50
100
150
# peptides in CHMSW
filtered data
Gauss
Bernoulli

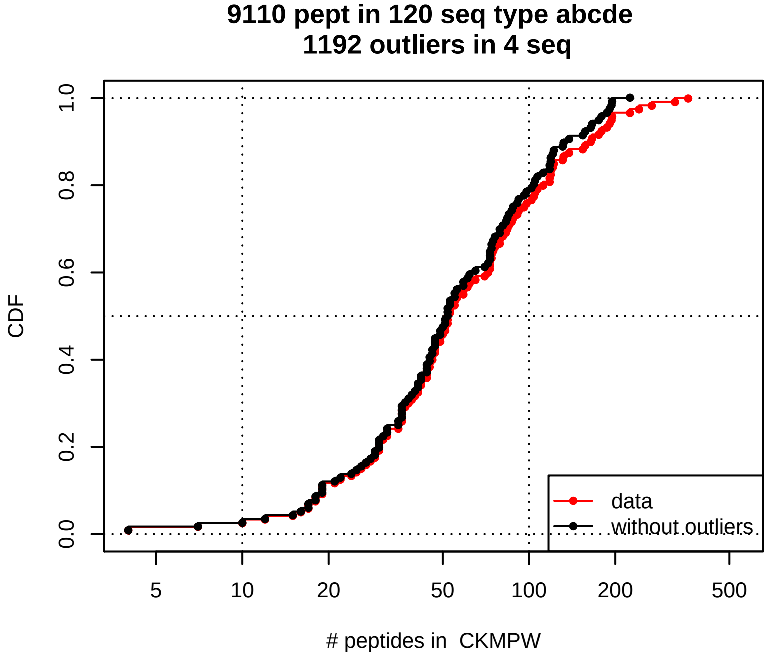
9110 pept in 120 seq type abcde
1192 outliers in 4 seq
CDF
0.0
0.2
0.4
0.6
0.8
1.0
5
10
20
50
100
200
500
# peptides in CKMPW
data
without outliers

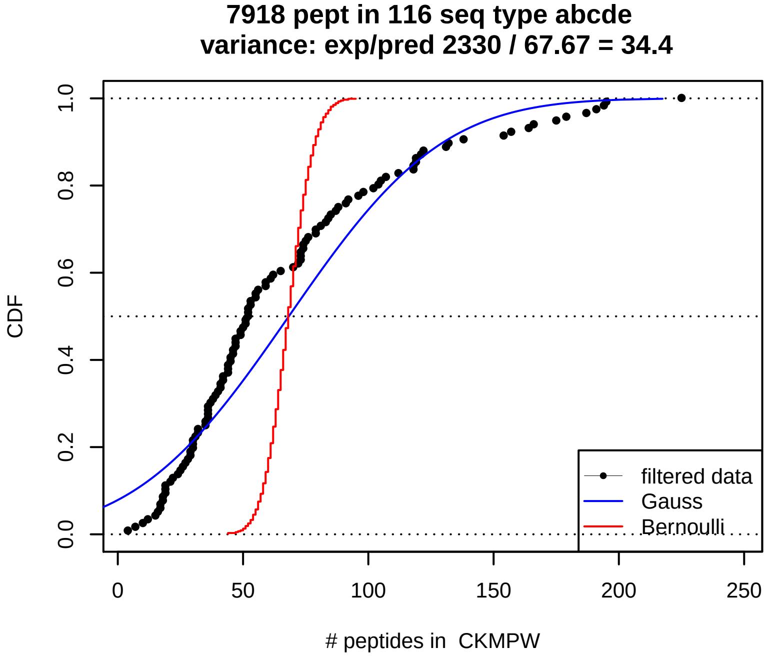
7918 pept in 116 seq type abcde
variance: exp/pred 2330 / 67.67 = 34.4
CDF
0.0
0.2
0.4
0.6
0.8
1.0
0
50
100
150
200
250
# peptides in CKMPW
filtered data
Gauss
Bernoulli

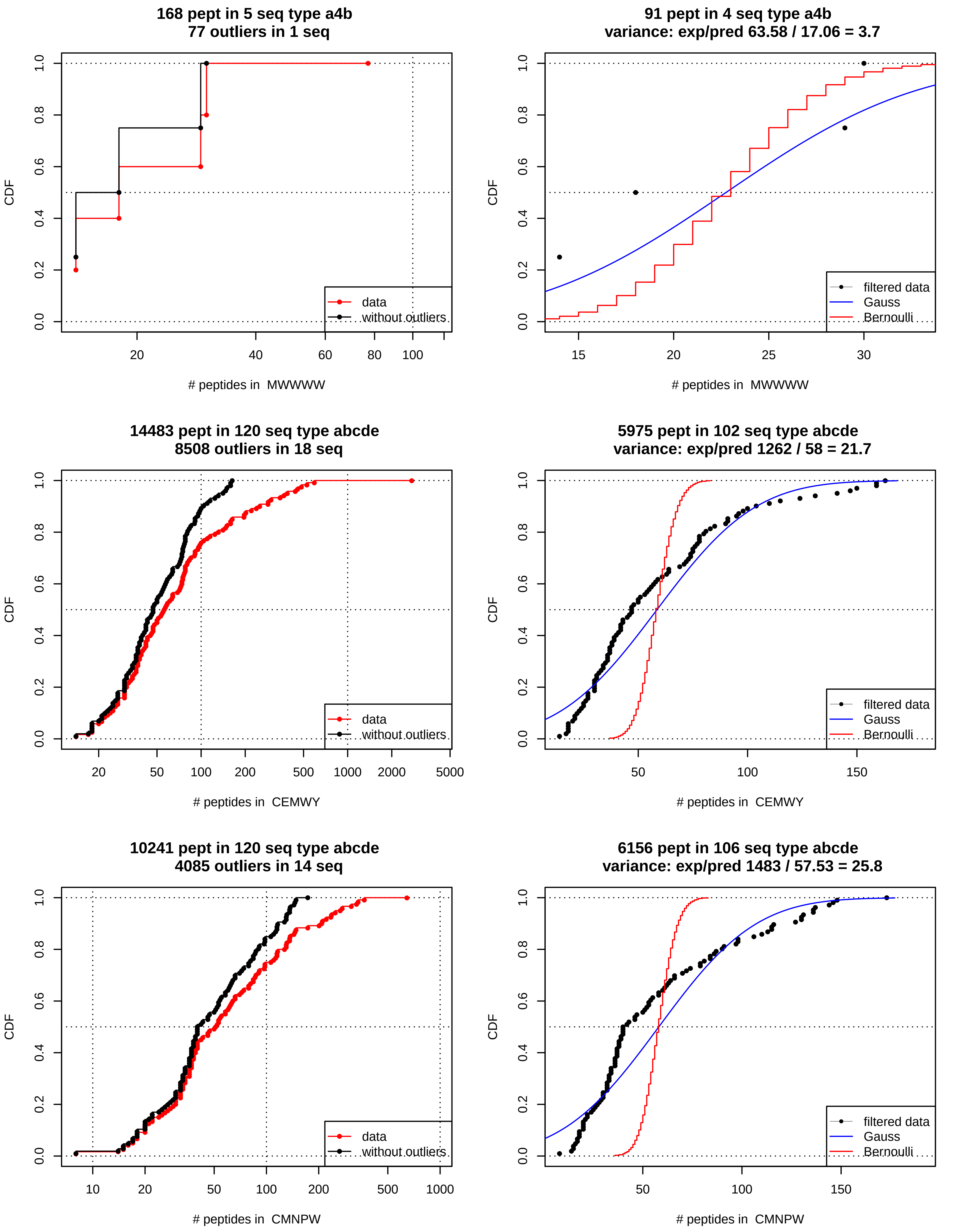
168 pept in 5 seq type a4b
77 outliers in 1 seq
CDF
# peptides in MWWWW
data
without outliers
91 pept in 4 seq type a4b
variance: exp/pred 63.58 / 17.06 = 3.7
CDF
# peptides in MWWWW
filtered data
Gauss
Bernoulli
14483 pept in 120 seq type abcde
8508 outliers in 18 seq
CDF
# peptides in CEMWY
data
without outliers
5975 pept in 102 seq type abcde
variance: exp/pred 1262 / 58 = 21.7
CDF
# peptides in CEMWY
filtered data
Gauss
Bernoulli
10241 pept in 120 seq type abcde
4085 outliers in 14 seq
CDF
# peptides in CMNPW
data
without outliers
6156 pept in 106 seq type abcde
variance: exp/pred 1483 / 57.53 = 25.8
CDF
# peptides in CMNPW
filtered data
Gauss
Bernoulli

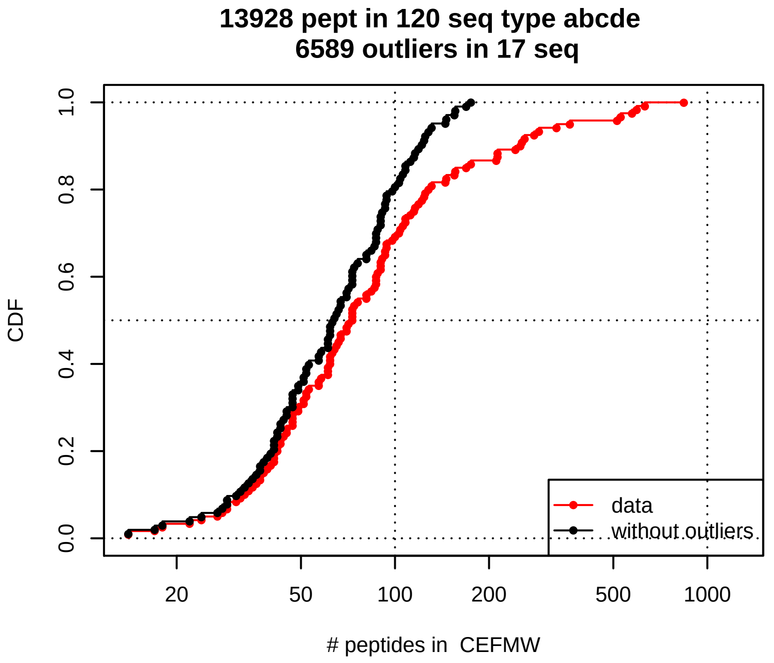
13928 pept in 120 seq type abcde
6589 outliers in 17 seq
CDF
# peptides in CEFMW
data
without outliers

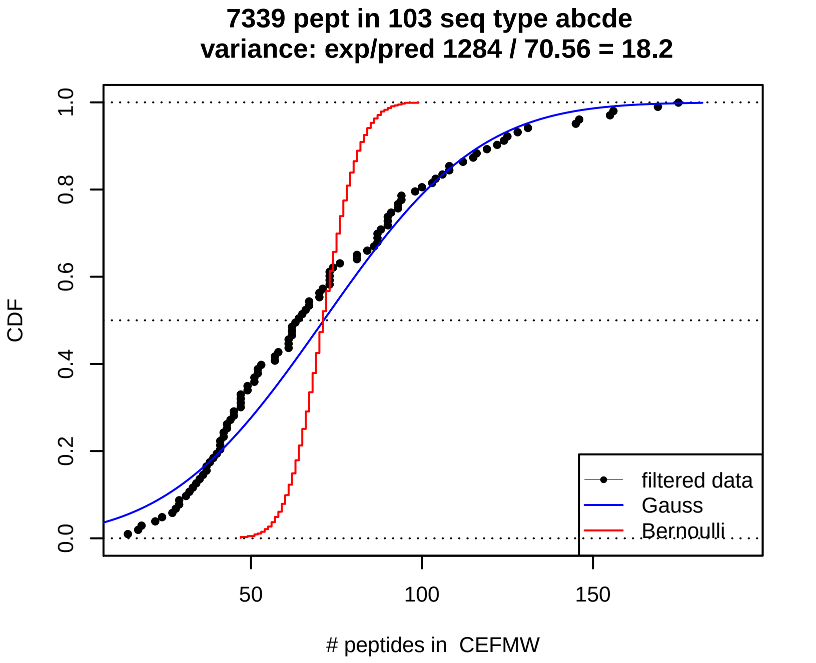
7339 pept in 103 seq type abcde
variance: exp/pred 1284 / 70.56 = 18.2
CDF
# peptides in CEFMW
filtered data
Gauss
Bernoulli

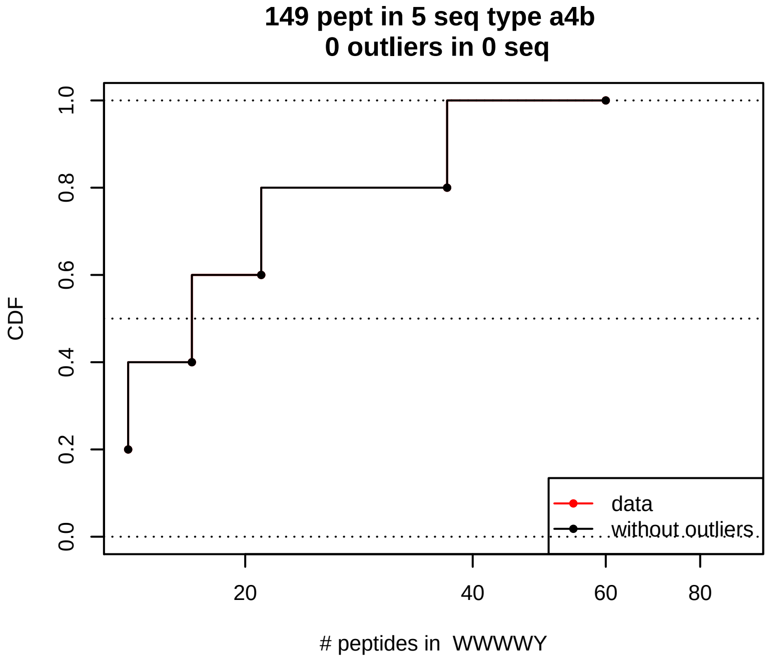
149 pept in 5 seq type a4b
0 outliers in 0 seq
CDF
# peptides in WWWWY
data
without outliers

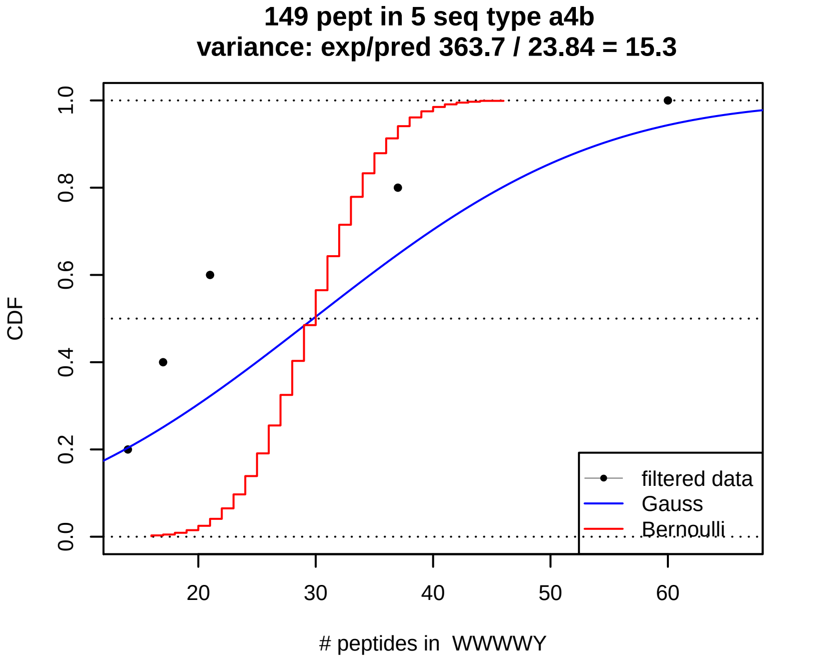
149 pept in 5 seq type a4b
variance: exp/pred 363.7 / 23.84 = 15.3
CDF
# peptides in WWWWY
filtered data
Gauss
Bernoulli

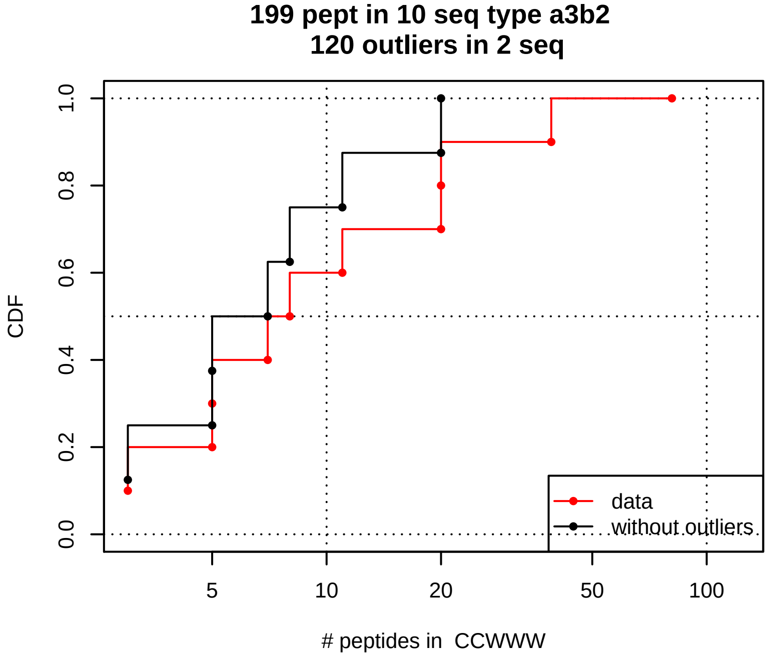
199 pept in 10 seq type a3b2
120 outliers in 2 seq
CDF
# peptides in CCWWW
data
without outliers

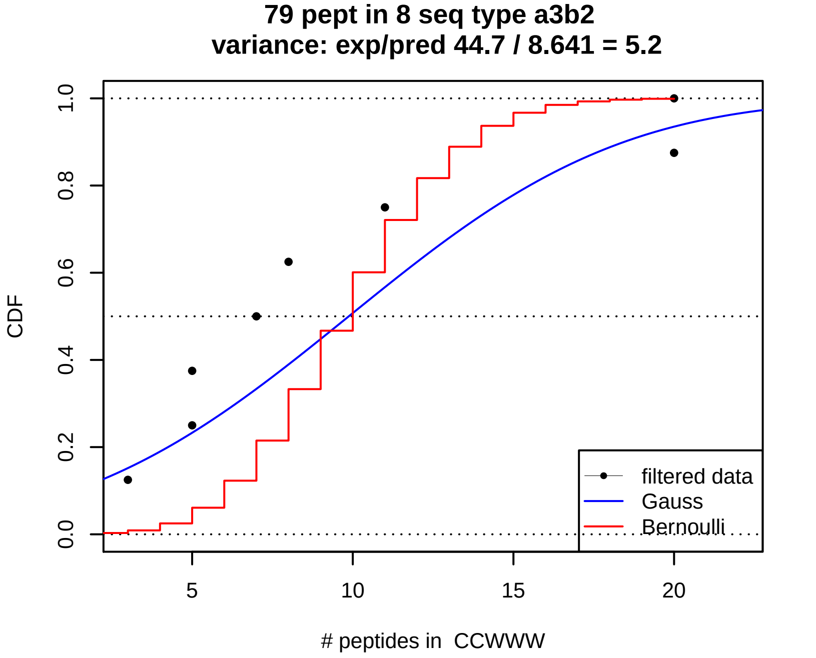
79 pept in 8 seq type a3b2
variance: exp/pred 44.7 / 8.641 = 5.2
CDF
# peptides in CCWWW
filtered data
Gauss
Bernoulli

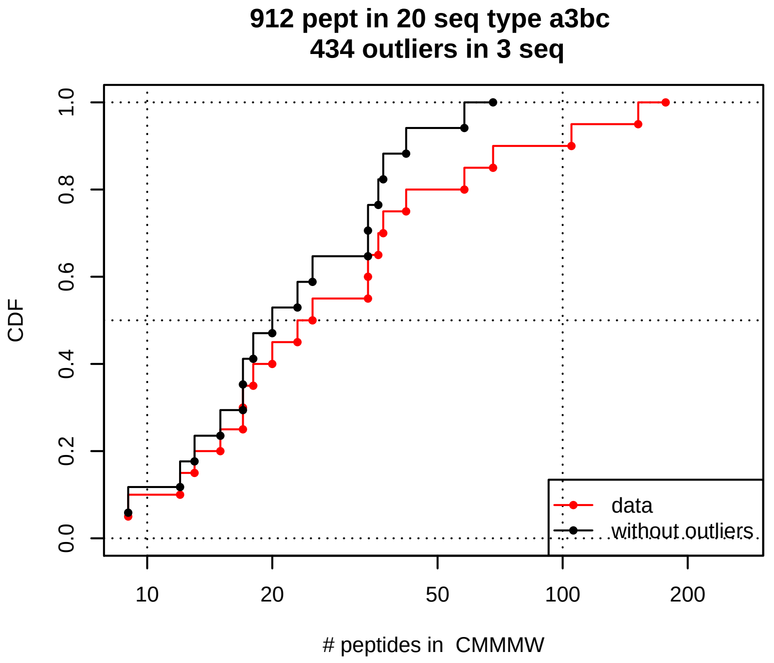
912 pept in 20 seq type a3bc
434 outliers in 3 seq
CDF
# peptides in CMMMW
data
without outliers

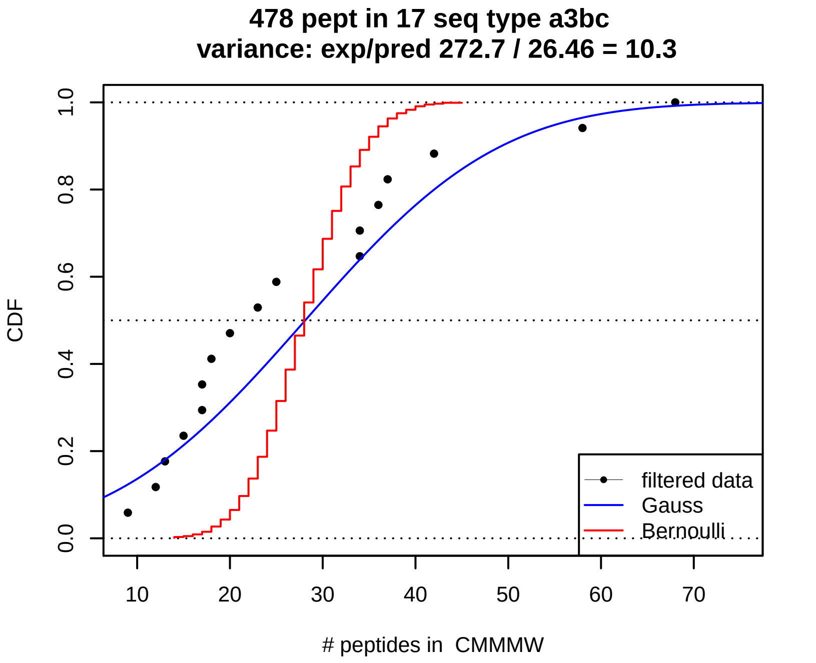
478 pept in 17 seq type a3bc
variance: exp/pred 272.7 / 26.46 = 10.3
CDF
# peptides in CMMMW
filtered data
Gauss
Bernoulli

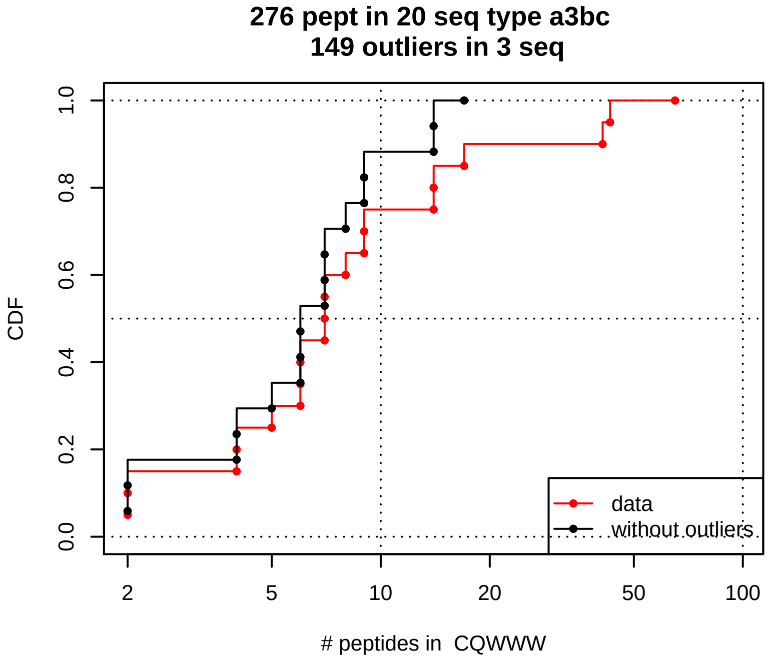
276 pept in 20 seq type a3bc
149 outliers in 3 seq
CDF
# peptides in CQWWW
data
without outliers

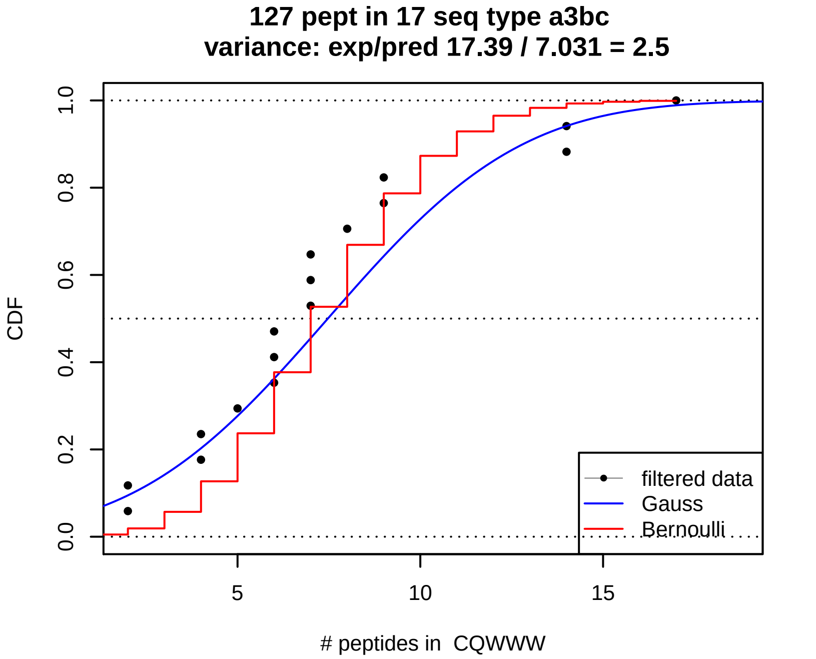
127 pept in 17 seq type a3bc
variance: exp/pred 17.39 / 7.031 = 2.5
CDF
# peptides in CQWWW
filtered data
Gauss
Bernoulli

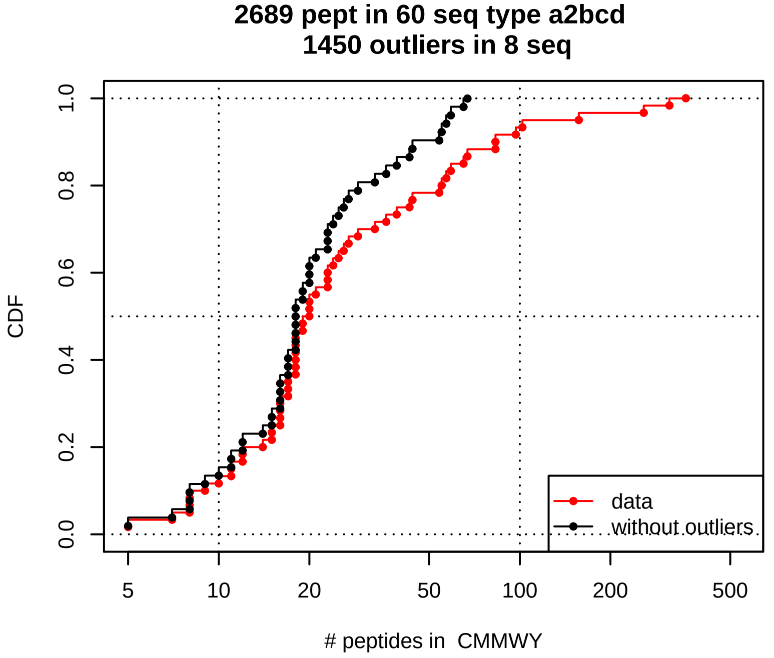
2689 pept in 60 seq type a2bcd
1450 outliers in 8 seq
CDF
# peptides in CMMWY
data
without outliers

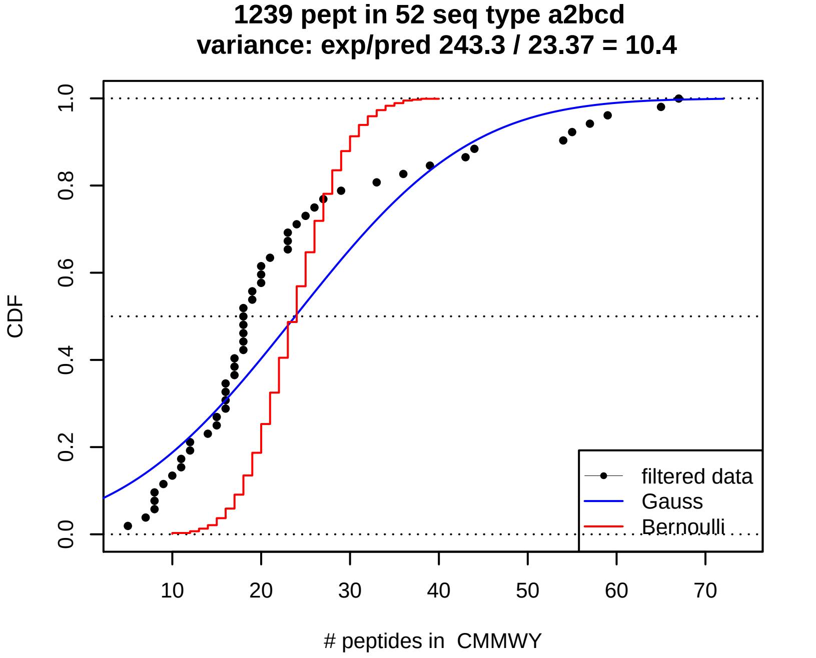
1239 pept in 52 seq type a2bcd
variance: exp/pred 243.3 / 23.37 = 10.4
CDF
# peptides in CMMWY
filtered data
Gauss
Bernoulli

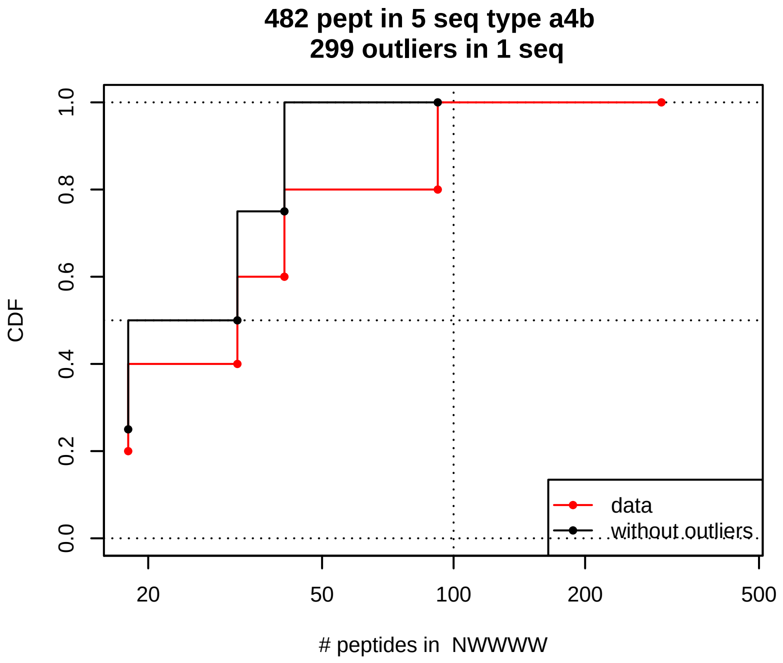
482 pept in 5 seq type a4b
299 outliers in 1 seq
CDF
# peptides in NWWWW
data
without outliers
0.0
0.2
0.4
0.6
0.8
1.0
20
50
100
200
500

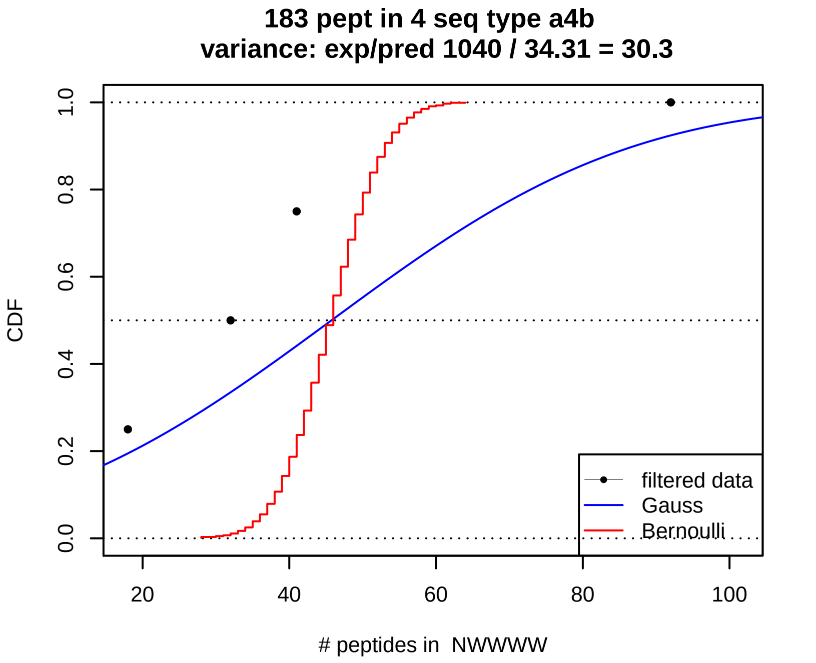
183 pept in 4 seq type a4b
variance: exp/pred 1040 / 34.31 = 30.3
CDF
# peptides in NWWWW
filtered data
Gauss
Bernoulli
0.0
0.2
0.4
0.6
0.8
1.0
20
40
60
80
100

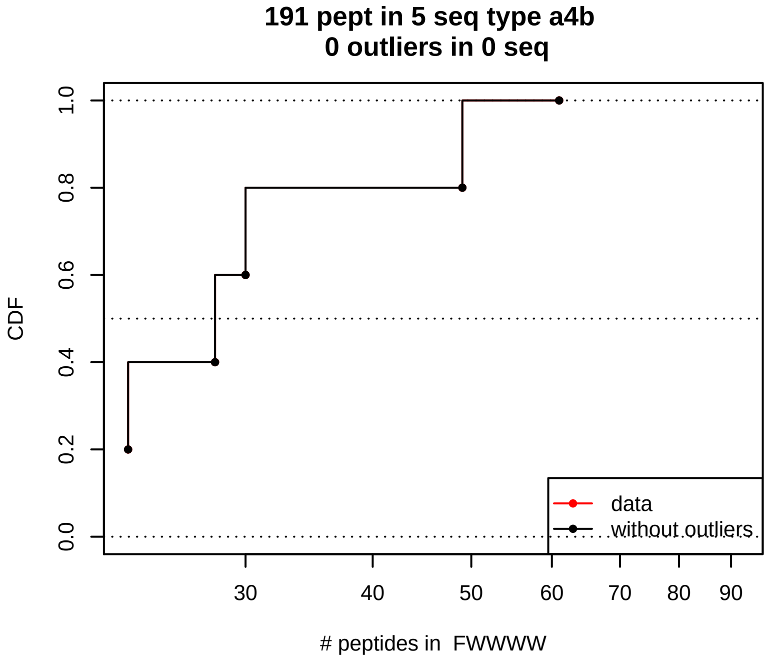
191 pept in 5 seq type a4b
0 outliers in 0 seq
CDF
# peptides in FWWWW
data
without outliers
0.0
0.2
0.4
0.6
0.8
1.0
30
40
50
60
70
80
90

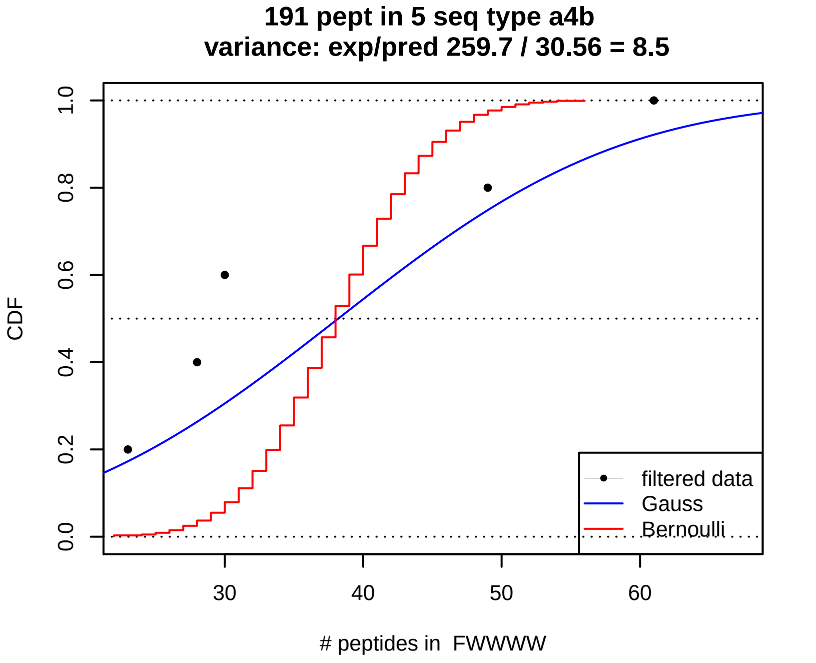
191 pept in 5 seq type a4b
variance: exp/pred 259.7 / 30.56 = 8.5
CDF
# peptides in FWWWW
filtered data
Gauss
Bernoulli
0.0
0.2
0.4
0.6
0.8
1.0
30
40
50
60

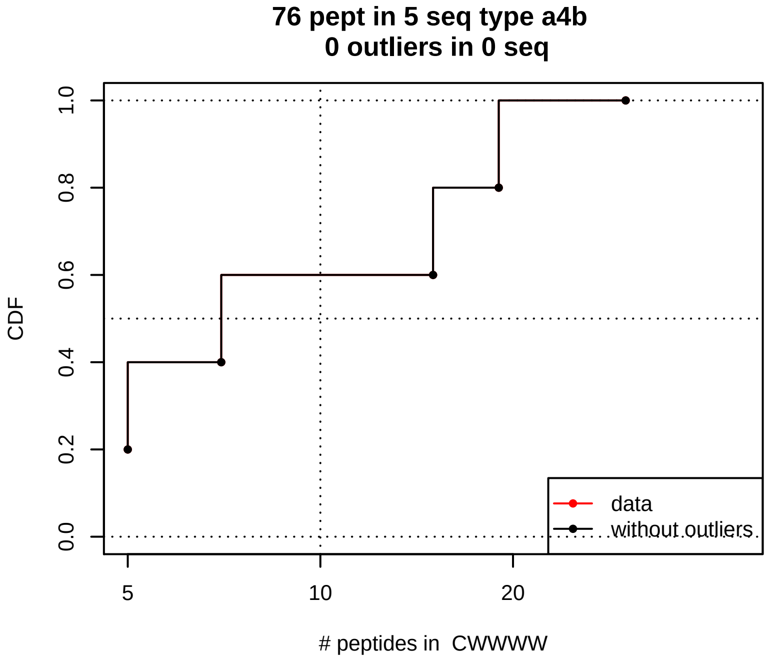
76 pept in 5 seq type a4b
0 outliers in 0 seq
CDF
# peptides in CWWWW
data
without outliers
0.0
0.2
0.4
0.6
0.8
1.0
5
10
20

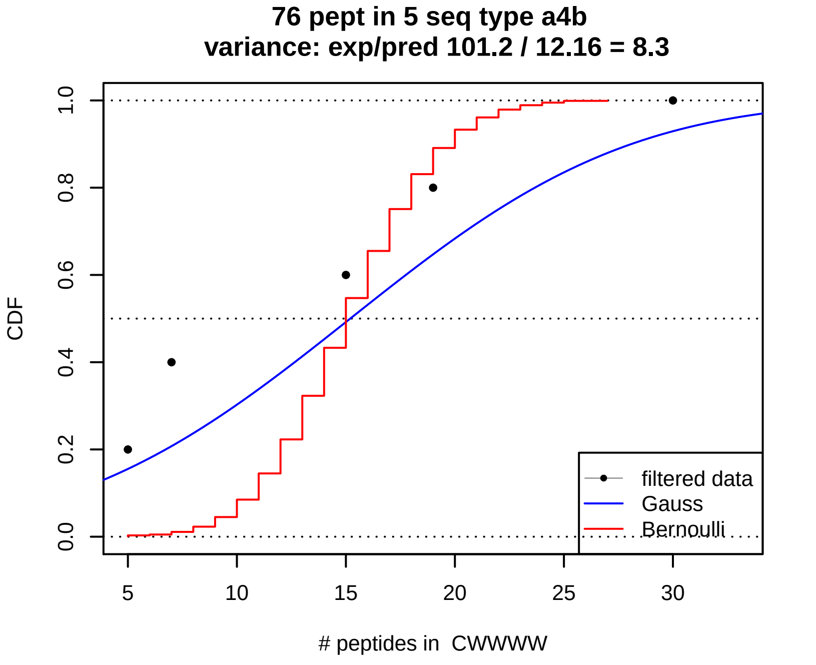
76 pept in 5 seq type a4b
variance: exp/pred 101.2 / 12.16 = 8.3
CDF
# peptides in CWWWW
filtered data
Gauss
Bernoulli
0.0
0.2
0.4
0.6
0.8
1.0
5
10
15
20
25
30

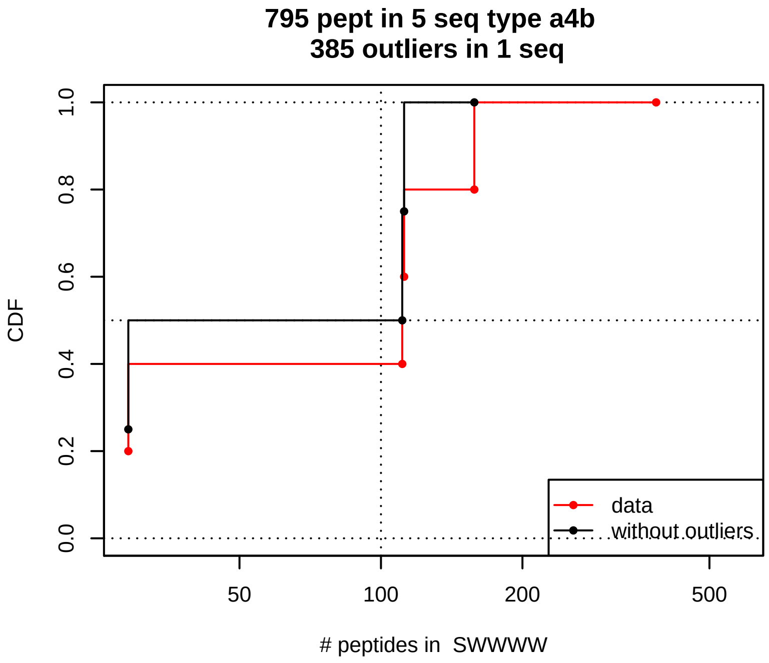
795 pept in 5 seq type a4b
385 outliers in 1 seq
CDF
0.0
0.2
0.4
0.6
0.8
1.0
50
100
200
500
# peptides in SWWWW
data
without outliers

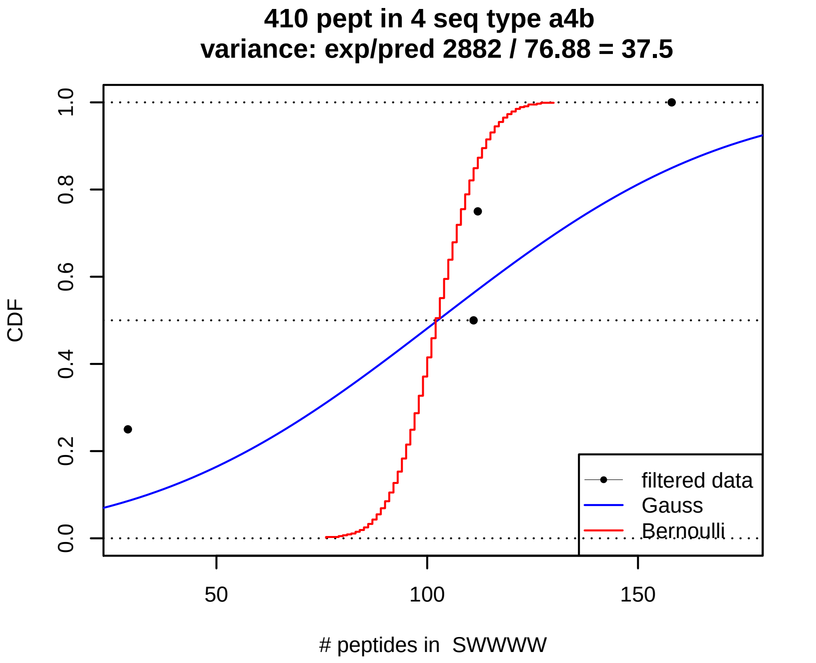
410 pept in 4 seq type a4b
variance: exp/pred 2882 / 76.88 = 37.5
CDF
0.0
0.2
0.4
0.6
0.8
1.0
50
100
150
# peptides in SWWWW
filtered data
Gauss
Bernoulli

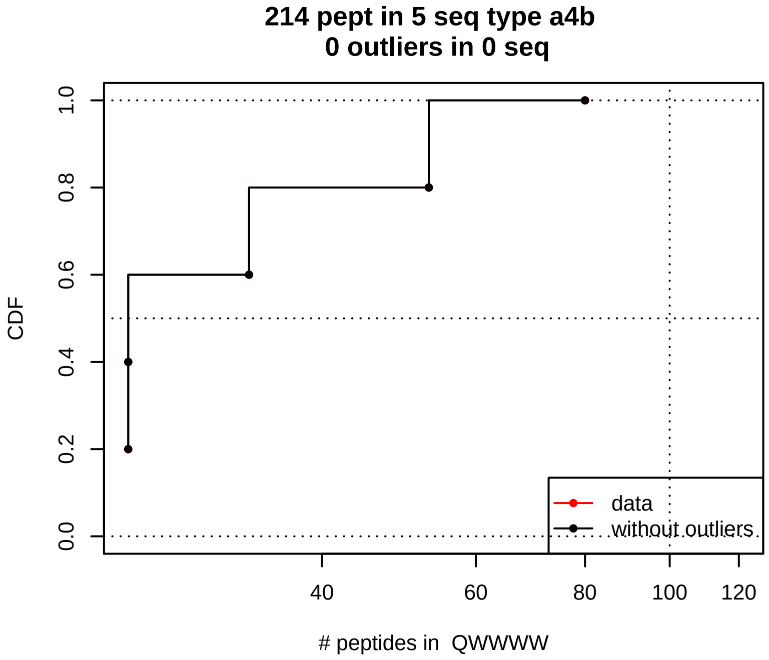
214 pept in 5 seq type a4b
0 outliers in 0 seq
CDF
0.0
0.2
0.4
0.6
0.8
1.0
40
60
80
100
120
# peptides in QWWWW
data
without outliers

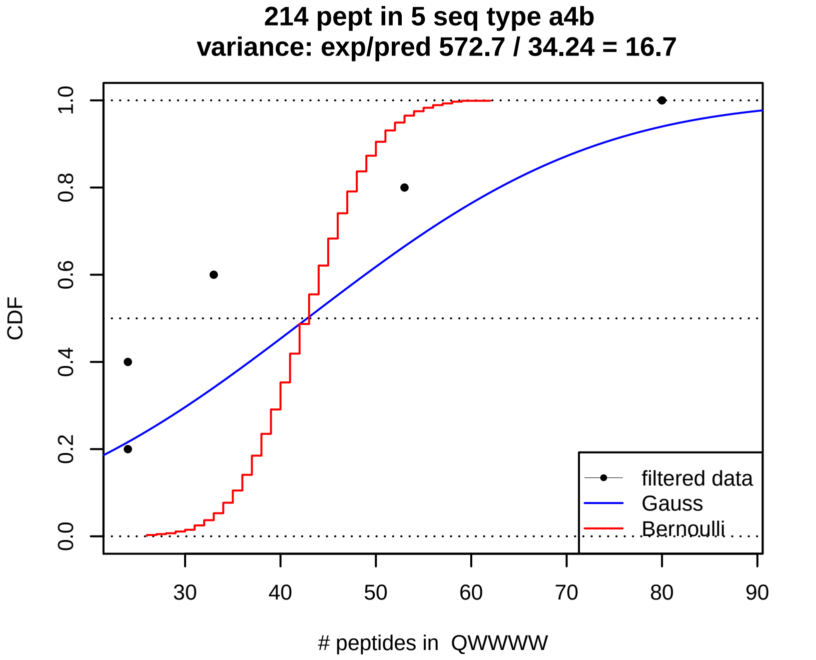
214 pept in 5 seq type a4b
variance: exp/pred 572.7 / 34.24 = 16.7
CDF
0.0
0.2
0.4
0.6
0.8
1.0
30
40
50
60
70
80
90
# peptides in QWWWW
filtered data
Gauss
Bernoulli

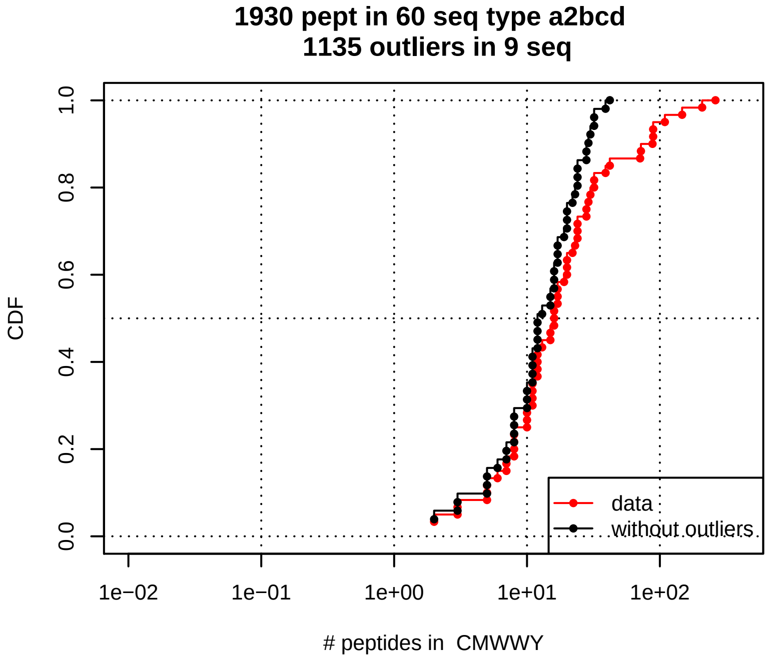
1930 pept in 60 seq type a2bcd
1135 outliers in 9 seq
CDF
0.0
0.2
0.4
0.6
0.8
1.0
1e−02
1e−01
1e+00
1e+01
1e+02
# peptides in CMWWY
data
without outliers

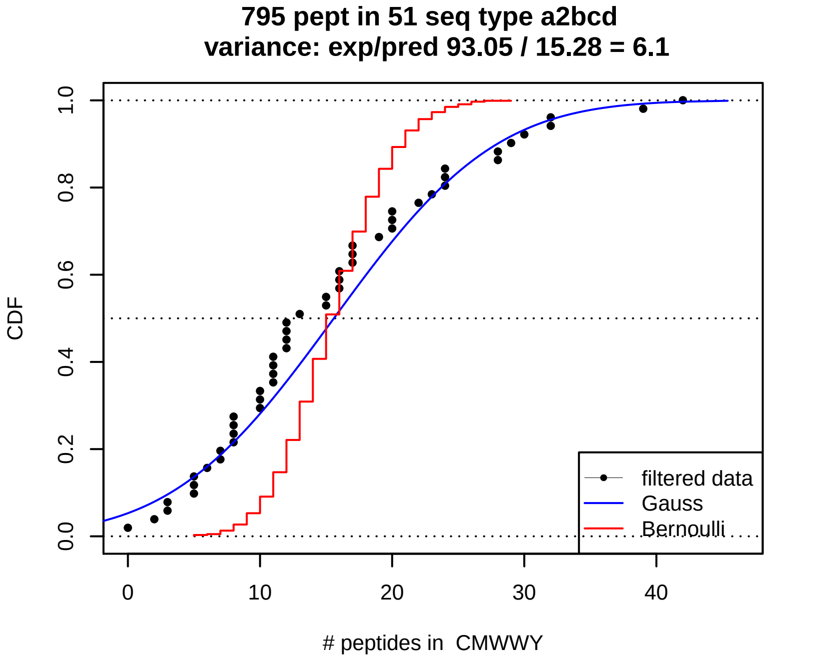
795 pept in 51 seq type a2bcd
variance: exp/pred 93.05 / 15.28 = 6.1
CDF
0.0
0.2
0.4
0.6
0.8
1.0
0
10
20
30
40
# peptides in CMWWY
filtered data
Gauss
Bernoulli

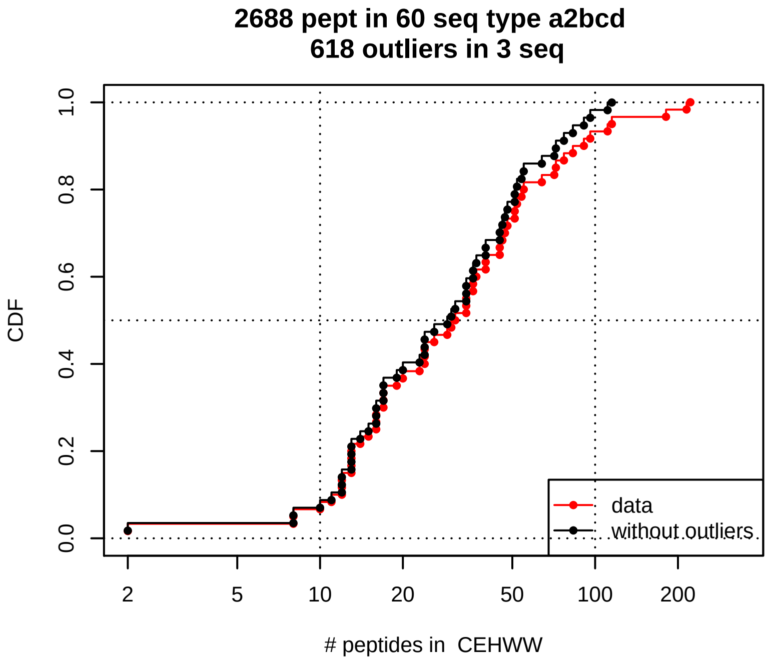
2688 pept in 60 seq type a2bcd
618 outliers in 3 seq
CDF
0.0
0.2
0.4
0.6
0.8
1.0
2
5
10
20
50
100
200
# peptides in CEHWW
data
without outliers

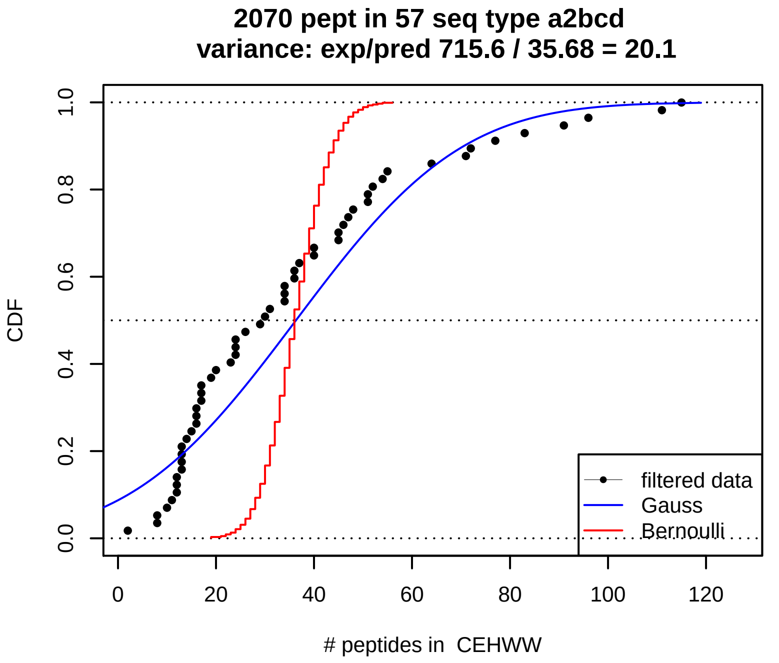
2070 pept in 57 seq type a2bcd
variance: exp/pred 715.6 / 35.68 = 20.1
CDF
0.0
0.2
0.4
0.6
0.8
1.0
0
20
40
60
80
100
120
# peptides in CEHWW
filtered data
Gauss
Bernoulli

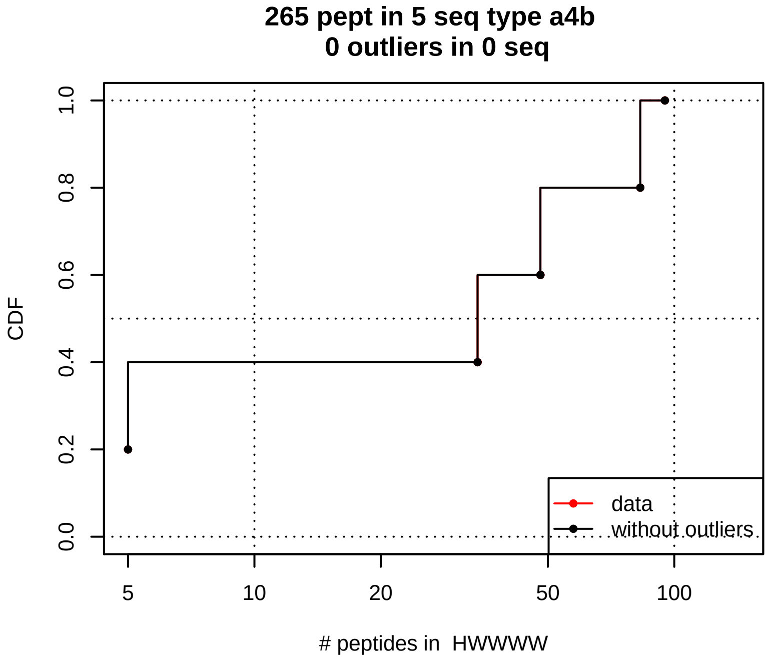
265 pept in 5 seq type a4b
0 outliers in 0 seq
CDF
0.0
0.2
0.4
0.6
0.8
1.0
5
10
20
50
100
# peptides in HWWWW
data
without outliers

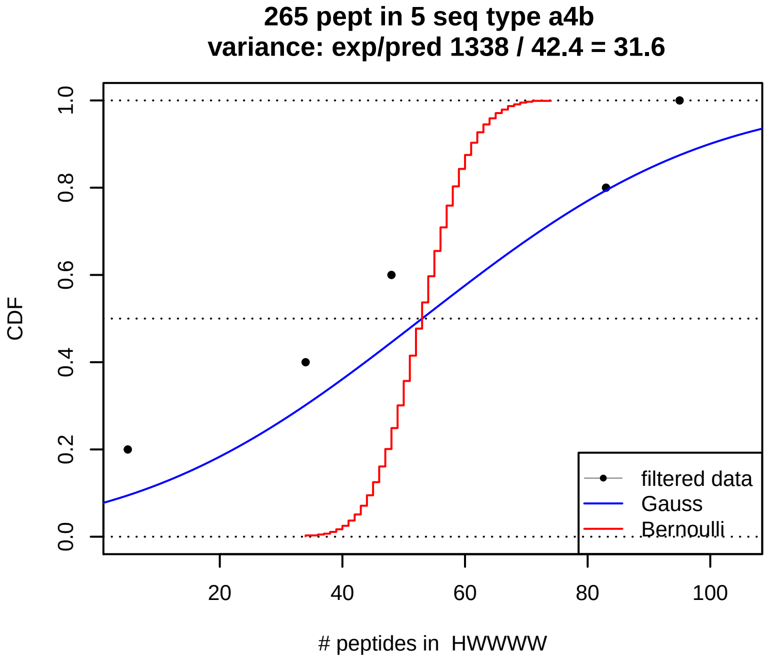
265 pept in 5 seq type a4b
variance: exp/pred 1338 / 42.4 = 31.6
CDF
0.0
0.2
0.4
0.6
0.8
1.0
20
40
60
80
100
# peptides in HWWWW
filtered data
Gauss
Bernoulli

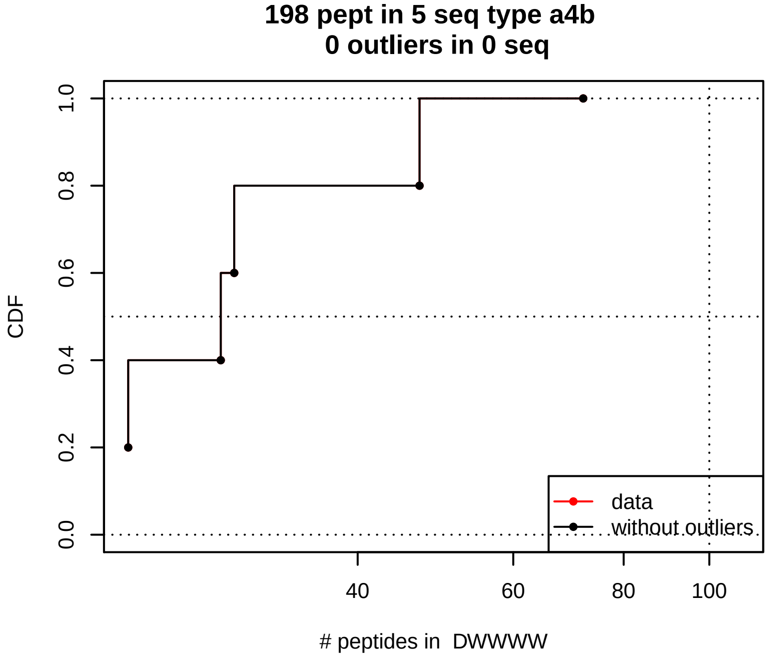
198 pept in 5 seq type a4b
0 outliers in 0 seq
CDF
0.0
0.2
0.4
0.6
0.8
1.0
40
60
80
100
# peptides in DWWWW
data
without outliers

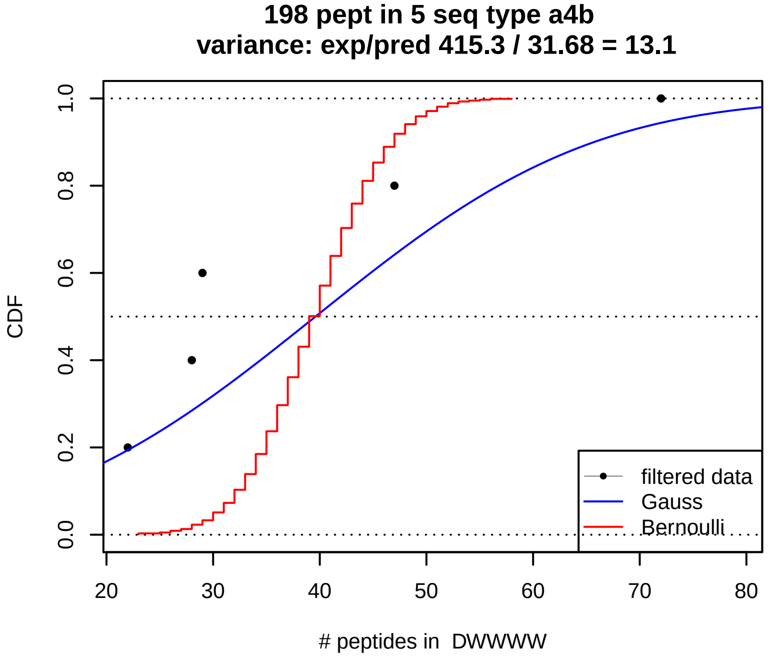
198 pept in 5 seq type a4b
variance: exp/pred 415.3 / 31.68 = 13.1
CDF
0.0
0.2
0.4
0.6
0.8
1.0
20
30
40
50
60
70
80
# peptides in DWWWW
filtered data
Gauss
Bernoulli

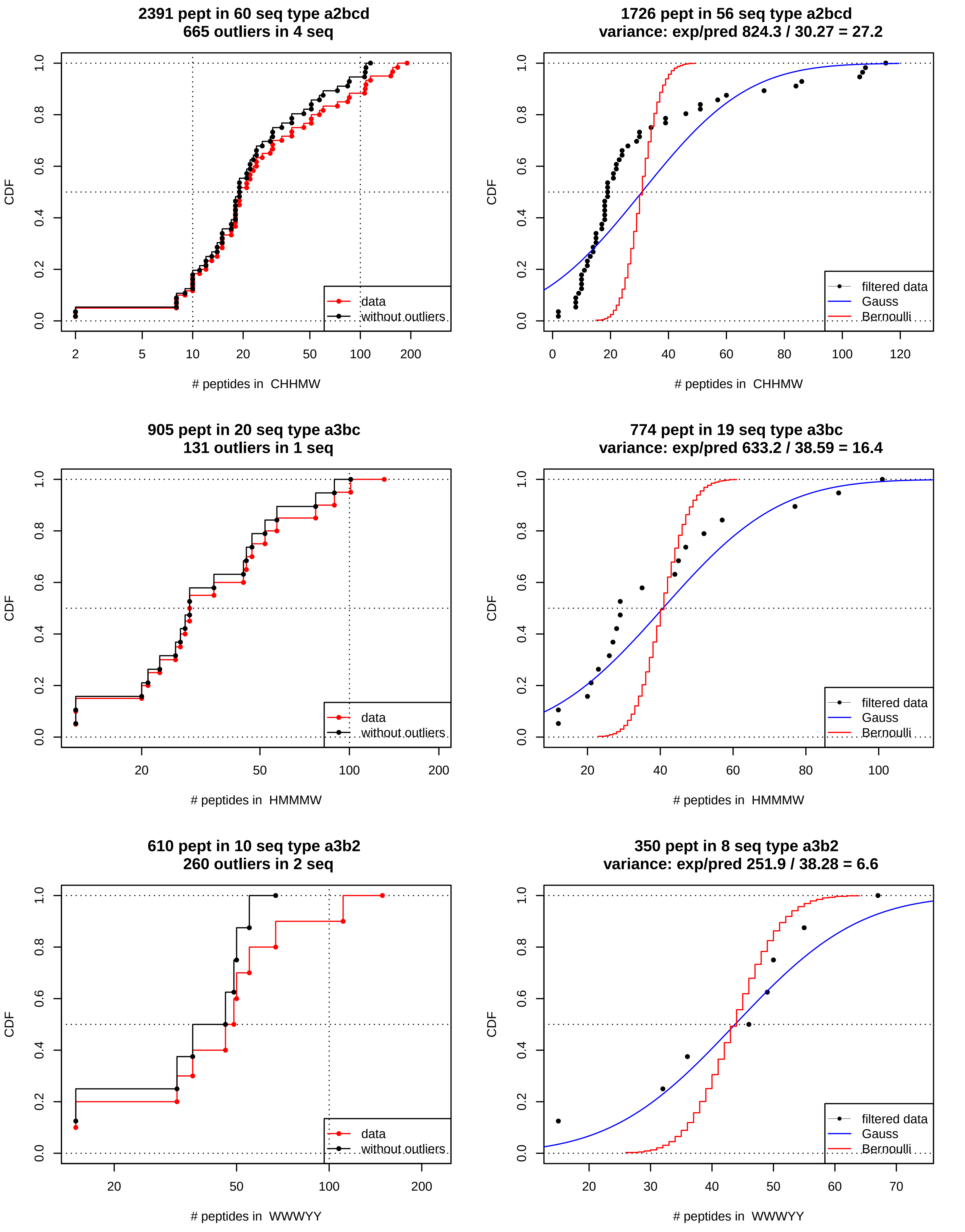
2391 pept in 60 seq type a2bcd
665 outliers in 4 seq
CDF
# peptides in CHHMW
data
without outliers
1726 pept in 56 seq type a2bcd
variance: exp/pred 824.3 / 30.27 = 27.2
CDF
# peptides in CHHMW
filtered data
Gauss
Bernoulli
905 pept in 20 seq type a3bc
131 outliers in 1 seq
CDF
# peptides in HMMMW
data
without outliers
774 pept in 19 seq type a3bc
variance: exp/pred 633.2 / 38.59 = 16.4
CDF
# peptides in HMMMW
filtered data
Gauss
Bernoulli
610 pept in 10 seq type a3b2
260 outliers in 2 seq
CDF
# peptides in WWWYY
data
without outliers
350 pept in 8 seq type a3b2
variance: exp/pred 251.9 / 38.28 = 6.6
CDF
# peptides in WWWYY
filtered data
Gauss
Bernoulli

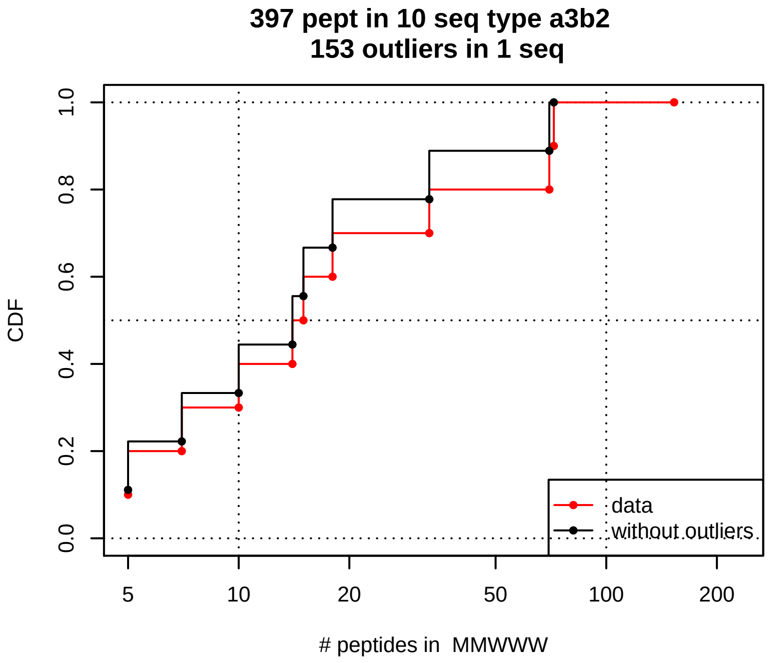
397 pept in 10 seq type a3b2
153 outliers in 1 seq
CDF
0.0 0.2 0.4 0.6 0.8 1.0
5 10 20 50 100 200
# peptides in MMWWW
data
without outliers

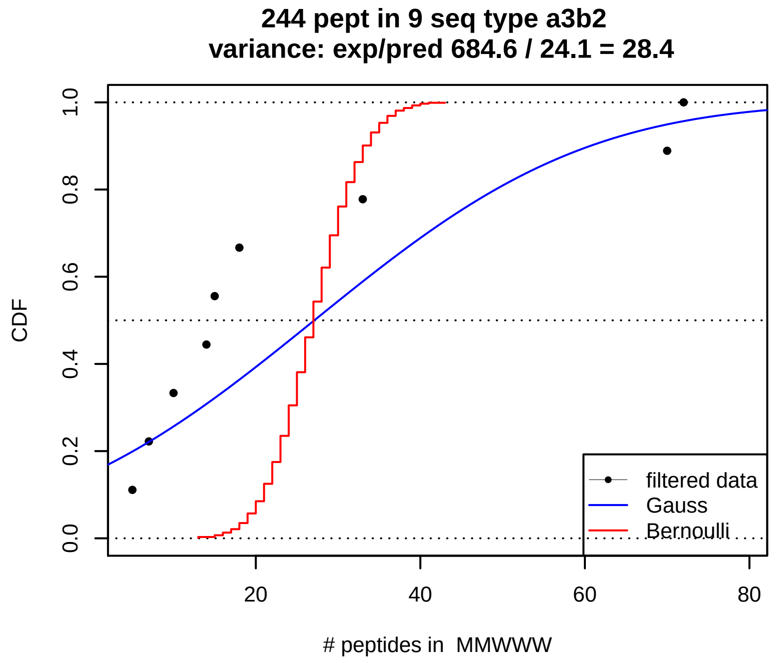
244 pept in 9 seq type a3b2
variance: exp/pred 684.6 / 24.1 = 28.4
CDF
0.0 0.2 0.4 0.6 0.8 1.0
20 40 60 80
# peptides in MMWWW
filtered data
Gauss
Bernoulli

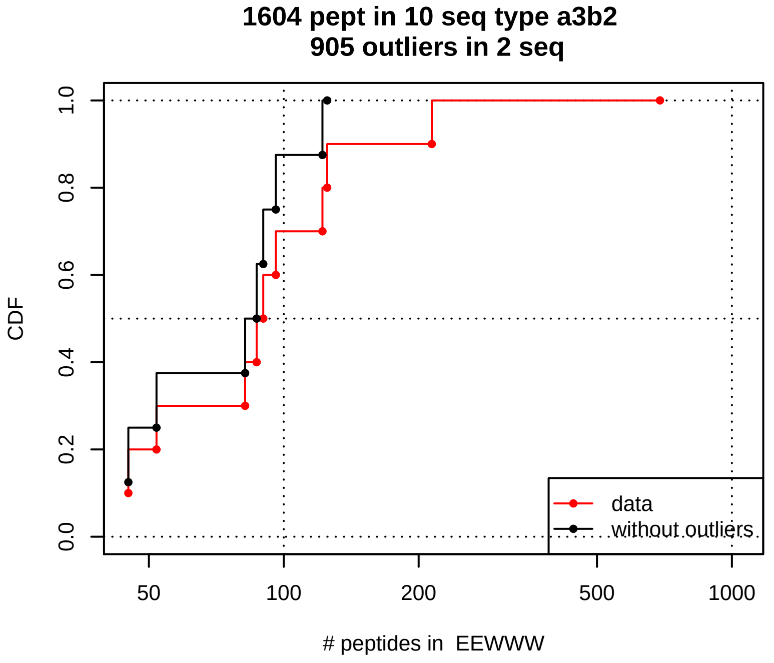
1604 pept in 10 seq type a3b2
905 outliers in 2 seq
CDF
0.0 0.2 0.4 0.6 0.8 1.0
50 100 200 500 1000
# peptides in EEWWW
data
without outliers

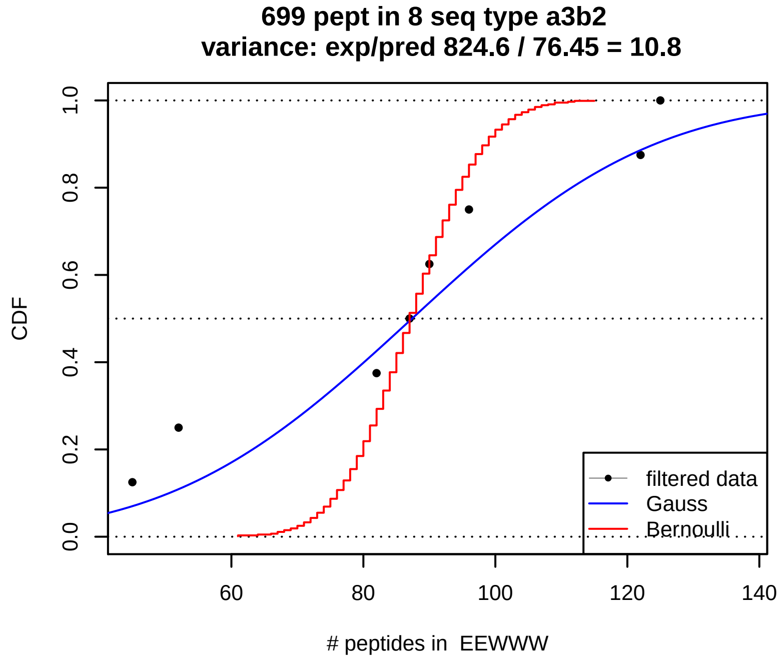
699 pept in 8 seq type a3b2
variance: exp/pred 824.6 / 76.45 = 10.8
CDF
0.0 0.2 0.4 0.6 0.8 1.0
60 80 100 120 140
# peptides in EEWWW
filtered data
Gauss
Bernoulli

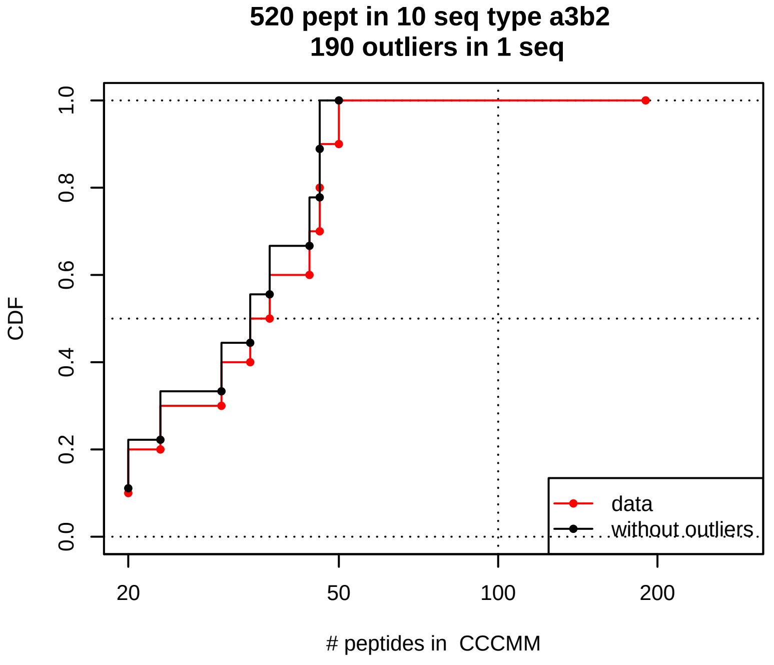
520 pept in 10 seq type a3b2
190 outliers in 1 seq
CDF
0.0 0.2 0.4 0.6 0.8 1.0
20 50 100 200
# peptides in CCCMM
data
without outliers

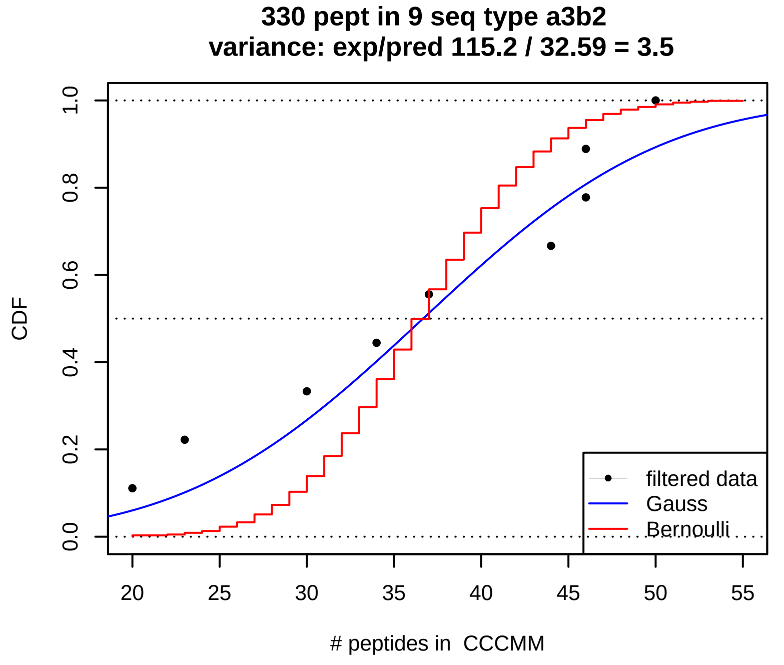
330 pept in 9 seq type a3b2
variance: exp/pred 115.2 / 32.59 = 3.5
CDF
0.0 0.2 0.4 0.6 0.8 1.0
20 25 30 35 40 45 50 55
# peptides in CCCMM
filtered data
Gauss
Bernoulli

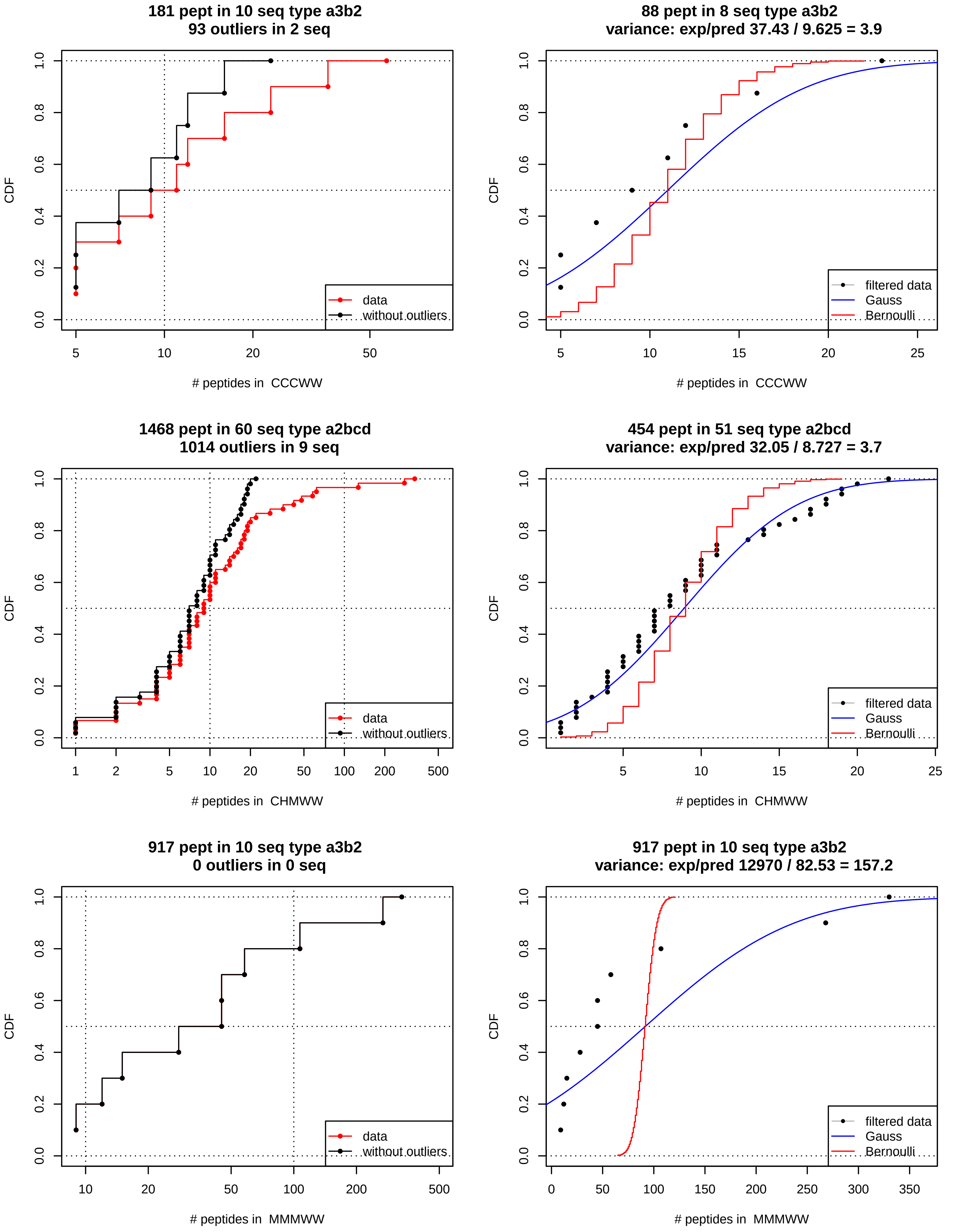

181 pept in 10 seq type a3b2
93 outliers in 2 seq
CDF
0.0
0.2
0.4
0.6
0.8
1.0
5
10
20
50
# peptides in CCCWW
data
without outliers
88 pept in 8 seq type a3b2
variance: exp/pred 37.43 / 9.625 = 3.9
CDF
0.0
0.2
0.4
0.6
0.8
1.0
5
10
15
20
25
# peptides in CCCWW
filtered data
Gauss
Bernoulli
1468 pept in 60 seq type a2bcd
1014 outliers in 9 seq
CDF
0.0
0.2
0.4
0.6
0.8
1.0
1
2
5
10
20
50
100
200
500
# peptides in CHMWW
data
without outliers
454 pept in 51 seq type a2bcd
variance: exp/pred 32.05 / 8.727 = 3.7
CDF
0.0
0.2
0.4
0.6
0.8
1.0
5
10
15
20
25
# peptides in CHMWW
filtered data
Gauss
Bernoulli
917 pept in 10 seq type a3b2
0 outliers in 0 seq
CDF
0.0
0.2
0.4
0.6
0.8
1.0
10
20
50
100
200
500
# peptides in MMMWW
data
without outliers
917 pept in 10 seq type a3b2
variance: exp/pred 12970 / 82.53 = 157.2
CDF
0.0
0.2
0.4
0.6
0.8
1.0
0
50
100
150
200
250
300
350
# peptides in MMMWW
filtered data
Gauss
Bernoulli

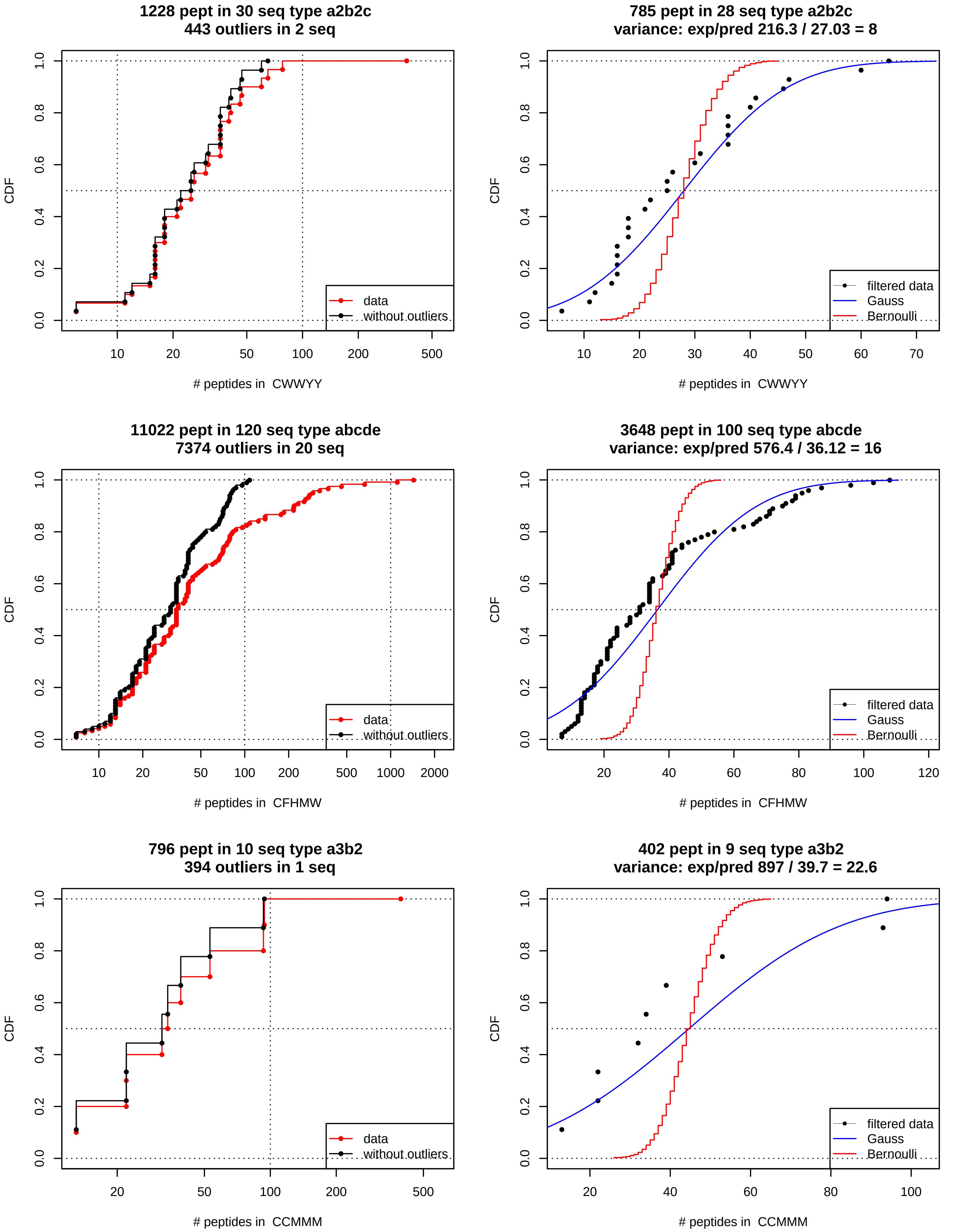
1228 pept in 30 seq type a2b2c
443 outliers in 2 seq
CDF
# peptides in CWWYY
data
without outliers
785 pept in 28 seq type a2b2c
variance: exp/pred 216.3 / 27.03 = 8
CDF
# peptides in CWWYY
filtered data
Gauss
Bernoulli
11022 pept in 120 seq type abcde
7374 outliers in 20 seq
CDF
# peptides in CFHMW
data
without outliers
3648 pept in 100 seq type abcde
variance: exp/pred 576.4 / 36.12 = 16
CDF
# peptides in CFHMW
filtered data
Gauss
Bernoulli
796 pept in 10 seq type a3b2
394 outliers in 1 seq
CDF
# peptides in CCMMM
data
without outliers
402 pept in 9 seq type a3b2
variance: exp/pred 897 / 39.7 = 22.6
CDF
# peptides in CCMMM
filtered data
Gauss
Bernoulli

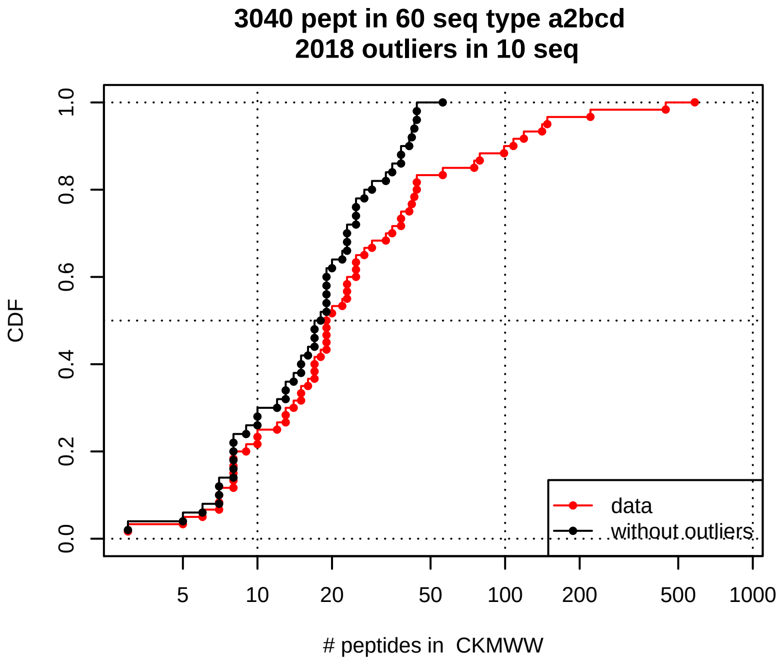
3040 pept in 60 seq type a2bcd
2018 outliers in 10 seq
CDF
# peptides in CKMWW
data
without outliers

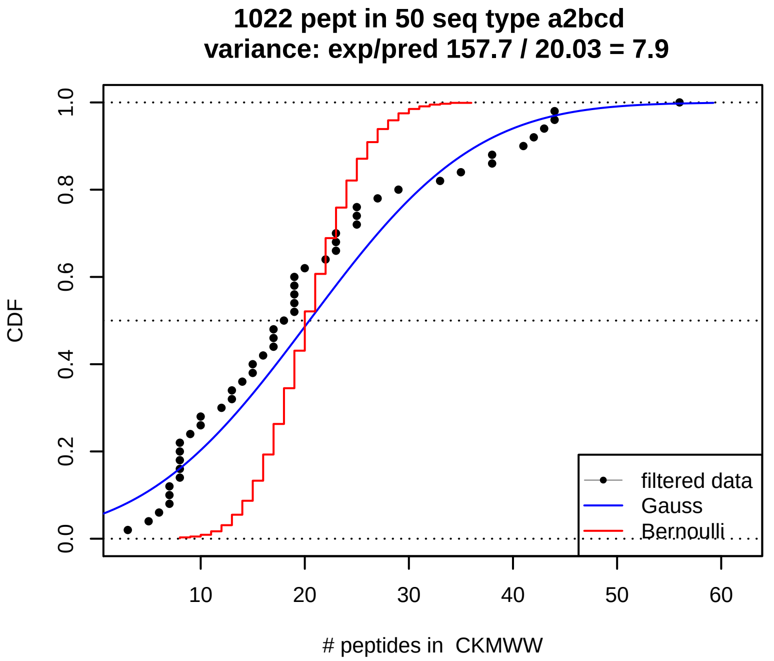
1022 pept in 50 seq type a2bcd
variance: exp/pred 157.7 / 20.03 = 7.9
CDF
# peptides in CKMWW
filtered data
Gauss
Bernoulli

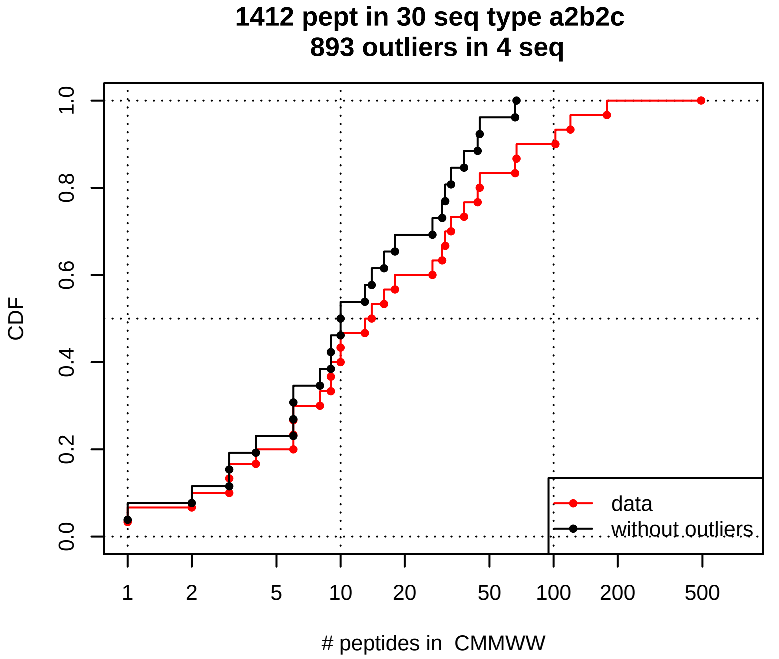
1412 pept in 30 seq type a2b2c
893 outliers in 4 seq
CDF
# peptides in CMMWW
data
without outliers

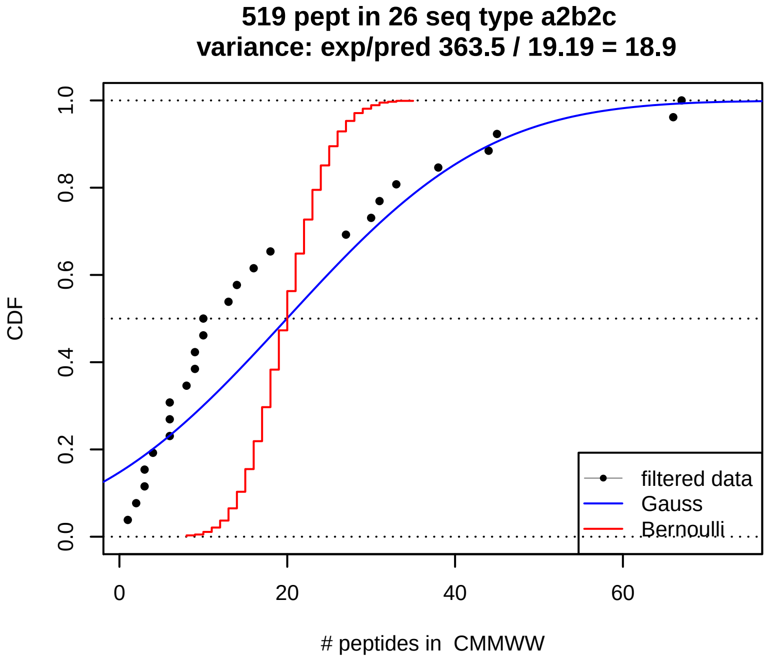
519 pept in 26 seq type a2b2c
variance: exp/pred 363.5 / 19.19 = 18.9
CDF
# peptides in CMMWW
filtered data
Gauss
Bernoulli

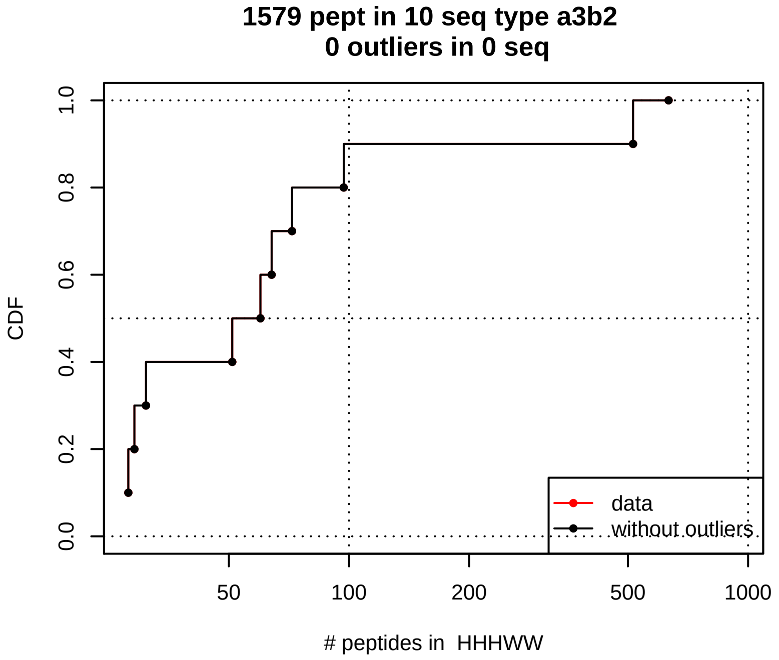
1579 pept in 10 seq type a3b2
0 outliers in 0 seq
CDF
# peptides in HHHWW
data
without outliers

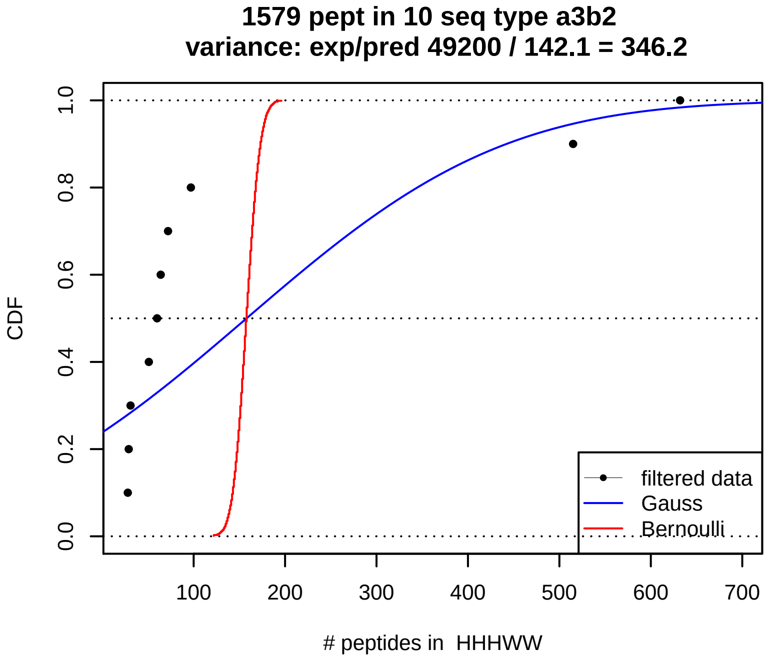
1579 pept in 10 seq type a3b2
variance: exp/pred 49200 / 142.1 = 346.2
CDF
# peptides in HHHWW
filtered data
Gauss
Bernoulli

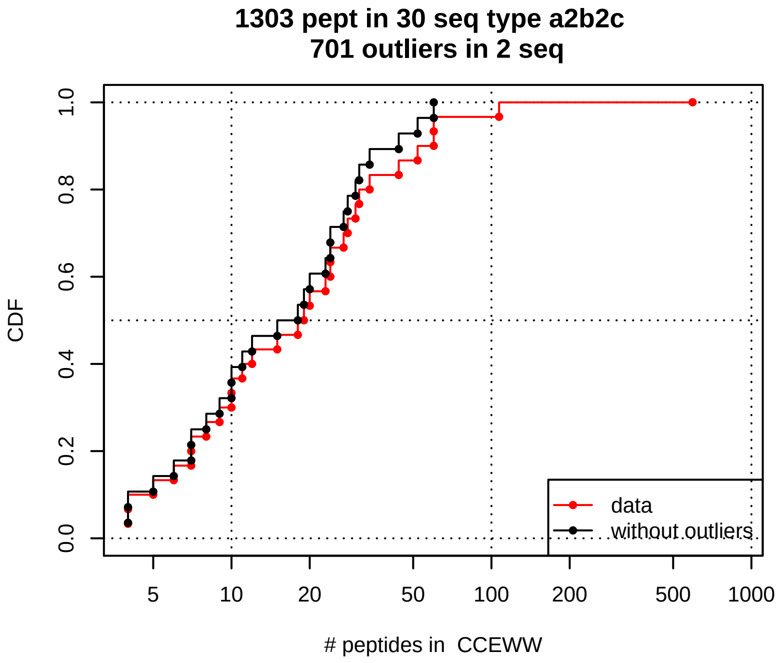
1303 pept in 30 seq type a2b2c
701 outliers in 2 seq
CDF
0.0
0.2
0.4
0.6
0.8
1.0
5
10
20
50
100
200
500
1000
# peptides in CCEWW
data
without outliers

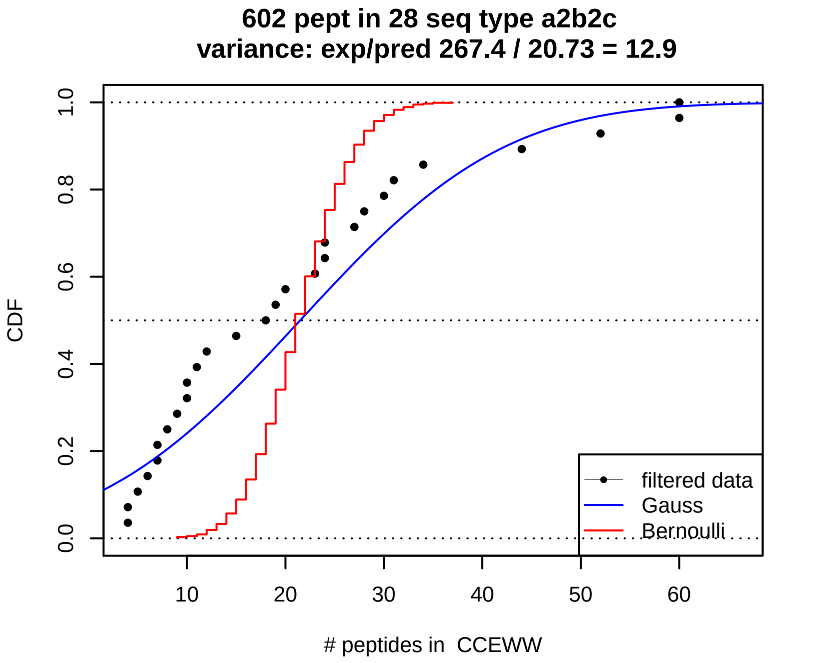
602 pept in 28 seq type a2b2c
variance: exp/pred 267.4 / 20.73 = 12.9
CDF
0.0
0.2
0.4
0.6
0.8
1.0
10
20
30
40
50
60
# peptides in CCEWW
filtered data
Gauss
Bernoulli

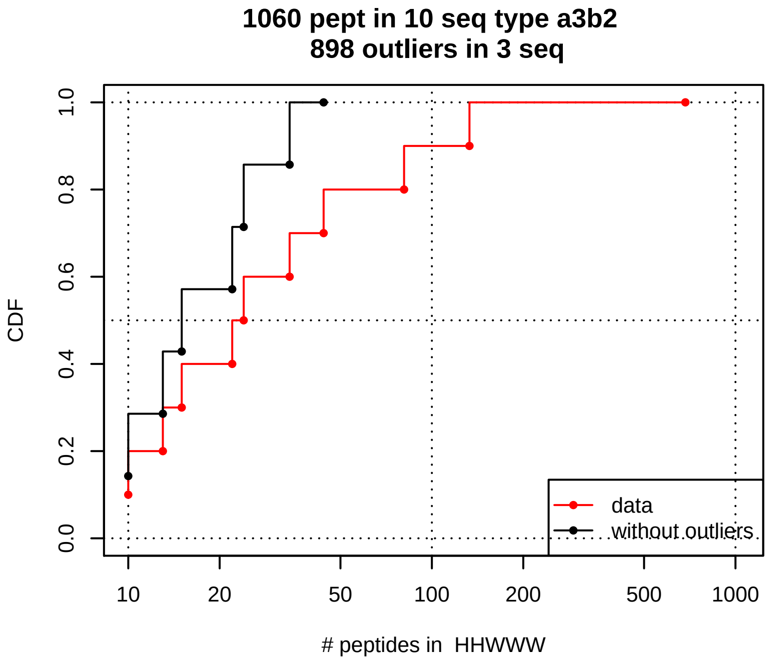
1060 pept in 10 seq type a3b2
898 outliers in 3 seq
CDF
0.0
0.2
0.4
0.6
0.8
1.0
10
20
50
100
200
500
1000
# peptides in HHWWW
data
without outliers

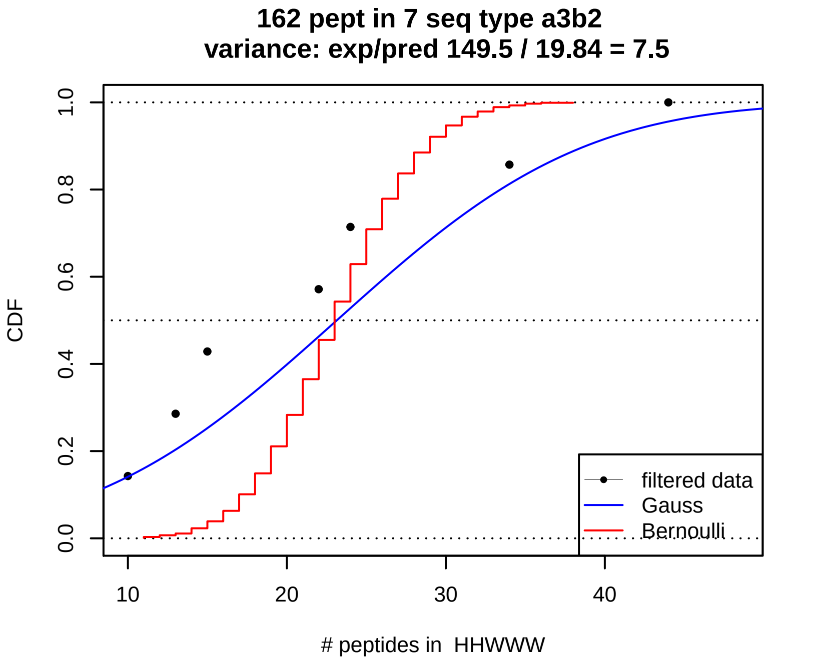
162 pept in 7 seq type a3b2
variance: exp/pred 149.5 / 19.84 = 7.5
CDF
0.0
0.2
0.4
0.6
0.8
1.0
10
20
30
40
# peptides in HHWWW
filtered data
Gauss
Bernoulli

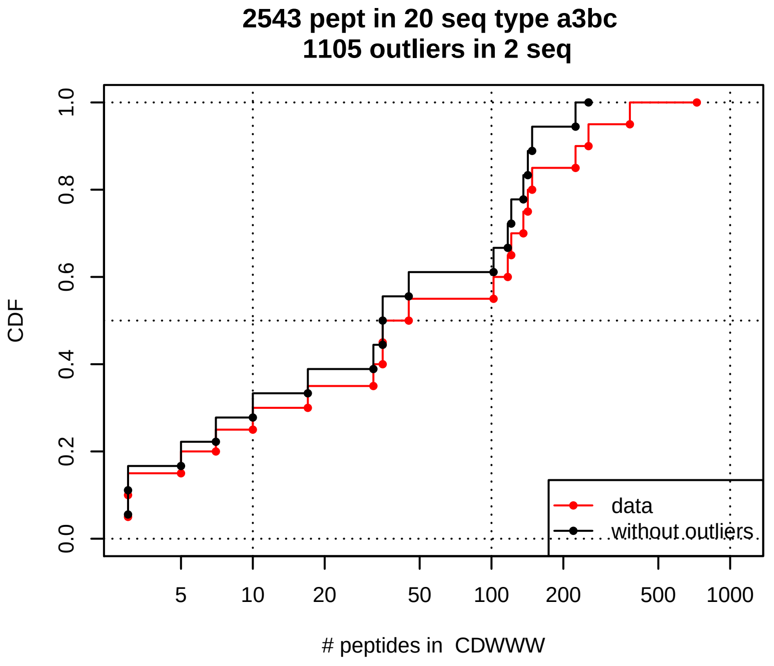
2543 pept in 20 seq type a3bc
1105 outliers in 2 seq
CDF
0.0
0.2
0.4
0.6
0.8
1.0
5
10
20
50
100
200
500
1000
# peptides in CDWWW
data
without outliers

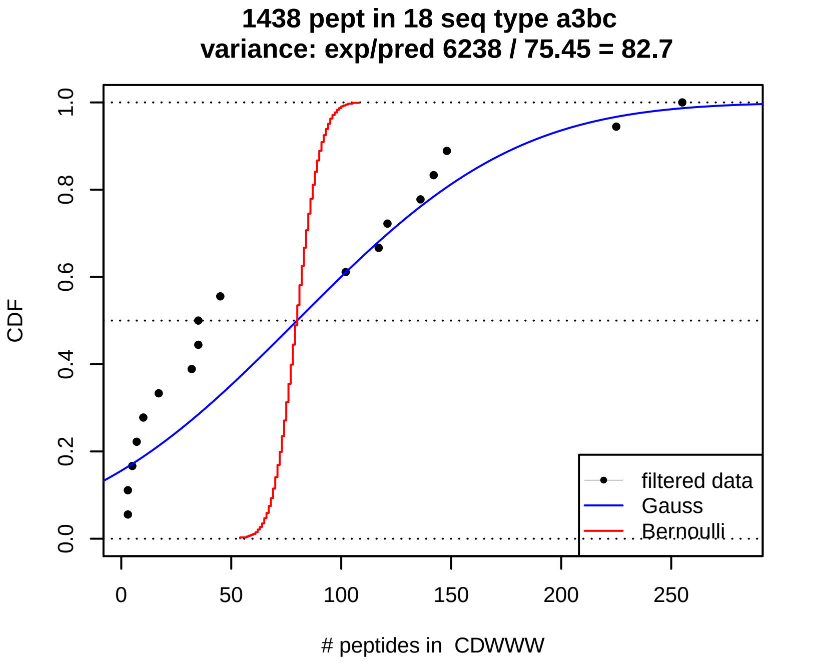
1438 pept in 18 seq type a3bc
variance: exp/pred 6238 / 75.45 = 82.7
CDF
0.0
0.2
0.4
0.6
0.8
1.0
0
50
100
150
200
250
# peptides in CDWWW
filtered data
Gauss
Bernoulli

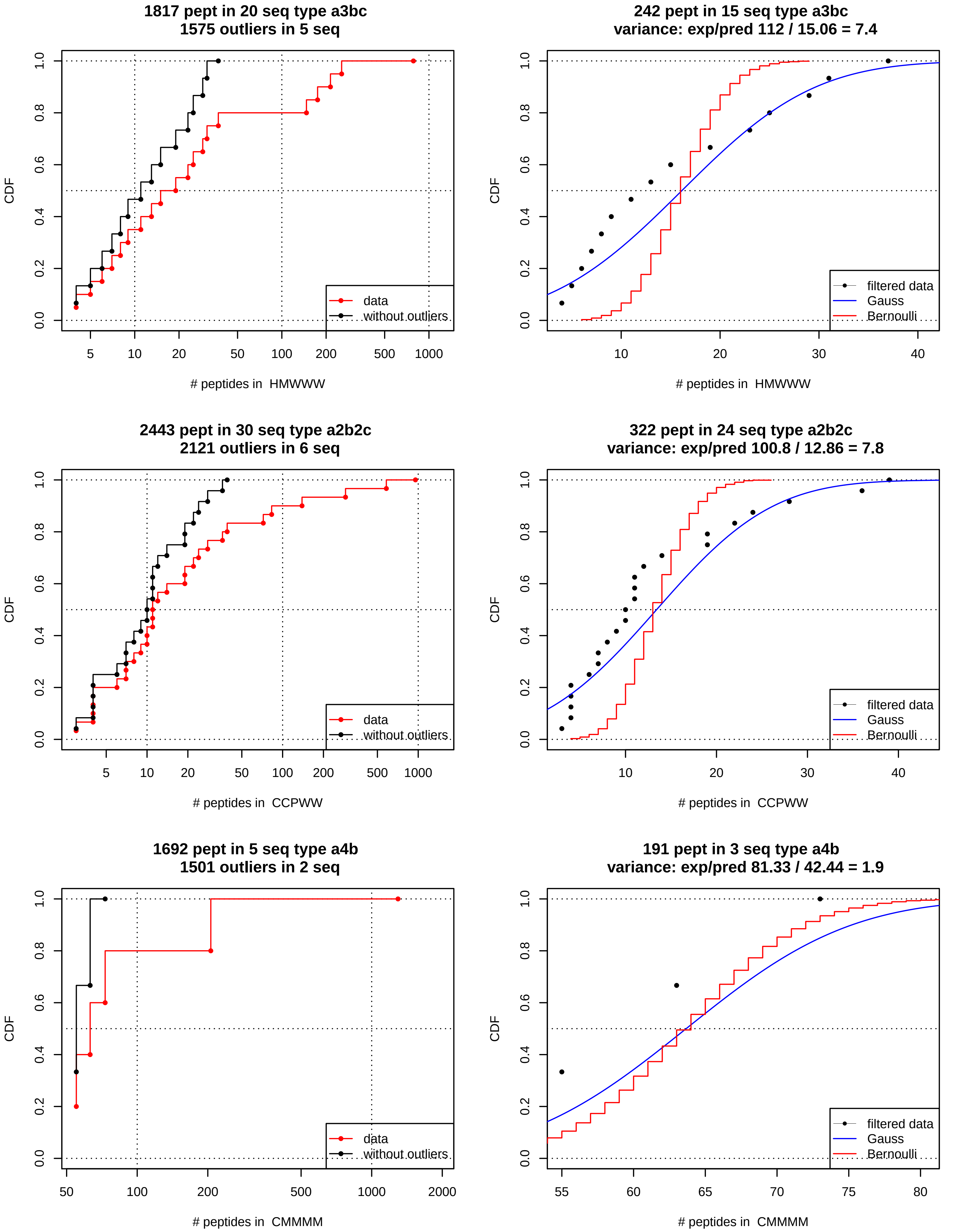

1817 pept in 20 seq type a3bc
1575 outliers in 5 seq
CDF
# peptides in HMWWW
data
without outliers
242 pept in 15 seq type a3bc
variance: exp/pred 112 / 15.06 = 7.4
filtered data
Gauss
Bernoulli
2443 pept in 30 seq type a2b2c
2121 outliers in 6 seq
# peptides in CCPWW
322 pept in 24 seq type a2b2c
variance: exp/pred 100.8 / 12.86 = 7.8
1692 pept in 5 seq type a4b
1501 outliers in 2 seq
# peptides in CMMMM
191 pept in 3 seq type a4b
variance: exp/pred 81.33 / 42.44 = 1.9

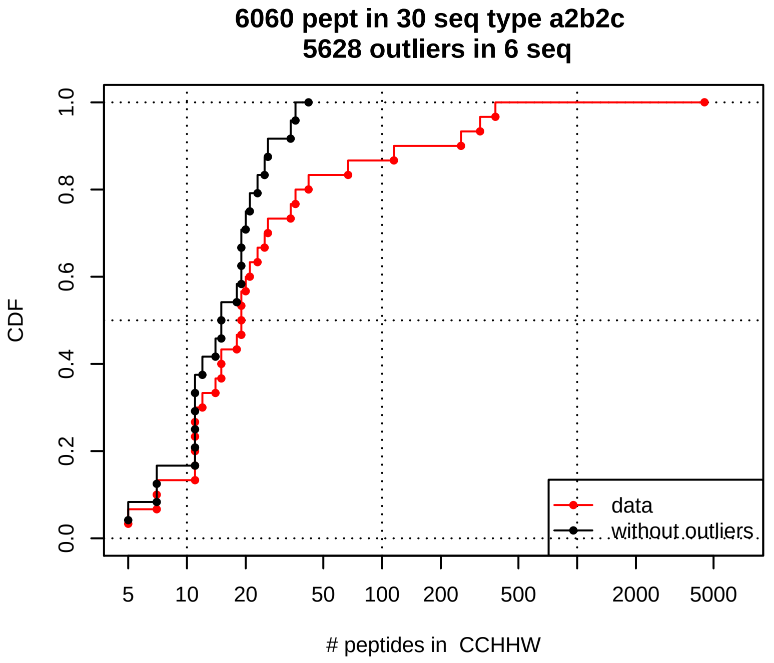
6060 pept in 30 seq type a2b2c
5628 outliers in 6 seq
CDF
# peptides in CCHHW
data
without outliers

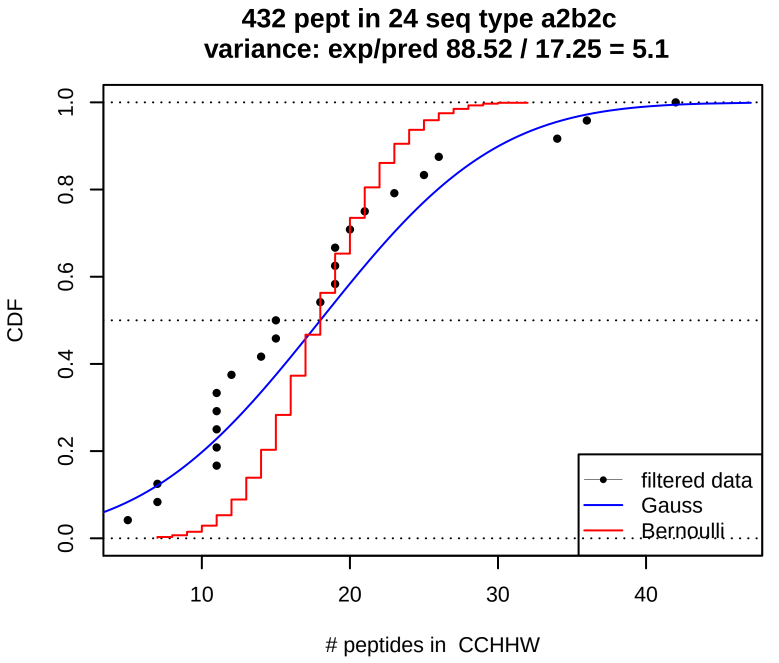
432 pept in 24 seq type a2b2c
variance: exp/pred 88.52 / 17.25 = 5.1
CDF
# peptides in CCHHW
filtered data
Gauss
Bernoulli

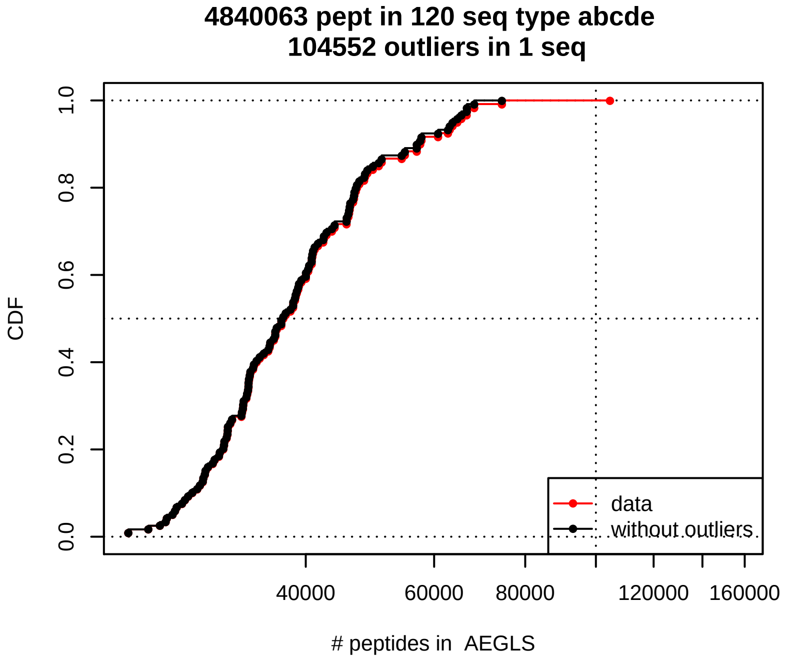
4840063 pept in 120 seq type abcde
104552 outliers in 1 seq
CDF
# peptides in AEGLS
data
without outliers

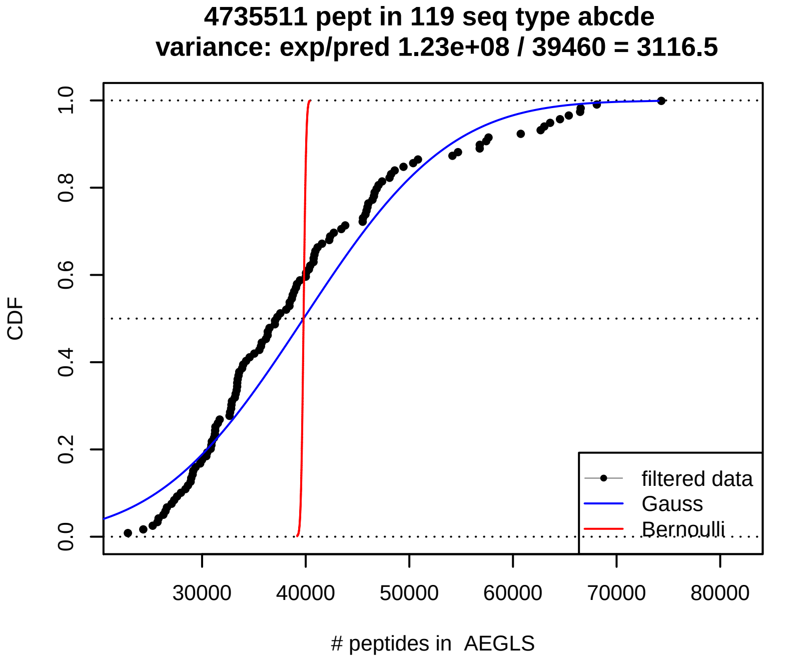
4735511 pept in 119 seq type abcde
variance: exp/pred 1.23e+08 / 39460 = 3116.5
CDF
# peptides in AEGLS
filtered data
Gauss
Bernoulli

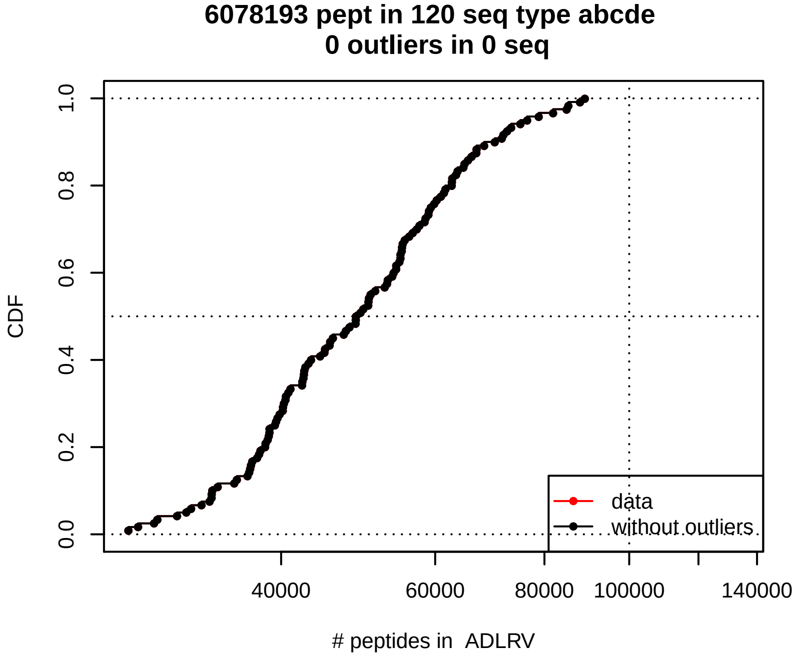
6078193 pept in 120 seq type abcde
0 outliers in 0 seq
CDF
# peptides in ADLRV
data
without outliers

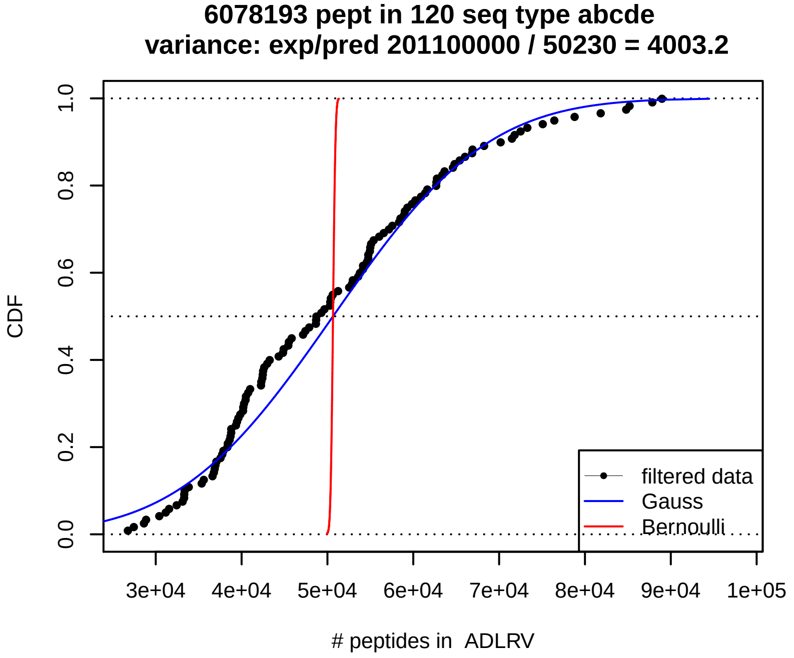
6078193 pept in 120 seq type abcde
variance: exp/pred 201100000 / 50230 = 4003.2
CDF
# peptides in ADLRV
filtered data
Gauss
Bernoulli

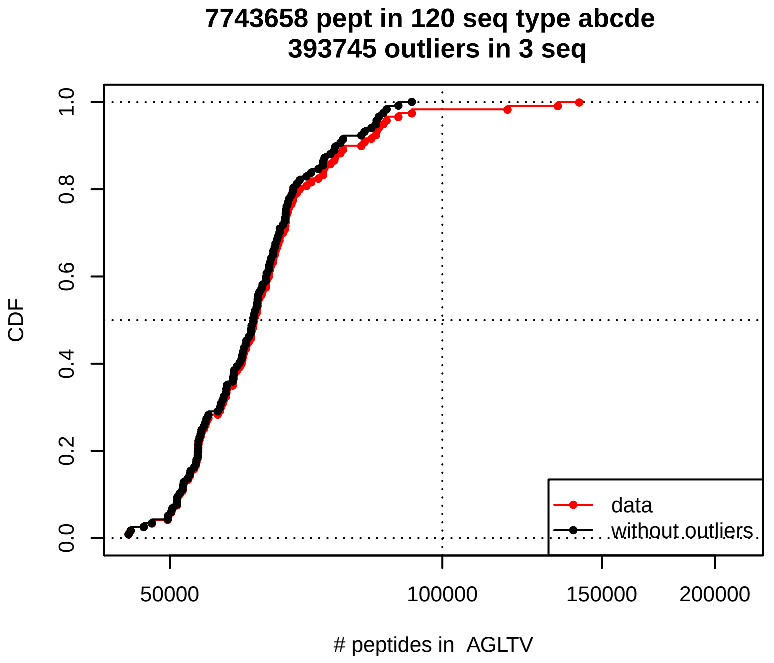
7743658 pept in 120 seq type abcde
393745 outliers in 3 seq
CDF
0.0 0.2 0.4 0.6 0.8 1.0
50000 100000 150000 200000
# peptides in AGLTV
data
without outliers

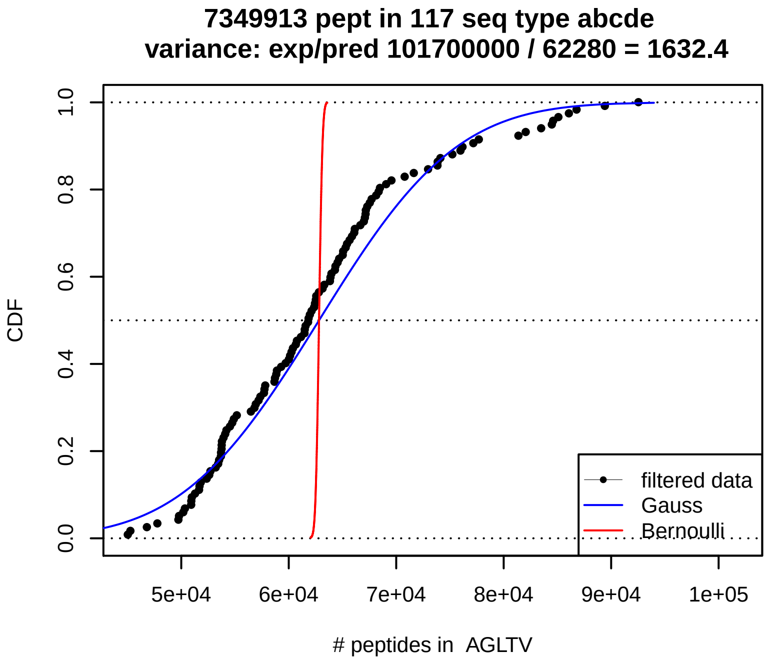
7349913 pept in 117 seq type abcde
variance: exp/pred 101700000 / 62280 = 1632.4
CDF
0.0 0.2 0.4 0.6 0.8 1.0
5e+04 6e+04 7e+04 8e+04 9e+04 1e+05
# peptides in AGLTV
filtered data
Gauss
Bernoulli

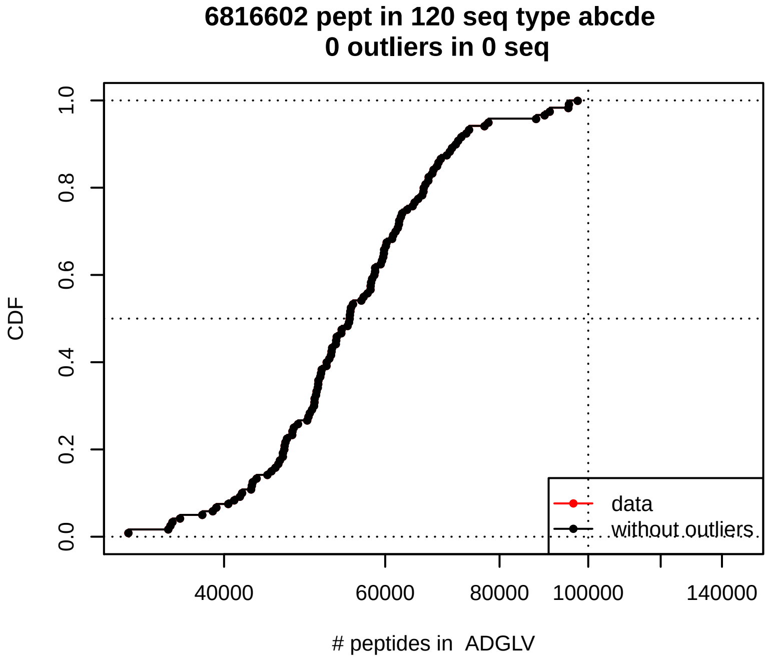
6816602 pept in 120 seq type abcde
0 outliers in 0 seq
CDF
0.0 0.2 0.4 0.6 0.8 1.0
40000 60000 80000 100000 140000
# peptides in ADGLV
data
without outliers

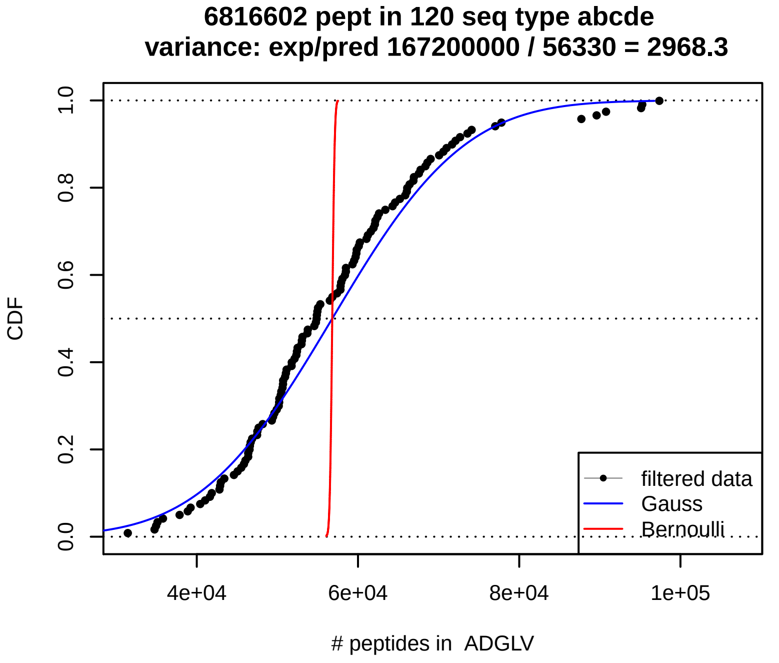
6816602 pept in 120 seq type abcde
variance: exp/pred 167200000 / 56330 = 2968.3
CDF
0.0 0.2 0.4 0.6 0.8 1.0
4e+04 6e+04 8e+04 1e+05
# peptides in ADGLV
filtered data
Gauss
Bernoulli

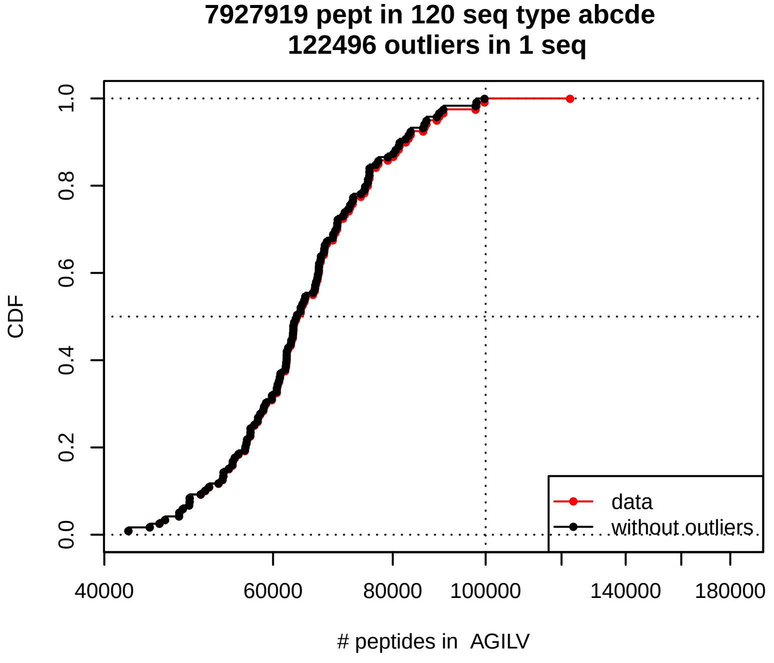
7927919 pept in 120 seq type abcde
122496 outliers in 1 seq
CDF
0.0 0.2 0.4 0.6 0.8 1.0
40000 60000 80000 100000 140000 180000
# peptides in AGILV
data
without outliers

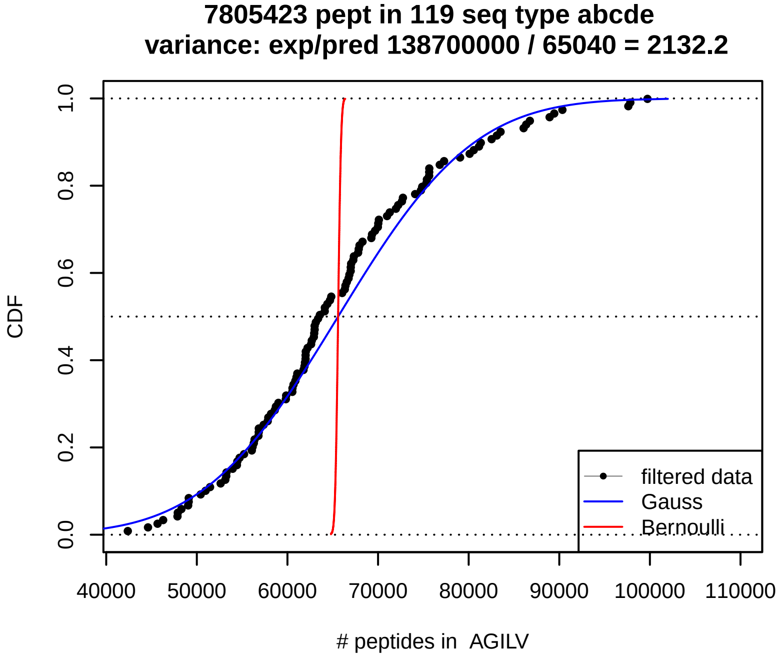
7805423 pept in 119 seq type abcde
variance: exp/pred 138700000 / 65040 = 2132.2
CDF
0.0 0.2 0.4 0.6 0.8 1.0
40000 50000 60000 70000 80000 90000 100000 110000
# peptides in AGILV
filtered data
Gauss
Bernoulli

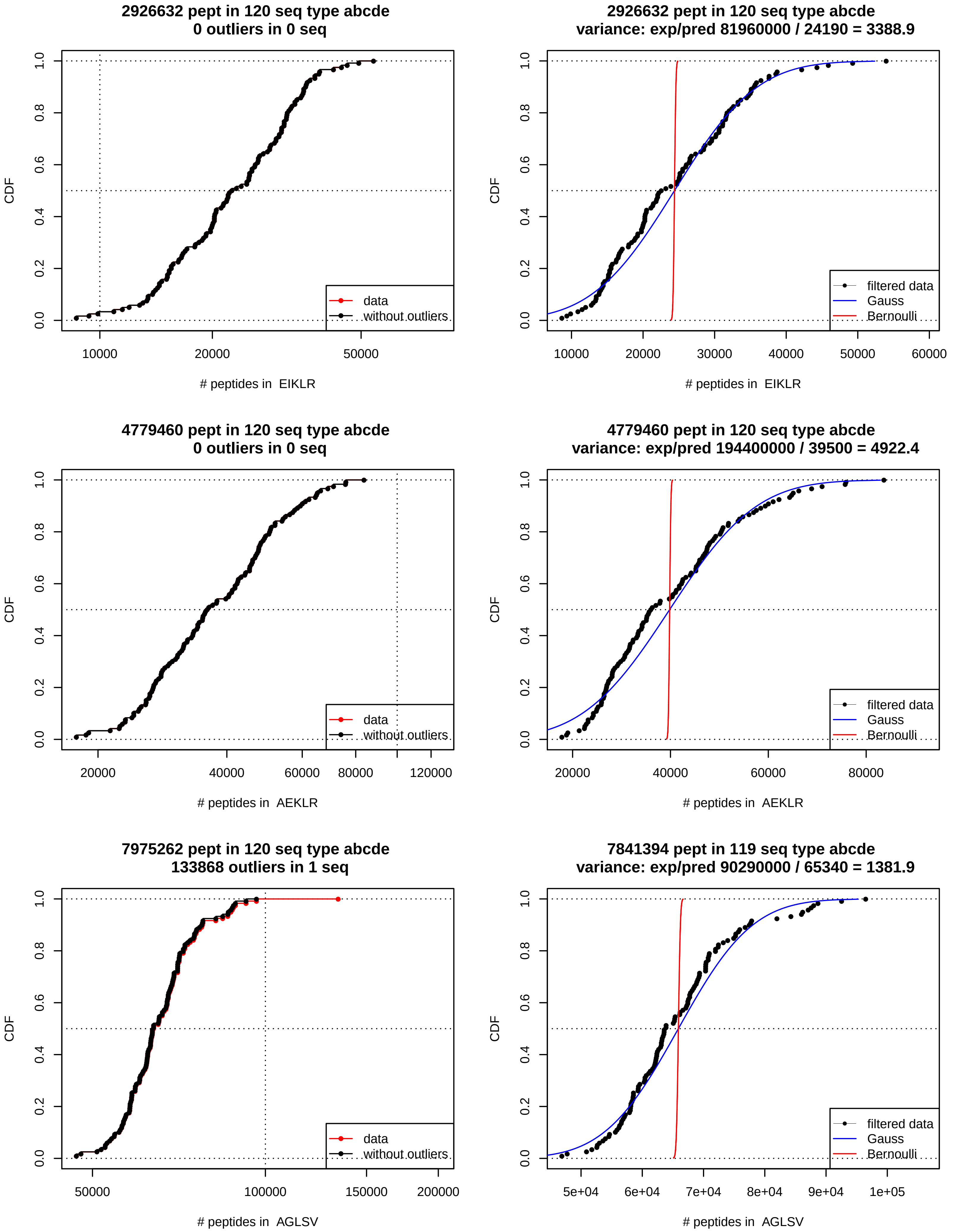
2926632 pept in 120 seq type abcde
0 outliers in 0 seq
CDF
# peptides in EIKLR
data
without outliers
2926632 pept in 120 seq type abcde
variance: exp/pred 81960000 / 24190 = 3388.9
filtered data
Gauss
Bernoulli
4779460 pept in 120 seq type abcde
0 outliers in 0 seq
# peptides in AEKLR
4779460 pept in 120 seq type abcde
variance: exp/pred 194400000 / 39500 = 4922.4
7975262 pept in 120 seq type abcde
133868 outliers in 1 seq
# peptides in AGLSV
7841394 pept in 119 seq type abcde
variance: exp/pred 90290000 / 65340 = 1381.9

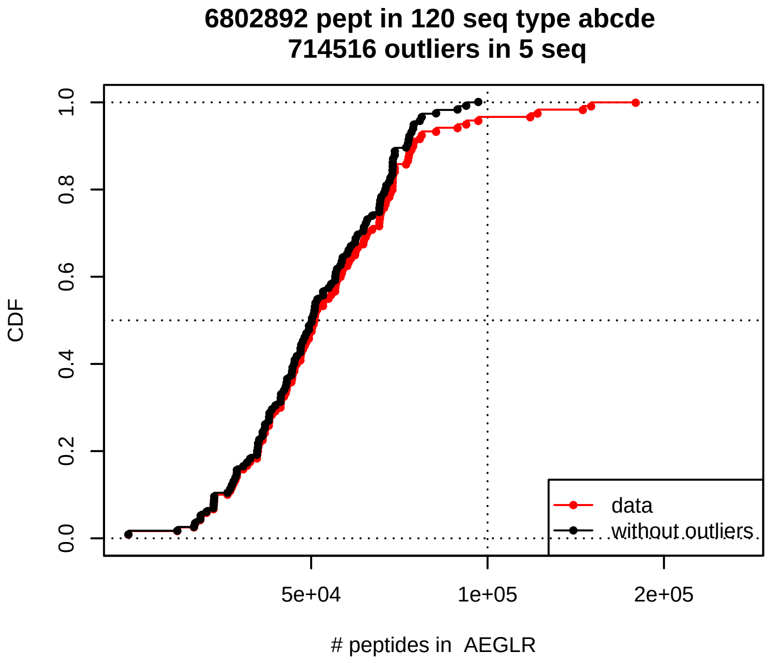
6802892 pept in 120 seq type abcde
714516 outliers in 5 seq
CDF
0.0
0.2
0.4
0.6
0.8
1.0
5e+04
1e+05
2e+05
# peptides in AEGLR
data
without outliers

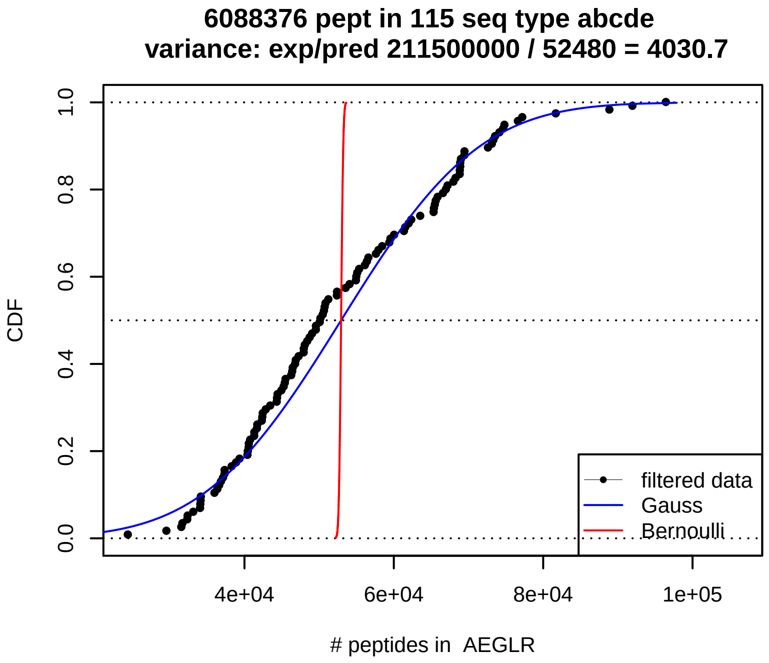
6088376 pept in 115 seq type abcde
variance: exp/pred 211500000 / 52480 = 4030.7
CDF
0.0
0.2
0.4
0.6
0.8
1.0
4e+04
6e+04
8e+04
1e+05
# peptides in AEGLR
filtered data
Gauss
Bernoulli

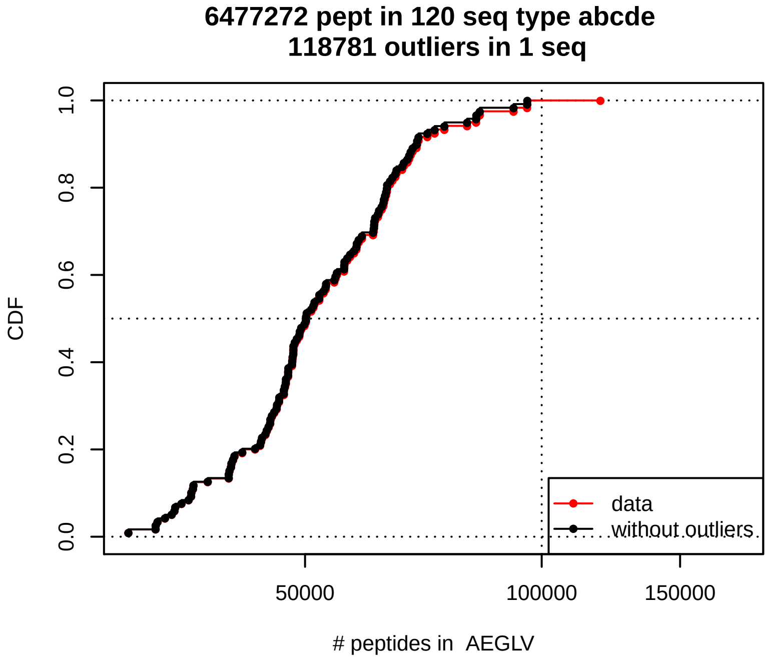
6477272 pept in 120 seq type abcde
118781 outliers in 1 seq
CDF
0.0
0.2
0.4
0.6
0.8
1.0
50000
100000
150000
# peptides in AEGLV
data
without outliers

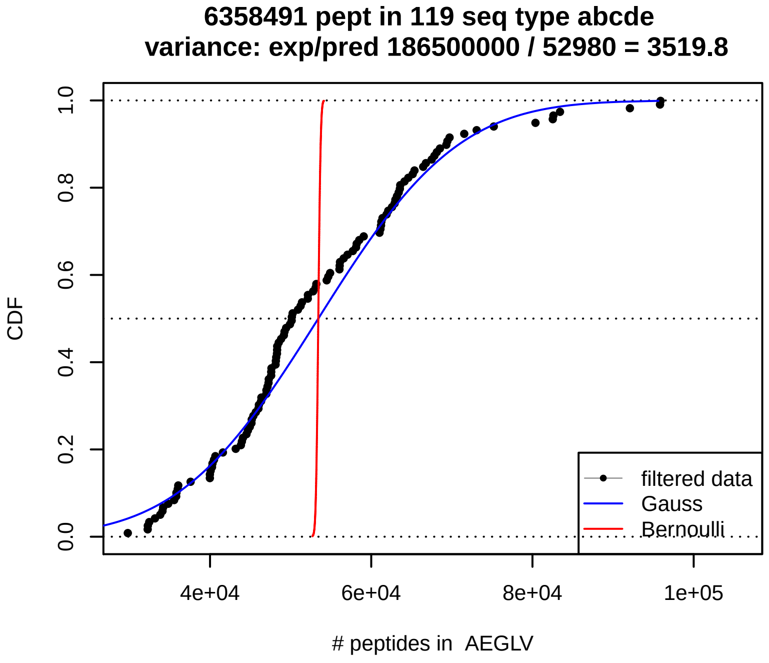
6358491 pept in 119 seq type abcde
variance: exp/pred 186500000 / 52980 = 3519.8
CDF
0.0
0.2
0.4
0.6
0.8
1.0
4e+04
6e+04
8e+04
1e+05
# peptides in AEGLV
filtered data
Gauss
Bernoulli

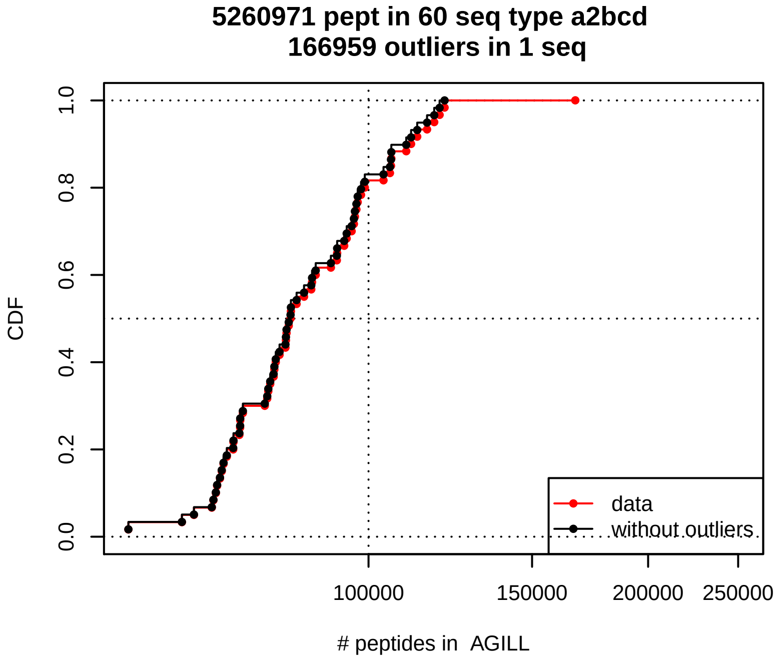
5260971 pept in 60 seq type a2bcd
166959 outliers in 1 seq
CDF
0.0
0.2
0.4
0.6
0.8
1.0
100000
150000
200000
250000
# peptides in AGILL
data
without outliers

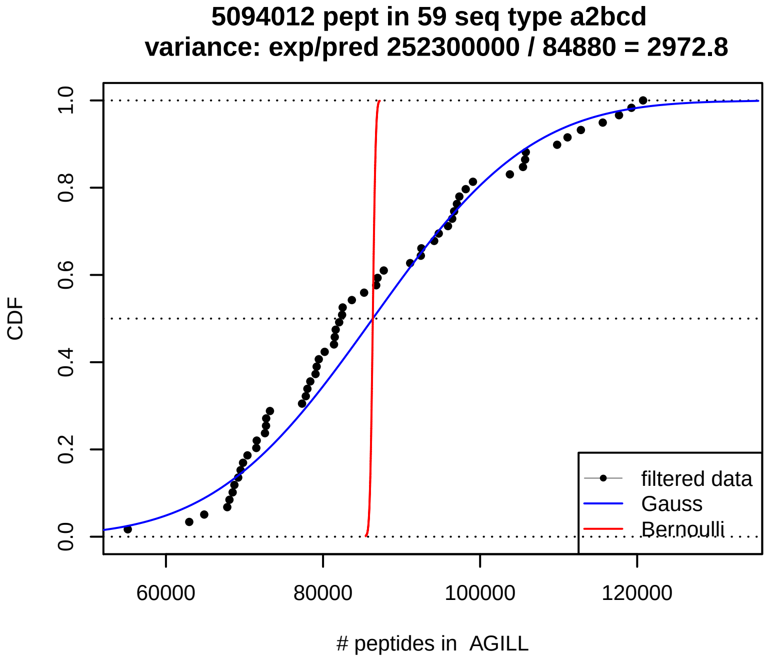
5094012 pept in 59 seq type a2bcd
variance: exp/pred 252300000 / 84880 = 2972.8
CDF
0.0
0.2
0.4
0.6
0.8
1.0
60000
80000
100000
120000
# peptides in AGILL
filtered data
Gauss
Bernoulli

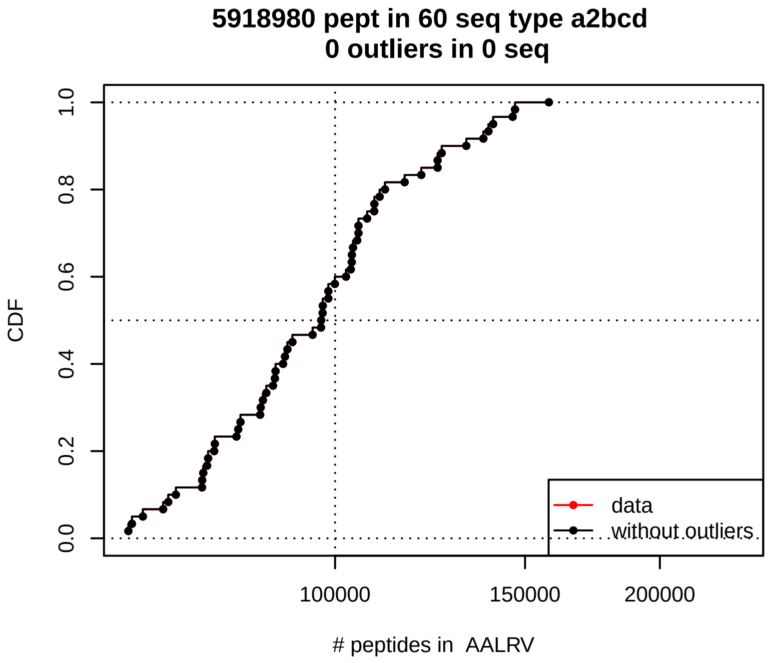
5918980 pept in 60 seq type a2bcd
0 outliers in 0 seq
CDF
0.0 0.2 0.4 0.6 0.8 1.0
100000 150000 200000
# peptides in AALRV
data
without outliers

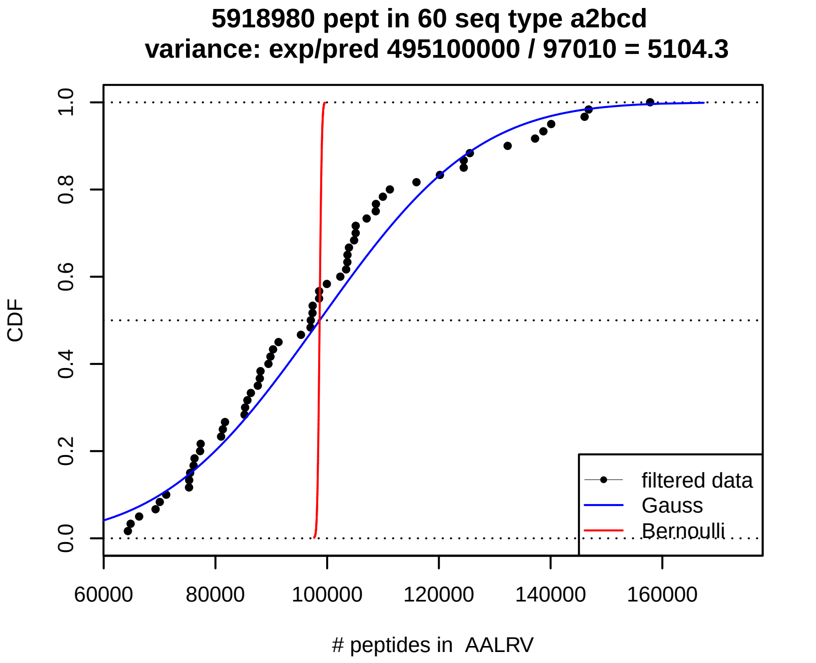
5918980 pept in 60 seq type a2bcd
variance: exp/pred 495100000 / 97010 = 5104.3
CDF
0.0 0.2 0.4 0.6 0.8 1.0
60000 80000 100000 120000 140000 160000
# peptides in AALRV
filtered data
Gauss
Bernoulli

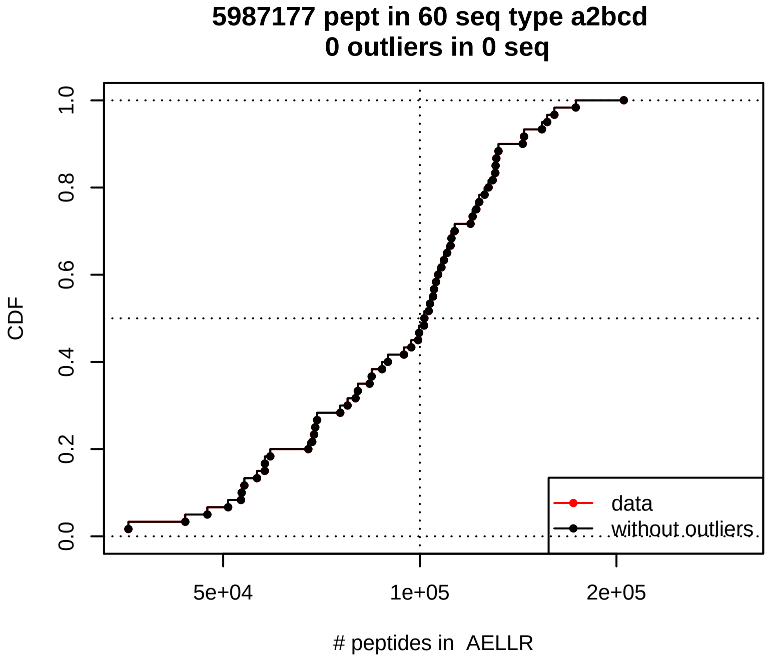
5987177 pept in 60 seq type a2bcd
0 outliers in 0 seq
CDF
0.0 0.2 0.4 0.6 0.8 1.0
5e+04 1e+05 2e+05
# peptides in AELLR
data
without outliers

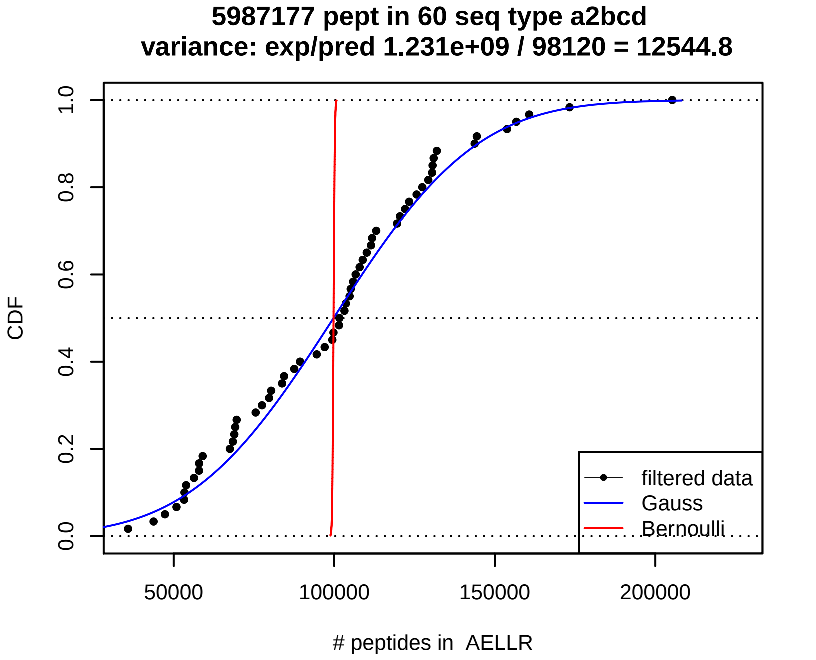
5987177 pept in 60 seq type a2bcd
variance: exp/pred 1.231e+09 / 98120 = 12544.8
CDF
0.0 0.2 0.4 0.6 0.8 1.0
50000 100000 150000 200000
# peptides in AELLR
filtered data
Gauss
Bernoulli

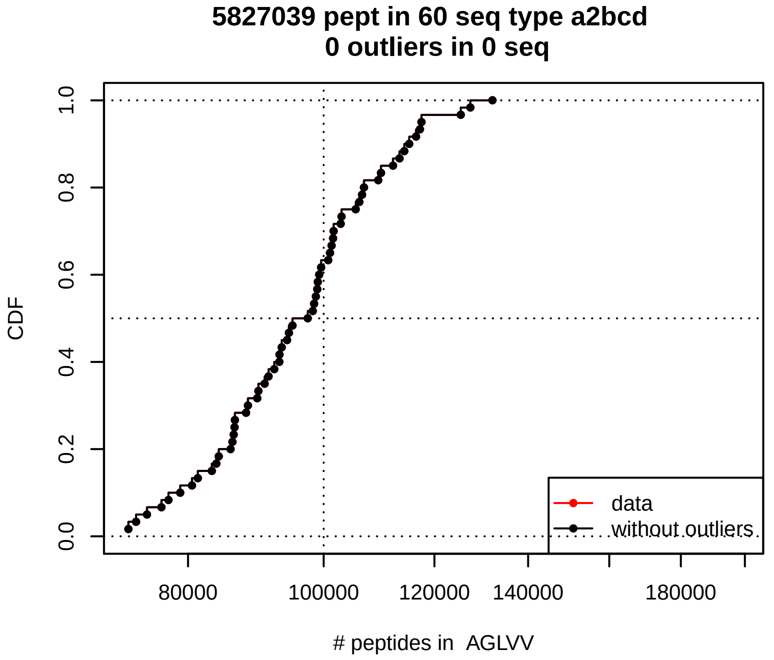
5827039 pept in 60 seq type a2bcd
0 outliers in 0 seq
CDF
0.0 0.2 0.4 0.6 0.8 1.0
80000 100000 120000 140000 180000
# peptides in AGLVV
data
without outliers

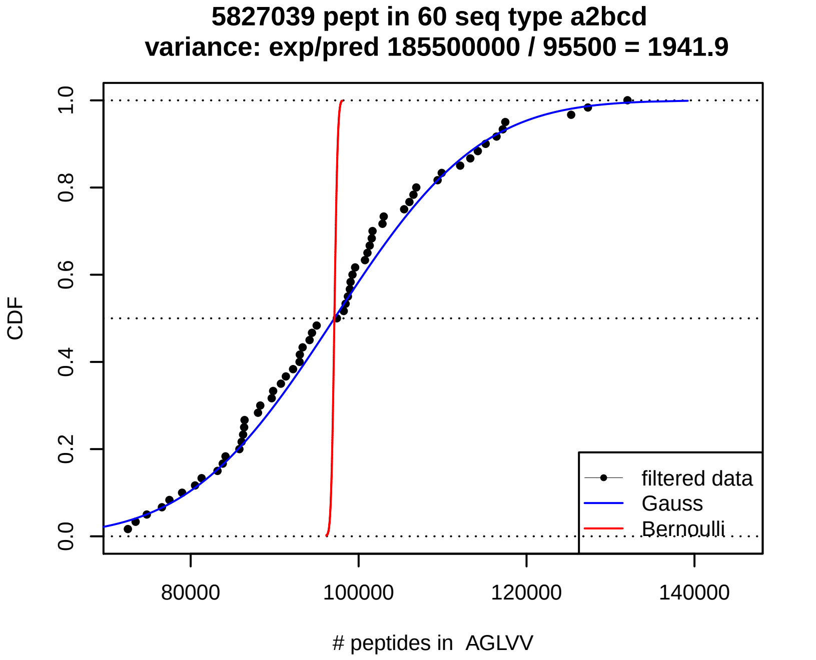
5827039 pept in 60 seq type a2bcd
variance: exp/pred 185500000 / 95500 = 1941.9
CDF
0.0 0.2 0.4 0.6 0.8 1.0
80000 100000 120000 140000
# peptides in AGLVV
filtered data
Gauss
Bernoulli

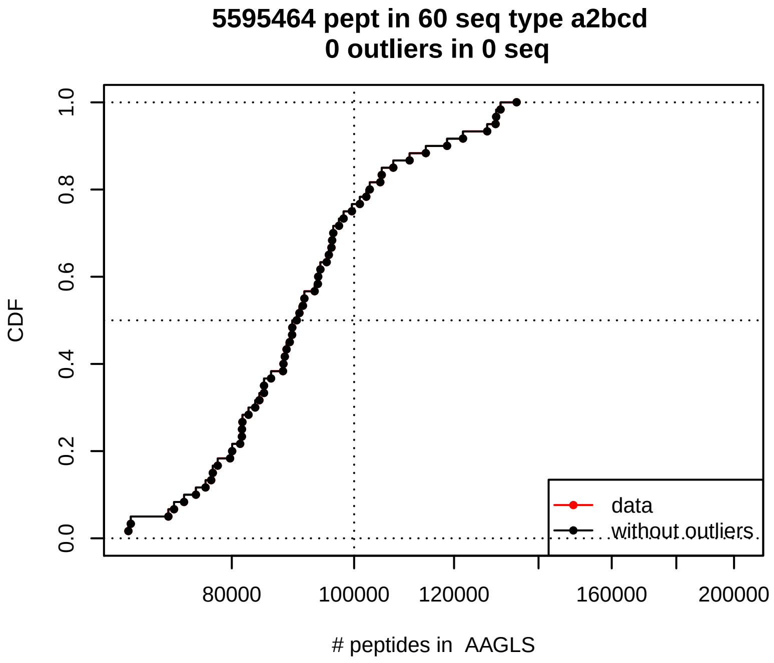
5595464 pept in 60 seq type a2bcd
0 outliers in 0 seq
CDF
0.0
0.2
0.4
0.6
0.8
1.0
80000
100000
120000
160000
200000
data
without outliers
# peptides in AAGLS

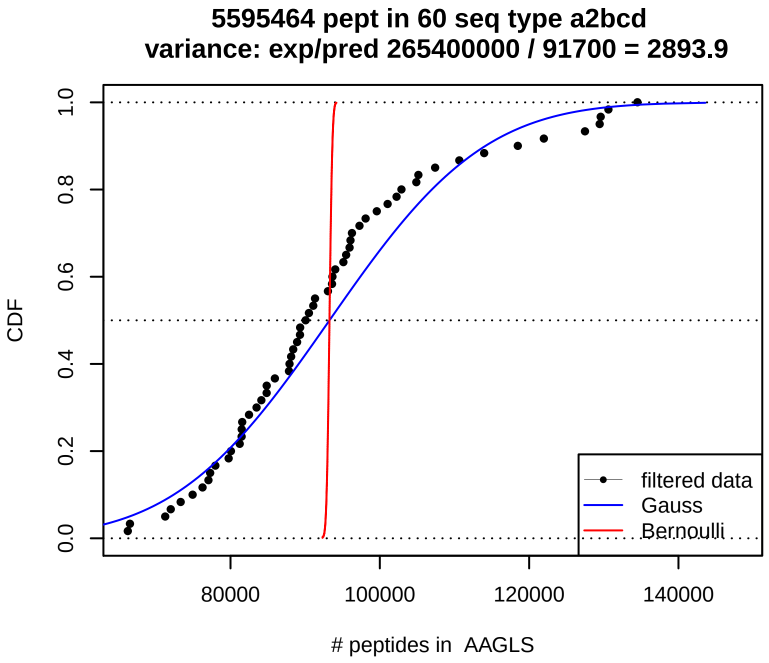
5595464 pept in 60 seq type a2bcd
variance: exp/pred 265400000 / 91700 = 2893.9
CDF
0.0
0.2
0.4
0.6
0.8
1.0
80000
100000
120000
140000
filtered data
Gauss
Bernoulli
# peptides in AAGLS

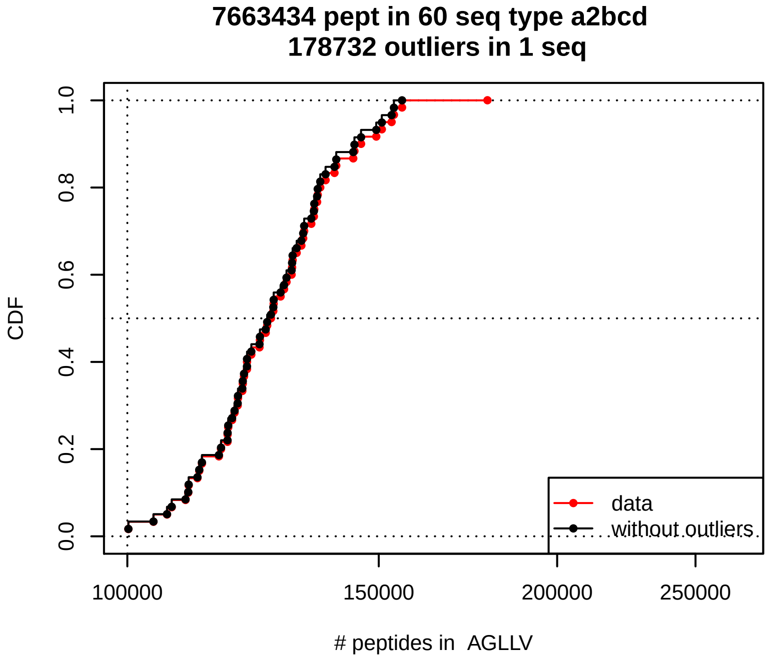
7663434 pept in 60 seq type a2bcd
178732 outliers in 1 seq
CDF
0.0
0.2
0.4
0.6
0.8
1.0
100000
150000
200000
250000
data
without outliers
# peptides in AGLLV

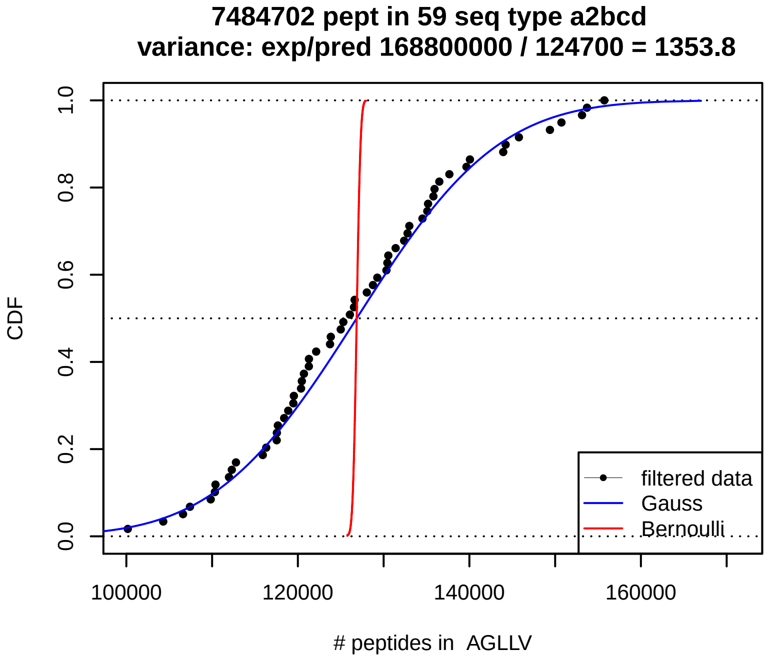
7484702 pept in 59 seq type a2bcd
variance: exp/pred 168800000 / 124700 = 1353.8
CDF
0.0
0.2
0.4
0.6
0.8
1.0
100000
120000
140000
160000
filtered data
Gauss
Bernoulli
# peptides in AGLLV

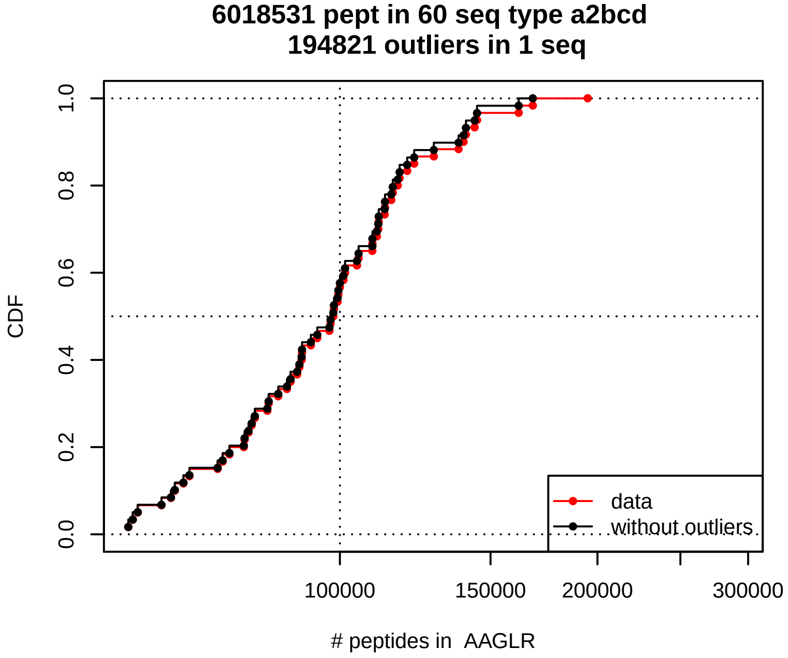
6018531 pept in 60 seq type a2bcd
194821 outliers in 1 seq
CDF
0.0
0.2
0.4
0.6
0.8
1.0
100000
150000
200000
300000
data
without outliers
# peptides in AAGLR

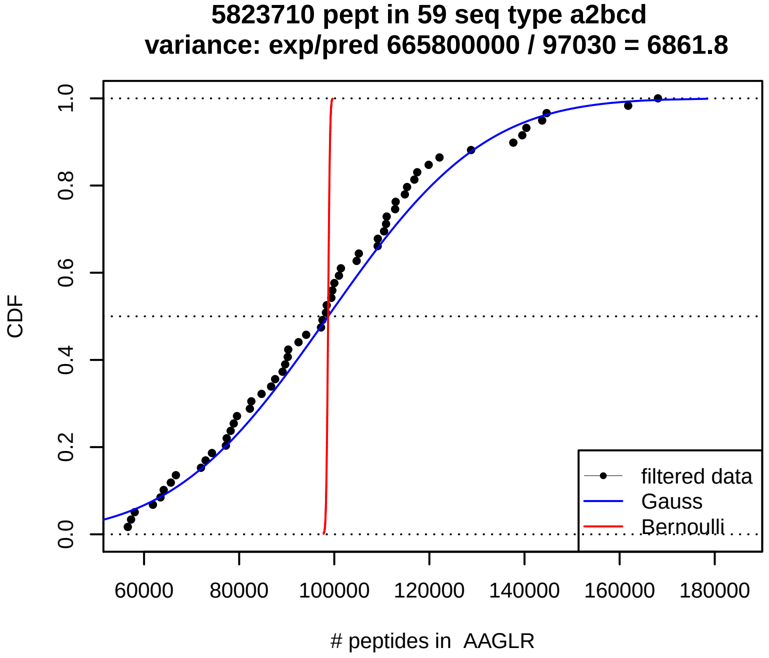
5823710 pept in 59 seq type a2bcd
variance: exp/pred 665800000 / 97030 = 6861.8
CDF
0.0
0.2
0.4
0.6
0.8
1.0
60000
80000
100000
120000
140000
160000
180000
filtered data
Gauss
Bernoulli
# peptides in AAGLR

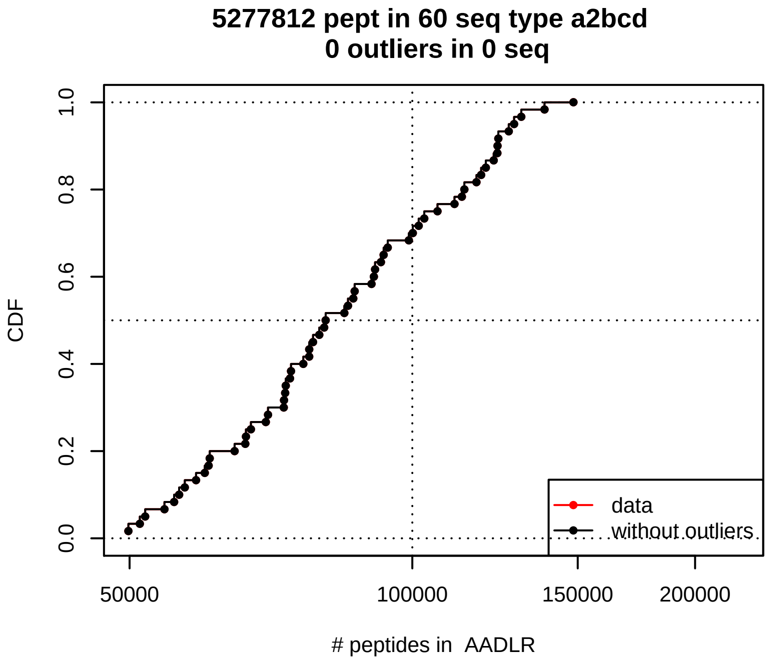
5277812 pept in 60 seq type a2bcd
0 outliers in 0 seq
CDF
# peptides in AADLR
data
without outliers

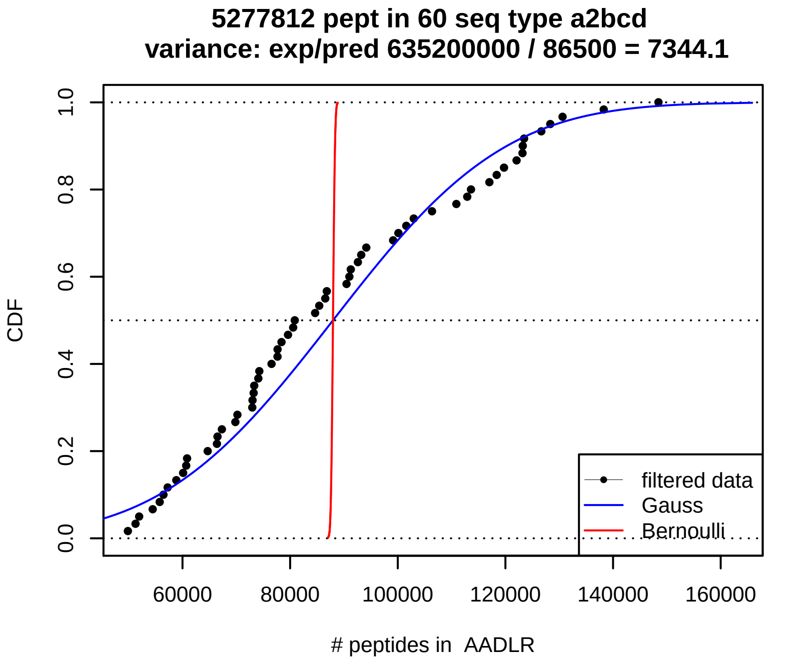
5277812 pept in 60 seq type a2bcd
variance: exp/pred 635200000 / 86500 = 7344.1
CDF
# peptides in AADLR
filtered data
Gauss
Bernoulli

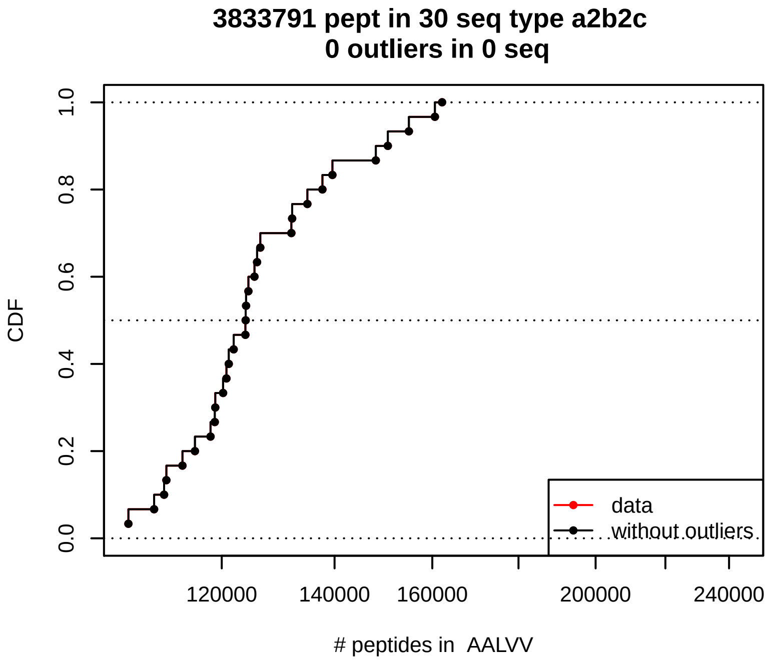
3833791 pept in 30 seq type a2b2c
0 outliers in 0 seq
CDF
# peptides in AALVV
data
without outliers

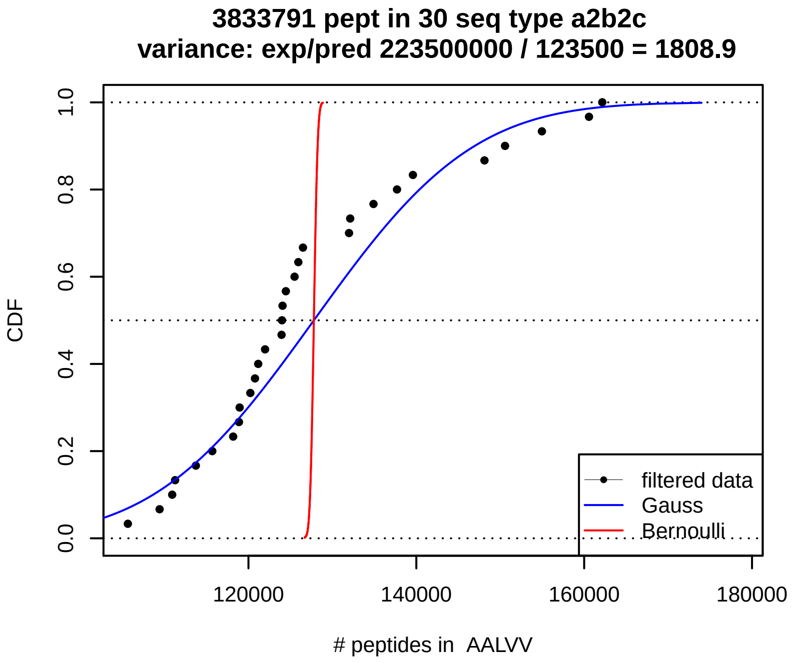
3833791 pept in 30 seq type a2b2c
variance: exp/pred 223500000 / 123500 = 1808.9
CDF
# peptides in AALVV
filtered data
Gauss
Bernoulli

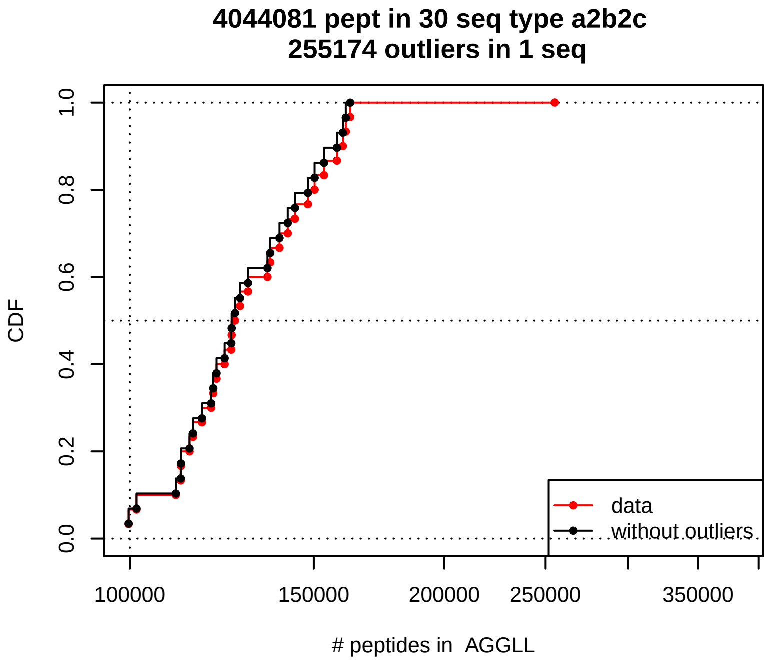
4044081 pept in 30 seq type a2b2c
255174 outliers in 1 seq
CDF
# peptides in AGGLL
data
without outliers

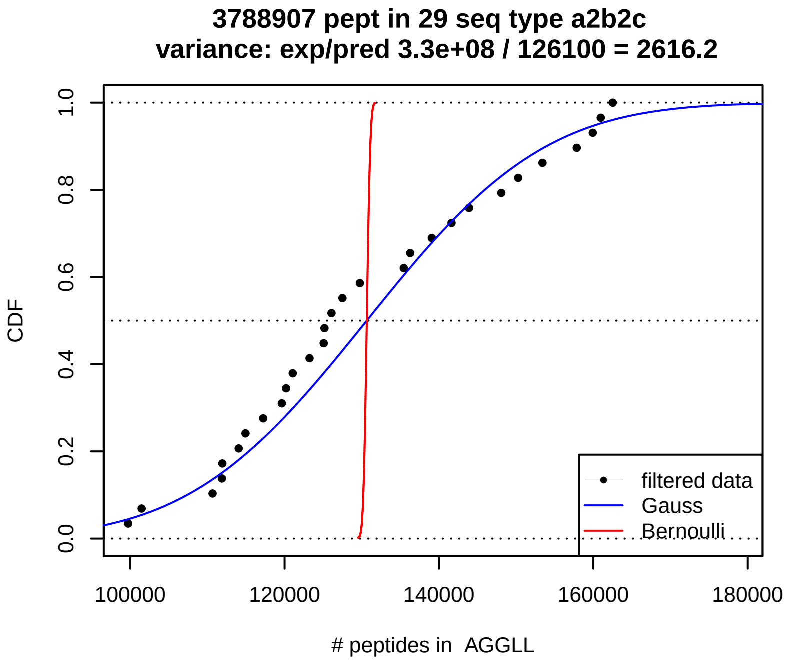
3788907 pept in 29 seq type a2b2c
variance: exp/pred 3.3e+08 / 126100 = 2616.2
CDF
# peptides in AGGLL
filtered data
Gauss
Bernoulli

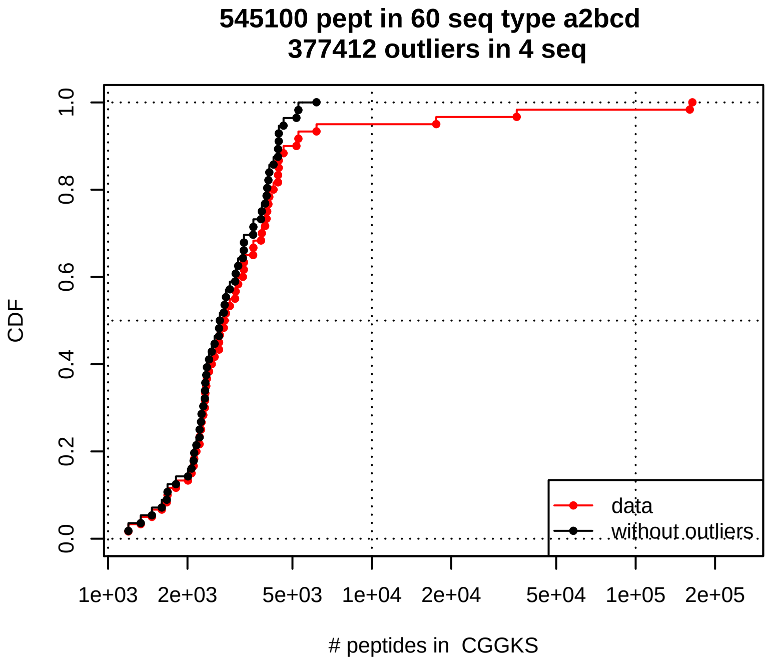
545100 pept in 60 seq type a2bcd
377412 outliers in 4 seq
CDF
0.0
0.2
0.4
0.6
0.8
1.0
1e+03
2e+03
5e+03
1e+04
2e+04
5e+04
1e+05
2e+05
# peptides in CGGKS
data
without outliers

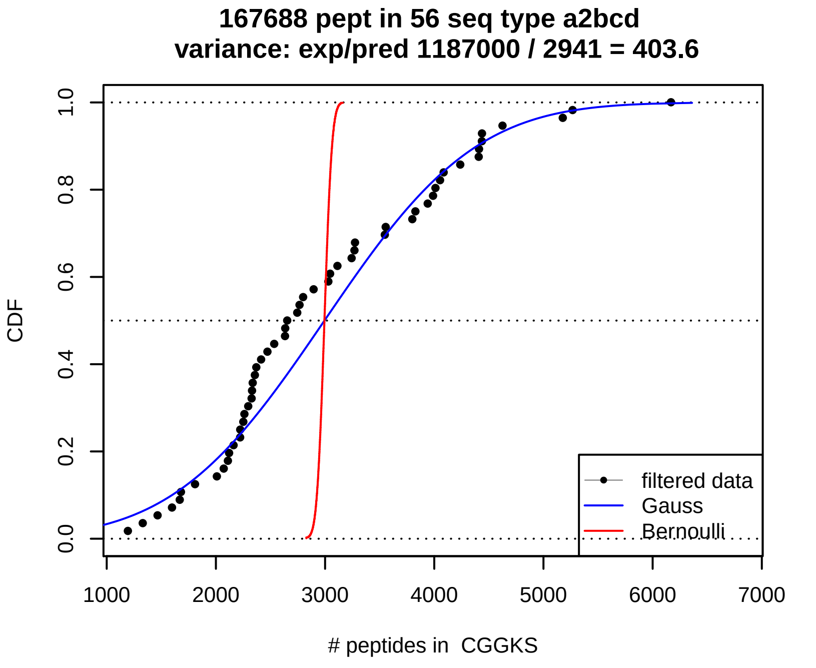
167688 pept in 56 seq type a2bcd
variance: exp/pred 1187000 / 2941 = 403.6
CDF
0.0
0.2
0.4
0.6
0.8
1.0
1000
2000
3000
4000
5000
6000
7000
# peptides in CGGKS
filtered data
Gauss
Bernoulli

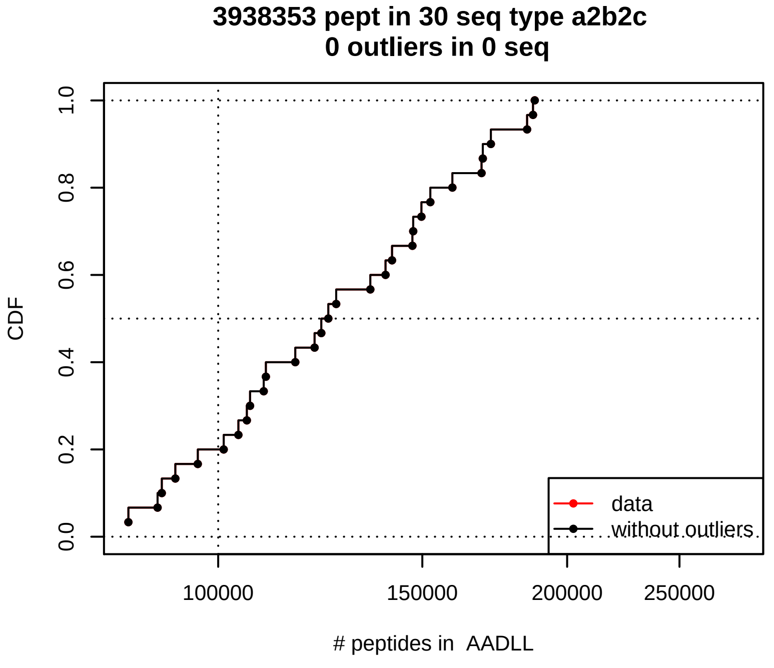
3938353 pept in 30 seq type a2b2c
0 outliers in 0 seq
CDF
0.0
0.2
0.4
0.6
0.8
1.0
100000
150000
200000
250000
# peptides in AADLL
data
without outliers

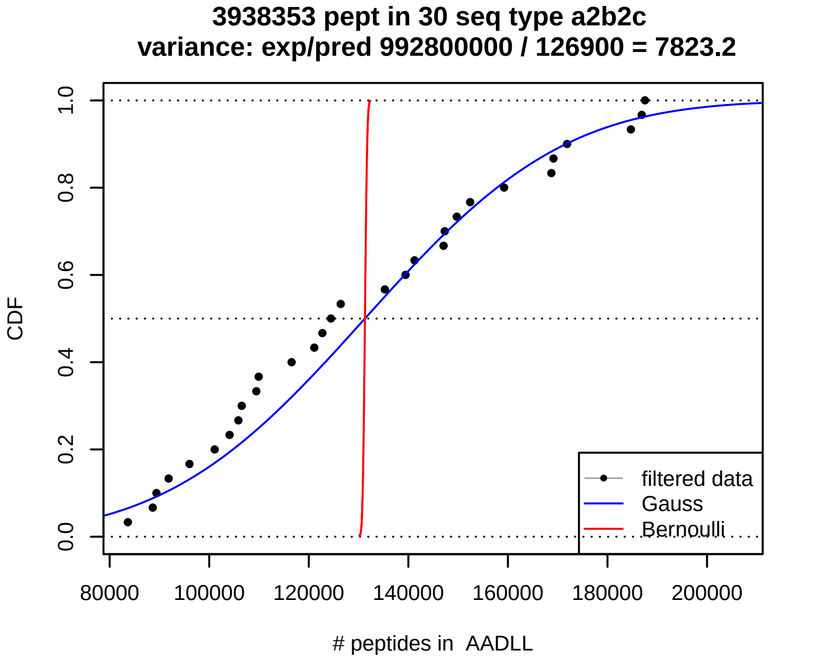
3938353 pept in 30 seq type a2b2c
variance: exp/pred 992800000 / 126900 = 7823.2
CDF
0.0
0.2
0.4
0.6
0.8
1.0
80000
100000
120000
140000
160000
180000
200000
# peptides in AADLL
filtered data
Gauss
Bernoulli

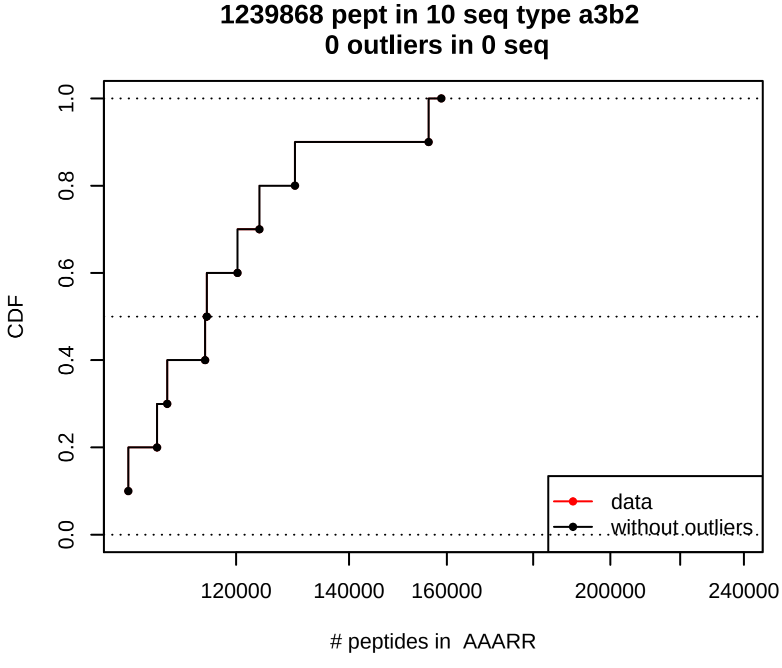
1239868 pept in 10 seq type a3b2
0 outliers in 0 seq
CDF
0.0
0.2
0.4
0.6
0.8
1.0
120000
140000
160000
200000
240000
# peptides in AAARR
data
without outliers

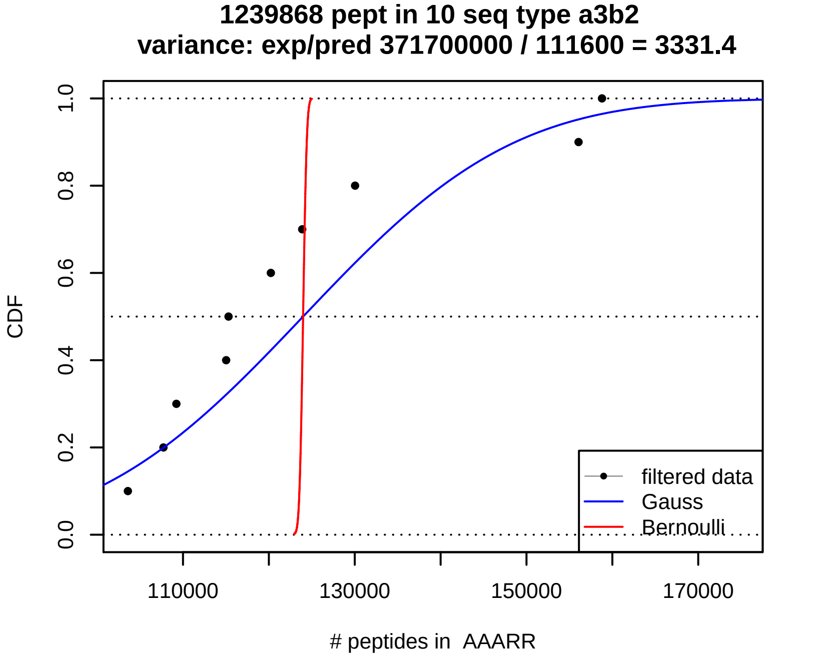
1239868 pept in 10 seq type a3b2
variance: exp/pred 371700000 / 111600 = 3331.4
CDF
0.0
0.2
0.4
0.6
0.8
1.0
110000
130000
150000
170000
# peptides in AAARR
filtered data
Gauss
Bernoulli

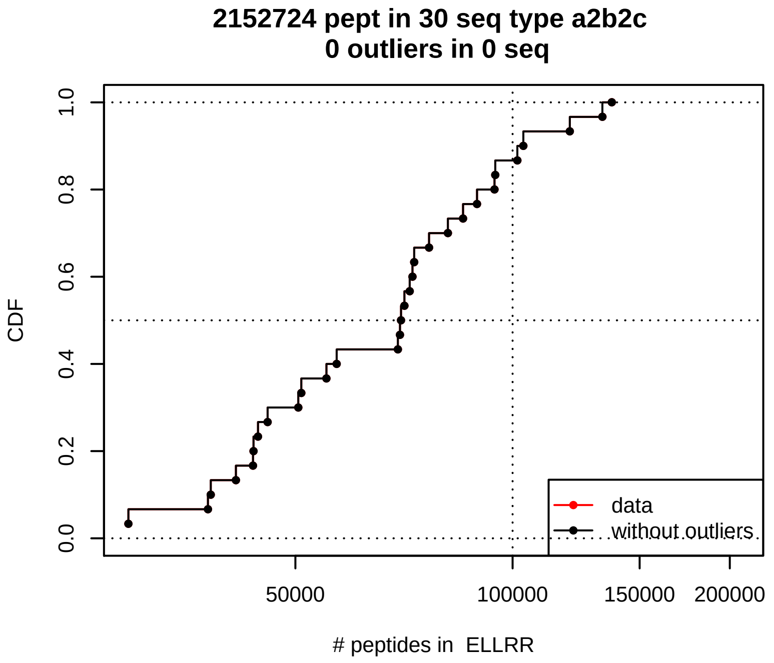
2152724 pept in 30 seq type a2b2c
0 outliers in 0 seq
CDF
0.0
0.2
0.4
0.6
0.8
1.0
50000
100000
150000
200000
# peptides in ELLRR
data
without outliers

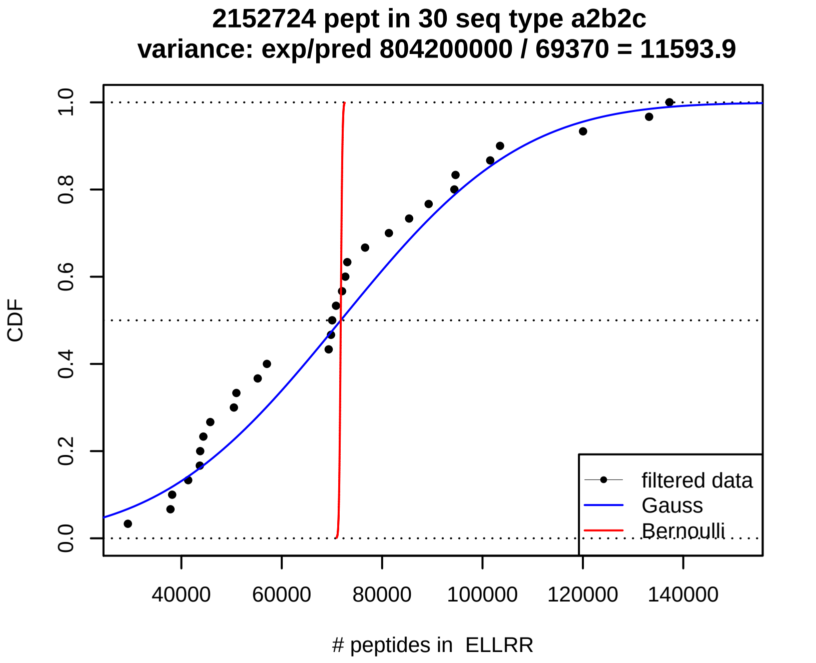
2152724 pept in 30 seq type a2b2c
variance: exp/pred 804200000 / 69370 = 11593.9
CDF
0.0
0.2
0.4
0.6
0.8
1.0
40000
60000
80000
100000
120000
140000
# peptides in ELLRR
filtered data
Gauss
Bernoulli

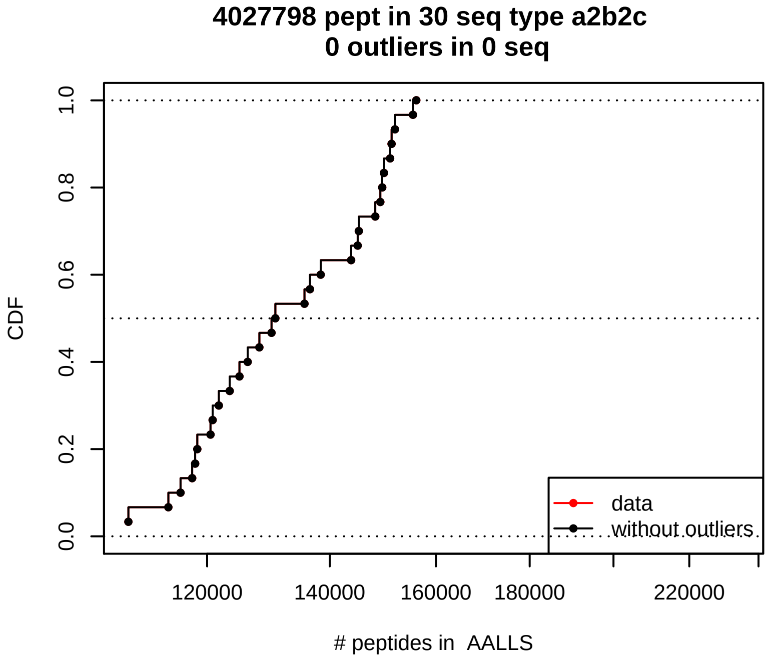
4027798 pept in 30 seq type a2b2c
0 outliers in 0 seq
CDF
0.0
0.2
0.4
0.6
0.8
1.0
120000
140000
160000
180000
220000
# peptides in AALLS
data
without outliers

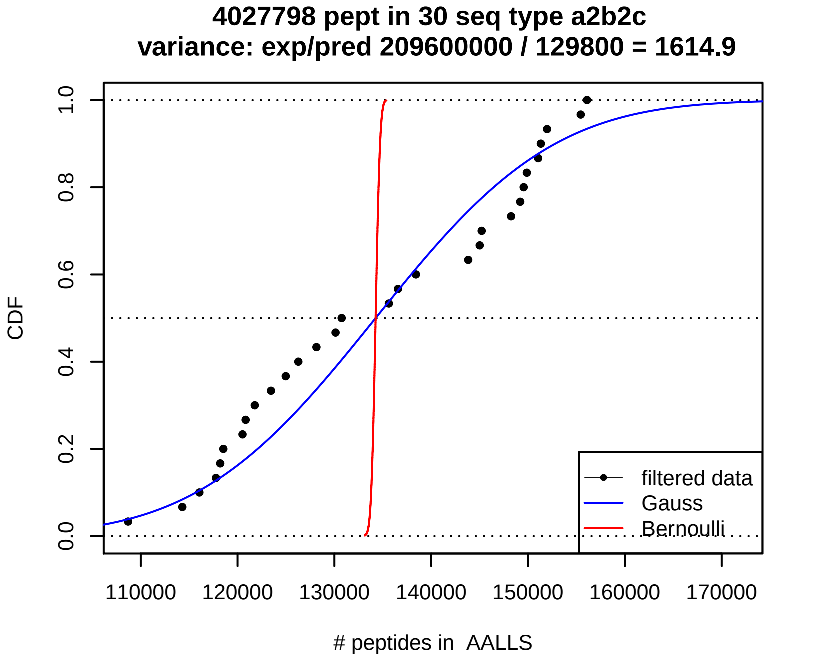
4027798 pept in 30 seq type a2b2c
variance: exp/pred 209600000 / 129800 = 1614.9
CDF
0.0
0.2
0.4
0.6
0.8
1.0
110000
120000
130000
140000
150000
160000
170000
# peptides in AALLS
filtered data
Gauss
Bernoulli

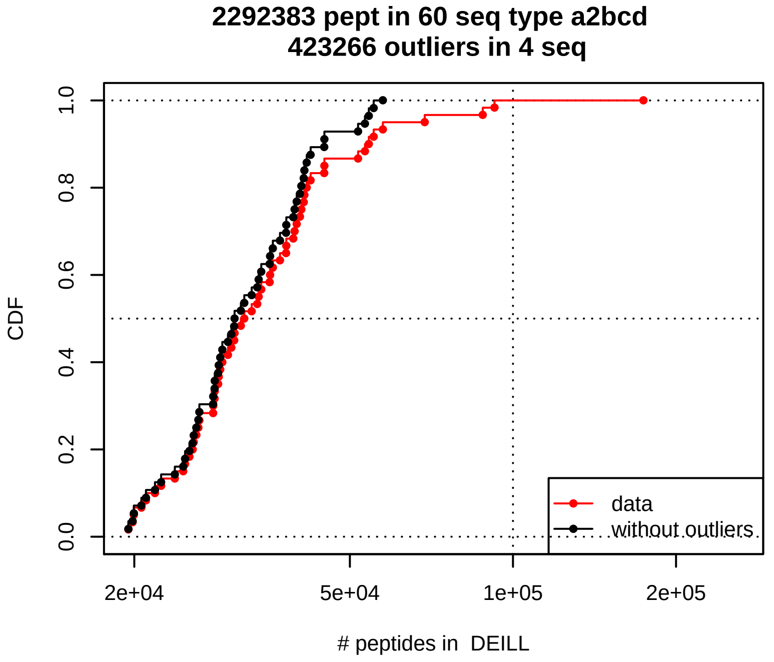
2292383 pept in 60 seq type a2bcd
423266 outliers in 4 seq
CDF
0.0
0.2
0.4
0.6
0.8
1.0
2e+04
5e+04
1e+05
2e+05
# peptides in DEILL
data
without outliers

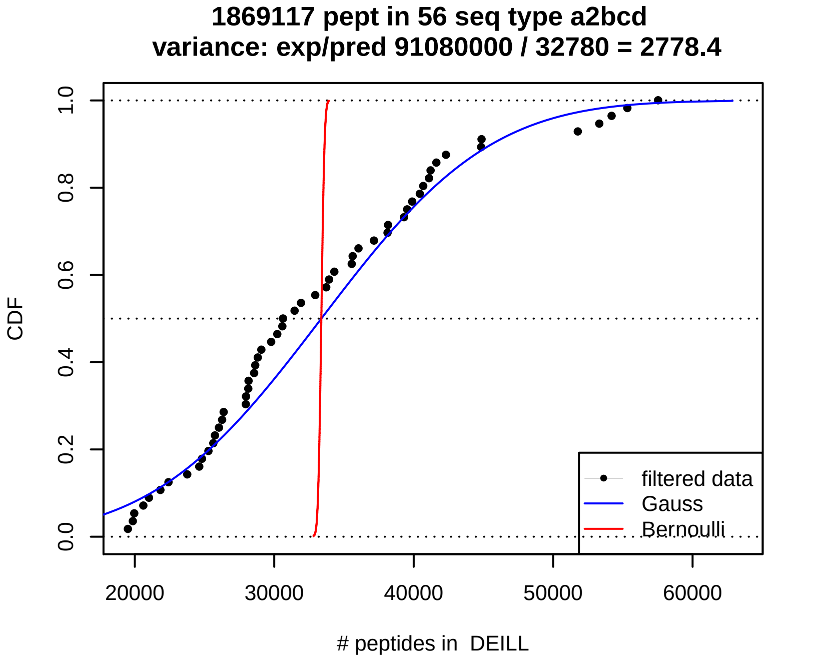
1869117 pept in 56 seq type a2bcd
variance: exp/pred 91080000 / 32780 = 2778.4
CDF
0.0
0.2
0.4
0.6
0.8
1.0
20000
30000
40000
50000
60000
# peptides in DEILL
filtered data
Gauss
Bernoulli

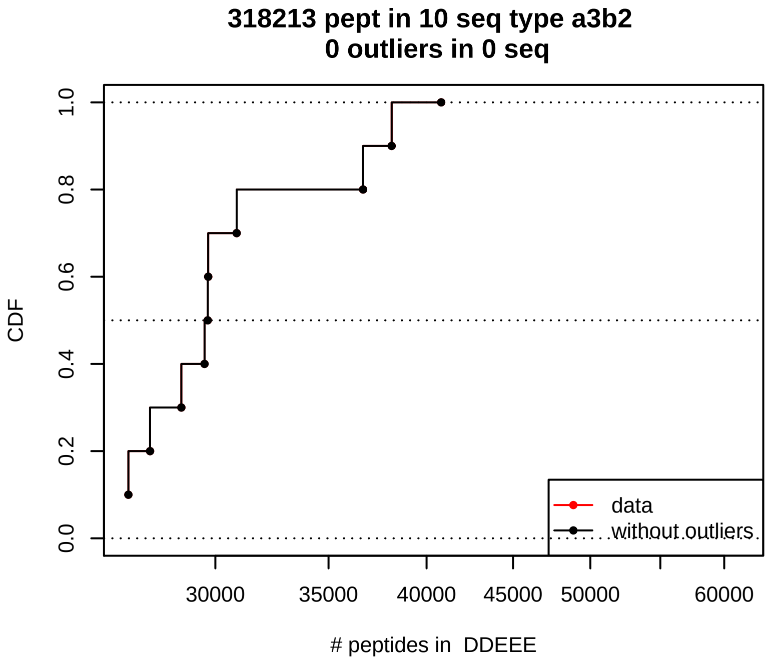
318213 pept in 10 seq type a3b2
0 outliers in 0 seq
CDF
# peptides in DDEEE
data
without outliers

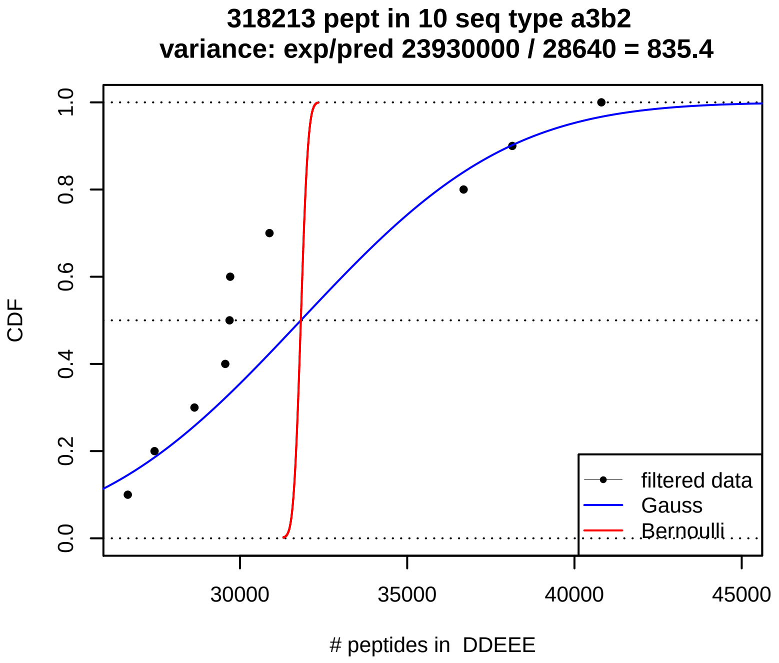
318213 pept in 10 seq type a3b2
variance: exp/pred 23930000 / 28640 = 835.4
CDF
# peptides in DDEEE
filtered data
Gauss
Bernoulli

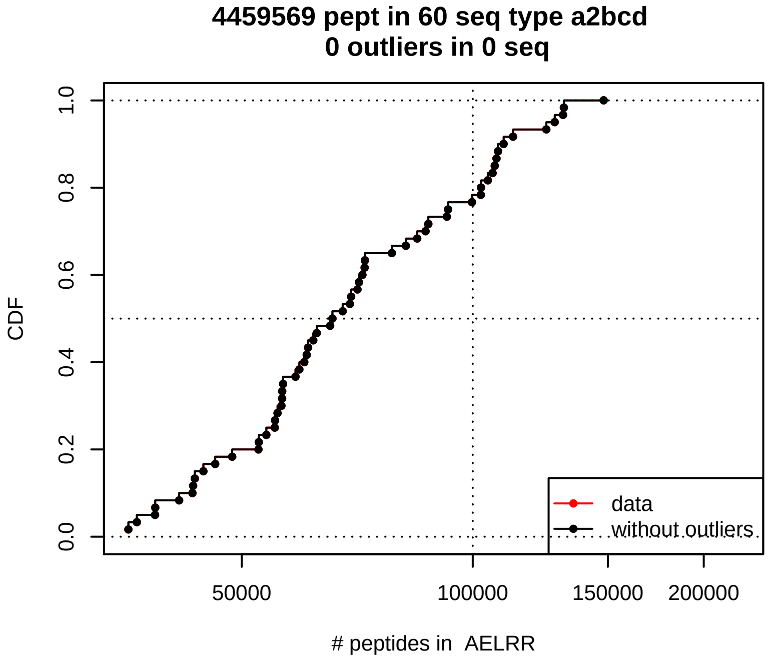
4459569 pept in 60 seq type a2bcd
0 outliers in 0 seq
CDF
# peptides in AELRR
data
without outliers

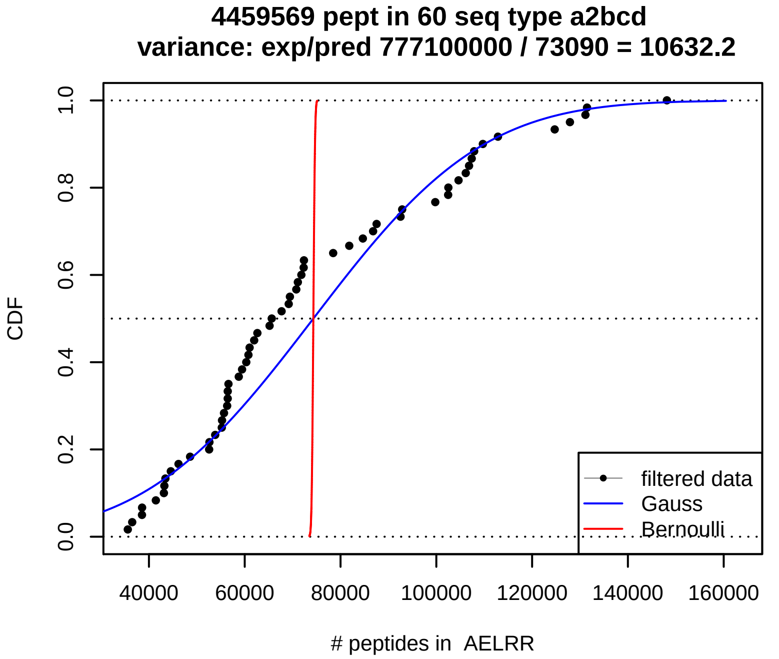
4459569 pept in 60 seq type a2bcd
variance: exp/pred 777100000 / 73090 = 10632.2
CDF
# peptides in AELRR
filtered data
Gauss
Bernoulli

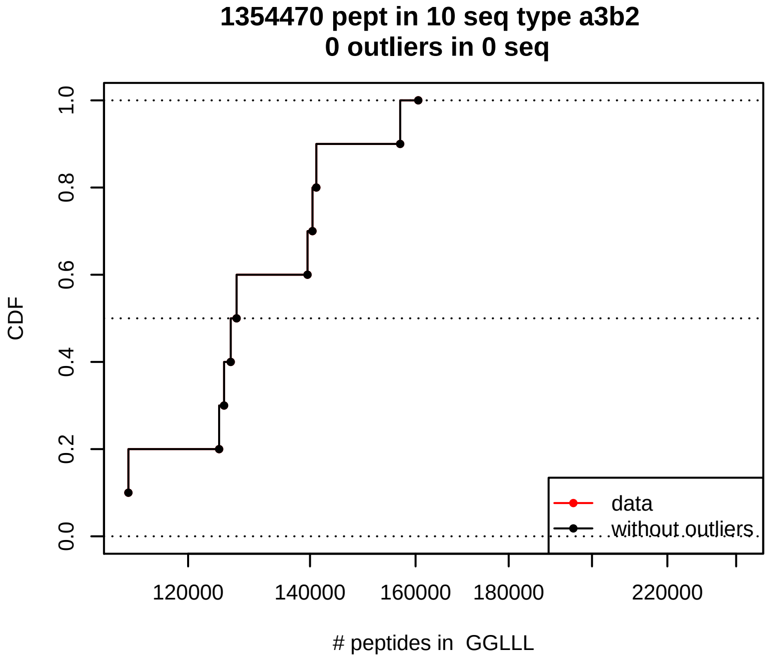
1354470 pept in 10 seq type a3b2
0 outliers in 0 seq
CDF
# peptides in GGLLL
data
without outliers

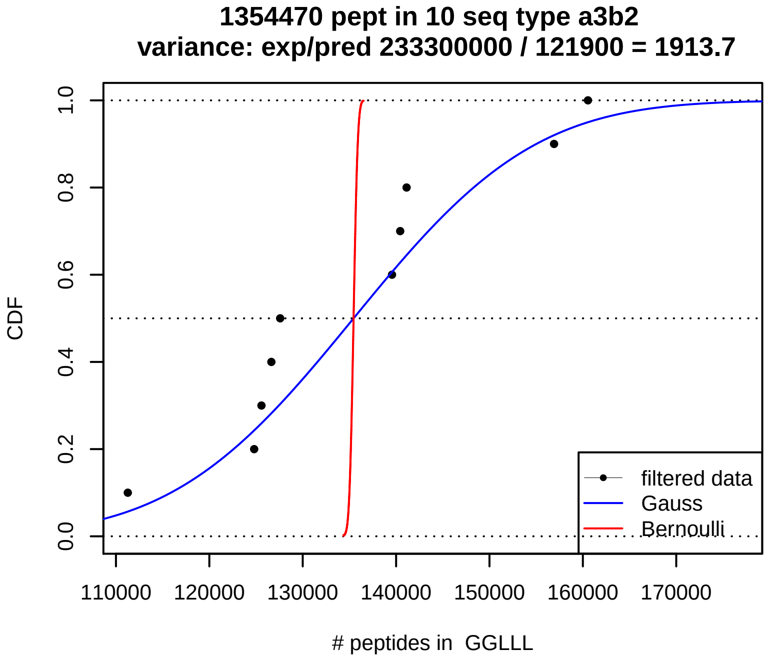
1354470 pept in 10 seq type a3b2
variance: exp/pred 233300000 / 121900 = 1913.7
CDF
# peptides in GGLLL
filtered data
Gauss
Bernoulli

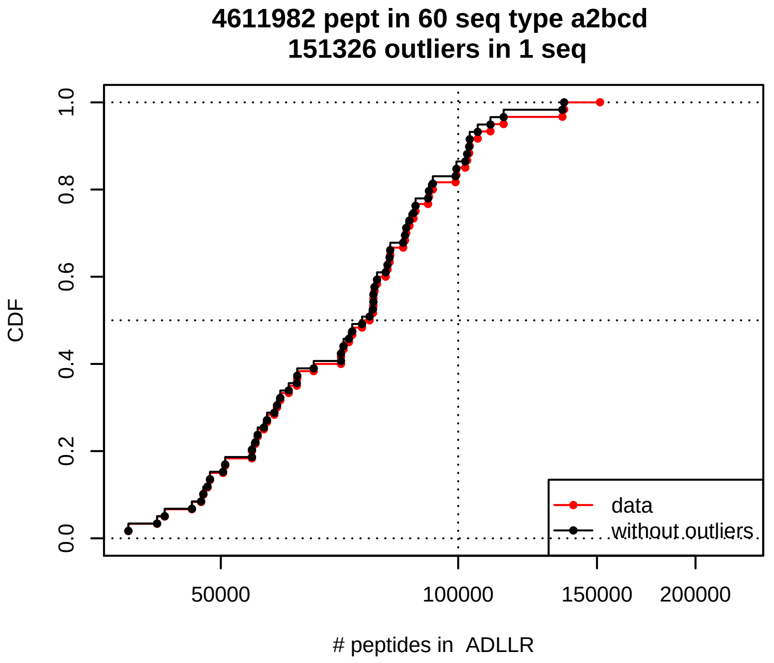
4611982 pept in 60 seq type a2bcd
151326 outliers in 1 seq
CDF
0.0
0.2
0.4
0.6
0.8
1.0
50000
100000
150000
200000
# peptides in ADLLR
data
without outliers

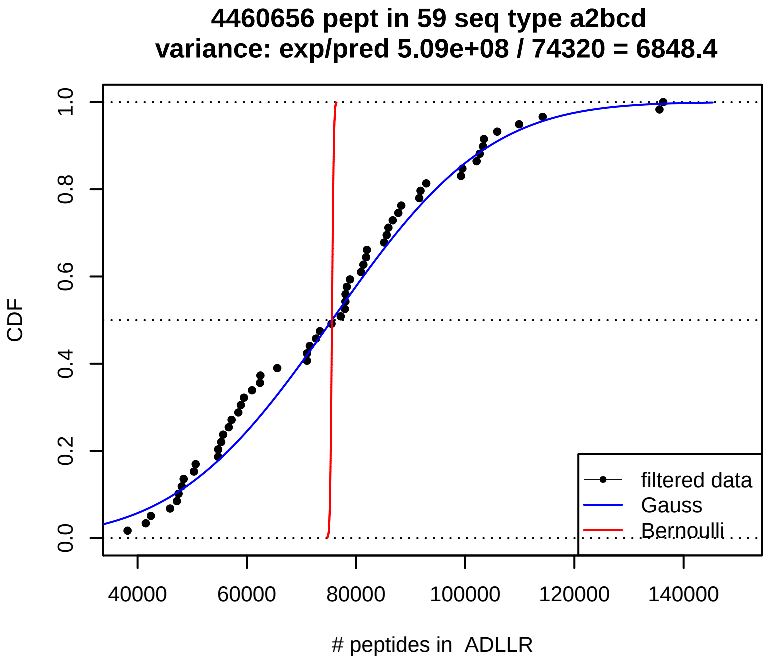
4460656 pept in 59 seq type a2bcd
variance: exp/pred 5.09e+08 / 74320 = 6848.4
CDF
0.0
0.2
0.4
0.6
0.8
1.0
40000
60000
80000
100000
120000
140000
# peptides in ADLLR
filtered data
Gauss
Bernoulli

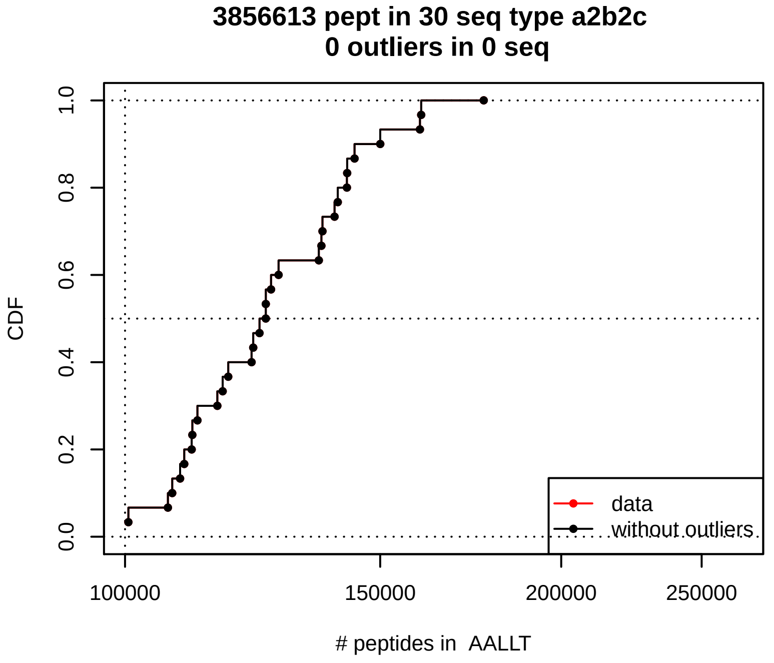
3856613 pept in 30 seq type a2b2c
0 outliers in 0 seq
CDF
0.0
0.2
0.4
0.6
0.8
1.0
100000
150000
200000
250000
# peptides in AALLT
data
without outliers

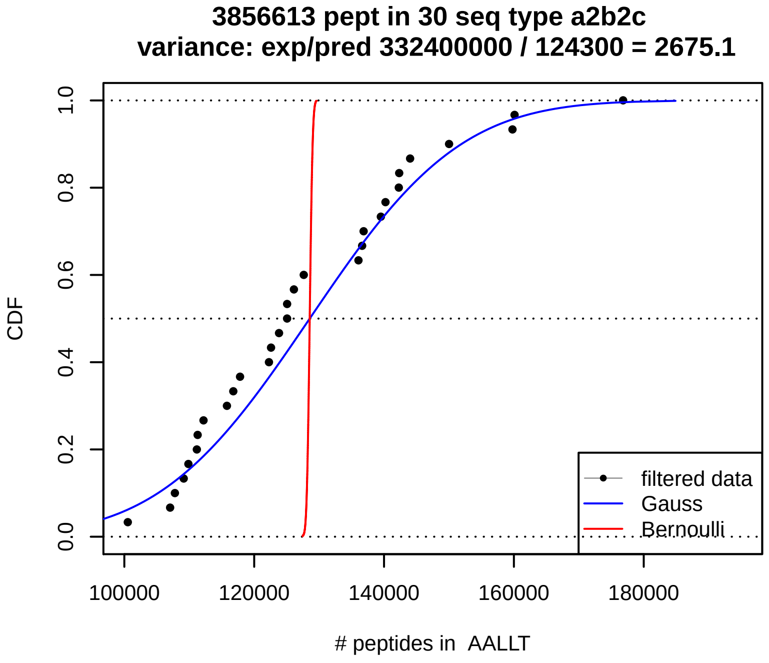
3856613 pept in 30 seq type a2b2c
variance: exp/pred 332400000 / 124300 = 2675.1
CDF
0.0
0.2
0.4
0.6
0.8
1.0
100000
120000
140000
160000
180000
# peptides in AALLT
filtered data
Gauss
Bernoulli

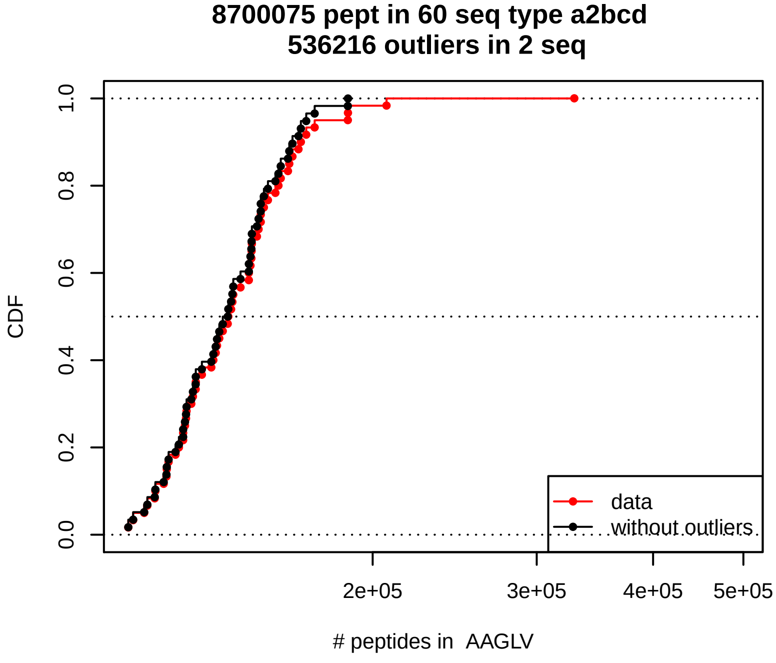
8700075 pept in 60 seq type a2bcd
536216 outliers in 2 seq
CDF
0.0
0.2
0.4
0.6
0.8
1.0
2e+05
3e+05
4e+05
5e+05
# peptides in AAGLV
data
without outliers

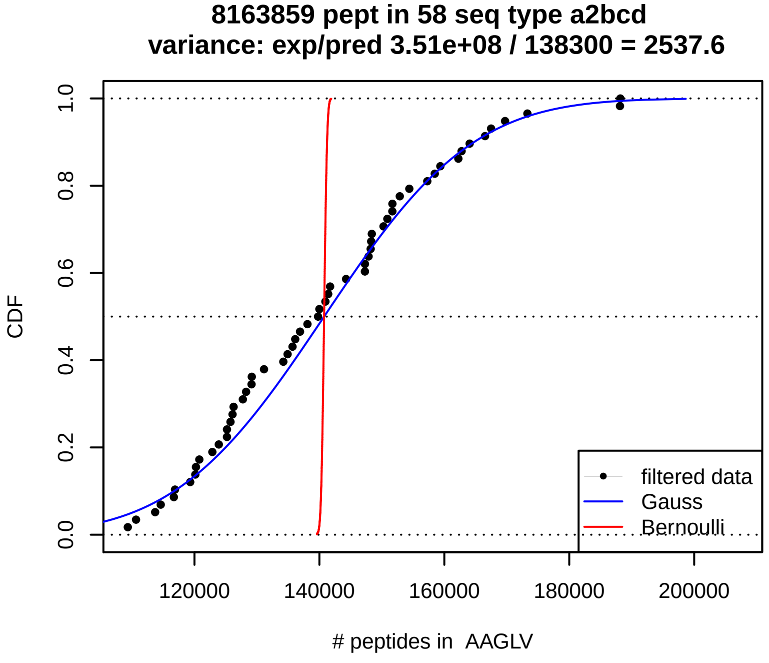
8163859 pept in 58 seq type a2bcd
variance: exp/pred 3.51e+08 / 138300 = 2537.6
CDF
0.0
0.2
0.4
0.6
0.8
1.0
120000
140000
160000
180000
200000
# peptides in AAGLV
filtered data
Gauss
Bernoulli

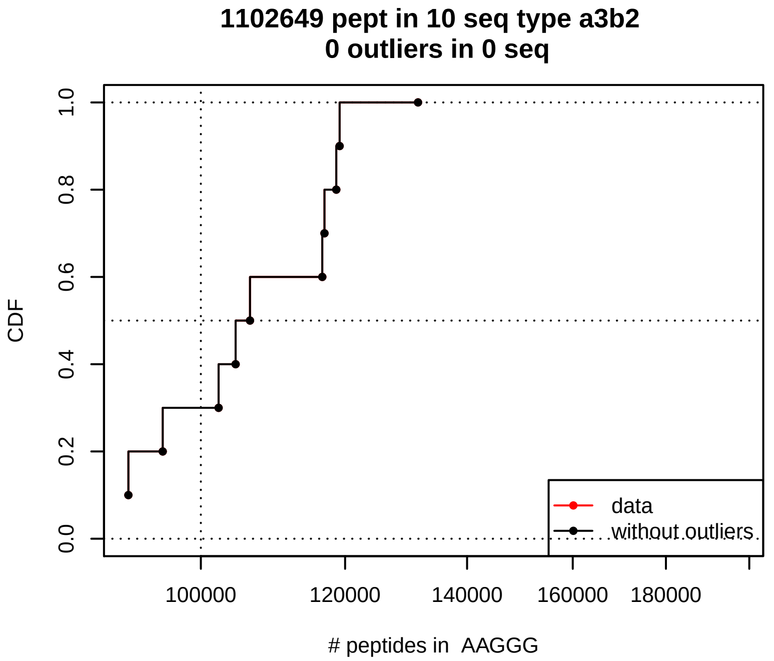
1102649 pept in 10 seq type a3b2
0 outliers in 0 seq
CDF
# peptides in AAGGG
data
without outliers

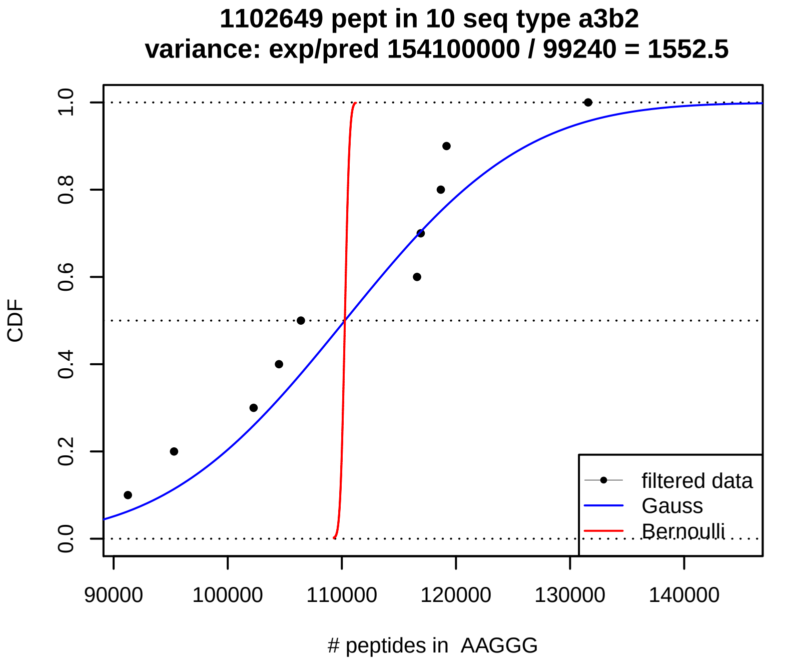
1102649 pept in 10 seq type a3b2
variance: exp/pred 154100000 / 99240 = 1552.5
CDF
# peptides in AAGGG
filtered data
Gauss
Bernoulli

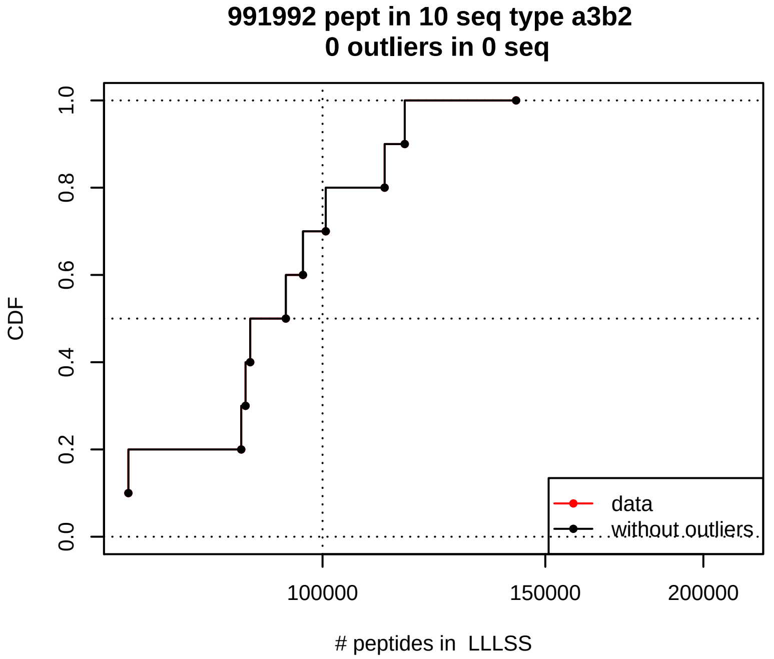
991992 pept in 10 seq type a3b2
0 outliers in 0 seq
CDF
# peptides in LLLSS
data
without outliers

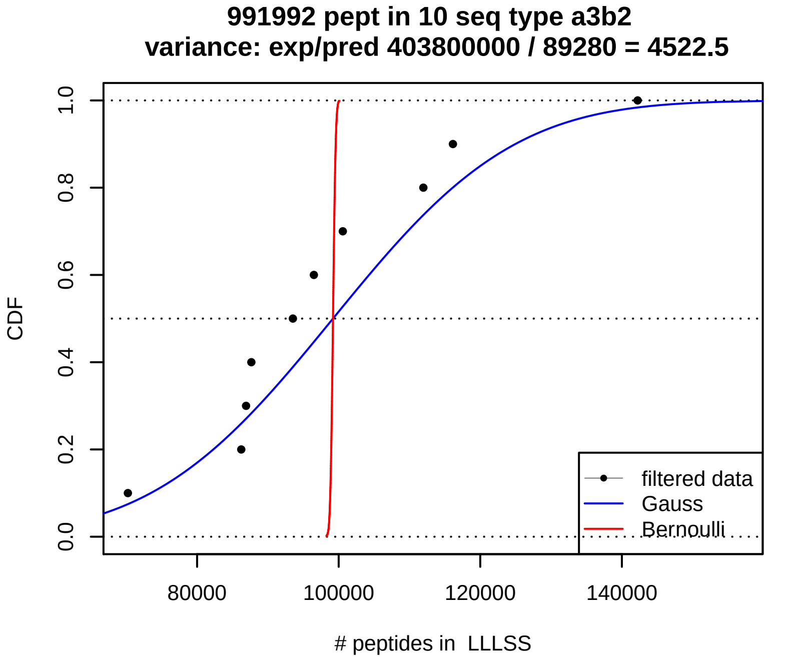
991992 pept in 10 seq type a3b2
variance: exp/pred 403800000 / 89280 = 4522.5
CDF
# peptides in LLLSS
filtered data
Gauss
Bernoulli

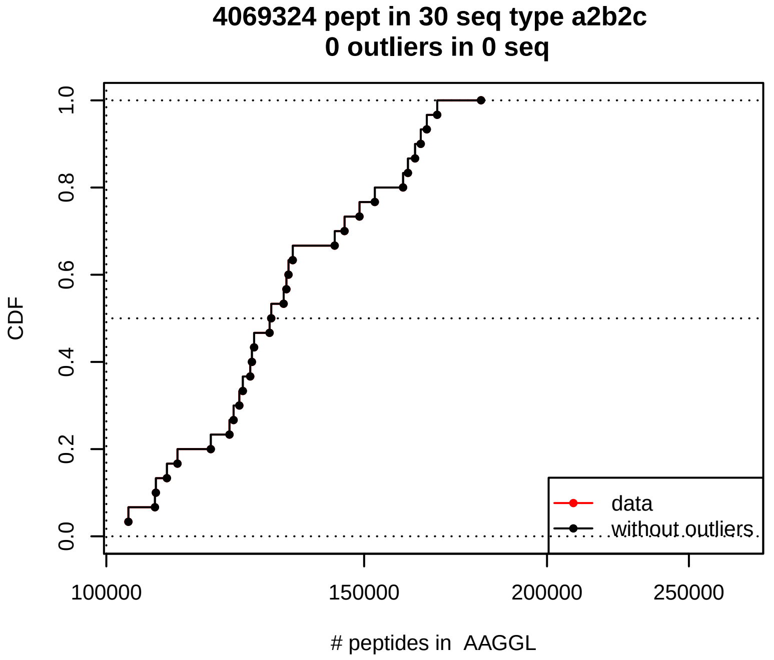
4069324 pept in 30 seq type a2b2c
0 outliers in 0 seq
CDF
# peptides in AAGGL
data
without outliers

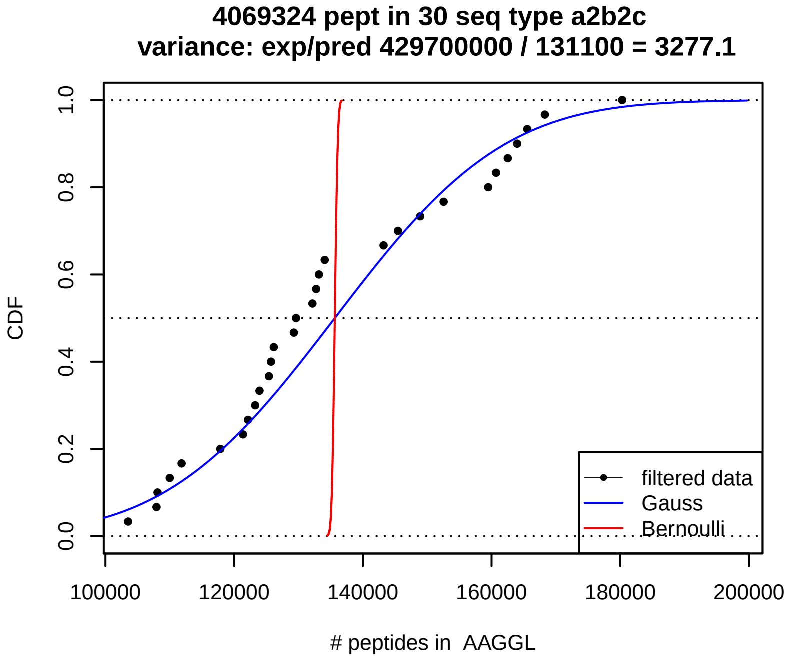
4069324 pept in 30 seq type a2b2c
variance: exp/pred 429700000 / 131100 = 3277.1
CDF
# peptides in AAGGL
filtered data
Gauss
Bernoulli

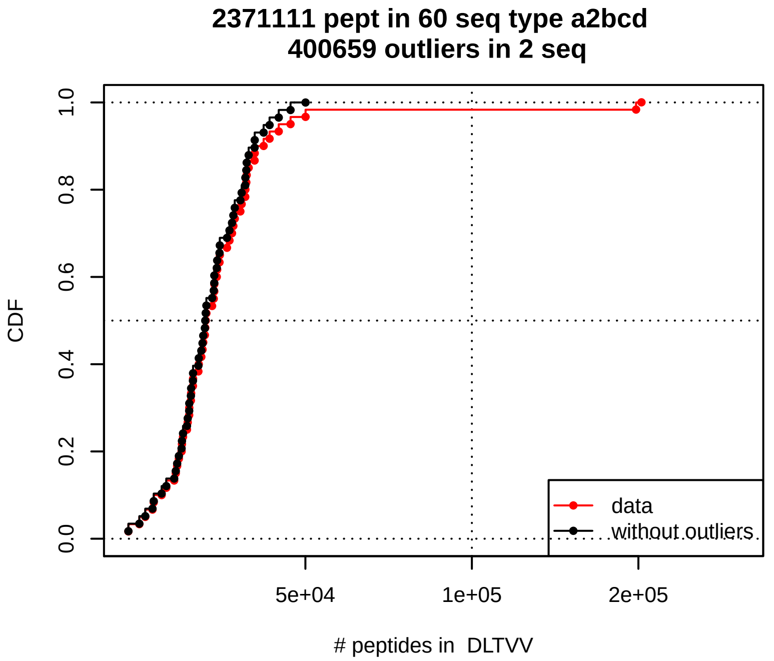
2371111 pept in 60 seq type a2bcd
400659 outliers in 2 seq
CDF
0.0
0.2
0.4
0.6
0.8
1.0
5e+04
1e+05
2e+05
# peptides in DLTVV
data
without outliers

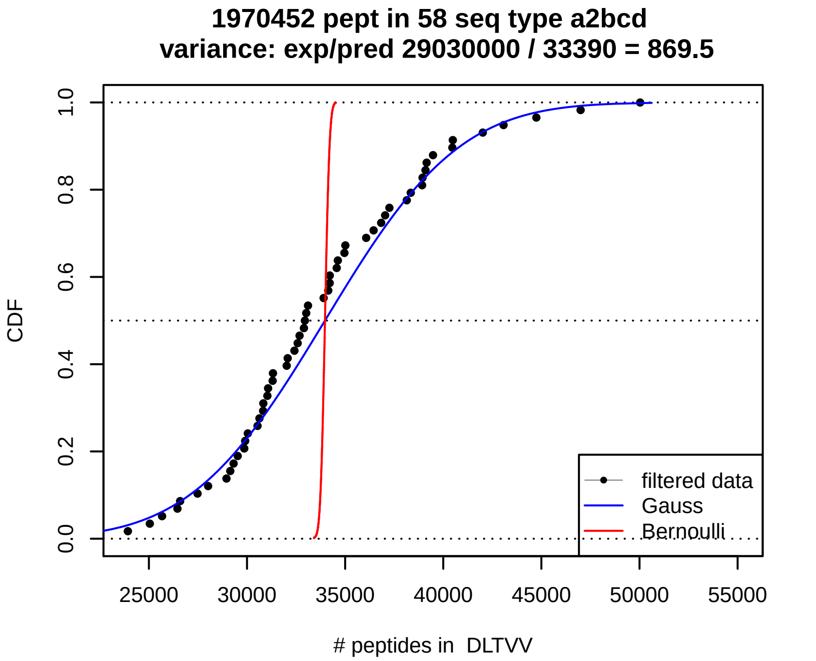
1970452 pept in 58 seq type a2bcd
variance: exp/pred 29030000 / 33390 = 869.5
CDF
0.0
0.2
0.4
0.6
0.8
1.0
25000
30000
35000
40000
45000
50000
55000
# peptides in DLTVV
filtered data
Gauss
Bernoulli

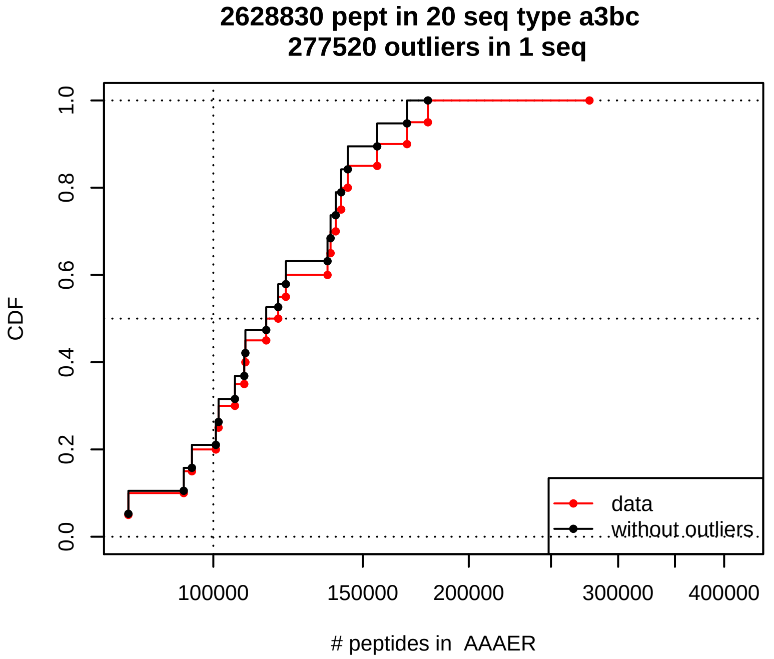
2628830 pept in 20 seq type a3bc
277520 outliers in 1 seq
CDF
0.0
0.2
0.4
0.6
0.8
1.0
100000
150000
200000
300000
400000
# peptides in AAAER
data
without outliers

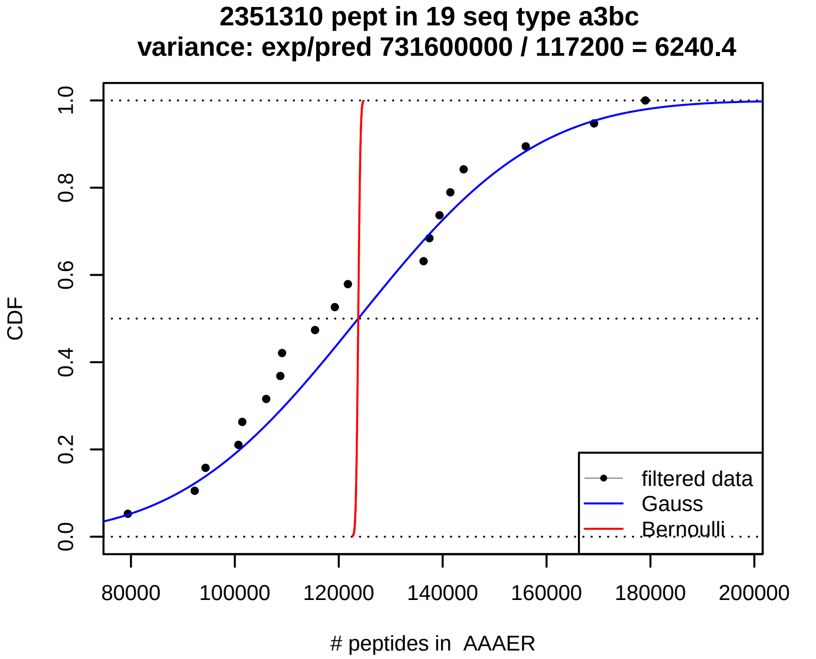
2351310 pept in 19 seq type a3bc
variance: exp/pred 731600000 / 117200 = 6240.4
CDF
0.0
0.2
0.4
0.6
0.8
1.0
80000
100000
120000
140000
160000
180000
200000
# peptides in AAAER
filtered data
Gauss
Bernoulli

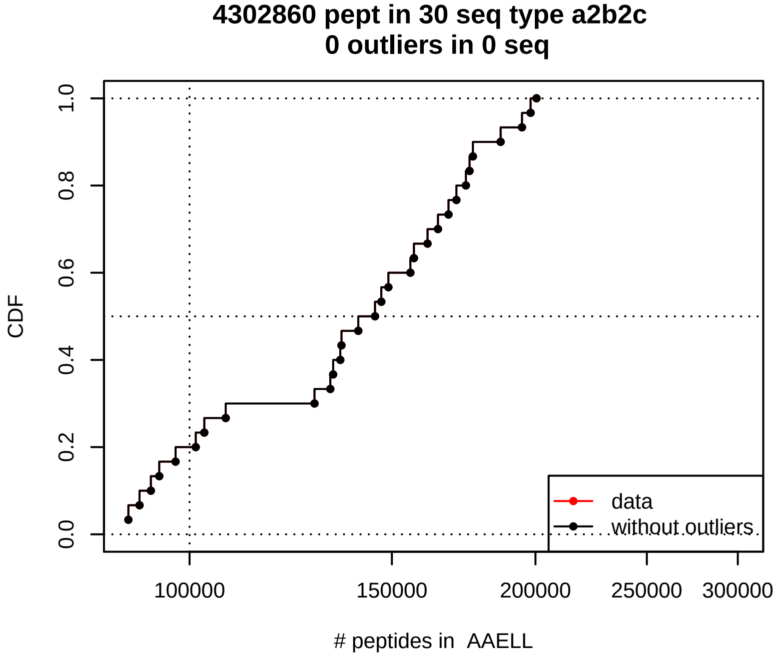
4302860 pept in 30 seq type a2b2c
0 outliers in 0 seq
CDF
0.0
0.2
0.4
0.6
0.8
1.0
100000
150000
200000
250000
300000
# peptides in AAELL
data
without outliers

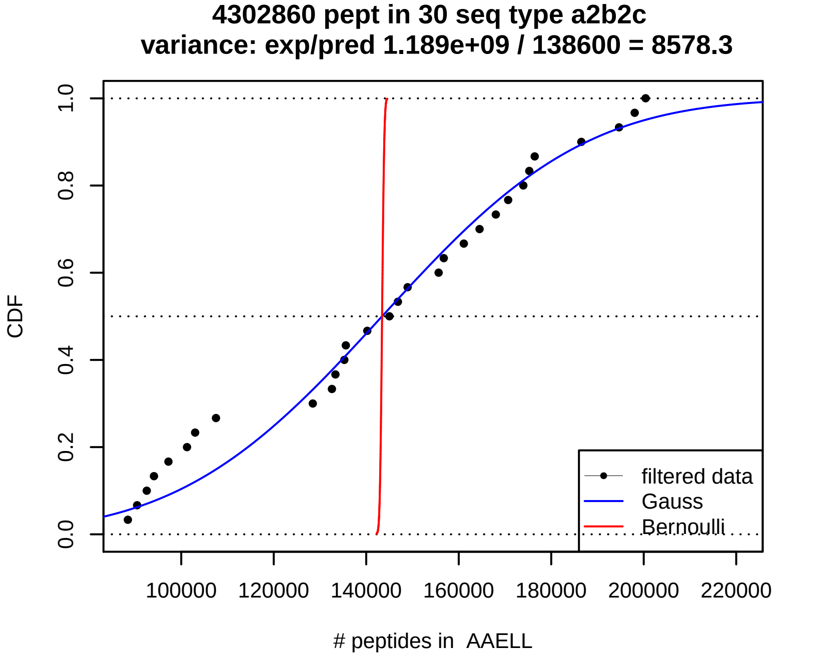
4302860 pept in 30 seq type a2b2c
variance: exp/pred 1.189e+09 / 138600 = 8578.3
CDF
0.0
0.2
0.4
0.6
0.8
1.0
100000
120000
140000
160000
180000
200000
220000
# peptides in AAELL
filtered data
Gauss
Bernoulli

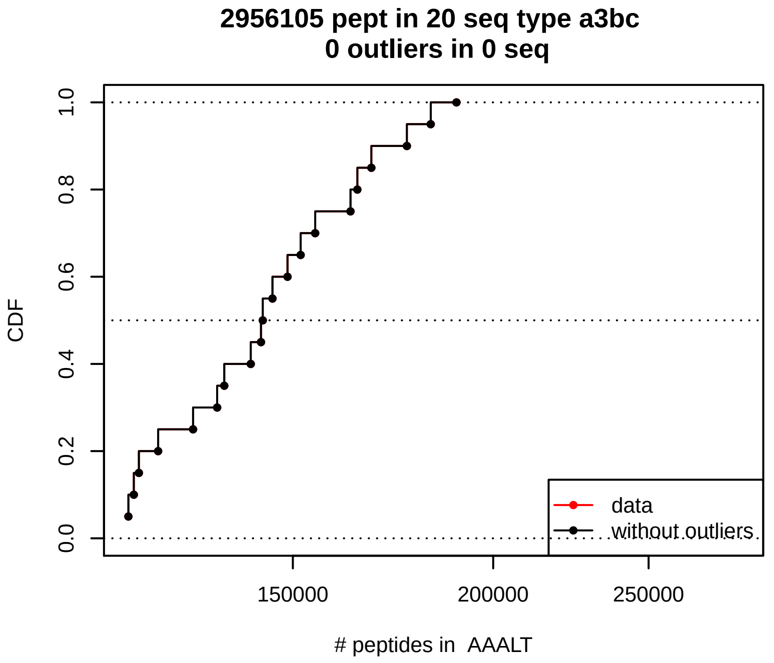
2956105 pept in 20 seq type a3bc
0 outliers in 0 seq
CDF
0.0
0.2
0.4
0.6
0.8
1.0
150000
200000
250000
# peptides in AAALT
data
without outliers

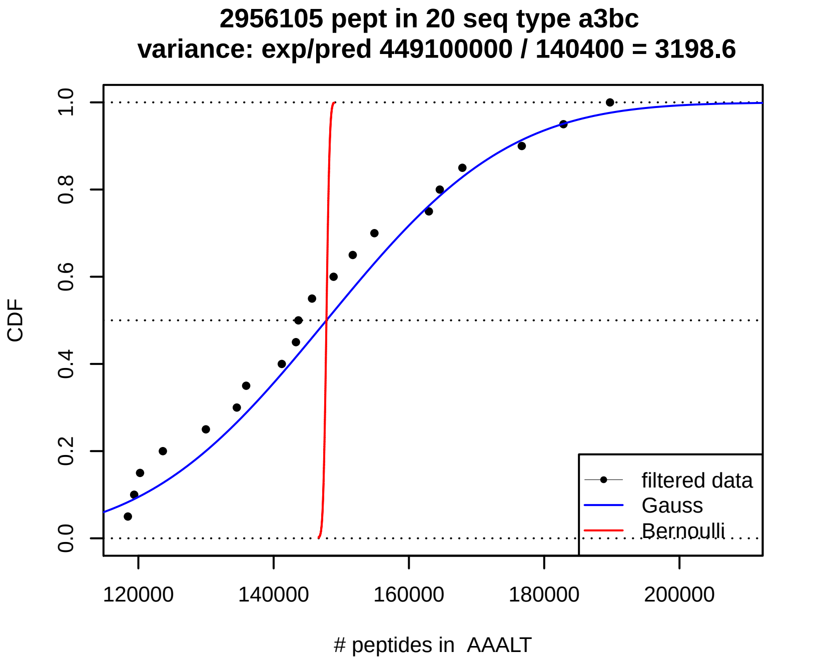
2956105 pept in 20 seq type a3bc
variance: exp/pred 449100000 / 140400 = 3198.6
CDF
0.0
0.2
0.4
0.6
0.8
1.0
120000
140000
160000
180000
200000
# peptides in AAALT
filtered data
Gauss
Bernoulli

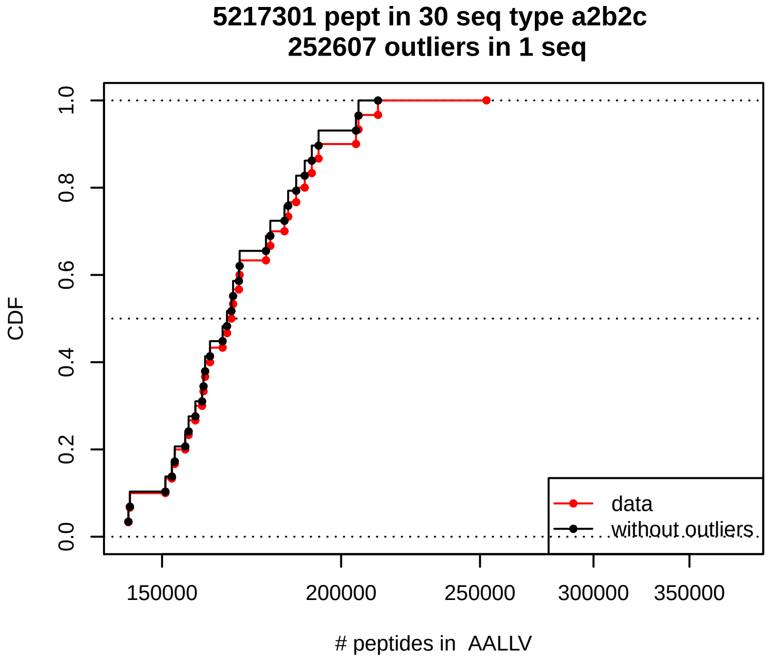
5217301 pept in 30 seq type a2b2c
252607 outliers in 1 seq
CDF
0.0
0.2
0.4
0.6
0.8
1.0
150000
200000
250000
300000
350000
# peptides in AALLV
data
without outliers

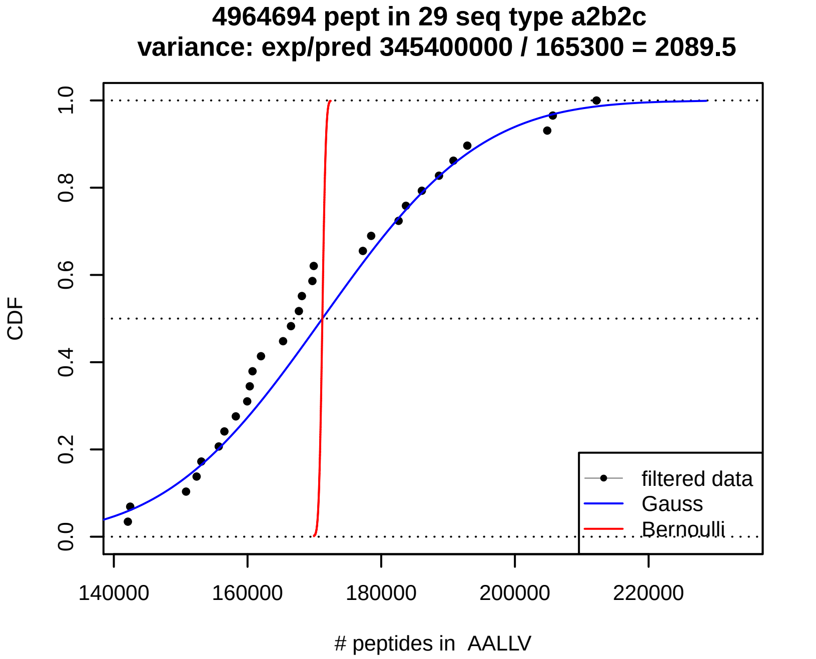
4964694 pept in 29 seq type a2b2c
variance: exp/pred 345400000 / 165300 = 2089.5
CDF
0.0
0.2
0.4
0.6
0.8
1.0
140000
160000
180000
200000
220000
# peptides in AALLV
filtered data
Gauss
Bernoulli

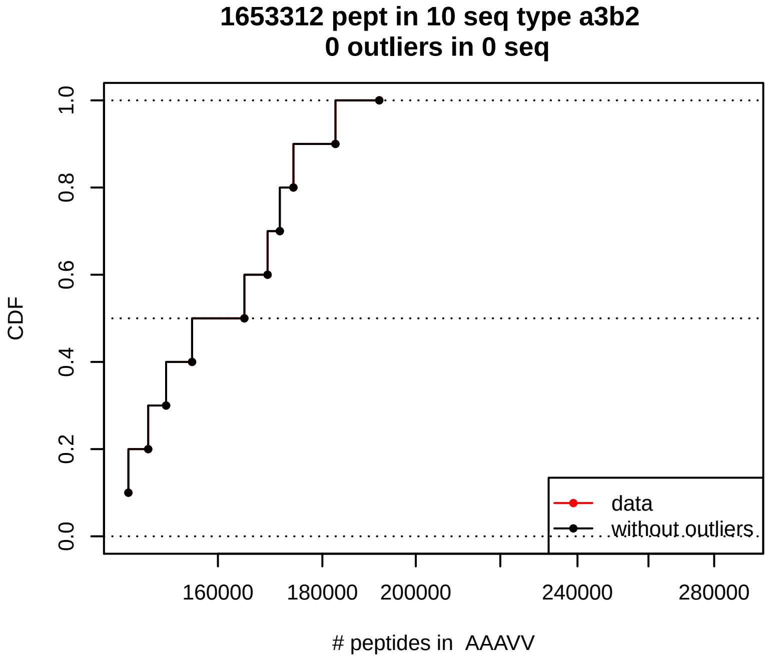
1653312 pept in 10 seq type a3b2
0 outliers in 0 seq
CDF
0.0
0.2
0.4
0.6
0.8
1.0
160000
180000
200000
240000
280000
# peptides in AAAVV
data
without outliers

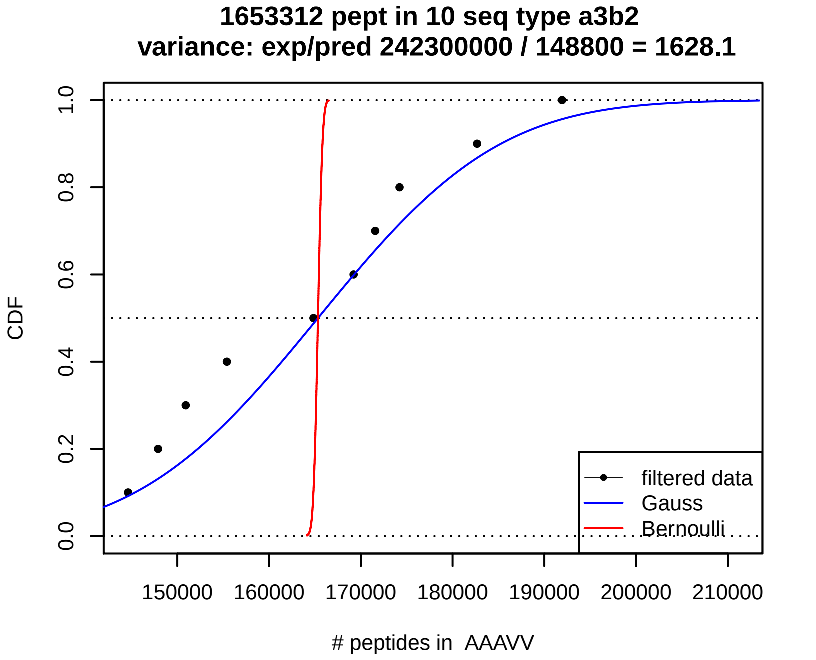
1653312 pept in 10 seq type a3b2
variance: exp/pred 242300000 / 148800 = 1628.1
CDF
0.0
0.2
0.4
0.6
0.8
1.0
150000
160000
170000
180000
190000
200000
210000
# peptides in AAAVV
filtered data
Gauss
Bernoulli

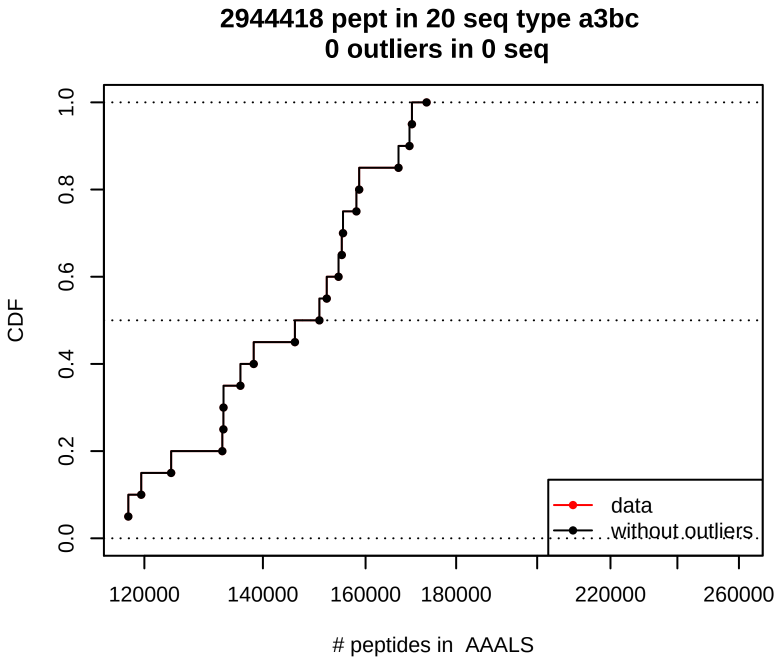

2944418 pept in 20 seq type a3bc
0 outliers in 0 seq
CDF
# peptides in AAALS
data
without outliers


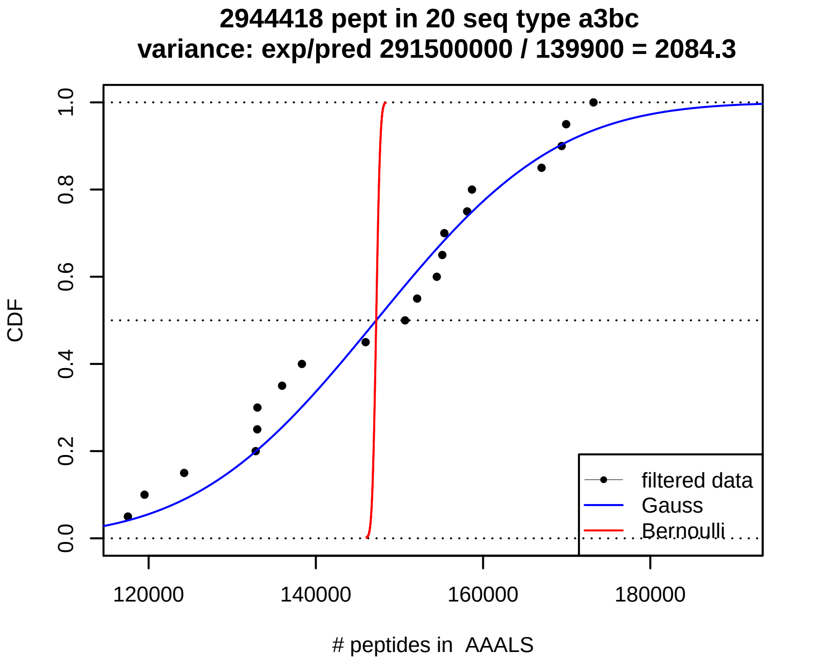

2944418 pept in 20 seq type a3bc
variance: exp/pred 291500000 / 139900 = 2084.3
CDF
# peptides in AAALS
filtered data
Gauss
Bernoulli


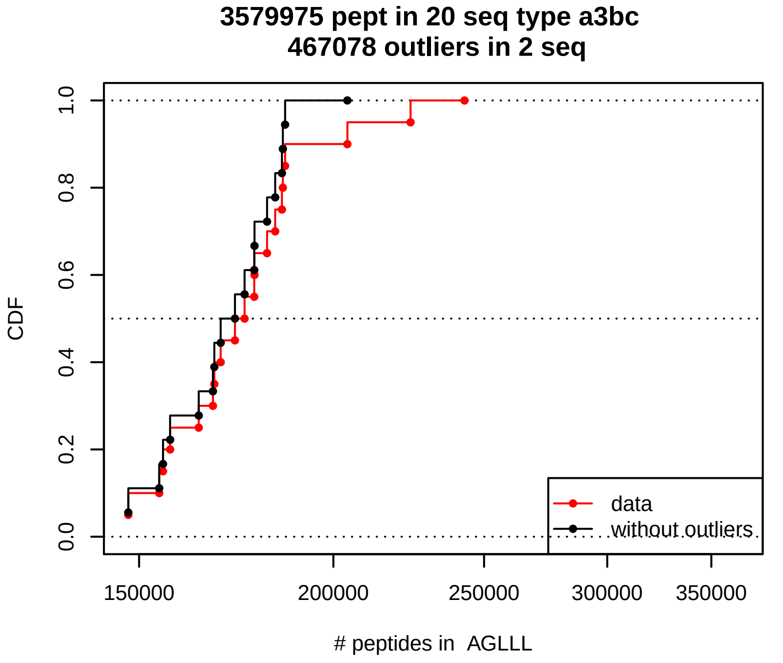

3579975 pept in 20 seq type a3bc
467078 outliers in 2 seq
CDF
# peptides in AGLLL
data
without outliers


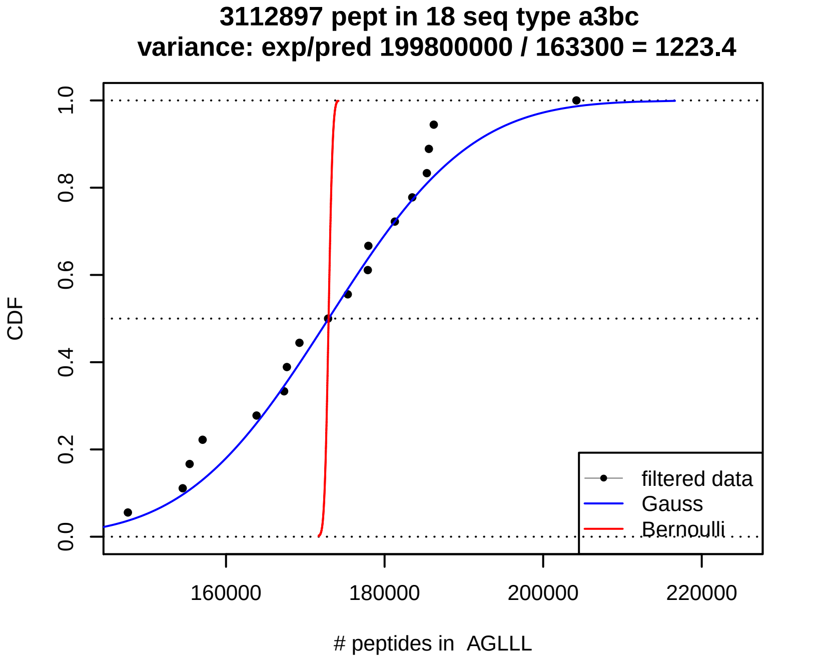

3112897 pept in 18 seq type a3bc
variance: exp/pred 199800000 / 163300 = 1223.4
CDF
# peptides in AGLLL
filtered data
Gauss
Bernoulli


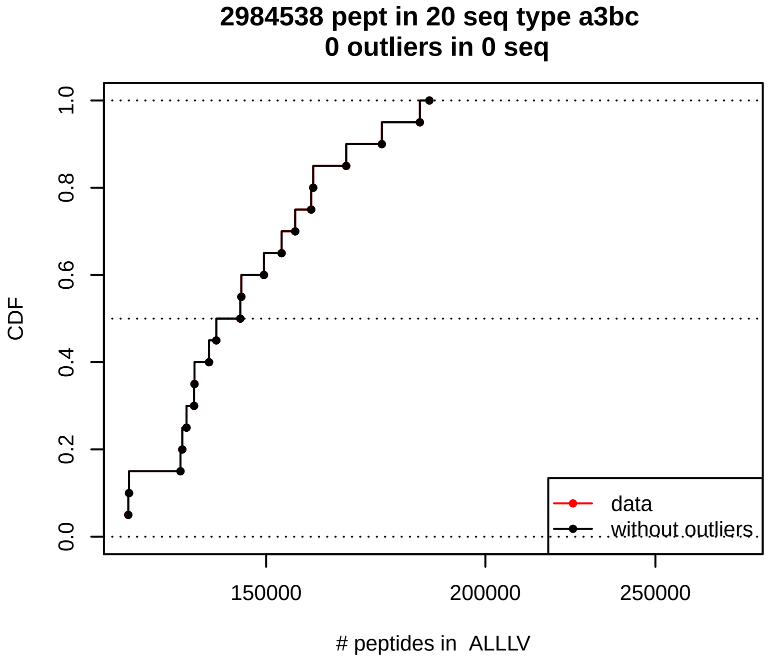

2984538 pept in 20 seq type a3bc
0 outliers in 0 seq
CDF
# peptides in ALLLV
data
without outliers


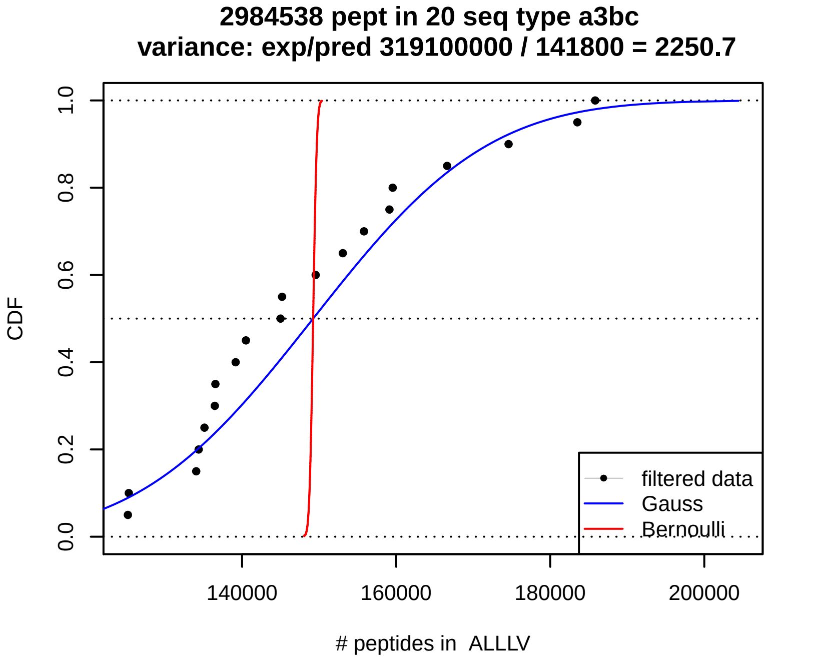

2984538 pept in 20 seq type a3bc
variance: exp/pred 319100000 / 141800 = 2250.7
CDF
# peptides in ALLLV
filtered data
Gauss
Bernoulli

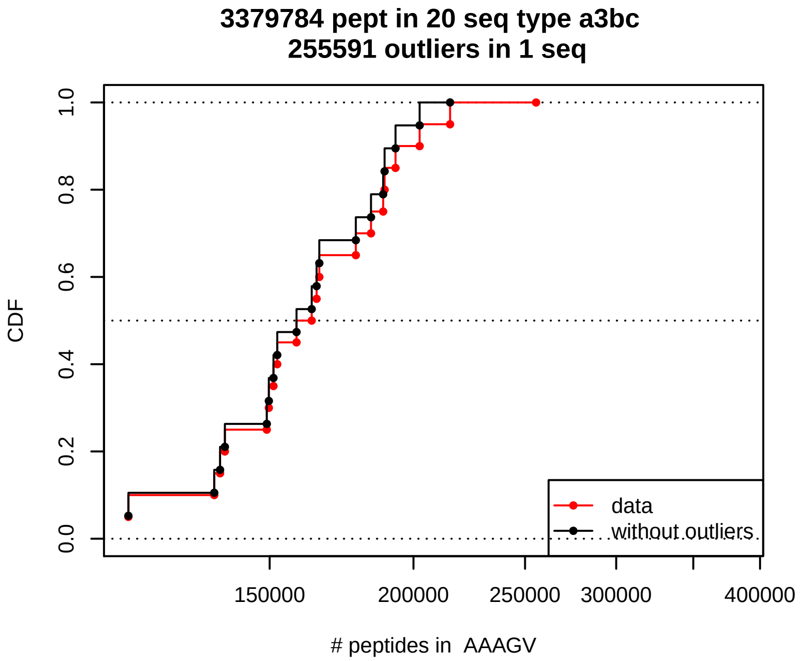
3379784 pept in 20 seq type a3bc
255591 outliers in 1 seq
CDF
0.0
0.2
0.4
0.6
0.8
1.0
150000
200000
250000
300000
400000
# peptides in AAAGV
data
without outliers

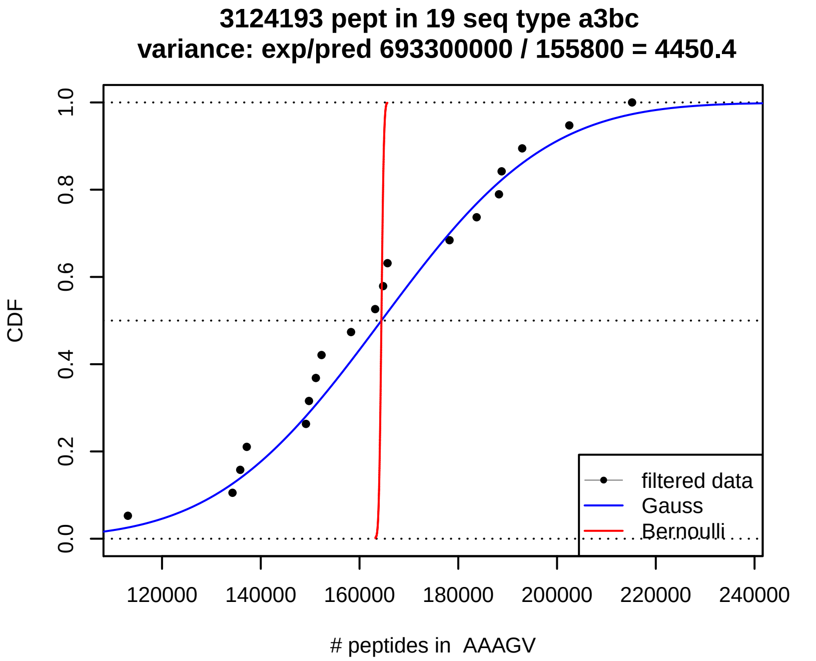
3124193 pept in 19 seq type a3bc
variance: exp/pred 693300000 / 155800 = 4450.4
CDF
0.0
0.2
0.4
0.6
0.8
1.0
120000
140000
160000
180000
200000
220000
240000
# peptides in AAAGV
filtered data
Gauss
Bernoulli

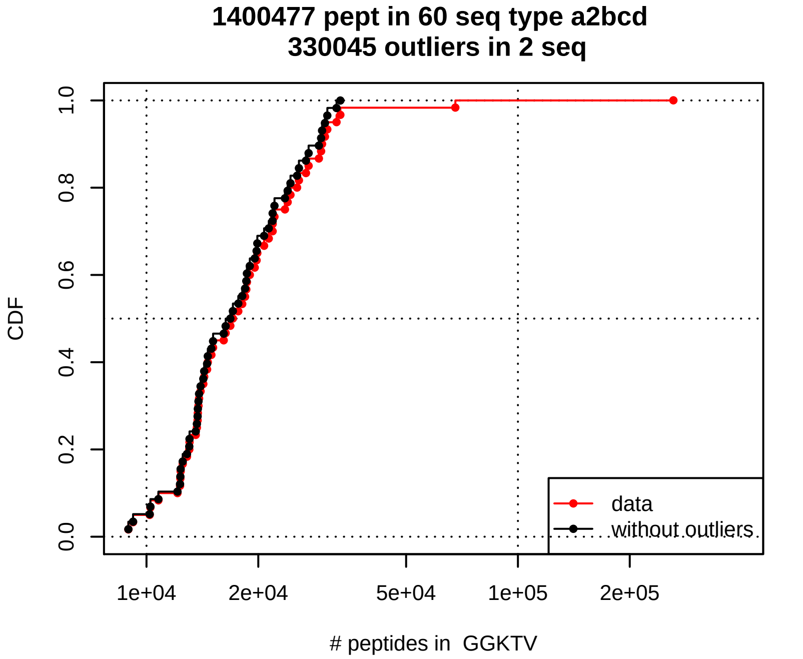
1400477 pept in 60 seq type a2bcd
330045 outliers in 2 seq
CDF
0.0
0.2
0.4
0.6
0.8
1.0
1e+04
2e+04
5e+04
1e+05
2e+05
# peptides in GGKTV
data
without outliers

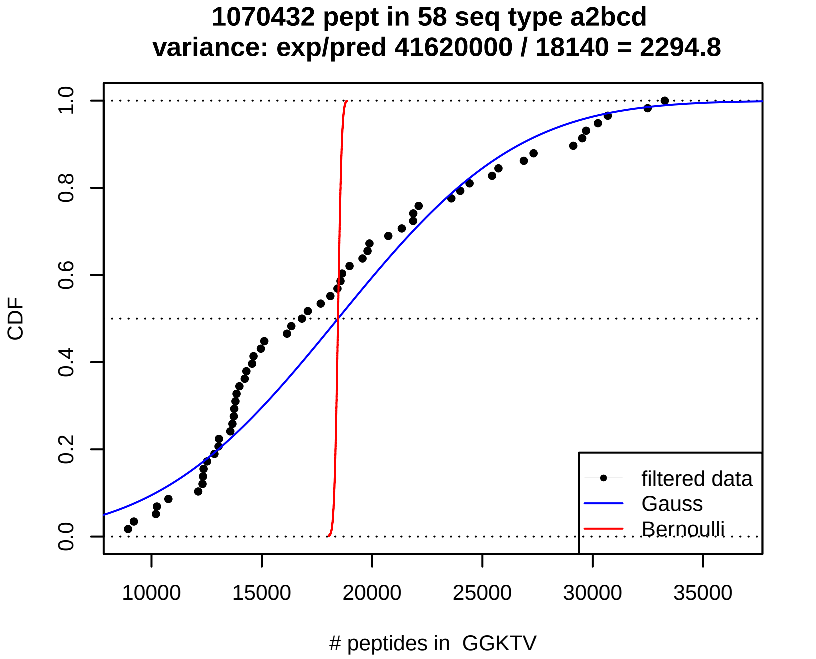
1070432 pept in 58 seq type a2bcd
variance: exp/pred 41620000 / 18140 = 2294.8
CDF
0.0
0.2
0.4
0.6
0.8
1.0
10000
15000
20000
25000
30000
35000
# peptides in GGKTV
filtered data
Gauss
Bernoulli

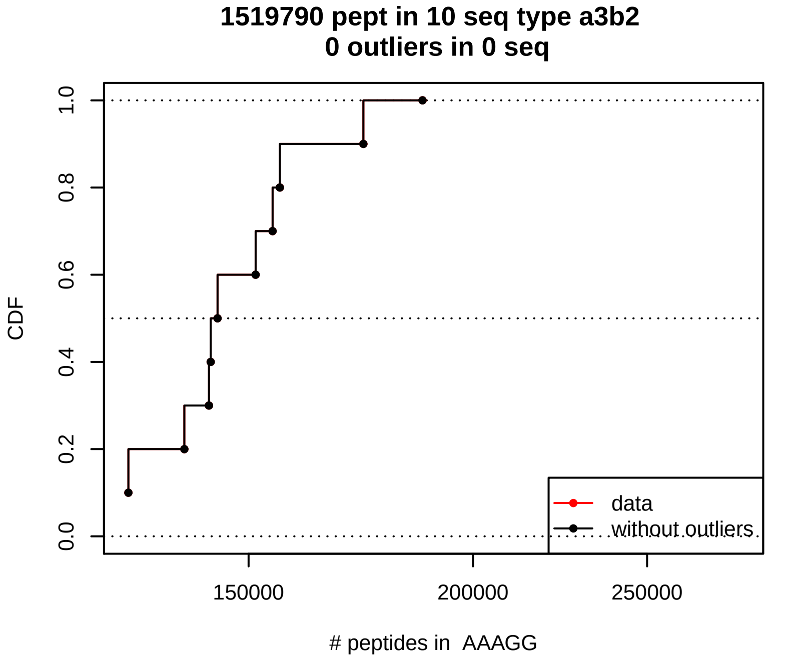
1519790 pept in 10 seq type a3b2
0 outliers in 0 seq
CDF
0.0
0.2
0.4
0.6
0.8
1.0
150000
200000
250000
# peptides in AAAGG
data
without outliers

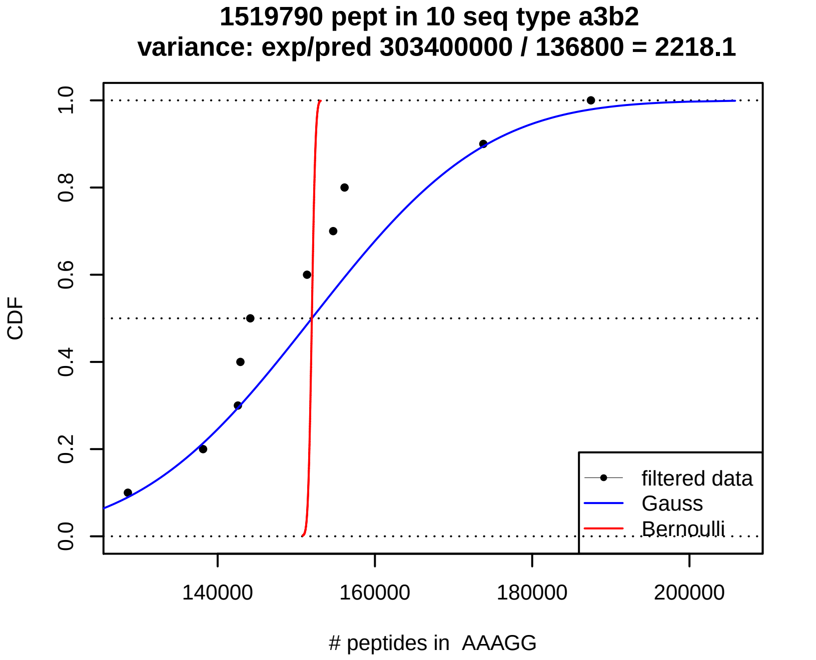
1519790 pept in 10 seq type a3b2
variance: exp/pred 303400000 / 136800 = 2218.1
CDF
0.0
0.2
0.4
0.6
0.8
1.0
140000
160000
180000
200000
# peptides in AAAGG
filtered data
Gauss
Bernoulli

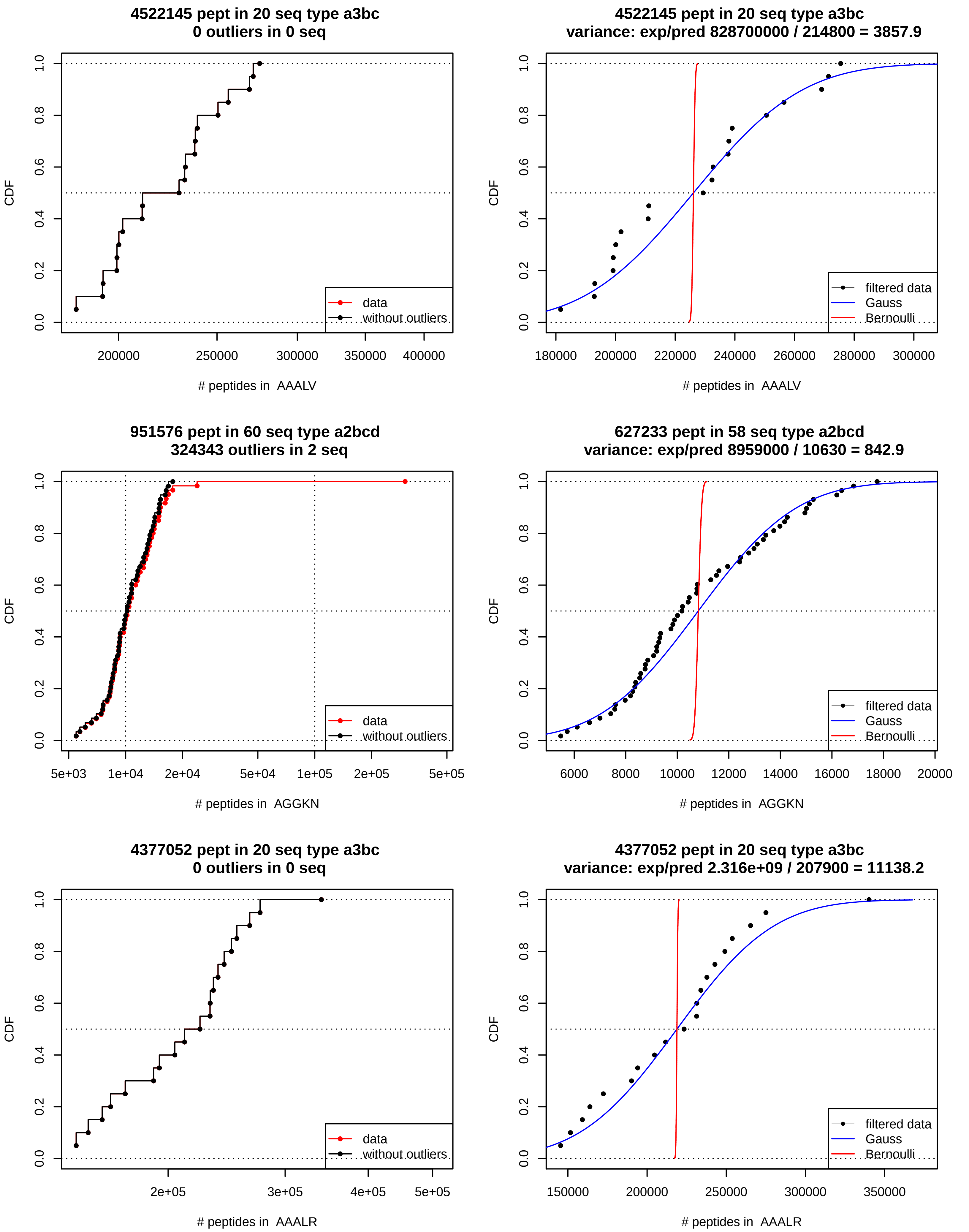
4522145 pept in 20 seq type a3bc
0 outliers in 0 seq
CDF
# peptides in AAALV
data
without outliers
4522145 pept in 20 seq type a3bc
variance: exp/pred 828700000 / 214800 = 3857.9
filtered data
Gauss
Bernoulli
951576 pept in 60 seq type a2bcd
324343 outliers in 2 seq
# peptides in AGGKN
627233 pept in 58 seq type a2bcd
variance: exp/pred 8959000 / 10630 = 842.9
4377052 pept in 20 seq type a3bc
0 outliers in 0 seq
# peptides in AAALR
4377052 pept in 20 seq type a3bc
variance: exp/pred 2.316e+09 / 207900 = 11138.2

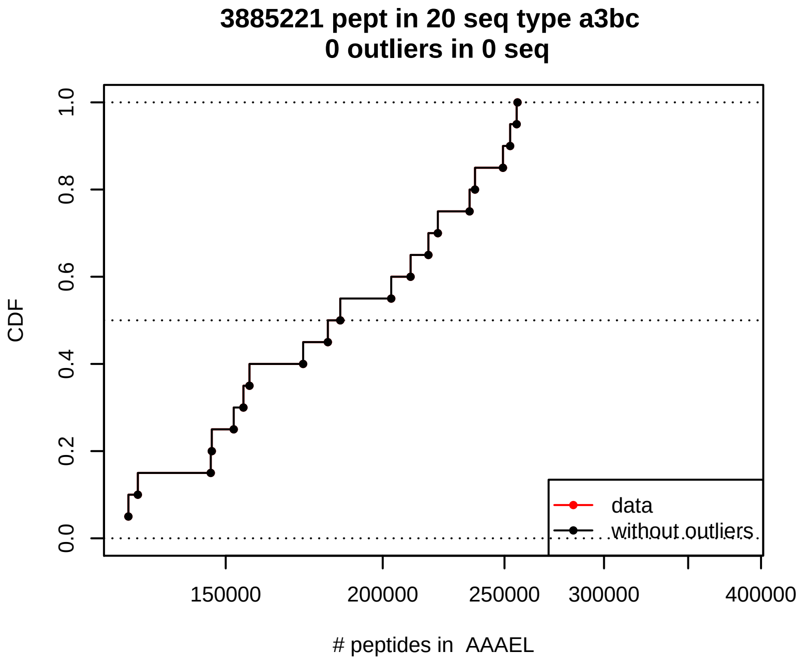
3885221 pept in 20 seq type a3bc
0 outliers in 0 seq
CDF
0.0
0.2
0.4
0.6
0.8
1.0
150000
200000
250000
300000
400000
# peptides in AAAEL
data
without outliers

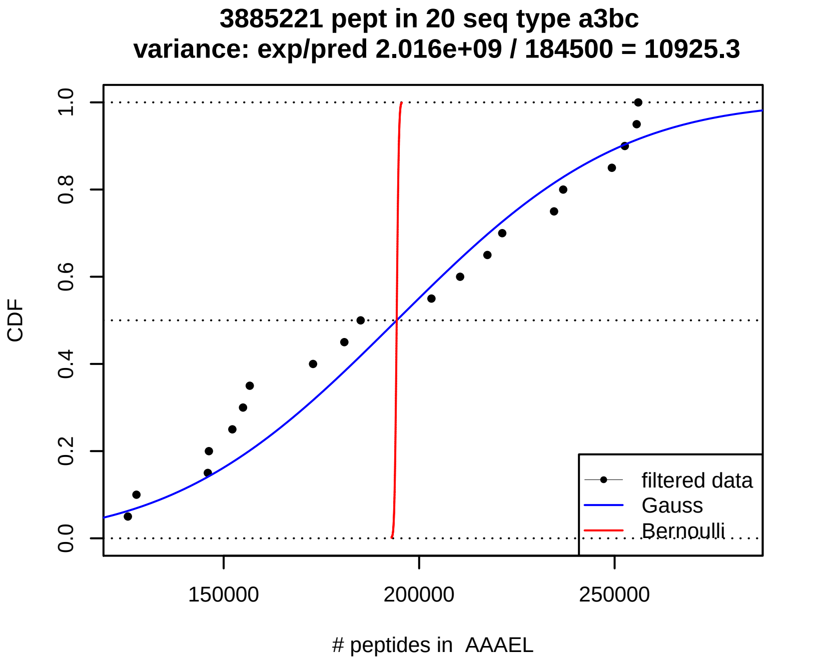
3885221 pept in 20 seq type a3bc
variance: exp/pred 2.016e+09 / 184500 = 10925.3
CDF
0.0
0.2
0.4
0.6
0.8
1.0
150000
200000
250000
# peptides in AAAEL
filtered data
Gauss
Bernoulli

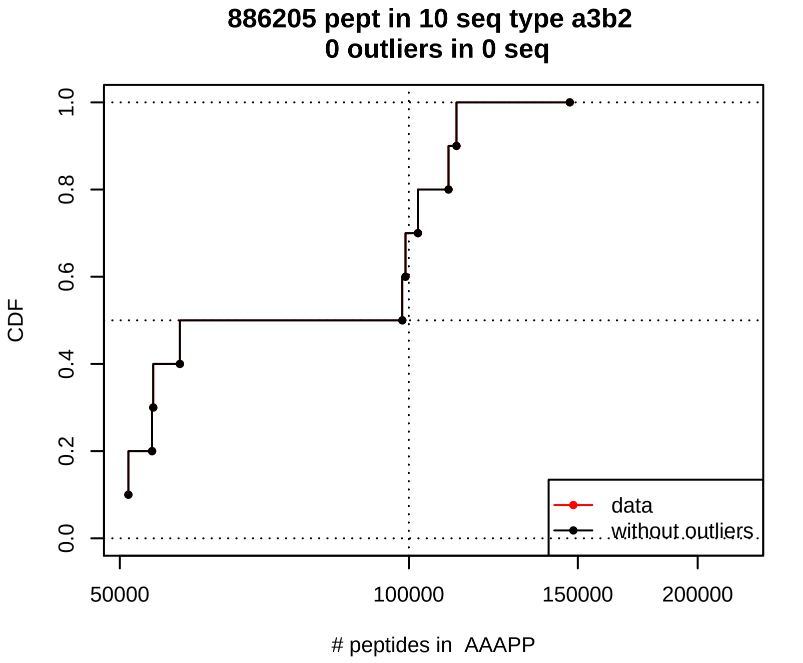
886205 pept in 10 seq type a3b2
0 outliers in 0 seq
CDF
0.0
0.2
0.4
0.6
0.8
1.0
50000
100000
150000
200000
# peptides in AAAPP
data
without outliers

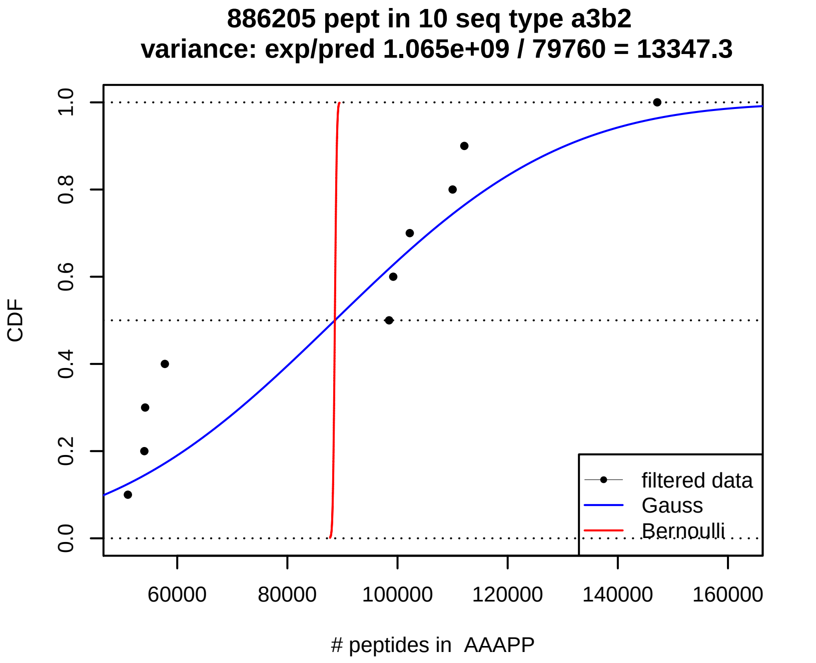
886205 pept in 10 seq type a3b2
variance: exp/pred 1.065e+09 / 79760 = 13347.3
CDF
0.0
0.2
0.4
0.6
0.8
1.0
60000
80000
100000
120000
140000
160000
# peptides in AAAPP
filtered data
Gauss
Bernoulli

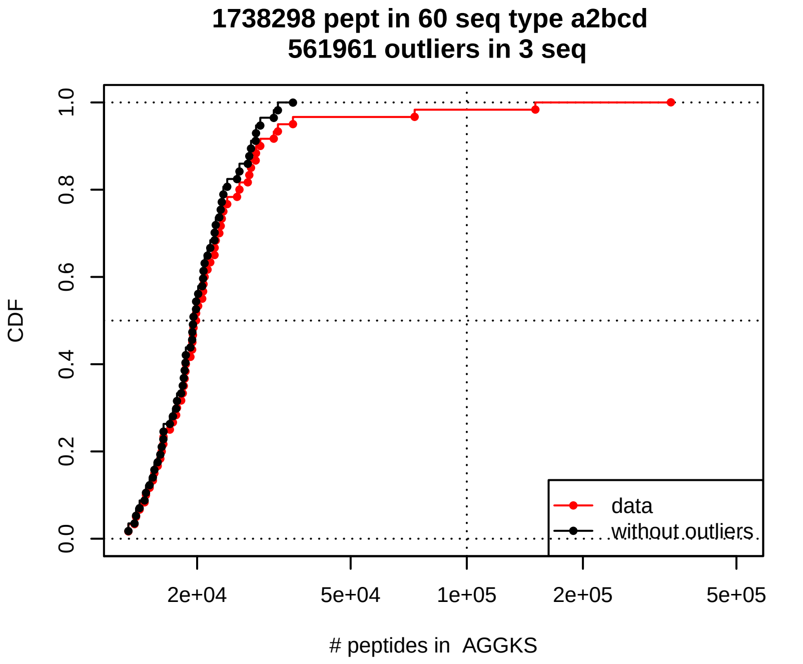
1738298 pept in 60 seq type a2bcd
561961 outliers in 3 seq
CDF
0.0
0.2
0.4
0.6
0.8
1.0
2e+04
5e+04
1e+05
2e+05
5e+05
# peptides in AGGKS
data
without outliers

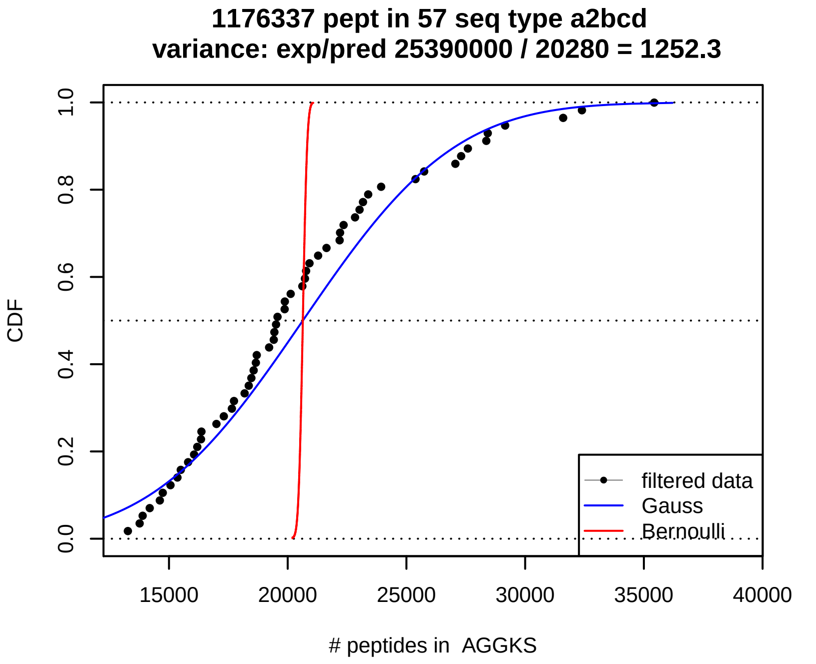
1176337 pept in 57 seq type a2bcd
variance: exp/pred 25390000 / 20280 = 1252.3
CDF
0.0
0.2
0.4
0.6
0.8
1.0
15000
20000
25000
30000
35000
40000
# peptides in AGGKS
filtered data
Gauss
Bernoulli

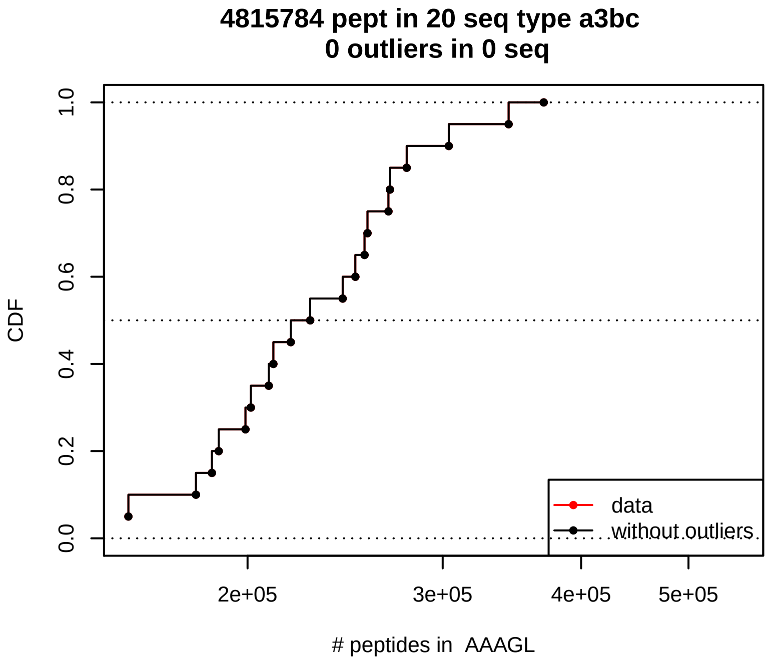
4815784 pept in 20 seq type a3bc
0 outliers in 0 seq
CDF
0.0
0.2
0.4
0.6
0.8
1.0
2e+05
3e+05
4e+05
5e+05
# peptides in AAAGL
data
without outliers

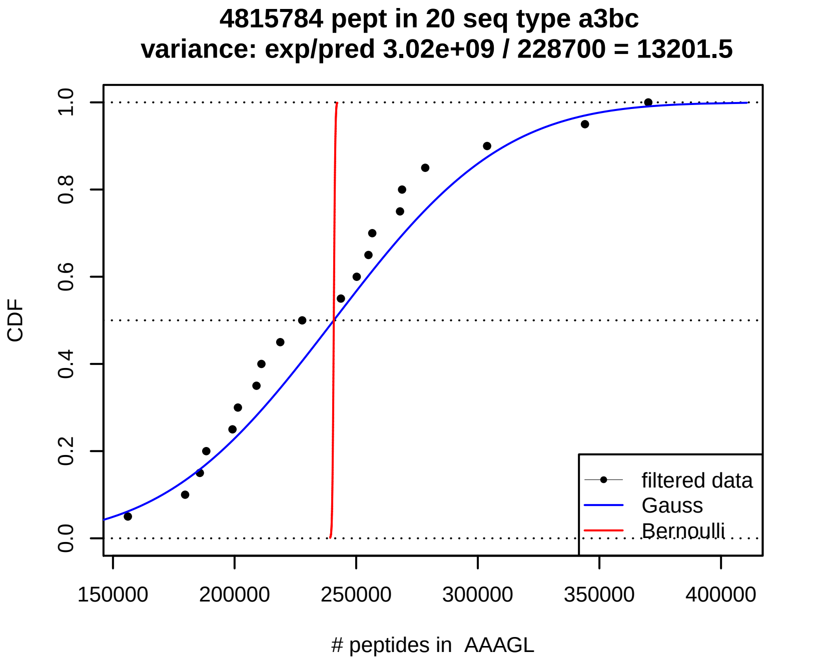
4815784 pept in 20 seq type a3bc
variance: exp/pred 3.02e+09 / 228700 = 13201.5
CDF
0.0
0.2
0.4
0.6
0.8
1.0
150000
200000
250000
300000
350000
400000
# peptides in AAAGL
filtered data
Gauss
Bernoulli

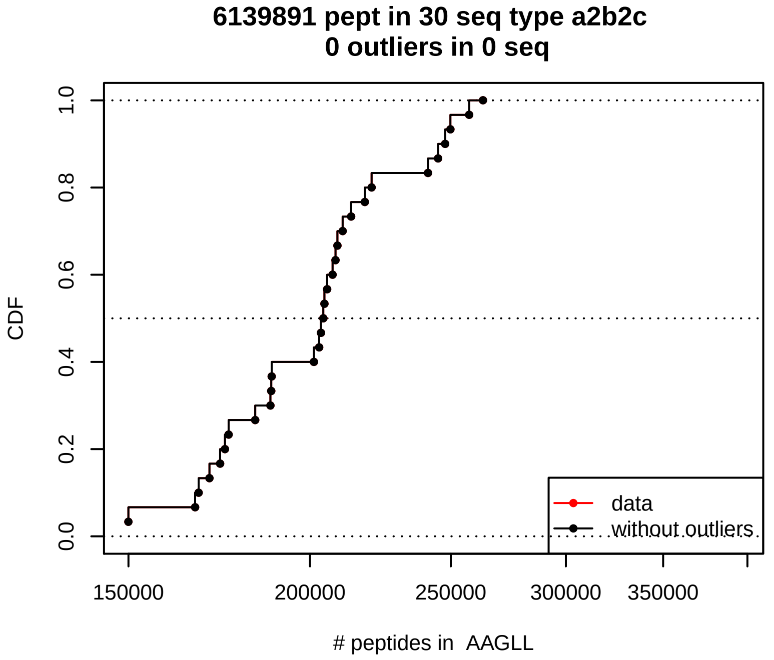
6139891 pept in 30 seq type a2b2c
0 outliers in 0 seq
CDF
0.0
0.2
0.4
0.6
0.8
1.0
150000
200000
250000
300000
350000
# peptides in AAGLL
data
without outliers

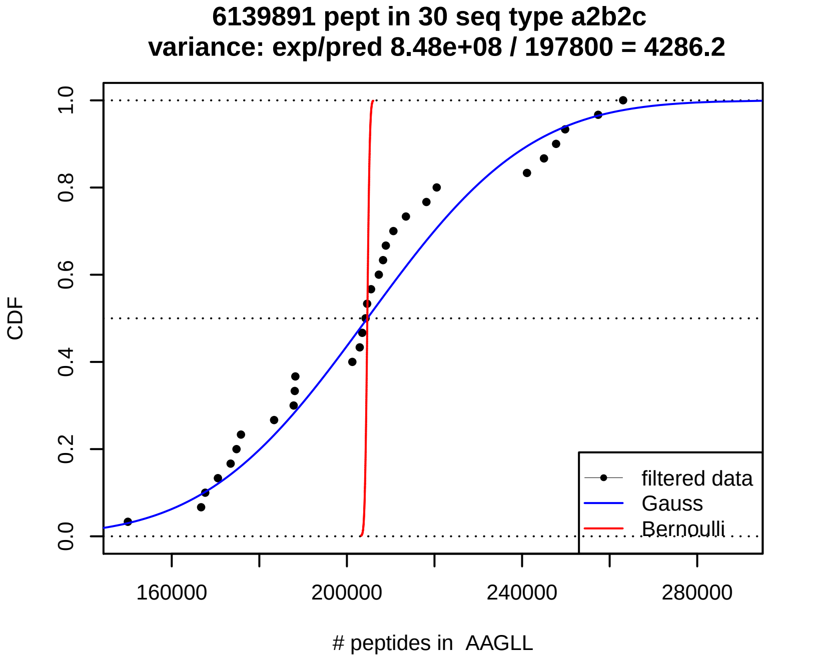
6139891 pept in 30 seq type a2b2c
variance: exp/pred 8.48e+08 / 197800 = 4286.2
CDF
0.0
0.2
0.4
0.6
0.8
1.0
160000
200000
240000
280000
# peptides in AAGLL
filtered data
Gauss
Bernoulli

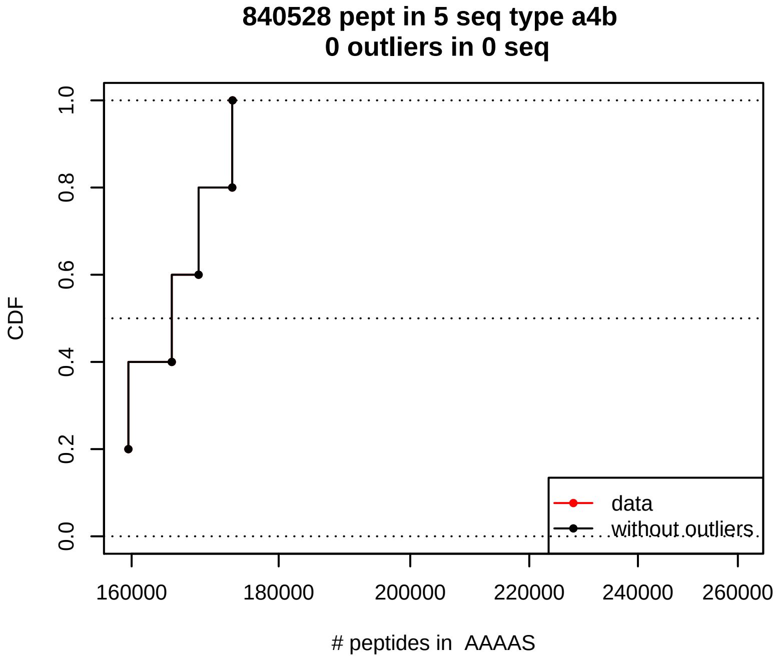
840528 pept in 5 seq type a4b
0 outliers in 0 seq
CDF
0.0
0.2
0.4
0.6
0.8
1.0
160000
180000
200000
220000
240000
260000
# peptides in AAAAS
data
without outliers

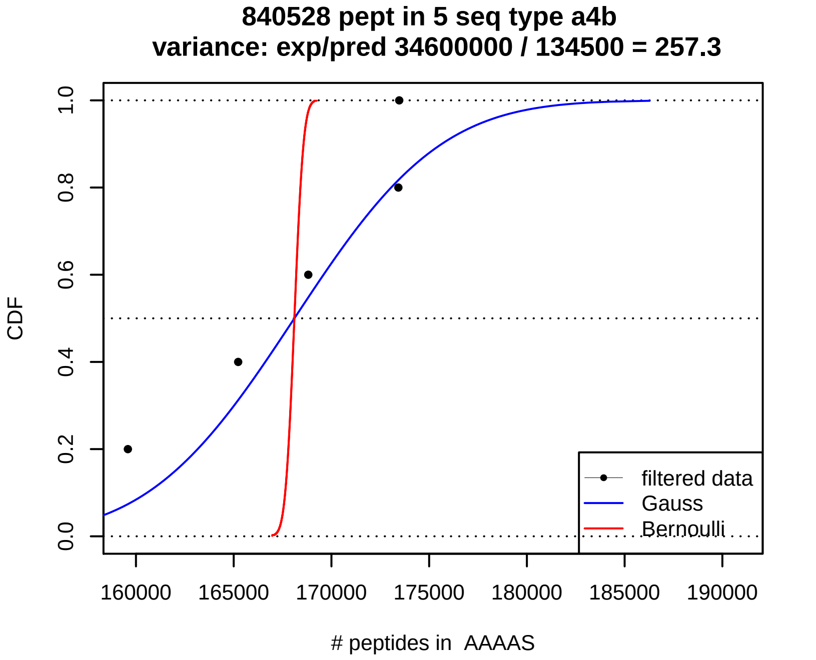
840528 pept in 5 seq type a4b
variance: exp/pred 34600000 / 134500 = 257.3
CDF
0.0
0.2
0.4
0.6
0.8
1.0
160000
165000
170000
175000
180000
185000
190000
# peptides in AAAAS
filtered data
Gauss
Bernoulli

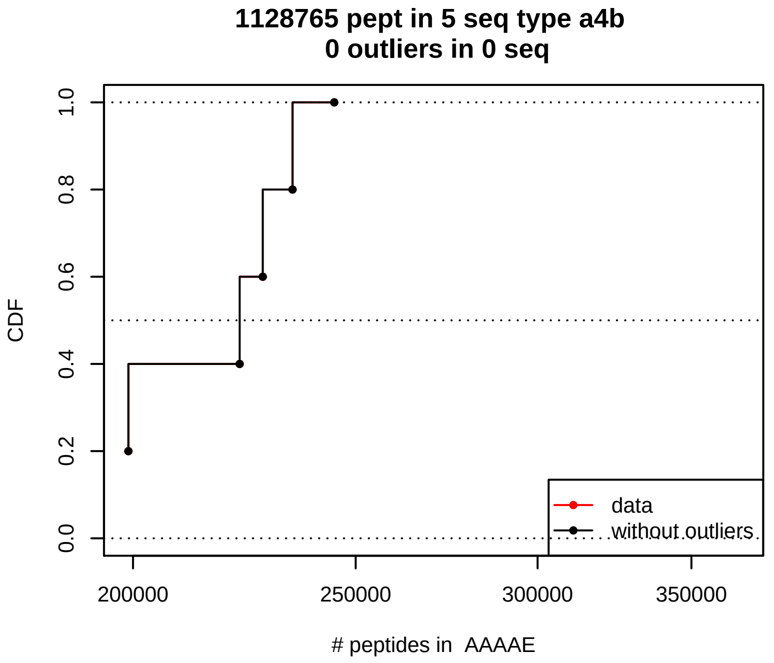
1128765 pept in 5 seq type a4b
0 outliers in 0 seq
CDF
0.0
0.2
0.4
0.6
0.8
1.0
200000
250000
300000
350000
# peptides in AAAAE
data
without outliers

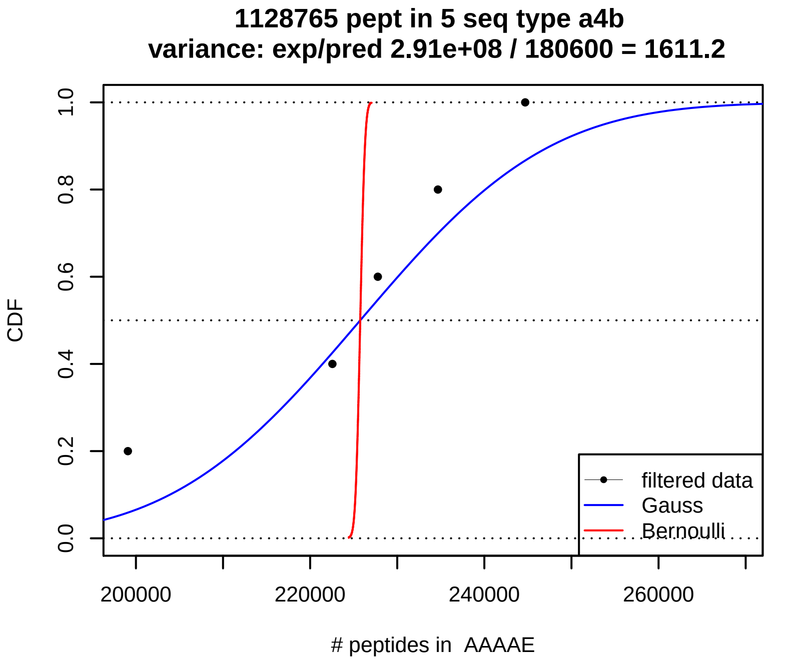
1128765 pept in 5 seq type a4b
variance: exp/pred 2.91e+08 / 180600 = 1611.2
CDF
0.0
0.2
0.4
0.6
0.8
1.0
200000
220000
240000
260000
# peptides in AAAAE
filtered data
Gauss
Bernoulli

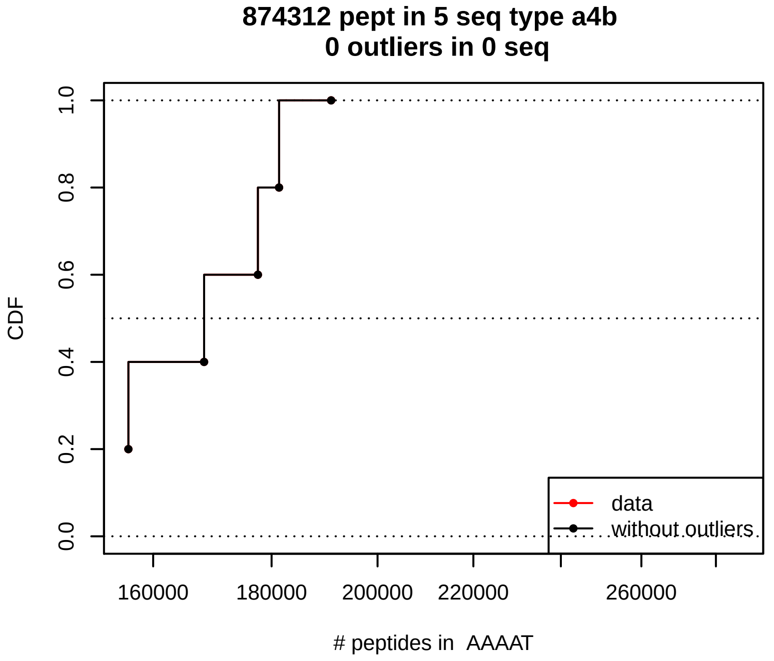
874312 pept in 5 seq type a4b
0 outliers in 0 seq
CDF
0.0
0.2
0.4
0.6
0.8
1.0
160000
180000
200000
220000
260000
# peptides in AAAAT
data
without outliers

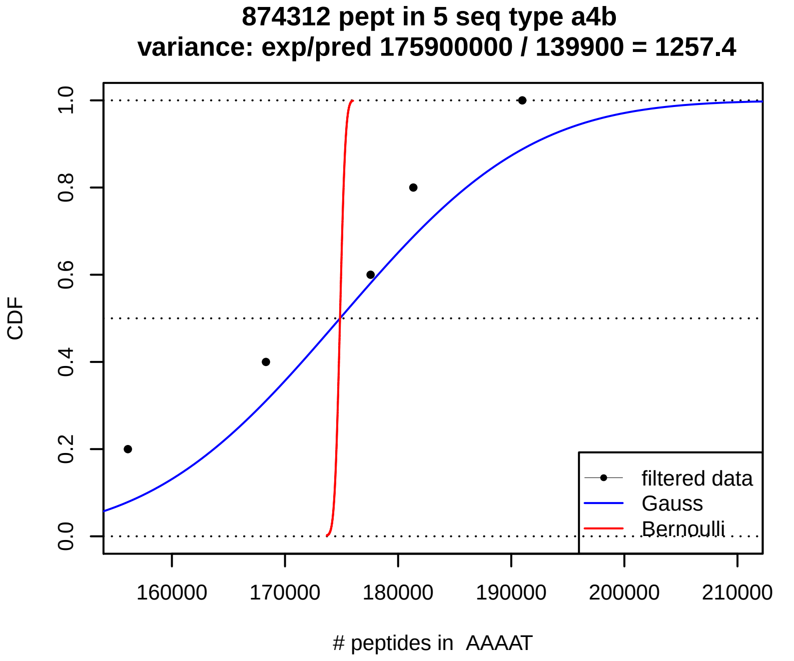
874312 pept in 5 seq type a4b
variance: exp/pred 175900000 / 139900 = 1257.4
CDF
0.0
0.2
0.4
0.6
0.8
1.0
160000
170000
180000
190000
200000
210000
# peptides in AAAAT
filtered data
Gauss
Bernoulli

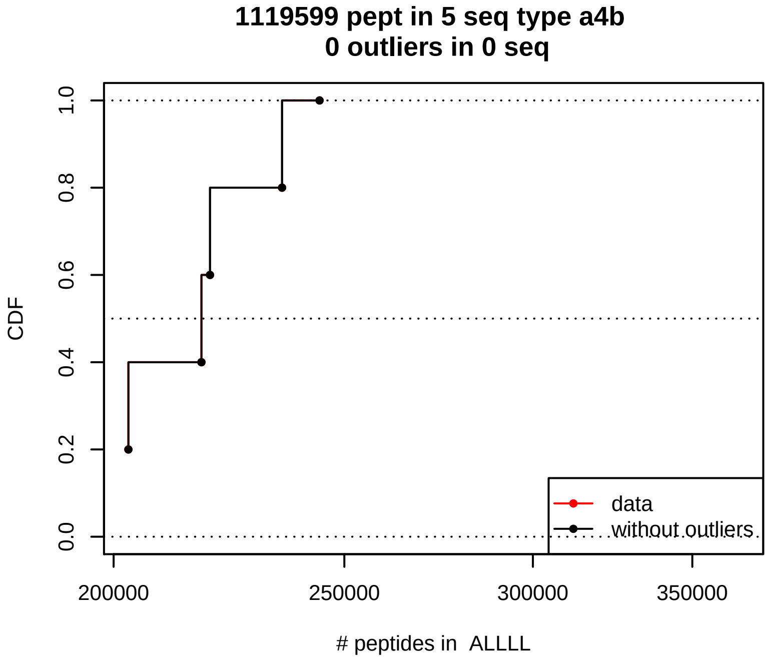
1119599 pept in 5 seq type a4b
0 outliers in 0 seq
CDF
0.0
0.2
0.4
0.6
0.8
1.0
200000
250000
300000
350000
# peptides in ALLLL
data
without outliers

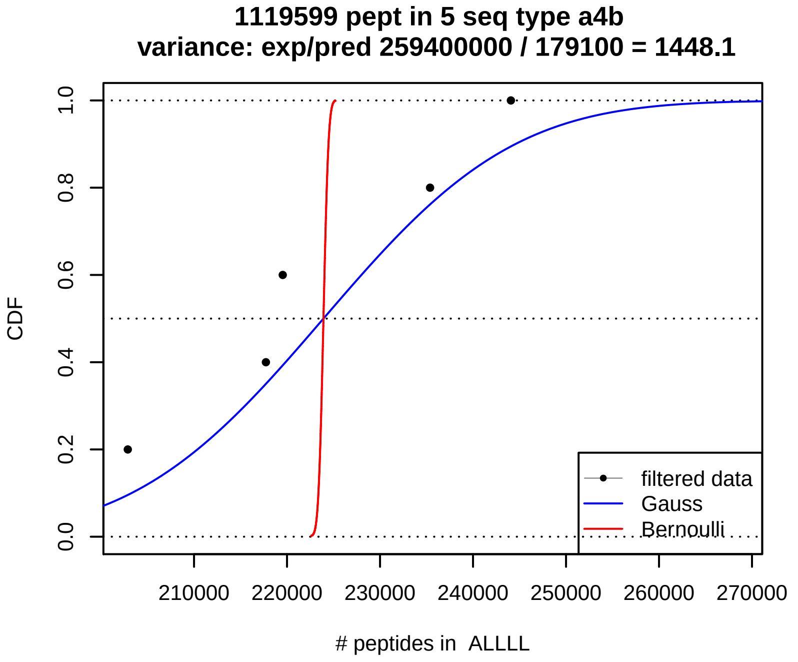
1119599 pept in 5 seq type a4b
variance: exp/pred 259400000 / 179100 = 1448.1
CDF
0.0
0.2
0.4
0.6
0.8
1.0
210000
220000
230000
240000
250000
260000
270000
# peptides in ALLLL
filtered data
Gauss
Bernoulli

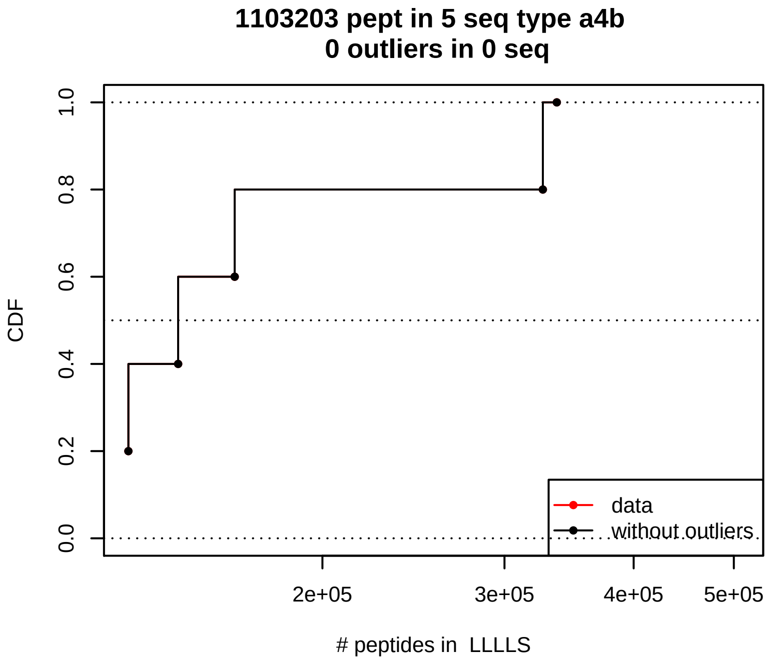
1103203 pept in 5 seq type a4b
0 outliers in 0 seq
CDF
0.0
0.2
0.4
0.6
0.8
1.0
2e+05
3e+05
4e+05
5e+05
# peptides in LLLLS
data
without outliers

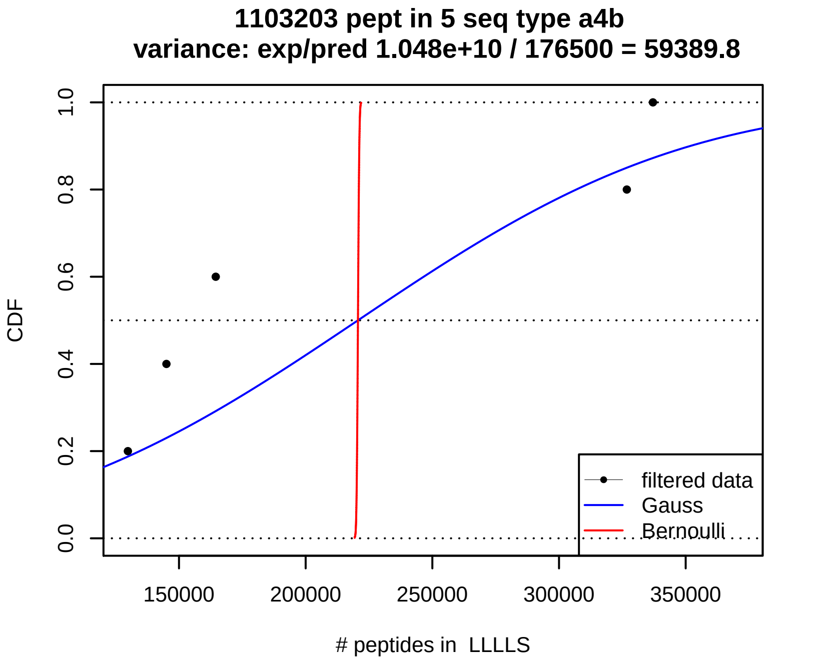
1103203 pept in 5 seq type a4b
variance: exp/pred 1.048e+10 / 176500 = 59389.8
CDF
0.0
0.2
0.4
0.6
0.8
1.0
150000
200000
250000
300000
350000
# peptides in LLLLS
filtered data
Gauss
Bernoulli

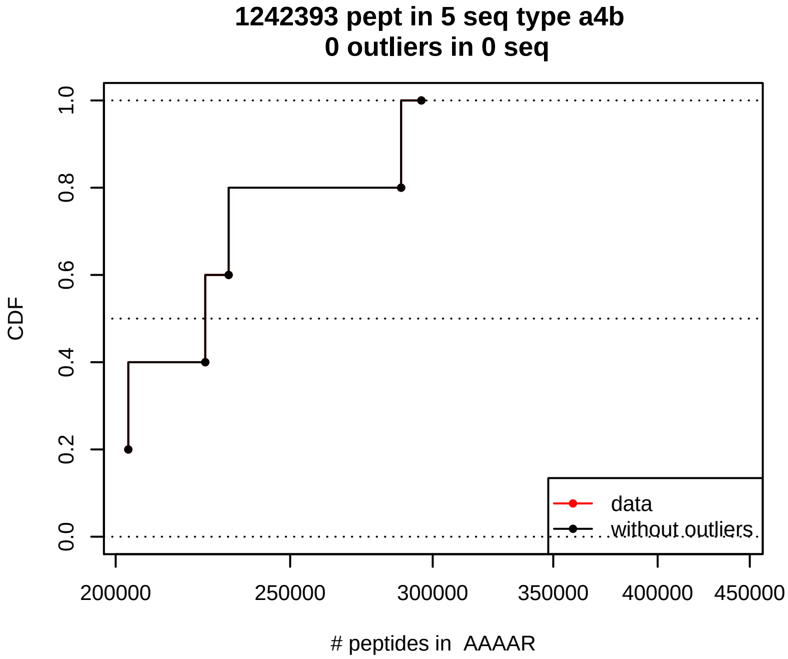
1242393 pept in 5 seq type a4b
0 outliers in 0 seq
CDF
0.0
0.2
0.4
0.6
0.8
1.0
200000
250000
300000
350000
400000
450000
# peptides in AAAAR
data
without outliers

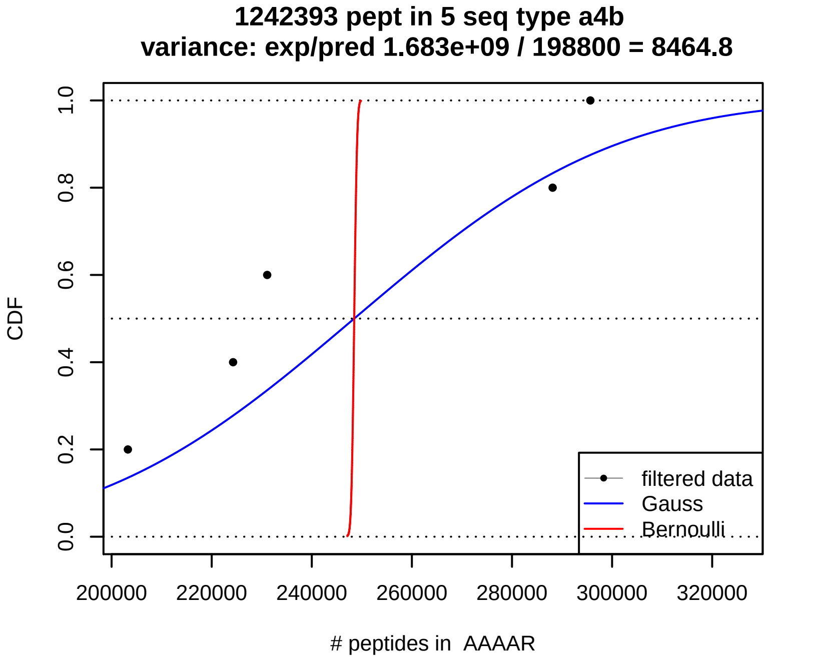
1242393 pept in 5 seq type a4b
variance: exp/pred 1.683e+09 / 198800 = 8464.8
CDF
0.0
0.2
0.4
0.6
0.8
1.0
200000
220000
240000
260000
280000
300000
320000
# peptides in AAAAR
filtered data
Gauss
Bernoulli

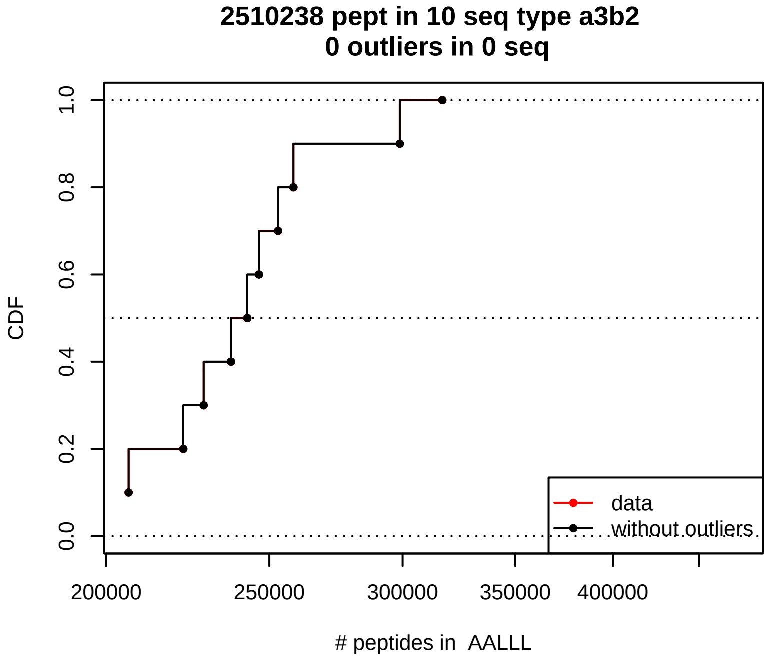
2510238 pept in 10 seq type a3b2
0 outliers in 0 seq
CDF
0.0
0.2
0.4
0.6
0.8
1.0
200000
250000
300000
350000
400000
# peptides in AALLL
data
without outliers

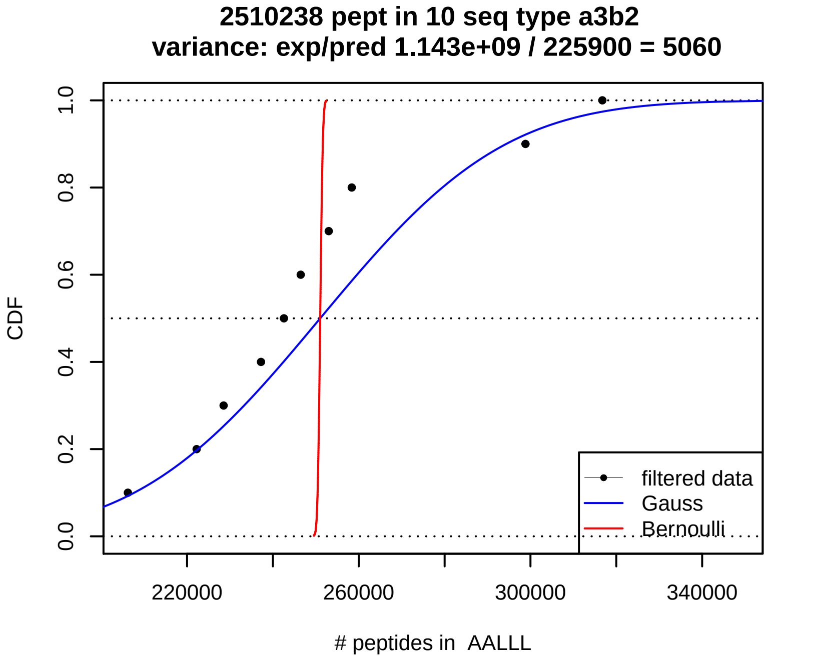
2510238 pept in 10 seq type a3b2
variance: exp/pred 1.143e+09 / 225900 = 5060
CDF
0.0
0.2
0.4
0.6
0.8
1.0
220000
260000
300000
340000
# peptides in AALLL
filtered data
Gauss
Bernoulli

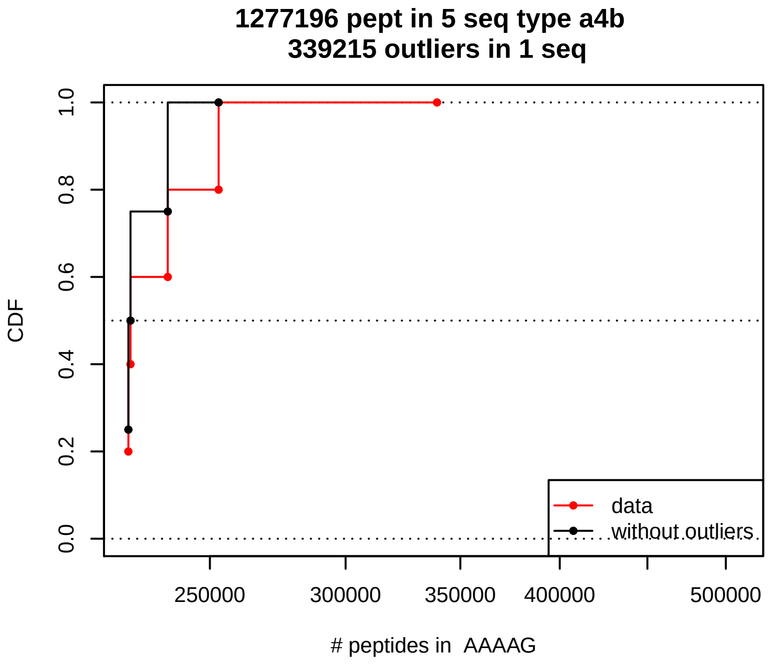
1277196 pept in 5 seq type a4b
339215 outliers in 1 seq
CDF
# peptides in AAAAG
data
without outliers

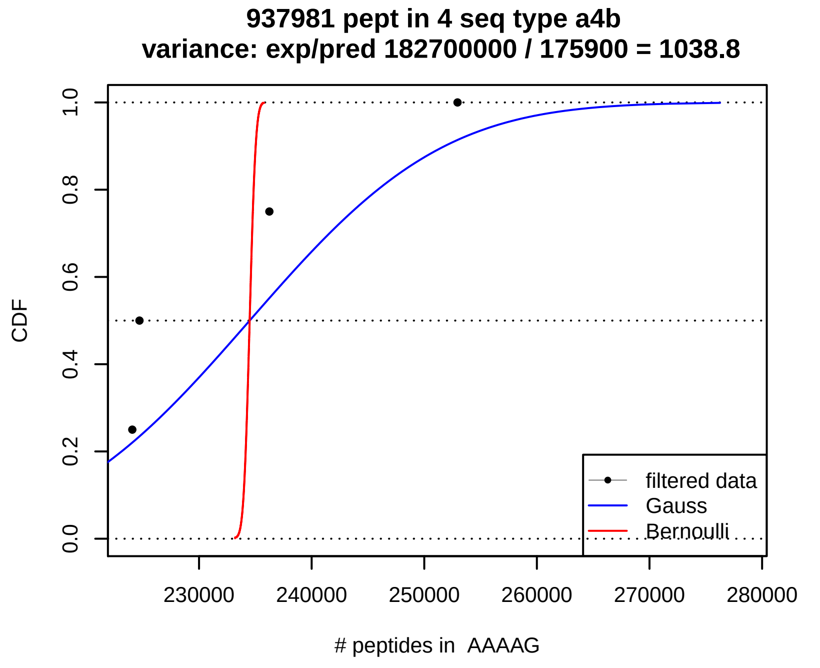
937981 pept in 4 seq type a4b
variance: exp/pred 182700000 / 175900 = 1038.8
CDF
# peptides in AAAAG
filtered data
Gauss
Bernoulli

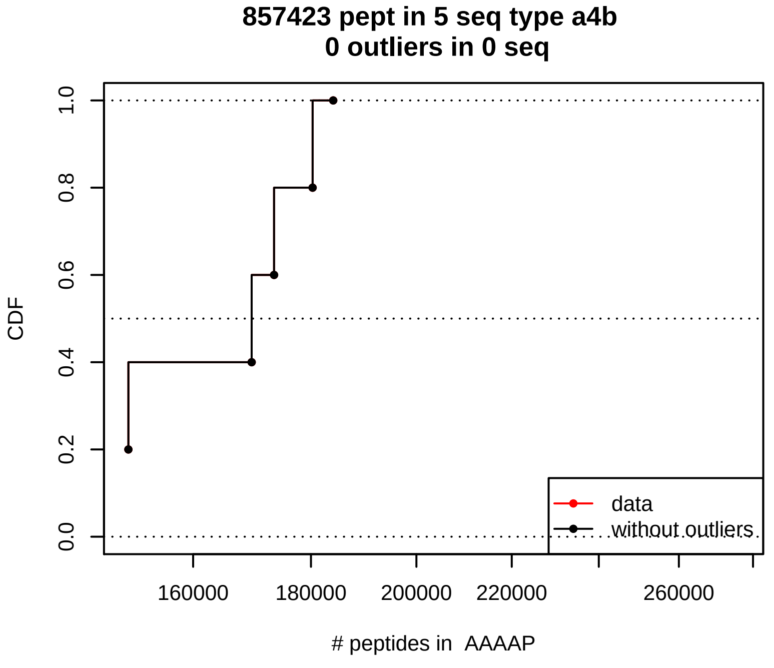
857423 pept in 5 seq type a4b
0 outliers in 0 seq
CDF
# peptides in AAAAP
data
without outliers

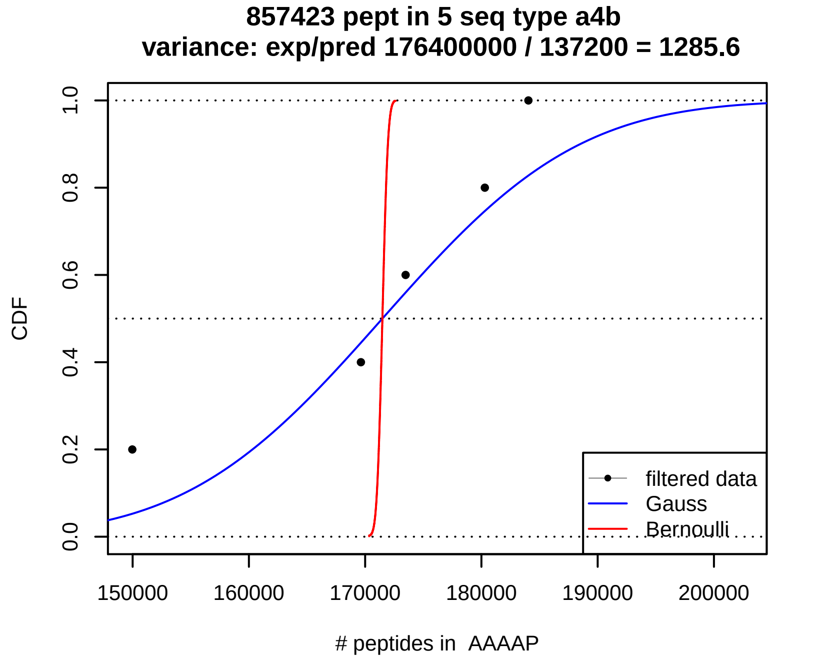
857423 pept in 5 seq type a4b
variance: exp/pred 176400000 / 137200 = 1285.6
CDF
# peptides in AAAAP
filtered data
Gauss
Bernoulli

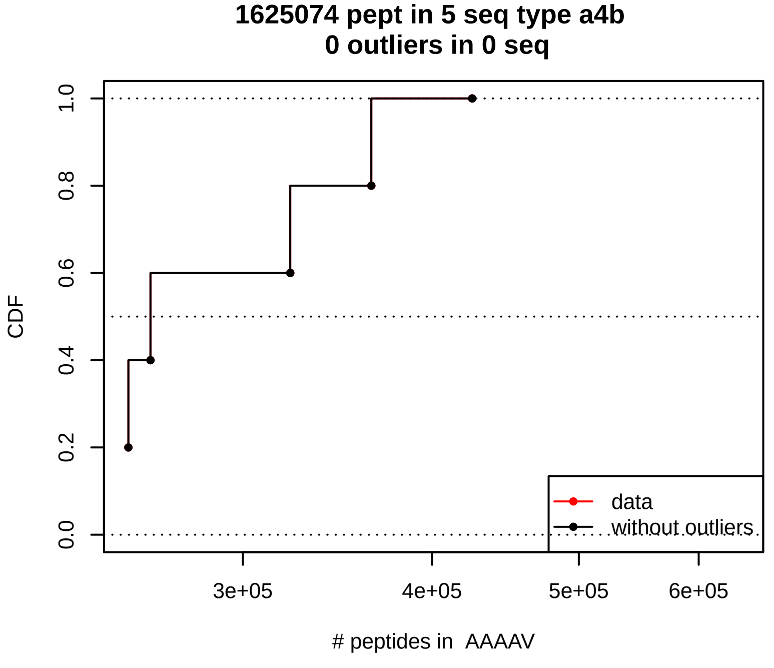
1625074 pept in 5 seq type a4b
0 outliers in 0 seq
CDF
# peptides in AAAAV
data
without outliers

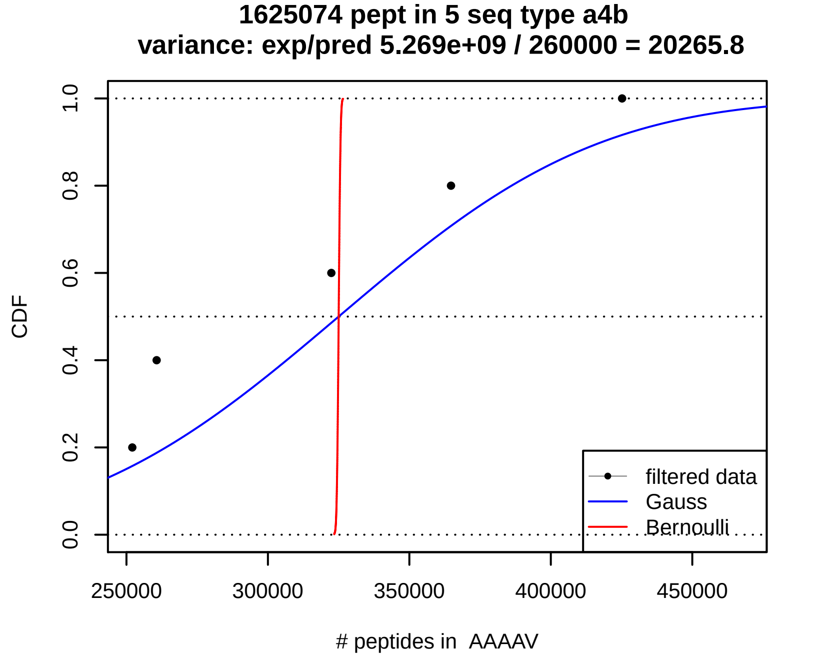
1625074 pept in 5 seq type a4b
variance: exp/pred 5.269e+09 / 260000 = 20265.8
CDF
# peptides in AAAAV
filtered data
Gauss
Bernoulli

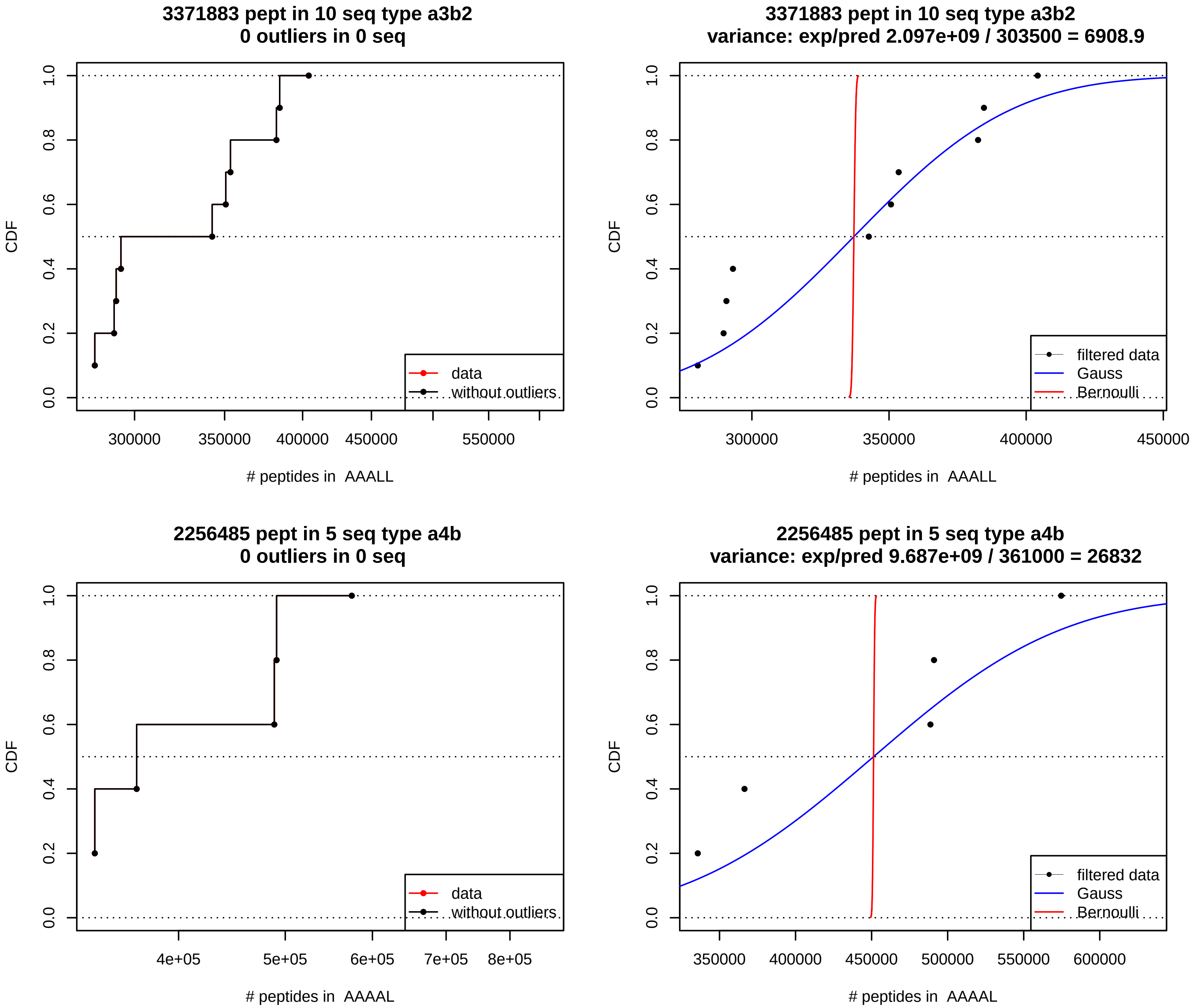


Suppl Fig. S1A
The CDFs of peptide occurrences for the representative permutation classes belonging to various pentapeptide categories are shown before (red circles) and after (black circles) outlier filtering (left panels). The distributions of peptide occurrences after outlier filtering compared with the Normal (Gauss) and the expected Binomial (Bernoulli) distributions are shown in right panels. Summary statistics concerning contribution of outliers and variance are shown above each panel. The outliers were analyzed at 0.05 (Fig S1A) or 0.001 (Fig S1B) significance level.

Suppl. Fig. S1B
legend on last page

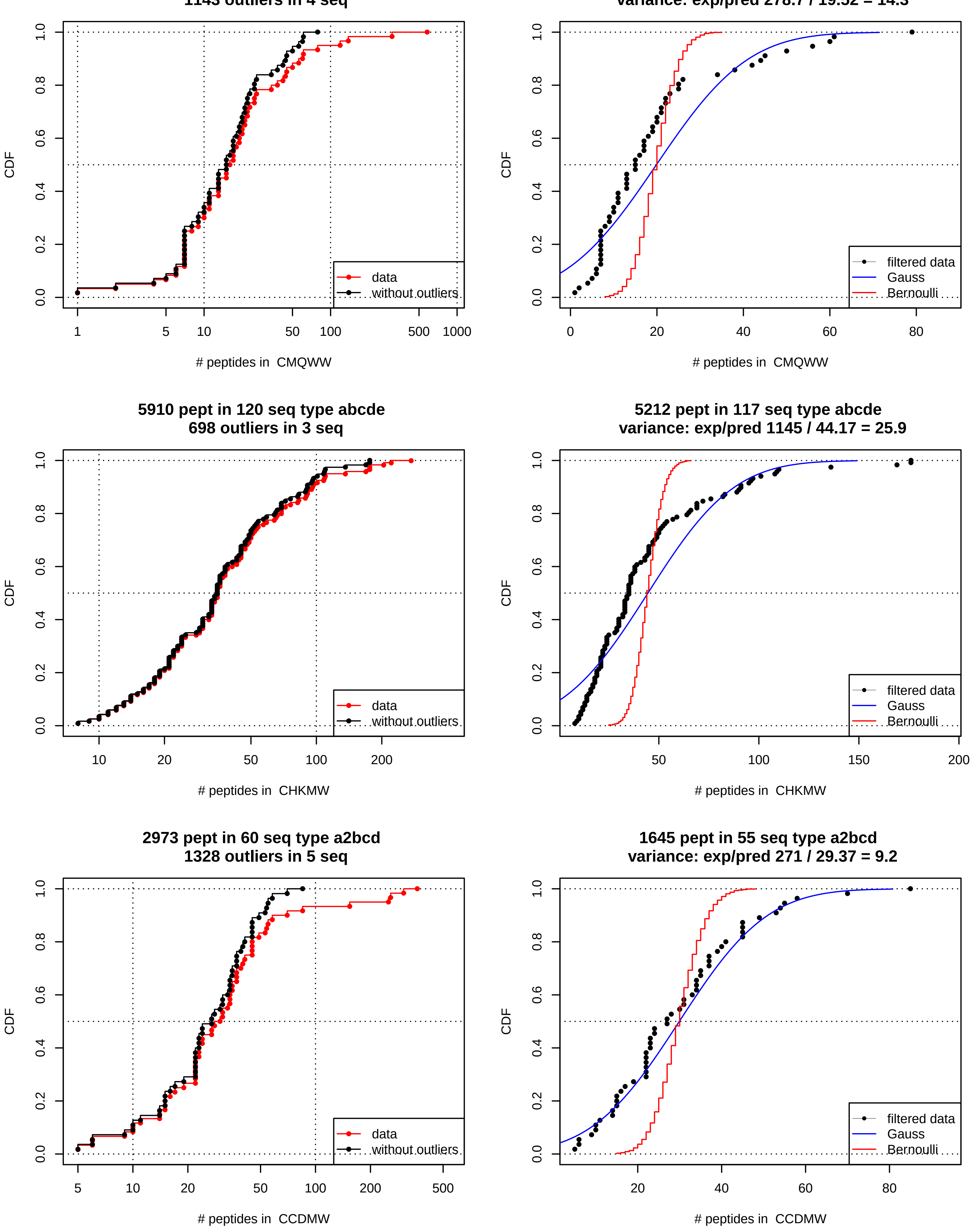

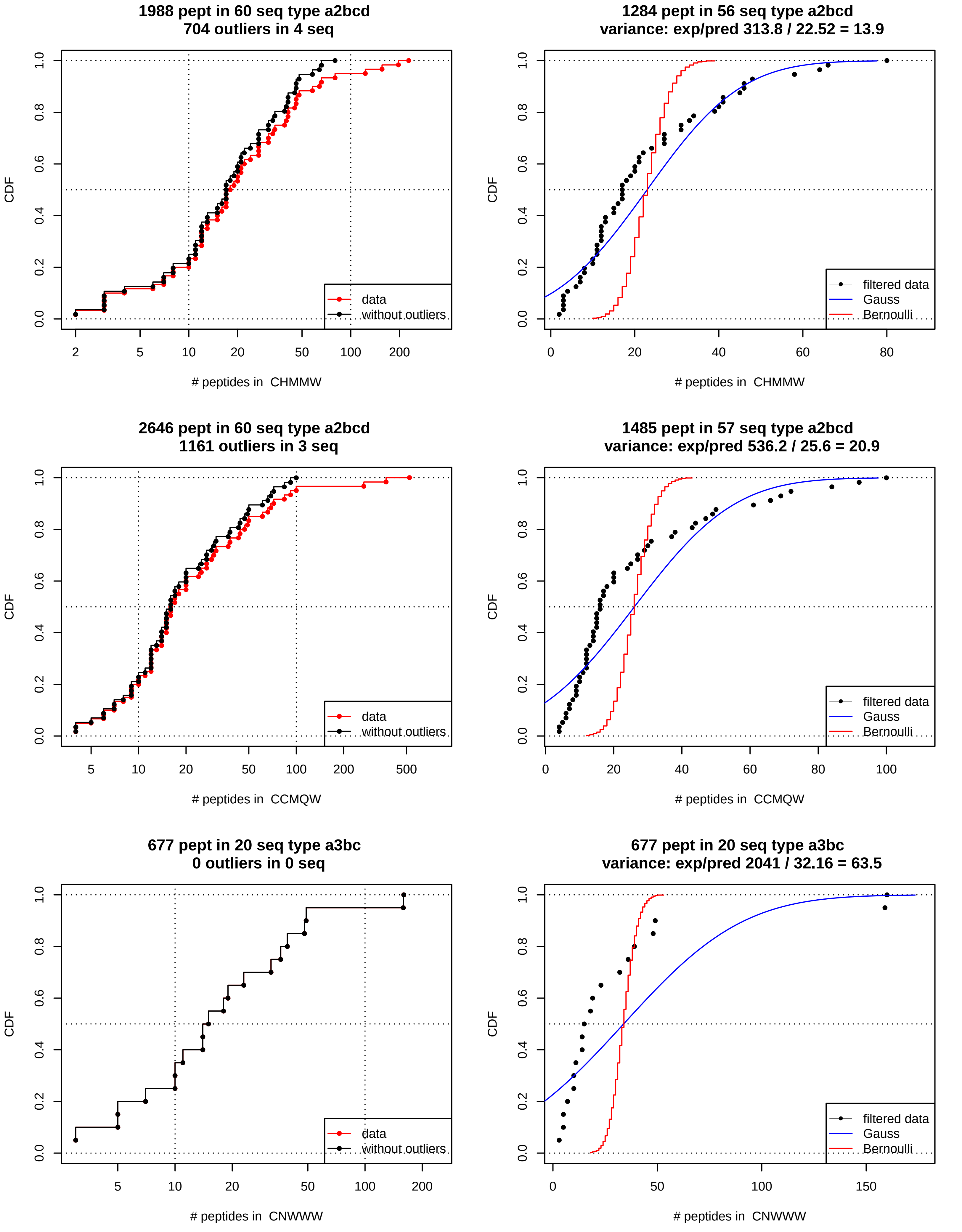

1988 pept in 60 seq type a2bcd
704 outliers in 4 seq
CDF
# peptides in CHMMW
data
without outliers
1284 pept in 56 seq type a2bcd
variance: exp/pred 313.8 / 22.52 = 13.9
CDF
# peptides in CHMMW
filtered data
Gauss
Bernoulli
2646 pept in 60 seq type a2bcd
1161 outliers in 3 seq
CDF
# peptides in CCMQW
data
without outliers
1485 pept in 57 seq type a2bcd
variance: exp/pred 536.2 / 25.6 = 20.9
CDF
# peptides in CCMQW
filtered data
Gauss
Bernoulli
677 pept in 20 seq type a3bc
0 outliers in 0 seq
CDF
# peptides in CNWWW
data
without outliers
677 pept in 20 seq type a3bc
variance: exp/pred 2041 / 32.16 = 63.5
CDF
# peptides in CNWWW
filtered data
Gauss
Bernoulli

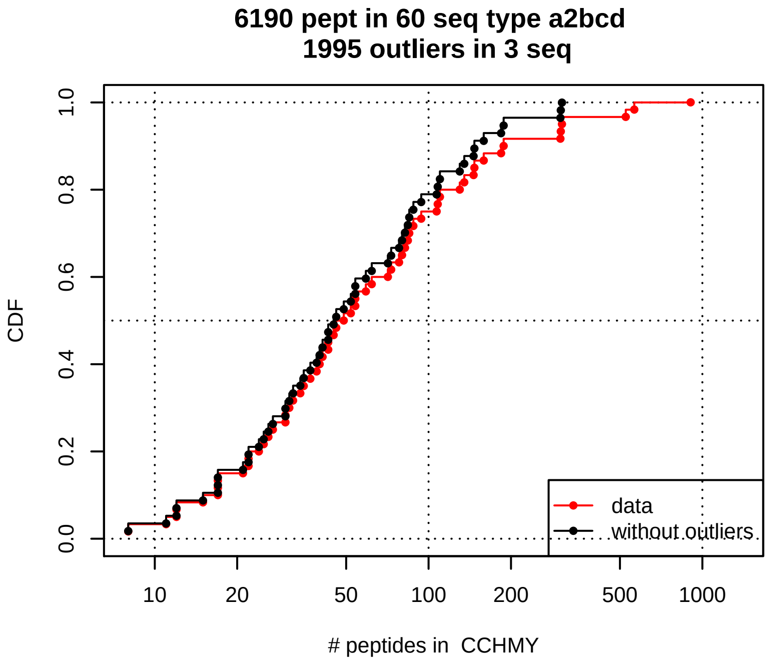
6190 pept in 60 seq type a2bcd
1995 outliers in 3 seq
CDF
# peptides in CCHMY
data
without outliers

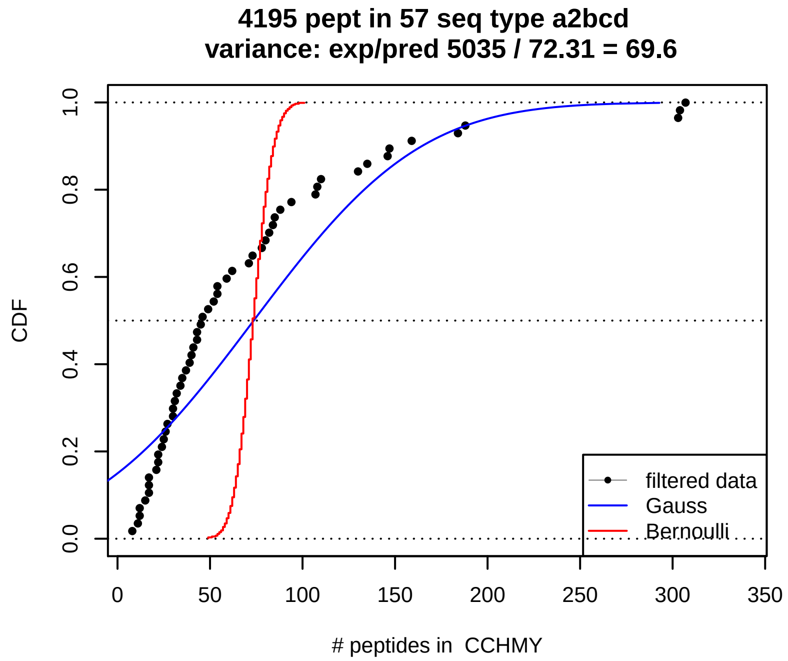
4195 pept in 57 seq type a2bcd
variance: exp/pred 5035 / 72.31 = 69.6
CDF
# peptides in CCHMY
filtered data
Gauss
Bernoulli

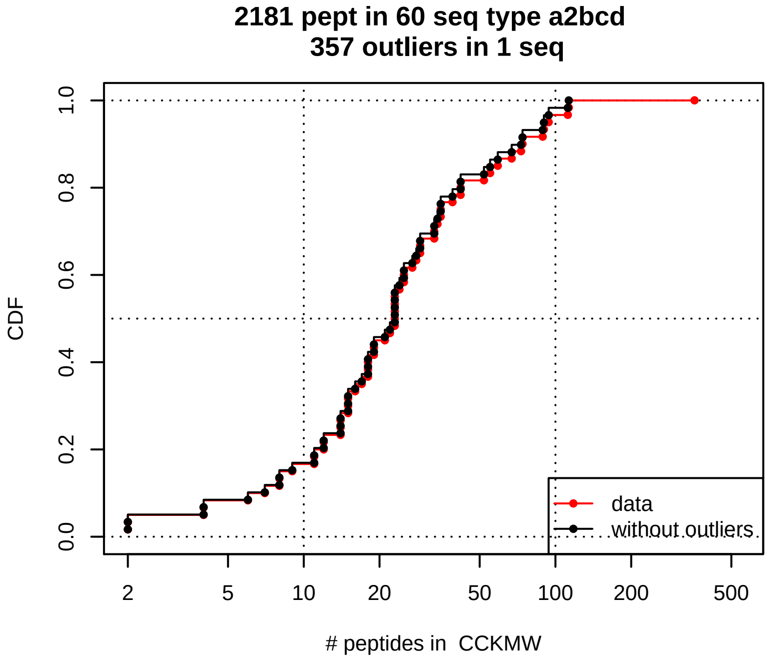
2181 pept in 60 seq type a2bcd
357 outliers in 1 seq
CDF
# peptides in CCKMW
data
without outliers

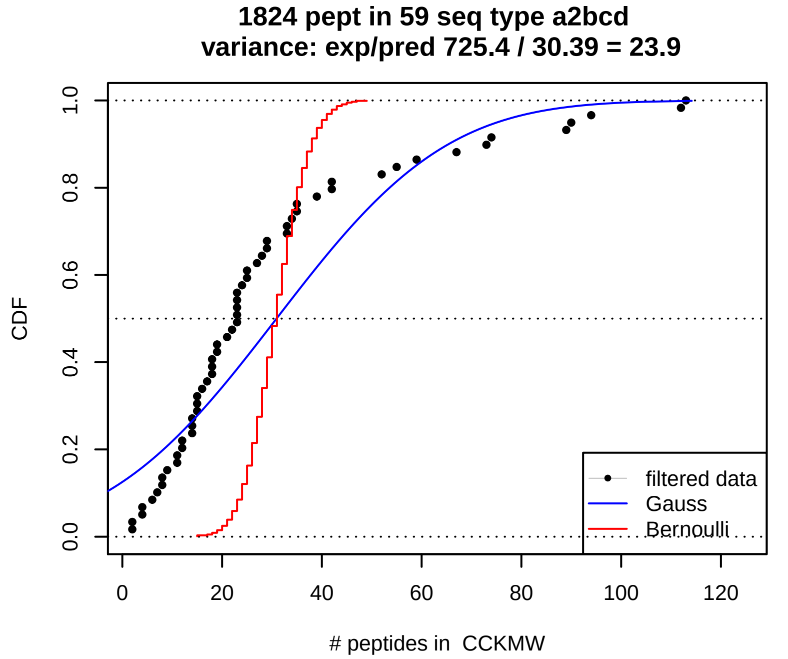
1824 pept in 59 seq type a2bcd
variance: exp/pred 725.4 / 30.39 = 23.9
CDF
# peptides in CCKMW
filtered data
Gauss
Bernoulli

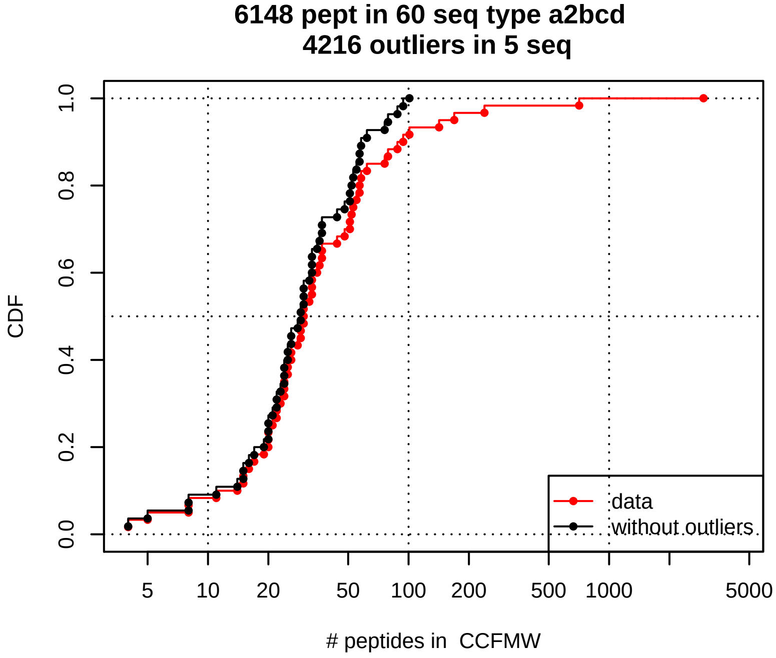
6148 pept in 60 seq type a2bcd
4216 outliers in 5 seq
CDF
# peptides in CCFMW
data
without outliers

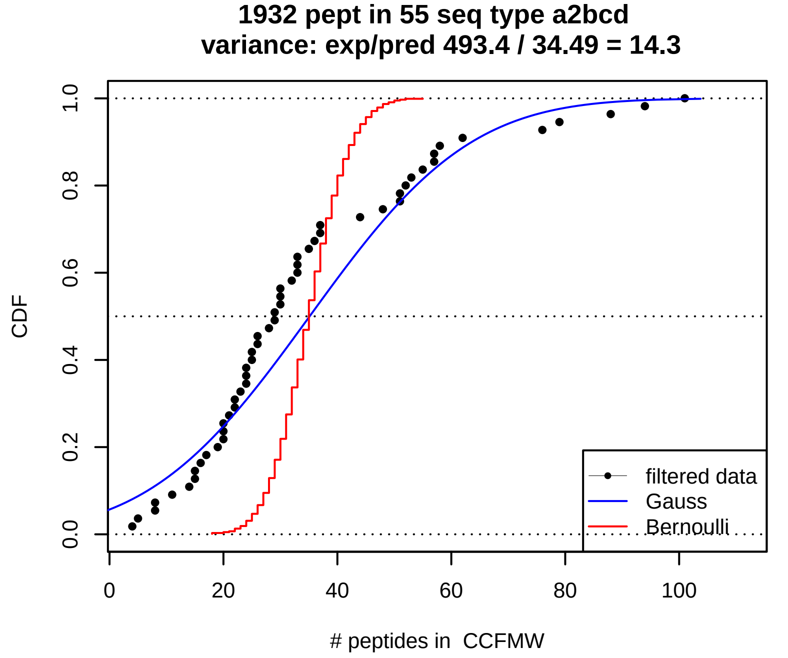
1932 pept in 55 seq type a2bcd
variance: exp/pred 493.4 / 34.49 = 14.3
CDF
# peptides in CCFMW
filtered data
Gauss
Bernoulli

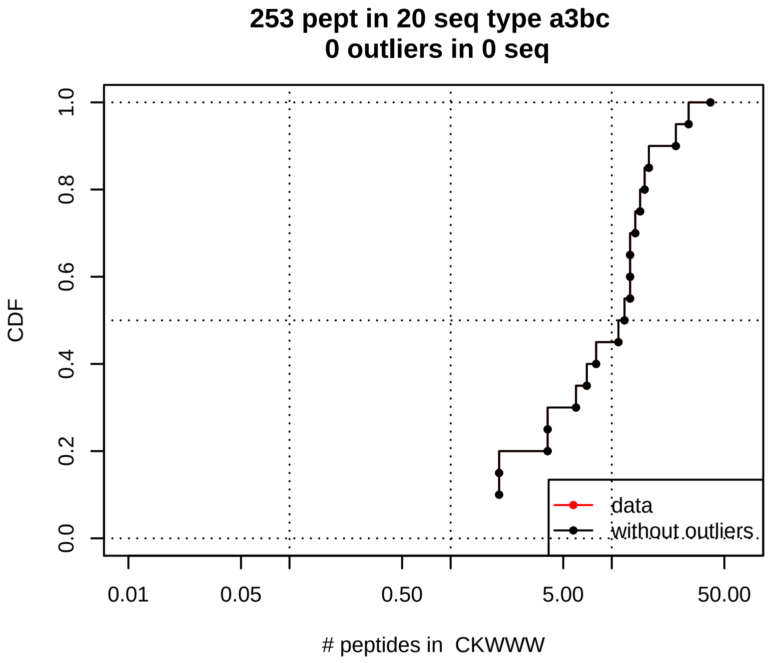
253 pept in 20 seq type a3bc
0 outliers in 0 seq
CDF
0.0
0.2
0.4
0.6
0.8
1.0
0.01
0.05
0.50
5.00
50.00
# peptides in CKWWW
data
without outliers

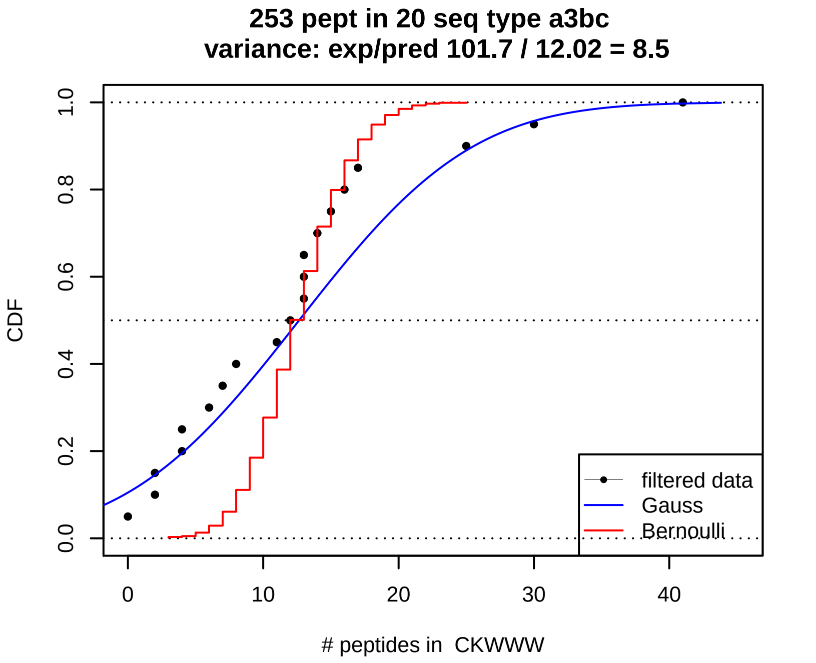
253 pept in 20 seq type a3bc
variance: exp/pred 101.7 / 12.02 = 8.5
CDF
0.0
0.2
0.4
0.6
0.8
1.0
0
10
20
30
40
# peptides in CKWWW
filtered data
Gauss
Bernoulli

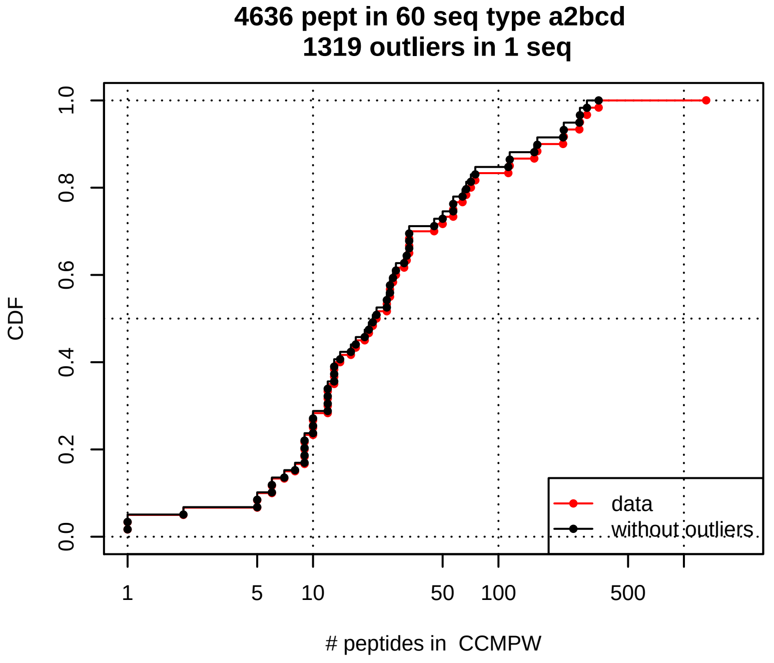
4636 pept in 60 seq type a2bcd
1319 outliers in 1 seq
CDF
0.0
0.2
0.4
0.6
0.8
1.0
1
5
10
50
100
500
# peptides in CCMPW
data
without outliers

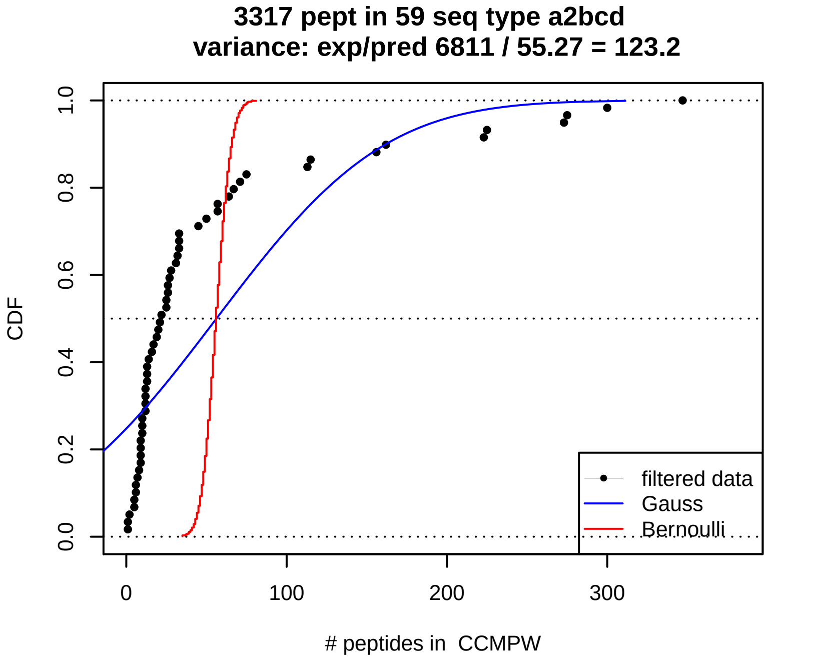
3317 pept in 59 seq type a2bcd
variance: exp/pred 6811 / 55.27 = 123.2
CDF
0.0
0.2
0.4
0.6
0.8
1.0
0
100
200
300
# peptides in CCMPW
filtered data
Gauss
Bernoulli

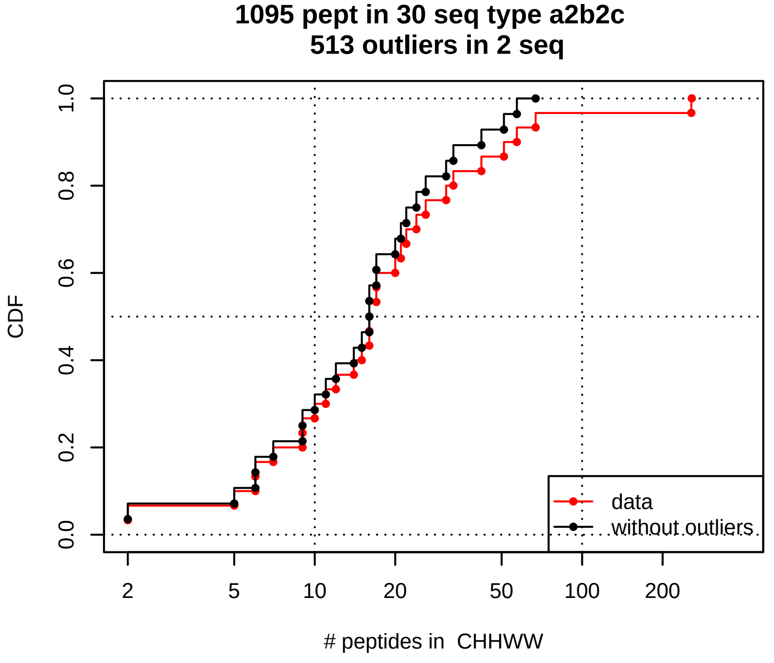
1095 pept in 30 seq type a2b2c
513 outliers in 2 seq
CDF
0.0
0.2
0.4
0.6
0.8
1.0
2
5
10
20
50
100
200
# peptides in CHHWW
data
without outliers

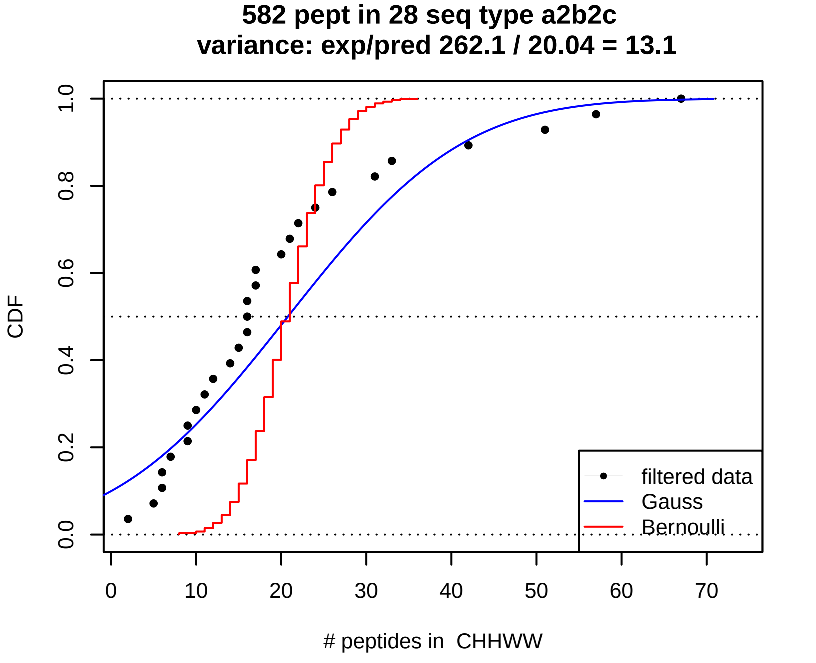
582 pept in 28 seq type a2b2c
variance: exp/pred 262.1 / 20.04 = 13.1
CDF
0.0
0.2
0.4
0.6
0.8
1.0
0
10
20
30
40
50
60
70
# peptides in CHHWW
filtered data
Gauss
Bernoulli

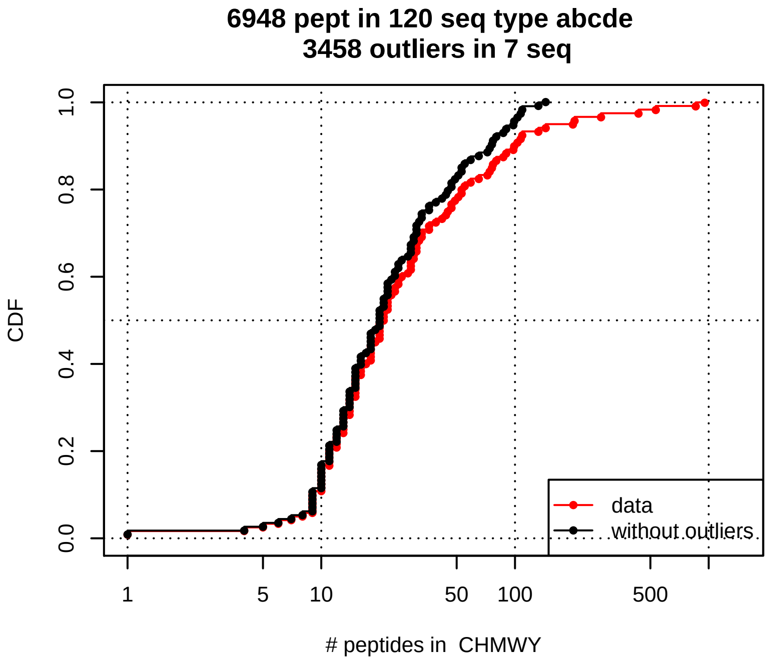
6948 pept in 120 seq type abcde
3458 outliers in 7 seq
CDF
# peptides in CHMWY
data
without outliers

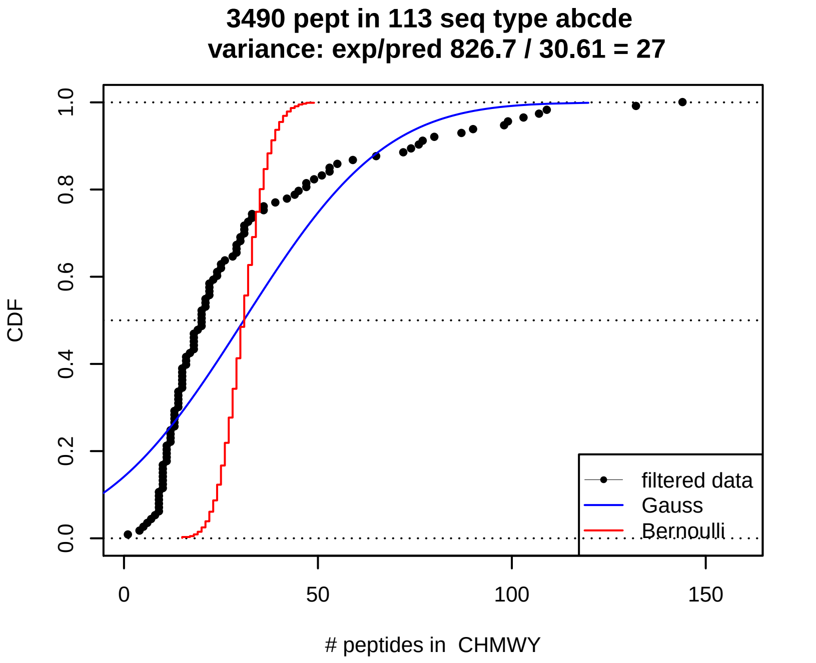
3490 pept in 113 seq type abcde
variance: exp/pred 826.7 / 30.61 = 27
CDF
# peptides in CHMWY
filtered data
Gauss
Bernoulli

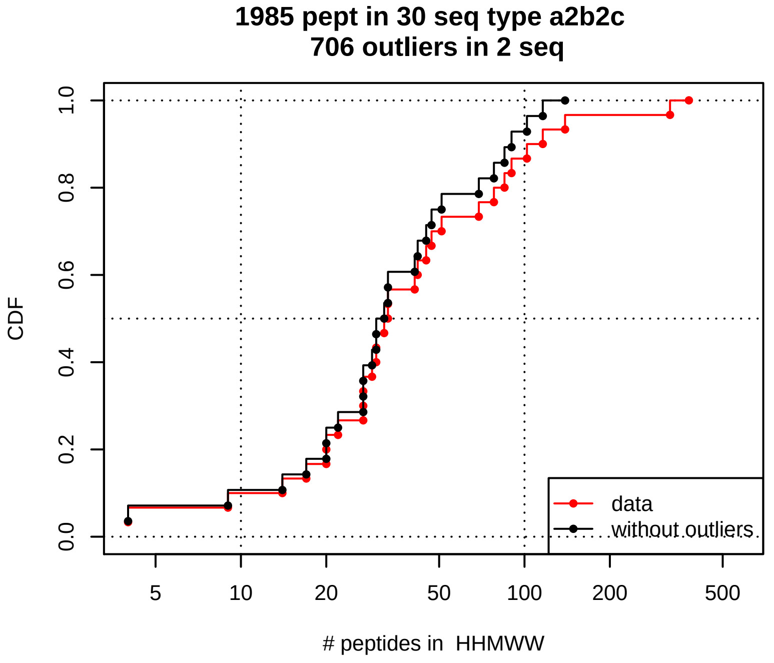
1985 pept in 30 seq type a2b2c
706 outliers in 2 seq
CDF
# peptides in HHMWW
data
without outliers

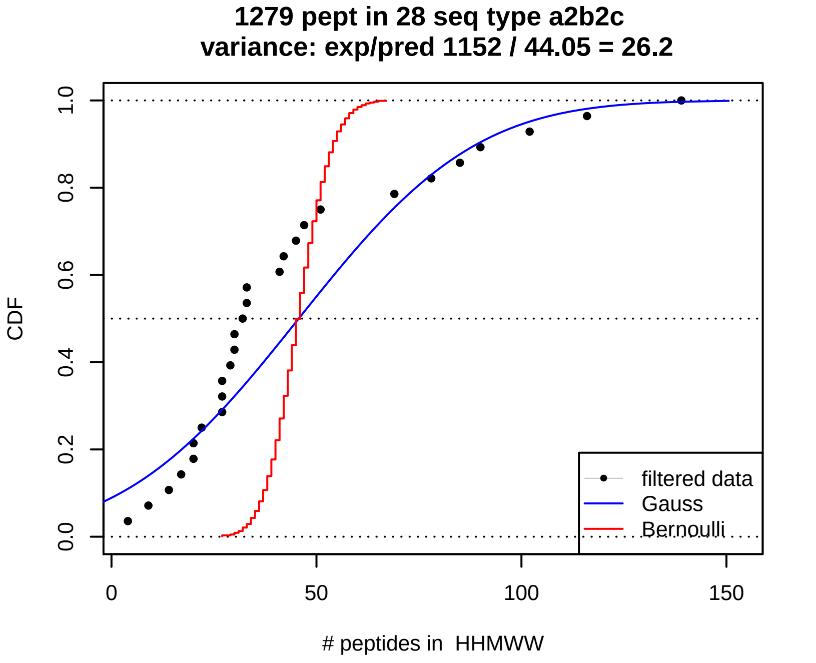
1279 pept in 28 seq type a2b2c
variance: exp/pred 1152 / 44.05 = 26.2
CDF
# peptides in HHMWW
filtered data
Gauss
Bernoulli

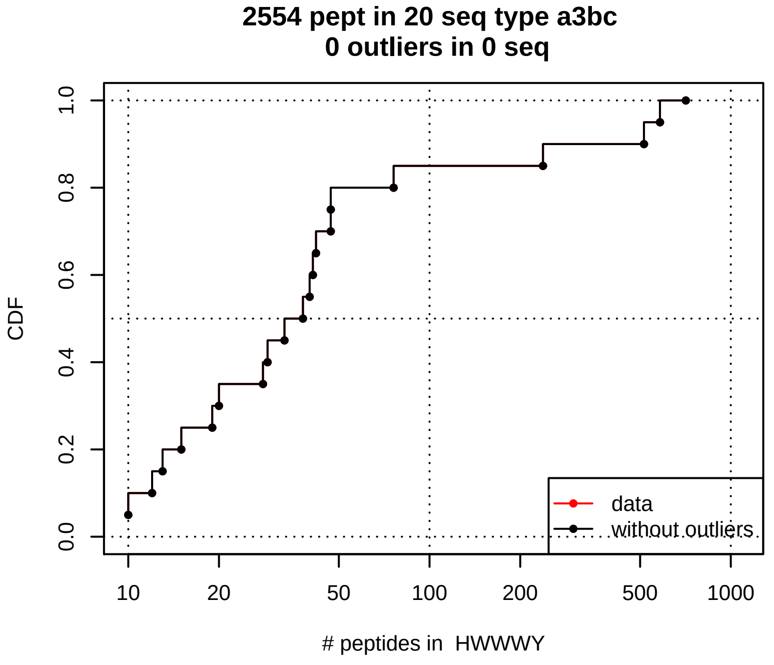
2554 pept in 20 seq type a3bc
0 outliers in 0 seq
CDF
# peptides in HWWWY
data
without outliers

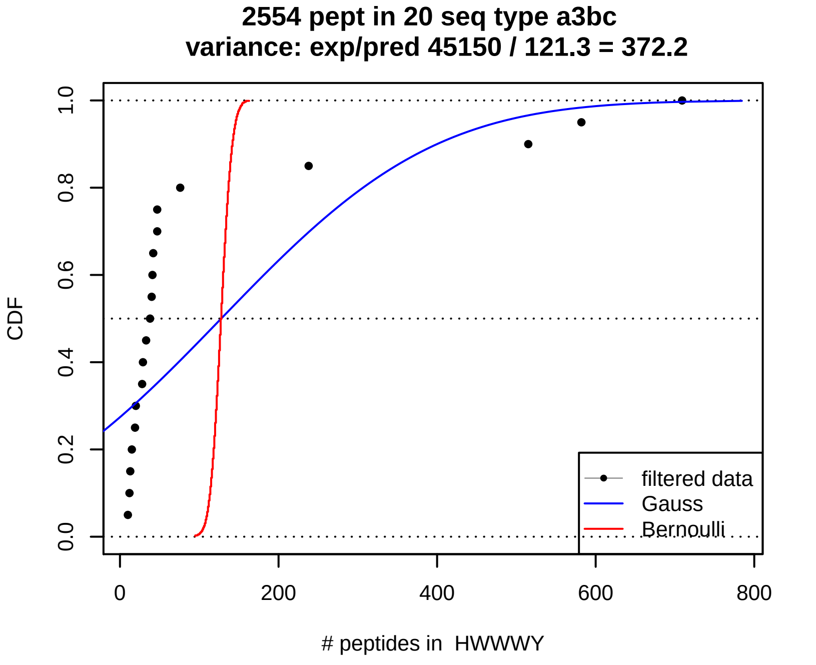
2554 pept in 20 seq type a3bc
variance: exp/pred 45150 / 121.3 = 372.2
CDF
# peptides in HWWWY
filtered data
Gauss
Bernoulli

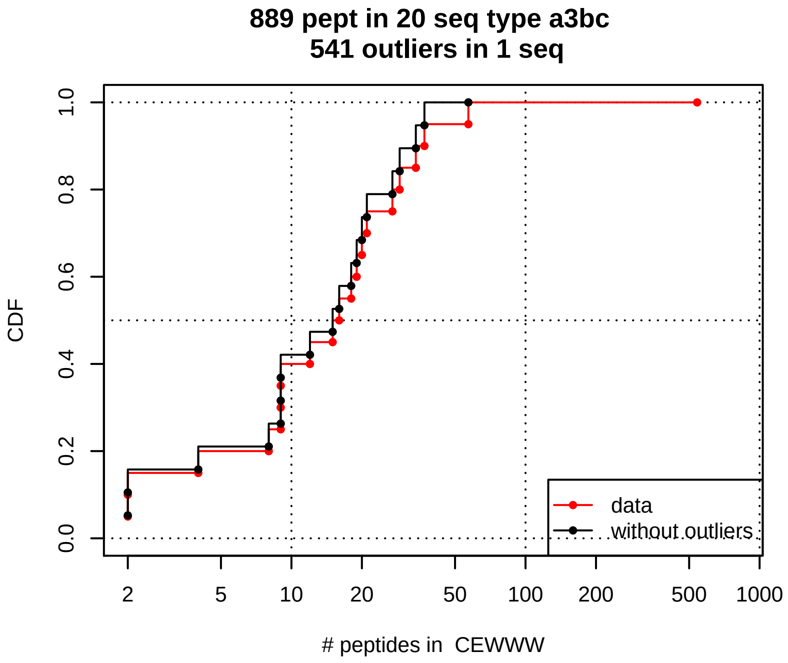
889 pept in 20 seq type a3bc
541 outliers in 1 seq
CDF
# peptides in CEWWW
data
without outliers

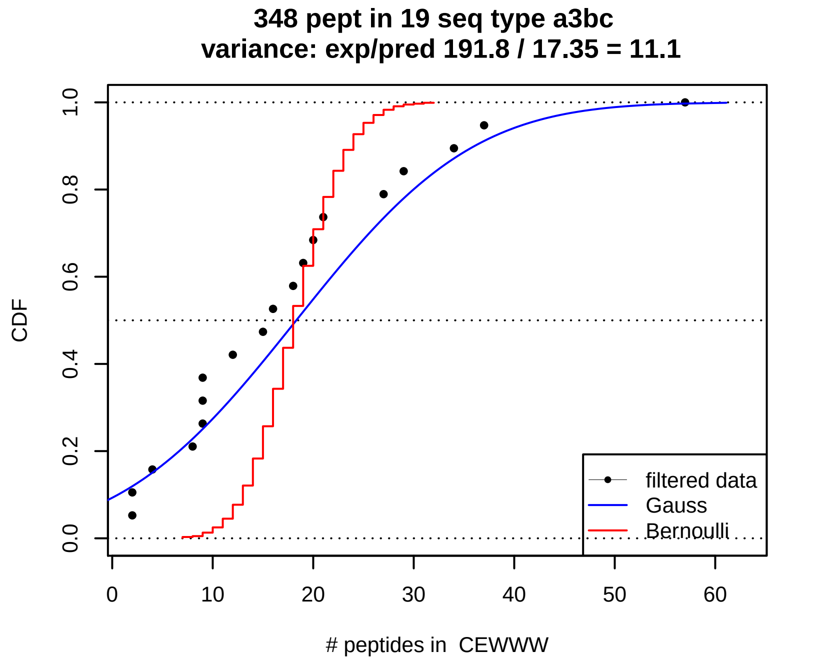
348 pept in 19 seq type a3bc
variance: exp/pred 191.8 / 17.35 = 11.1
CDF
# peptides in CEWWW
filtered data
Gauss
Bernoulli

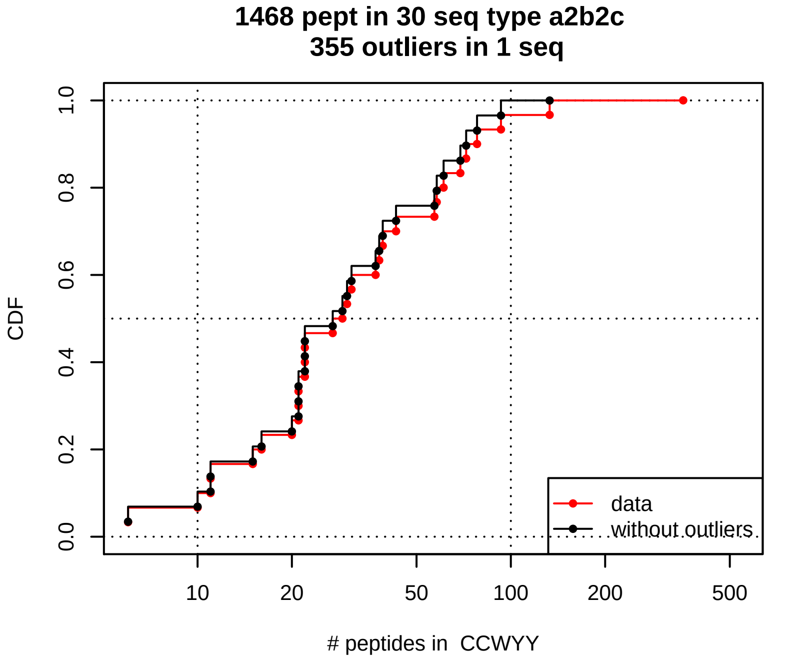
1468 pept in 30 seq type a2b2c
355 outliers in 1 seq
CDF
# peptides in CCWYY
data
without outliers

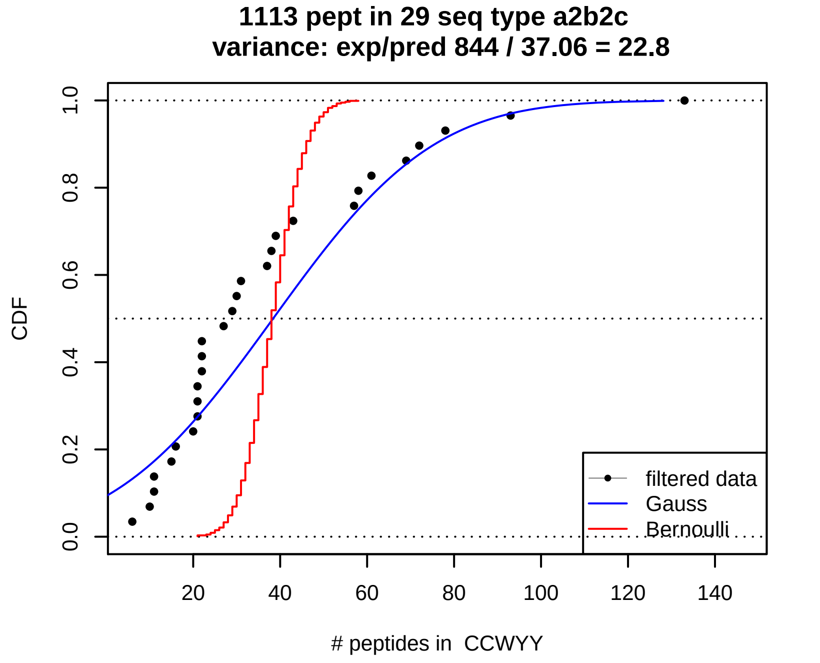
1113 pept in 29 seq type a2b2c
variance: exp/pred 844 / 37.06 = 22.8
CDF
# peptides in CCWYY
filtered data
Gauss
Bernoulli

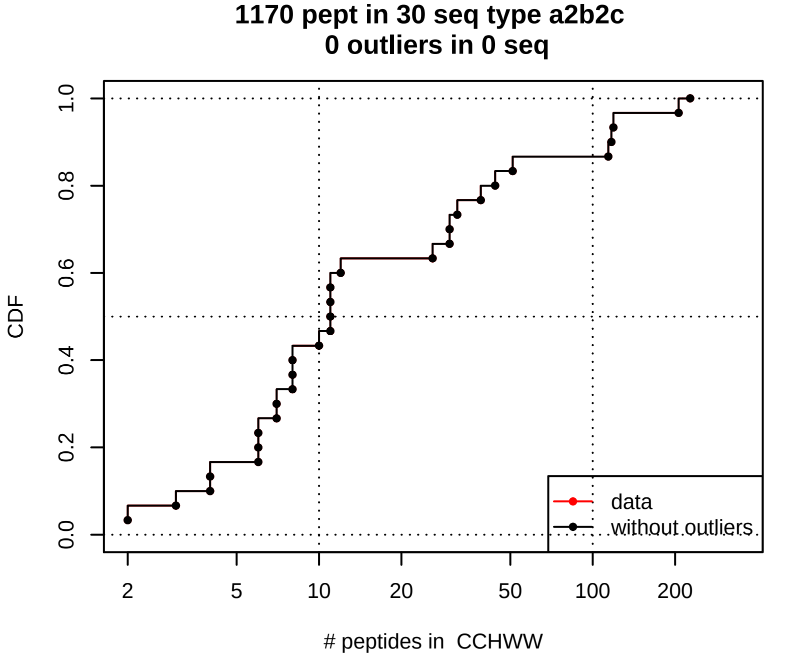
1170 pept in 30 seq type a2b2c
0 outliers in 0 seq
CDF
# peptides in CCHWW
data
without outliers

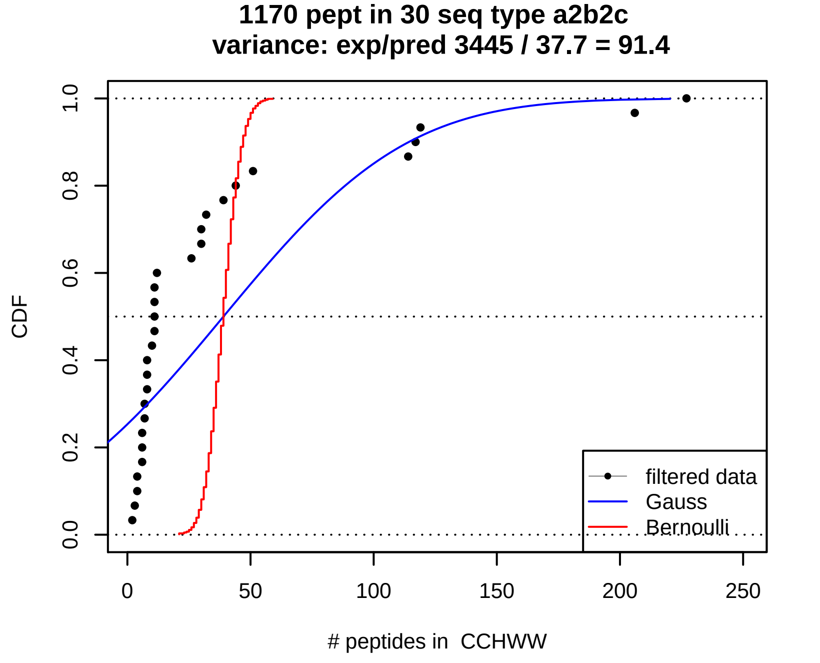
1170 pept in 30 seq type a2b2c
variance: exp/pred 3445 / 37.7 = 91.4
CDF
# peptides in CCHWW
filtered data
Gauss
Bernoulli

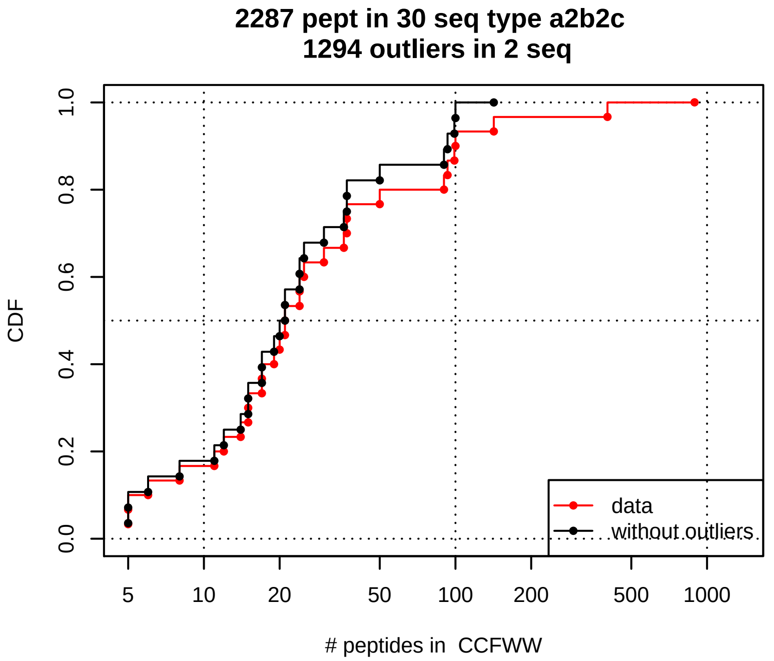
2287 pept in 30 seq type a2b2c
1294 outliers in 2 seq
CDF
# peptides in CCFWW
data
without outliers

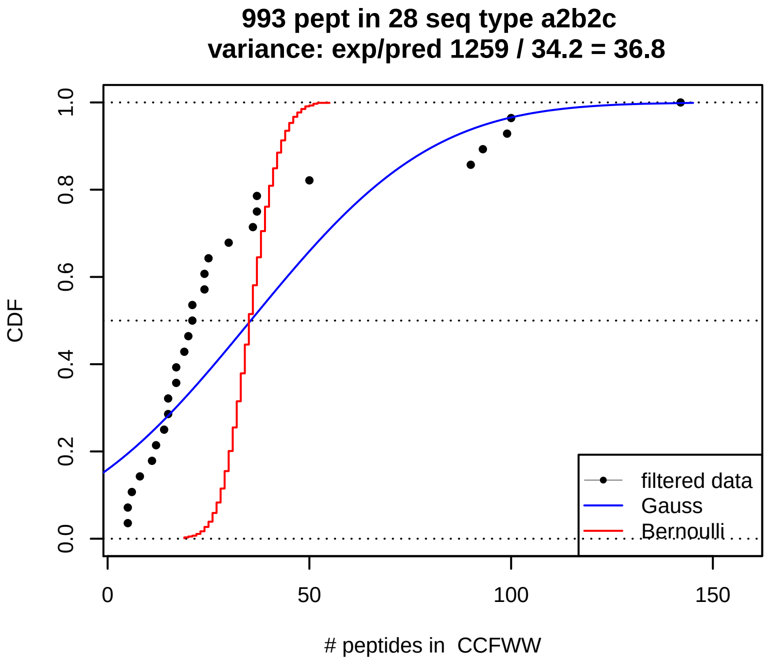
993 pept in 28 seq type a2b2c
variance: exp/pred 1259 / 34.2 = 36.8
CDF
# peptides in CCFWW
filtered data
Gauss
Bernoulli

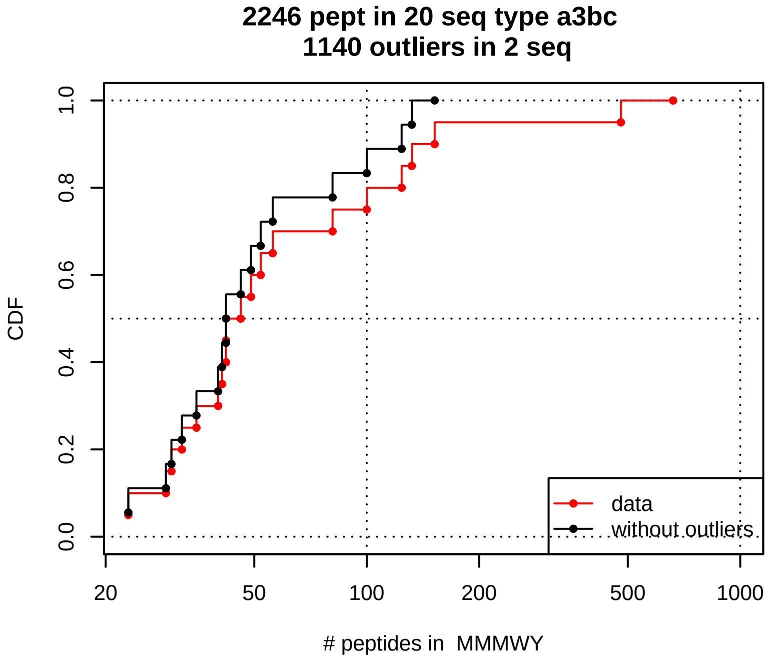
2246 pept in 20 seq type a3bc
1140 outliers in 2 seq
CDF
# peptides in MMMWY
data
without outliers

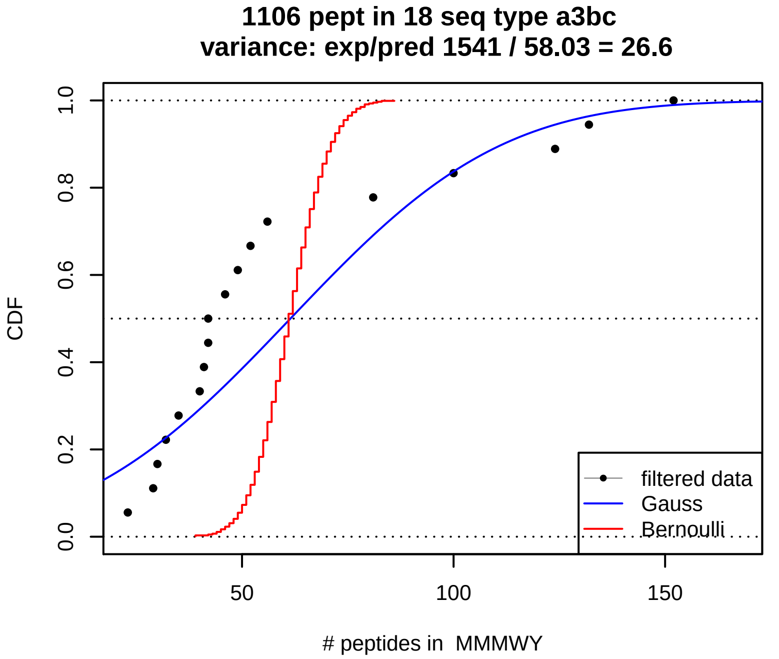
1106 pept in 18 seq type a3bc
variance: exp/pred 1541 / 58.03 = 26.6
CDF
# peptides in MMMWY
filtered data
Gauss
Bernoulli

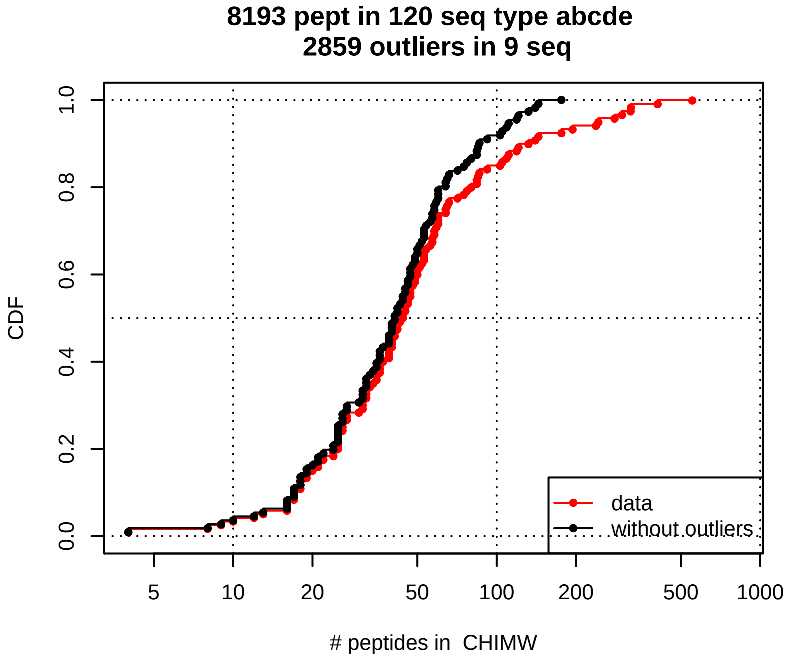
8193 pept in 120 seq type abcde
2859 outliers in 9 seq
CDF
# peptides in CHIMW
data
without outliers

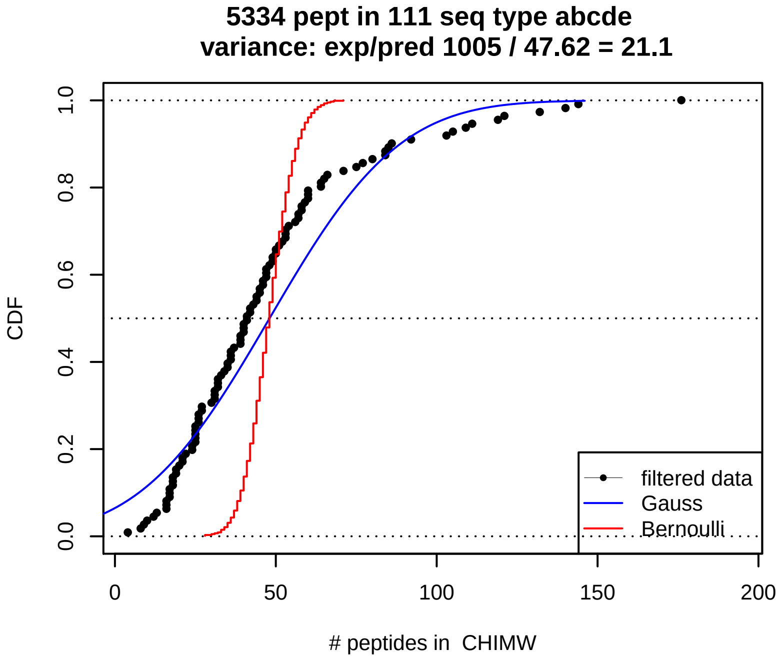
5334 pept in 111 seq type abcde
variance: exp/pred 1005 / 47.62 = 21.1
CDF
# peptides in CHIMW
filtered data
Gauss
Bernoulli

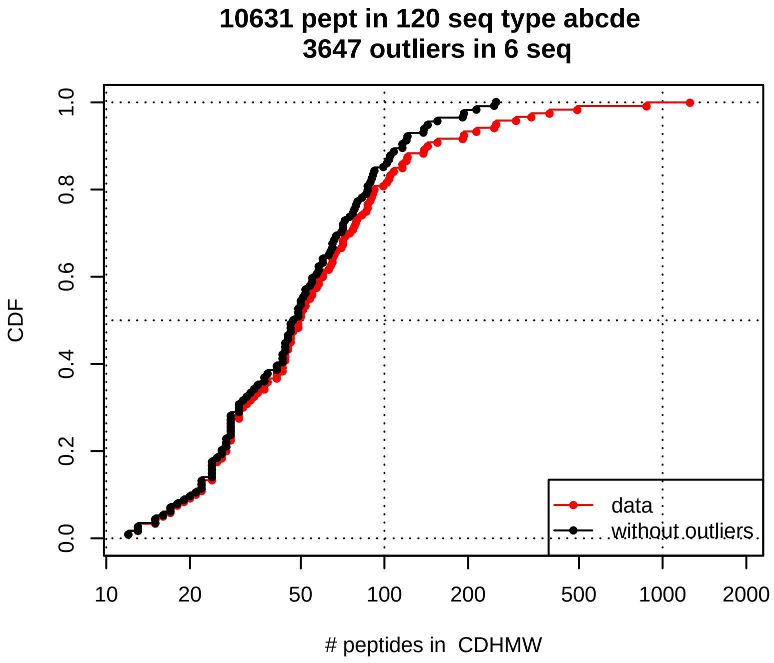
10631 pept in 120 seq type abcde
3647 outliers in 6 seq
CDF
# peptides in CDHMW
data
without outliers

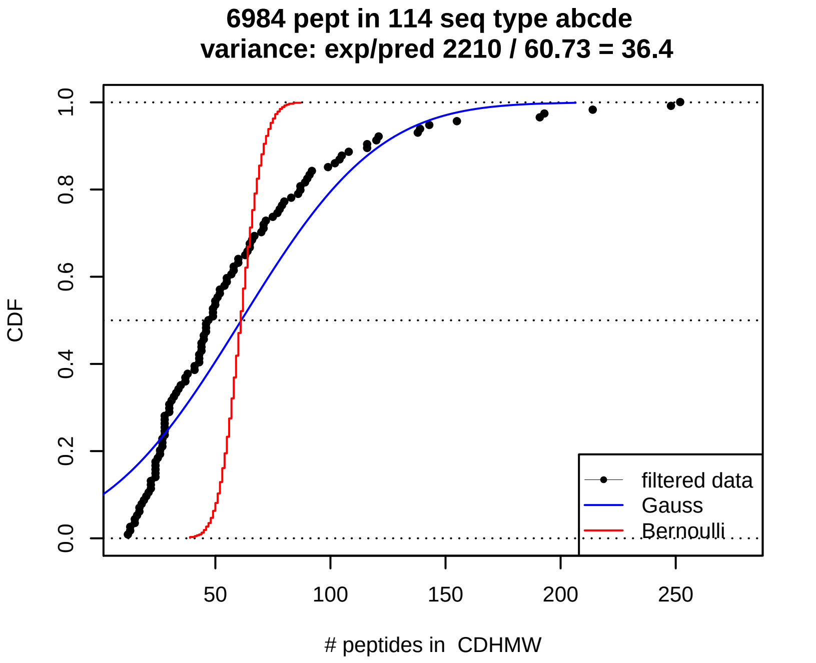
6984 pept in 114 seq type abcde
variance: exp/pred 2210 / 60.73 = 36.4
CDF
# peptides in CDHMW
filtered data
Gauss
Bernoulli

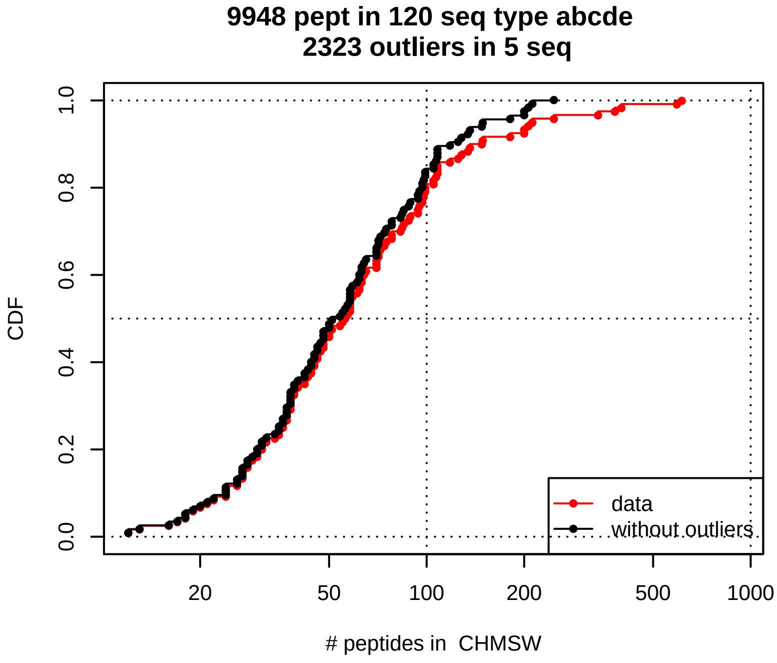
9948 pept in 120 seq type abcde
2323 outliers in 5 seq
CDF
# peptides in CHMSW
data
without outliers

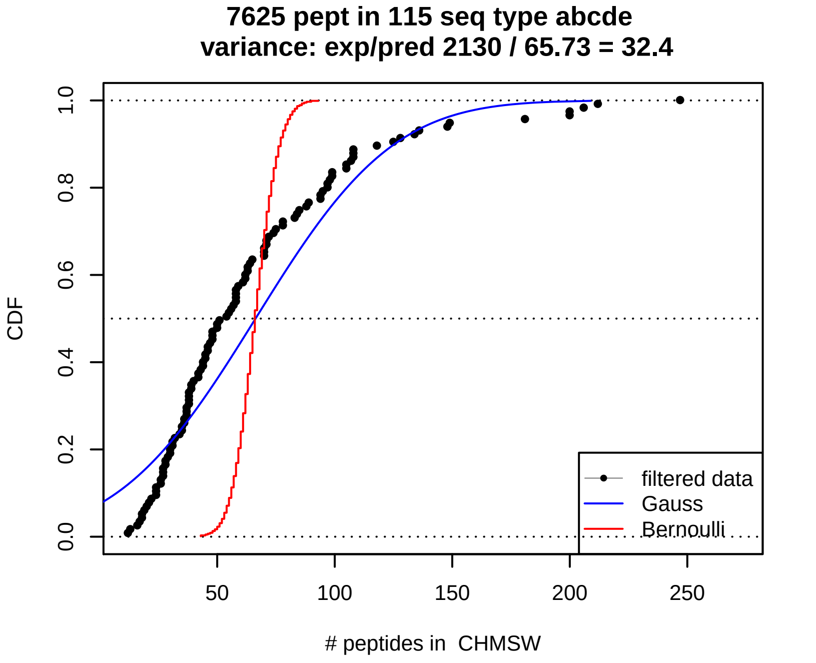
7625 pept in 115 seq type abcde
variance: exp/pred 2130 / 65.73 = 32.4
CDF
# peptides in CHMSW
filtered data
Gauss
Bernoulli

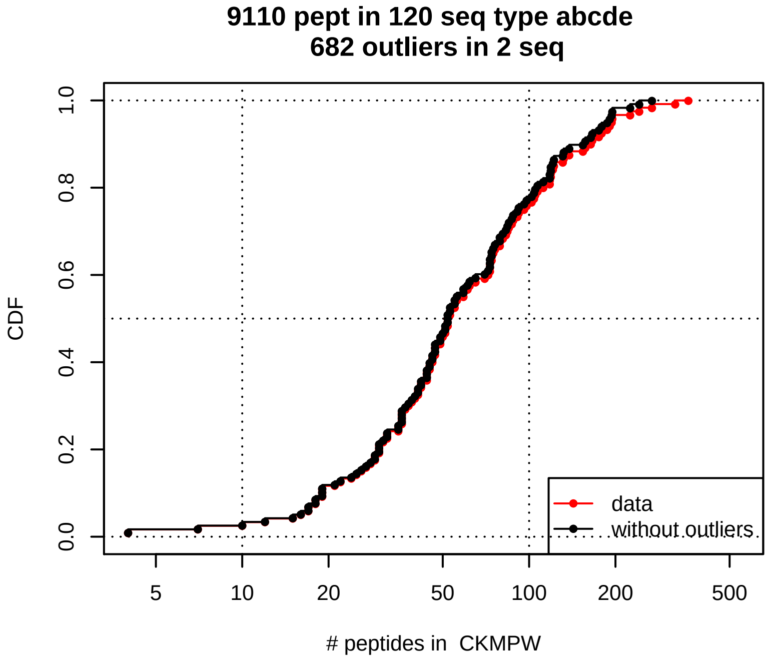
9110 pept in 120 seq type abcde
682 outliers in 2 seq
CDF
# peptides in CKMPW
data
without outliers

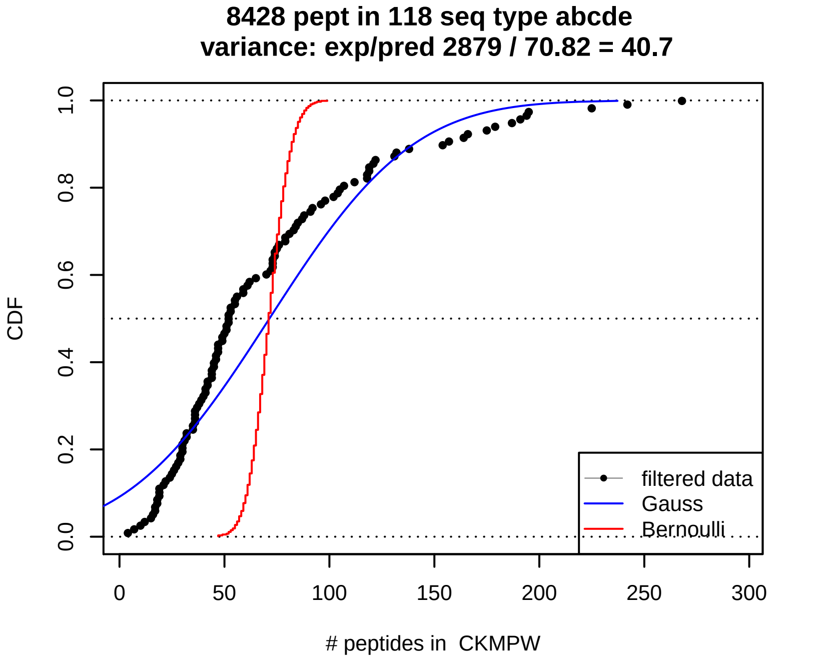
8428 pept in 118 seq type abcde
variance: exp/pred 2879 / 70.82 = 40.7
CDF
# peptides in CKMPW
filtered data
Gauss
Bernoulli

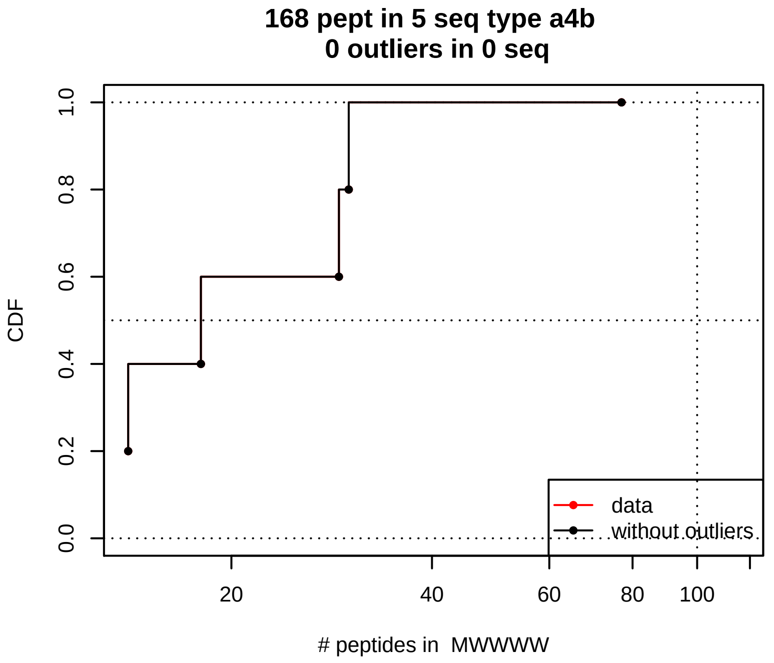
168 pept in 5 seq type a4b
0 outliers in 0 seq
CDF
# peptides in MWWWW
data
without outliers

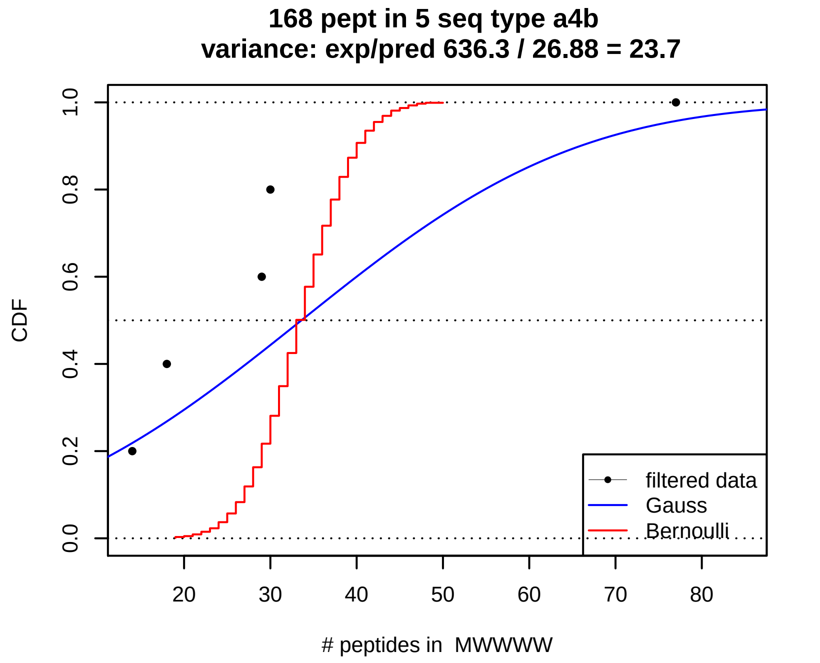
168 pept in 5 seq type a4b
variance: exp/pred 636.3 / 26.88 = 23.7
CDF
# peptides in MWWWW
filtered data
Gauss
Bernoulli

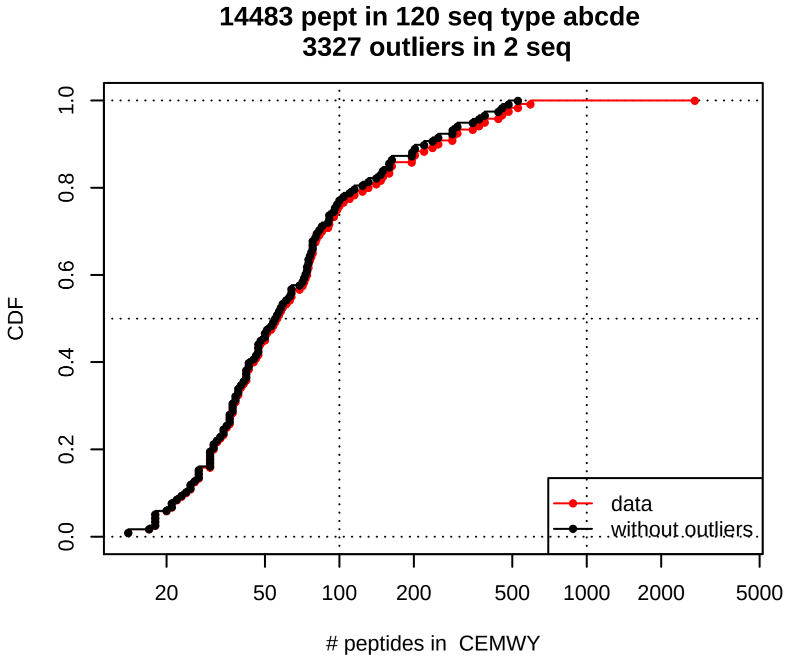
14483 pept in 120 seq type abcde
3327 outliers in 2 seq
CDF
# peptides in CEMWY
data
without outliers

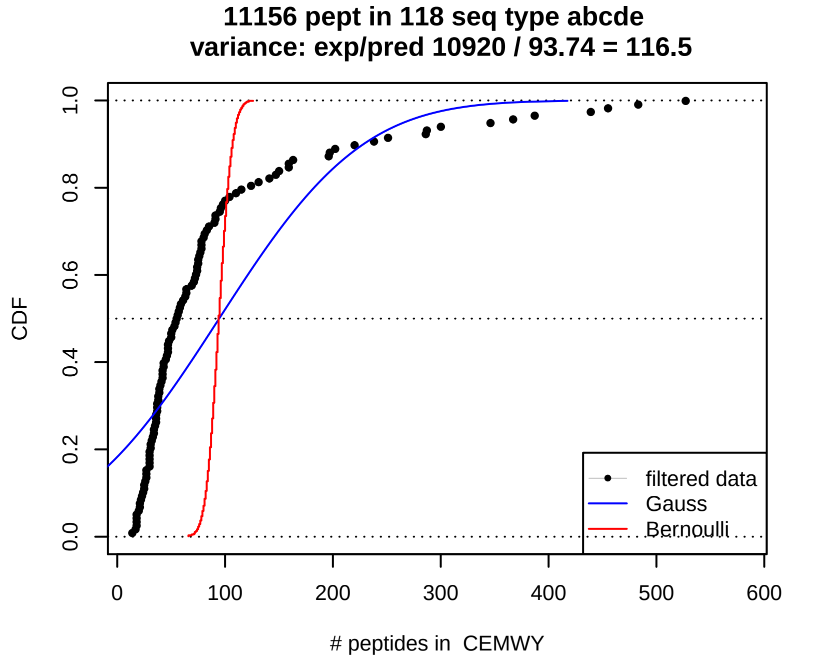
11156 pept in 118 seq type abcde
variance: exp/pred 10920 / 93.74 = 116.5
CDF
# peptides in CEMWY
filtered data
Gauss
Bernoulli

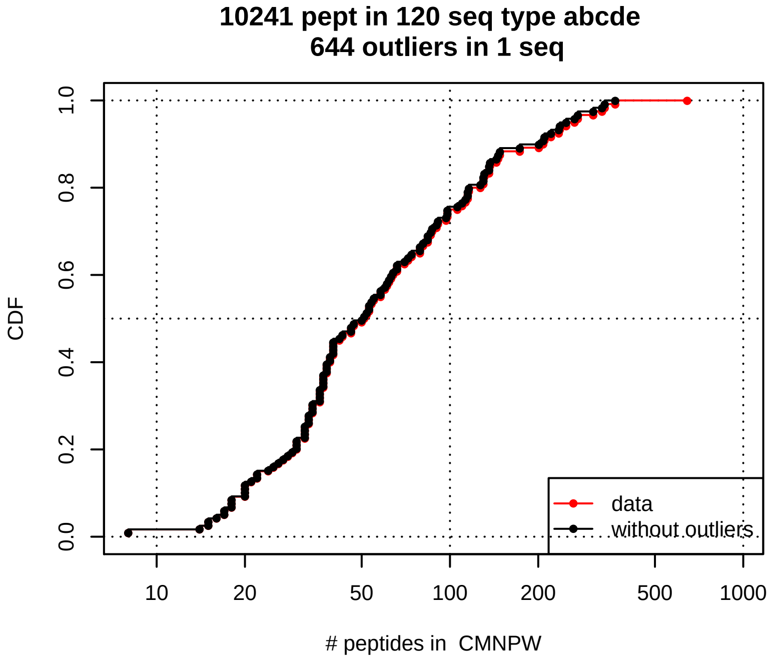
10241 pept in 120 seq type abcde
644 outliers in 1 seq
CDF
# peptides in CMNPW
data
without outliers

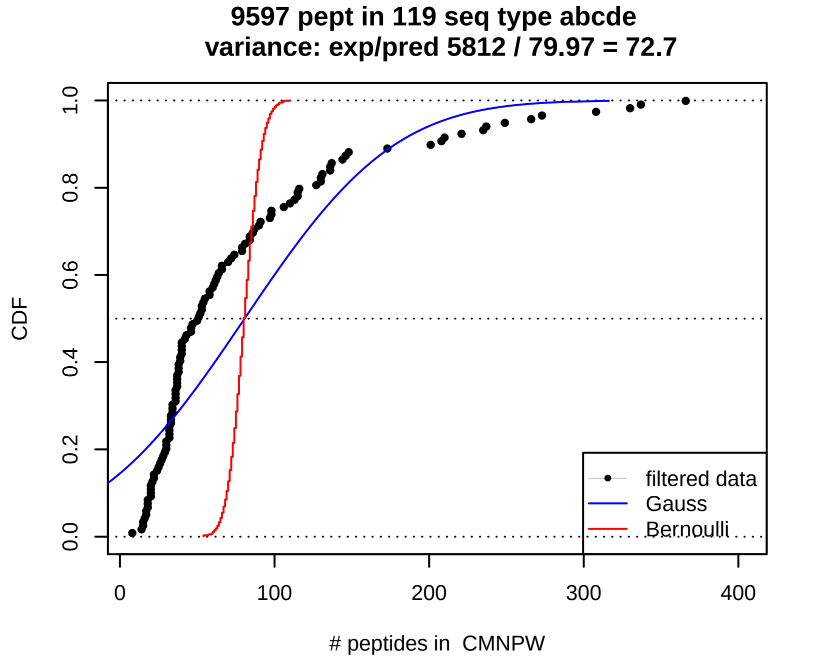
9597 pept in 119 seq type abcde
variance: exp/pred 5812 / 79.97 = 72.7
CDF
# peptides in CMNPW
filtered data
Gauss
Bernoulli

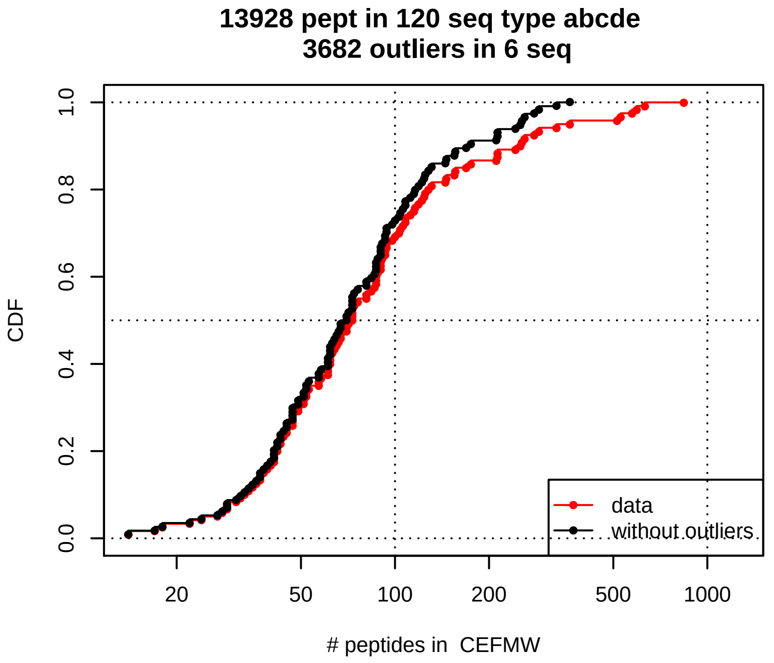
13928 pept in 120 seq type abcde
3682 outliers in 6 seq
CDF
# peptides in CEFMW
data
without outliers
0.0
0.2
0.4
0.6
0.8
1.0
20
50
100
200
500
1000

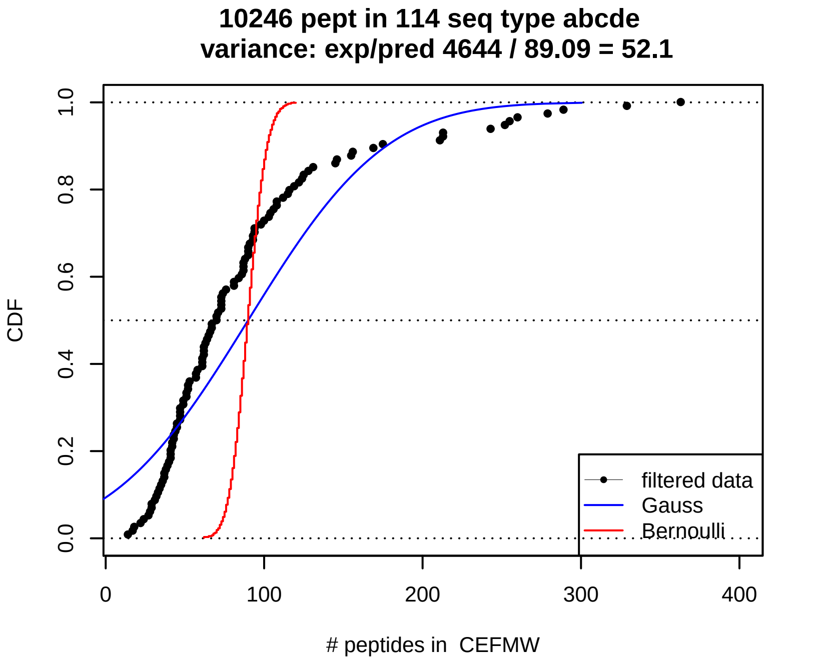
10246 pept in 114 seq type abcde
variance: exp/pred 4644 / 89.09 = 52.1
CDF
# peptides in CEFMW
filtered data
Gauss
Bernoulli
0.0
0.2
0.4
0.6
0.8
1.0
0
100
200
300
400

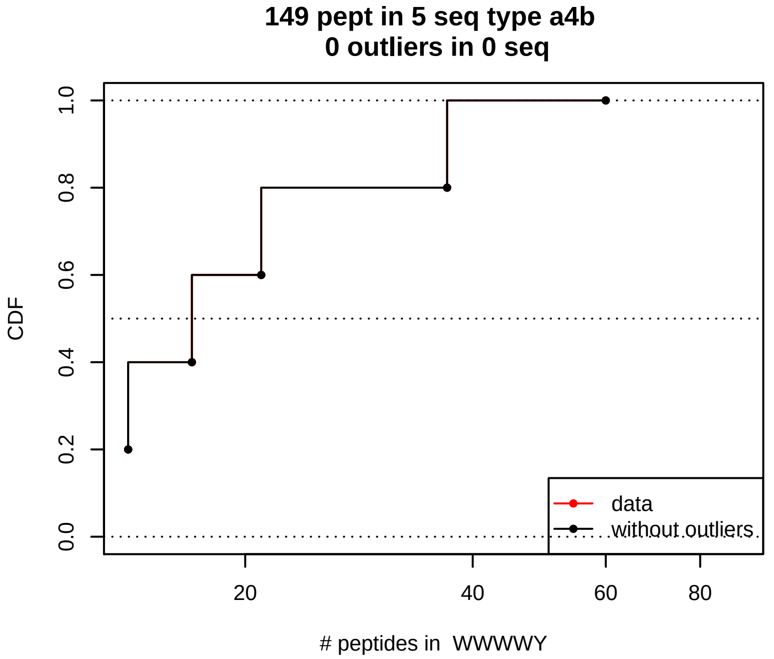
149 pept in 5 seq type a4b
0 outliers in 0 seq
CDF
# peptides in WWWWY
data
without outliers
0.0
0.2
0.4
0.6
0.8
1.0
20
40
60
80

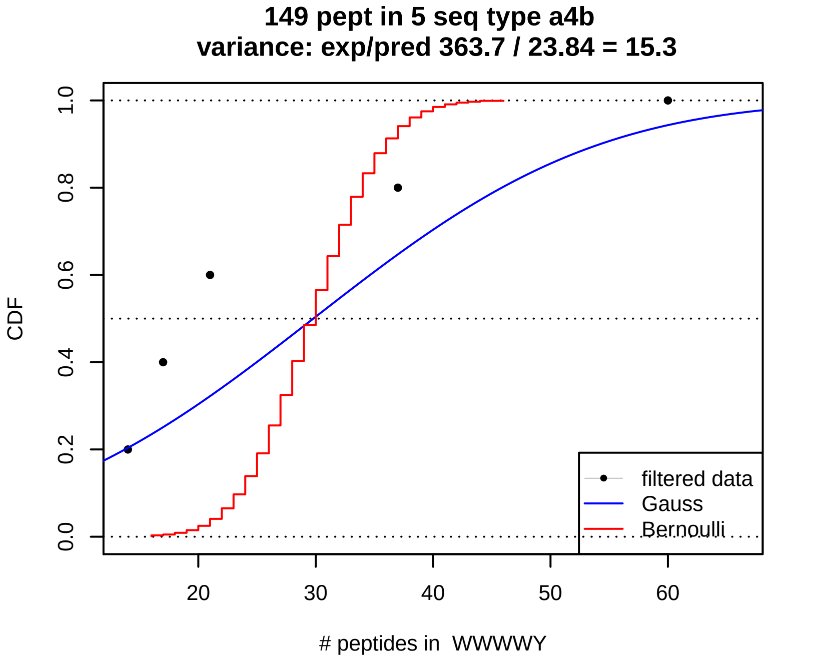
149 pept in 5 seq type a4b
variance: exp/pred 363.7 / 23.84 = 15.3
CDF
# peptides in WWWWY
filtered data
Gauss
Bernoulli
0.0
0.2
0.4
0.6
0.8
1.0
20
30
40
50
60

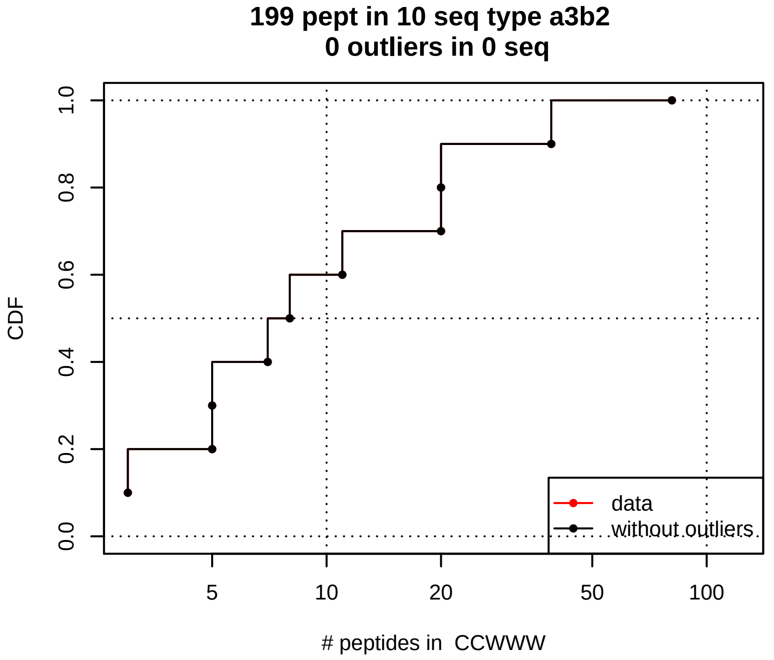
199 pept in 10 seq type a3b2
0 outliers in 0 seq
CDF
# peptides in CCWWW
data
without outliers
0.0
0.2
0.4
0.6
0.8
1.0
5
10
20
50
100

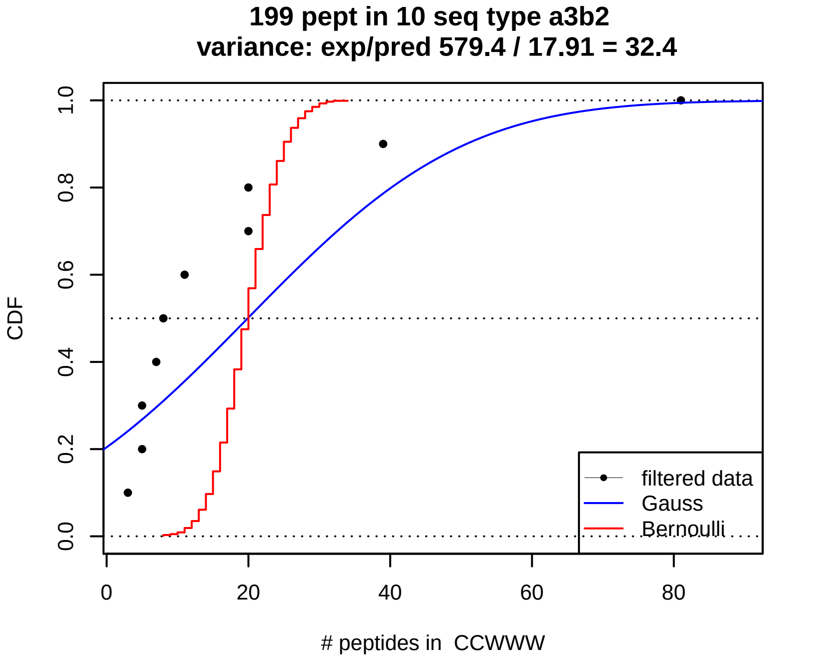
199 pept in 10 seq type a3b2
variance: exp/pred 579.4 / 17.91 = 32.4
CDF
# peptides in CCWWW
filtered data
Gauss
Bernoulli
0.0
0.2
0.4
0.6
0.8
1.0
0
20
40
60
80

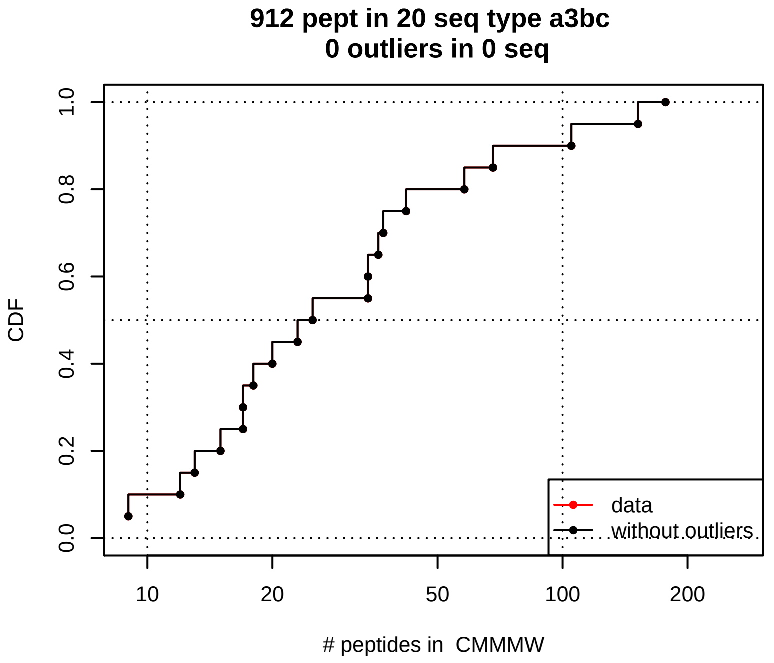
912 pept in 20 seq type a3bc
0 outliers in 0 seq
CDF
# peptides in CMMMW
data
without outliers

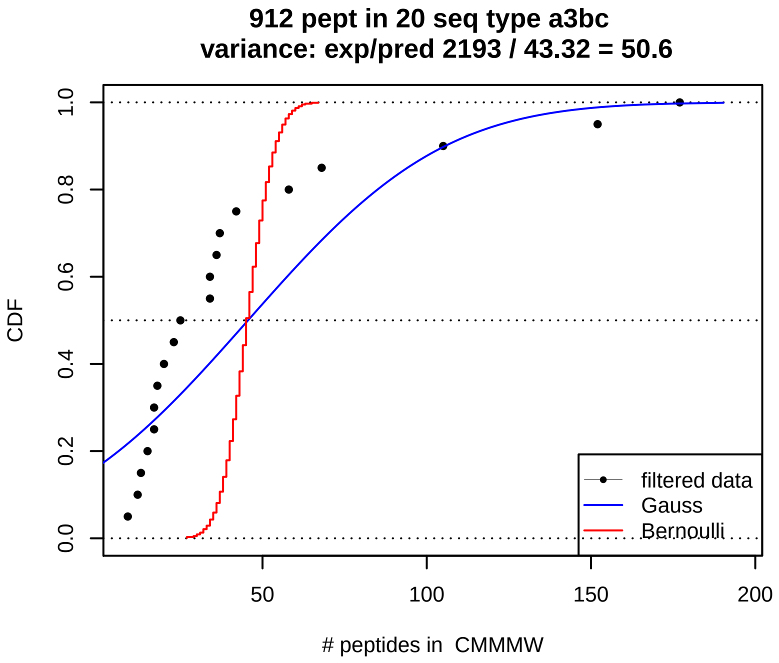
912 pept in 20 seq type a3bc
variance: exp/pred 2193 / 43.32 = 50.6
CDF
# peptides in CMMMW
filtered data
Gauss
Bernoulli

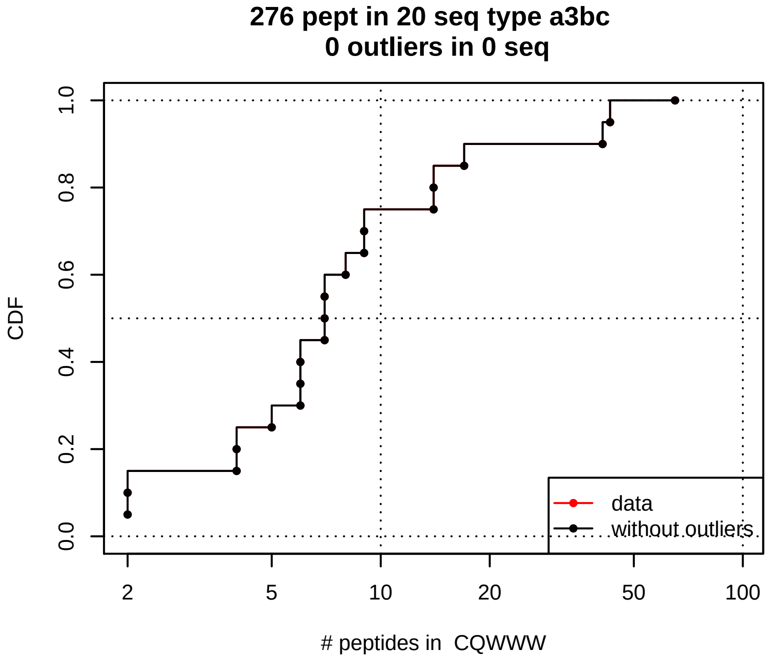
276 pept in 20 seq type a3bc
0 outliers in 0 seq
CDF
# peptides in CQWWW
data
without outliers

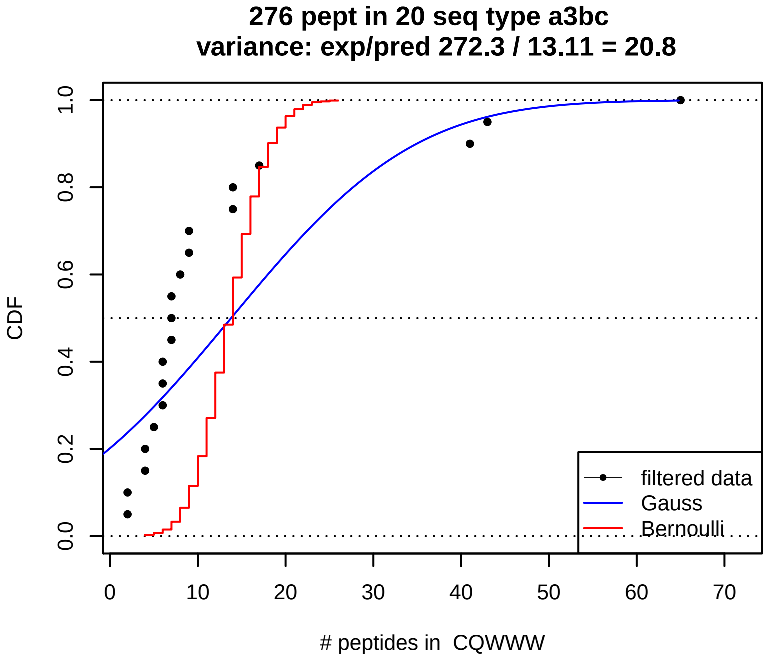
276 pept in 20 seq type a3bc
variance: exp/pred 272.3 / 13.11 = 20.8
CDF
# peptides in CQWWW
filtered data
Gauss
Bernoulli

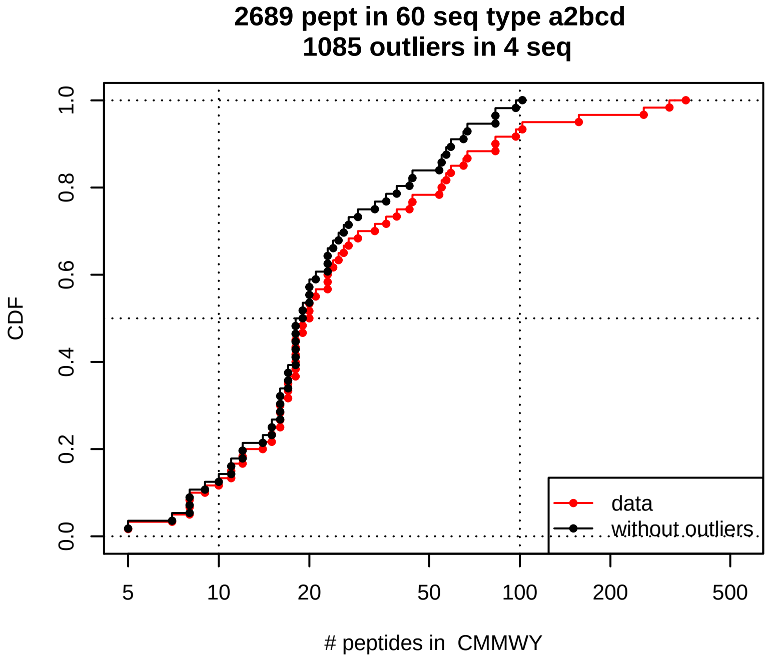
2689 pept in 60 seq type a2bcd
1085 outliers in 4 seq
CDF
# peptides in CMMWY
data
without outliers

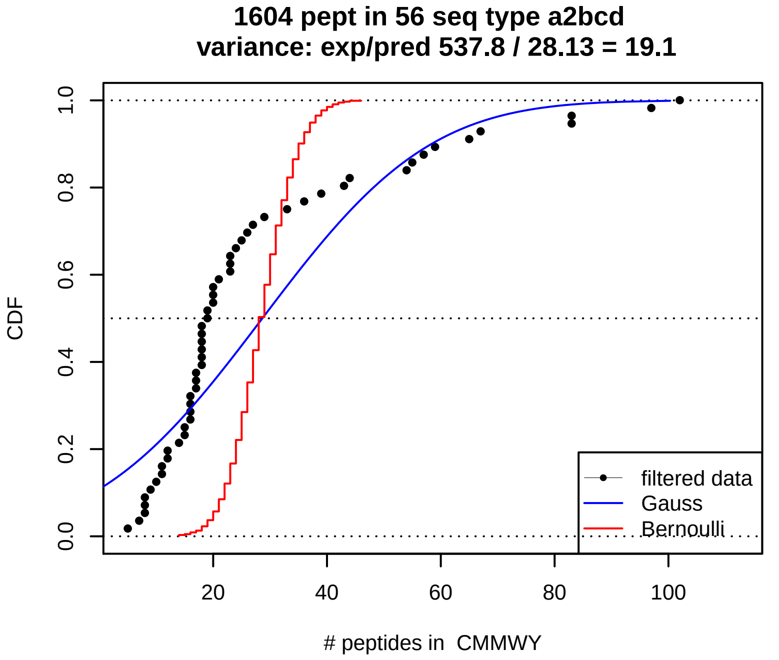
1604 pept in 56 seq type a2bcd
variance: exp/pred 537.8 / 28.13 = 19.1
CDF
# peptides in CMMWY
filtered data
Gauss
Bernoulli

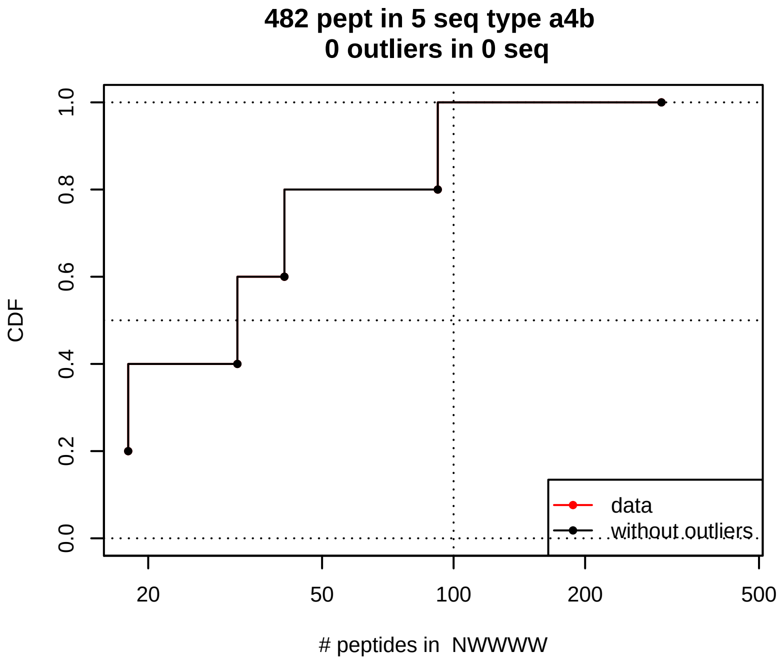

482 pept in 5 seq type a4b
0 outliers in 0 seq
CDF
data
without outliers
# peptides in NWWWW


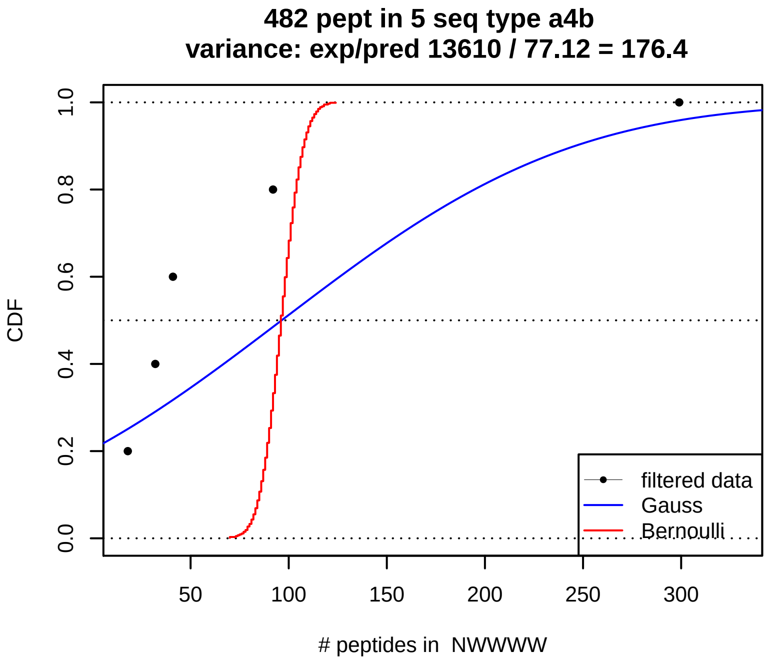

482 pept in 5 seq type a4b
variance: exp/pred 13610 / 77.12 = 176.4
CDF
filtered data
Gauss
Bernoulli
# peptides in NWWWW


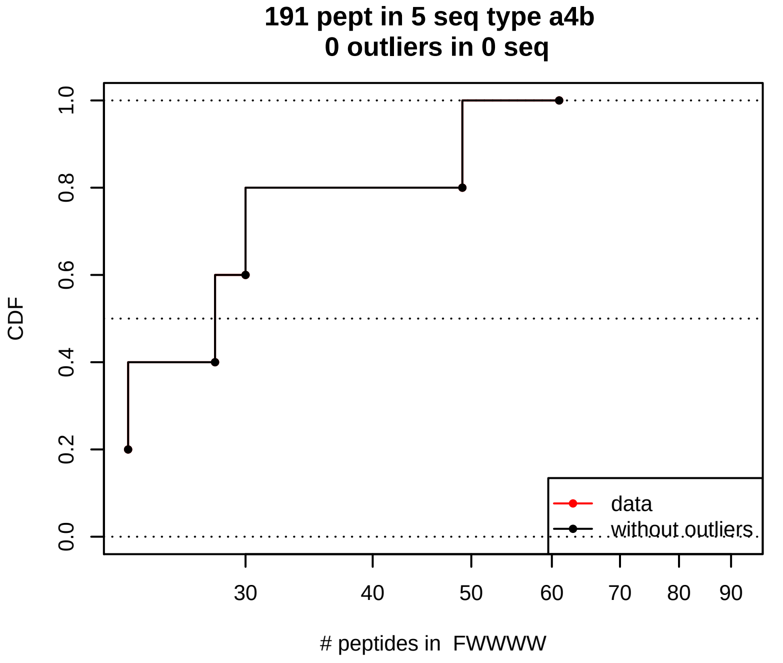

191 pept in 5 seq type a4b
0 outliers in 0 seq
CDF
data
without outliers
# peptides in FWWWW


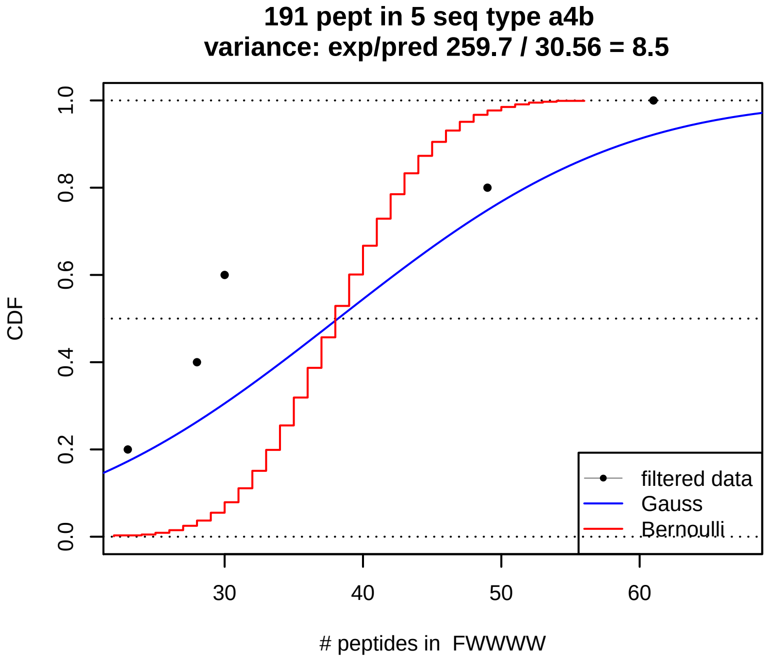

191 pept in 5 seq type a4b
variance: exp/pred 259.7 / 30.56 = 8.5
CDF
filtered data
Gauss
Bernoulli
# peptides in FWWWW


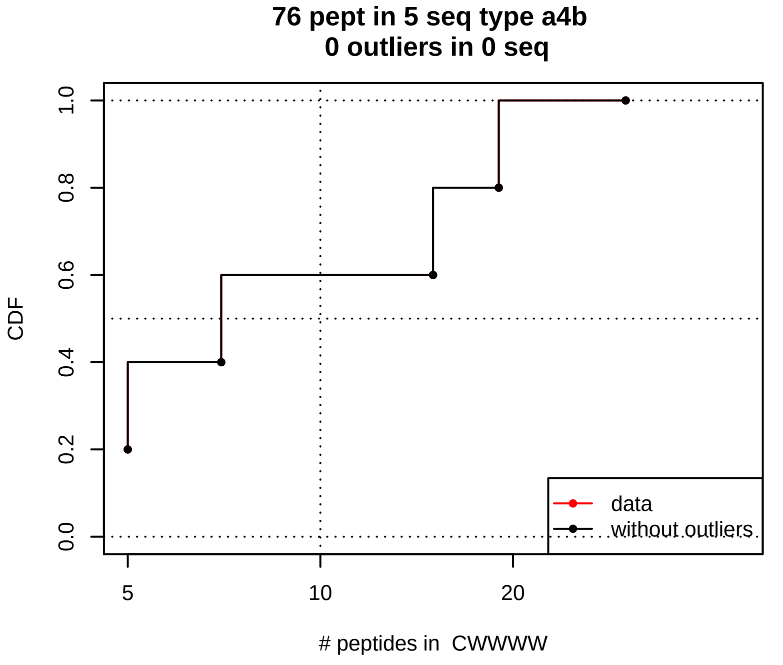

76 pept in 5 seq type a4b
0 outliers in 0 seq
CDF
data
without outliers
# peptides in CWWWW


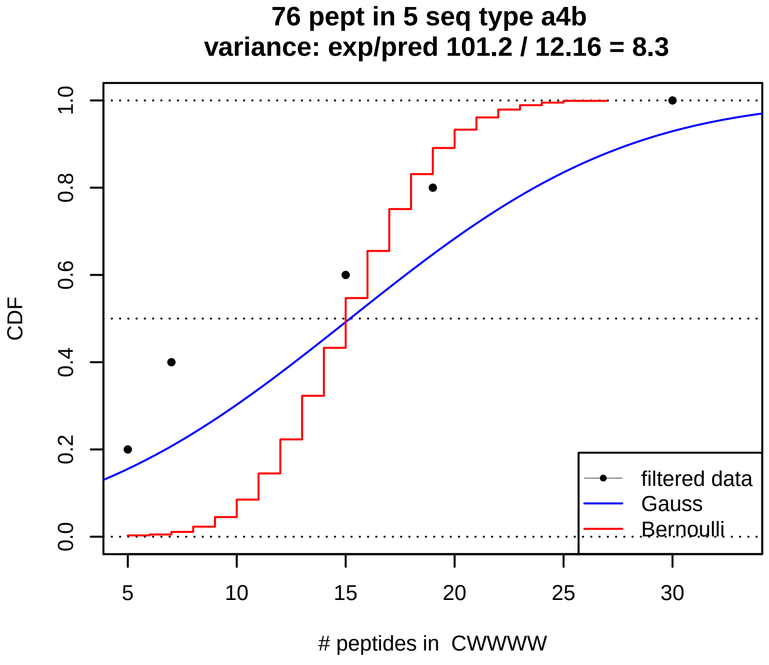

76 pept in 5 seq type a4b
variance: exp/pred 101.2 / 12.16 = 8.3
CDF
filtered data
Gauss
Bernoulli
# peptides in CWWWW

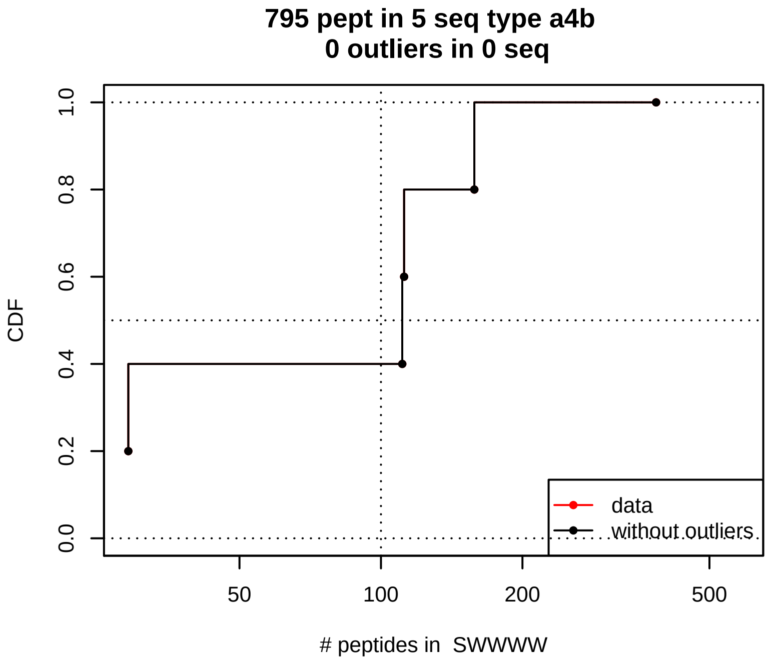
795 pept in 5 seq type a4b
0 outliers in 0 seq
CDF
0.0
0.2
0.4
0.6
0.8
1.0
50
100
200
500
data
without outliers
# peptides in SWWWW

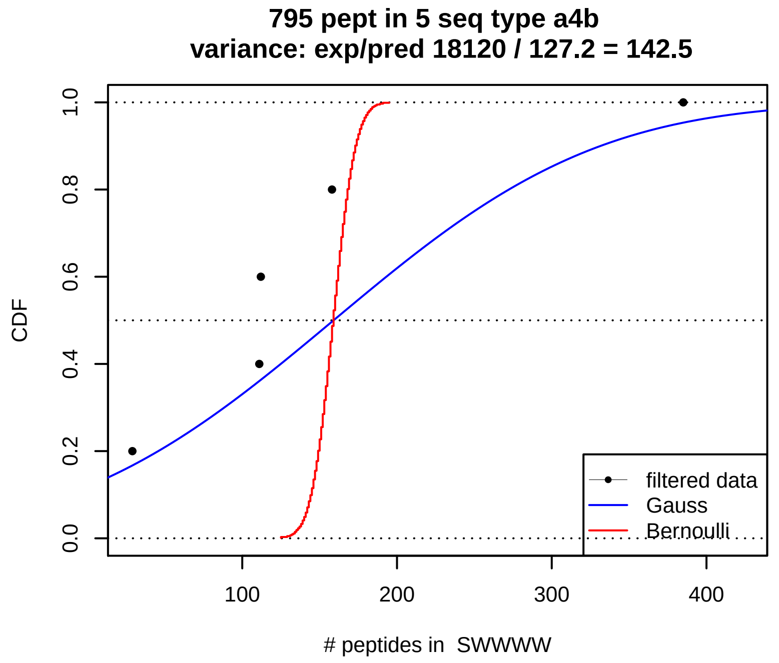
795 pept in 5 seq type a4b
variance: exp/pred 18120 / 127.2 = 142.5
CDF
0.0
0.2
0.4
0.6
0.8
1.0
100
200
300
400
filtered data
Gauss
Bernoulli
# peptides in SWWWW

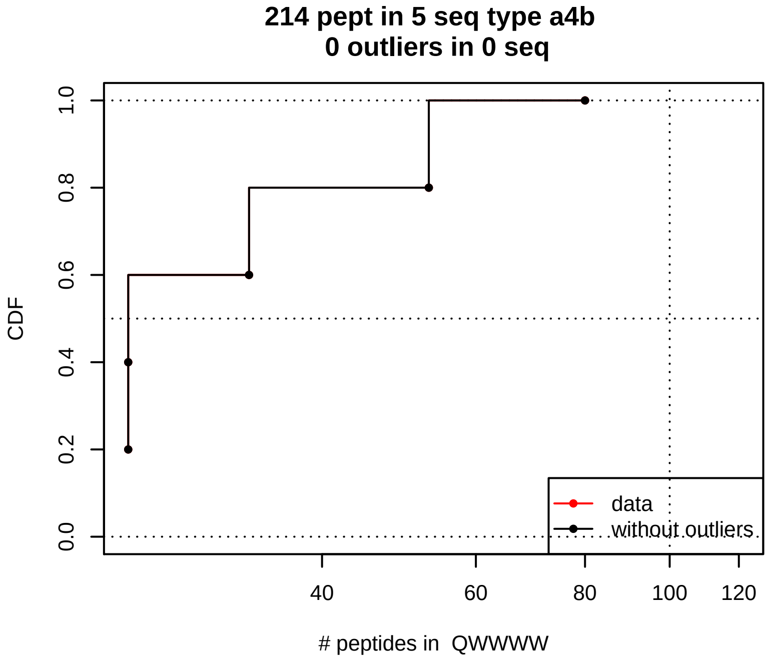
214 pept in 5 seq type a4b
0 outliers in 0 seq
CDF
0.0
0.2
0.4
0.6
0.8
1.0
40
60
80
100
120
data
without outliers
# peptides in QWWWW

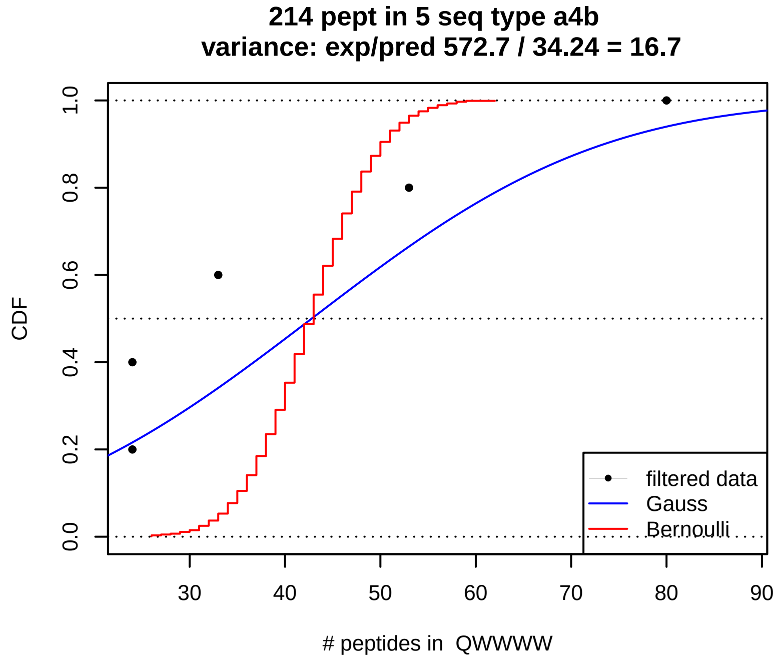
214 pept in 5 seq type a4b
variance: exp/pred 572.7 / 34.24 = 16.7
CDF
0.0
0.2
0.4
0.6
0.8
1.0
30
40
50
60
70
80
90
filtered data
Gauss
Bernoulli
# peptides in QWWWW

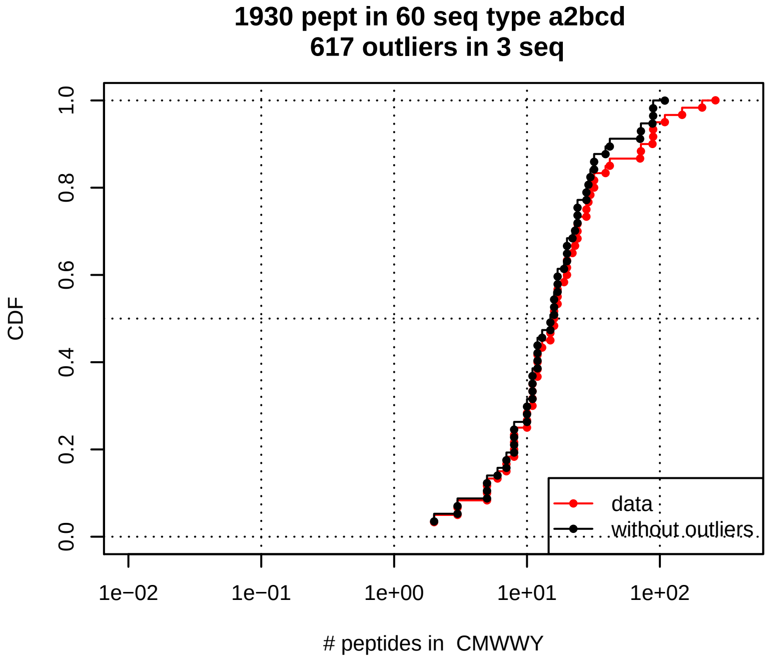
1930 pept in 60 seq type a2bcd
617 outliers in 3 seq
CDF
0.0
0.2
0.4
0.6
0.8
1.0
1e−02
1e−01
1e+00
1e+01
1e+02
data
without outliers
# peptides in CMWWY

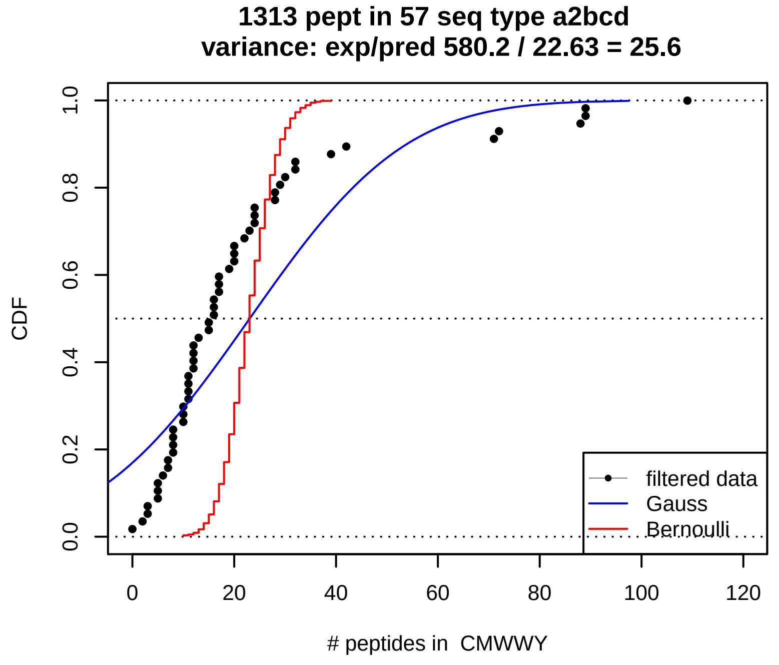
1313 pept in 57 seq type a2bcd
variance: exp/pred 580.2 / 22.63 = 25.6
CDF
0.0
0.2
0.4
0.6
0.8
1.0
0
20
40
60
80
100
120
filtered data
Gauss
Bernoulli
# peptides in CMWWY

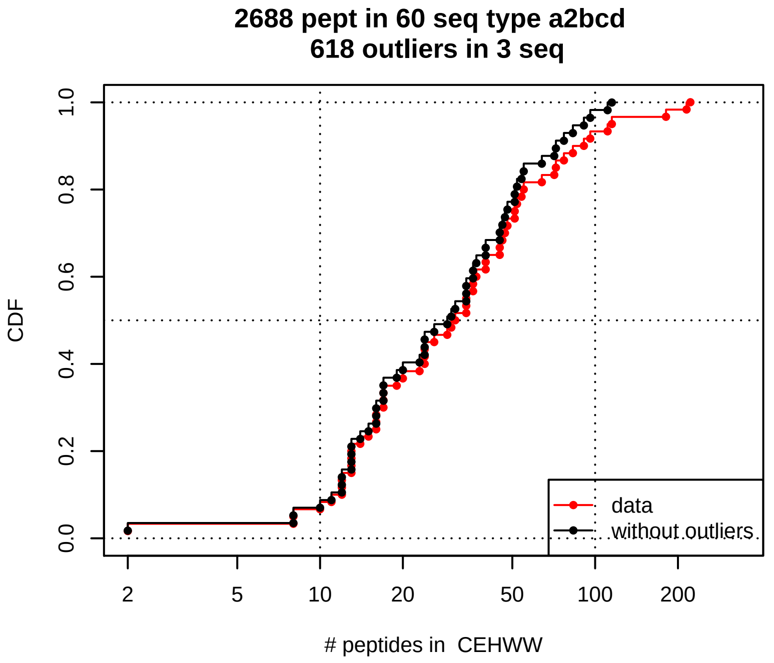
2688 pept in 60 seq type a2bcd
618 outliers in 3 seq
CDF
# peptides in CEHWW
data
without outliers

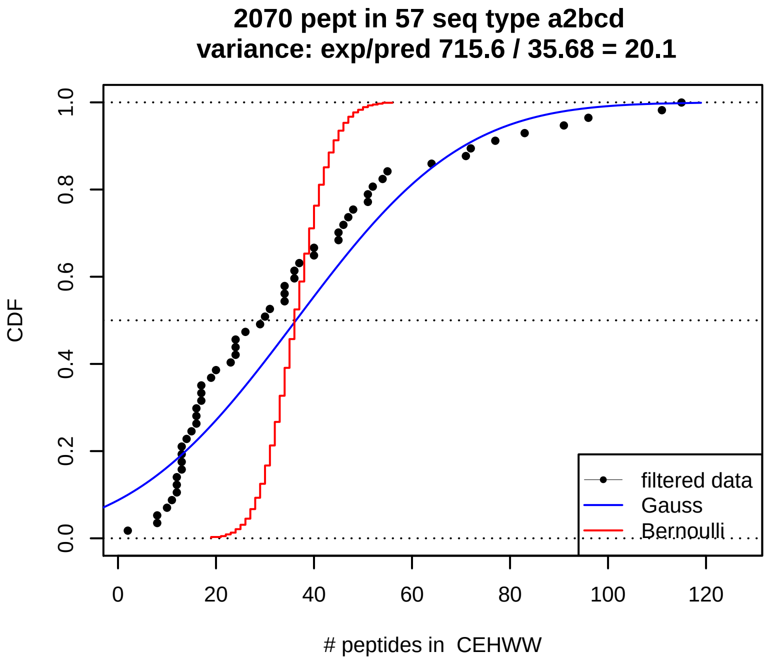
2070 pept in 57 seq type a2bcd
variance: exp/pred 715.6 / 35.68 = 20.1
CDF
# peptides in CEHWW
filtered data
Gauss
Bernoulli

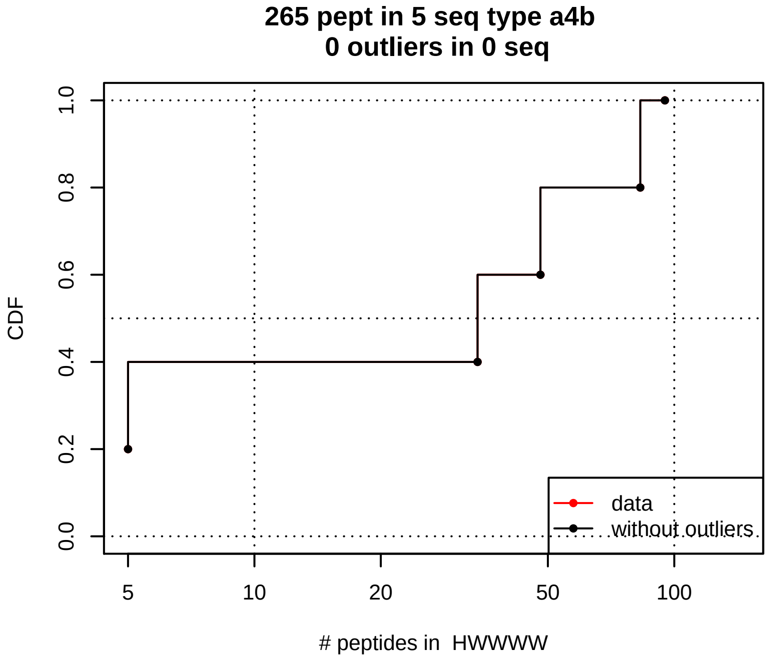
265 pept in 5 seq type a4b
0 outliers in 0 seq
CDF
# peptides in HWWWW
data
without outliers

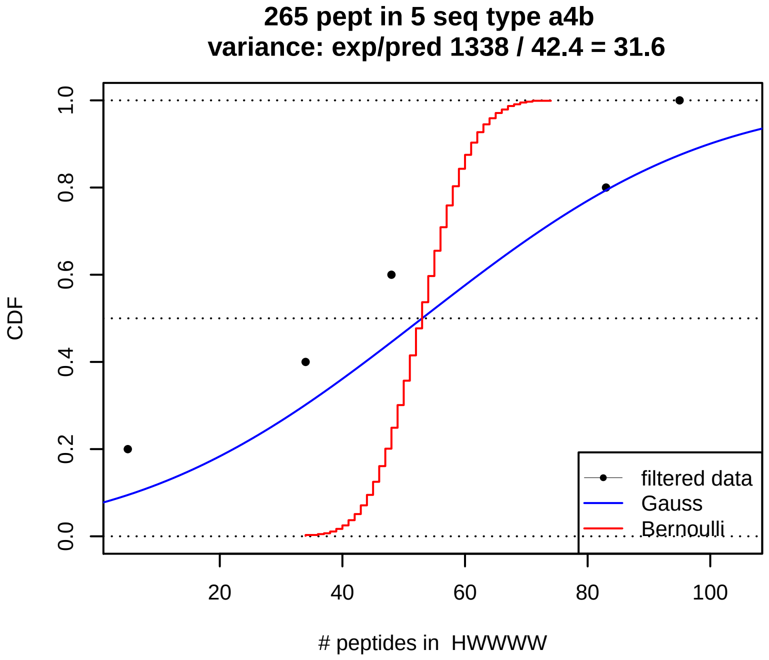
265 pept in 5 seq type a4b
variance: exp/pred 1338 / 42.4 = 31.6
CDF
# peptides in HWWWW
filtered data
Gauss
Bernoulli

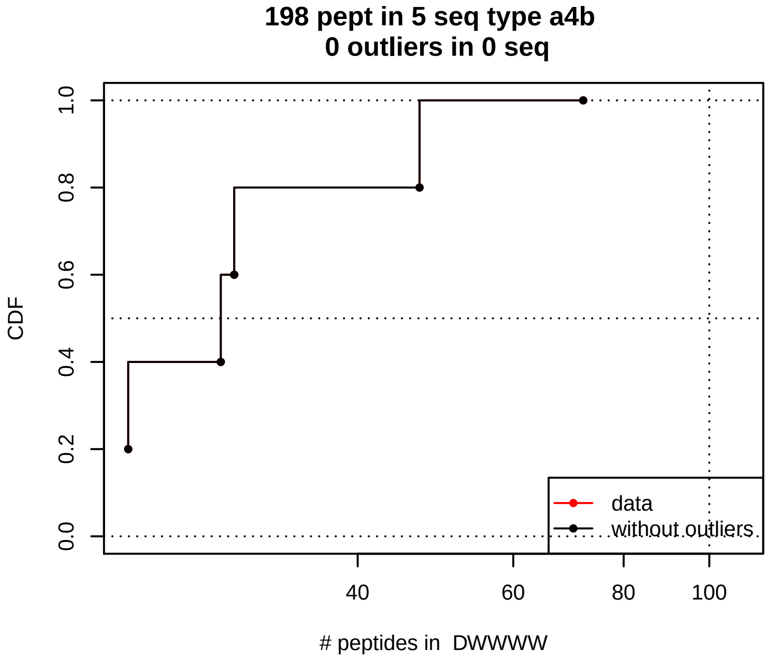
198 pept in 5 seq type a4b
0 outliers in 0 seq
CDF
# peptides in DWWWW
data
without outliers

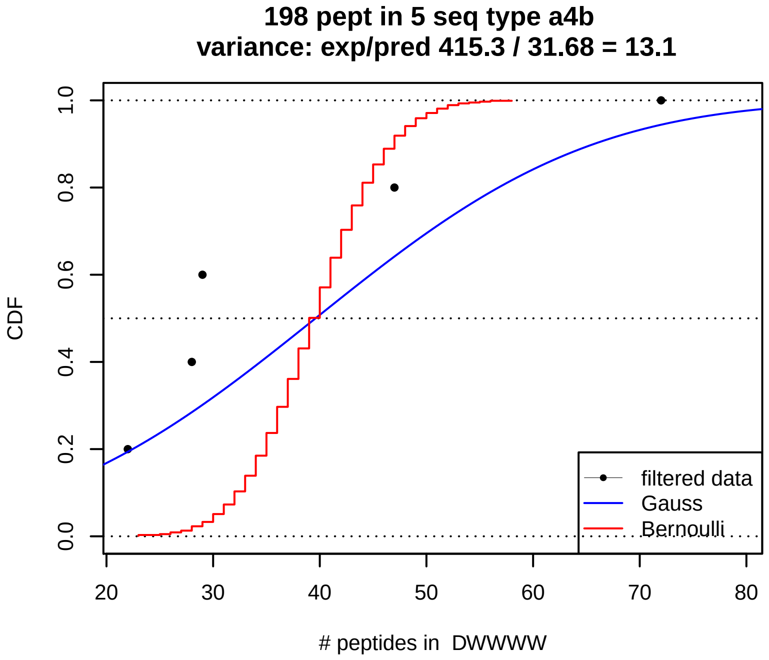
198 pept in 5 seq type a4b
variance: exp/pred 415.3 / 31.68 = 13.1
CDF
# peptides in DWWWW
filtered data
Gauss
Bernoulli

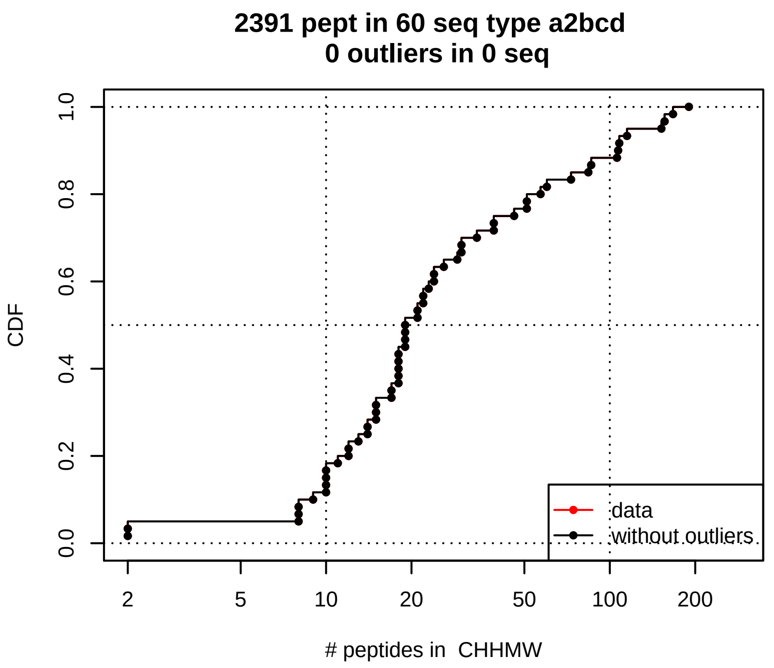
2391 pept in 60 seq type a2bcd
0 outliers in 0 seq
CDF
# peptides in CHHMW
data
without outliers

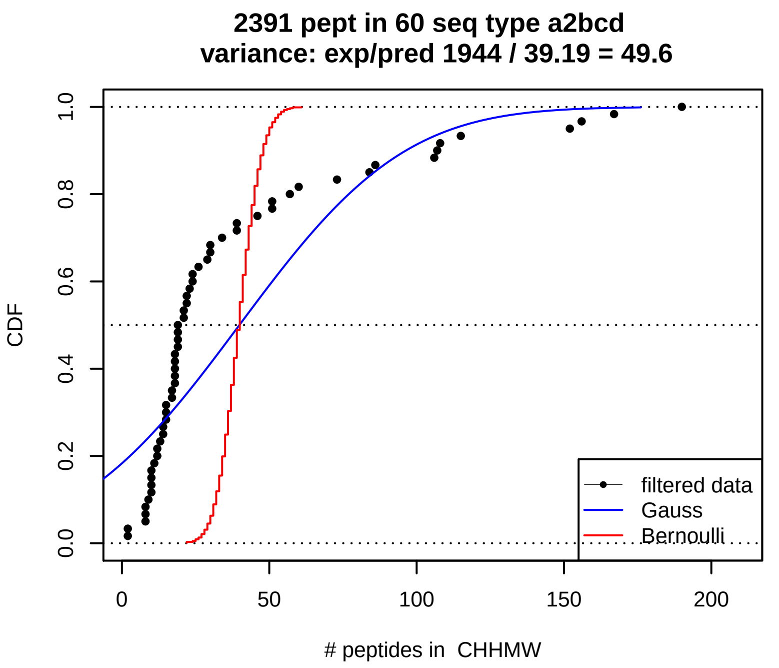
2391 pept in 60 seq type a2bcd
variance: exp/pred 1944 / 39.19 = 49.6
CDF
# peptides in CHHMW
filtered data
Gauss
Bernoulli

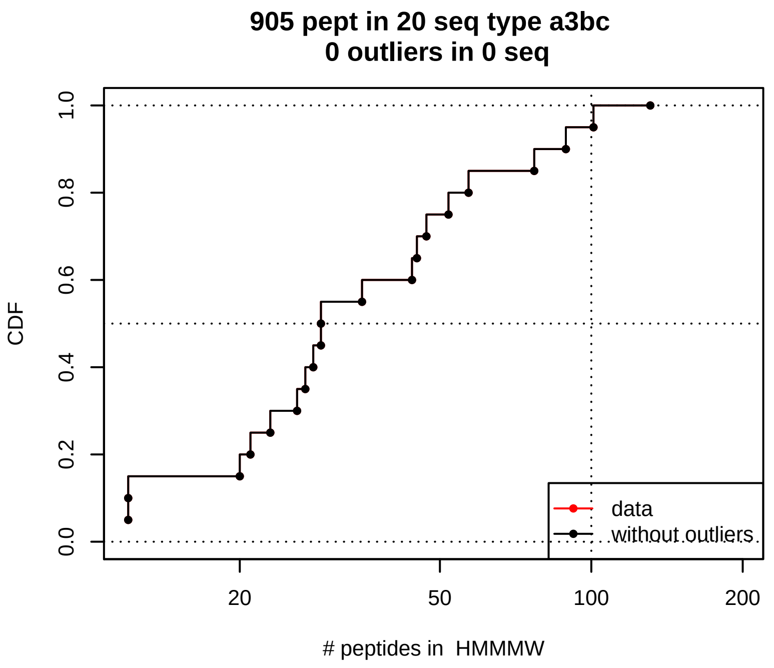
905 pept in 20 seq type a3bc
0 outliers in 0 seq
CDF
# peptides in HMMMW
data
without outliers

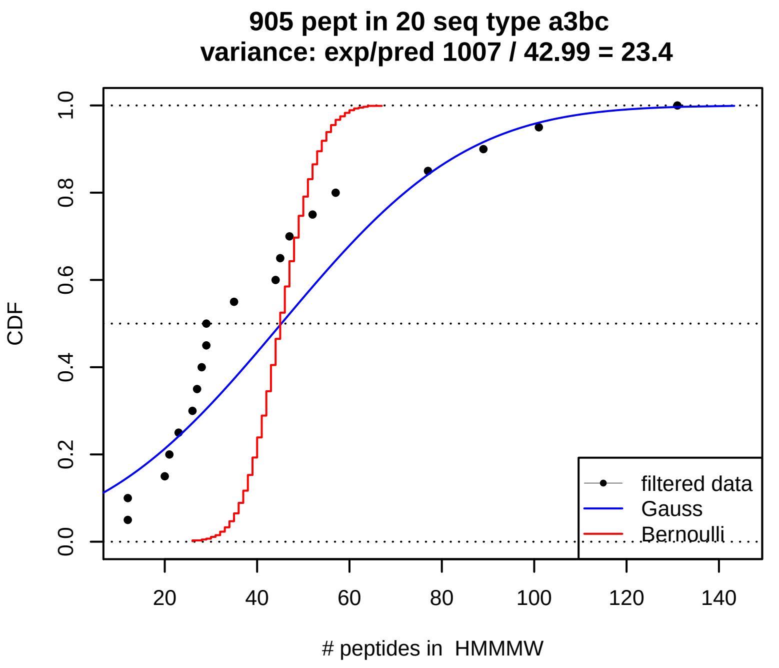
905 pept in 20 seq type a3bc
variance: exp/pred 1007 / 42.99 = 23.4
CDF
# peptides in HMMMW
filtered data
Gauss
Bernoulli

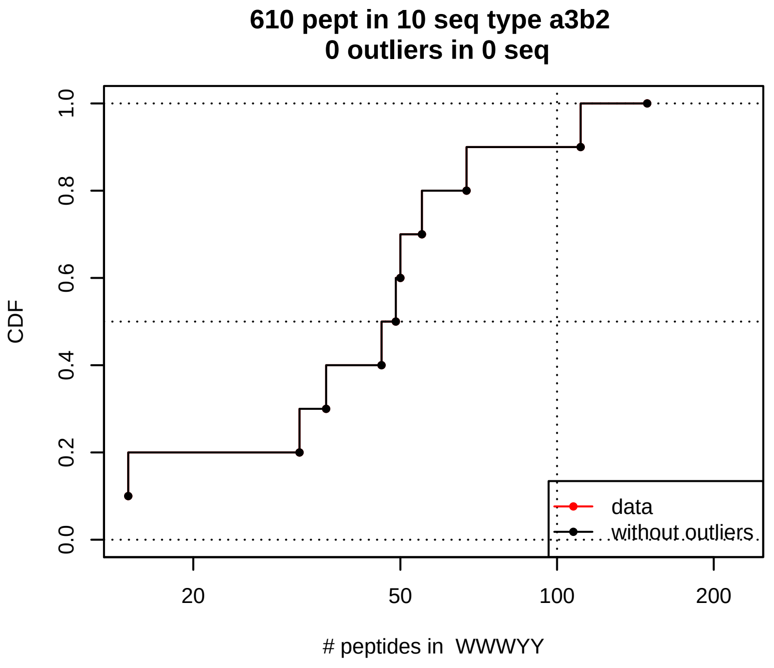
610 pept in 10 seq type a3b2
0 outliers in 0 seq
CDF
# peptides in WWWYY
data
without outliers

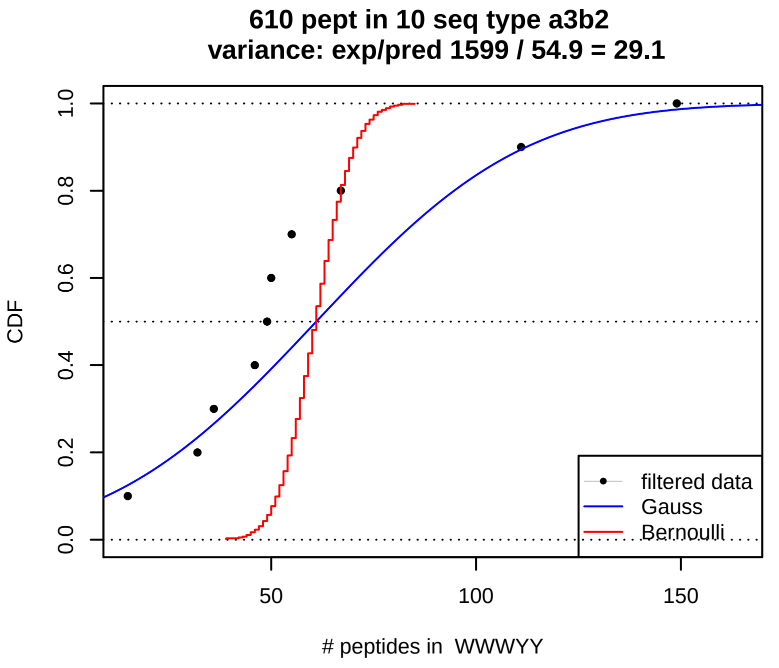
610 pept in 10 seq type a3b2
variance: exp/pred 1599 / 54.9 = 29.1
CDF
# peptides in WWWYY
filtered data
Gauss
Bernoulli

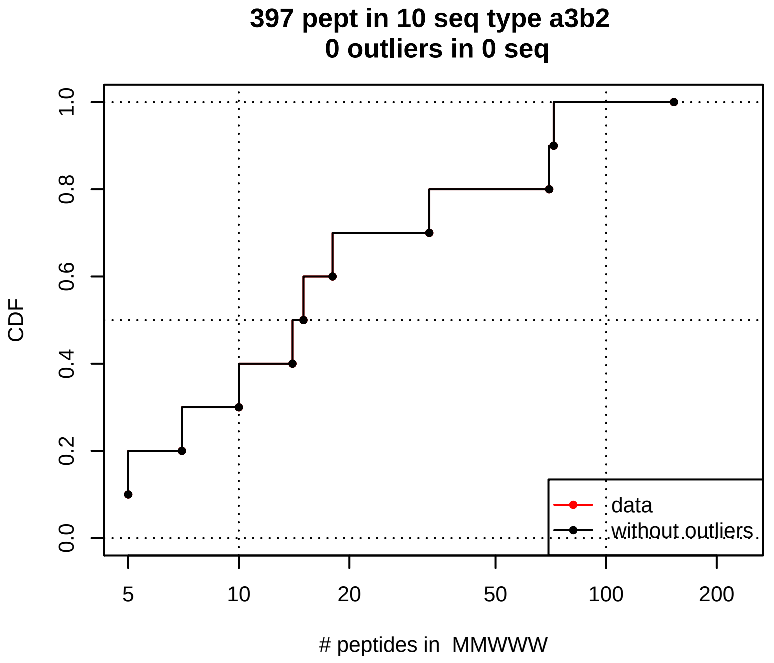
397 pept in 10 seq type a3b2
0 outliers in 0 seq
CDF
# peptides in MMWWW
data
without outliers

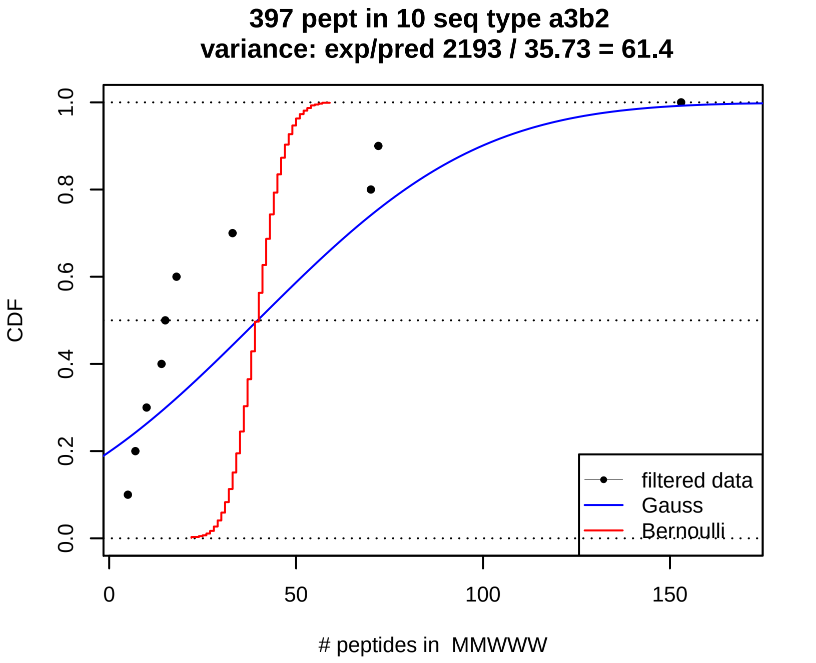
397 pept in 10 seq type a3b2
variance: exp/pred 2193 / 35.73 = 61.4
CDF
# peptides in MMWWW
filtered data
Gauss
Bernoulli

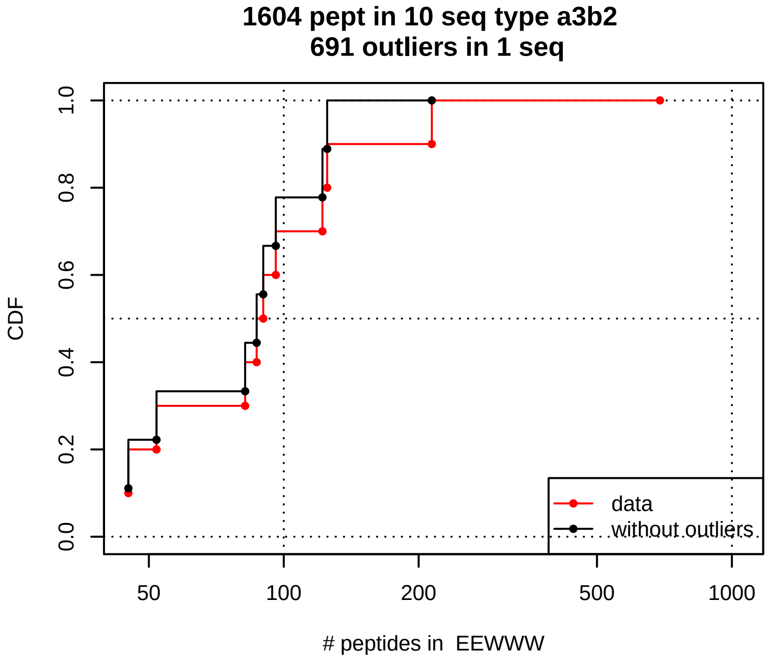
1604 pept in 10 seq type a3b2
691 outliers in 1 seq
CDF
# peptides in EEWWW
data
without outliers

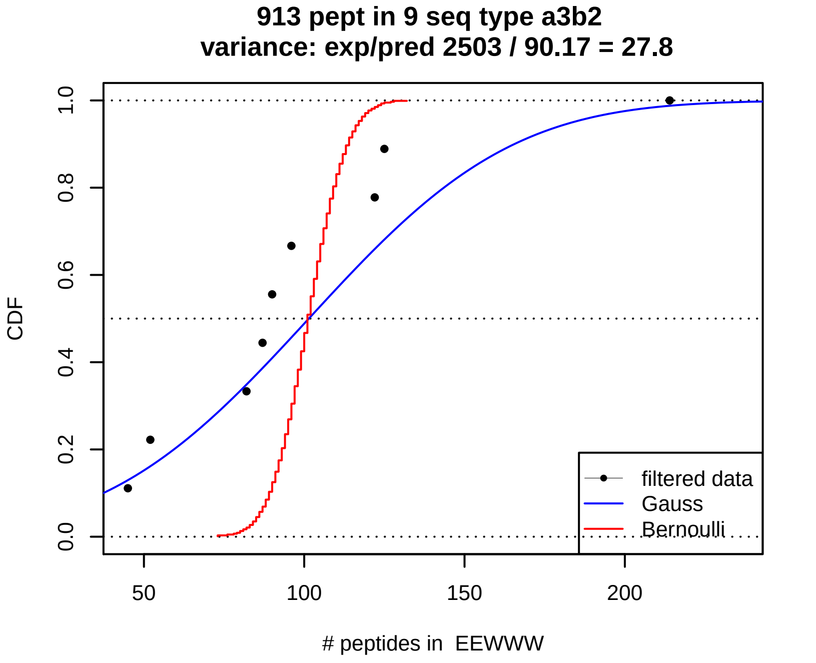
913 pept in 9 seq type a3b2
variance: exp/pred 2503 / 90.17 = 27.8
CDF
# peptides in EEWWW
filtered data
Gauss
Bernoulli

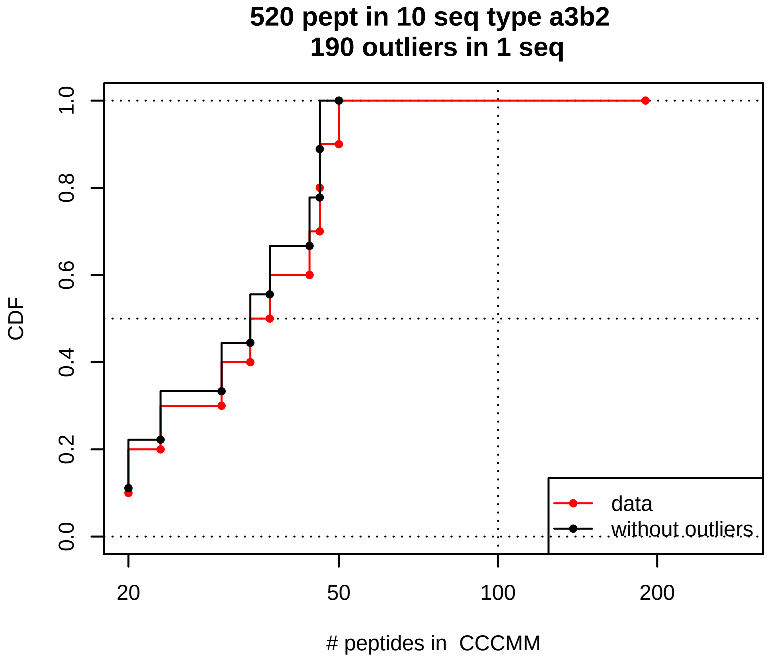
520 pept in 10 seq type a3b2
190 outliers in 1 seq
CDF
# peptides in CCCMM
data
without outliers

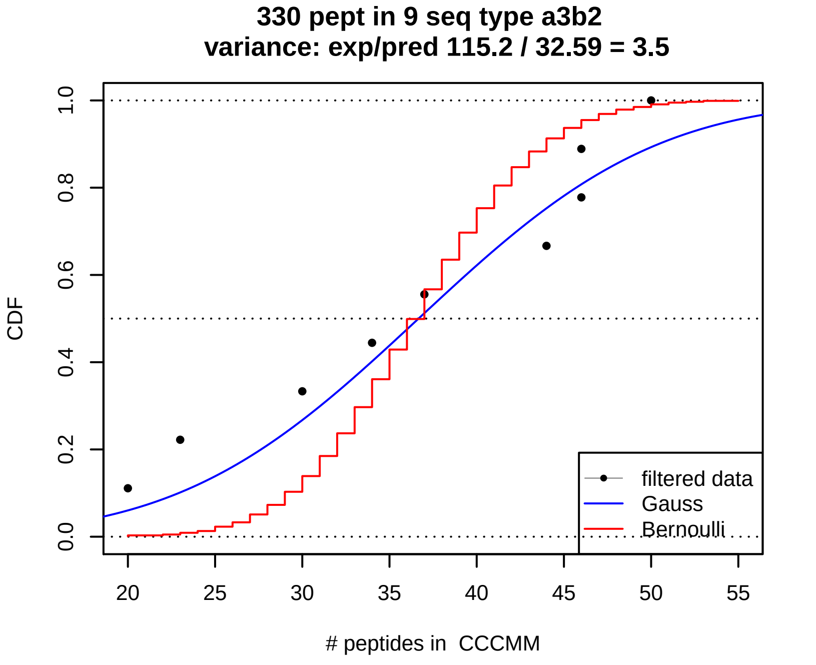
330 pept in 9 seq type a3b2
variance: exp/pred 115.2 / 32.59 = 3.5
CDF
# peptides in CCCMM
filtered data
Gauss
Bernoulli

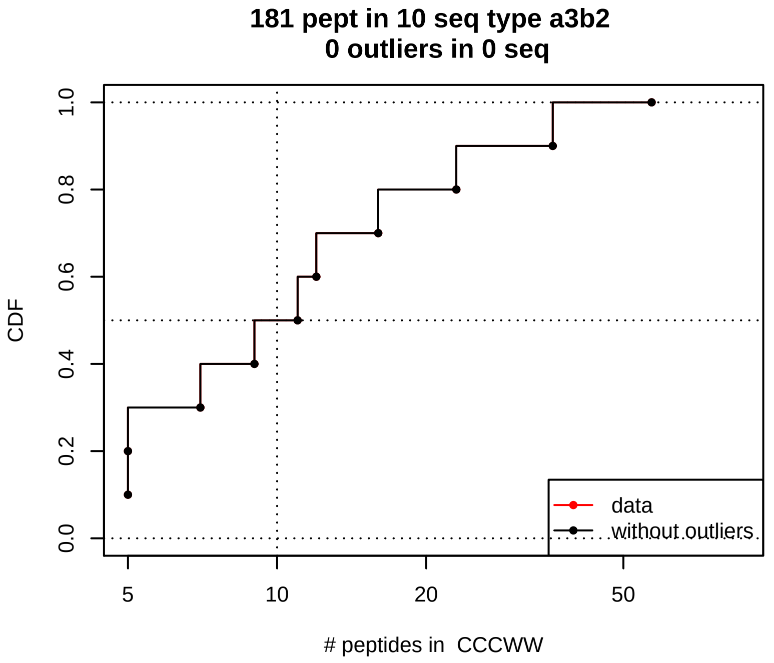

181 pept in 10 seq type a3b2
0 outliers in 0 seq
CDF
# peptides in CCCWW
data
without outliers


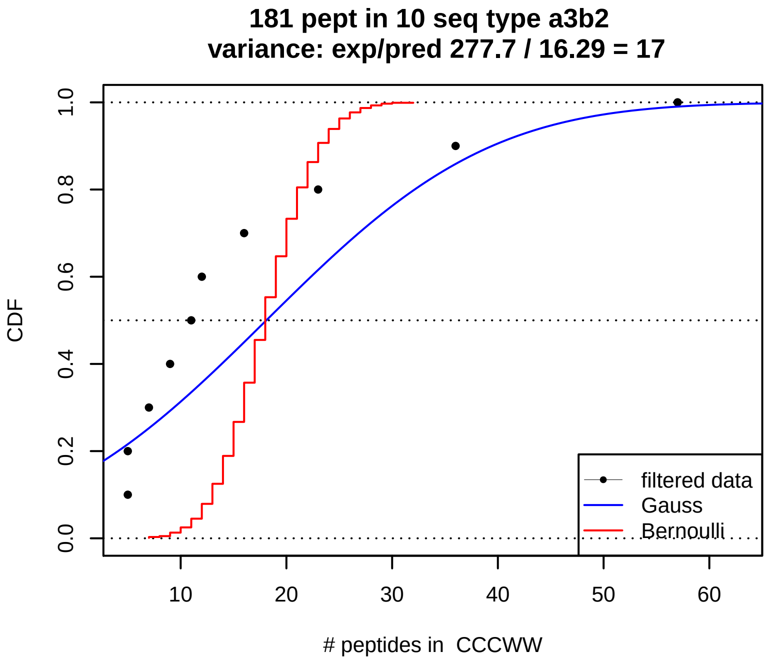

181 pept in 10 seq type a3b2
variance: exp/pred 277.7 / 16.29 = 17
CDF
# peptides in CCCWW
filtered data
Gauss
Bernoulli


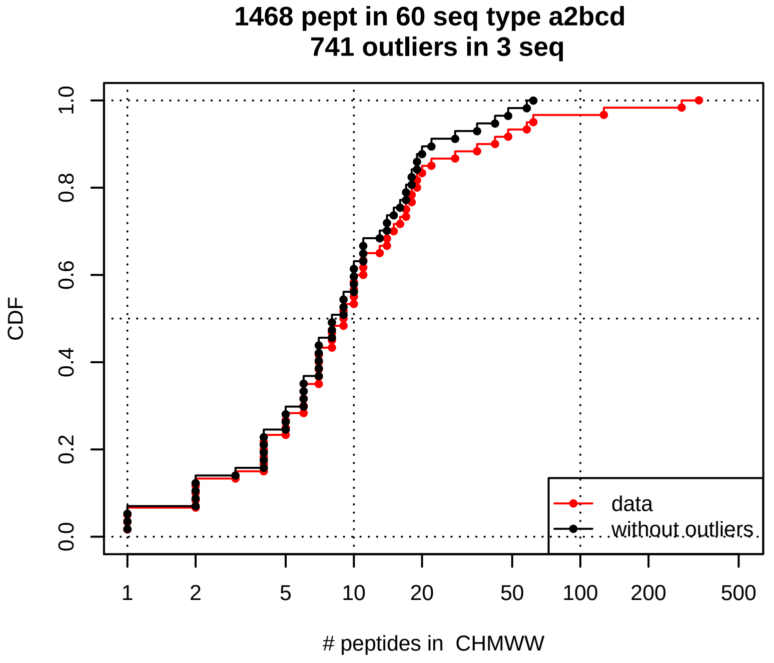

1468 pept in 60 seq type a2bcd
741 outliers in 3 seq
CDF
# peptides in CHMWW
data
without outliers


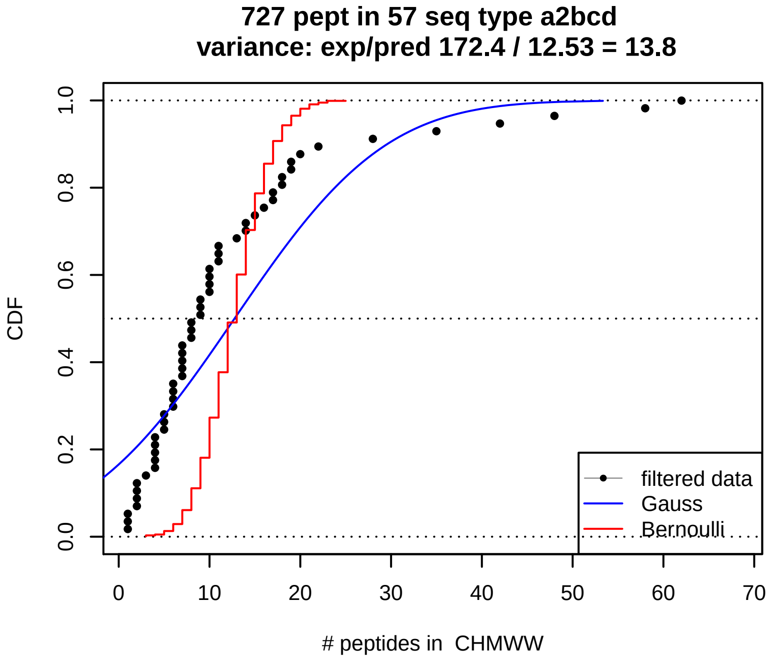

727 pept in 57 seq type a2bcd
variance: exp/pred 172.4 / 12.53 = 13.8
CDF
# peptides in CHMWW
filtered data
Gauss
Bernoulli


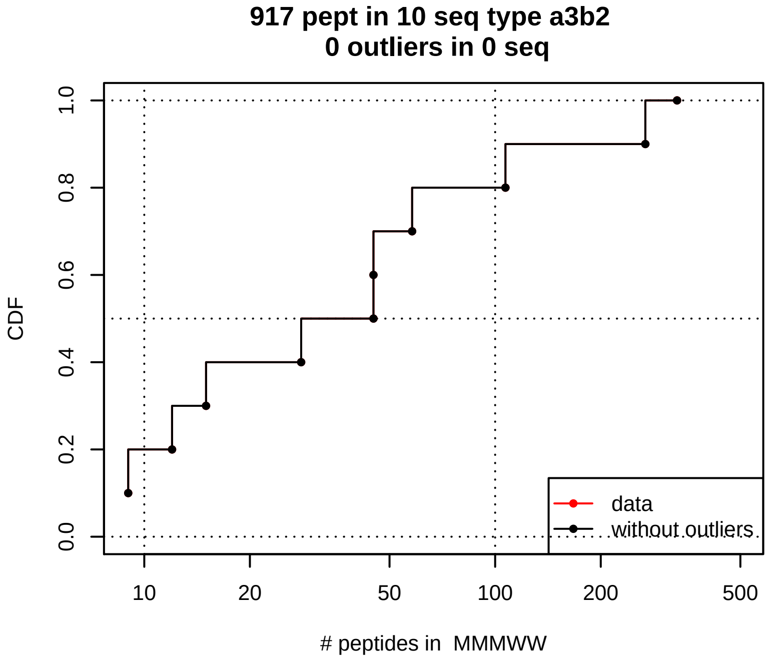

917 pept in 10 seq type a3b2
0 outliers in 0 seq
CDF
# peptides in MMMWW
data
without outliers


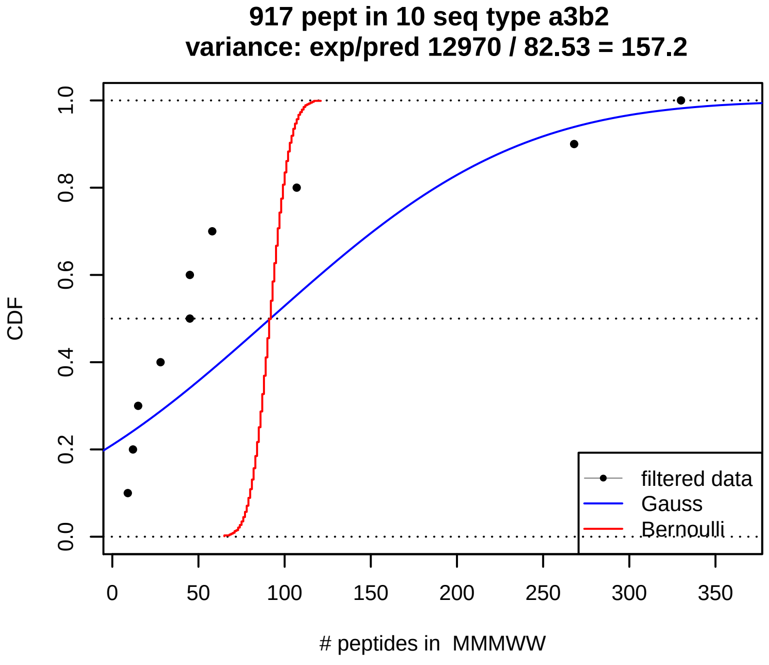

917 pept in 10 seq type a3b2
variance: exp/pred 12970 / 82.53 = 157.2
CDF
# peptides in MMMWW
filtered data
Gauss
Bernoulli

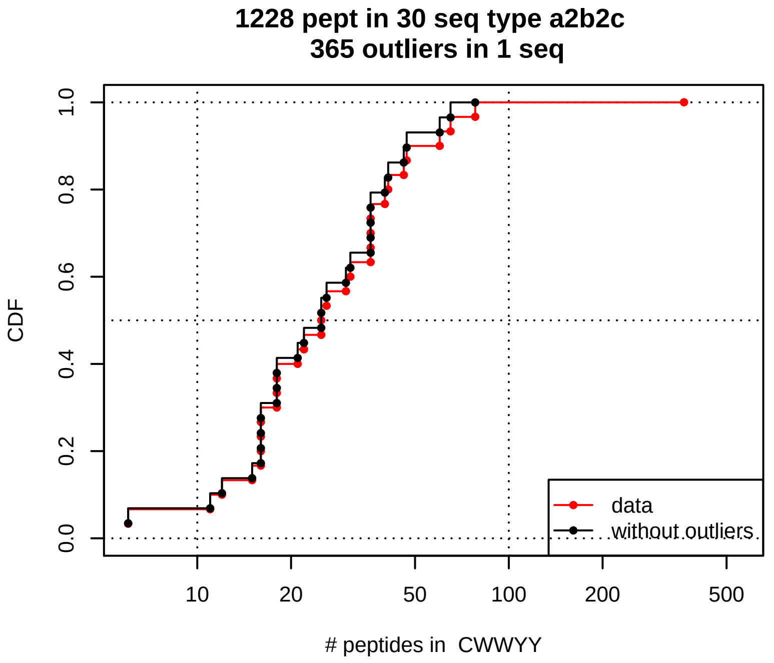
1228 pept in 30 seq type a2b2c
365 outliers in 1 seq
CDF
0.0
0.2
0.4
0.6
0.8
1.0
10
20
50
100
200
500
# peptides in CWWYY
data
without outliers

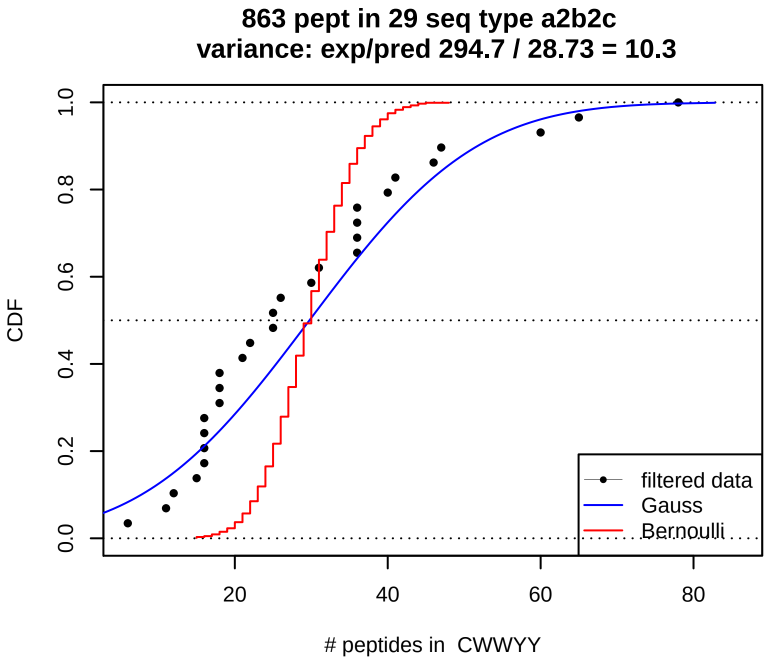
863 pept in 29 seq type a2b2c
variance: exp/pred 294.7 / 28.73 = 10.3
CDF
0.0
0.2
0.4
0.6
0.8
1.0
20
40
60
80
# peptides in CWWYY
filtered data
Gauss
Bernoulli

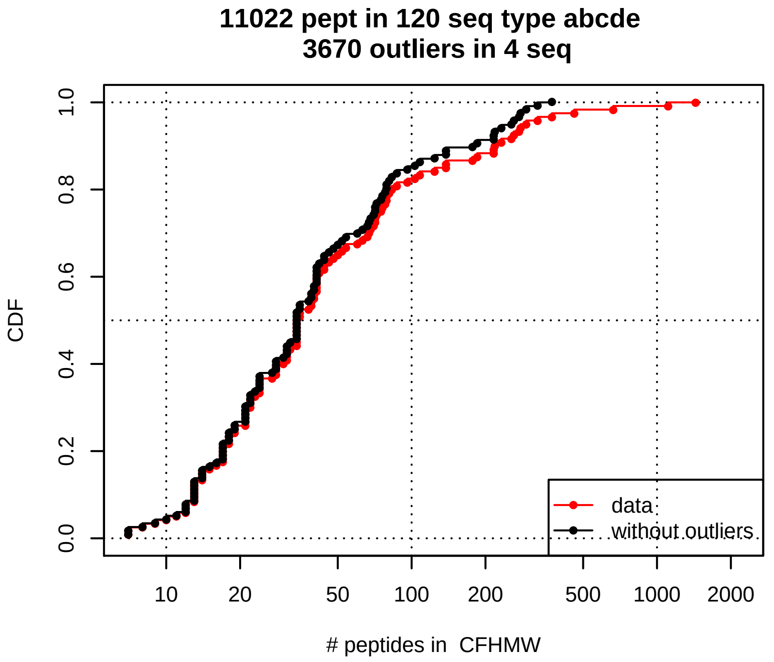
11022 pept in 120 seq type abcde
3670 outliers in 4 seq
CDF
0.0
0.2
0.4
0.6
0.8
1.0
10
20
50
100
200
500
1000
2000
# peptides in CFHMW
data
without outliers

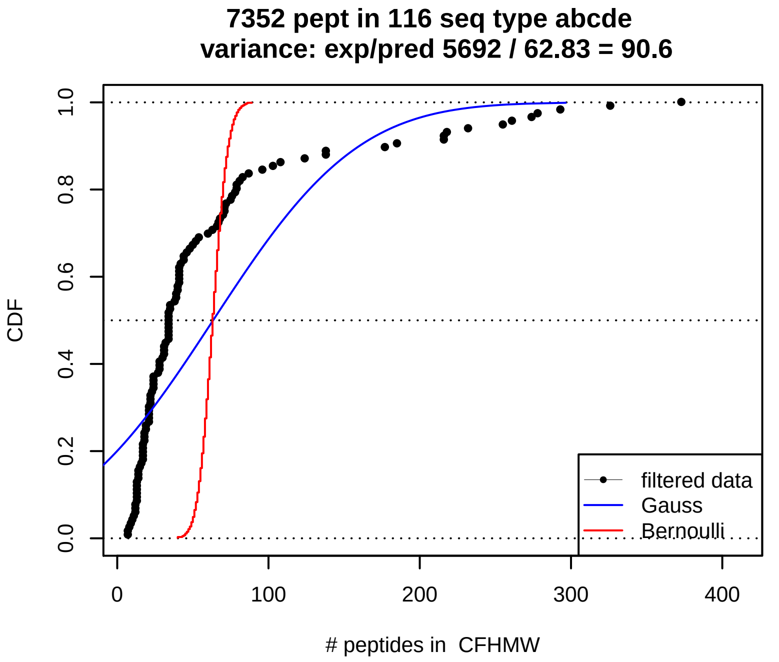
7352 pept in 116 seq type abcde
variance: exp/pred 5692 / 62.83 = 90.6
CDF
0.0
0.2
0.4
0.6
0.8
1.0
0
100
200
300
400
# peptides in CFHMW
filtered data
Gauss
Bernoulli

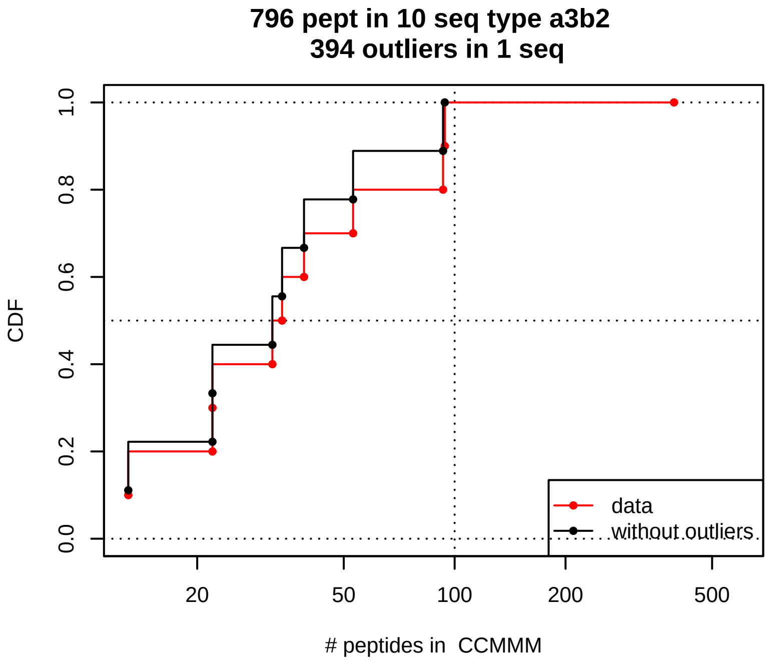
796 pept in 10 seq type a3b2
394 outliers in 1 seq
CDF
0.0
0.2
0.4
0.6
0.8
1.0
20
50
100
200
500
# peptides in CCMMM
data
without outliers

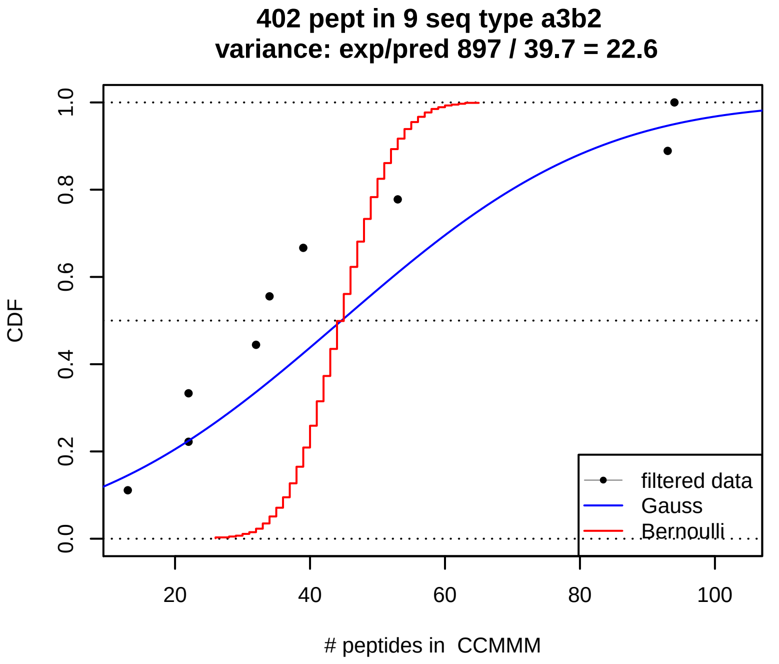
402 pept in 9 seq type a3b2
variance: exp/pred 897 / 39.7 = 22.6
CDF
0.0
0.2
0.4
0.6
0.8
1.0
20
40
60
80
100
# peptides in CCMMM
filtered data
Gauss
Bernoulli

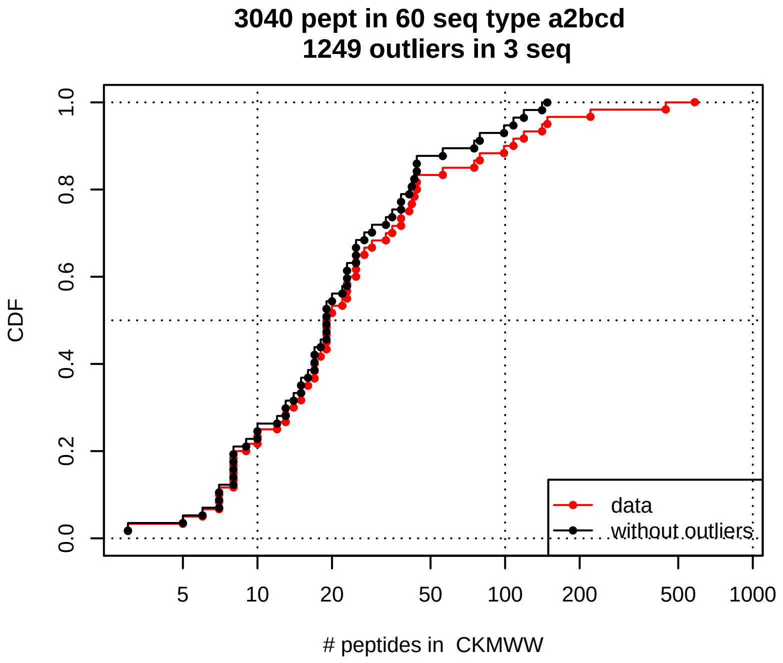
3040 pept in 60 seq type a2bcd
1249 outliers in 3 seq
CDF
# peptides in CKMWW
data
without outliers

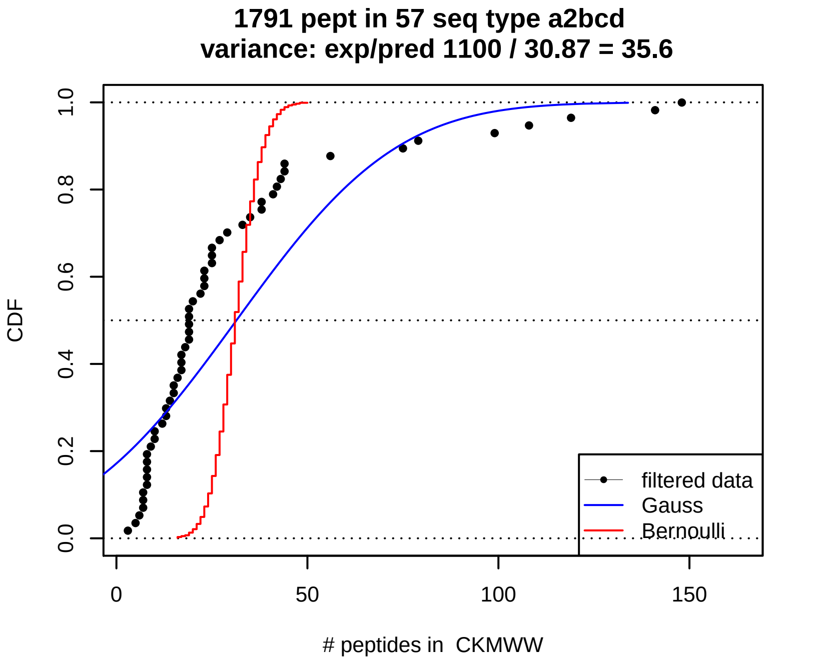
1791 pept in 57 seq type a2bcd
variance: exp/pred 1100 / 30.87 = 35.6
CDF
# peptides in CKMWW
filtered data
Gauss
Bernoulli

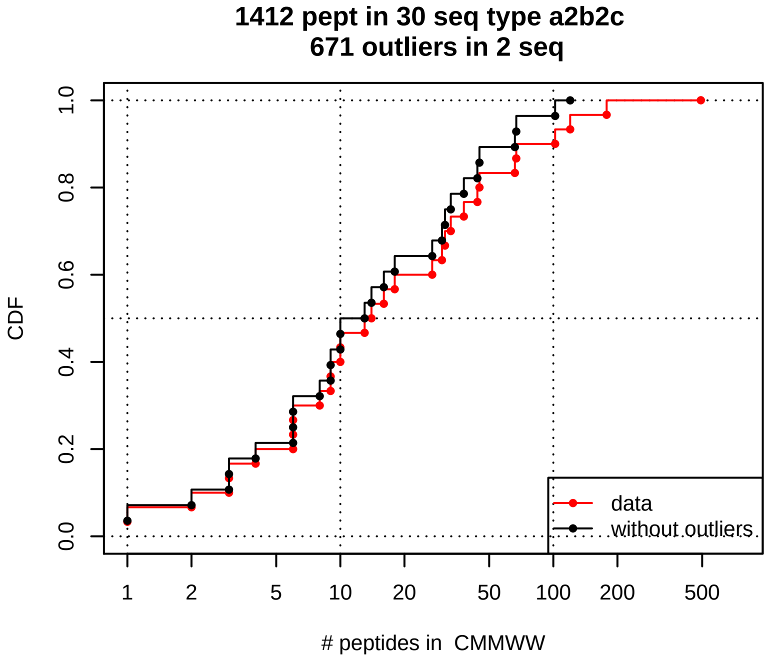
1412 pept in 30 seq type a2b2c
671 outliers in 2 seq
CDF
# peptides in CMMWW
data
without outliers

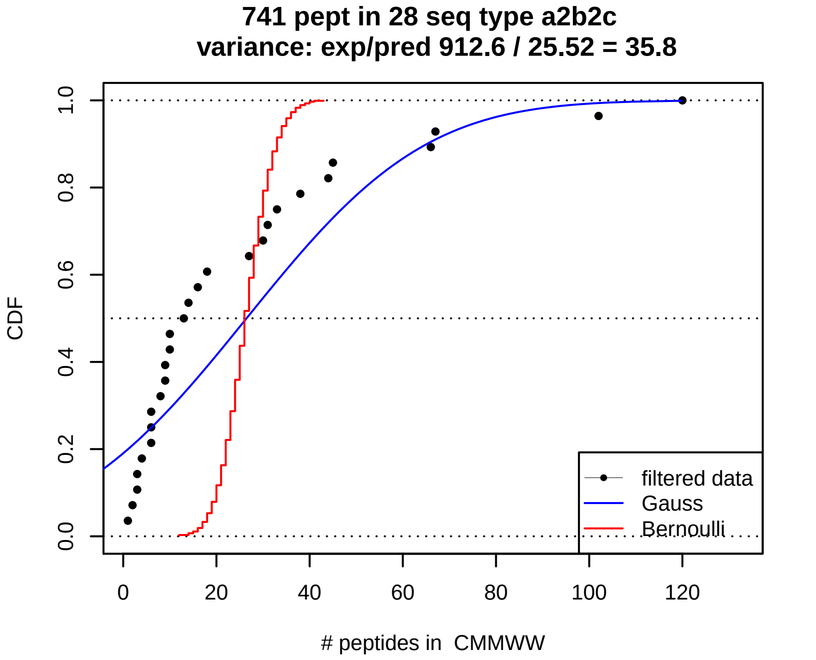
741 pept in 28 seq type a2b2c
variance: exp/pred 912.6 / 25.52 = 35.8
CDF
# peptides in CMMWW
filtered data
Gauss
Bernoulli

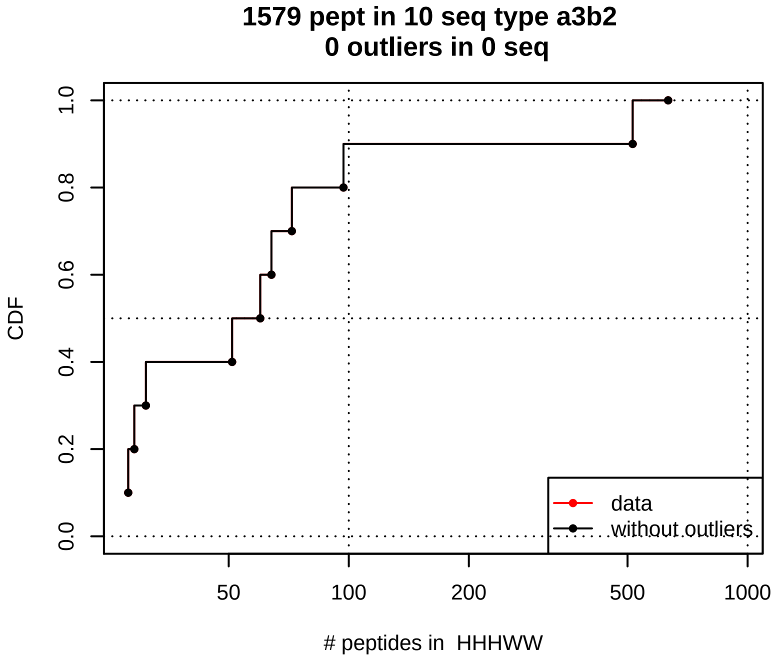
1579 pept in 10 seq type a3b2
0 outliers in 0 seq
CDF
# peptides in HHHWW
data
without outliers

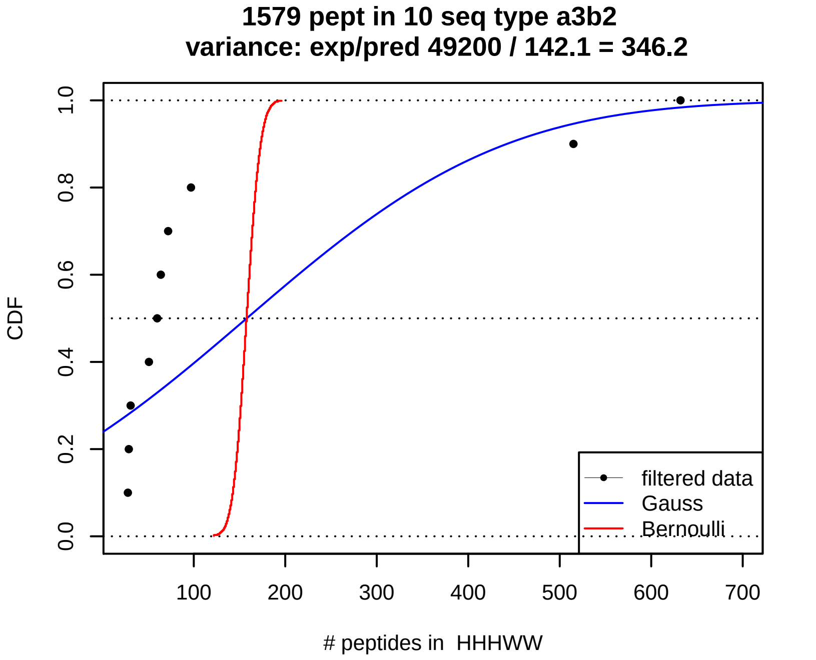
1579 pept in 10 seq type a3b2
variance: exp/pred 49200 / 142.1 = 346.2
CDF
# peptides in HHHWW
filtered data
Gauss
Bernoulli

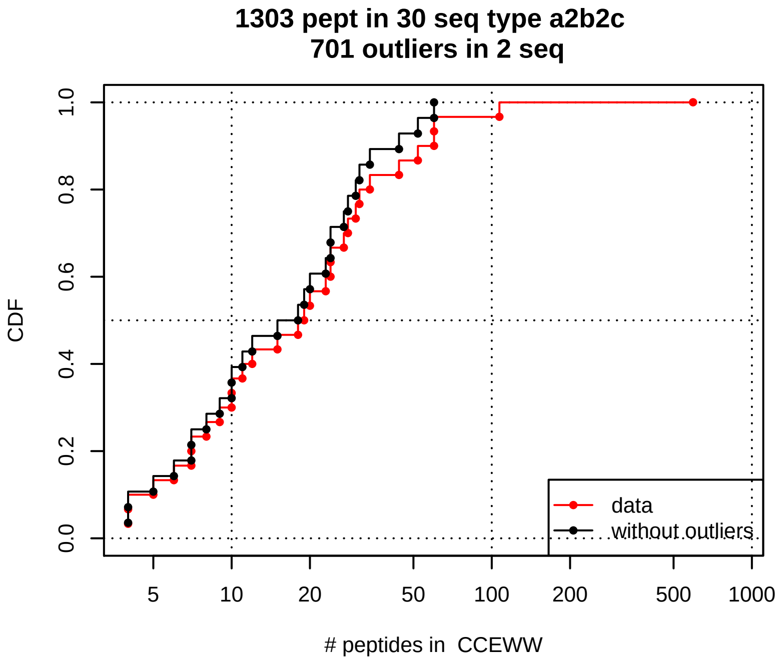
1303 pept in 30 seq type a2b2c
701 outliers in 2 seq
CDF
# peptides in CCEWW
data
without outliers

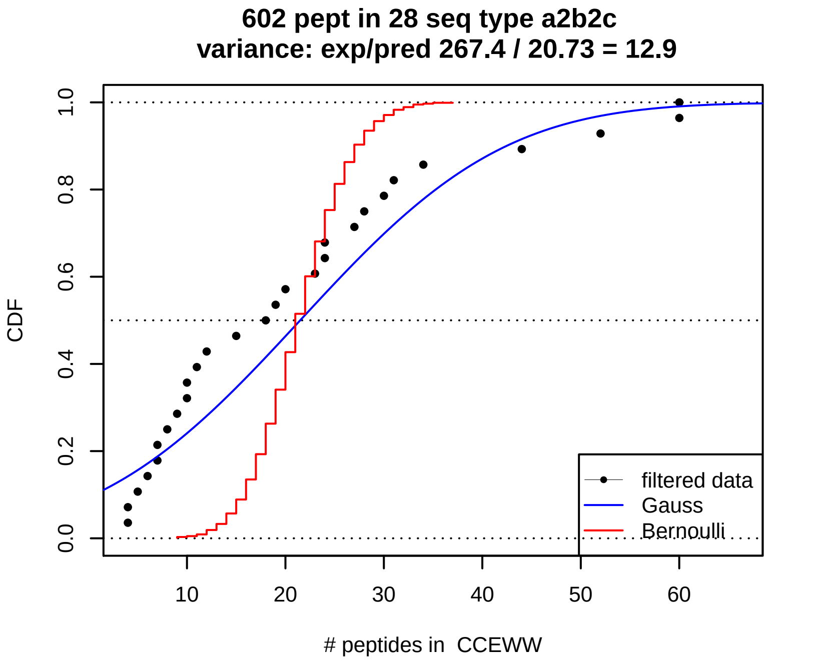
602 pept in 28 seq type a2b2c
variance: exp/pred 267.4 / 20.73 = 12.9
CDF
# peptides in CCEWW
filtered data
Gauss
Bernoulli

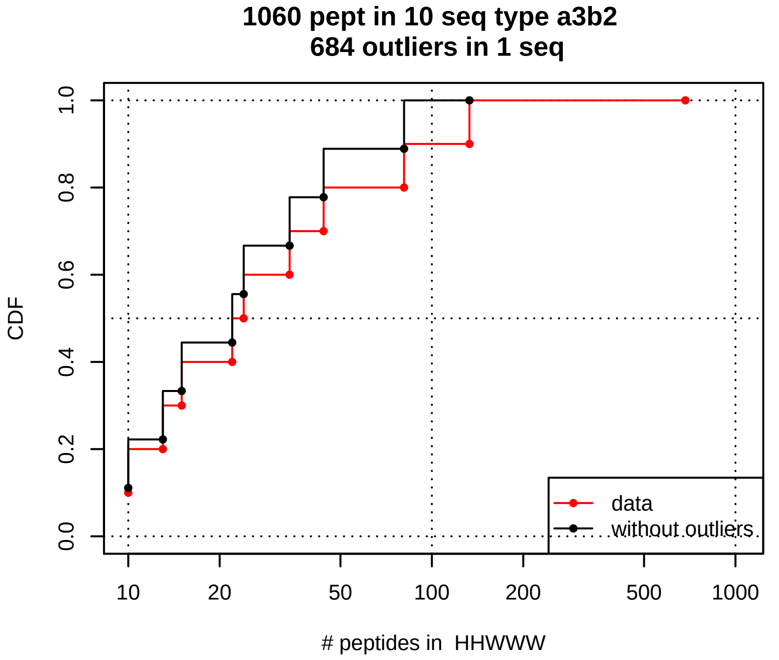
1060 pept in 10 seq type a3b2
684 outliers in 1 seq
CDF
# peptides in HHWWW
data
without outliers

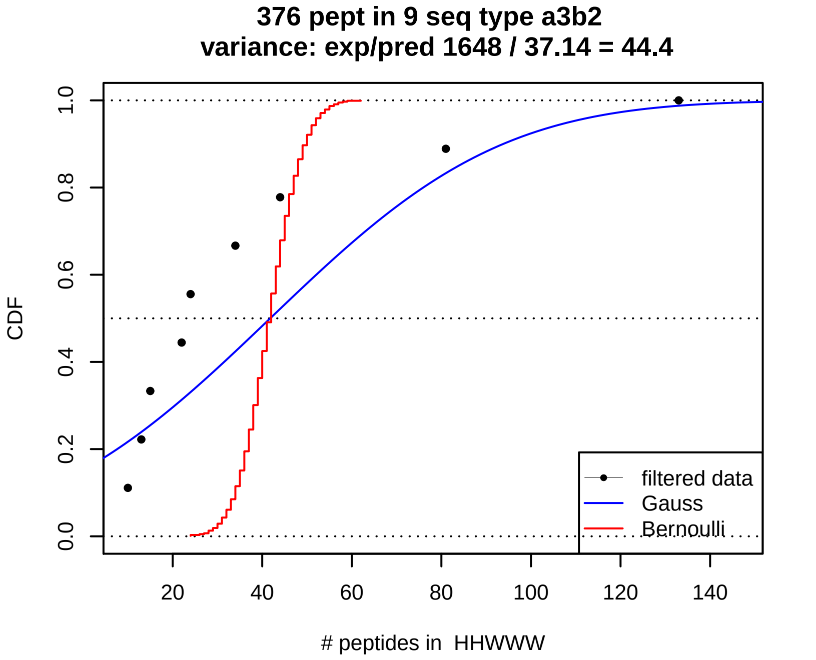
376 pept in 9 seq type a3b2
variance: exp/pred 1648 / 37.14 = 44.4
CDF
# peptides in HHWWW
filtered data
Gauss
Bernoulli

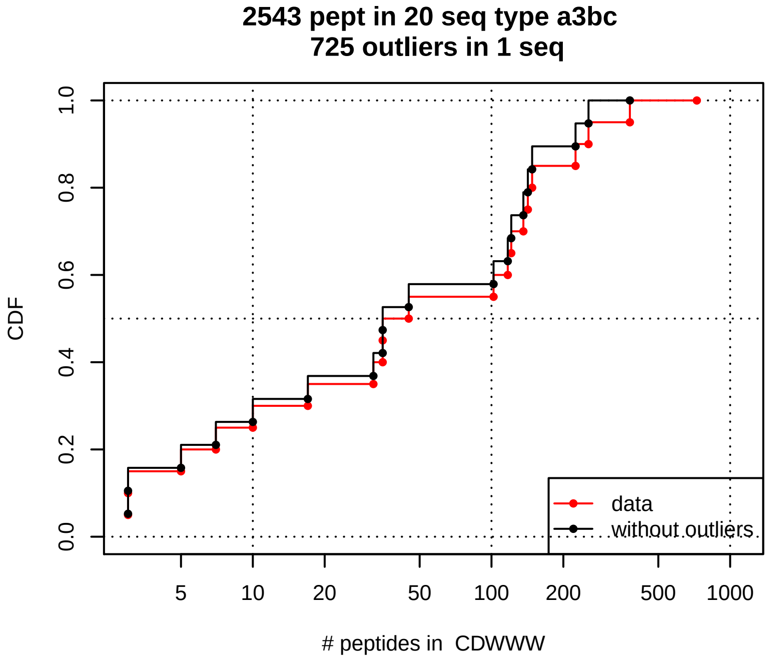
2543 pept in 20 seq type a3bc
725 outliers in 1 seq
CDF
# peptides in CDWWW
data
without outliers

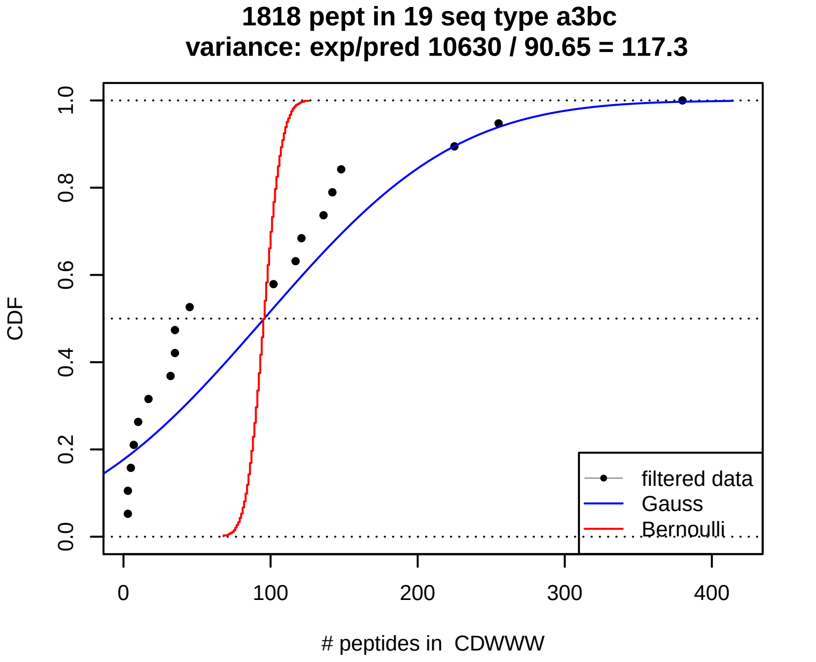
1818 pept in 19 seq type a3bc
variance: exp/pred 10630 / 90.65 = 117.3
CDF
# peptides in CDWWW
filtered data
Gauss
Bernoulli

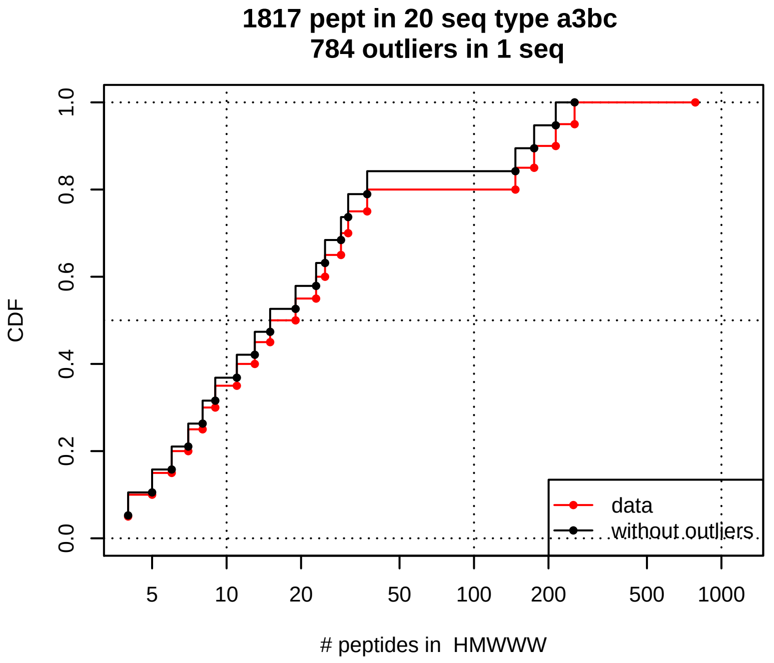
1817 pept in 20 seq type a3bc
784 outliers in 1 seq
CDF
# peptides in HMWWW
data
without outliers

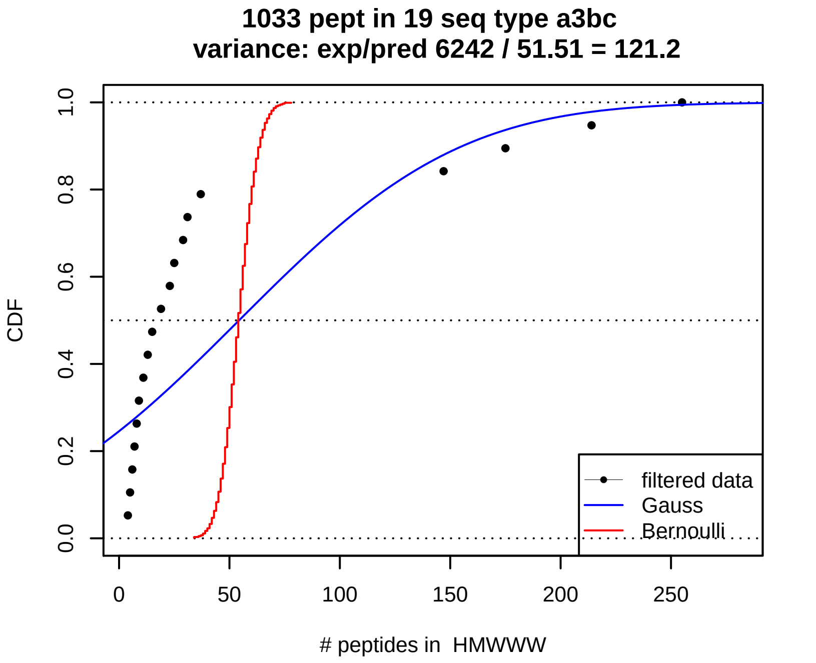
1033 pept in 19 seq type a3bc
variance: exp/pred 6242 / 51.51 = 121.2
CDF
# peptides in HMWWW
filtered data
Gauss
Bernoulli

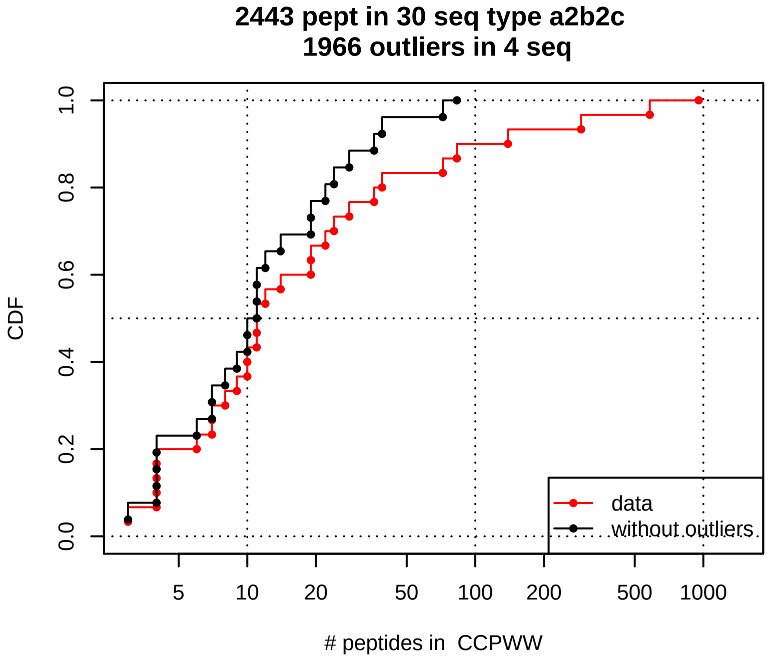
2443 pept in 30 seq type a2b2c
1966 outliers in 4 seq
CDF
# peptides in CCPWW
data
without outliers

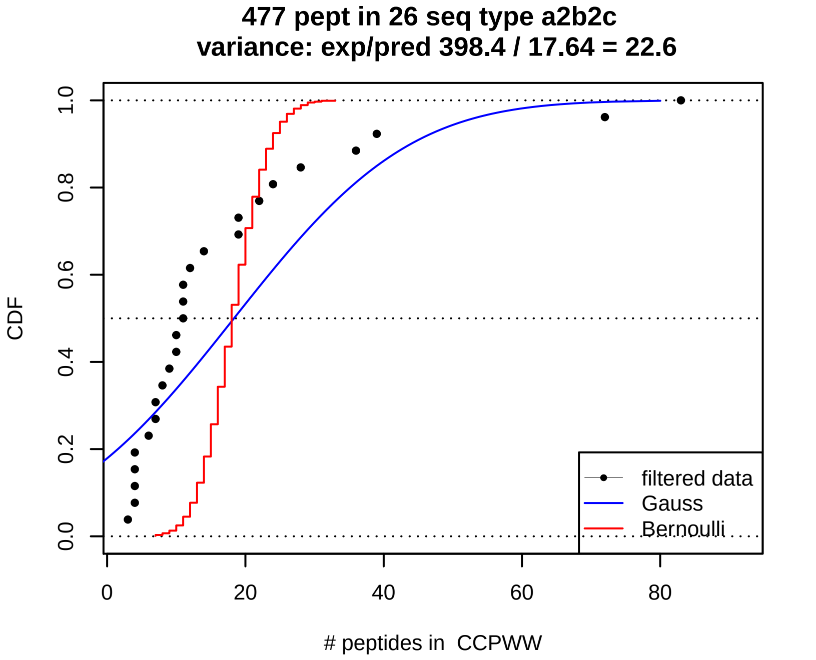
477 pept in 26 seq type a2b2c
variance: exp/pred 398.4 / 17.64 = 22.6
CDF
# peptides in CCPWW
filtered data
Gauss
Bernoulli

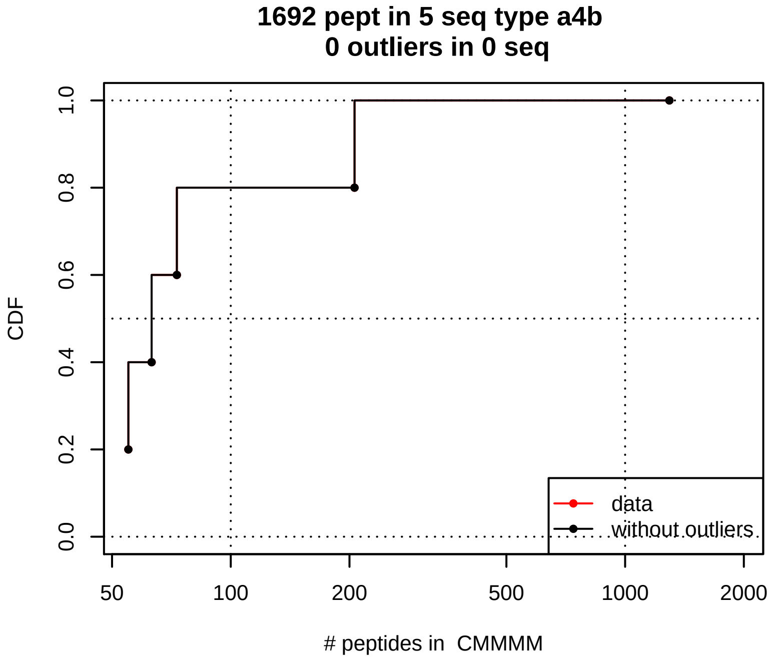
1692 pept in 5 seq type a4b
0 outliers in 0 seq
CDF
# peptides in CMMMM
data
without outliers

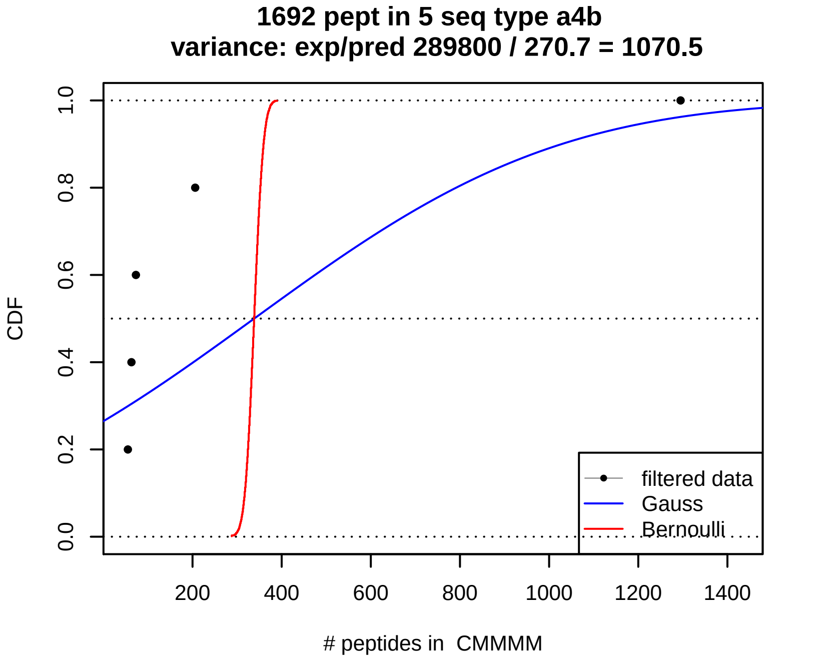
1692 pept in 5 seq type a4b
variance: exp/pred 289800 / 270.7 = 1070.5
CDF
# peptides in CMMMM
filtered data
Gauss
Bernoulli

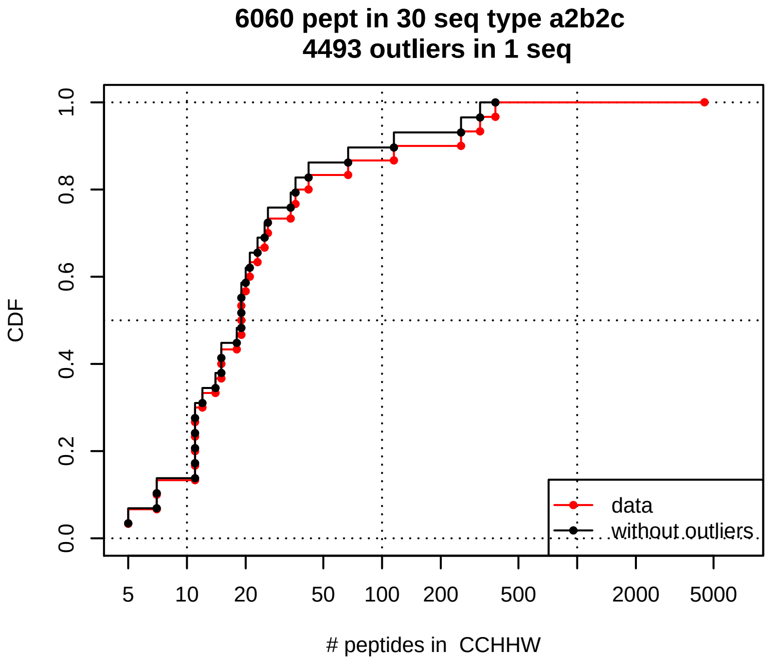
6060 pept in 30 seq type a2b2c
4493 outliers in 1 seq
CDF
# peptides in CCHHW
data
without outliers

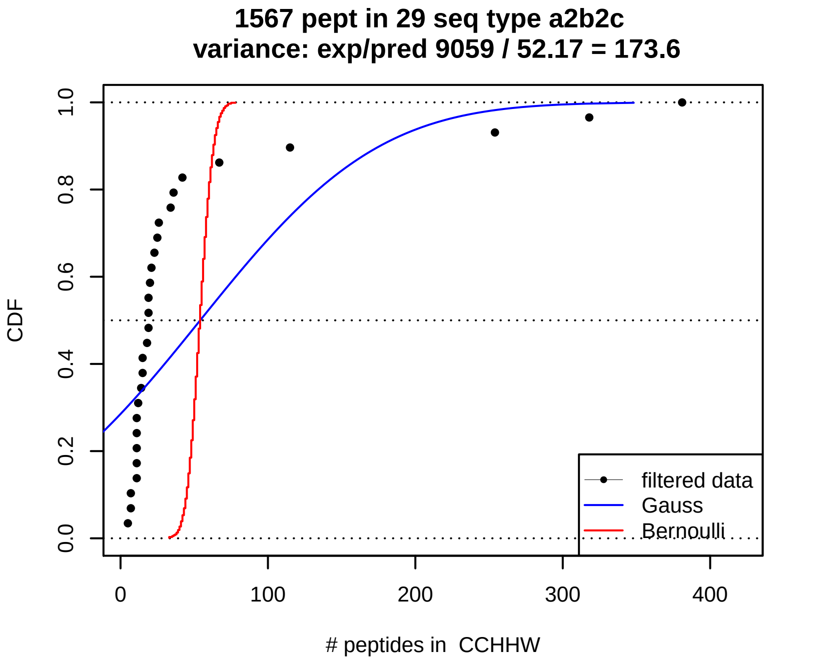
1567 pept in 29 seq type a2b2c
variance: exp/pred 9059 / 52.17 = 173.6
CDF
# peptides in CCHHW
filtered data
Gauss
Bernoulli

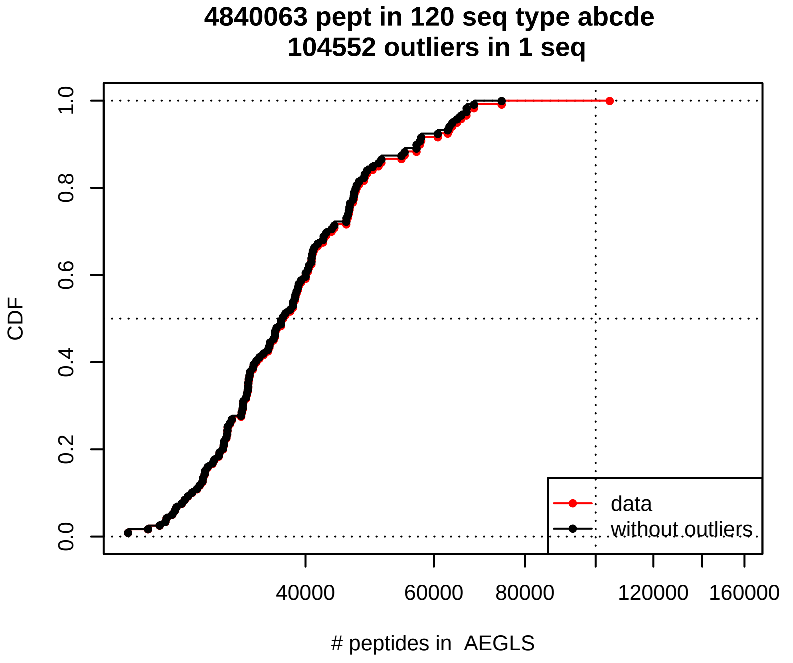
4840063 pept in 120 seq type abcde
104552 outliers in 1 seq
CDF
# peptides in AEGLS
data
without outliers

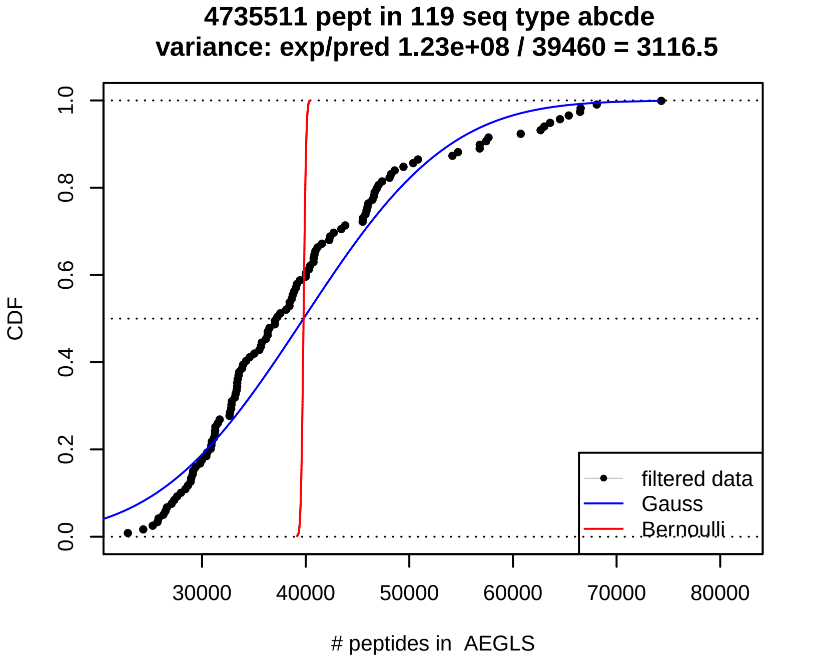
4735511 pept in 119 seq type abcde
variance: exp/pred 1.23e+08 / 39460 = 3116.5
CDF
# peptides in AEGLS
filtered data
Gauss
Bernoulli

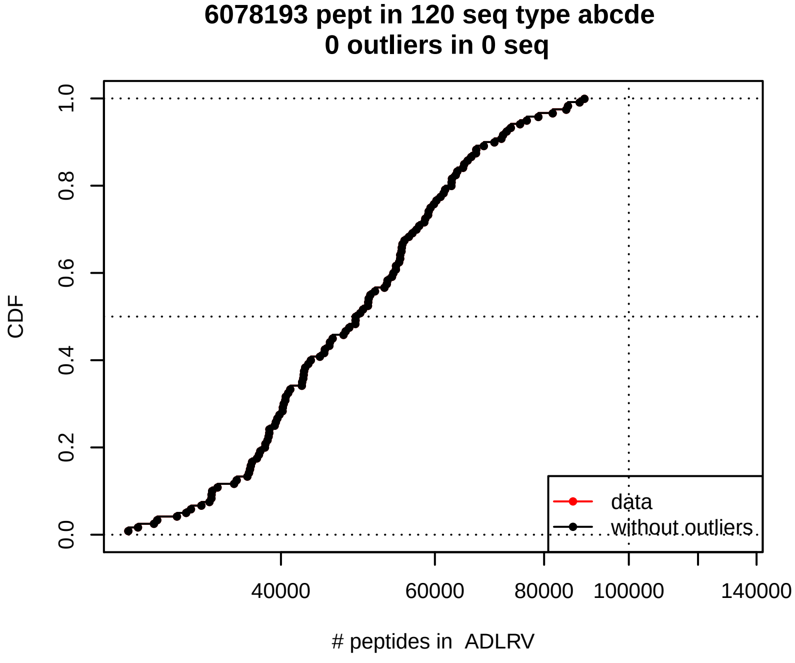
6078193 pept in 120 seq type abcde
0 outliers in 0 seq
CDF
# peptides in ADLRV
data
without outliers

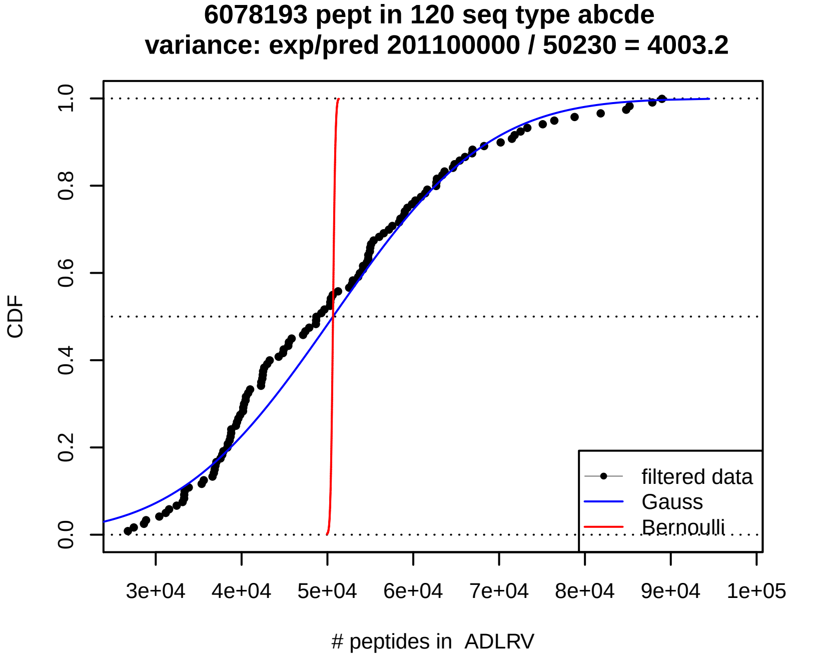
6078193 pept in 120 seq type abcde
variance: exp/pred 201100000 / 50230 = 4003.2
CDF
# peptides in ADLRV
filtered data
Gauss
Bernoulli

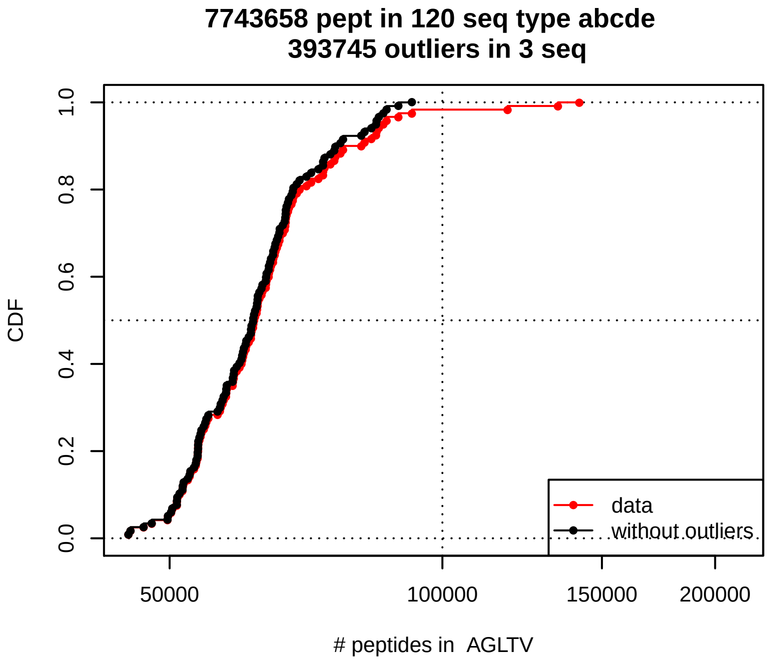
7743658 pept in 120 seq type abcde
393745 outliers in 3 seq
CDF
# peptides in AGLTV
data
without outliers
0.0
0.2
0.4
0.6
0.8
1.0
50000
100000
150000
200000

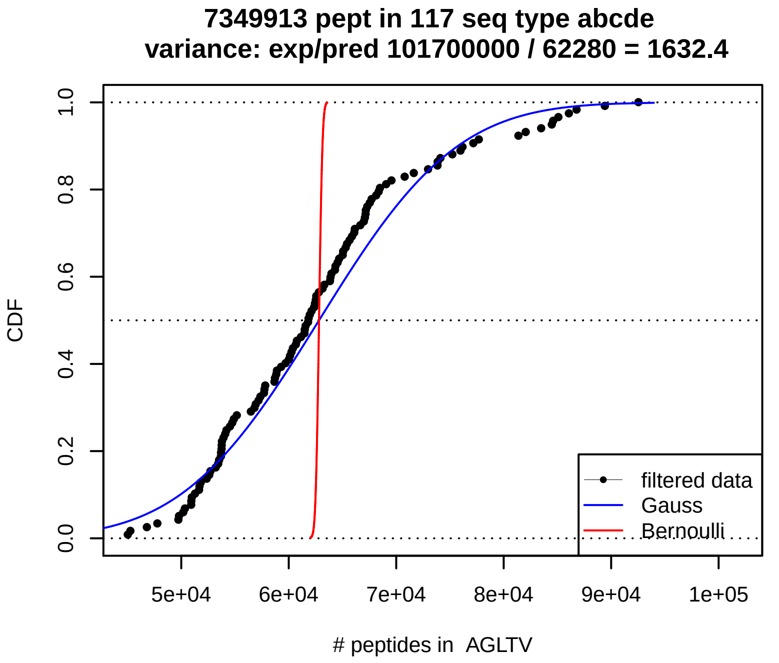
7349913 pept in 117 seq type abcde
variance: exp/pred 101700000 / 62280 = 1632.4
CDF
# peptides in AGLTV
filtered data
Gauss
Bernoulli
0.0
0.2
0.4
0.6
0.8
1.0
5e+04
6e+04
7e+04
8e+04
9e+04
1e+05

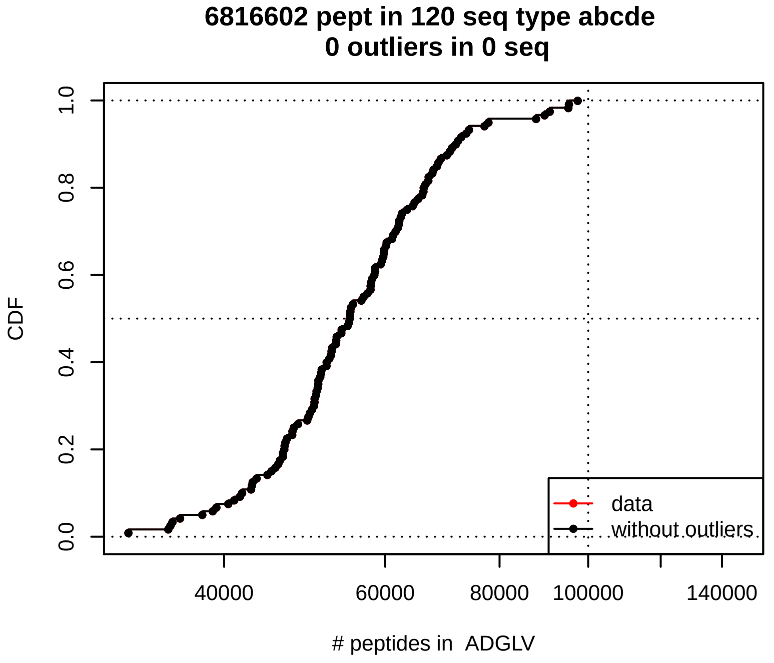
6816602 pept in 120 seq type abcde
0 outliers in 0 seq
CDF
# peptides in ADGLV
data
without outliers
0.0
0.2
0.4
0.6
0.8
1.0
40000
60000
80000
100000
140000

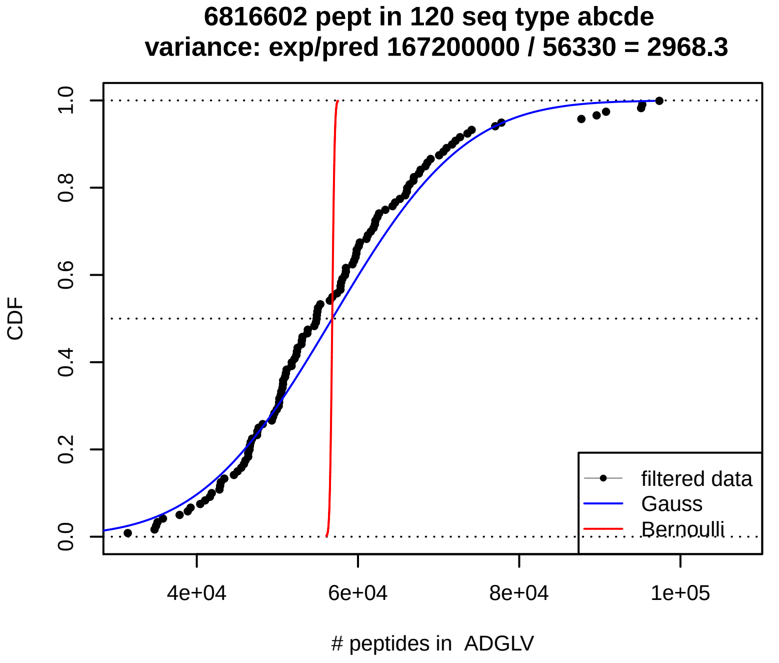
6816602 pept in 120 seq type abcde
variance: exp/pred 167200000 / 56330 = 2968.3
CDF
# peptides in ADGLV
filtered data
Gauss
Bernoulli
0.0
0.2
0.4
0.6
0.8
1.0
4e+04
6e+04
8e+04
1e+05

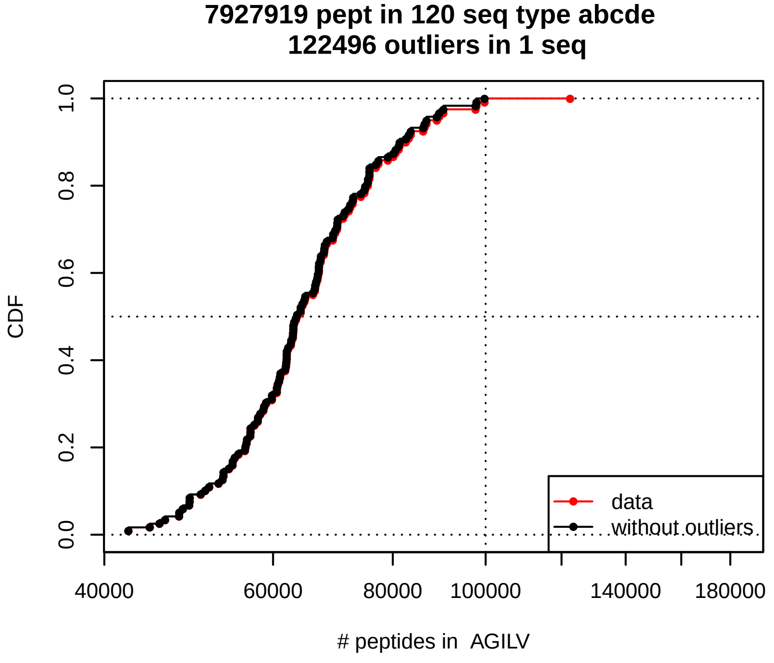
7927919 pept in 120 seq type abcde
122496 outliers in 1 seq
CDF
# peptides in AGILV
data
without outliers
0.0
0.2
0.4
0.6
0.8
1.0
40000
60000
80000
100000
140000
180000

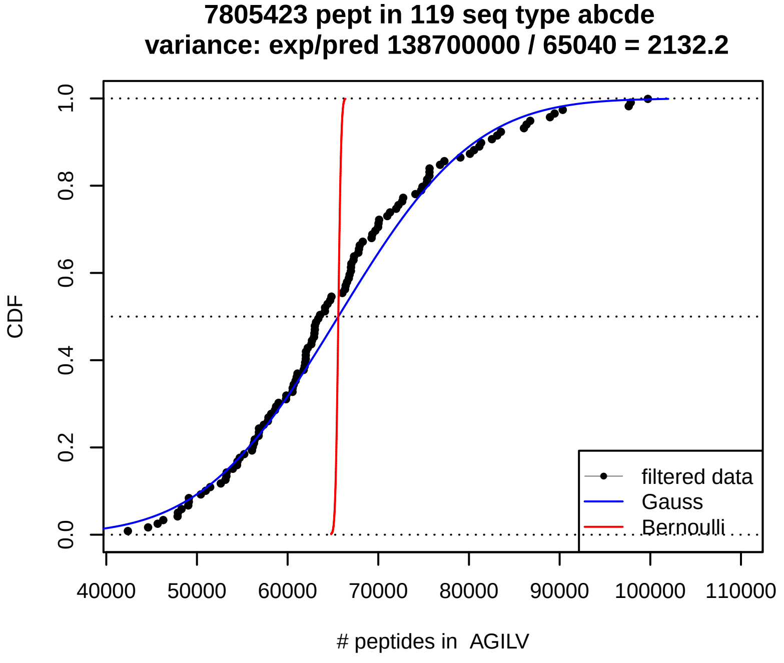
7805423 pept in 119 seq type abcde
variance: exp/pred 138700000 / 65040 = 2132.2
CDF
# peptides in AGILV
filtered data
Gauss
Bernoulli
0.0
0.2
0.4
0.6
0.8
1.0
40000
50000
60000
70000
80000
90000
100000
110000

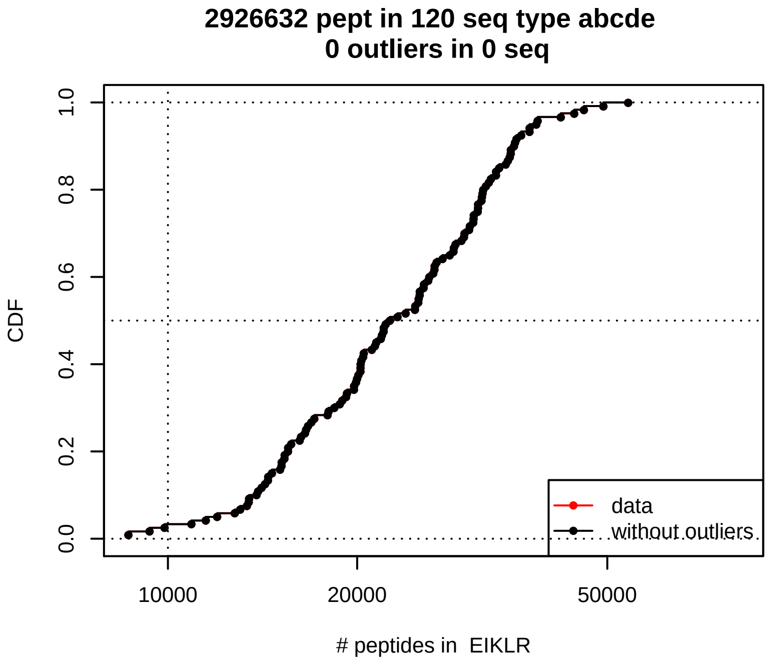
2926632 pept in 120 seq type abcde
0 outliers in 0 seq
CDF
0.0
0.2
0.4
0.6
0.8
1.0
10000
20000
50000
# peptides in EIKLR
data
without outliers

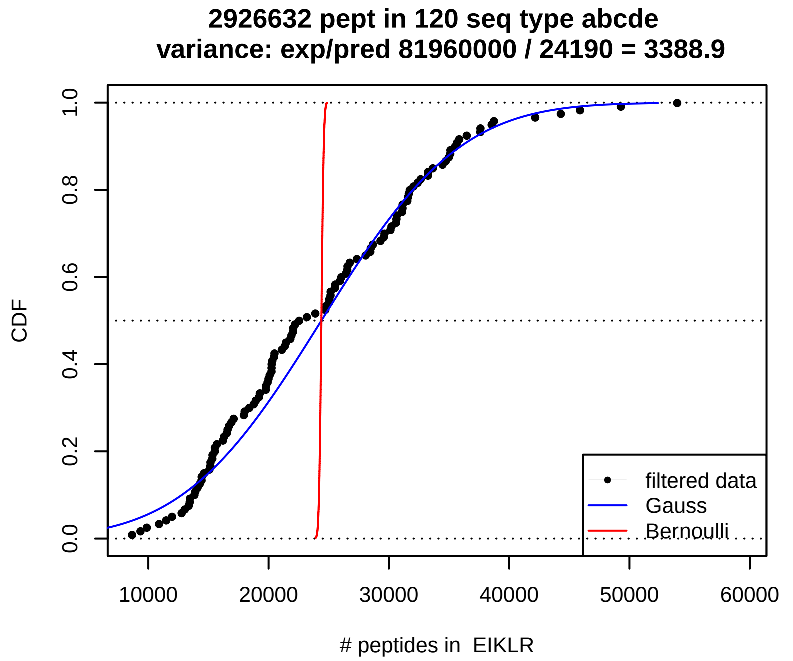
2926632 pept in 120 seq type abcde
variance: exp/pred 81960000 / 24190 = 3388.9
CDF
0.0
0.2
0.4
0.6
0.8
1.0
10000
20000
30000
40000
50000
60000
# peptides in EIKLR
filtered data
Gauss
Bernoulli

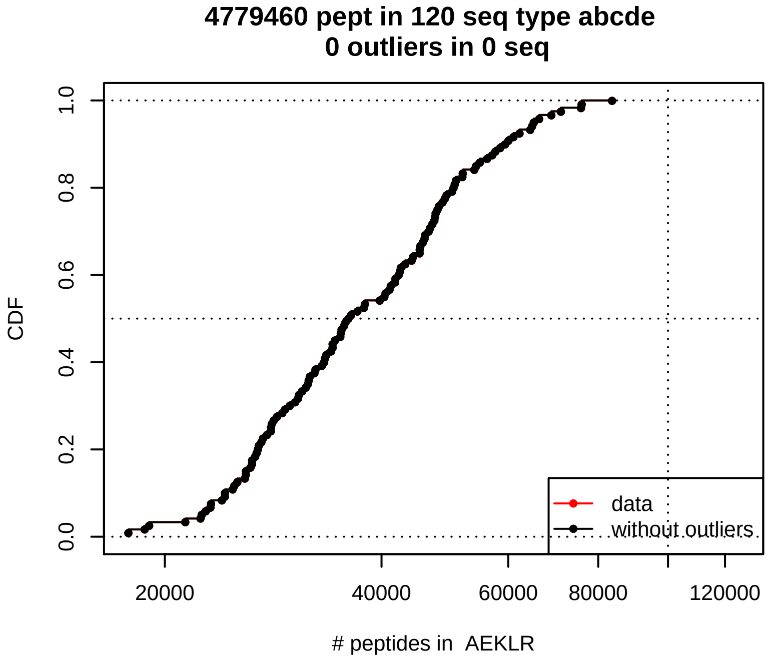
4779460 pept in 120 seq type abcde
0 outliers in 0 seq
CDF
0.0
0.2
0.4
0.6
0.8
1.0
20000
40000
60000
80000
120000
# peptides in AEKLR
data
without outliers

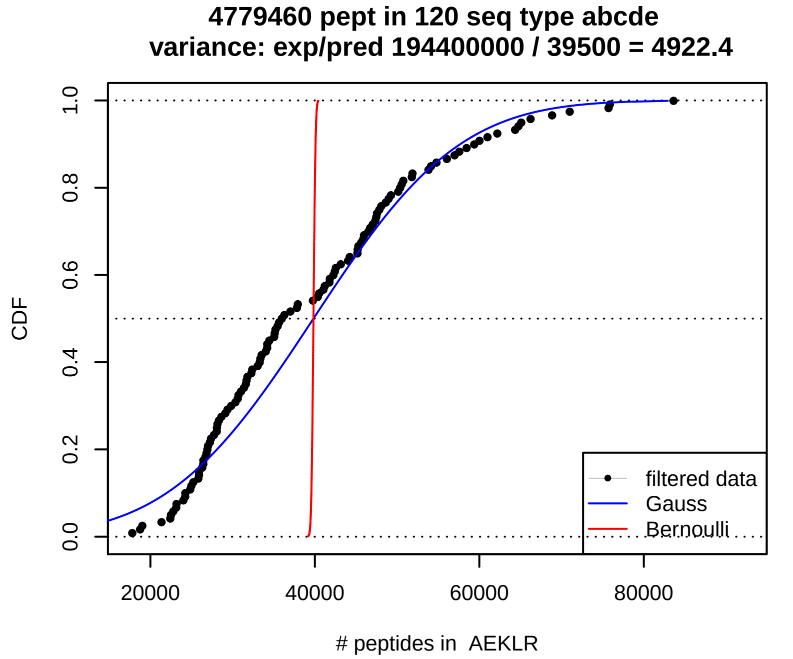
4779460 pept in 120 seq type abcde
variance: exp/pred 194400000 / 39500 = 4922.4
CDF
0.0
0.2
0.4
0.6
0.8
1.0
20000
40000
60000
80000
# peptides in AEKLR
filtered data
Gauss
Bernoulli

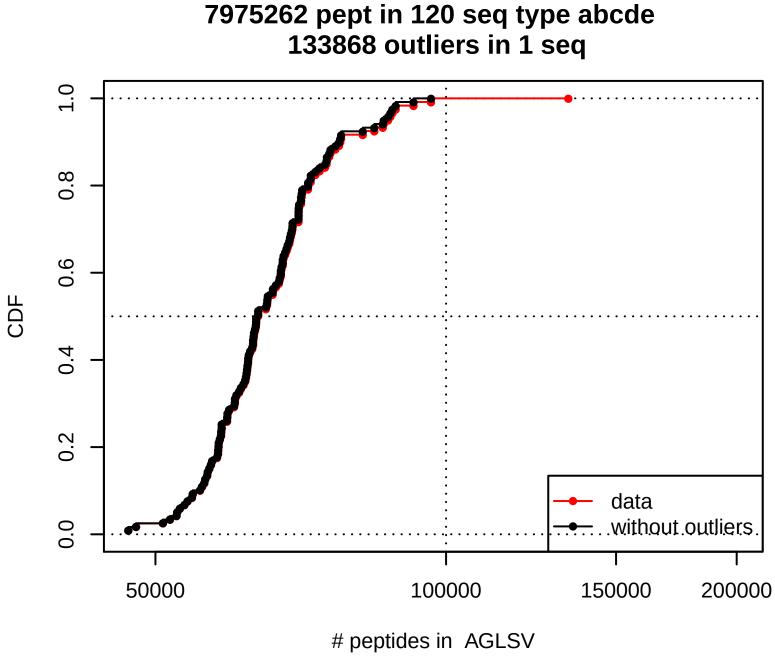
7975262 pept in 120 seq type abcde
133868 outliers in 1 seq
CDF
0.0
0.2
0.4
0.6
0.8
1.0
50000
100000
150000
200000
# peptides in AGLSV
data
without outliers

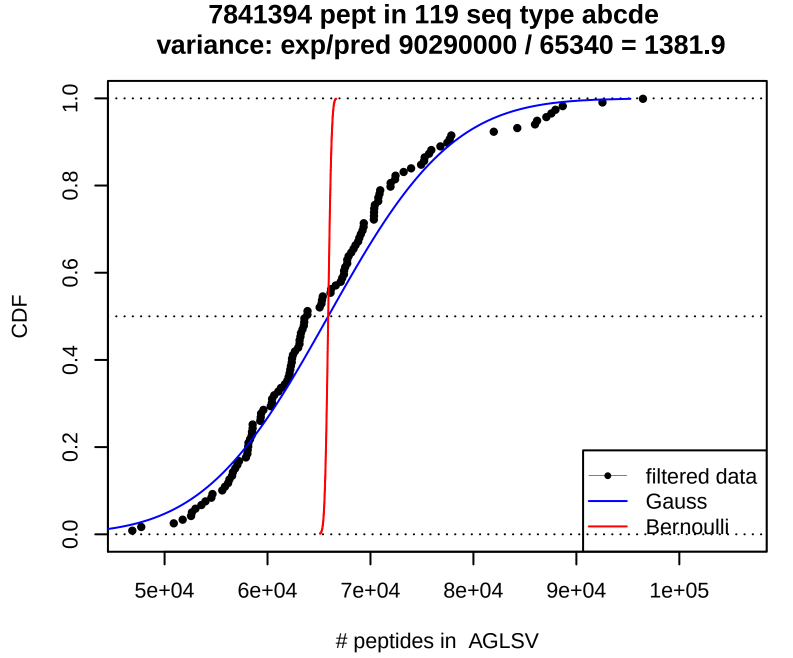
7841394 pept in 119 seq type abcde
variance: exp/pred 90290000 / 65340 = 1381.9
CDF
0.0
0.2
0.4
0.6
0.8
1.0
5e+04
6e+04
7e+04
8e+04
9e+04
1e+05
# peptides in AGLSV
filtered data
Gauss
Bernoulli

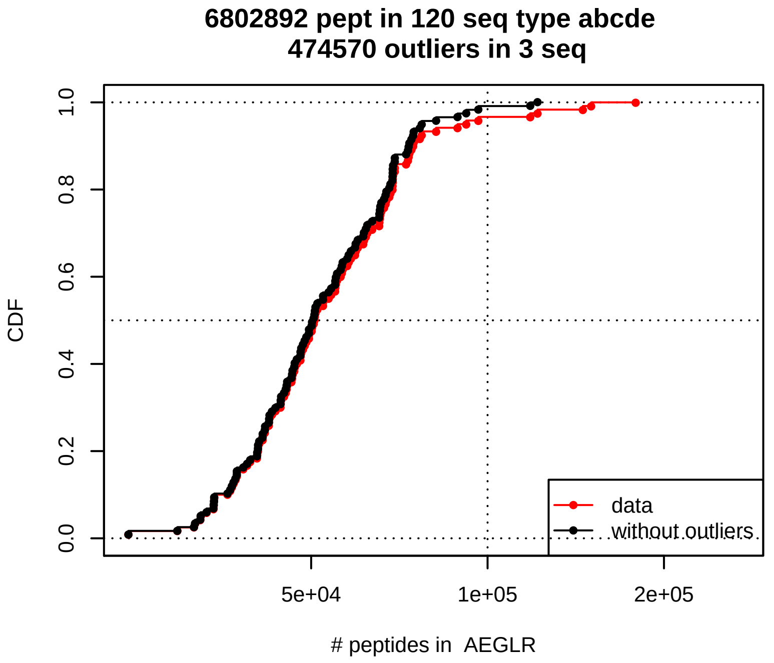
6802892 pept in 120 seq type abcde
474570 outliers in 3 seq
CDF
0.0
0.2
0.4
0.6
0.8
1.0
5e+04
1e+05
2e+05
# peptides in AEGLR
data
without outliers

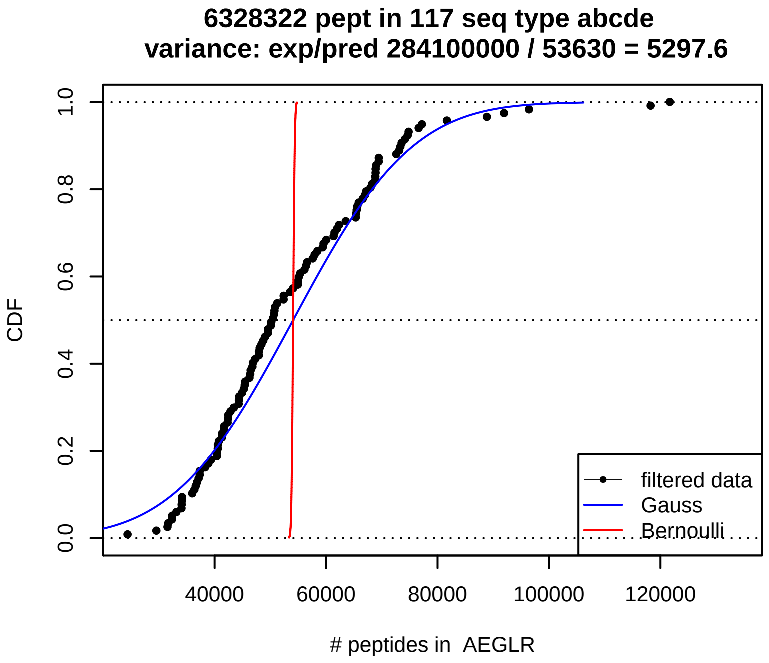
6328322 pept in 117 seq type abcde
variance: exp/pred 284100000 / 53630 = 5297.6
CDF
0.0
0.2
0.4
0.6
0.8
1.0
40000
60000
80000
100000
120000
# peptides in AEGLR
filtered data
Gauss
Bernoulli

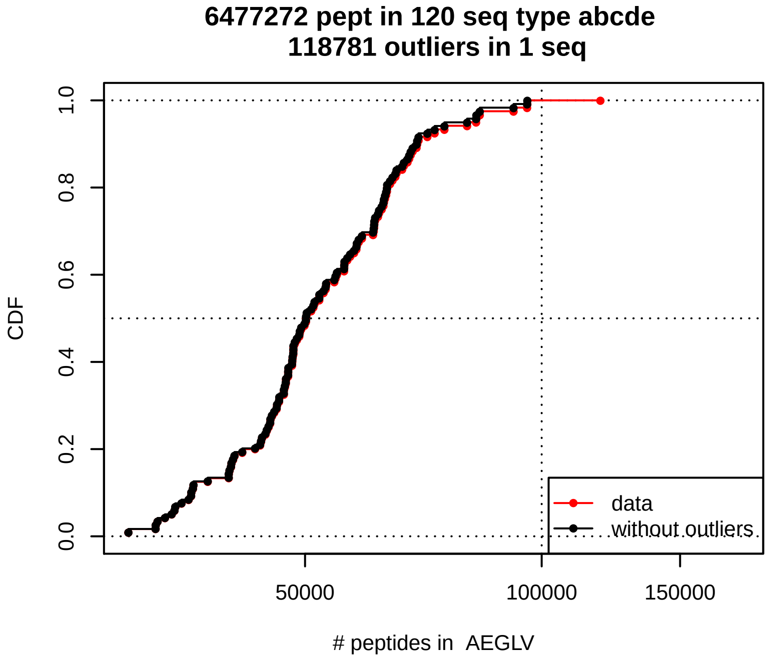
6477272 pept in 120 seq type abcde
118781 outliers in 1 seq
CDF
0.0
0.2
0.4
0.6
0.8
1.0
50000
100000
150000
# peptides in AEGLV
data
without outliers

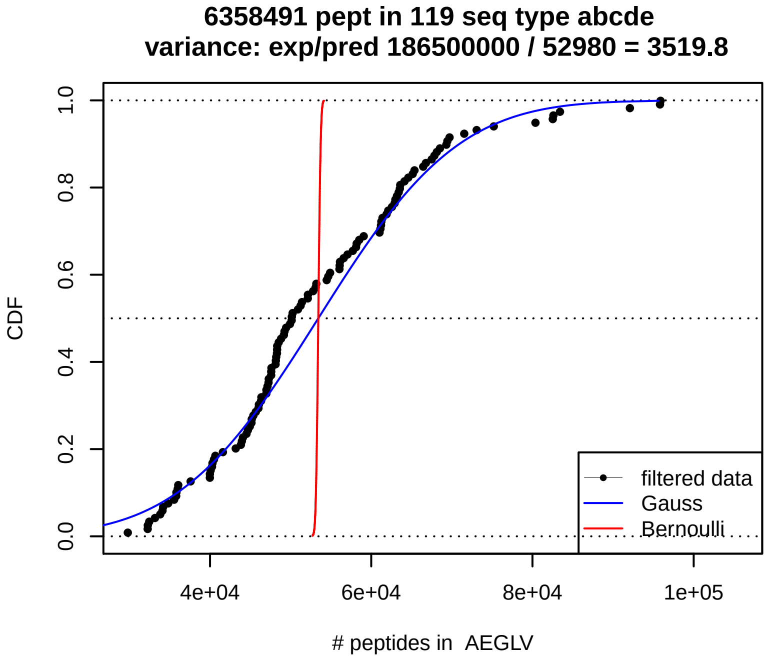
6358491 pept in 119 seq type abcde
variance: exp/pred 186500000 / 52980 = 3519.8
CDF
0.0
0.2
0.4
0.6
0.8
1.0
4e+04
6e+04
8e+04
1e+05
# peptides in AEGLV
filtered data
Gauss
Bernoulli

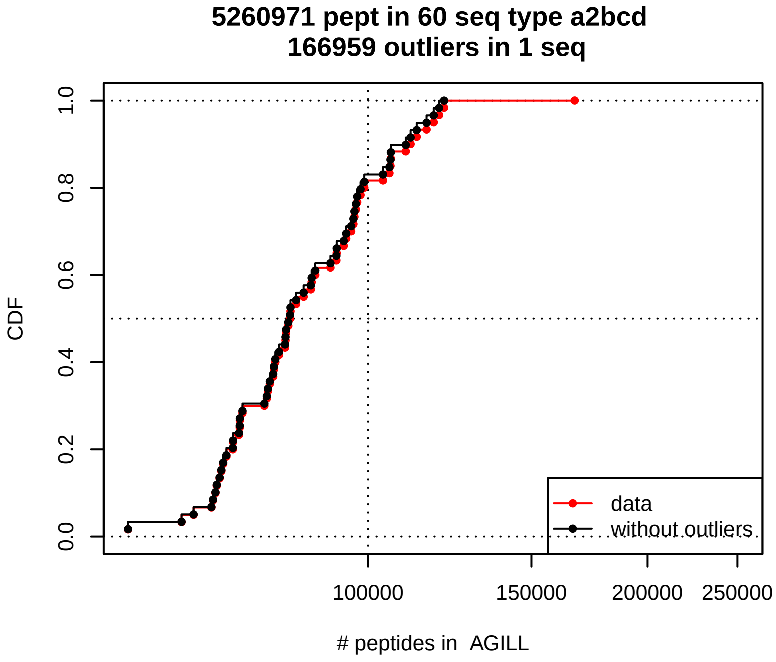
5260971 pept in 60 seq type a2bcd
166959 outliers in 1 seq
CDF
0.0
0.2
0.4
0.6
0.8
1.0
100000
150000
200000
250000
# peptides in AGILL
data
without outliers

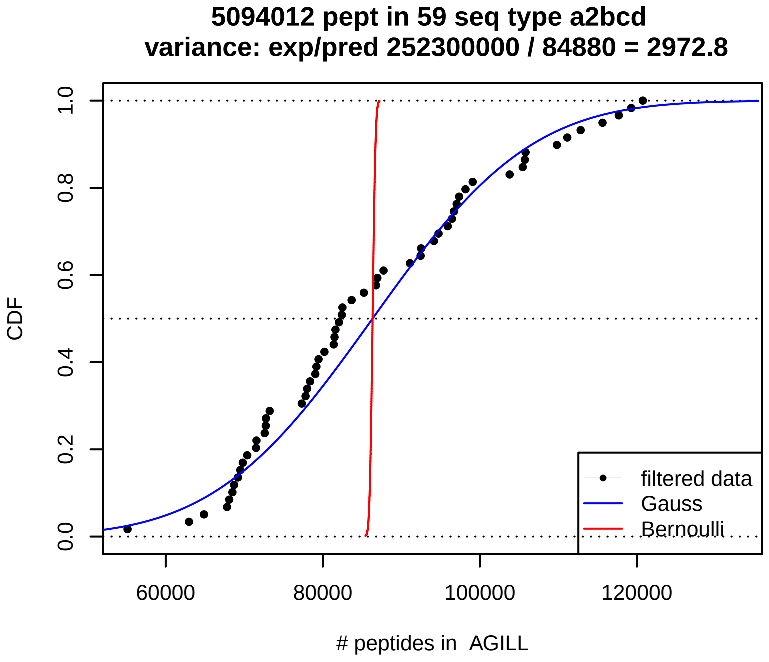
5094012 pept in 59 seq type a2bcd
variance: exp/pred 252300000 / 84880 = 2972.8
CDF
0.0
0.2
0.4
0.6
0.8
1.0
60000
80000
100000
120000
# peptides in AGILL
filtered data
Gauss
Bernoulli

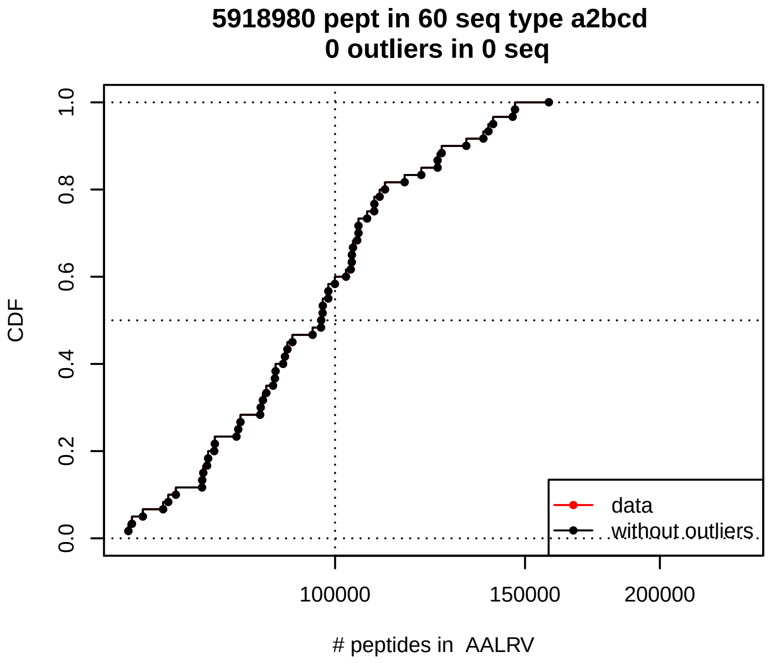
5918980 pept in 60 seq type a2bcd
0 outliers in 0 seq
CDF
0.0
0.2
0.4
0.6
0.8
1.0
100000
150000
200000
# peptides in AALRV
data
without outliers

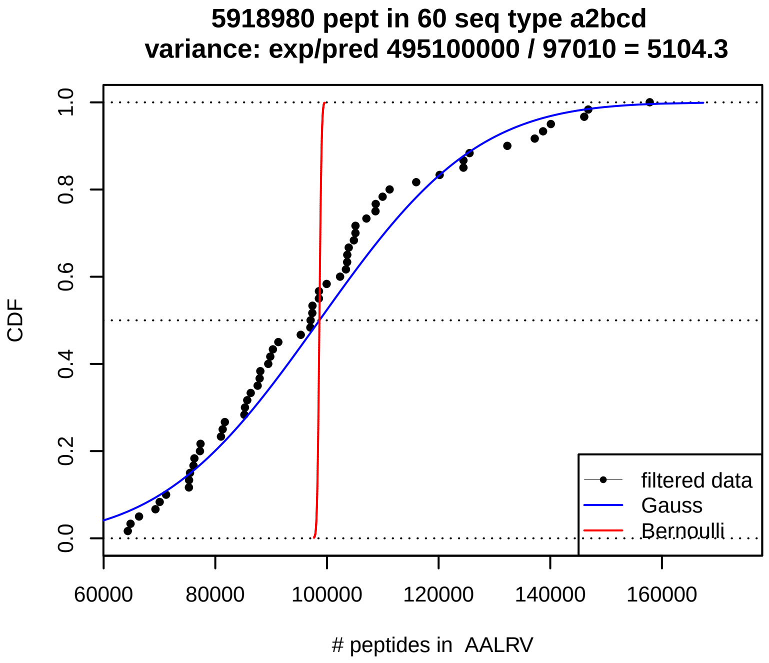
5918980 pept in 60 seq type a2bcd
variance: exp/pred 495100000 / 97010 = 5104.3
CDF
0.0
0.2
0.4
0.6
0.8
1.0
60000
80000
100000
120000
140000
160000
# peptides in AALRV
filtered data
Gauss
Bernoulli

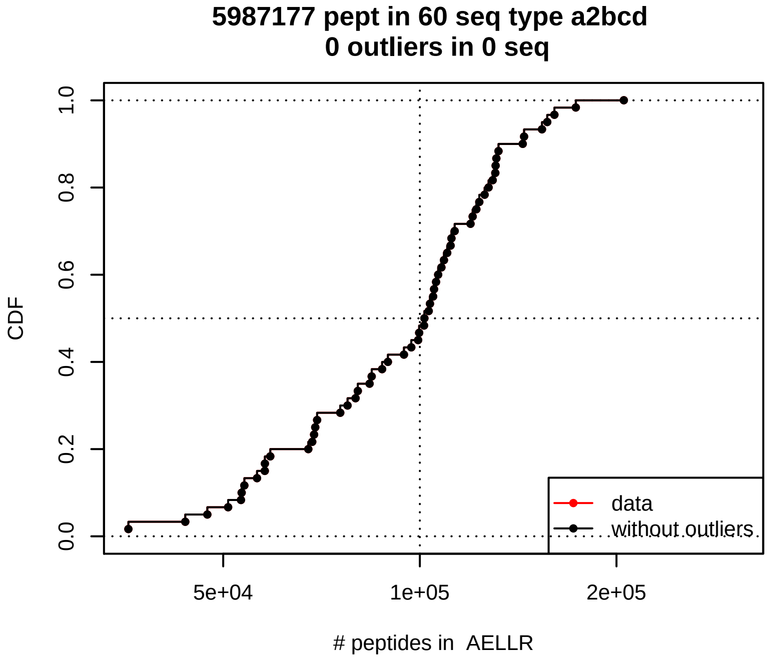
5987177 pept in 60 seq type a2bcd
0 outliers in 0 seq
CDF
0.0
0.2
0.4
0.6
0.8
1.0
5e+04
1e+05
2e+05
# peptides in AELLR
data
without outliers

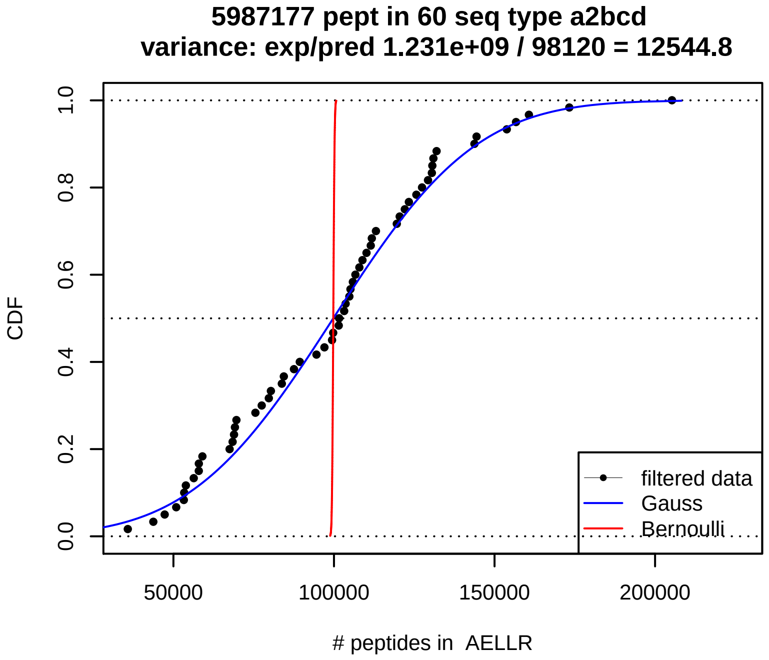
5987177 pept in 60 seq type a2bcd
variance: exp/pred 1.231e+09 / 98120 = 12544.8
CDF
0.0
0.2
0.4
0.6
0.8
1.0
50000
100000
150000
200000
# peptides in AELLR
filtered data
Gauss
Bernoulli

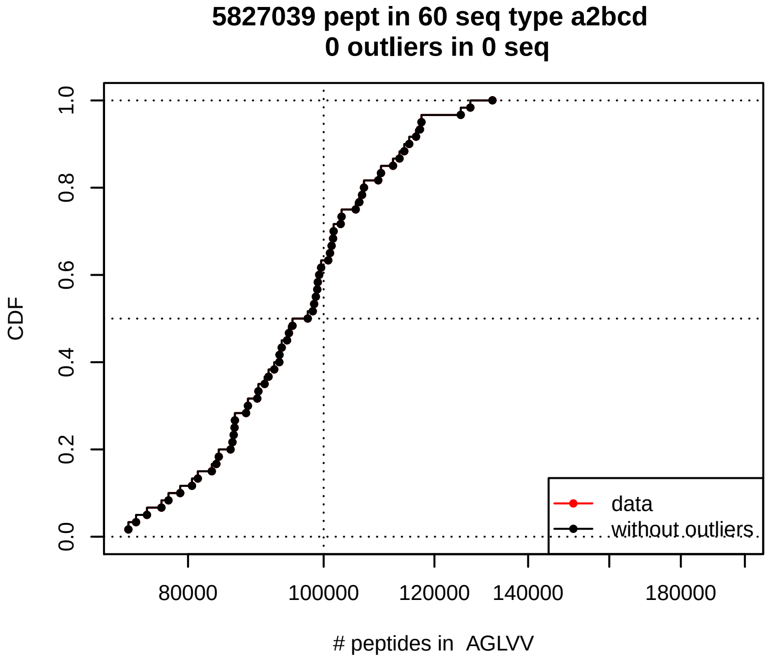
5827039 pept in 60 seq type a2bcd
0 outliers in 0 seq
CDF
0.0
0.2
0.4
0.6
0.8
1.0
80000
100000
120000
140000
180000
# peptides in AGLVV
data
without outliers

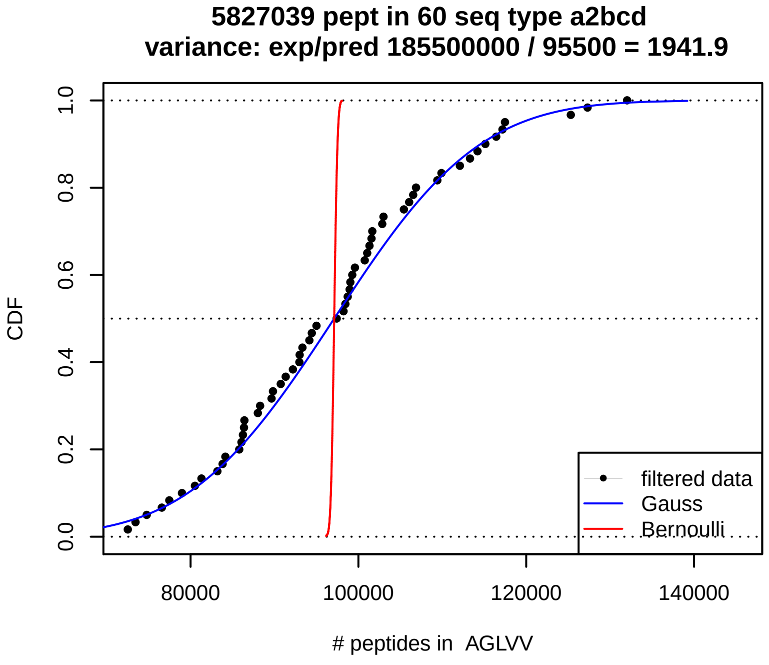
5827039 pept in 60 seq type a2bcd
variance: exp/pred 185500000 / 95500 = 1941.9
CDF
0.0
0.2
0.4
0.6
0.8
1.0
80000
100000
120000
140000
# peptides in AGLVV
filtered data
Gauss
Bernoulli

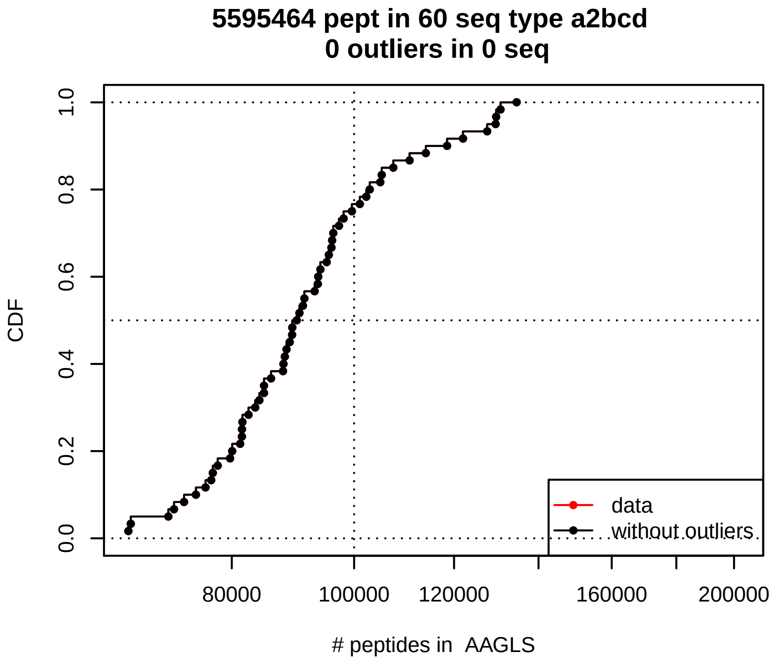
5595464 pept in 60 seq type a2bcd
0 outliers in 0 seq
CDF
# peptides in AAGLS
data
without outliers

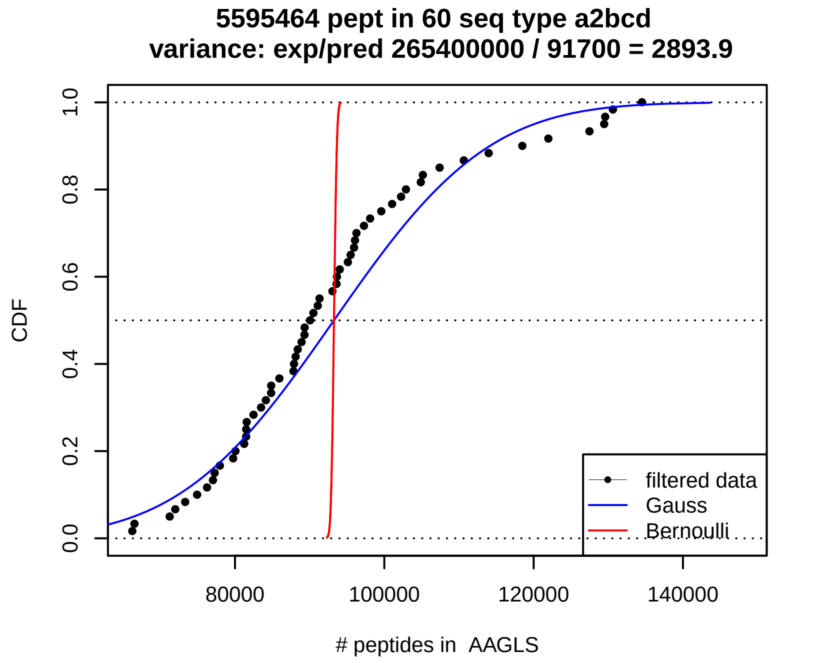
5595464 pept in 60 seq type a2bcd
variance: exp/pred 265400000 / 91700 = 2893.9
CDF
# peptides in AAGLS
filtered data
Gauss
Bernoulli

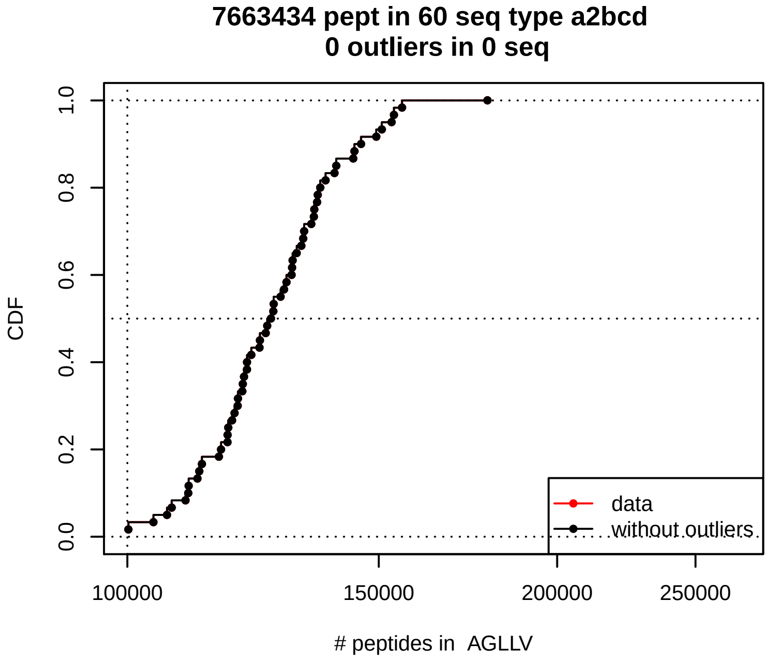
7663434 pept in 60 seq type a2bcd
0 outliers in 0 seq
CDF
# peptides in AGLLV
data
without outliers

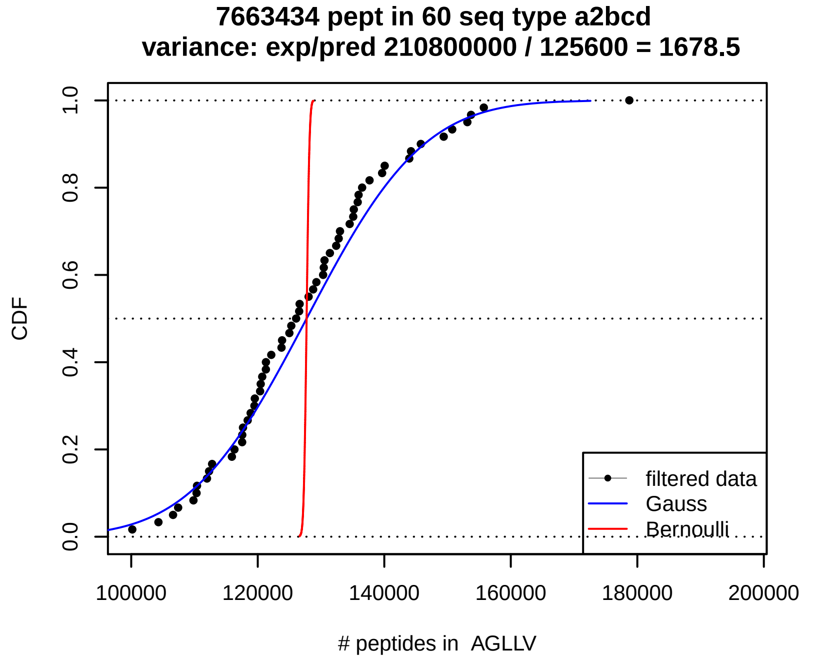
7663434 pept in 60 seq type a2bcd
variance: exp/pred 210800000 / 125600 = 1678.5
CDF
# peptides in AGLLV
filtered data
Gauss
Bernoulli

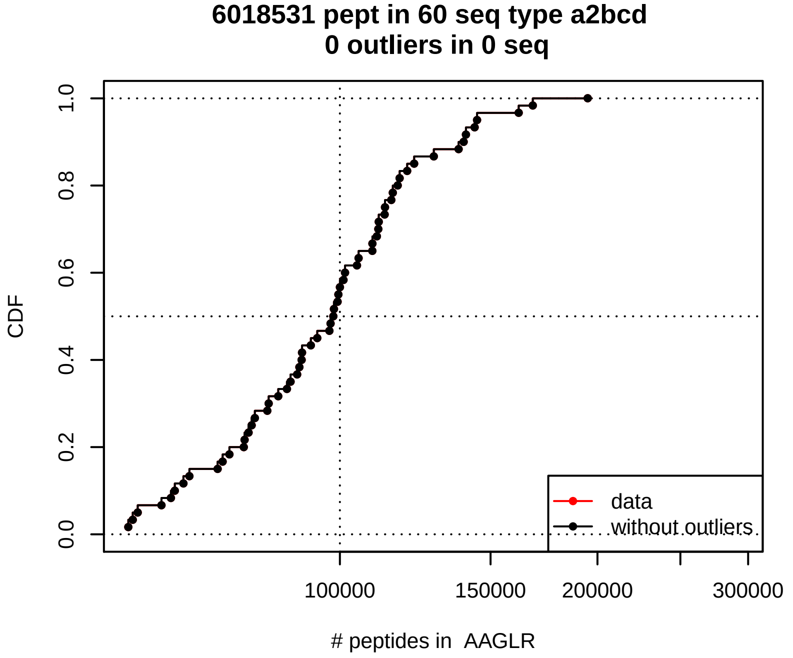
6018531 pept in 60 seq type a2bcd
0 outliers in 0 seq
CDF
# peptides in AAGLR
data
without outliers

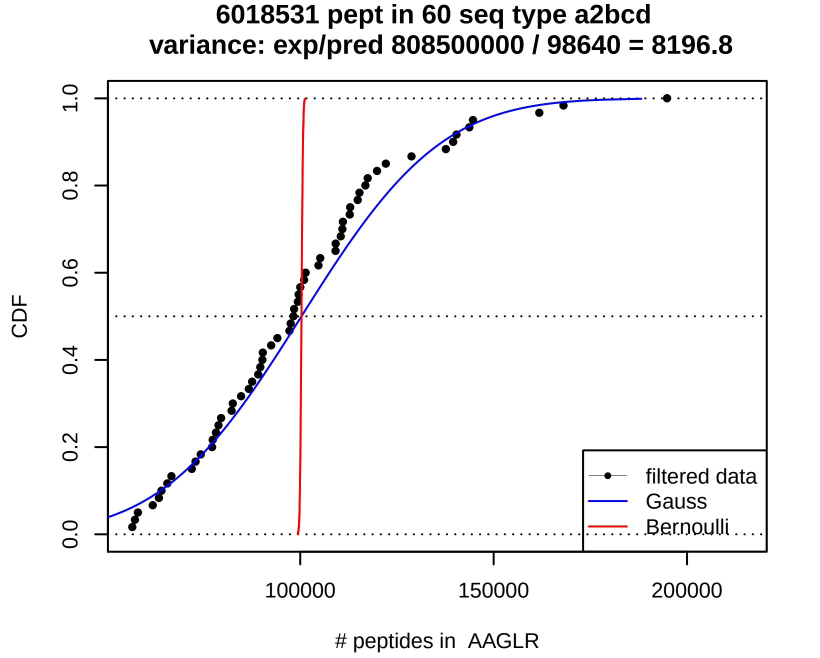
6018531 pept in 60 seq type a2bcd
variance: exp/pred 808500000 / 98640 = 8196.8
CDF
# peptides in AAGLR
filtered data
Gauss
Bernoulli

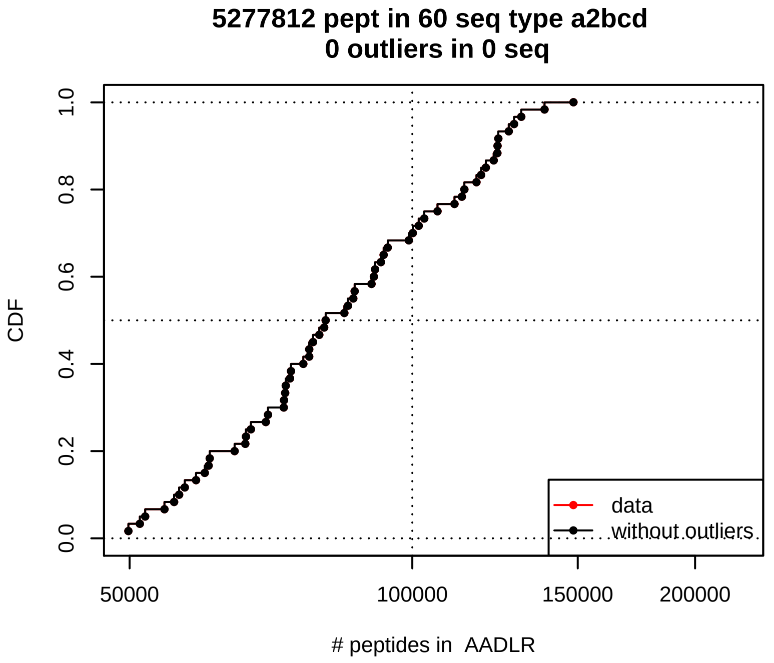
5277812 pept in 60 seq type a2bcd
0 outliers in 0 seq
CDF
# peptides in AADLR
data
without outliers

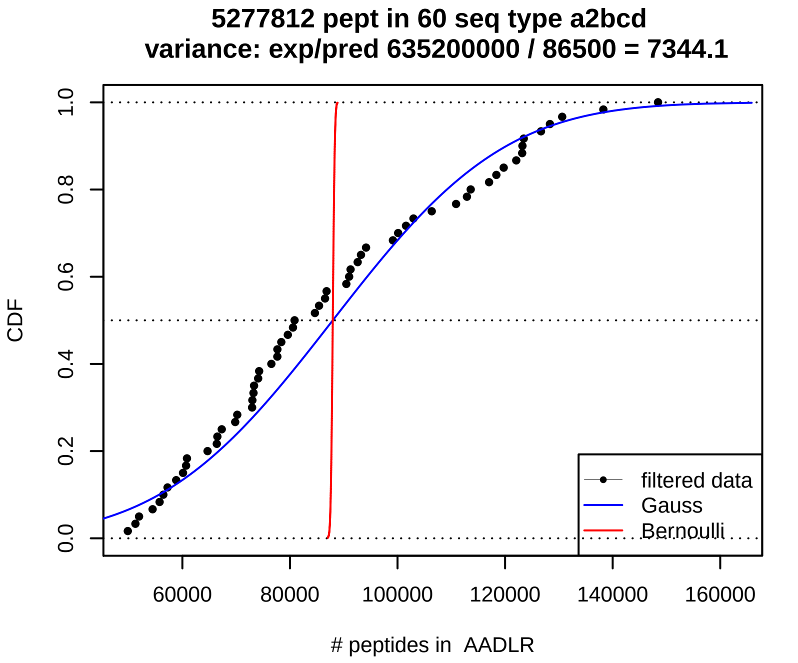
5277812 pept in 60 seq type a2bcd
variance: exp/pred 635200000 / 86500 = 7344.1
CDF
# peptides in AADLR
filtered data
Gauss
Bernoulli

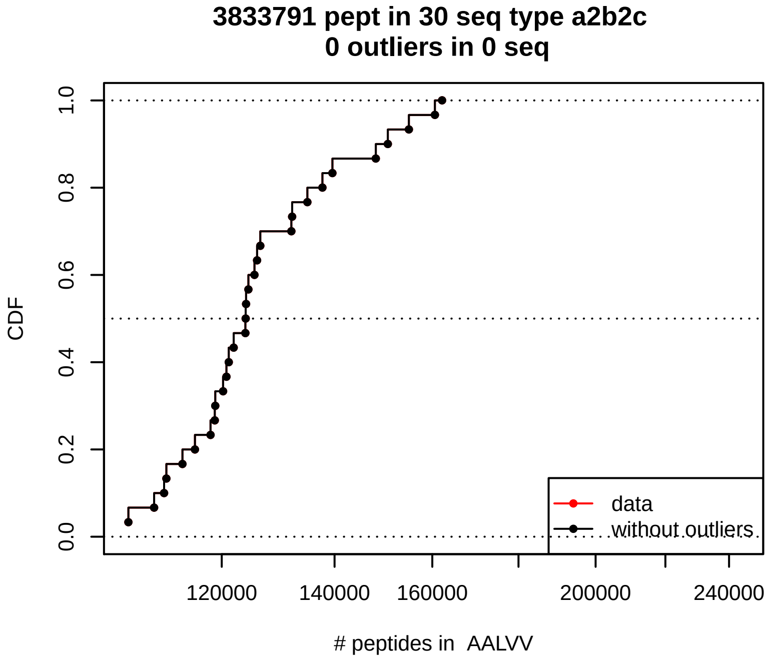
3833791 pept in 30 seq type a2b2c
0 outliers in 0 seq
CDF
# peptides in AALVV
data
without outliers

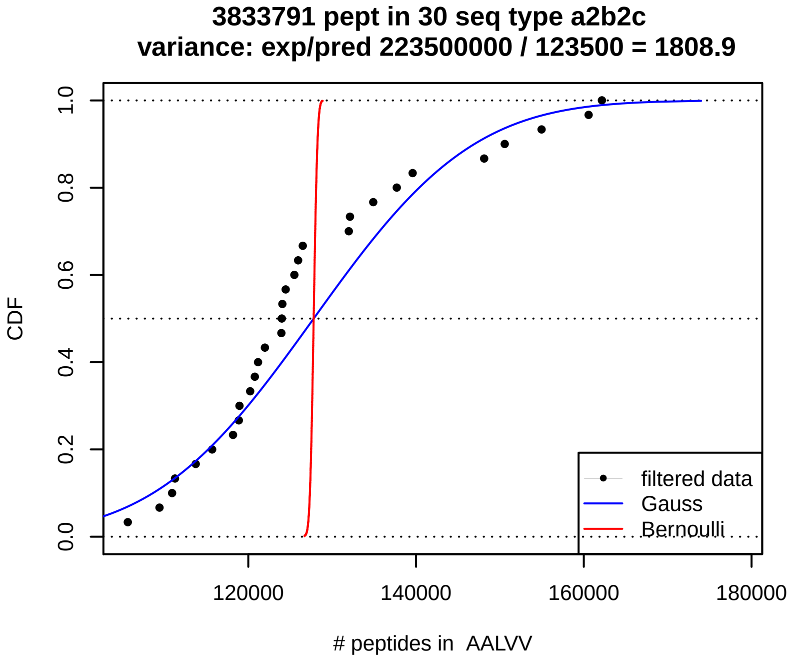
3833791 pept in 30 seq type a2b2c
variance: exp/pred 223500000 / 123500 = 1808.9
CDF
# peptides in AALVV
filtered data
Gauss
Bernoulli

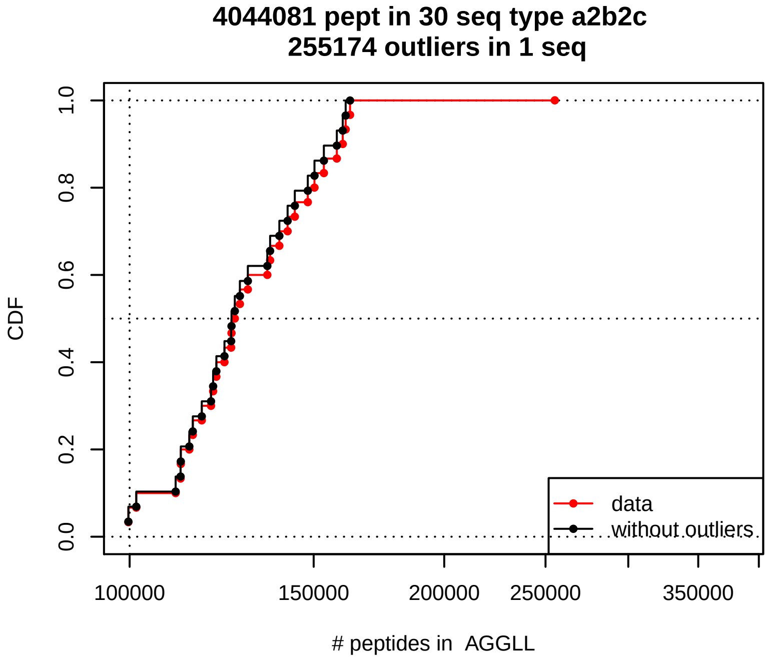
4044081 pept in 30 seq type a2b2c
255174 outliers in 1 seq
CDF
# peptides in AGGLL
data
without outliers

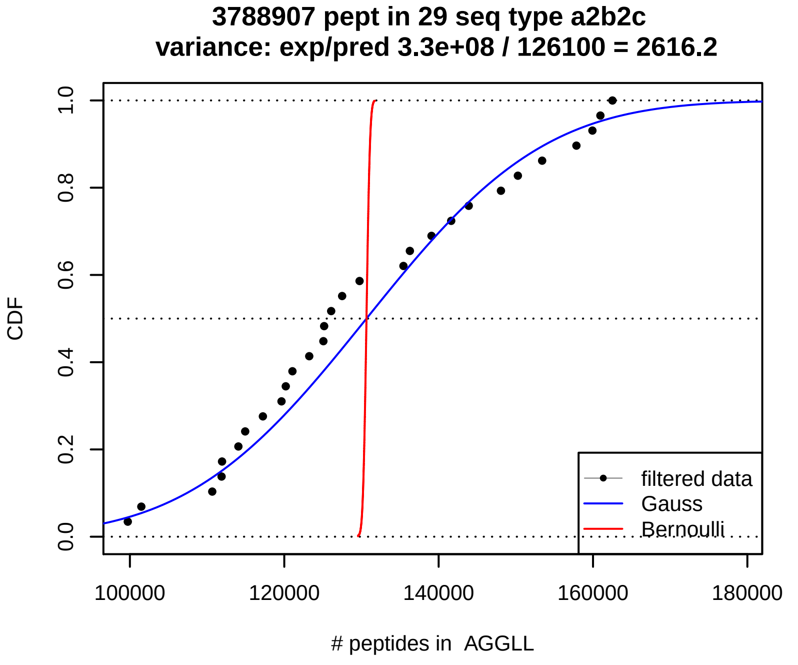
3788907 pept in 29 seq type a2b2c
variance: exp/pred 3.3e+08 / 126100 = 2616.2
CDF
# peptides in AGGLL
filtered data
Gauss
Bernoulli

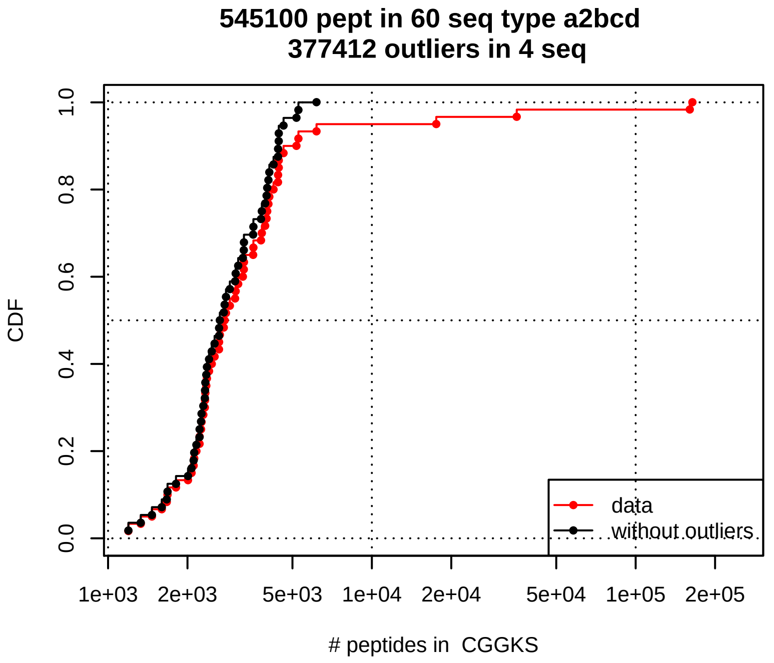
545100 pept in 60 seq type a2bcd
377412 outliers in 4 seq
CDF
# peptides in CGGKS
data
without outliers
1e+03 2e+03 5e+03 1e+04 2e+04 5e+04 1e+05 2e+05
0.0 0.2 0.4 0.6 0.8 1.0

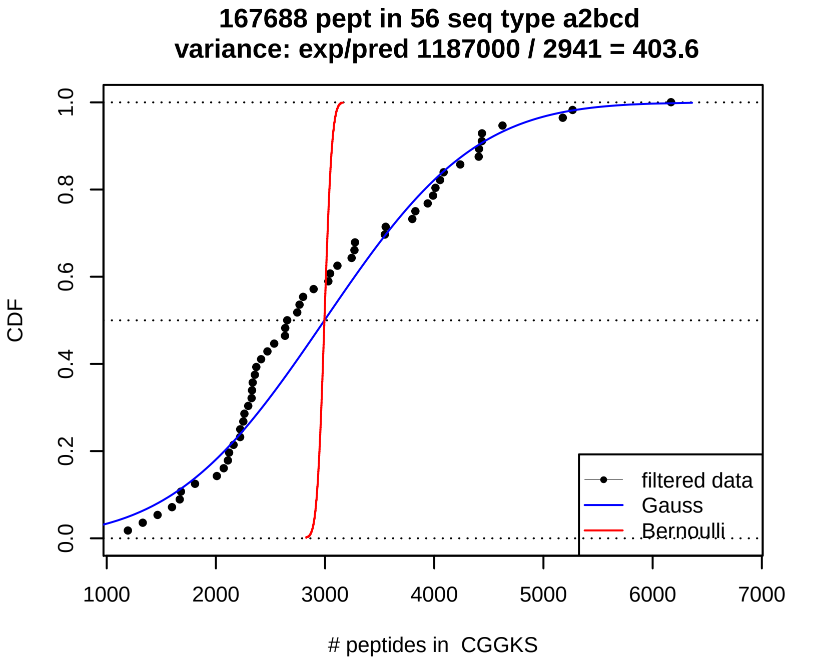
167688 pept in 56 seq type a2bcd
variance: exp/pred 1187000 / 2941 = 403.6
CDF
# peptides in CGGKS
filtered data
Gauss
Bernoulli
1000 2000 3000 4000 5000 6000 7000
0.0 0.2 0.4 0.6 0.8 1.0

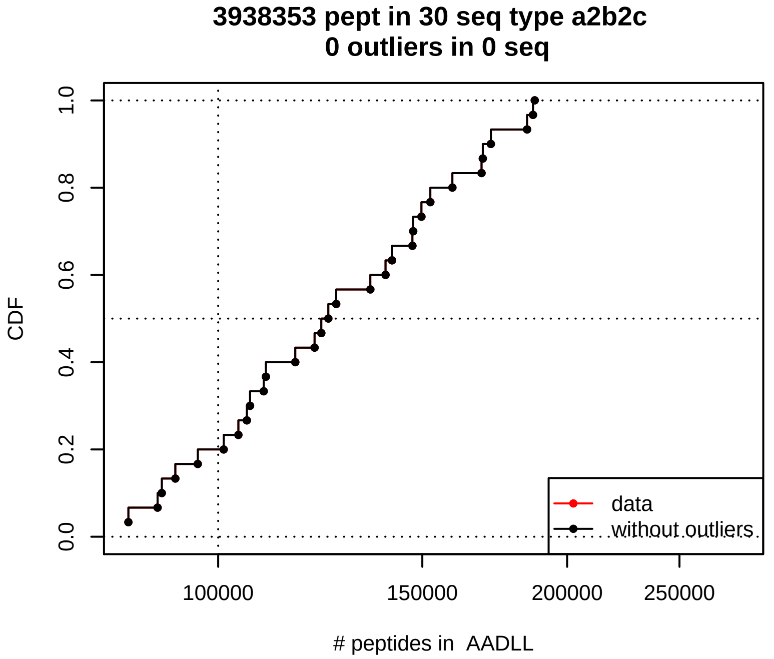
3938353 pept in 30 seq type a2b2c
0 outliers in 0 seq
CDF
# peptides in AADLL
data
without outliers
100000 150000 200000 250000
0.0 0.2 0.4 0.6 0.8 1.0

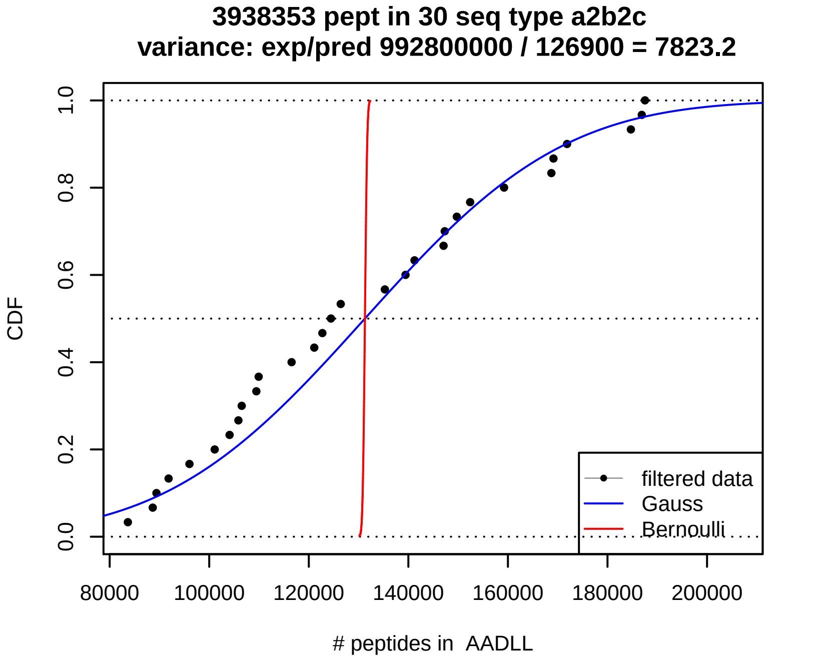
3938353 pept in 30 seq type a2b2c
variance: exp/pred 992800000 / 126900 = 7823.2
CDF
# peptides in AADLL
filtered data
Gauss
Bernoulli
80000 100000 120000 140000 160000 180000 200000
0.0 0.2 0.4 0.6 0.8 1.0

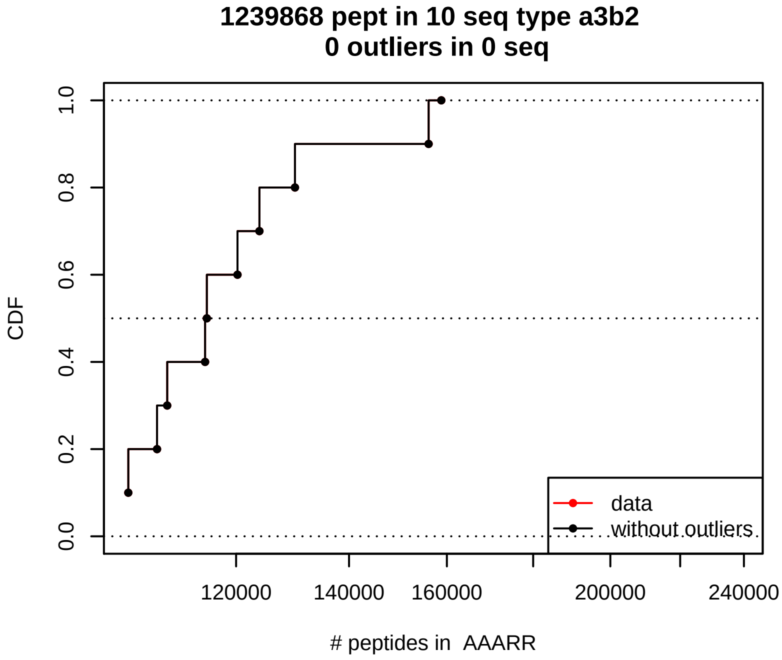
1239868 pept in 10 seq type a3b2
0 outliers in 0 seq
CDF
# peptides in AAARR
data
without outliers
120000 140000 160000 200000 240000
0.0 0.2 0.4 0.6 0.8 1.0

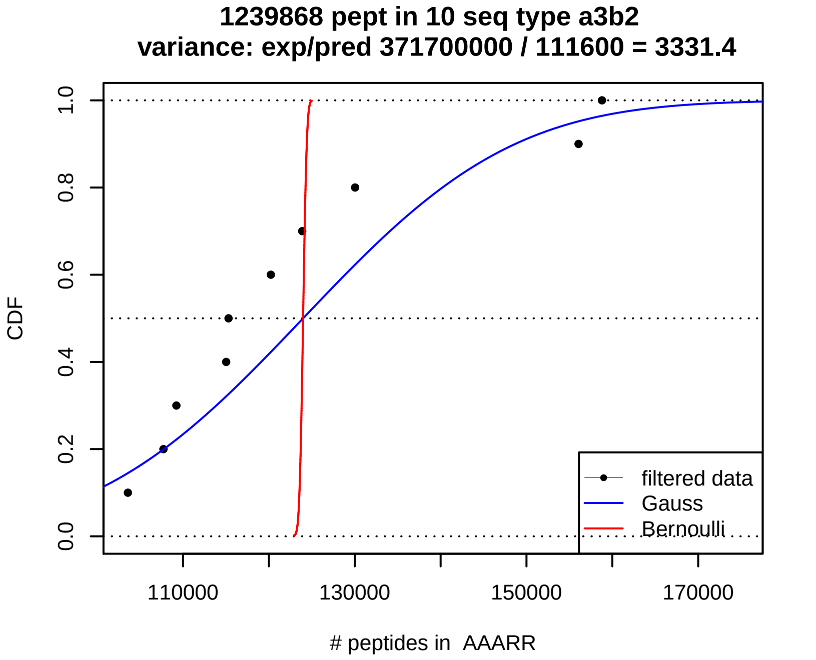
1239868 pept in 10 seq type a3b2
variance: exp/pred 371700000 / 111600 = 3331.4
CDF
# peptides in AAARR
filtered data
Gauss
Bernoulli
110000 130000 150000 170000
0.0 0.2 0.4 0.6 0.8 1.0

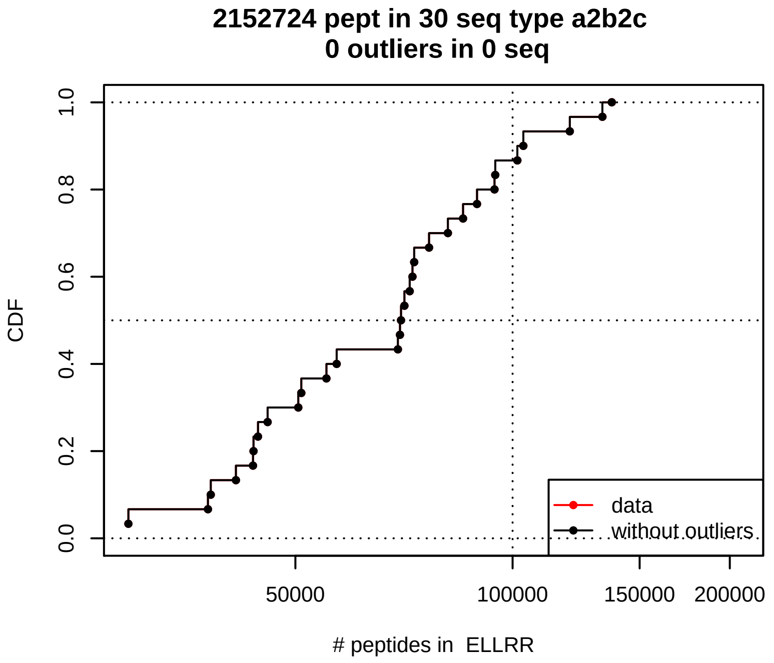
2152724 pept in 30 seq type a2b2c
0 outliers in 0 seq
CDF
0.0 0.2 0.4 0.6 0.8 1.0
50000 100000 150000 200000
# peptides in ELLRR
data
without outliers

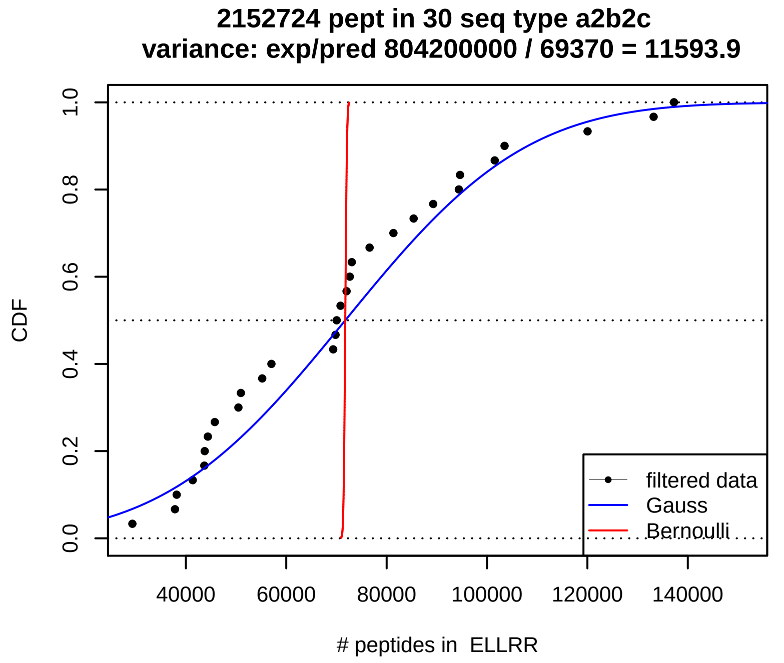
2152724 pept in 30 seq type a2b2c
variance: exp/pred 804200000 / 69370 = 11593.9
CDF
0.0 0.2 0.4 0.6 0.8 1.0
40000 60000 80000 100000 120000 140000
# peptides in ELLRR
filtered data
Gauss
Bernoulli

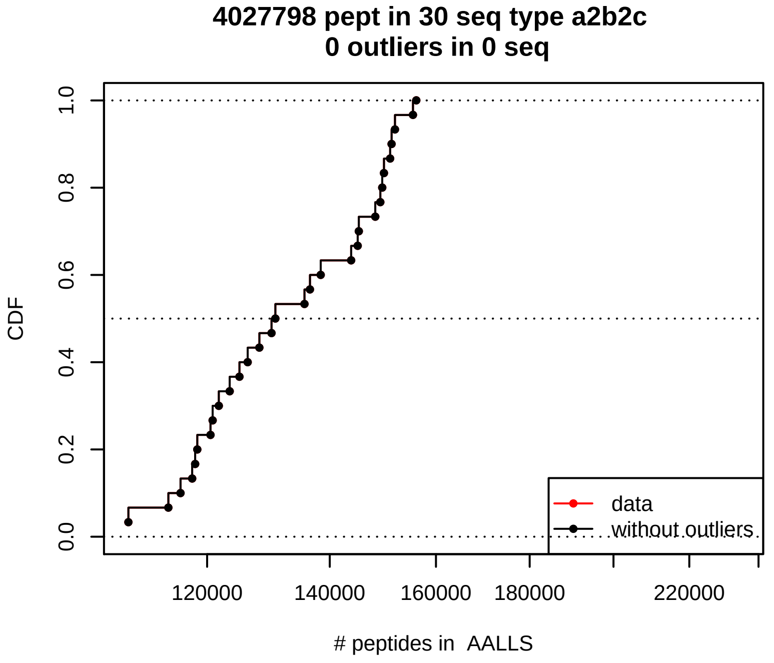
4027798 pept in 30 seq type a2b2c
0 outliers in 0 seq
CDF
0.0 0.2 0.4 0.6 0.8 1.0
120000 140000 160000 180000 220000
# peptides in AALLS
data
without outliers

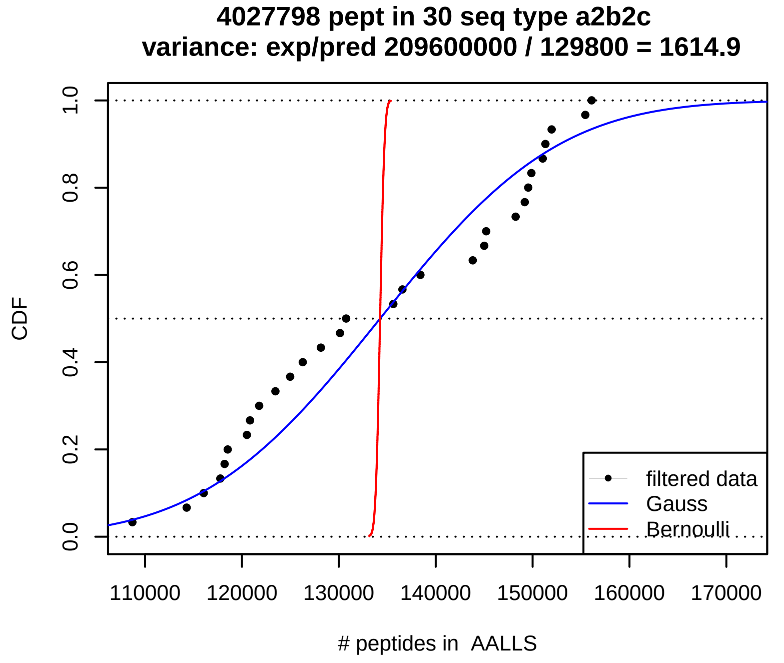
4027798 pept in 30 seq type a2b2c
variance: exp/pred 209600000 / 129800 = 1614.9
CDF
0.0 0.2 0.4 0.6 0.8 1.0
110000 120000 130000 140000 150000 160000 170000
# peptides in AALLS
filtered data
Gauss
Bernoulli

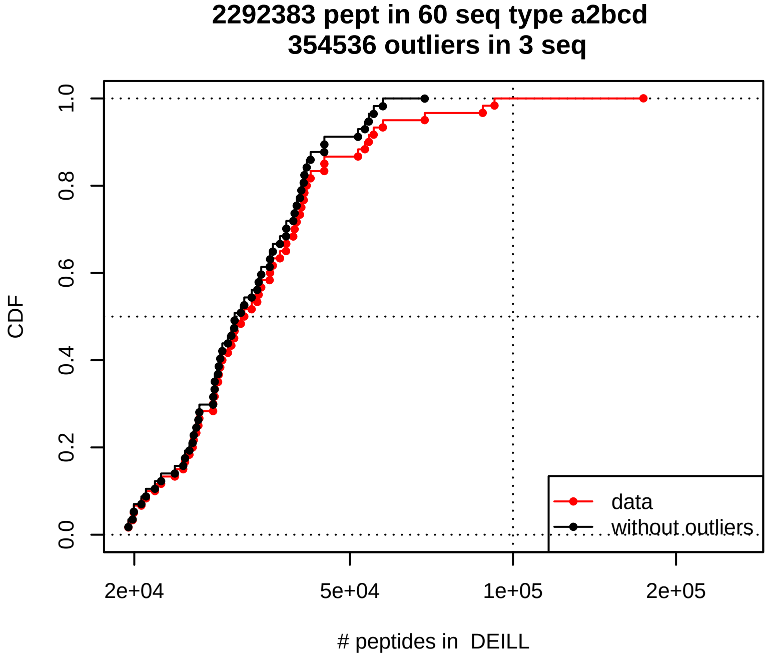
2292383 pept in 60 seq type a2bcd
354536 outliers in 3 seq
CDF
0.0 0.2 0.4 0.6 0.8 1.0
2e+04 5e+04 1e+05 2e+05
# peptides in DEILL
data
without outliers

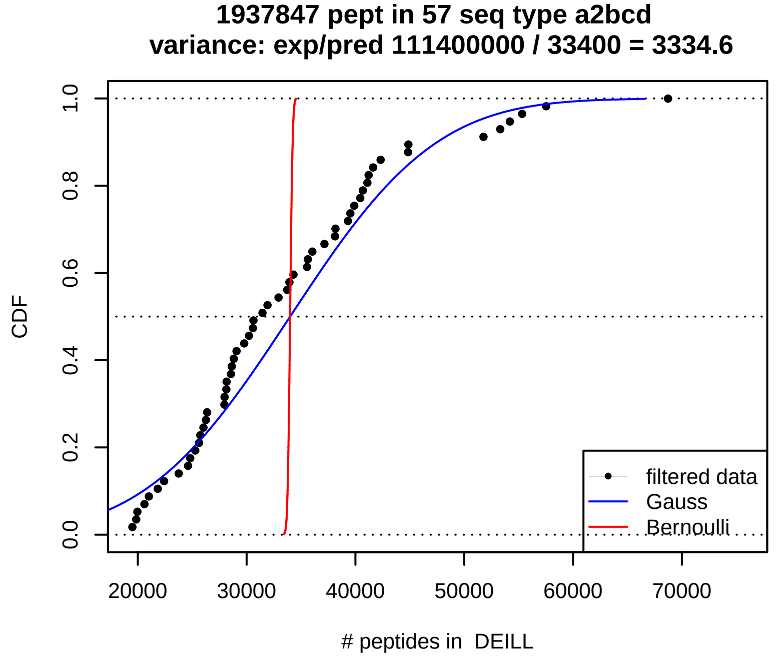
1937847 pept in 57 seq type a2bcd
variance: exp/pred 111400000 / 33400 = 3334.6
CDF
0.0 0.2 0.4 0.6 0.8 1.0
20000 30000 40000 50000 60000 70000
# peptides in DEILL
filtered data
Gauss
Bernoulli

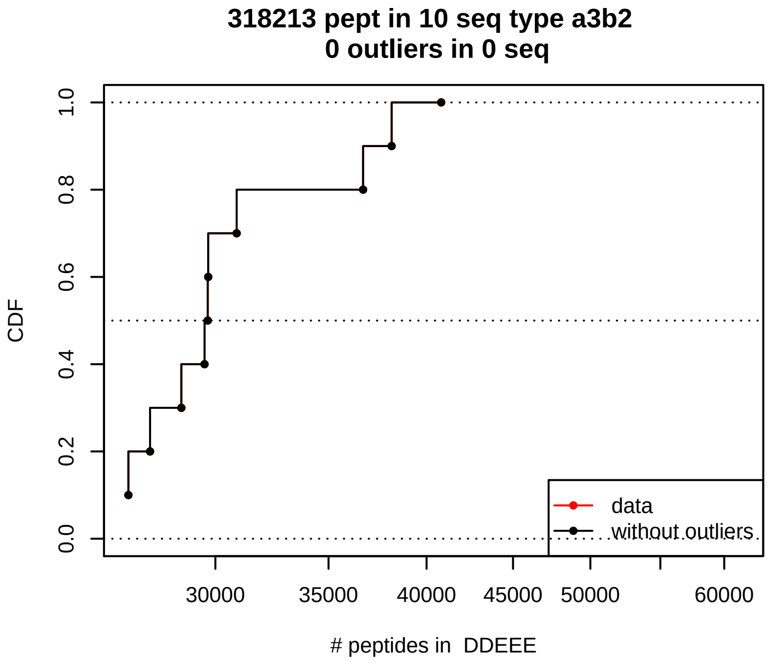
318213 pept in 10 seq type a3b2
0 outliers in 0 seq
CDF
# peptides in DDEEE
data
without outliers
0.0
0.2
0.4
0.6
0.8
1.0
30000
35000
40000
45000
50000
60000

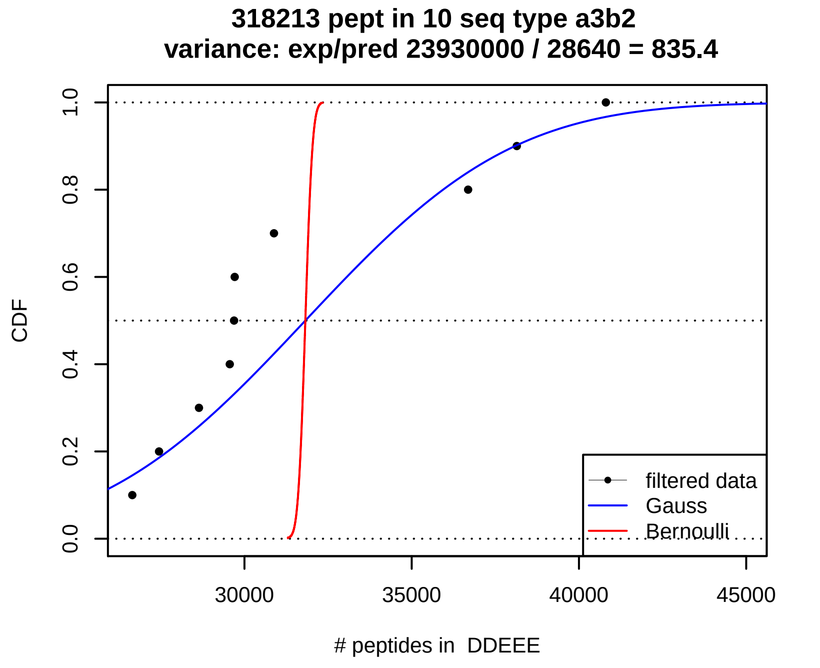
318213 pept in 10 seq type a3b2
variance: exp/pred 23930000 / 28640 = 835.4
CDF
# peptides in DDEEE
filtered data
Gauss
Bernoulli
0.0
0.2
0.4
0.6
0.8
1.0
30000
35000
40000
45000

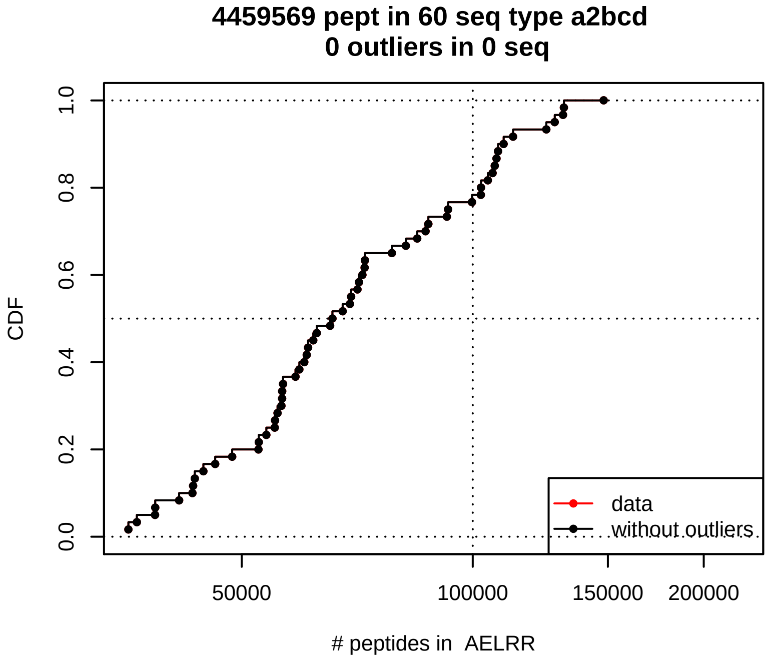
4459569 pept in 60 seq type a2bcd
0 outliers in 0 seq
CDF
# peptides in AELRR
data
without outliers
0.0
0.2
0.4
0.6
0.8
1.0
50000
100000
150000
200000

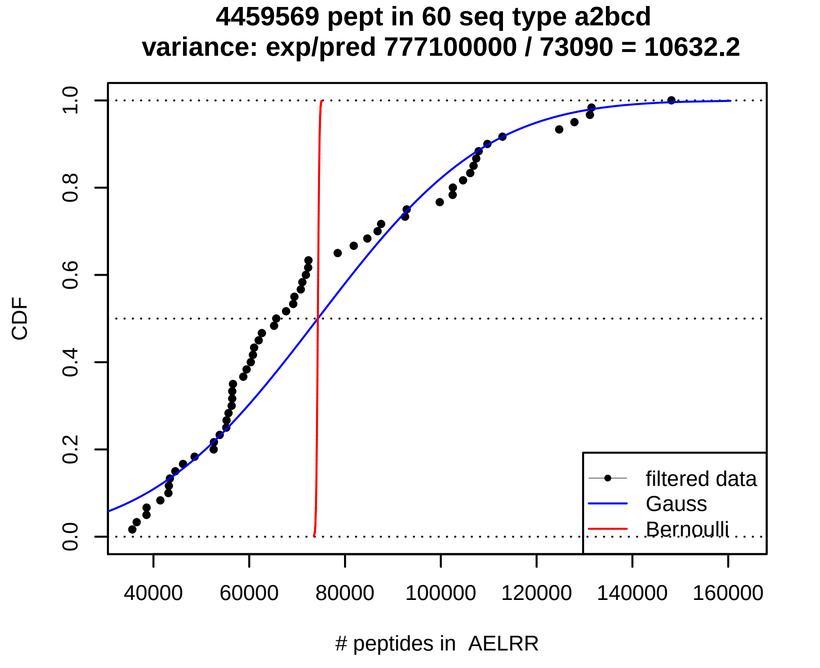
4459569 pept in 60 seq type a2bcd
variance: exp/pred 777100000 / 73090 = 10632.2
CDF
# peptides in AELRR
filtered data
Gauss
Bernoulli
0.0
0.2
0.4
0.6
0.8
1.0
40000
60000
80000
100000
120000
140000
160000

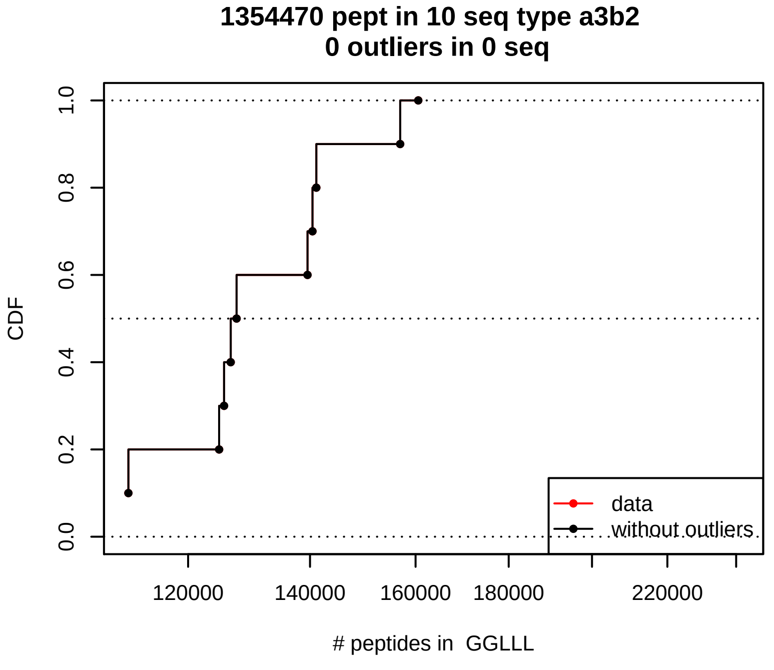
1354470 pept in 10 seq type a3b2
0 outliers in 0 seq
CDF
# peptides in GGLLL
data
without outliers
0.0
0.2
0.4
0.6
0.8
1.0
120000
140000
160000
180000
220000

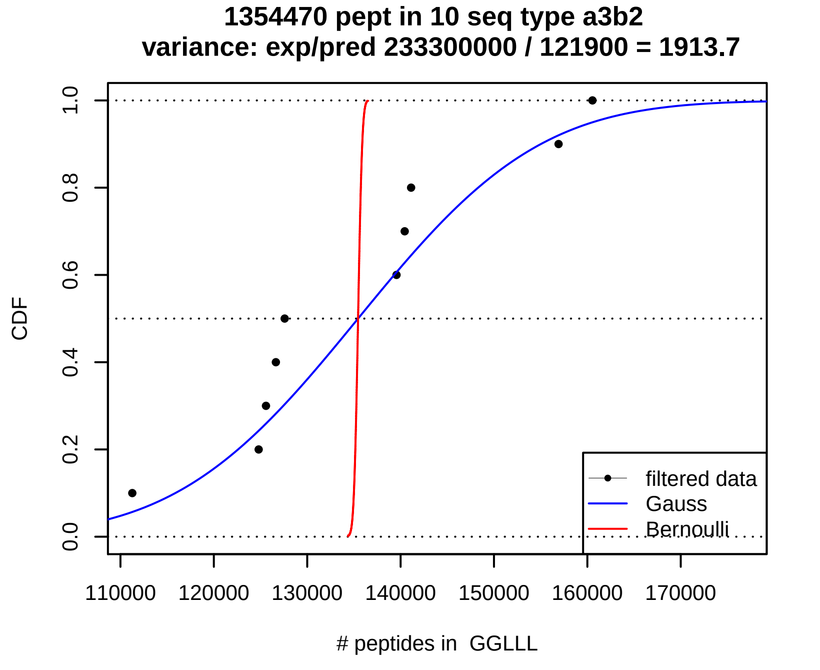
1354470 pept in 10 seq type a3b2
variance: exp/pred 233300000 / 121900 = 1913.7
CDF
# peptides in GGLLL
filtered data
Gauss
Bernoulli
0.0
0.2
0.4
0.6
0.8
1.0
110000
120000
130000
140000
150000
160000
170000

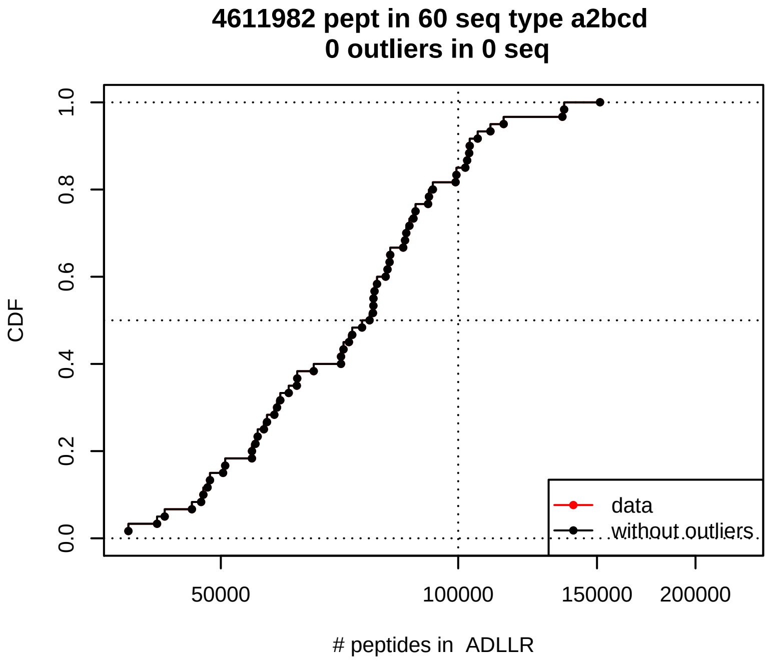
4611982 pept in 60 seq type a2bcd
0 outliers in 0 seq
CDF
0.0
0.2
0.4
0.6
0.8
1.0
50000
100000
150000
200000
# peptides in ADLLR
data
without outliers

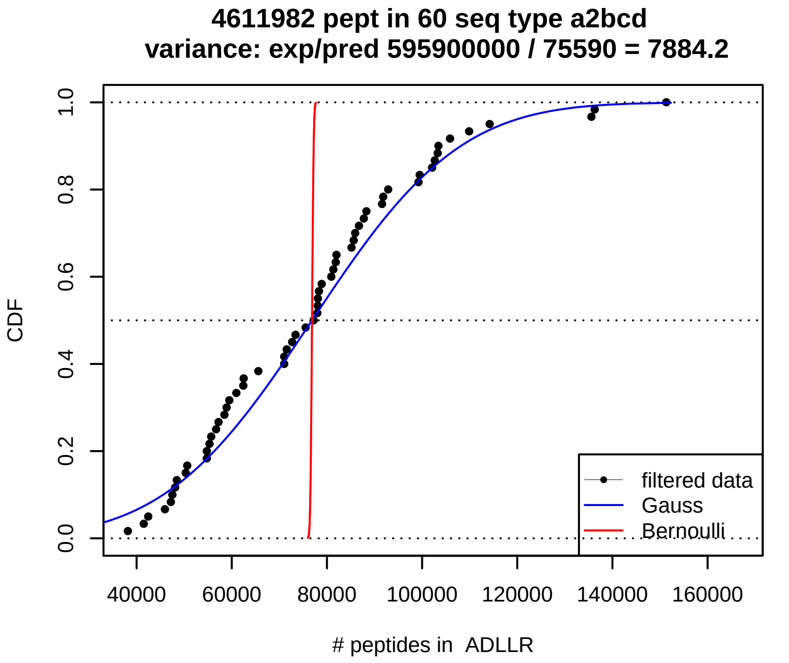
4611982 pept in 60 seq type a2bcd
variance: exp/pred 595900000 / 75590 = 7884.2
CDF
0.0
0.2
0.4
0.6
0.8
1.0
40000
60000
80000
100000
120000
140000
160000
# peptides in ADLLR
filtered data
Gauss
Bernoulli

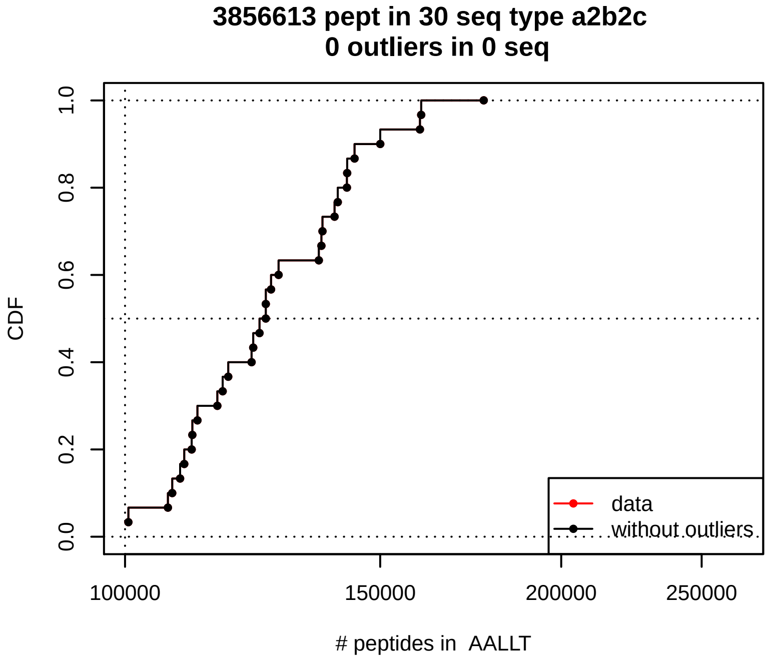
3856613 pept in 30 seq type a2b2c
0 outliers in 0 seq
CDF
0.0
0.2
0.4
0.6
0.8
1.0
100000
150000
200000
250000
# peptides in AALLT
data
without outliers

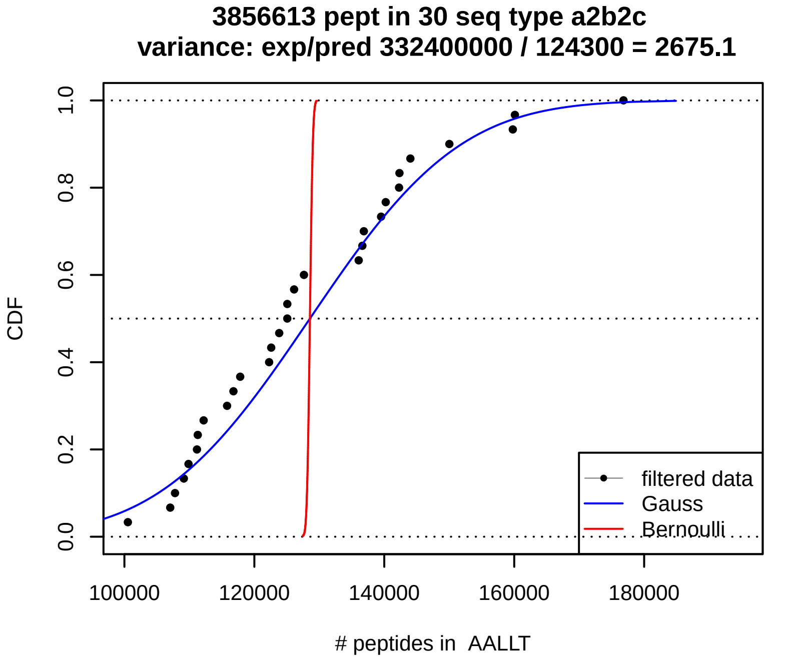
3856613 pept in 30 seq type a2b2c
variance: exp/pred 332400000 / 124300 = 2675.1
CDF
0.0
0.2
0.4
0.6
0.8
1.0
100000
120000
140000
160000
180000
# peptides in AALLT
filtered data
Gauss
Bernoulli

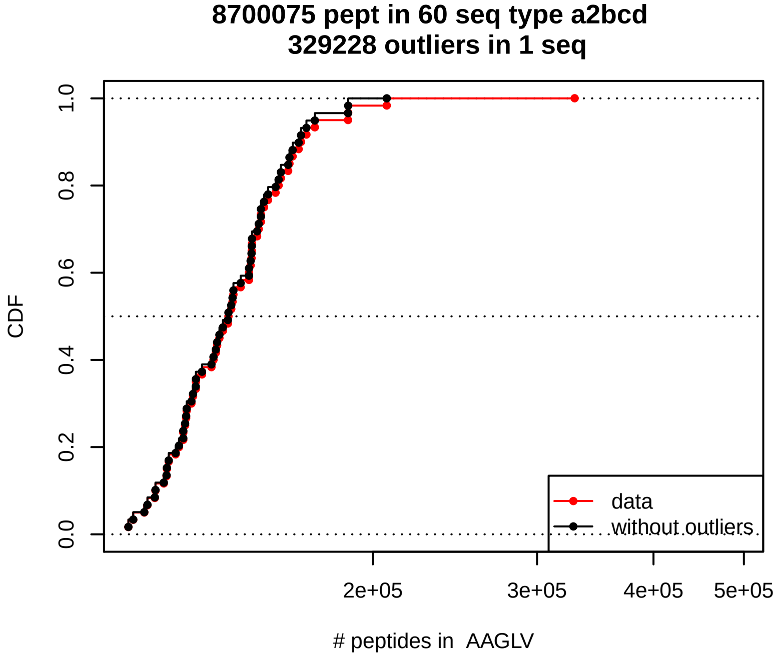
8700075 pept in 60 seq type a2bcd
329228 outliers in 1 seq
CDF
0.0
0.2
0.4
0.6
0.8
1.0
2e+05
3e+05
4e+05
5e+05
# peptides in AAGLV
data
without outliers

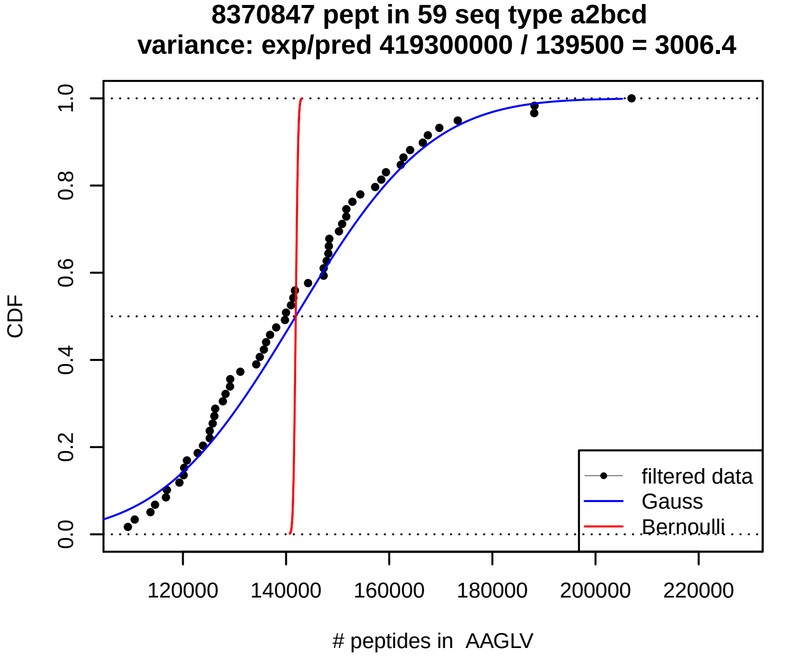
8370847 pept in 59 seq type a2bcd
variance: exp/pred 419300000 / 139500 = 3006.4
CDF
0.0
0.2
0.4
0.6
0.8
1.0
120000
140000
160000
180000
200000
220000
# peptides in AAGLV
filtered data
Gauss
Bernoulli

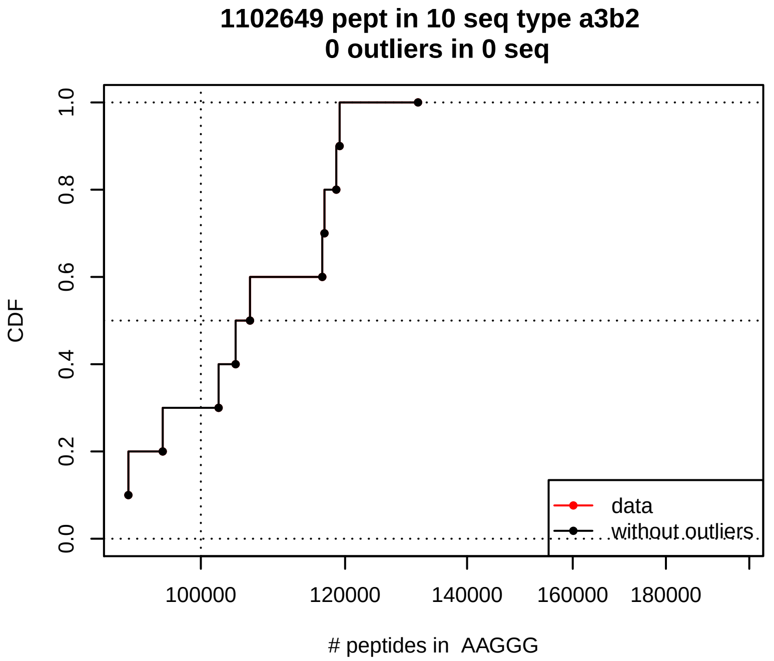
1102649 pept in 10 seq type a3b2
0 outliers in 0 seq
CDF
0.0
0.2
0.4
0.6
0.8
1.0
100000
120000
140000
160000
180000
# peptides in AAGGG
data
without outliers

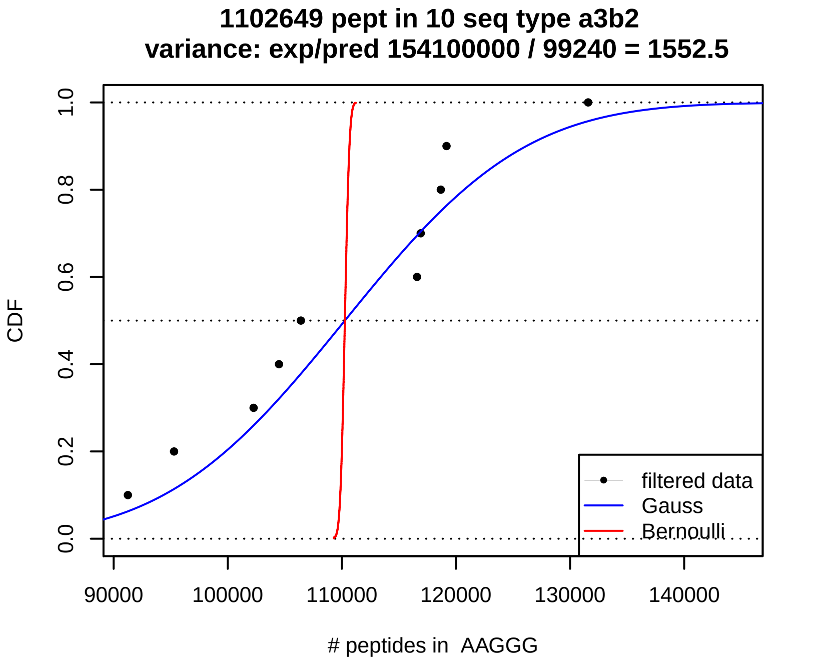
1102649 pept in 10 seq type a3b2
variance: exp/pred 154100000 / 99240 = 1552.5
CDF
0.0
0.2
0.4
0.6
0.8
1.0
90000
100000
110000
120000
130000
140000
# peptides in AAGGG
filtered data
Gauss
Bernoulli

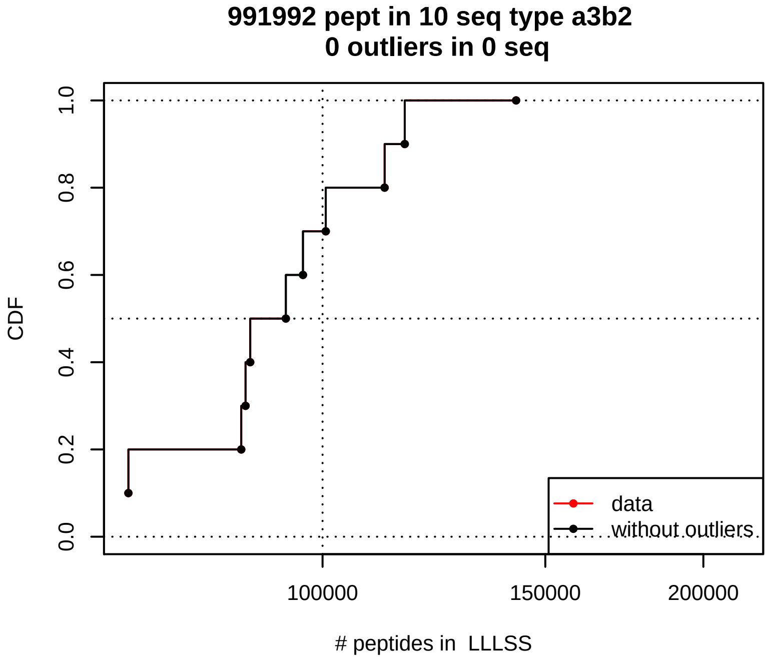
991992 pept in 10 seq type a3b2
0 outliers in 0 seq
CDF
0.0
0.2
0.4
0.6
0.8
1.0
100000
150000
200000
# peptides in LLLSS
data
without outliers

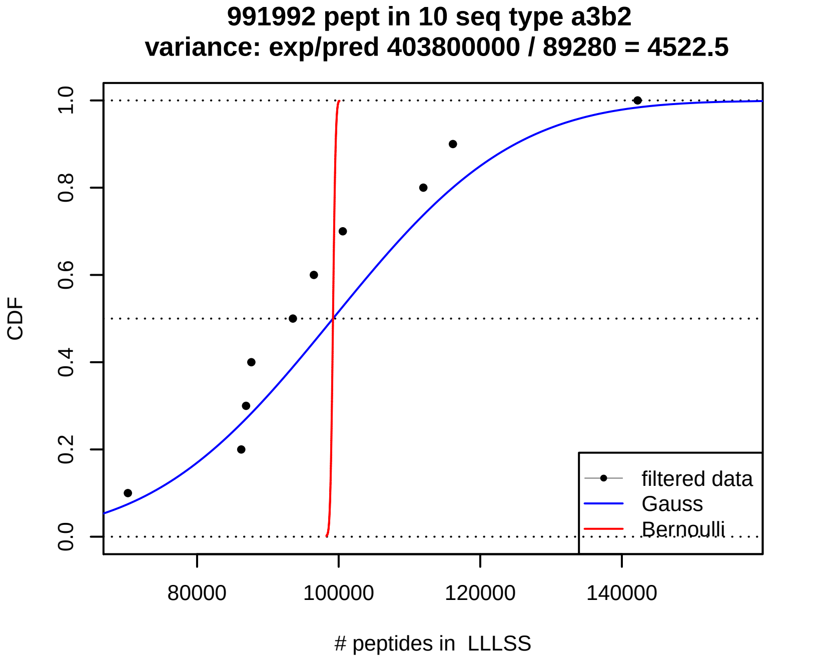
991992 pept in 10 seq type a3b2
variance: exp/pred 403800000 / 89280 = 4522.5
CDF
0.0
0.2
0.4
0.6
0.8
1.0
80000
100000
120000
140000
# peptides in LLLSS
filtered data
Gauss
Bernoulli

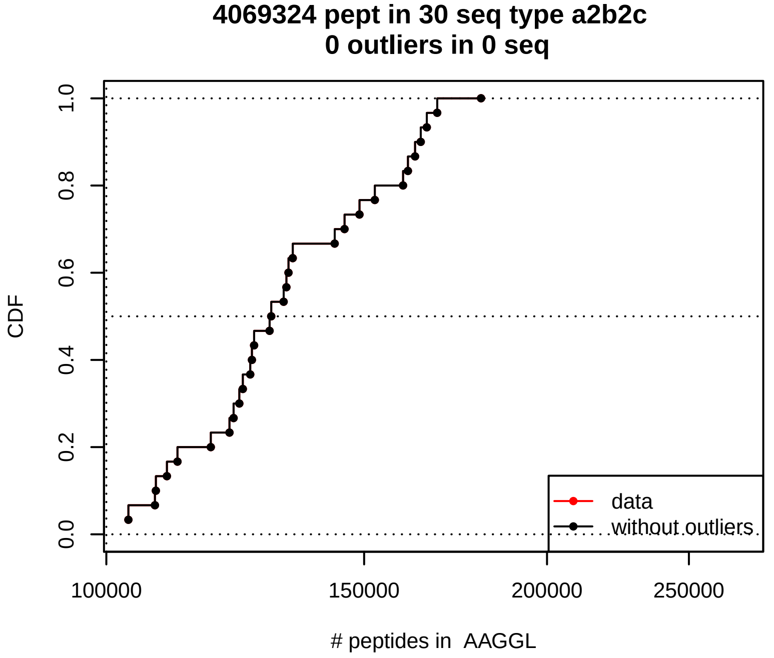
4069324 pept in 30 seq type a2b2c
0 outliers in 0 seq
CDF
0.0
0.2
0.4
0.6
0.8
1.0
100000
150000
200000
250000
# peptides in AAGGL
data
without outliers

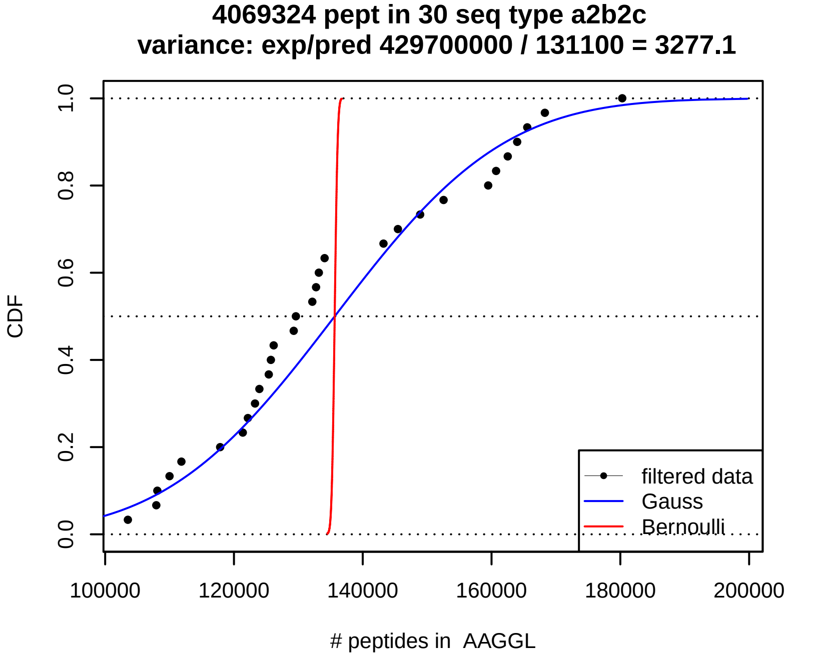
4069324 pept in 30 seq type a2b2c
variance: exp/pred 429700000 / 131100 = 3277.1
CDF
0.0
0.2
0.4
0.6
0.8
1.0
100000
120000
140000
160000
180000
200000
# peptides in AAGGL
filtered data
Gauss
Bernoulli

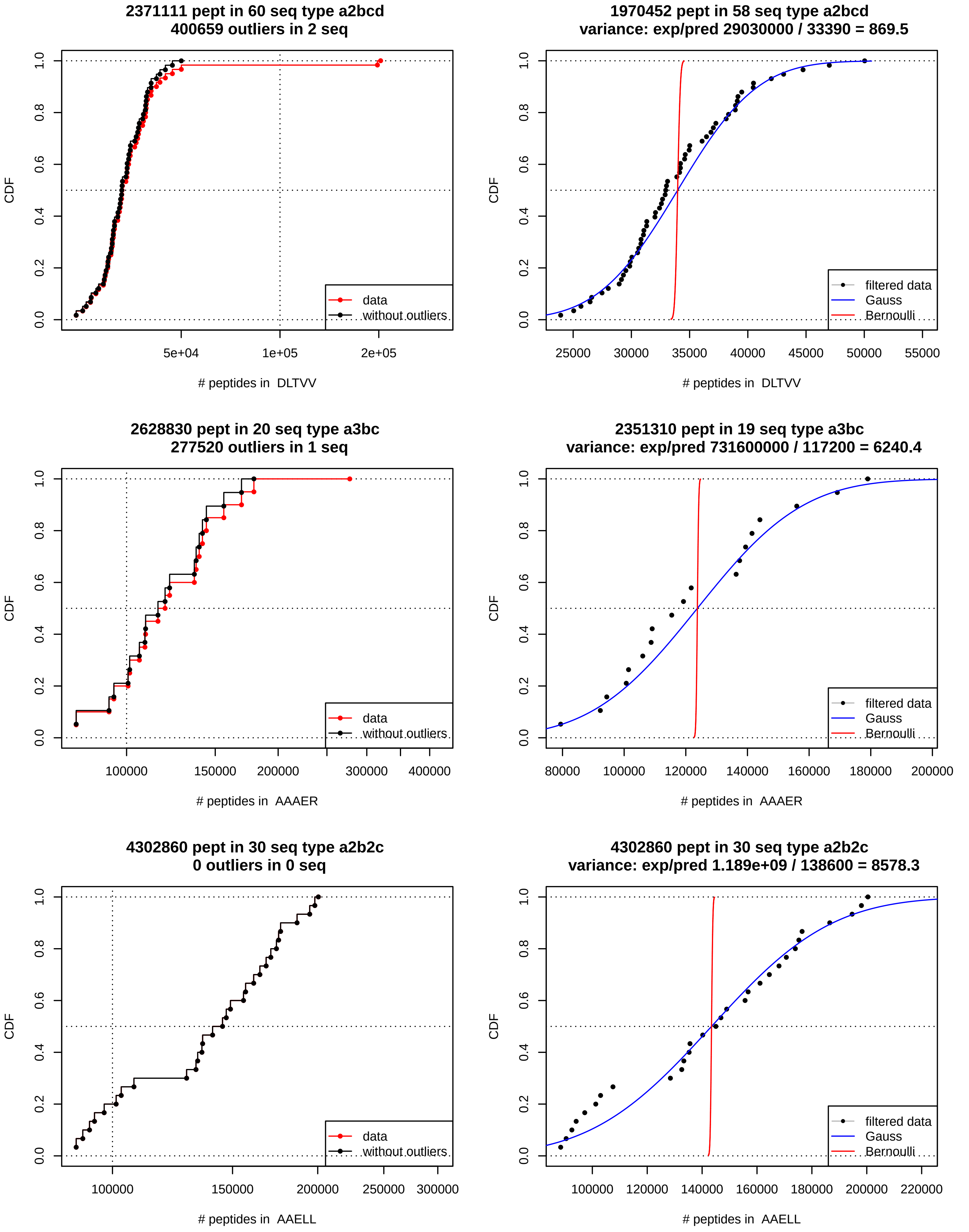

2371111 pept in 60 seq type a2bcd
400659 outliers in 2 seq
CDF
# peptides in DLTVV
5e+04
1e+05
2e+05
data
without outliers
1970452 pept in 58 seq type a2bcd
variance: exp/pred 29030000 / 33390 = 869.5
CDF
# peptides in DLTVV
25000
30000
35000
40000
45000
50000
55000
filtered data
Gauss
Bernoulli
2628830 pept in 20 seq type a3bc
277520 outliers in 1 seq
CDF
# peptides in AAAER
100000
150000
200000
300000
400000
data
without outliers
2351310 pept in 19 seq type a3bc
variance: exp/pred 731600000 / 117200 = 6240.4
CDF
# peptides in AAAER
80000
100000
120000
140000
160000
180000
200000
filtered data
Gauss
Bernoulli
4302860 pept in 30 seq type a2b2c
0 outliers in 0 seq
CDF
# peptides in AAELL
100000
150000
200000
250000
300000
data
without outliers
4302860 pept in 30 seq type a2b2c
variance: exp/pred 1.189e+09 / 138600 = 8578.3
CDF
# peptides in AAELL
100000
120000
140000
160000
180000
200000
220000
filtered data
Gauss
Bernoulli
0.0
0.2
0.4
0.6
0.8
1.0

**2956105 pept in 20 seq type a3bc**
**0 outliers in 0 seq**

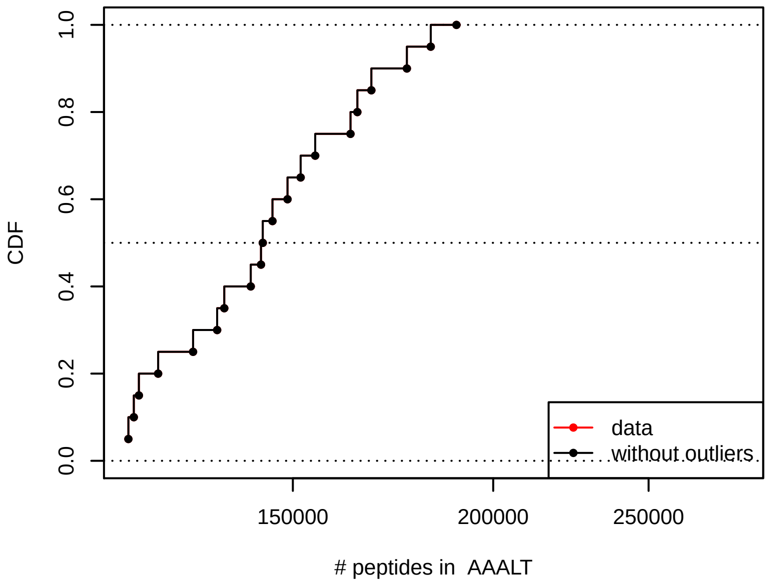


**2956105 pept in 20 seq type a3bc**
**variance: exp/pred 449100000 / 140400 = 3198.6**

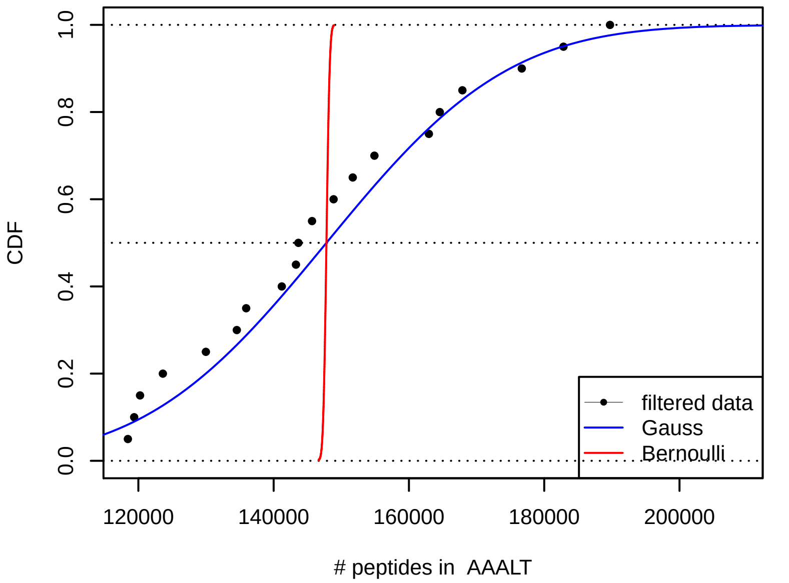


**5217301 pept in 30 seq type a2b2c**
**0 outliers in 0 seq**

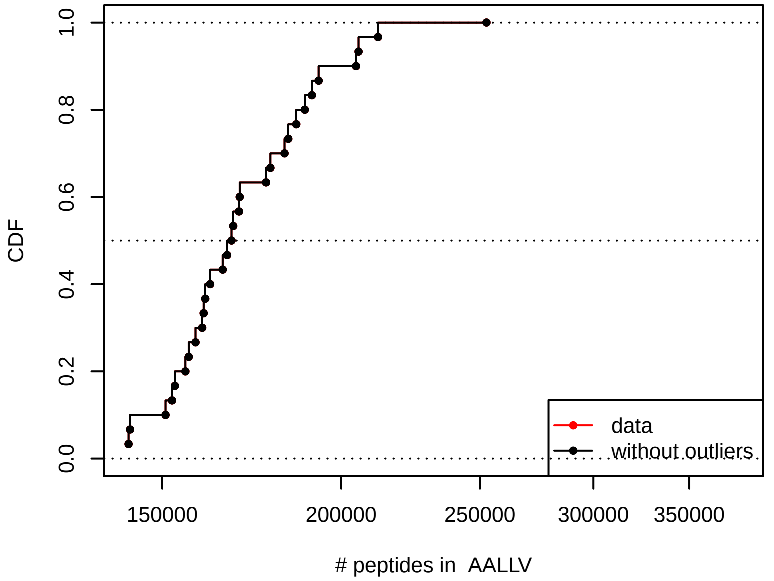


**5217301 pept in 30 seq type a2b2c**
**variance: exp/pred 554400000 / 168100 = 3297.8**

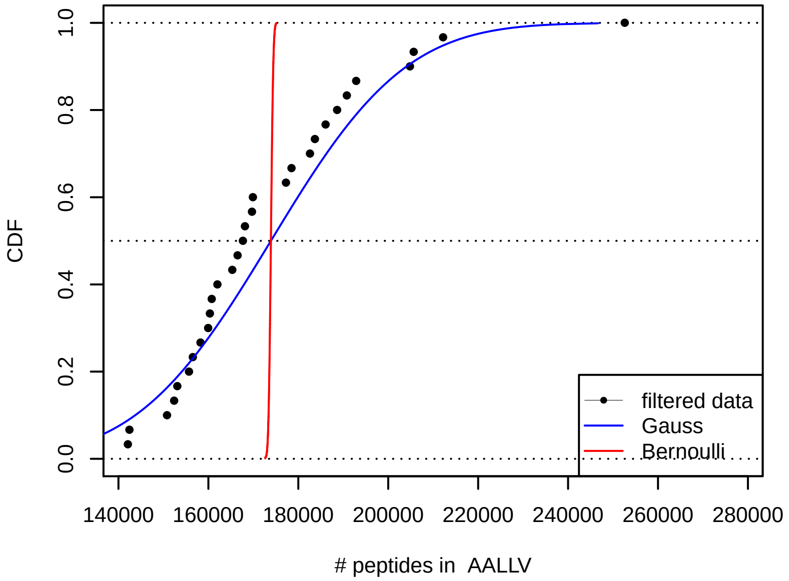


**1653312 pept in 10 seq type a3b2**
**0 outliers in 0 seq**

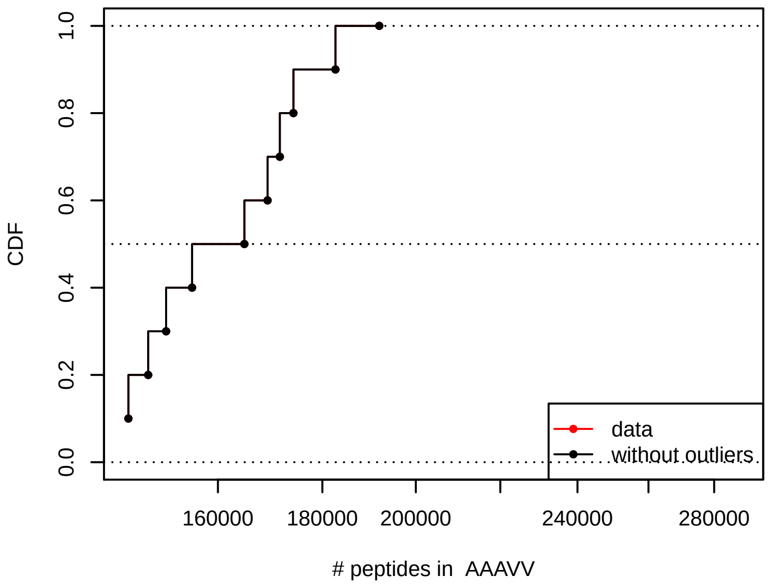


**1653312 pept in 10 seq type a3b2**
**variance: exp/pred 242300000 / 148800 = 1628.1**

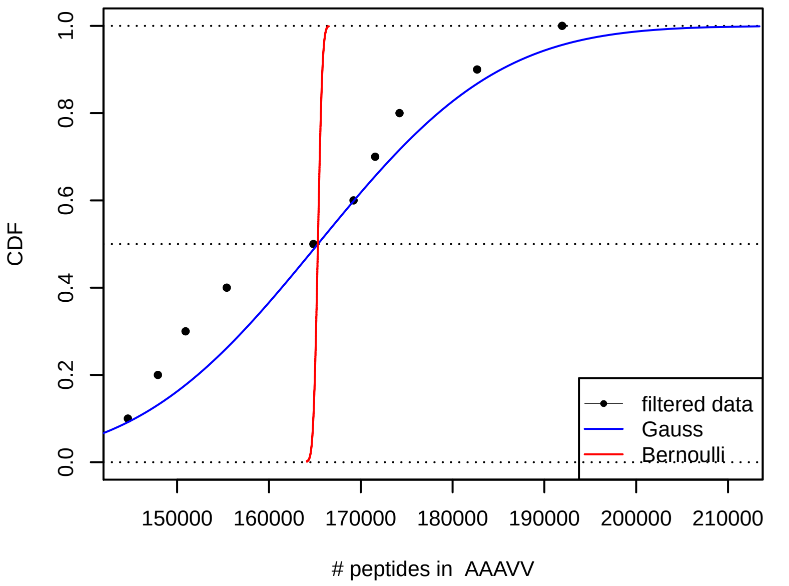

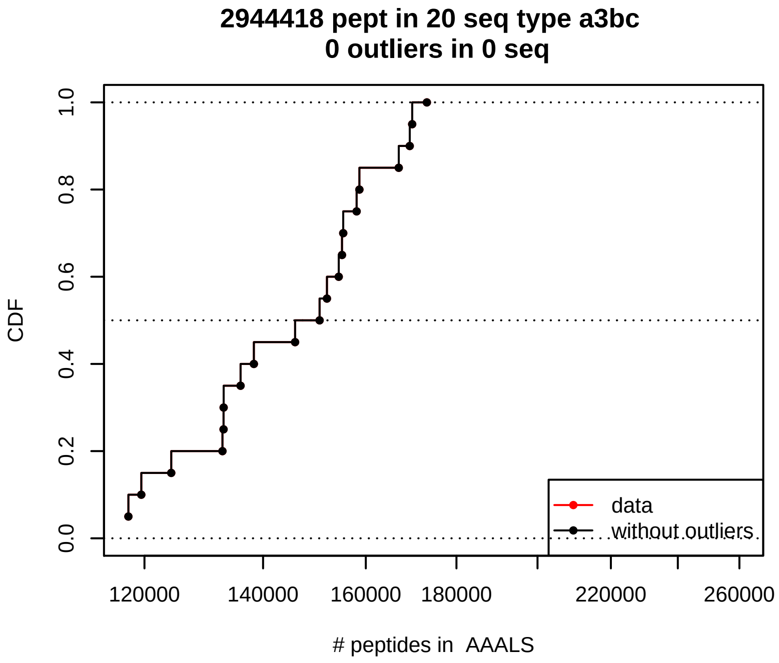
2944418 pept in 20 seq type a3bc
0 outliers in 0 seq
CDF
0.0
0.2
0.4
0.6
0.8
1.0
120000
140000
160000
180000
220000
260000
# peptides in AAALS
data
without outliers

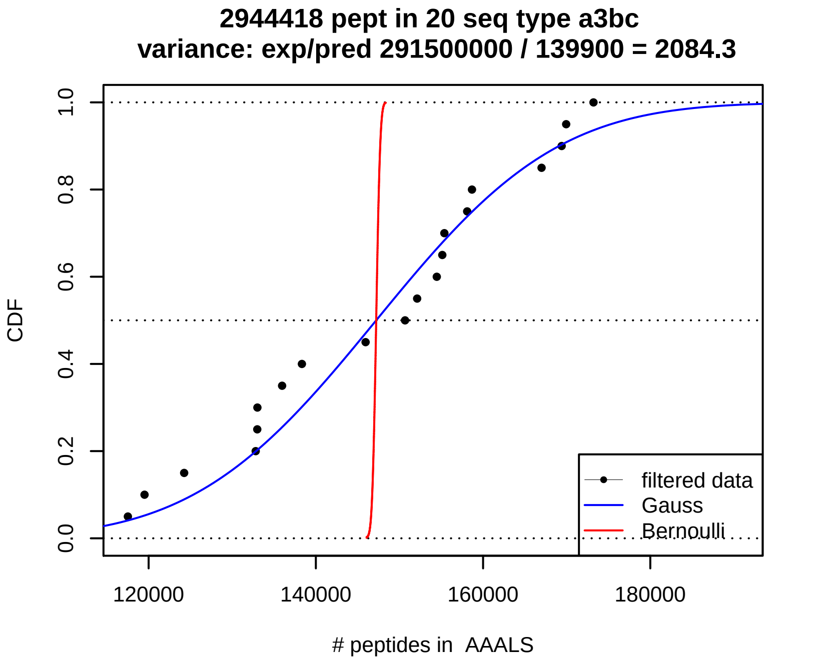
2944418 pept in 20 seq type a3bc
variance: exp/pred 291500000 / 139900 = 2084.3
CDF
0.0
0.2
0.4
0.6
0.8
1.0
120000
140000
160000
180000
# peptides in AAALS
filtered data
Gauss
Bernoulli

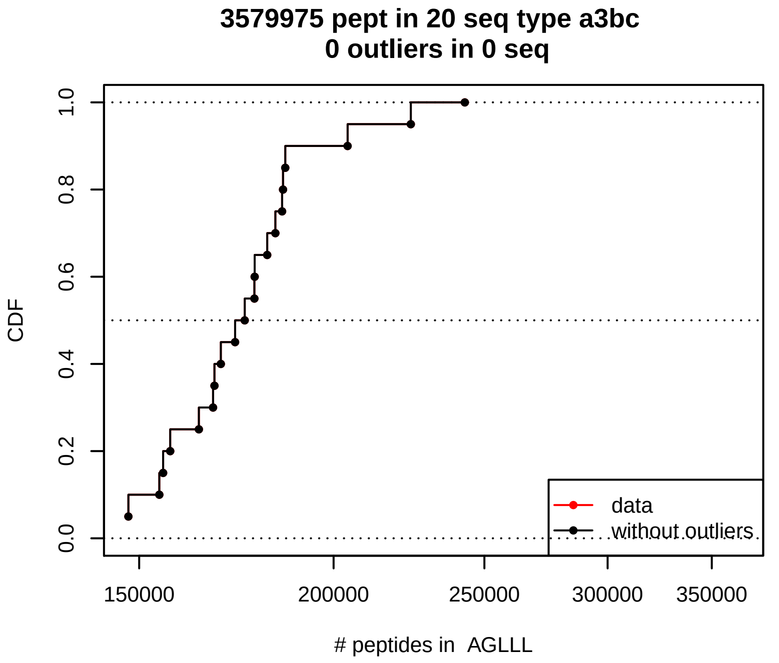
3579975 pept in 20 seq type a3bc
0 outliers in 0 seq
CDF
0.0
0.2
0.4
0.6
0.8
1.0
150000
200000
250000
300000
350000
# peptides in AGLLL
data
without outliers

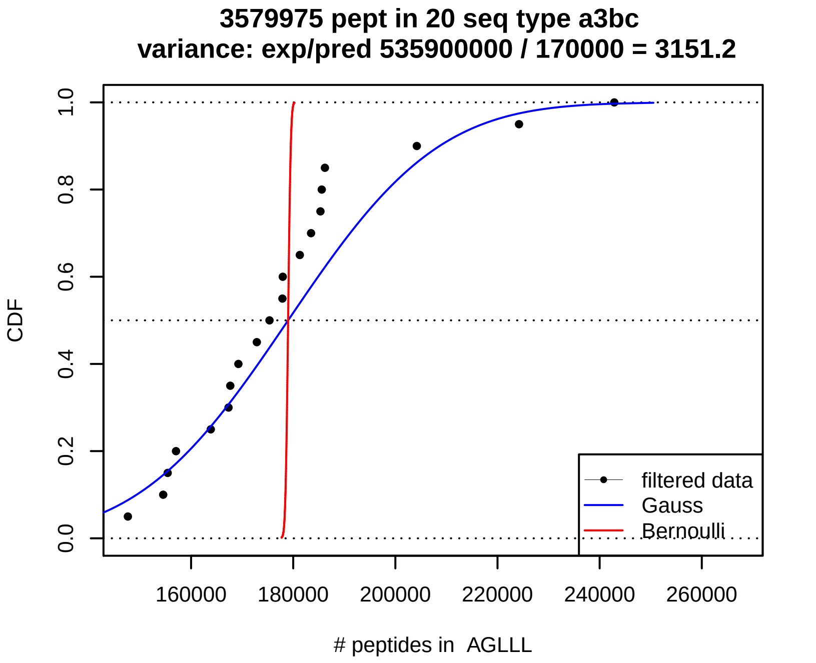
3579975 pept in 20 seq type a3bc
variance: exp/pred 535900000 / 170000 = 3151.2
CDF
0.0
0.2
0.4
0.6
0.8
1.0
160000
180000
200000
220000
240000
260000
# peptides in AGLLL
filtered data
Gauss
Bernoulli

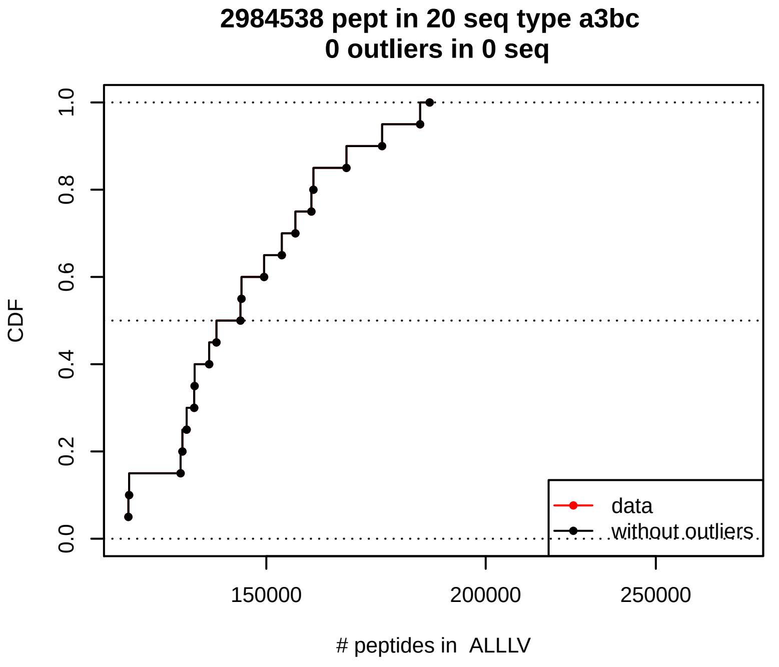
2984538 pept in 20 seq type a3bc
0 outliers in 0 seq
CDF
0.0
0.2
0.4
0.6
0.8
1.0
150000
200000
250000
# peptides in ALLLV
data
without outliers

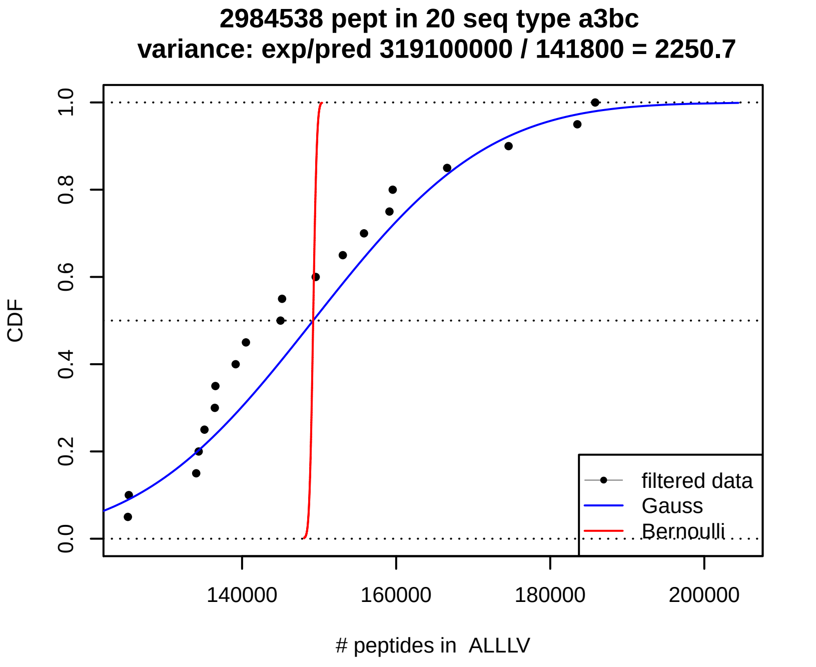
2984538 pept in 20 seq type a3bc
variance: exp/pred 319100000 / 141800 = 2250.7
CDF
0.0
0.2
0.4
0.6
0.8
1.0
140000
160000
180000
200000
# peptides in ALLLV
filtered data
Gauss
Bernoulli

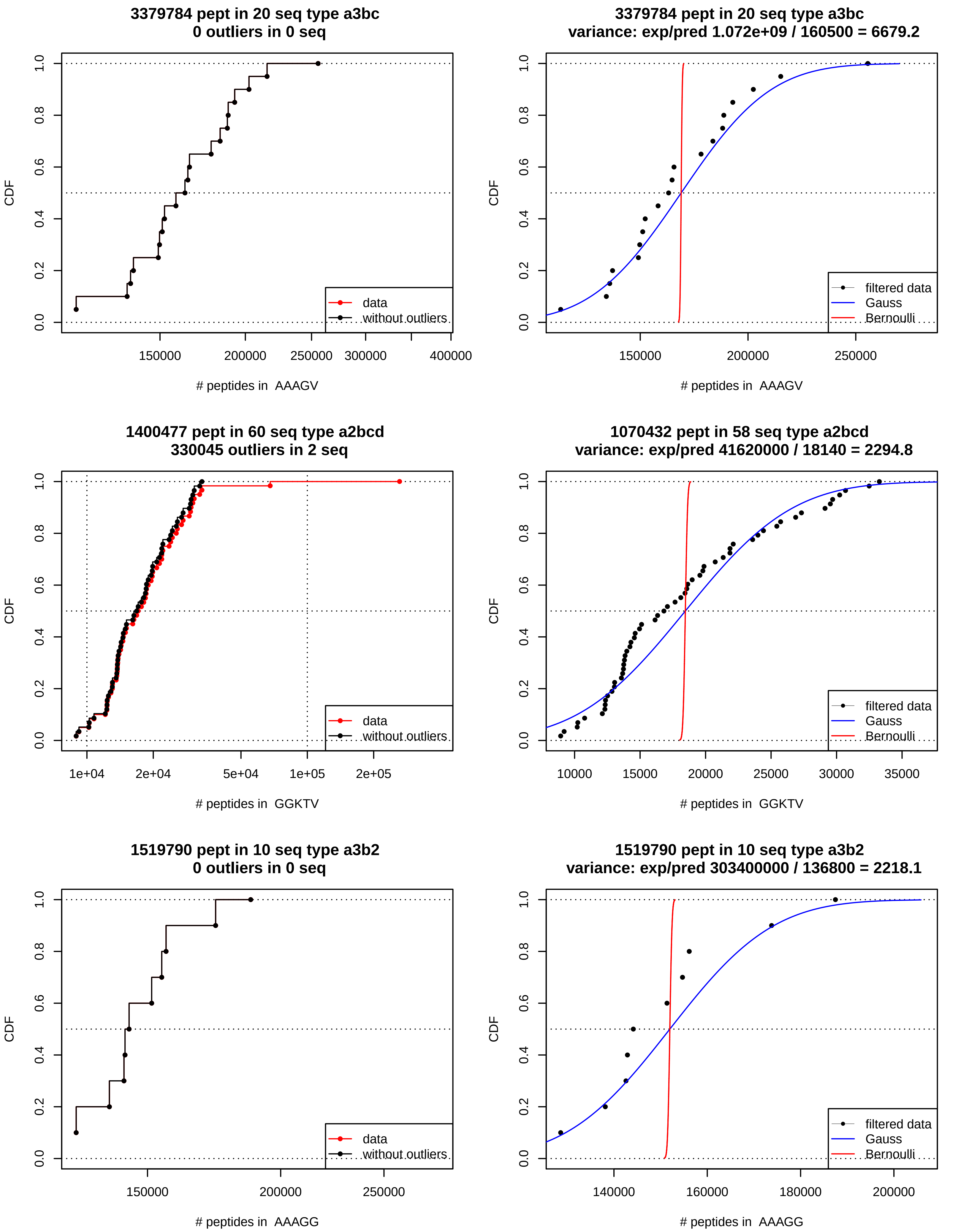

3379784 pept in 20 seq type a3bc
0 outliers in 0 seq
CDF
# peptides in AAAGV
data
without outliers
3379784 pept in 20 seq type a3bc
variance: exp/pred 1.072e+09 / 160500 = 6679.2
CDF
# peptides in AAAGV
filtered data
Gauss
Bernoulli
1400477 pept in 60 seq type a2bcd
330045 outliers in 2 seq
CDF
# peptides in GGKTV
data
without outliers
1070432 pept in 58 seq type a2bcd
variance: exp/pred 41620000 / 18140 = 2294.8
CDF
# peptides in GGKTV
filtered data
Gauss
Bernoulli
1519790 pept in 10 seq type a3b2
0 outliers in 0 seq
CDF
# peptides in AAAGG
data
without outliers
1519790 pept in 10 seq type a3b2
variance: exp/pred 303400000 / 136800 = 2218.1
CDF
# peptides in AAAGG
filtered data
Gauss
Bernoulli

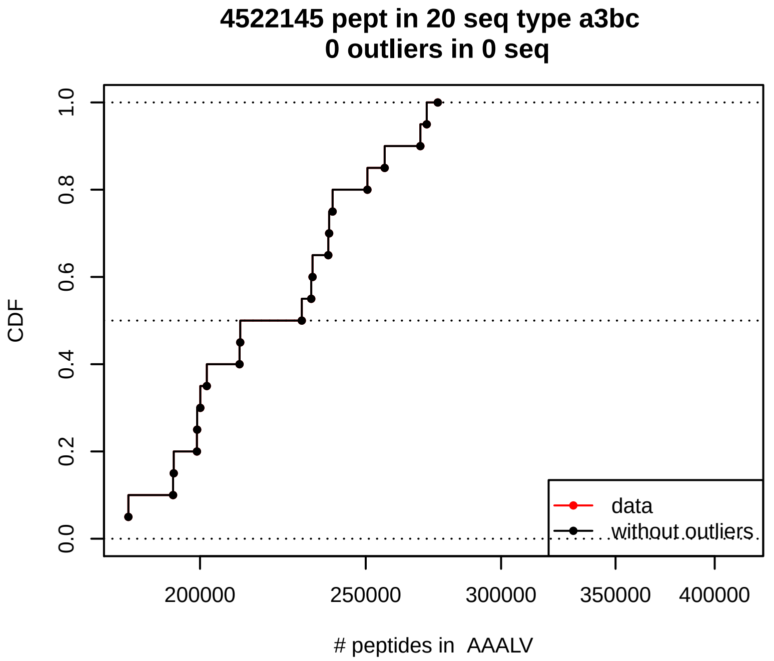

4522145 pept in 20 seq type a3bc
0 outliers in 0 seq
CDF
# peptides in AAALV
data
without outliers


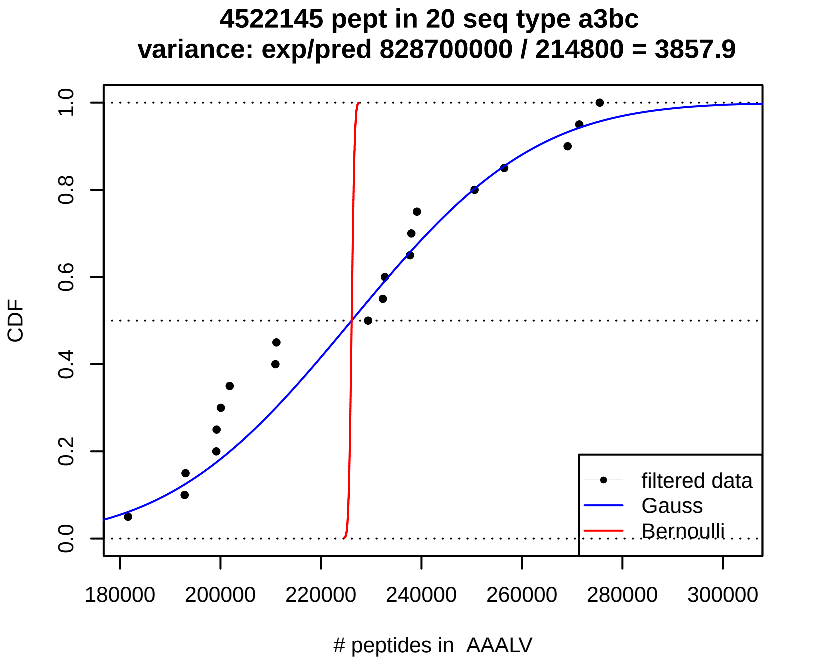

4522145 pept in 20 seq type a3bc
variance: exp/pred 828700000 / 214800 = 3857.9
CDF
# peptides in AAALV
filtered data
Gauss
Bernoulli


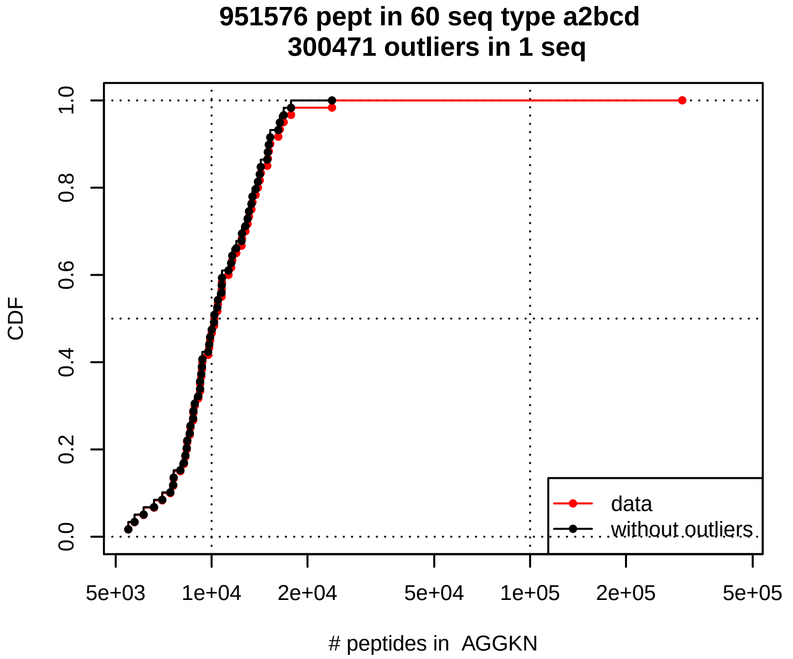

951576 pept in 60 seq type a2bcd
300471 outliers in 1 seq
CDF
# peptides in AGGKN
data
without outliers


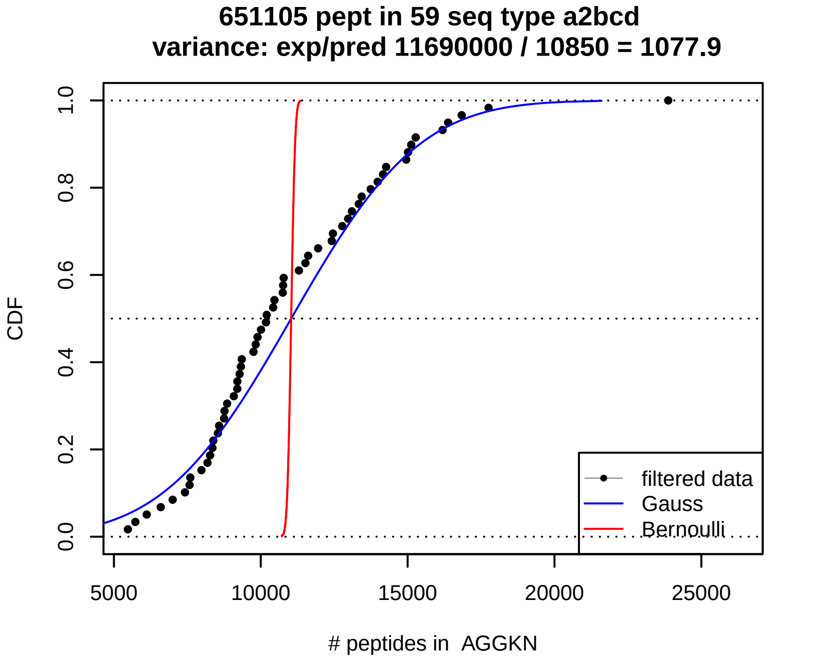

651105 pept in 59 seq type a2bcd
variance: exp/pred 11690000 / 10850 = 1077.9
CDF
# peptides in AGGKN
filtered data
Gauss
Bernoulli


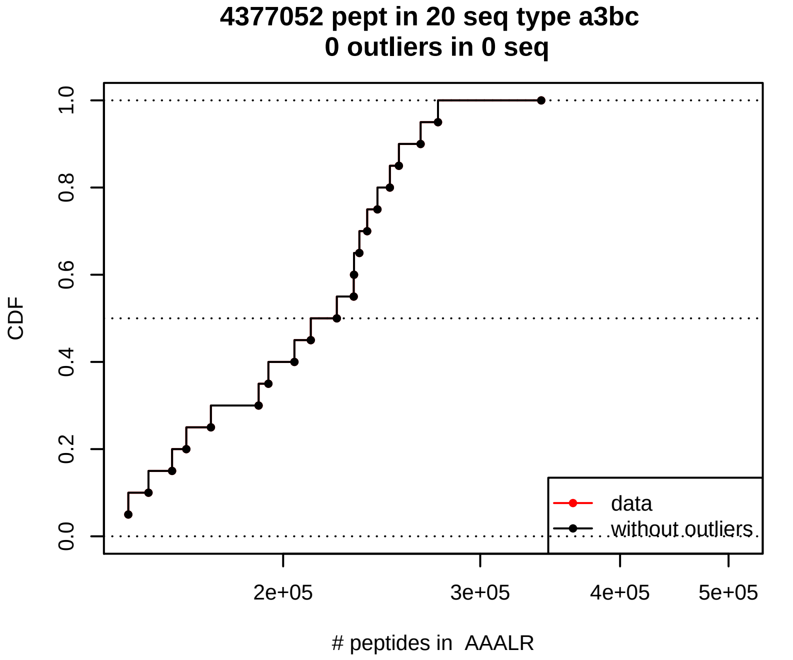

4377052 pept in 20 seq type a3bc
0 outliers in 0 seq
CDF
# peptides in AAALR
data
without outliers


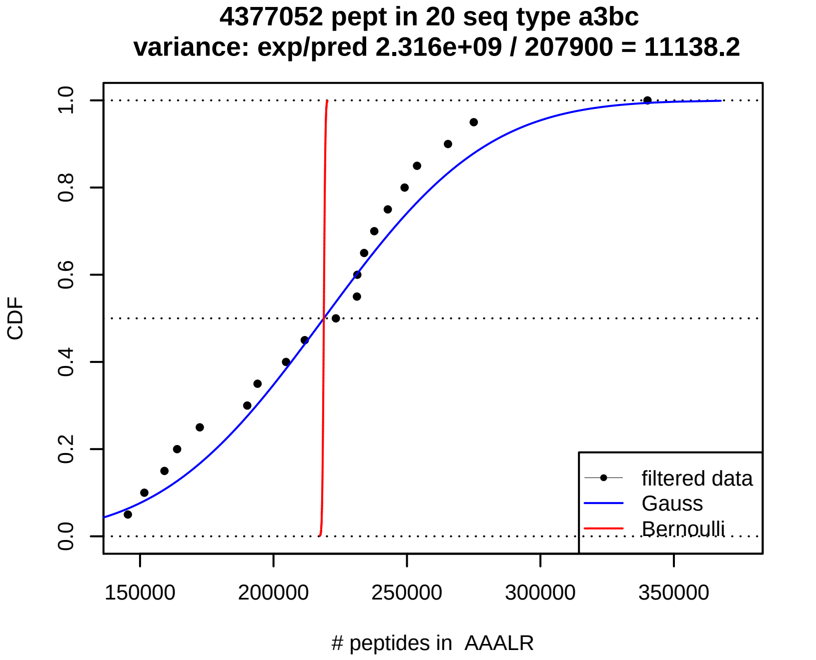

4377052 pept in 20 seq type a3bc
variance: exp/pred 2.316e+09 / 207900 = 11138.2
CDF
# peptides in AAALR
filtered data
Gauss
Bernoulli

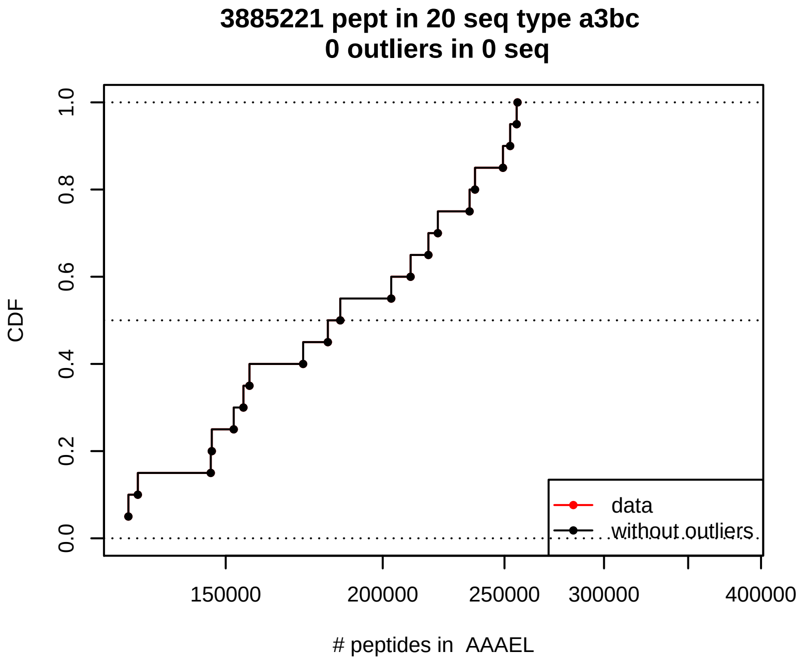
3885221 pept in 20 seq type a3bc
0 outliers in 0 seq
CDF
# peptides in AAAEL
data
without outliers

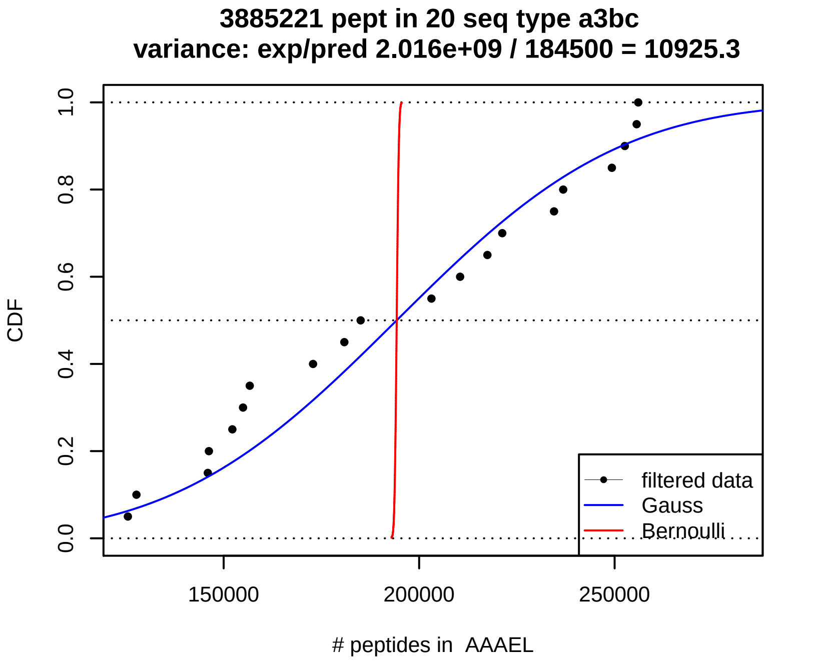
3885221 pept in 20 seq type a3bc
variance: exp/pred 2.016e+09 / 184500 = 10925.3
CDF
# peptides in AAAEL
filtered data
Gauss
Bernoulli

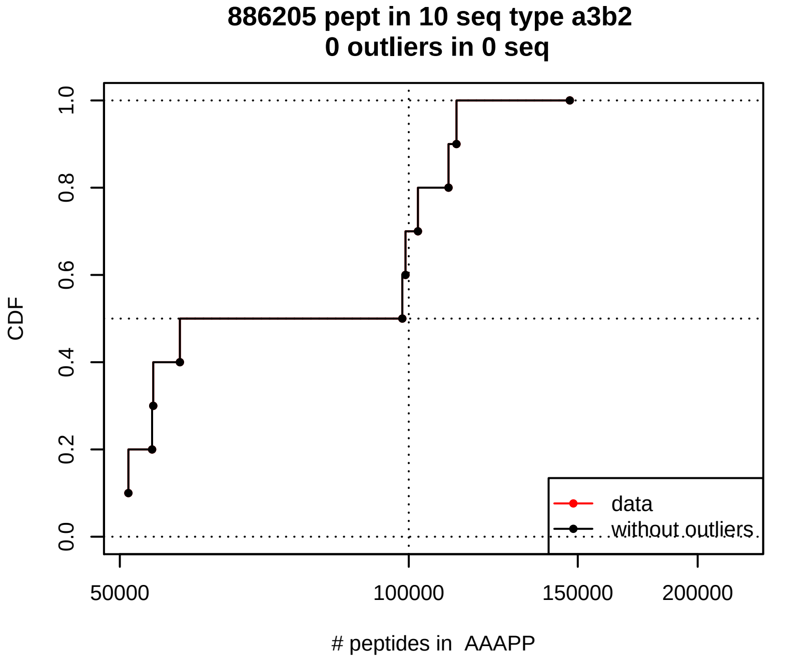
886205 pept in 10 seq type a3b2
0 outliers in 0 seq
CDF
# peptides in AAAPP
data
without outliers

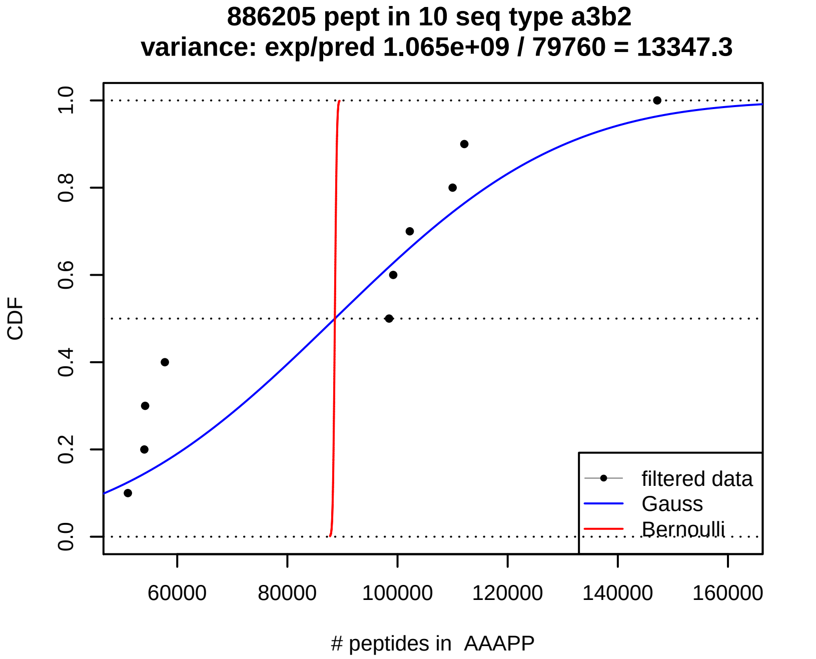
886205 pept in 10 seq type a3b2
variance: exp/pred 1.065e+09 / 79760 = 13347.3
CDF
# peptides in AAAPP
filtered data
Gauss
Bernoulli

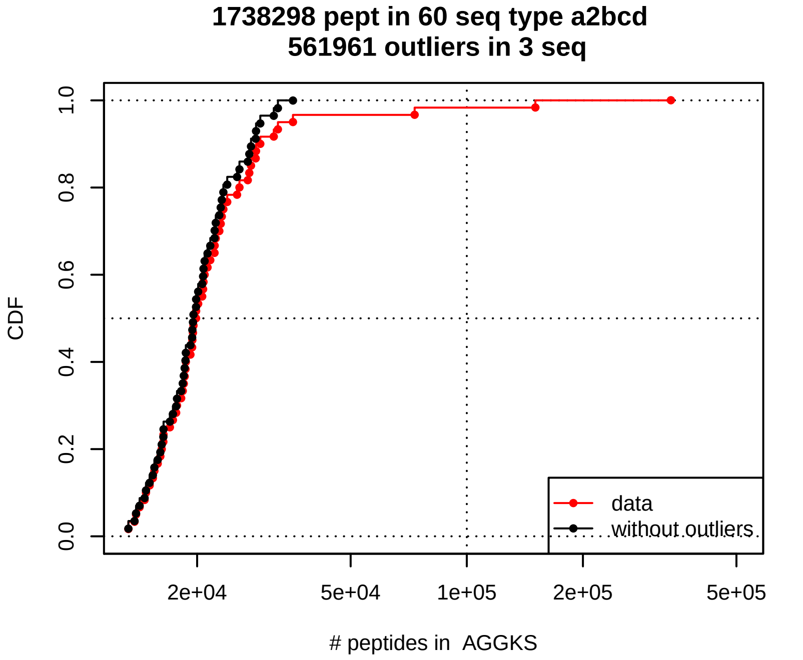
1738298 pept in 60 seq type a2bcd
561961 outliers in 3 seq
CDF
# peptides in AGGKS
data
without outliers

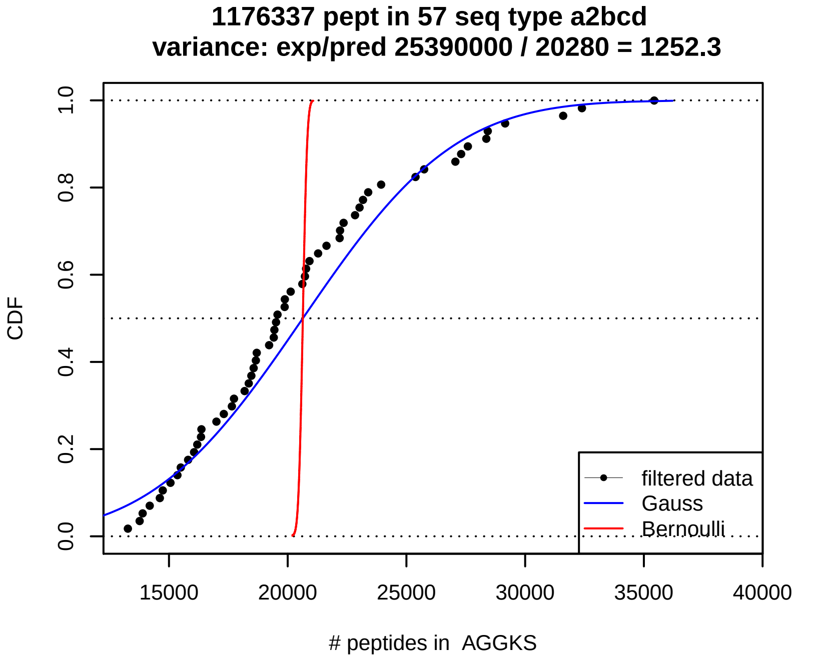
1176337 pept in 57 seq type a2bcd
variance: exp/pred 25390000 / 20280 = 1252.3
CDF
# peptides in AGGKS
filtered data
Gauss
Bernoulli

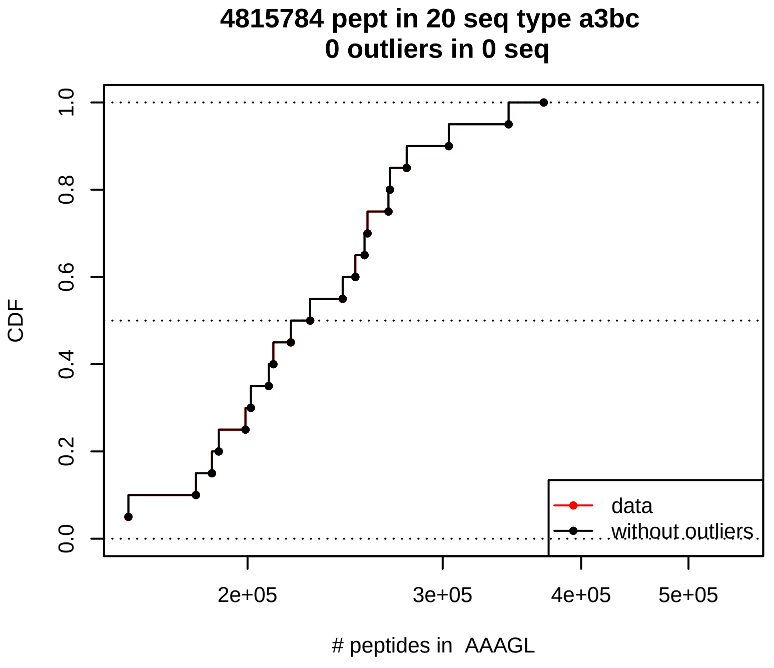
4815784 pept in 20 seq type a3bc
0 outliers in 0 seq
CDF
0.0
0.2
0.4
0.6
0.8
1.0
2e+05
3e+05
4e+05
5e+05
# peptides in AAAGL
data
without outliers

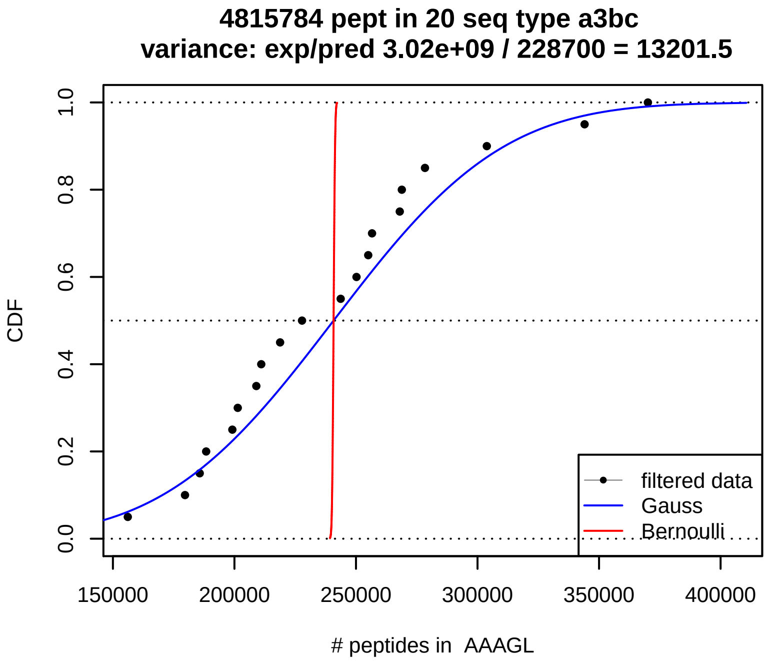
4815784 pept in 20 seq type a3bc
variance: exp/pred 3.02e+09 / 228700 = 13201.5
CDF
0.0
0.2
0.4
0.6
0.8
1.0
150000
200000
250000
300000
350000
400000
# peptides in AAAGL
filtered data
Gauss
Bernoulli

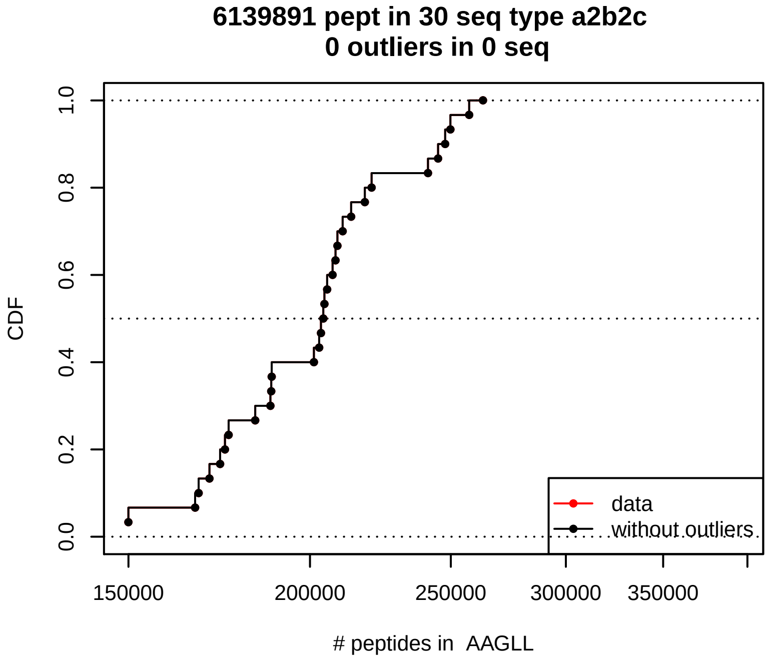
6139891 pept in 30 seq type a2b2c
0 outliers in 0 seq
CDF
0.0
0.2
0.4
0.6
0.8
1.0
150000
200000
250000
300000
350000
# peptides in AAGLL
data
without outliers

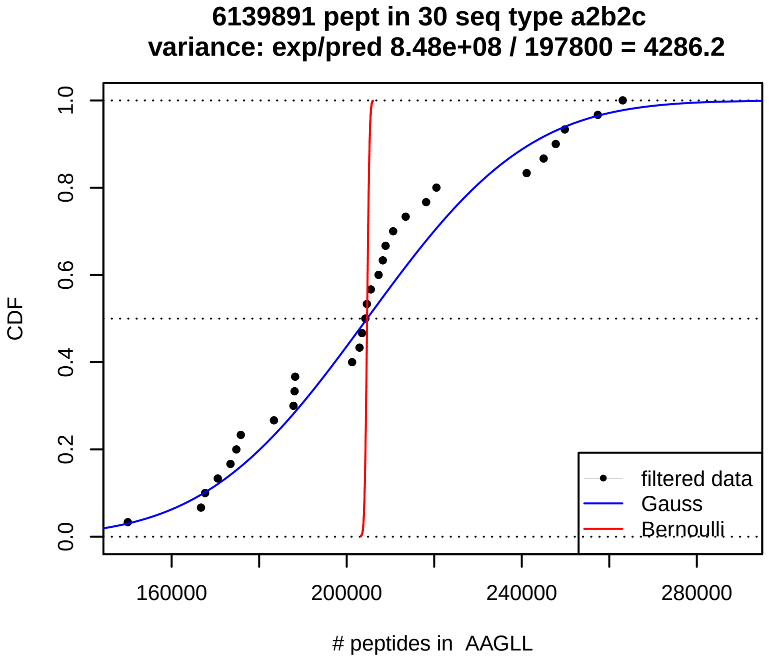
6139891 pept in 30 seq type a2b2c
variance: exp/pred 8.48e+08 / 197800 = 4286.2
CDF
0.0
0.2
0.4
0.6
0.8
1.0
160000
200000
240000
280000
# peptides in AAGLL
filtered data
Gauss
Bernoulli

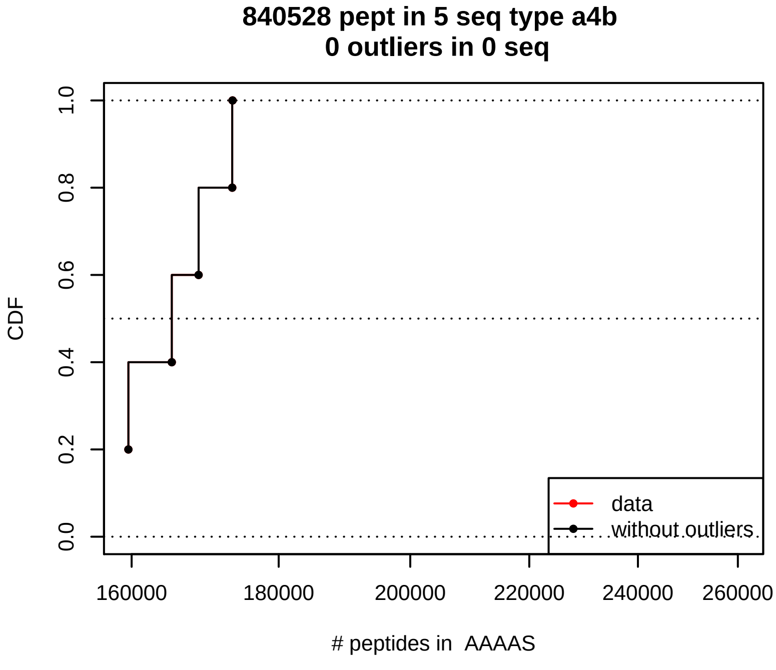
840528 pept in 5 seq type a4b
0 outliers in 0 seq
CDF
0.0
0.2
0.4
0.6
0.8
1.0
160000
180000
200000
220000
240000
260000
# peptides in AAAAS
data
without outliers

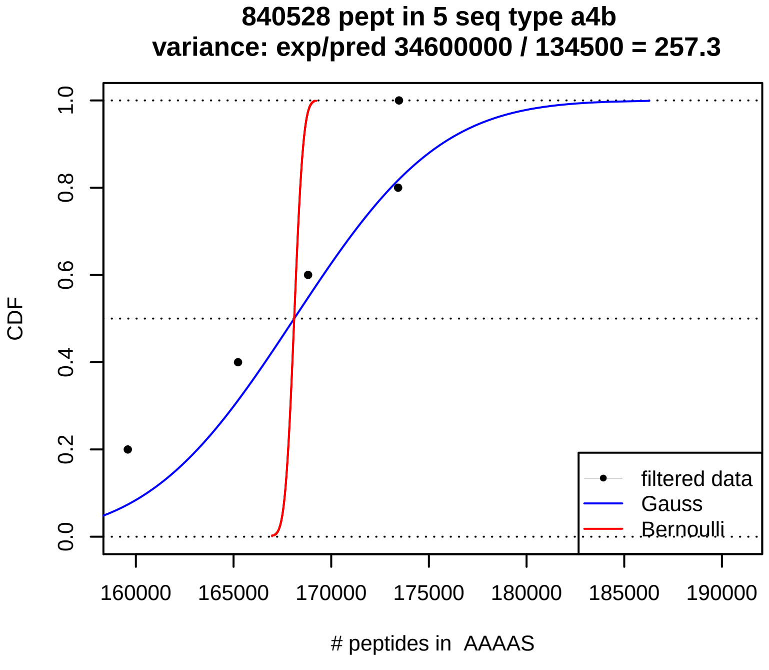
840528 pept in 5 seq type a4b
variance: exp/pred 34600000 / 134500 = 257.3
CDF
0.0
0.2
0.4
0.6
0.8
1.0
160000
165000
170000
175000
180000
185000
190000
# peptides in AAAAS
filtered data
Gauss
Bernoulli

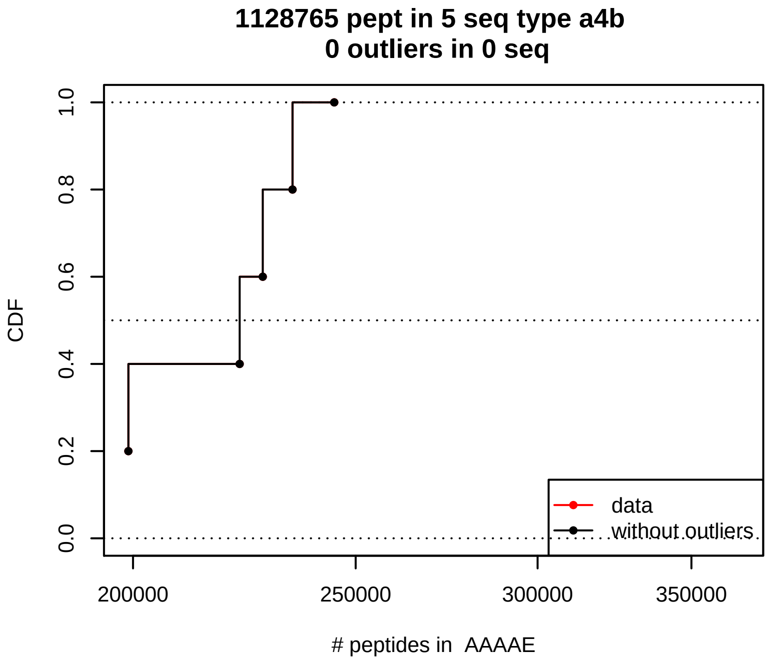
1128765 pept in 5 seq type a4b
0 outliers in 0 seq
CDF
# peptides in AAAAE
data
without outliers

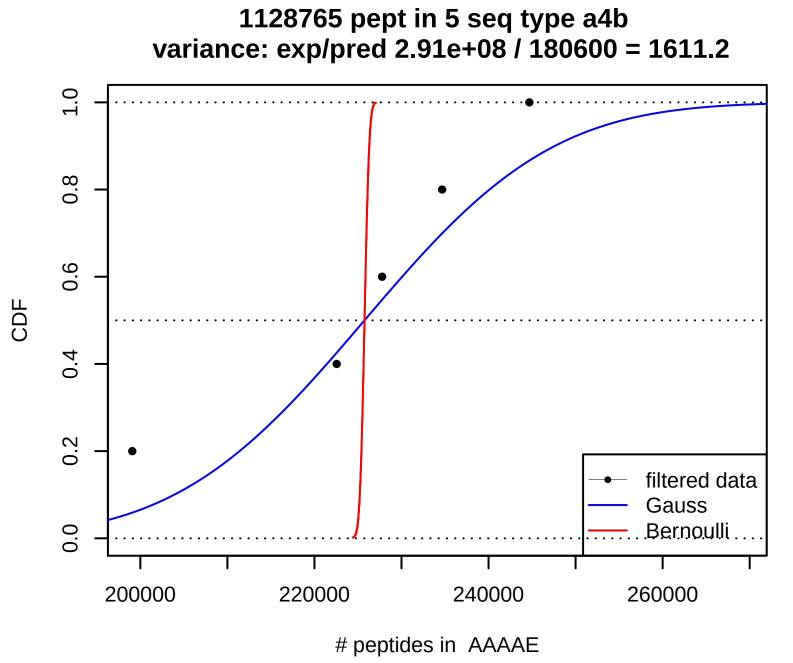
1128765 pept in 5 seq type a4b
variance: exp/pred 2.91e+08 / 180600 = 1611.2
CDF
# peptides in AAAAE
filtered data
Gauss
Bernoulli

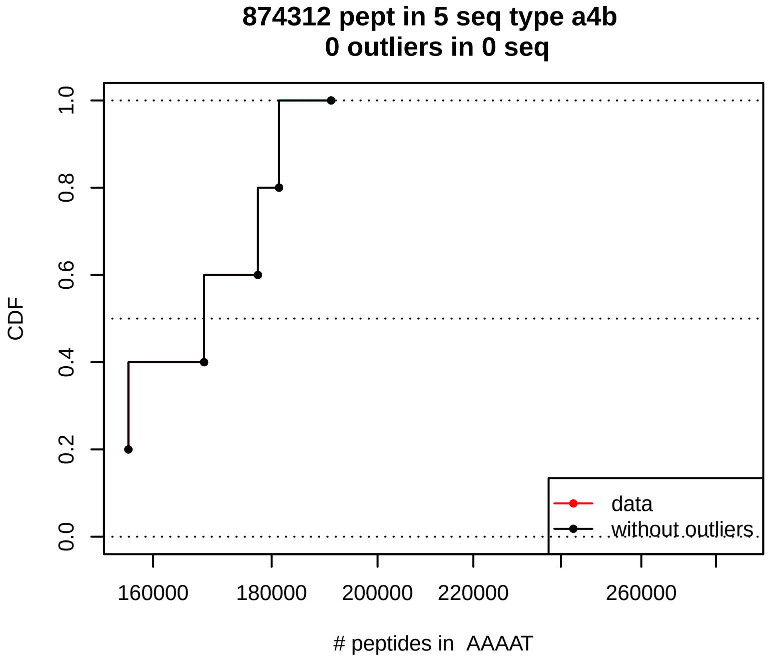
874312 pept in 5 seq type a4b
0 outliers in 0 seq
CDF
# peptides in AAAAT
data
without outliers

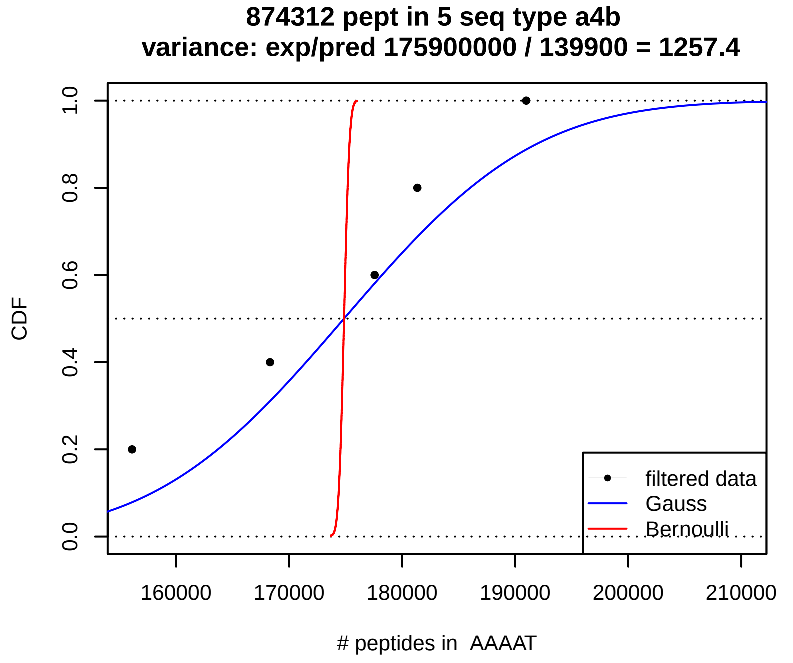
874312 pept in 5 seq type a4b
variance: exp/pred 175900000 / 139900 = 1257.4
CDF
# peptides in AAAAT
filtered data
Gauss
Bernoulli

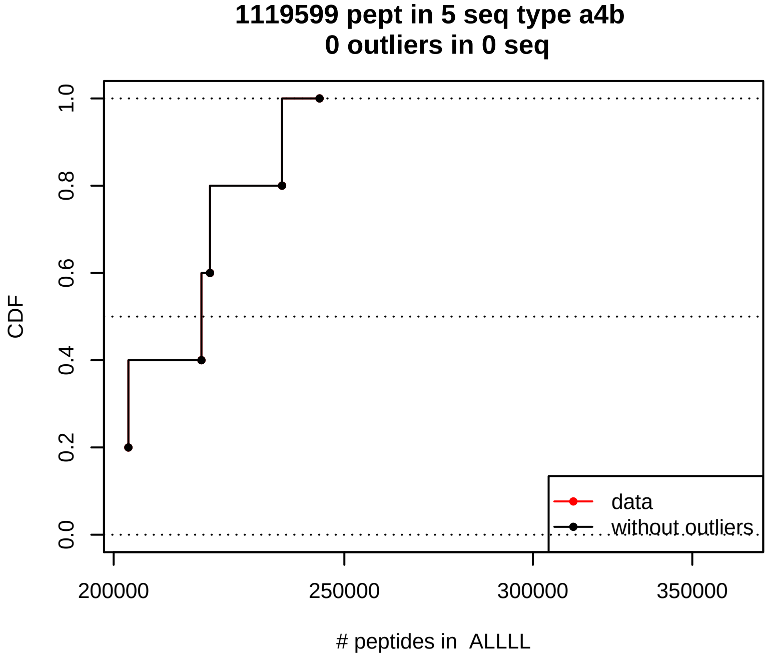
1119599 pept in 5 seq type a4b
0 outliers in 0 seq
CDF
# peptides in ALLLL
data
without outliers

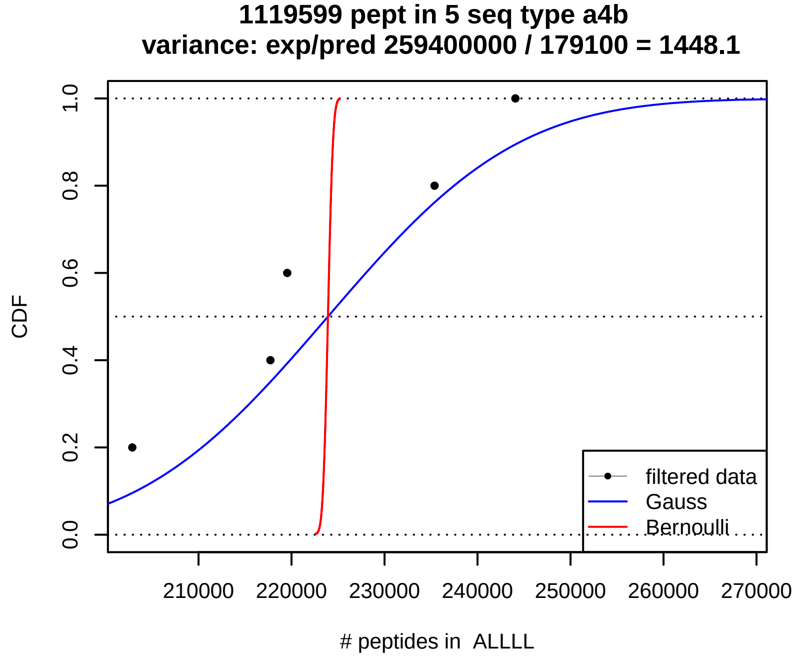
1119599 pept in 5 seq type a4b
variance: exp/pred 259400000 / 179100 = 1448.1
CDF
# peptides in ALLLL
filtered data
Gauss
Bernoulli

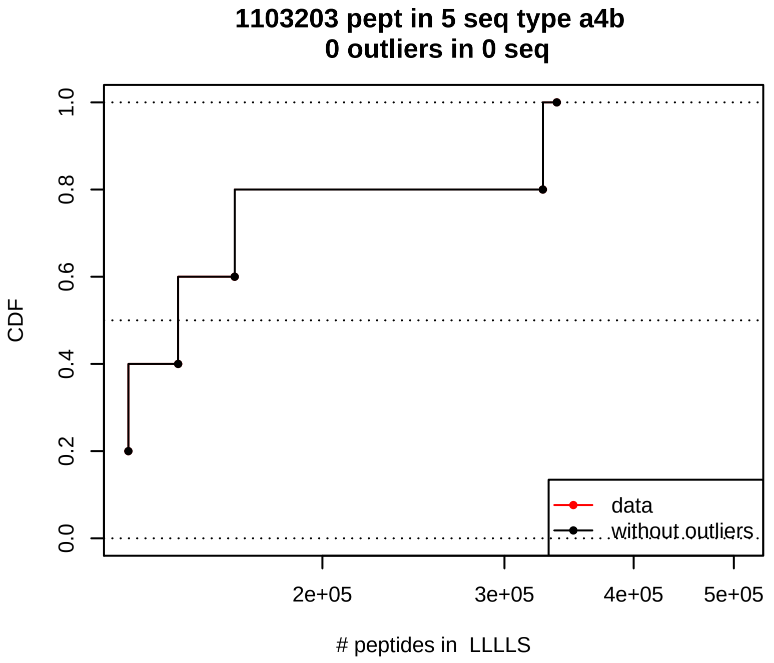
1103203 pept in 5 seq type a4b
0 outliers in 0 seq
CDF
# peptides in LLLLS
data
without outliers

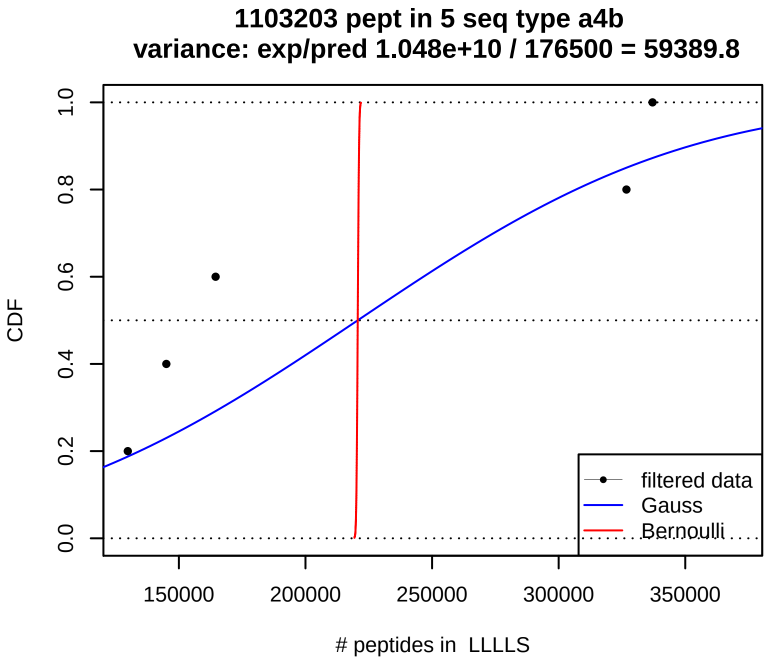
1103203 pept in 5 seq type a4b
variance: exp/pred 1.048e+10 / 176500 = 59389.8
CDF
# peptides in LLLLS
filtered data
Gauss
Bernoulli

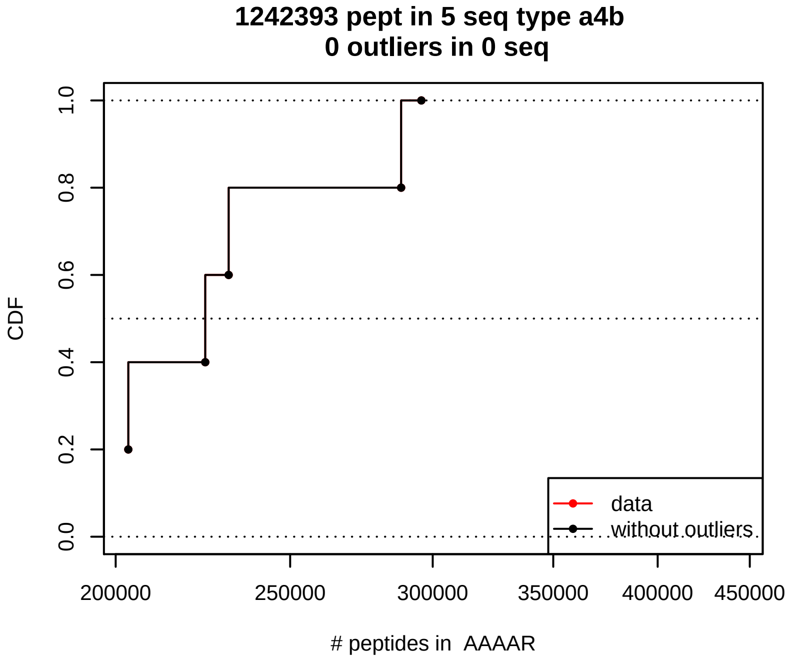
1242393 pept in 5 seq type a4b
0 outliers in 0 seq
CDF
# peptides in AAAAR
data
without outliers

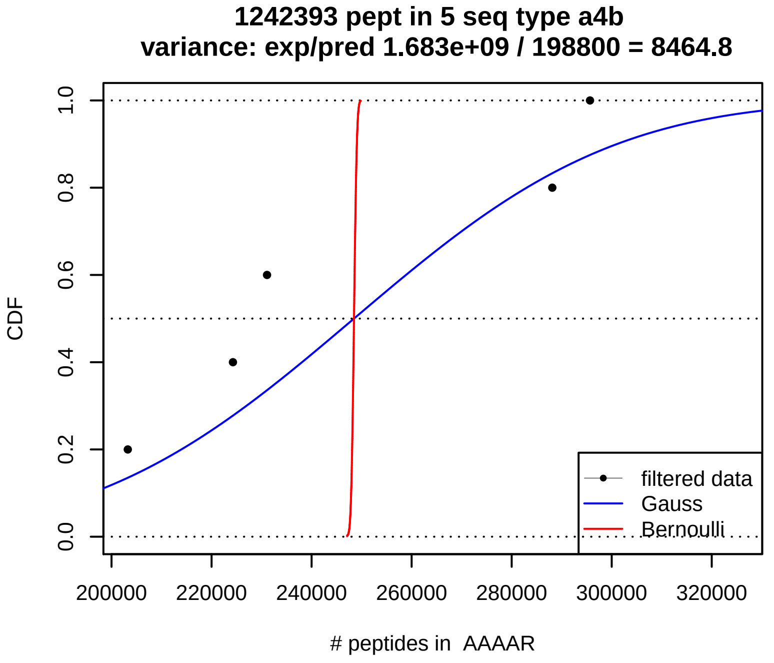
1242393 pept in 5 seq type a4b
variance: exp/pred 1.683e+09 / 198800 = 8464.8
CDF
# peptides in AAAAR
filtered data
Gauss
Bernoulli

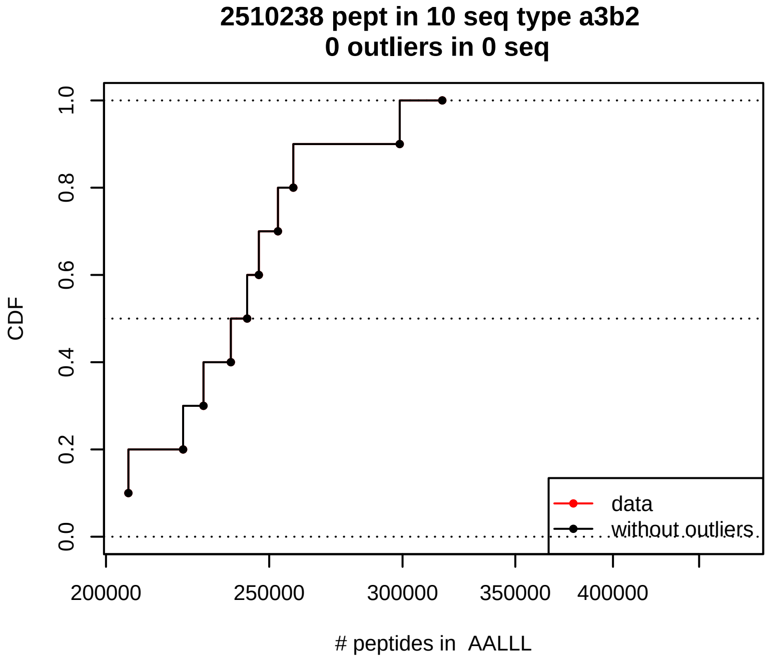
2510238 pept in 10 seq type a3b2
0 outliers in 0 seq
CDF
# peptides in AALLL
data
without outliers

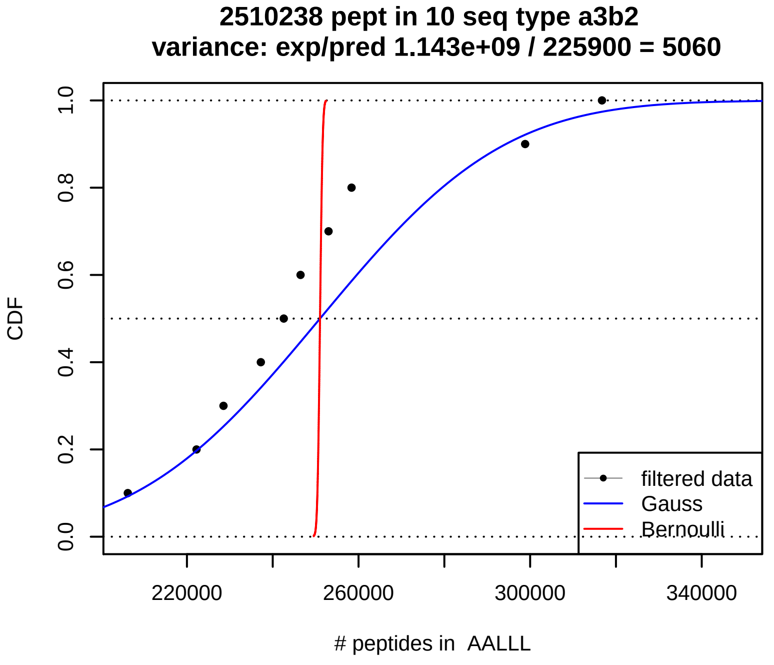
2510238 pept in 10 seq type a3b2
variance: exp/pred 1.143e+09 / 225900 = 5060
CDF
# peptides in AALLL
filtered data
Gauss
Bernoulli

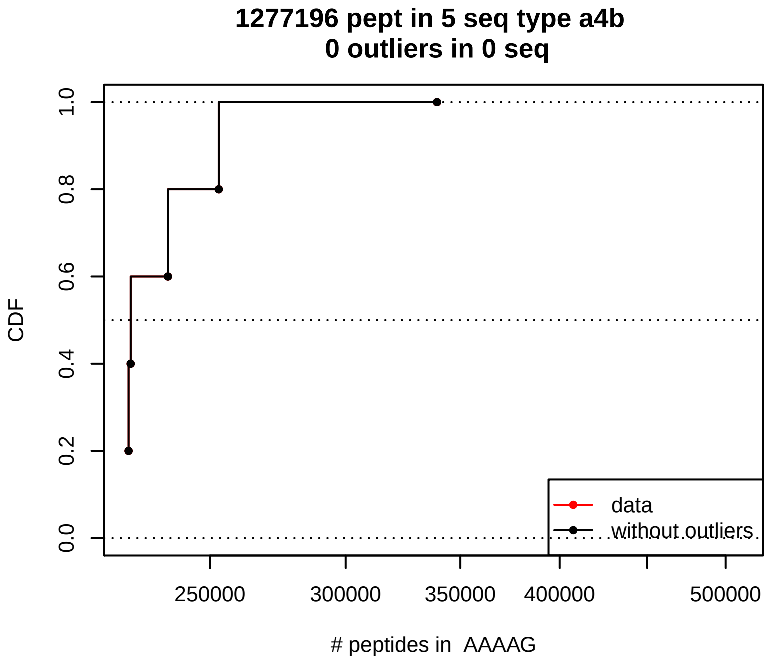
1277196 pept in 5 seq type a4b
0 outliers in 0 seq
CDF
0.0
0.2
0.4
0.6
0.8
1.0
data
without outliers
250000
300000
350000
400000
500000
# peptides in AAAAG

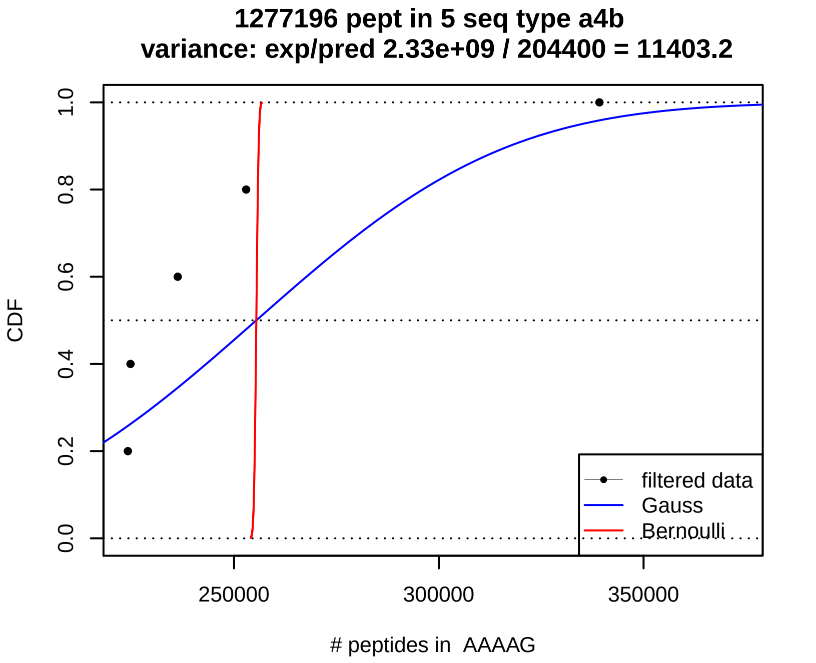
1277196 pept in 5 seq type a4b
variance: exp/pred 2.33e+09 / 204400 = 11403.2
CDF
0.0
0.2
0.4
0.6
0.8
1.0
filtered data
Gauss
Bernoulli
250000
300000
350000
# peptides in AAAAG

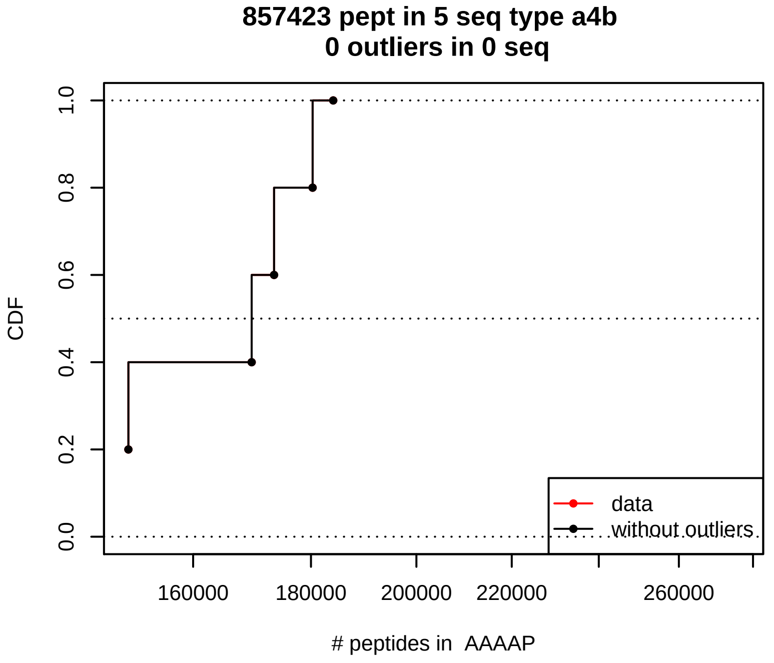
857423 pept in 5 seq type a4b
0 outliers in 0 seq
CDF
0.0
0.2
0.4
0.6
0.8
1.0
data
without outliers
160000
180000
200000
220000
260000
# peptides in AAAAP

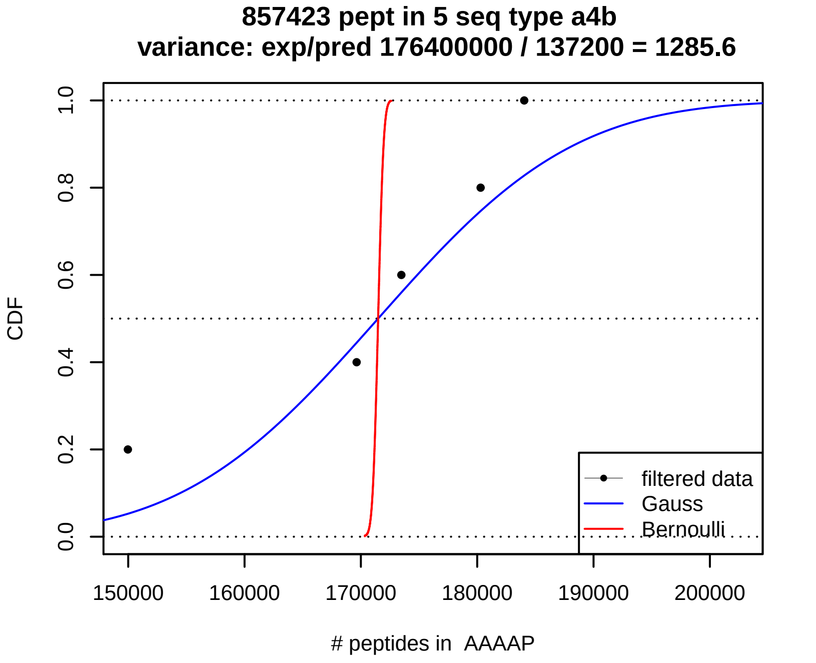
857423 pept in 5 seq type a4b
variance: exp/pred 176400000 / 137200 = 1285.6
CDF
0.0
0.2
0.4
0.6
0.8
1.0
filtered data
Gauss
Bernoulli
150000
160000
170000
180000
190000
200000
# peptides in AAAAP

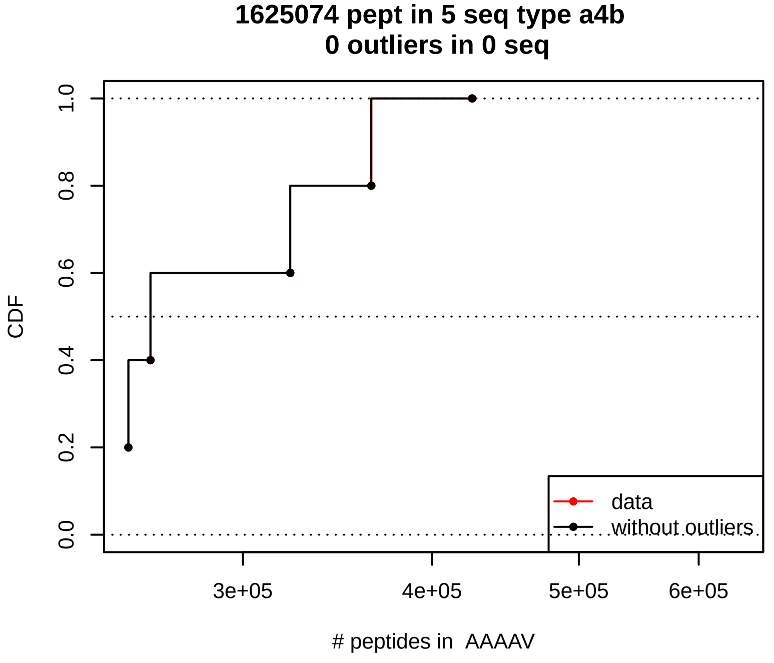
1625074 pept in 5 seq type a4b
0 outliers in 0 seq
CDF
0.0
0.2
0.4
0.6
0.8
1.0
data
without outliers
3e+05
4e+05
5e+05
6e+05
# peptides in AAAAV

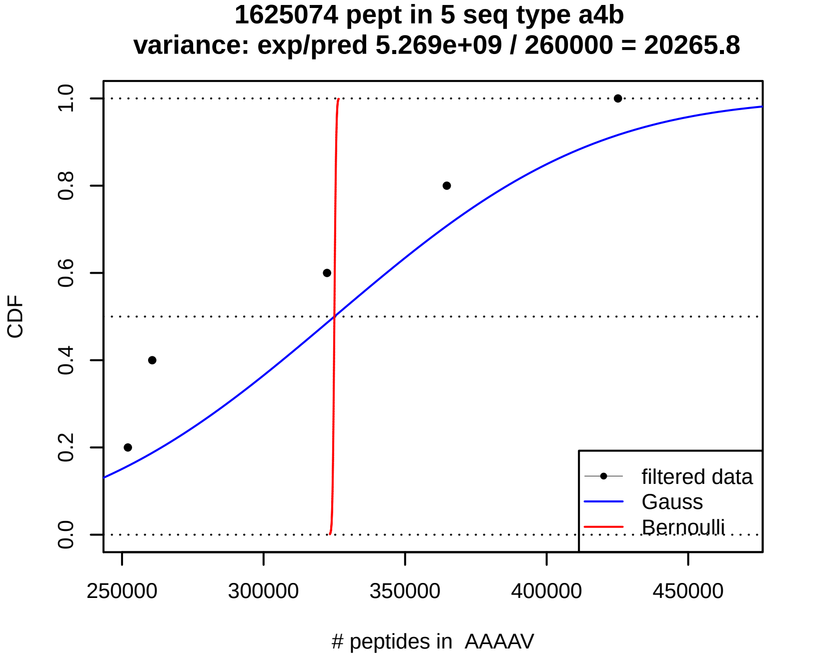
1625074 pept in 5 seq type a4b
variance: exp/pred 5.269e+09 / 260000 = 20265.8
CDF
0.0
0.2
0.4
0.6
0.8
1.0
filtered data
Gauss
Bernoulli
250000
300000
350000
400000
450000
# peptides in AAAAV

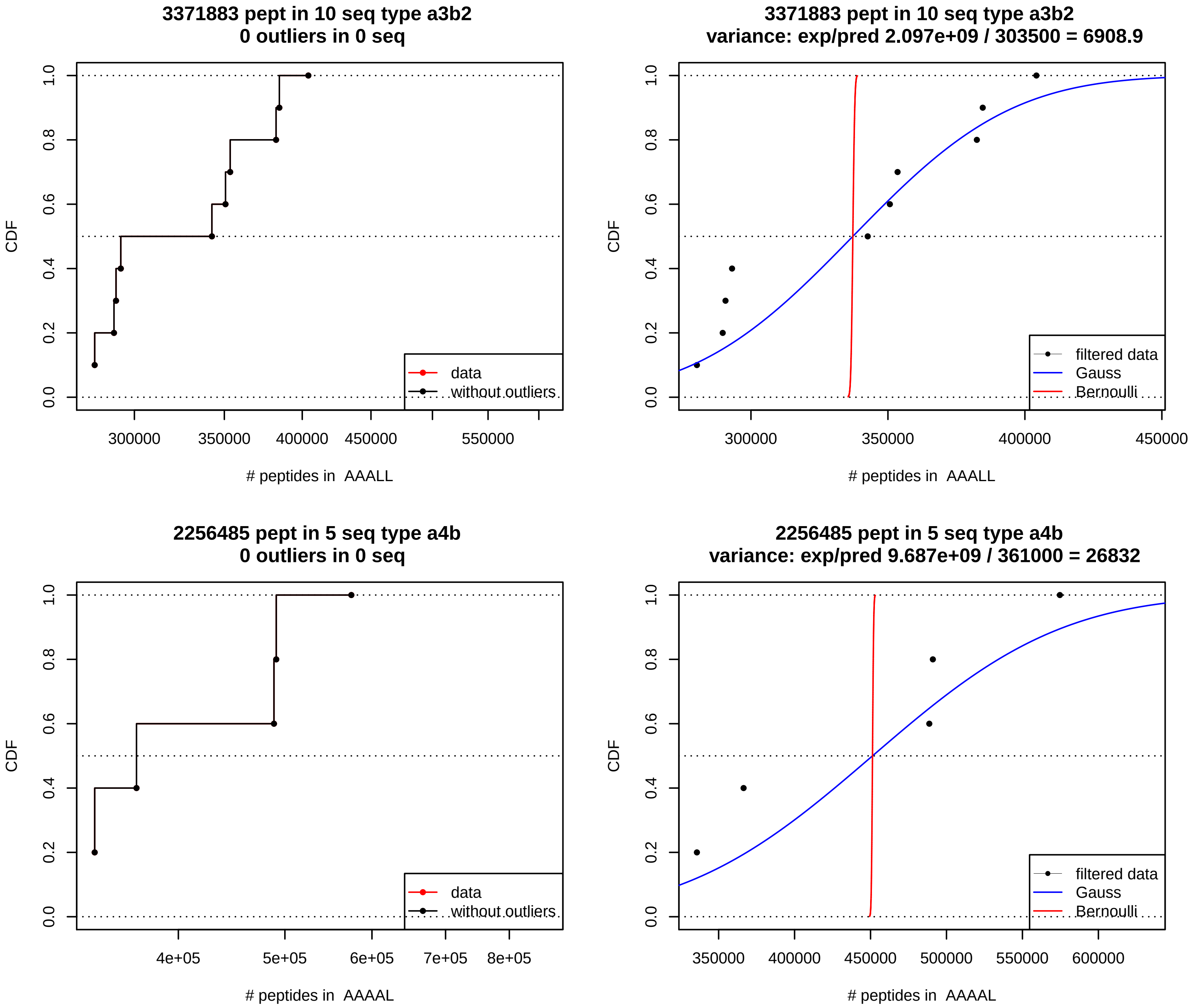


Suppl Fig. S1B
The CDFs of peptide occurrences for the representative permutation classes belonging to various pentapeptide categories are shown before (red circles) and after (black circles) outlier filtering (left panels). The distributions of peptide occurrences after outlier filtering compared with the Normal (Gauss) and the expected Binomial (Bernoulli) distributions are shown in right panels. Summary statistics concerning contribution of outliers and variance are shown above each panel. The outliers were analyzed at 0.05 (Fig S1A) or 0.001 (Fig S1B) significance level.

Suppl. Figure S2
N observed vs N expected ratios (see Methods), for domain and non-domain regions. Colouring by hydrophobocity scale (Guy, 1985) arithmetic average is taken when two points overlap. As in Fig. 2B., but human proteins only.

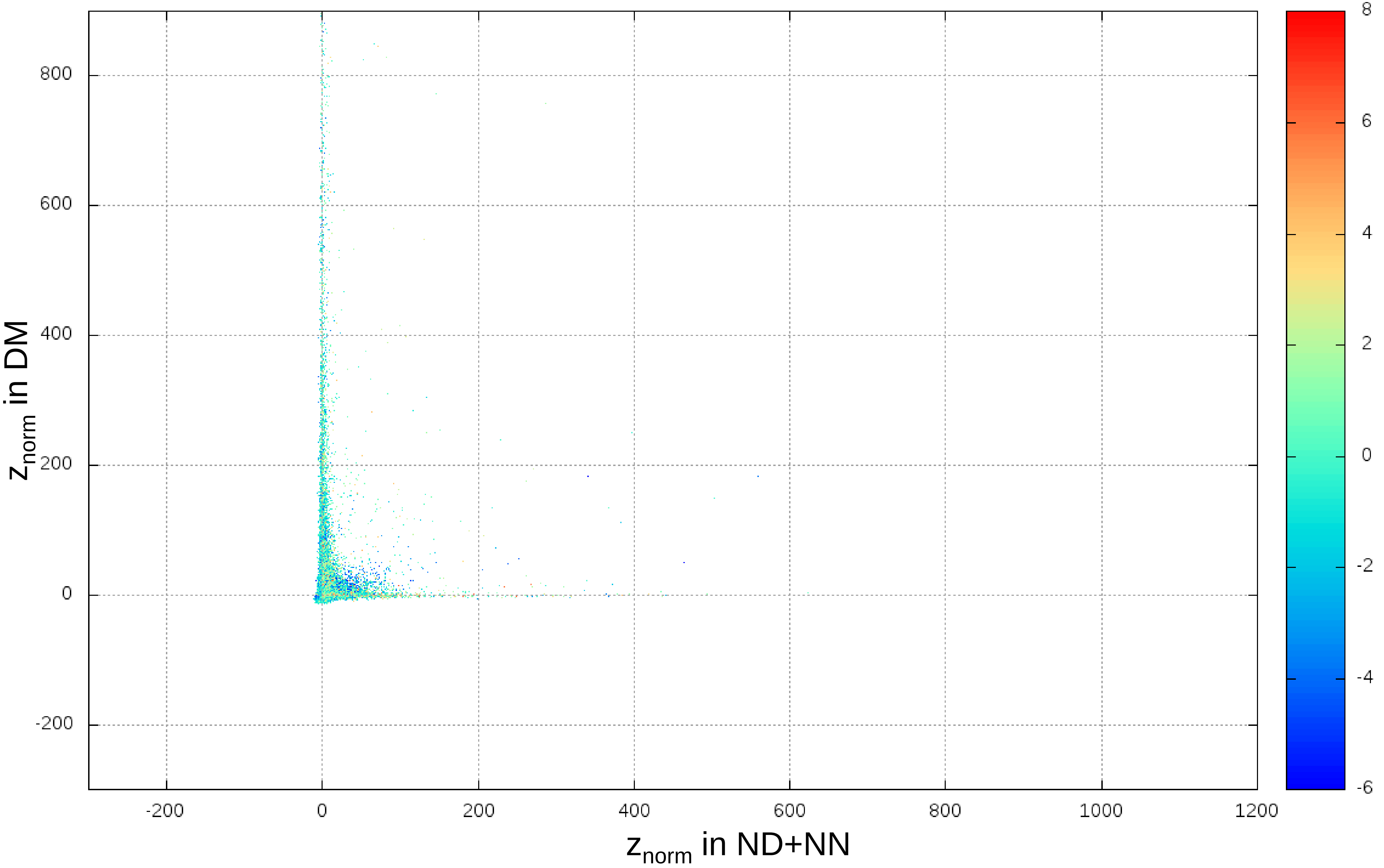

Suppl. Figure S3
Cumulative distribution functions (CDFs) of pentapeptide occurrences for selected permutation groups for domain regions (DM).
For each group, all permutants are shown.

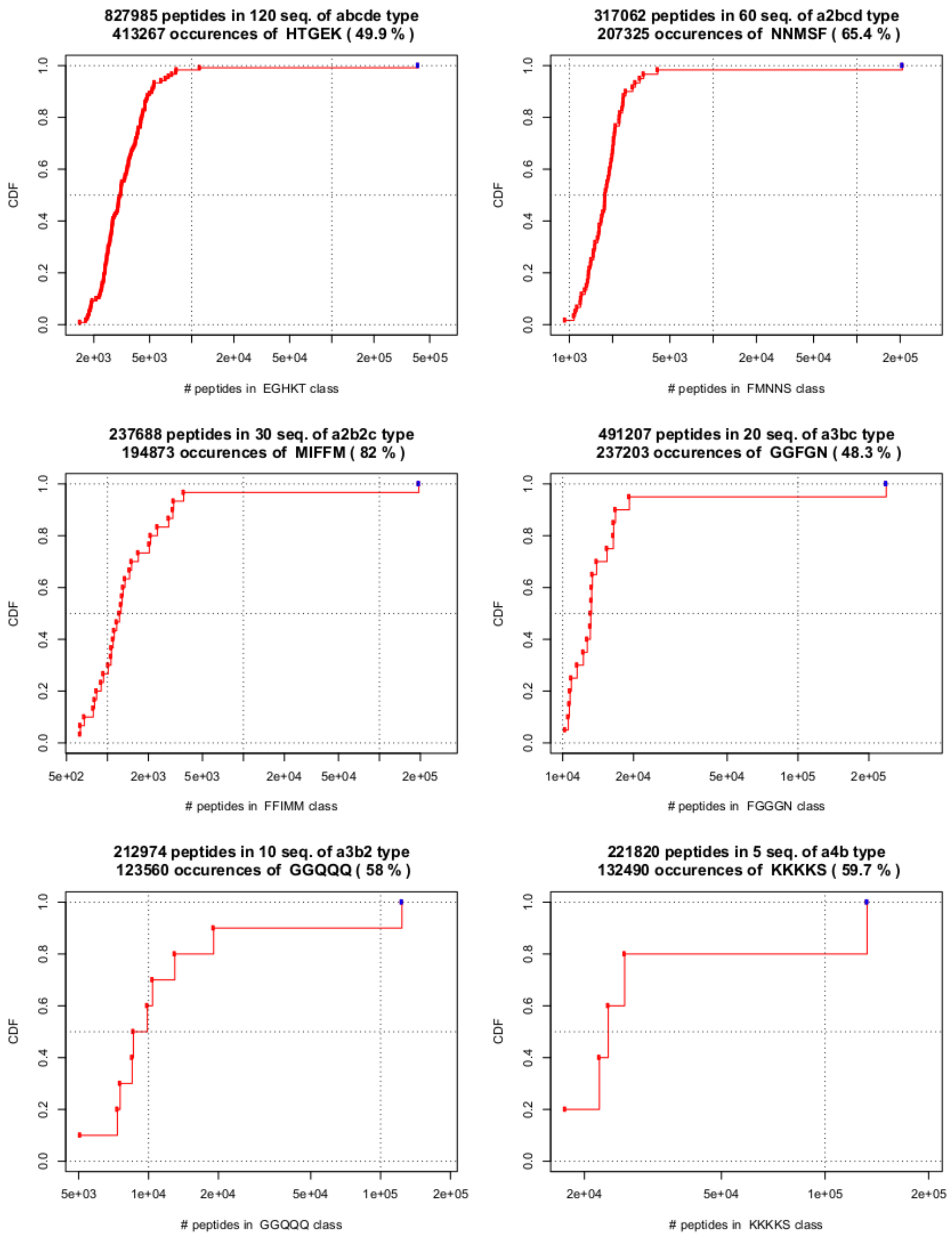

Suppl. Figure S4
Cumulative distribution functions (CDFs) of pentapeptide occurrences for selected permutation groups for non-domain regions (ND+NN).
For each group, all permutants are shown.

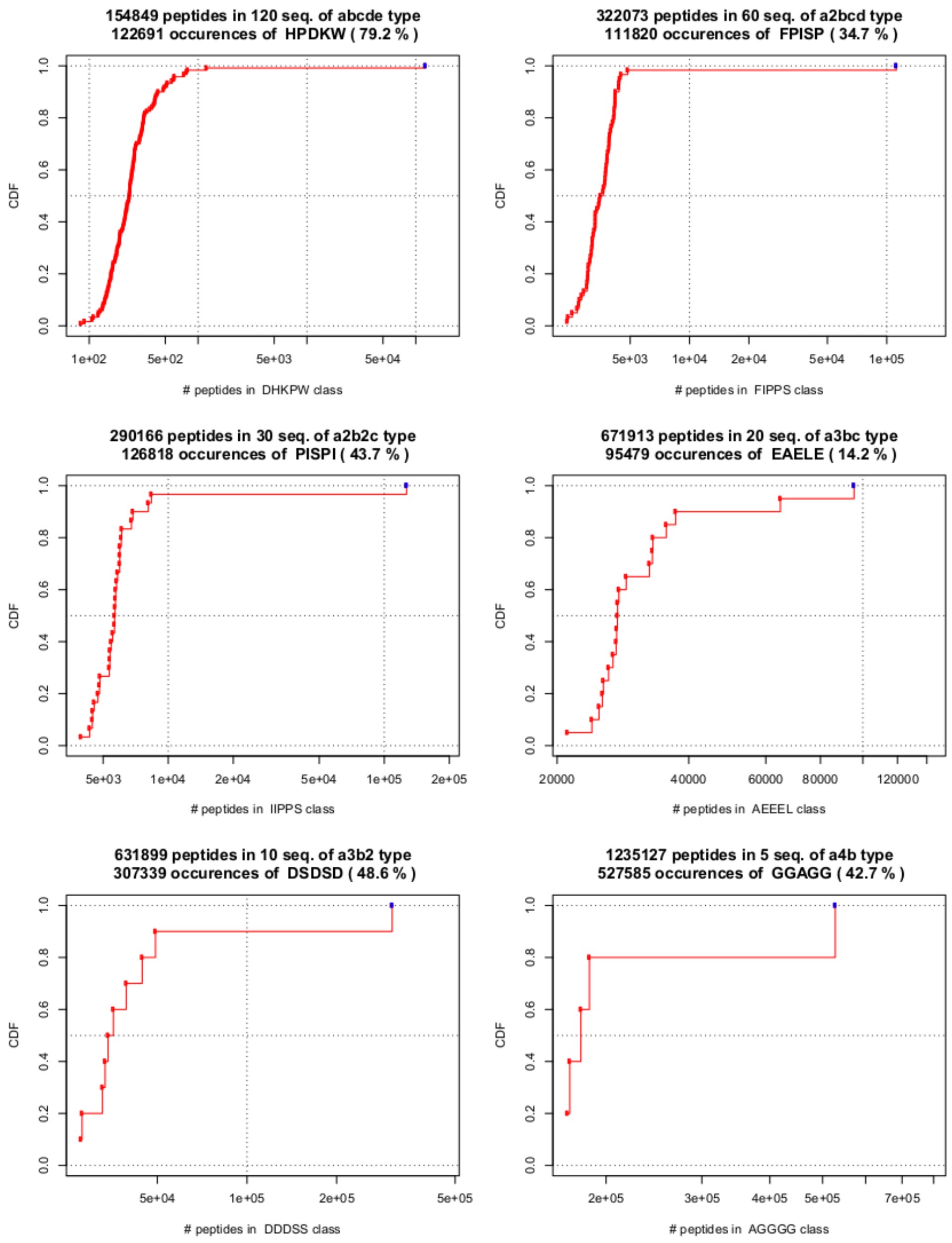

# Suppl. Fig. S5A. Gibbs clustering, DM outliers

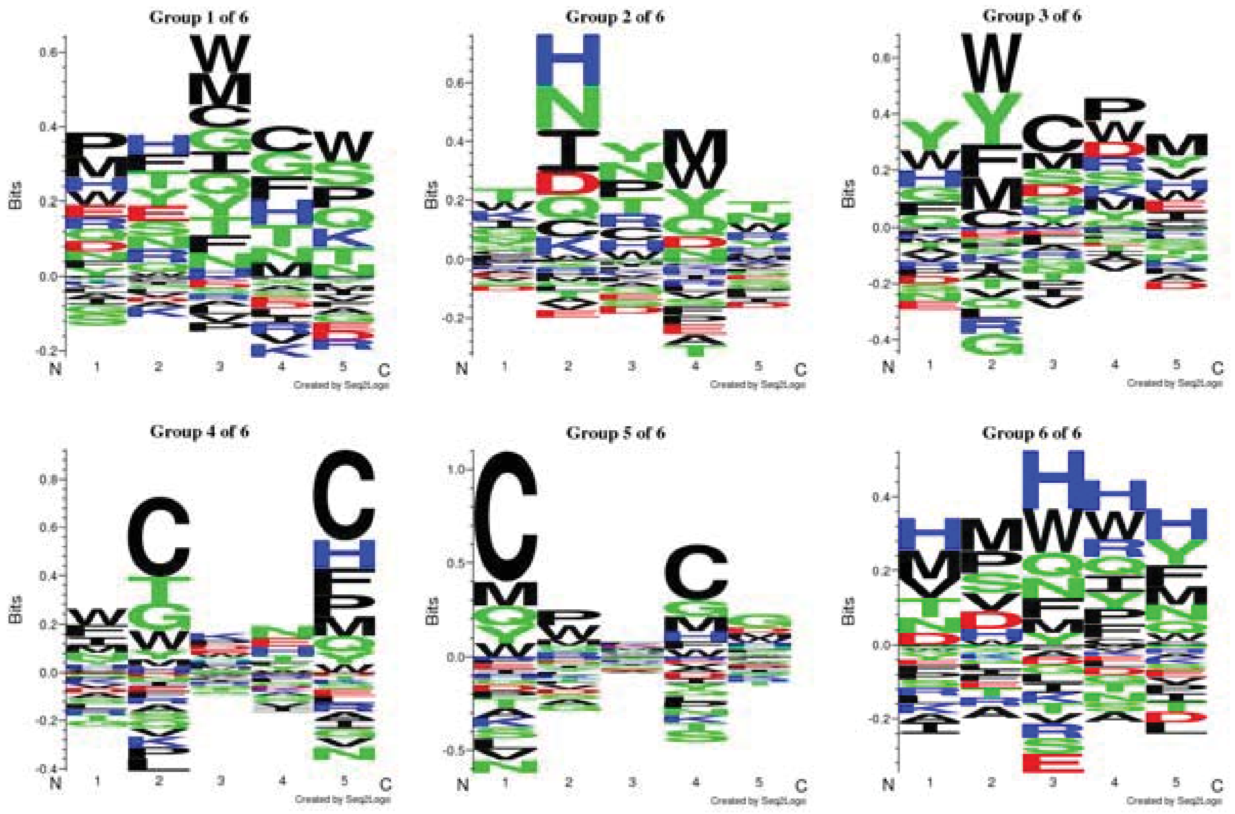

# Suppl. Fig. S5B. Gibbs clustering, ND+NN outliers

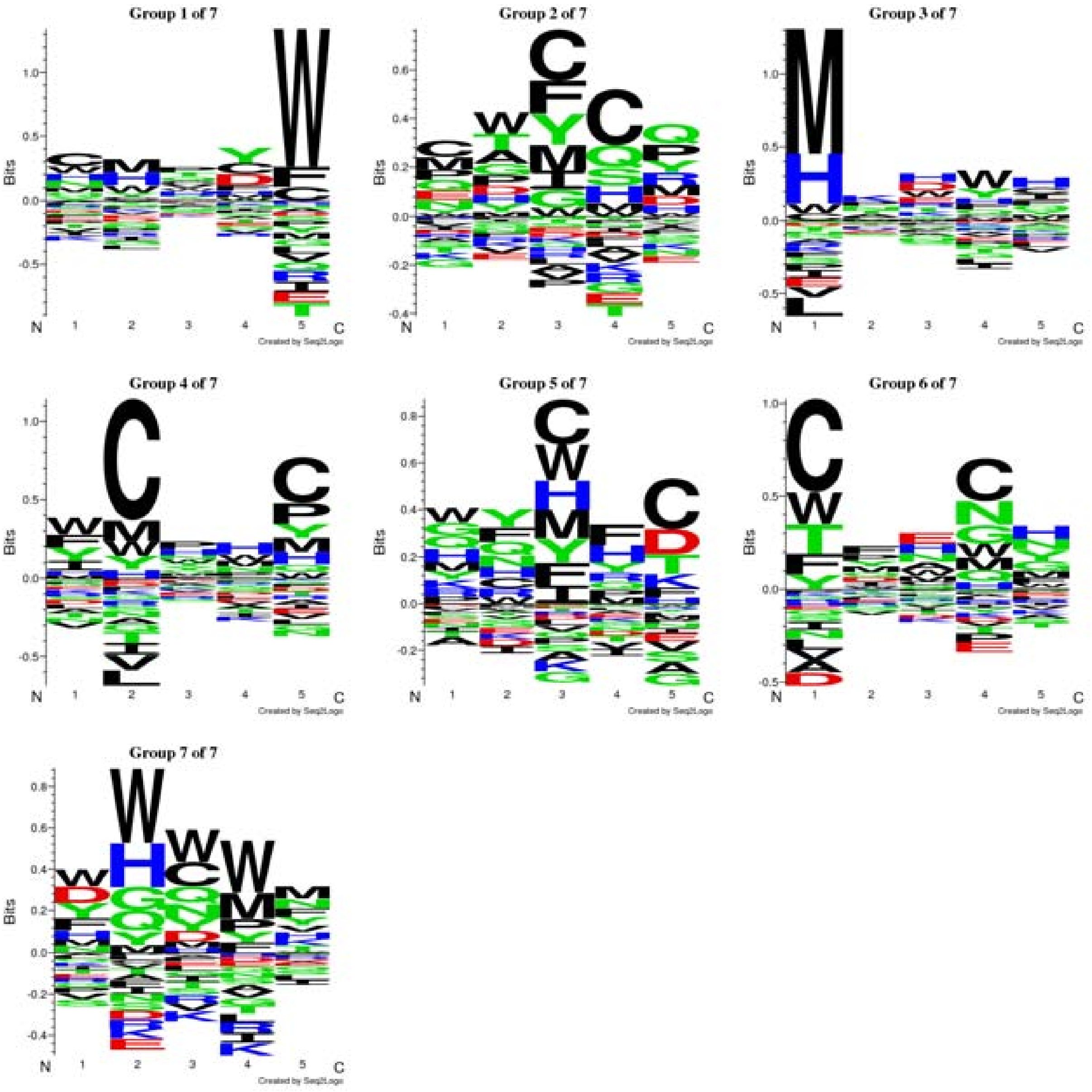

Supplement: Supplementary file 1 — Dataset 1 [file 41598_2018_33433_MOESM1_ESM.pdf]
